# Supplementary material for: Postmortem Stability Analysis of Lipids and Polar Metabolites in Human, Rat, and Mouse Brains
Source: Biomolecules. 2025 Sep 5;15(9):1288. doi: 10.3390/biom15091288 (PMC12467850; doi:10.3390/biom15091288)
Supplement: Supplementary file 1 [file Figure Sup All.pdf › Figure Sup All.pdf]

Figure S1. Normalized postmortem intensity changes of individual lipids in mouse, rat, and human brain tissue. Compound and species names are shown on top of the plot and the corresponding panels, respectively. Each line represents an individual donor (n=6 per species). Pearson's correlation coefficient (r) and significance (p-value) of the relationship between normalized lipid abundance levels and log_2-transformed postmortem interval duration are indicated within each panel.

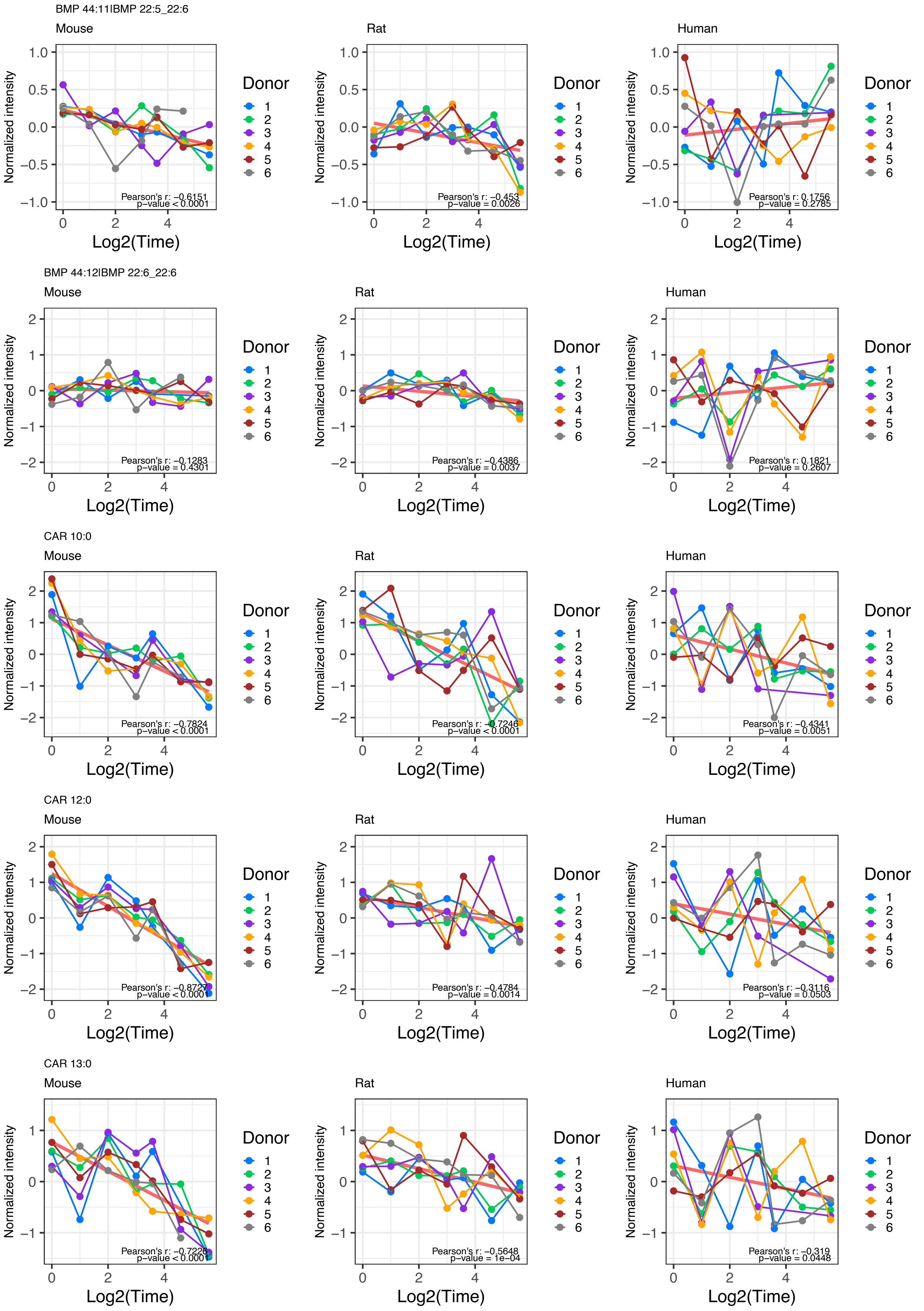
BMP 44:11|BMP 22:5_22:6
Mouse
Rat
Human
Normalized intensity
Log2(Time)
Donor
1
2
3
4
5
6
Pearson's r: −0.6151
p−value < 0.0001
Pearson's r: −0.453
p−value = 0.0026
Pearson's r: 0.1756
p−value = 0.2785
BMP 44:12|BMP 22:6_22:6
Pearson's r: −0.1283
p−value = 0.4301
Pearson's r: −0.4386
p−value = 0.0037
Pearson's r: 0.1821
p−value = 0.2607
CAR 10:0
Pearson's r: −0.7824
p−value < 0.0001
Pearson's r: −0.7246
p−value < 0.0001
Pearson's r: −0.4341
p−value = 0.0051
CAR 12:0
Pearson's r: −0.8727
p−value < 0.0001
Pearson's r: −0.4784
p−value = 0.0014
Pearson's r: −0.3116
p−value = 0.0503
CAR 13:0
Pearson's r: −0.7226
p−value < 0.0001
Pearson's r: −0.5648
p−value = 1e−04
Pearson's r: −0.319
p−value = 0.0448

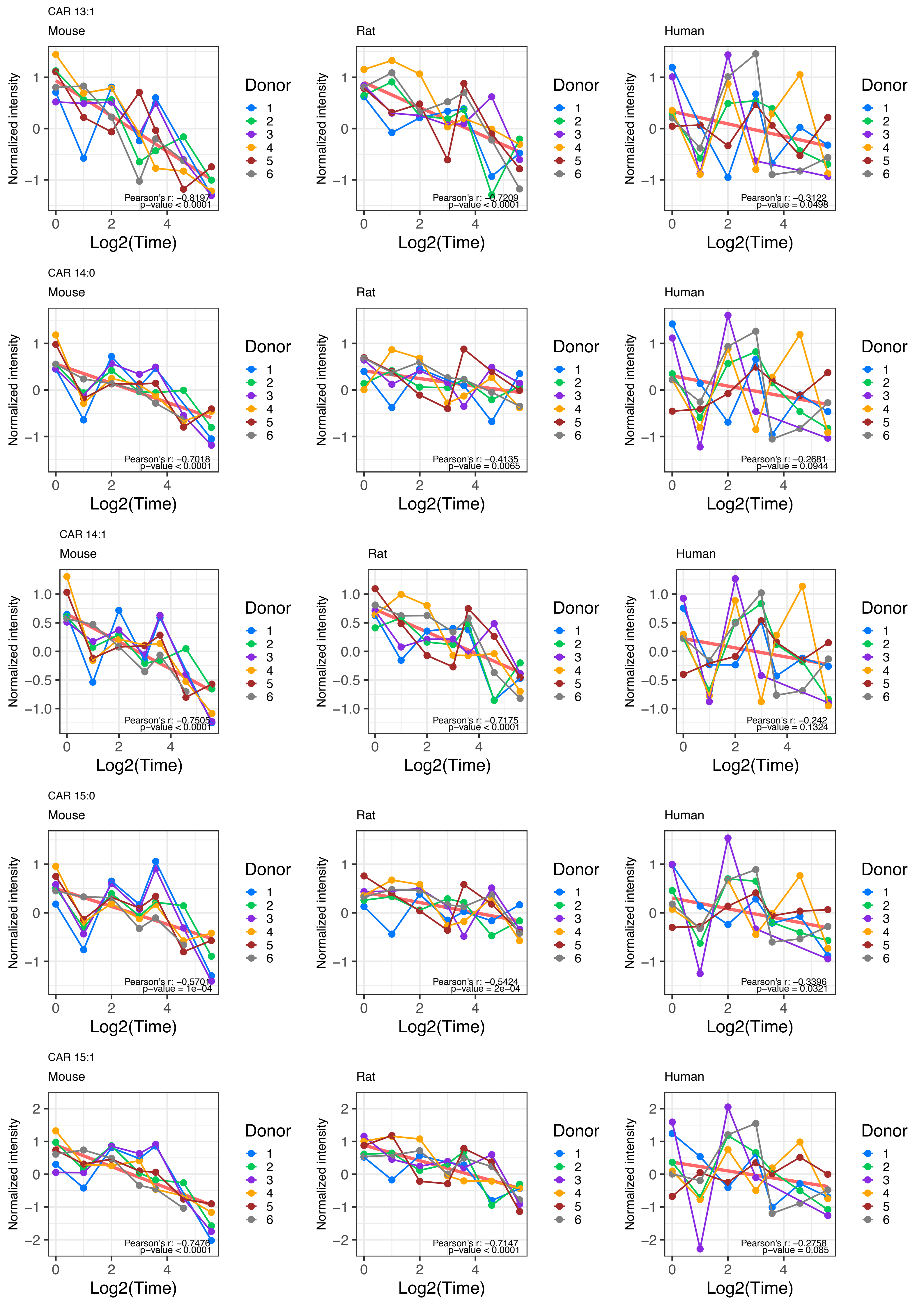

CAR 13:1
Mouse
Pearson's r: −0.8197
p−value < 0.0001
Rat
Pearson's r: −0.7209
p−value < 0.0001
Human
Pearson's r: −0.3122
p−value = 0.0498
CAR 14:0
Mouse
Pearson's r: −0.7018
p−value < 0.0001
Rat
Pearson's r: −0.4135
p−value = 0.0065
Human
Pearson's r: −0.2681
p−value = 0.0944
CAR 14:1
Mouse
Pearson's r: −0.7505
p−value < 0.0001
Rat
Pearson's r: −0.7175
p−value < 0.0001
Human
Pearson's r: −0.242
p−value = 0.1324
CAR 15:0
Mouse
Pearson's r: −0.5701
p−value = 1e−04
Rat
Pearson's r: −0.5424
p−value = 2e−04
Human
Pearson's r: −0.3396
p−value = 0.0321
CAR 15:1
Mouse
Pearson's r: −0.7476
p−value < 0.0001
Rat
Pearson's r: −0.7147
p−value < 0.0001
Human
Pearson's r: −0.2758
p−value = 0.085
Normalized intensity
Log2(Time)
Donor
1
2
3
4
5
6

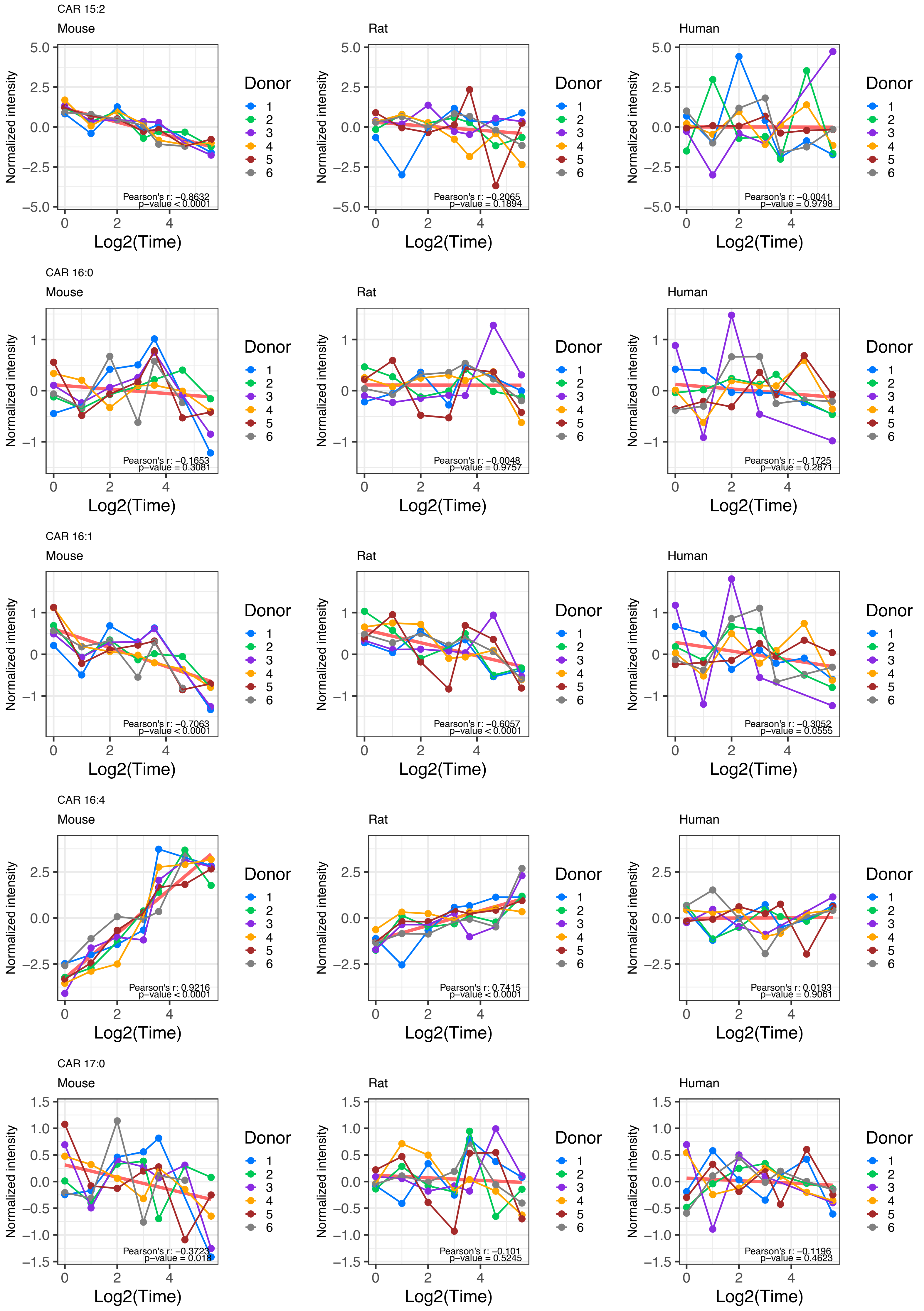
CAR 15:2
Mouse
Rat
Human
Normalized intensity
Log2(Time)
Donor
1
2
3
4
5
6
Pearson's r: −0.8632
p−value < 0.0001
Pearson's r: −0.2065
p−value = 0.1894
Pearson's r: −0.0041
p−value = 0.9798
CAR 16:0
Mouse
Rat
Human
Pearson's r: −0.1653
p−value = 0.3081
Pearson's r: −0.0048
p−value = 0.9757
Pearson's r: −0.1725
p−value = 0.2871
CAR 16:1
Mouse
Rat
Human
Pearson's r: −0.7063
p−value < 0.0001
Pearson's r: −0.6057
p−value < 0.0001
Pearson's r: −0.3052
p−value = 0.0555
CAR 16:4
Mouse
Rat
Human
Pearson's r: 0.9216
p−value < 0.0001
Pearson's r: 0.7415
p−value < 0.0001
Pearson's r: 0.0193
p−value = 0.9061
CAR 17:0
Mouse
Rat
Human
Pearson's r: −0.3723
p−value = 0.018
Pearson's r: −0.101
p−value = 0.5245
Pearson's r: −0.1196
p−value = 0.4623

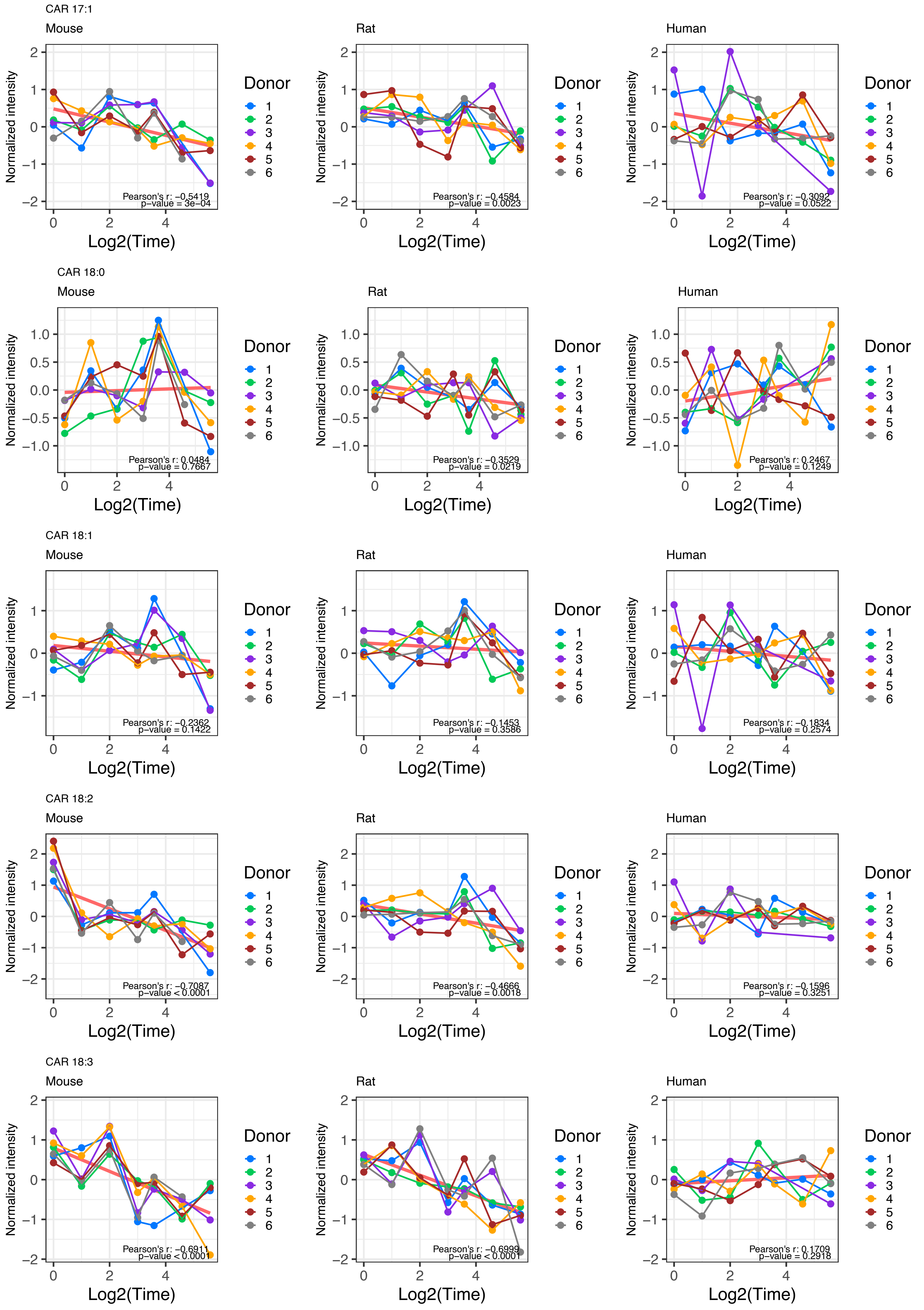
CAR 17:1
Mouse
Pearson's r: −0.5419
p−value = 3e−04
Rat
Pearson's r: −0.4584
p−value = 0.0023
Human
Pearson's r: −0.3092
p−value = 0.0522
CAR 18:0
Mouse
Pearson's r: 0.0484
p−value = 0.7667
Rat
Pearson's r: −0.3529
p−value = 0.0219
Human
Pearson's r: 0.2467
p−value = 0.1249
CAR 18:1
Mouse
Pearson's r: −0.2362
p−value = 0.1422
Rat
Pearson's r: −0.1453
p−value = 0.3586
Human
Pearson's r: −0.1834
p−value = 0.2574
CAR 18:2
Mouse
Pearson's r: −0.7087
p−value < 0.0001
Rat
Pearson's r: −0.4666
p−value = 0.0018
Human
Pearson's r: −0.1596
p−value = 0.3251
CAR 18:3
Mouse
Pearson's r: −0.6911
p−value < 0.0001
Rat
Pearson's r: −0.6999
p−value < 0.0001
Human
Pearson's r: 0.1709
p−value = 0.2918
Normalized intensity
Log2(Time)
Donor
1
2
3
4
5
6

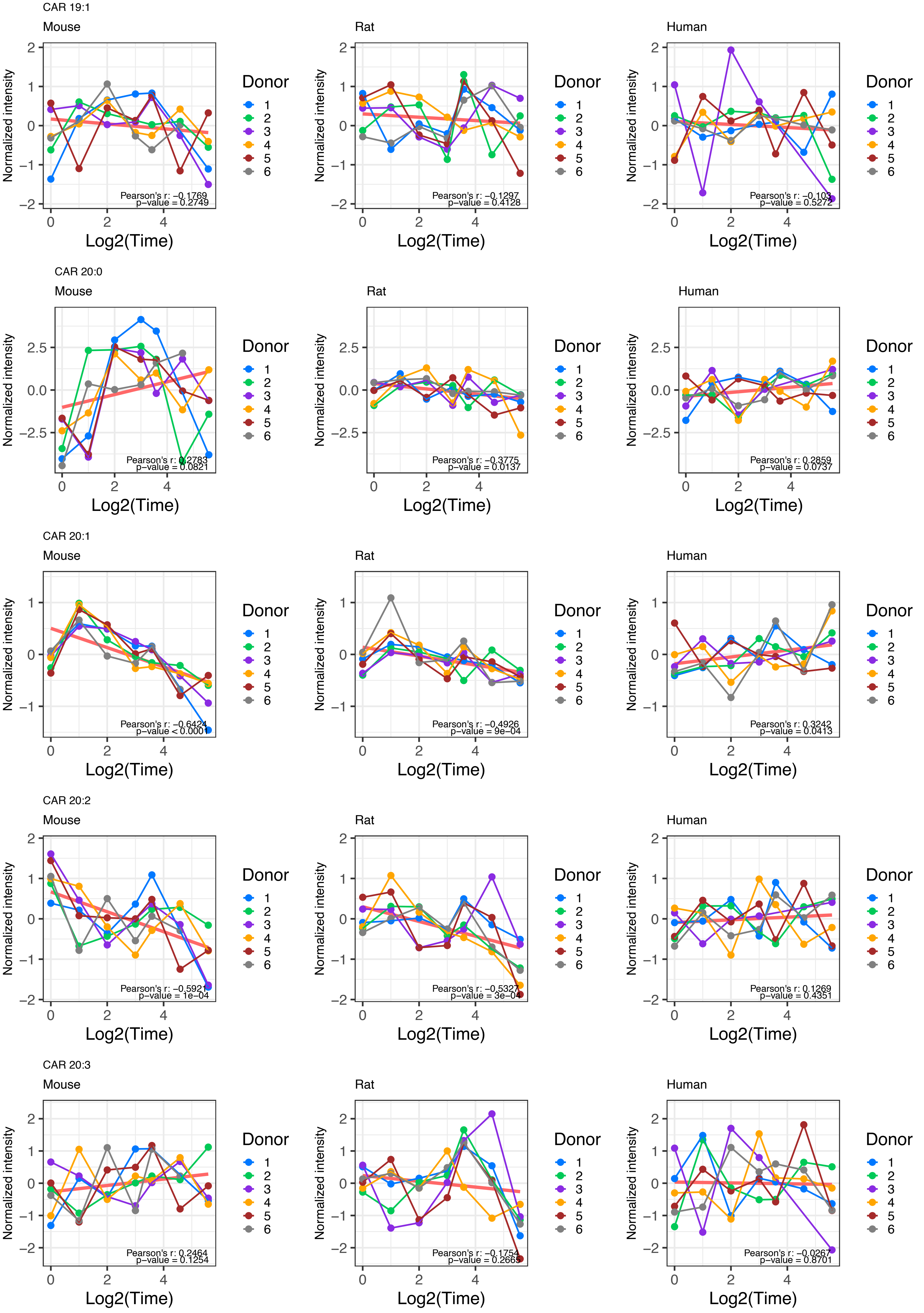
CAR 19:1
Mouse
Pearson's r: −0.1769
p−value = 0.2749
Rat
Pearson's r: −0.1297
p−value = 0.4128
Human
Pearson's r: −0.103
p−value = 0.5272
CAR 20:0
Mouse
Pearson's r: 0.2783
p−value = 0.0821
Rat
Pearson's r: −0.3775
p−value = 0.0137
Human
Pearson's r: 0.2859
p−value = 0.0737
CAR 20:1
Mouse
Pearson's r: −0.6424
p−value < 0.0001
Rat
Pearson's r: −0.4926
p−value = 9e−04
Human
Pearson's r: 0.3242
p−value = 0.0413
CAR 20:2
Mouse
Pearson's r: −0.5921
p−value = 1e−04
Rat
Pearson's r: −0.5327
p−value = 3e−04
Human
Pearson's r: 0.1269
p−value = 0.4351
CAR 20:3
Mouse
Pearson's r: 0.2464
p−value = 0.1254
Rat
Pearson's r: −0.1754
p−value = 0.2665
Human
Pearson's r: −0.0267
p−value = 0.8701
Normalized intensity
Log2(Time)
Donor
1
2
3
4
5
6

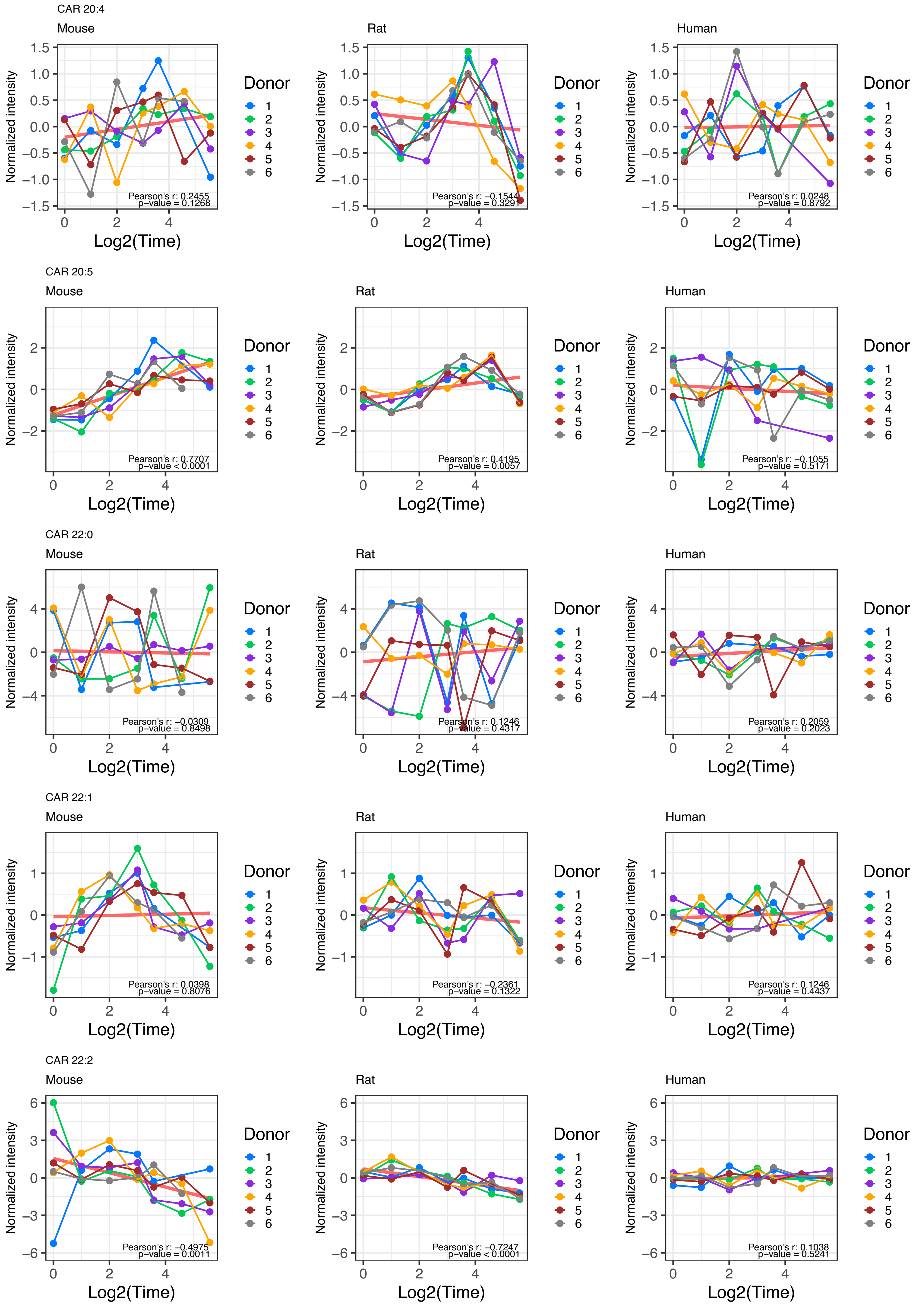
CAR 20:4
Mouse
Rat
Human
Normalized intensity
Log2(Time)
Donor
1
2
3
4
5
6
Pearson's r: 0.2455
p−value = 0.1268
Pearson's r: −0.1544
p−value = 0.3291
Pearson's r: 0.0248
p−value = 0.8792
CAR 20:5
Pearson's r: 0.7707
p−value < 0.0001
Pearson's r: 0.4195
p−value = 0.0057
Pearson's r: −0.1055
p−value = 0.5171
CAR 22:0
Pearson's r: −0.0309
p−value = 0.8498
Pearson's r: 0.1246
p−value = 0.4317
Pearson's r: 0.2059
p−value = 0.2023
CAR 22:1
Pearson's r: 0.0398
p−value = 0.8076
Pearson's r: −0.2361
p−value = 0.1322
Pearson's r: 0.1246
p−value = 0.4437
CAR 22:2
Pearson's r: −0.4975
p−value = 0.0011
Pearson's r: −0.7247
p−value < 0.0001
Pearson's r: 0.1038
p−value = 0.5241

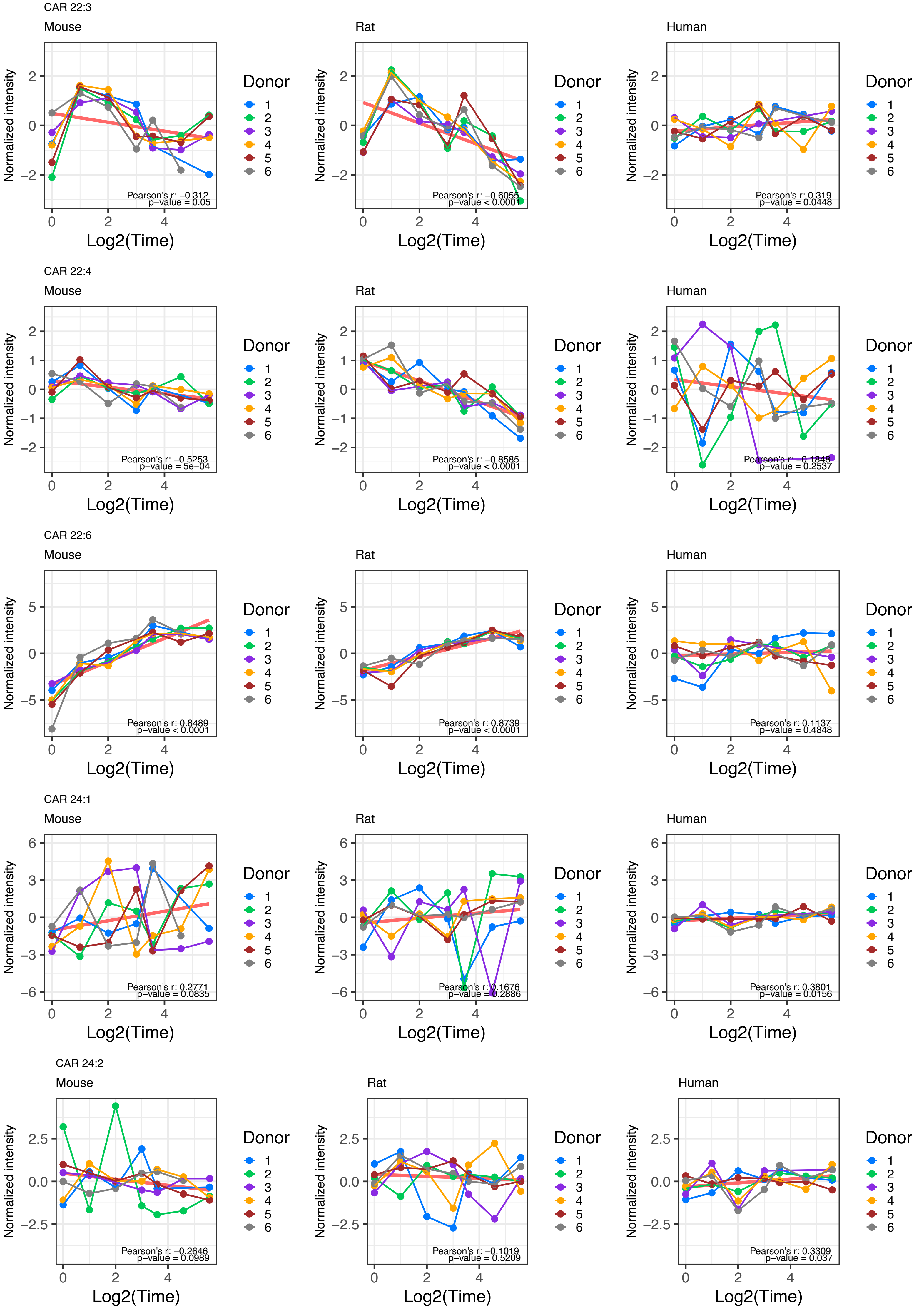
CAR 22:3
Mouse
Pearson's r: −0.312
p−value = 0.05
Rat
Pearson's r: −0.6055
p−value < 0.0001
Human
Pearson's r: 0.319
p−value = 0.0448
CAR 22:4
Mouse
Pearson's r: −0.5253
p−value = 5e−04
Rat
Pearson's r: −0.8585
p−value < 0.0001
Human
Pearson's r: −0.1848
p−value = 0.2537
CAR 22:6
Mouse
Pearson's r: 0.8489
p−value < 0.0001
Rat
Pearson's r: 0.8739
p−value < 0.0001
Human
Pearson's r: 0.1137
p−value = 0.4848
CAR 24:1
Mouse
Pearson's r: 0.2771
p−value = 0.0835
Rat
Pearson's r: 0.1676
p−value = 0.2886
Human
Pearson's r: 0.3801
p−value = 0.0156
CAR 24:2
Mouse
Pearson's r: −0.2646
p−value = 0.0989
Rat
Pearson's r: −0.1019
p−value = 0.5209
Human
Pearson's r: 0.3309
p−value = 0.037
Normalized intensity
Log2(Time)
Donor
1
2
3
4
5
6

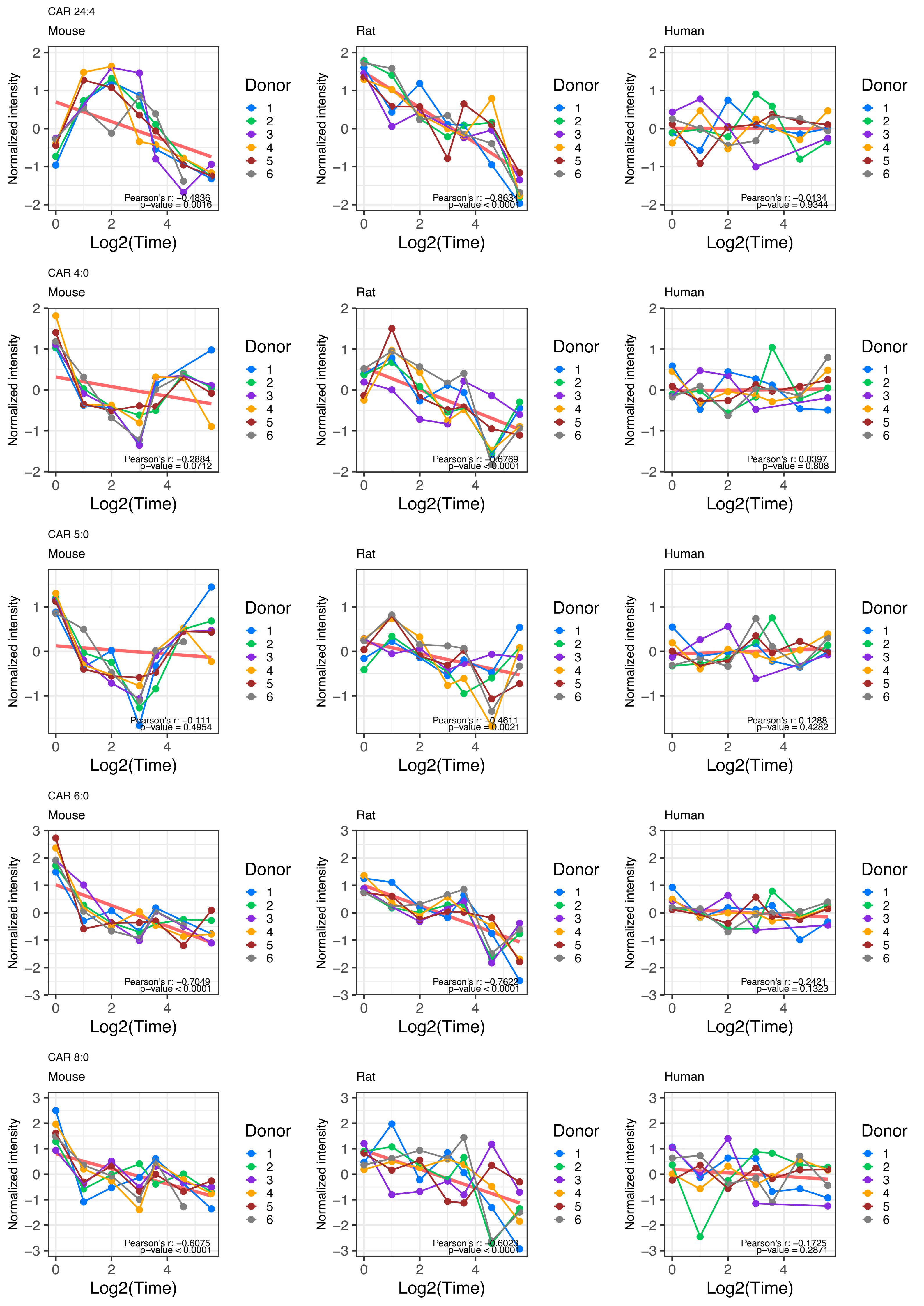
CAR 24:4
Mouse
Pearson's r: −0.4836
p−value = 0.0016
Rat
Pearson's r: −0.8634
p−value < 0.0001
Human
Pearson's r: −0.0134
p−value = 0.9344
CAR 4:0
Mouse
Pearson's r: −0.2884
p−value = 0.0712
Rat
Pearson's r: −0.6769
p−value < 0.0001
Human
Pearson's r: 0.0397
p−value = 0.808
CAR 5:0
Mouse
Pearson's r: −0.111
p−value = 0.4954
Rat
Pearson's r: −0.4611
p−value = 0.0021
Human
Pearson's r: 0.1288
p−value = 0.4282
CAR 6:0
Mouse
Pearson's r: −0.7049
p−value < 0.0001
Rat
Pearson's r: −0.7622
p−value < 0.0001
Human
Pearson's r: −0.2421
p−value = 0.1323
CAR 8:0
Mouse
Pearson's r: −0.6075
p−value < 0.0001
Rat
Pearson's r: −0.6023
p−value < 0.0001
Human
Pearson's r: −0.1725
p−value = 0.2871
Normalized intensity
Log2(Time)
Donor
1
2
3
4
5
6

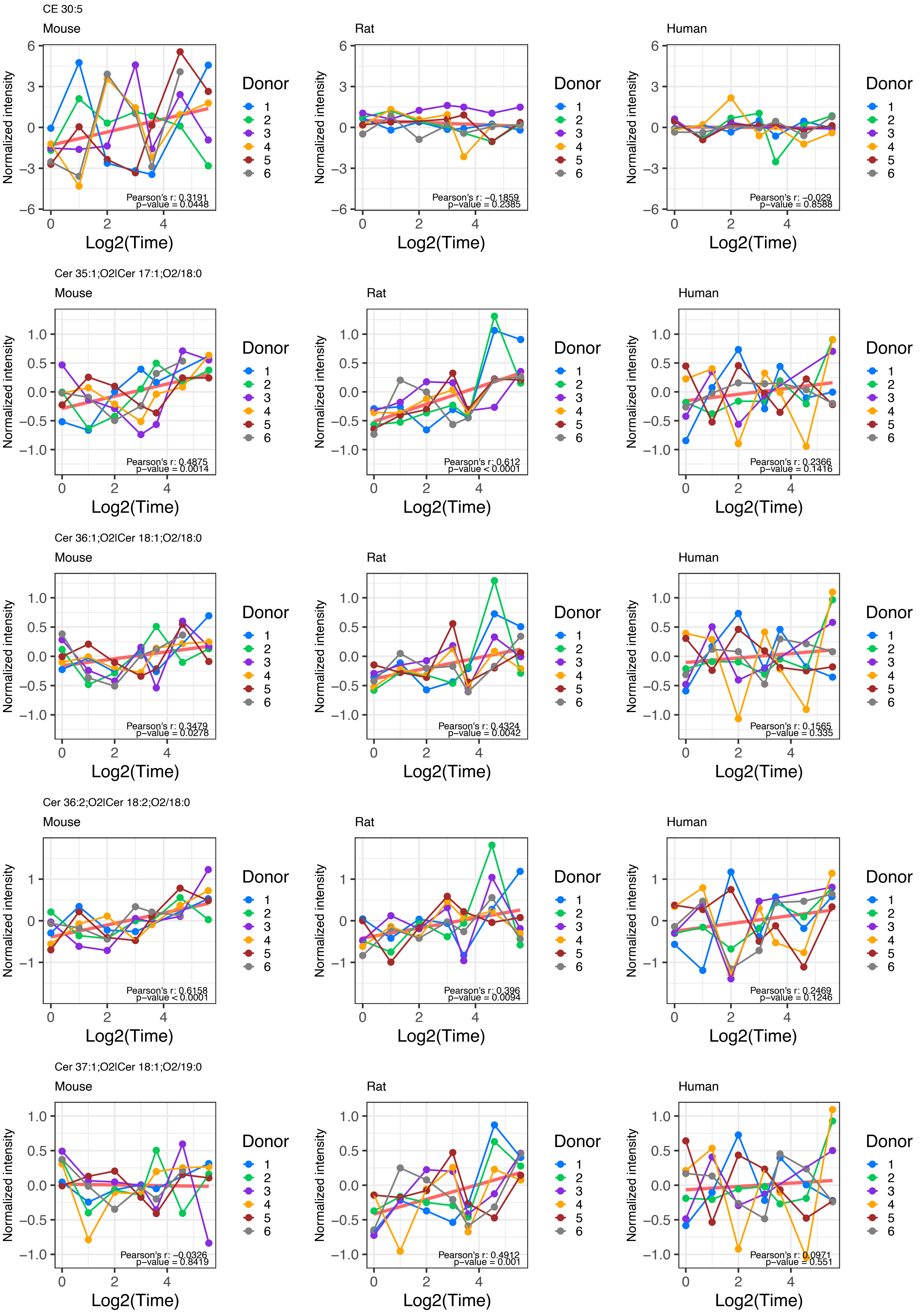
CE 30:5
Mouse
Pearson's r: 0.3191
p−value = 0.0448
Rat
Pearson's r: −0.1859
p−value = 0.2385
Human
Pearson's r: −0.029
p−value = 0.8588
Cer 35:1;O2|Cer 17:1;O2/18:0
Mouse
Pearson's r: 0.4875
p−value = 0.0014
Rat
Pearson's r: 0.612
p−value < 0.0001
Human
Pearson's r: 0.2366
p−value = 0.1416
Cer 36:1;O2|Cer 18:1;O2/18:0
Mouse
Pearson's r: 0.3479
p−value = 0.0278
Rat
Pearson's r: 0.4324
p−value = 0.0042
Human
Pearson's r: 0.1565
p−value = 0.335
Cer 36:2;O2|Cer 18:2;O2/18:0
Mouse
Pearson's r: 0.6158
p−value < 0.0001
Rat
Pearson's r: 0.396
p−value = 0.0094
Human
Pearson's r: 0.2469
p−value = 0.1246
Cer 37:1;O2|Cer 18:1;O2/19:0
Mouse
Pearson's r: −0.0326
p−value = 0.8419
Rat
Pearson's r: 0.4912
p−value = 0.001
Human
Pearson's r: 0.0971
p−value = 0.551
Normalized intensity
Log2(Time)
Donor
1
2
3
4
5
6

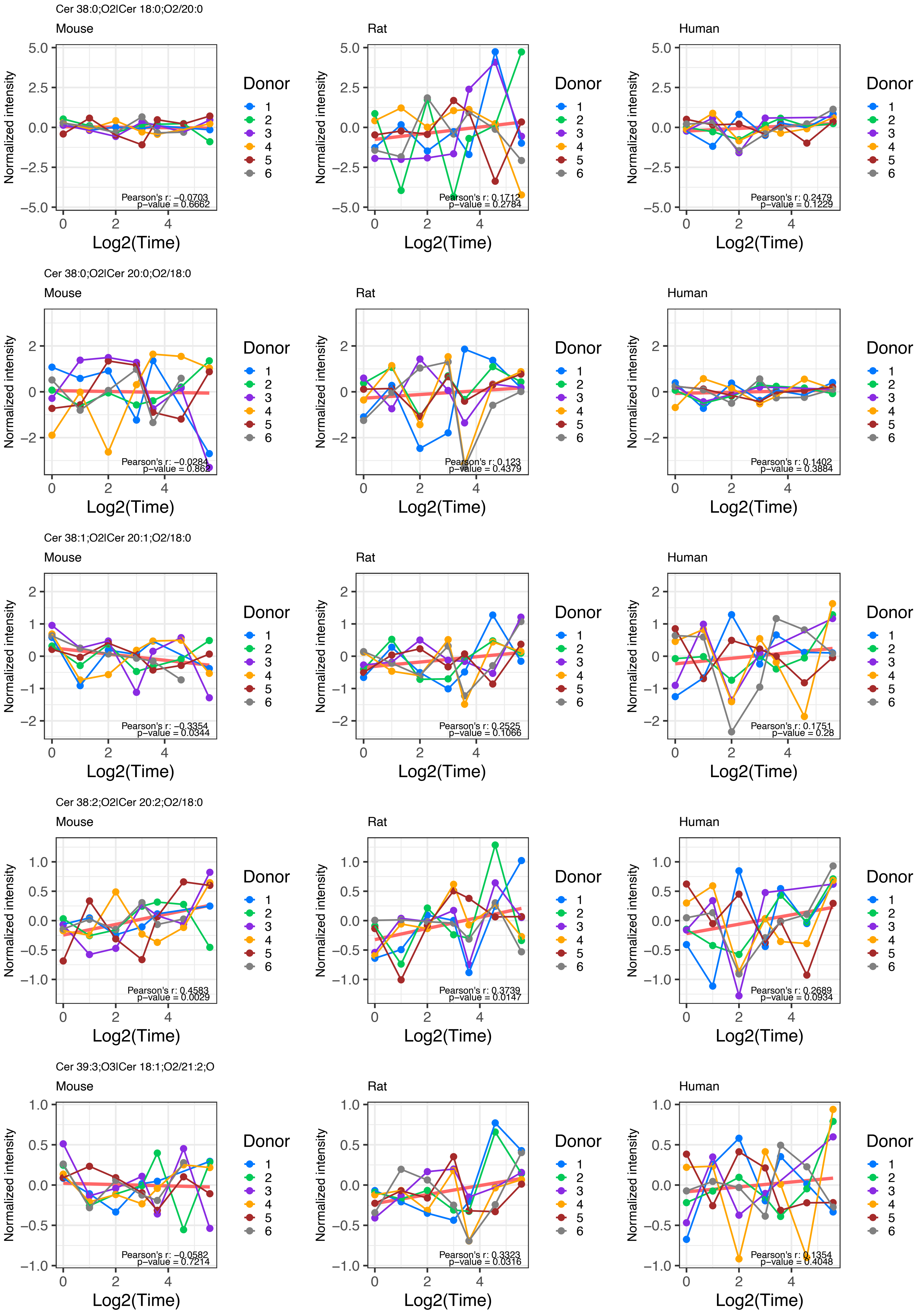
Cer 38:0;O2|Cer 18:0;O2/20:0
Mouse
Pearson's r: −0.0703
p−value = 0.6662
Rat
Pearson's r: 0.1712
p−value = 0.2784
Human
Pearson's r: 0.2479
p−value = 0.1229
Cer 38:0;O2|Cer 20:0;O2/18:0
Mouse
Pearson's r: −0.0284
p−value = 0.862
Rat
Pearson's r: 0.123
p−value = 0.4379
Human
Pearson's r: 0.1402
p−value = 0.3884
Cer 38:1;O2|Cer 20:1;O2/18:0
Mouse
Pearson's r: −0.3354
p−value = 0.0344
Rat
Pearson's r: 0.2525
p−value = 0.1066
Human
Pearson's r: 0.1751
p−value = 0.28
Cer 38:2;O2|Cer 20:2;O2/18:0
Mouse
Pearson's r: 0.4583
p−value = 0.0029
Rat
Pearson's r: 0.3739
p−value = 0.0147
Human
Pearson's r: 0.2689
p−value = 0.0934
Cer 39:3;O3|Cer 18:1;O2/21:2;O
Mouse
Pearson's r: −0.0582
p−value = 0.7214
Rat
Pearson's r: 0.3323
p−value = 0.0316
Human
Pearson's r: 0.1354
p−value = 0.4048
Normalized intensity
Log2(Time)
Donor
1
2
3
4
5
6

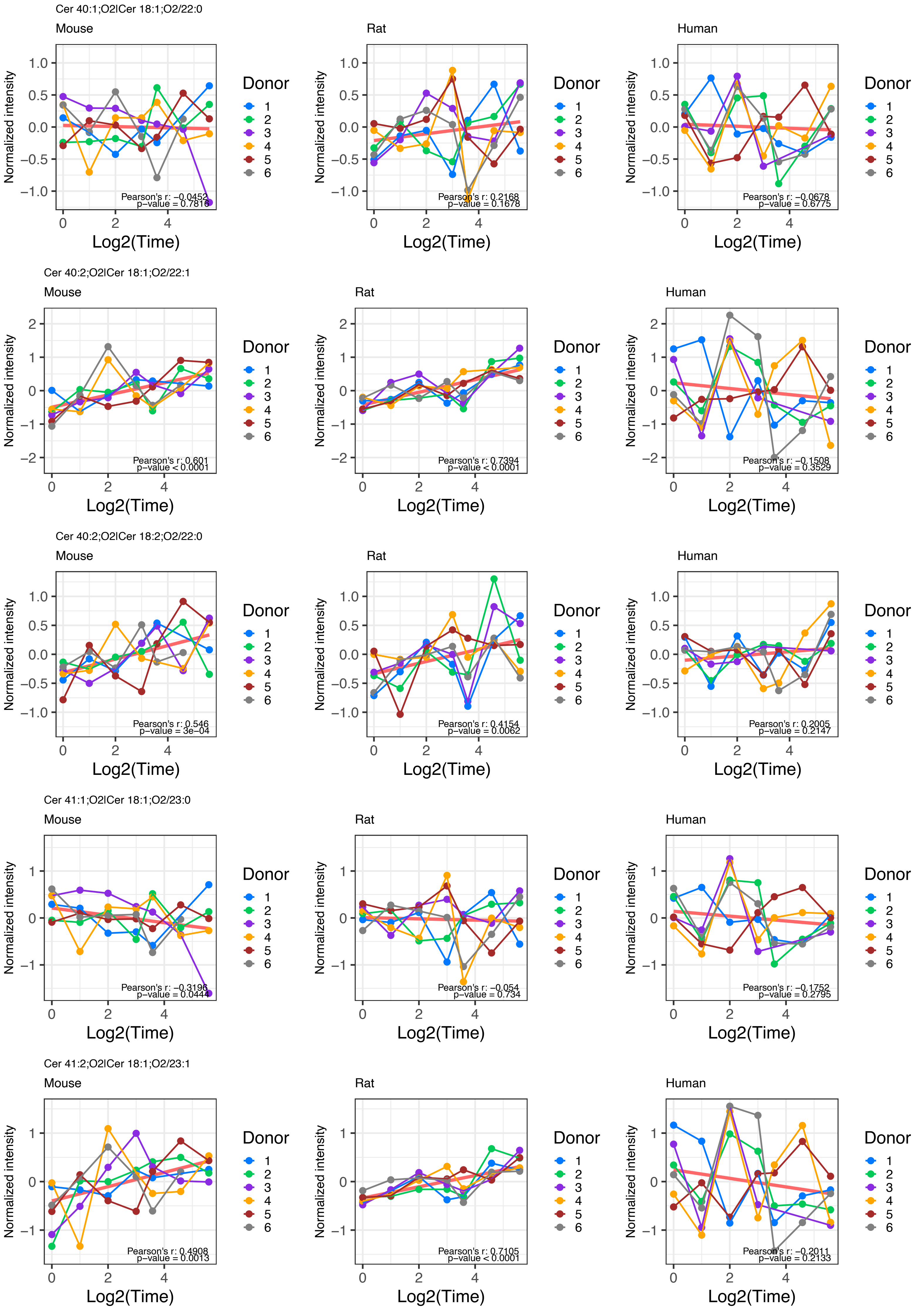

Cer 40:1;O2|Cer 18:1;O2/22:0
Mouse
Pearson's r: −0.0452
p−value = 0.7816
Rat
Pearson's r: 0.2168
p−value = 0.1678
Human
Pearson's r: −0.0678
p−value = 0.6775
Cer 40:2;O2|Cer 18:1;O2/22:1
Mouse
Pearson's r: 0.601
p−value < 0.0001
Rat
Pearson's r: 0.7394
p−value < 0.0001
Human
Pearson's r: −0.1508
p−value = 0.3529
Cer 40:2;O2|Cer 18:2;O2/22:0
Mouse
Pearson's r: 0.546
p−value = 3e−04
Rat
Pearson's r: 0.4154
p−value = 0.0062
Human
Pearson's r: 0.2005
p−value = 0.2147
Cer 41:1;O2|Cer 18:1;O2/23:0
Mouse
Pearson's r: −0.3196
p−value = 0.0444
Rat
Pearson's r: −0.054
p−value = 0.734
Human
Pearson's r: −0.1752
p−value = 0.2795
Cer 41:2;O2|Cer 18:1;O2/23:1
Mouse
Pearson's r: 0.4908
p−value = 0.0013
Rat
Pearson's r: 0.7105
p−value < 0.0001
Human
Pearson's r: −0.2011
p−value = 0.2133
Normalized intensity
Log2(Time)
Donor
1
2
3
4
5
6

Cer 41:2;O2|Cer 18:1;O2/23:1

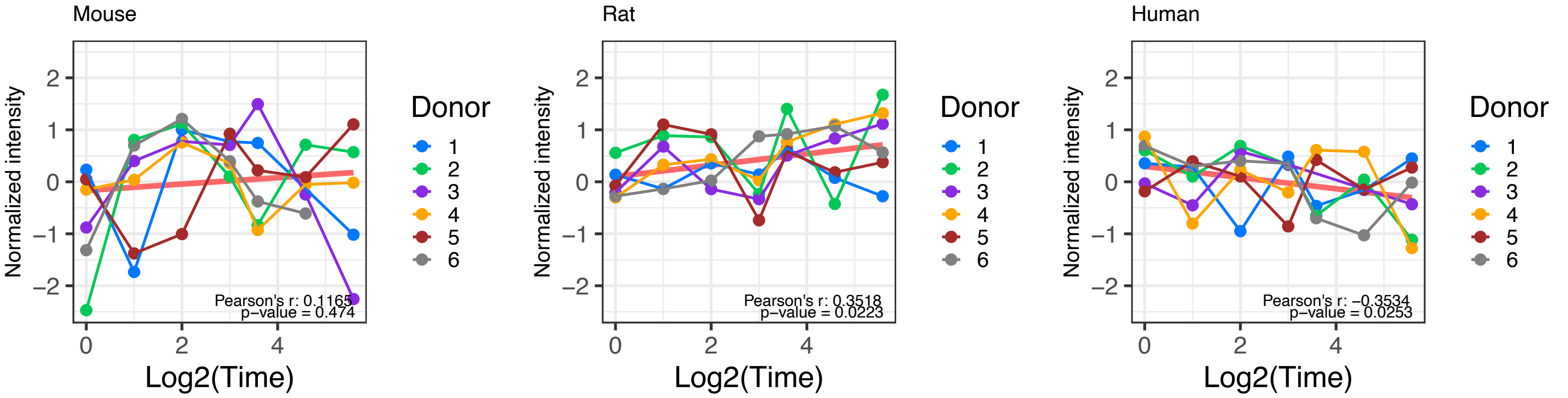

Cer 42:1;O2|Cer 18:1;O2/24:0

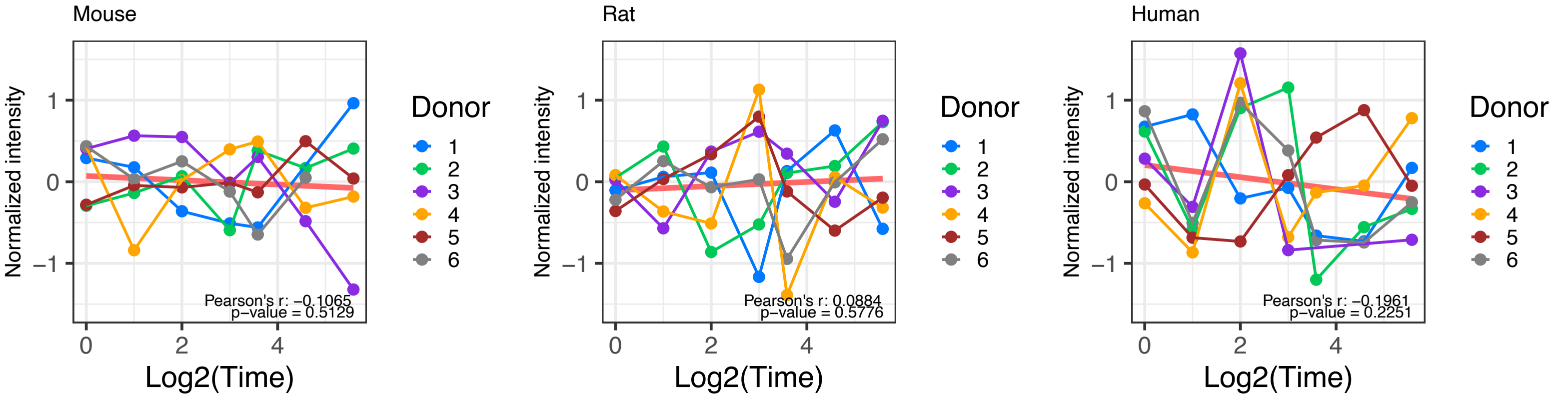

Cer 42:1;O3|Cer 18:1;O2/24:0;O

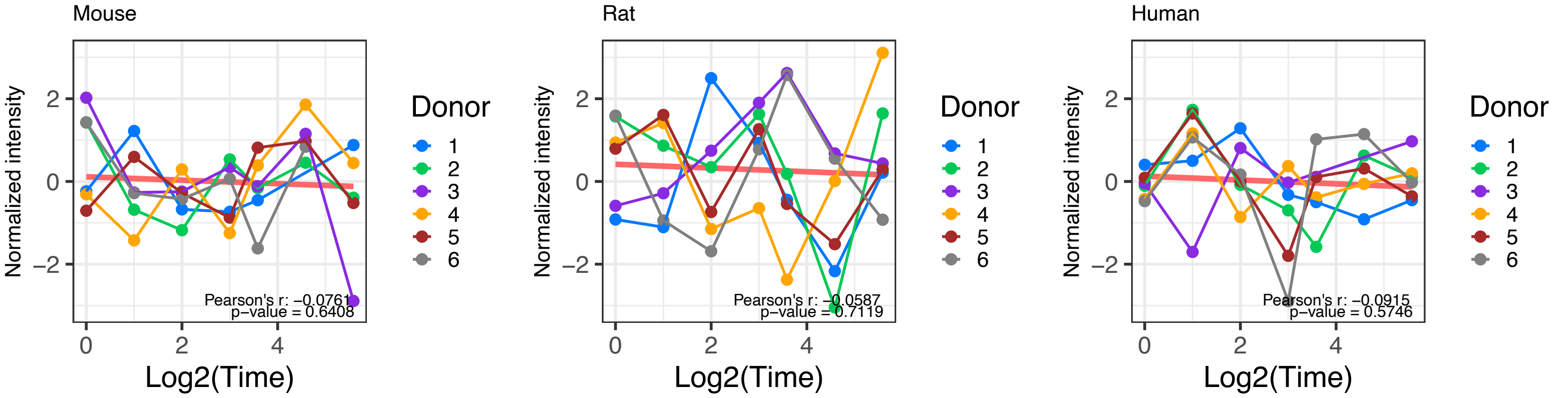

Cer 42:2;O2|Cer 18:1;O2/24:1

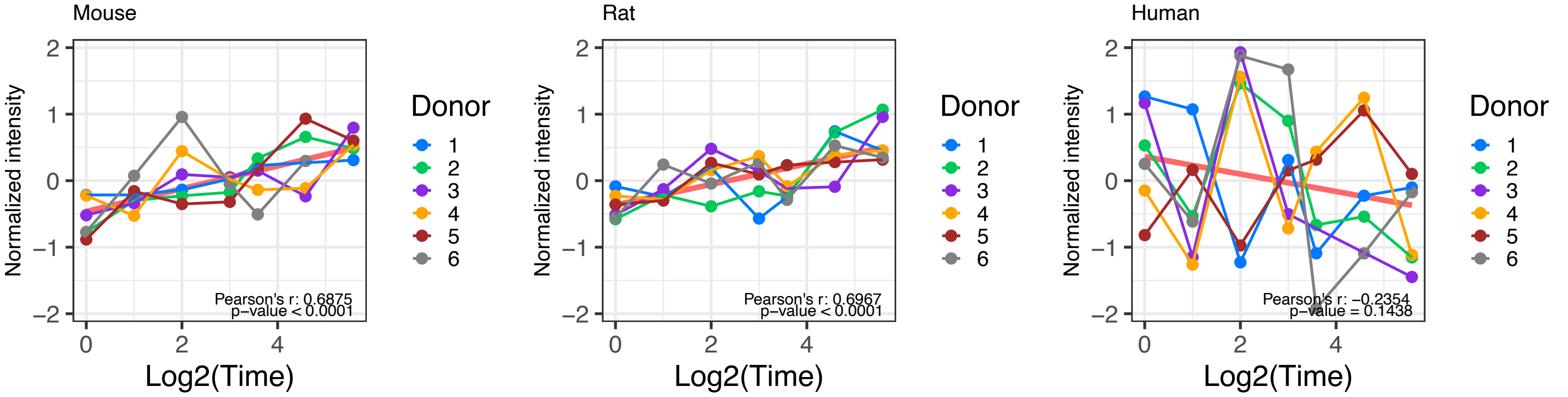

Cer 42:2;O2|Cer 18:2;O2/24:0

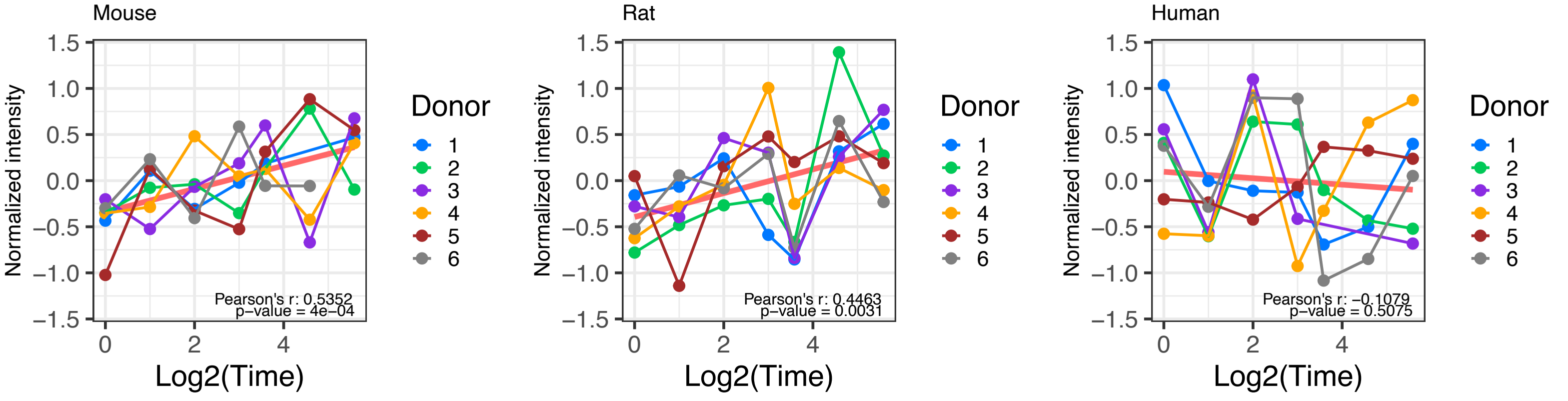

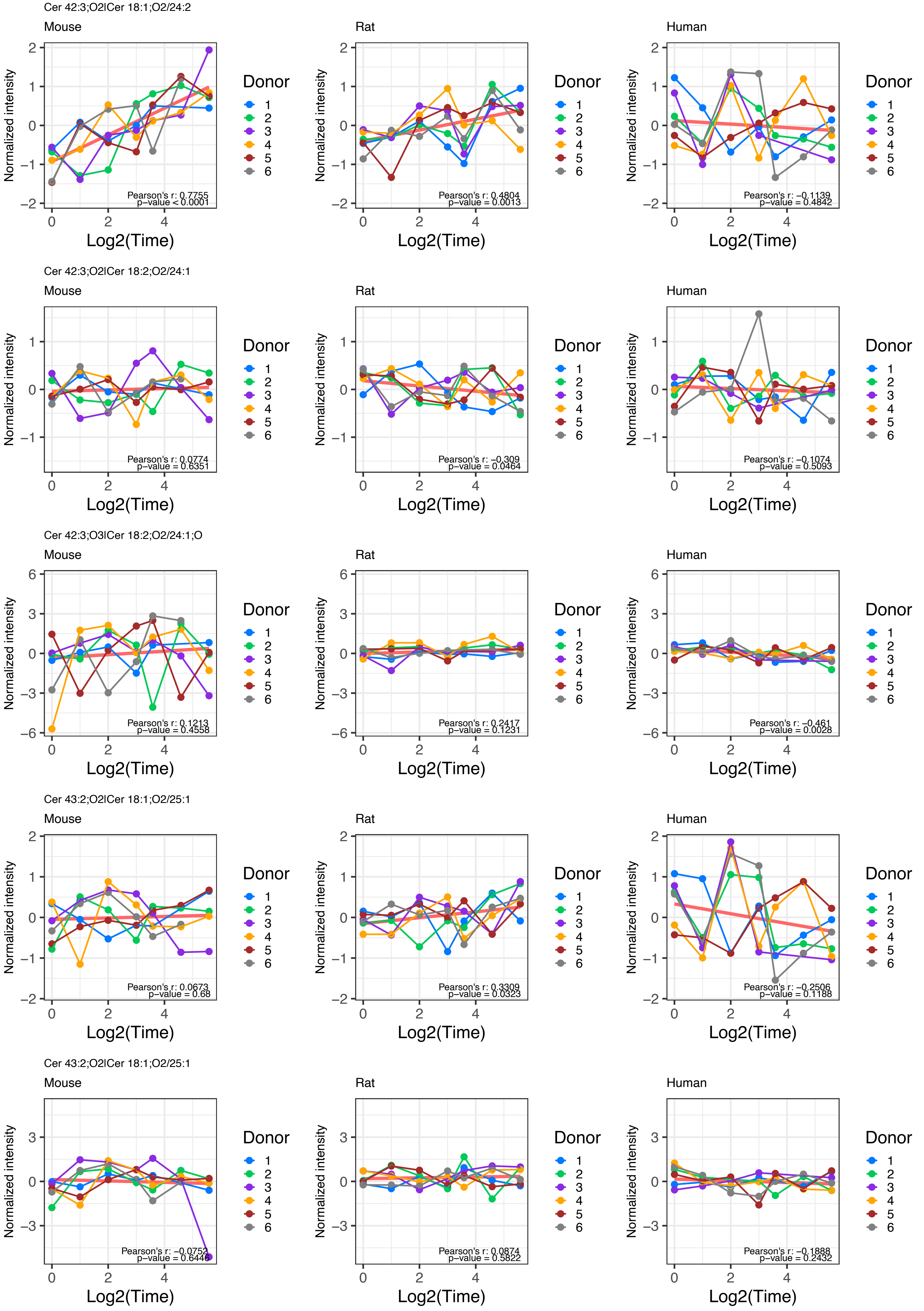

Cer 42:3;O2|Cer 18:1;O2/24:2
Mouse
Pearson's r: 0.7755
p−value < 0.0001
Rat
Pearson's r: 0.4804
p−value = 0.0013
Human
Pearson's r: −0.1139
p−value = 0.4842
Cer 42:3;O2|Cer 18:2;O2/24:1
Mouse
Pearson's r: 0.0774
p−value = 0.6351
Rat
Pearson's r: −0.309
p−value = 0.0464
Human
Pearson's r: −0.1074
p−value = 0.5093
Cer 42:3;O3|Cer 18:2;O2/24:1;O
Mouse
Pearson's r: 0.1213
p−value = 0.4558
Rat
Pearson's r: 0.2417
p−value = 0.1231
Human
Pearson's r: −0.461
p−value = 0.0028
Cer 43:2;O2|Cer 18:1;O2/25:1
Mouse
Pearson's r: 0.0673
p−value = 0.68
Rat
Pearson's r: 0.3309
p−value = 0.0323
Human
Pearson's r: −0.2506
p−value = 0.1188
Cer 43:2;O2|Cer 18:1;O2/25:1
Mouse
Pearson's r: −0.0752
p−value = 0.6446
Rat
Pearson's r: 0.0874
p−value = 0.5822
Human
Pearson's r: −0.1888
p−value = 0.2432
Normalized intensity
Log2(Time)
Donor
1
2
3
4
5
6

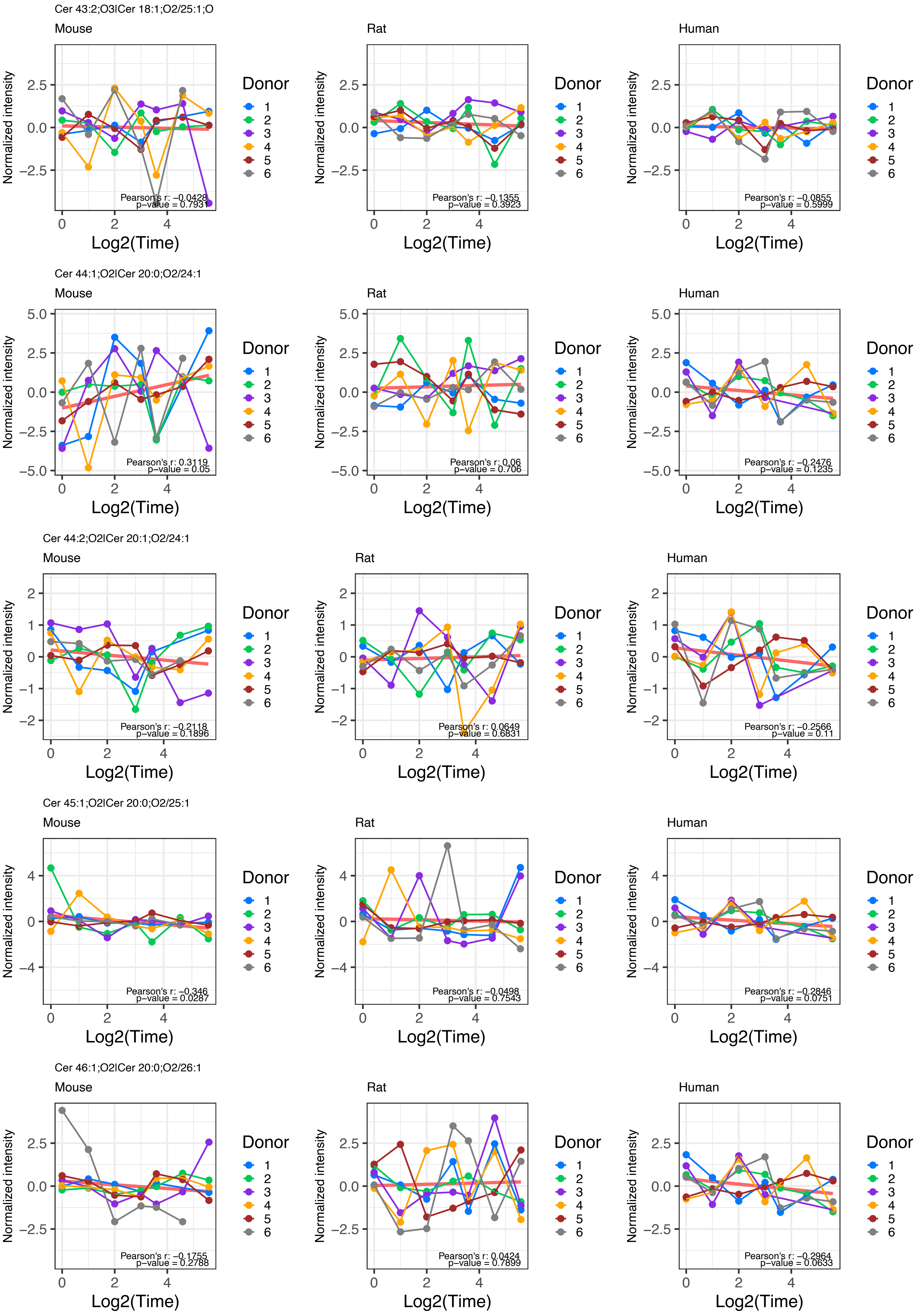

Cer 43:2;O3|Cer 18:1;O2/25:1;O
Mouse
Pearson's r: −0.0428
p−value = 0.7931
Rat
Pearson's r: −0.1355
p−value = 0.3923
Human
Pearson's r: −0.0855
p−value = 0.5999
Cer 44:1;O2|Cer 20:0;O2/24:1
Mouse
Pearson's r: 0.3119
p−value = 0.05
Rat
Pearson's r: 0.06
p−value = 0.706
Human
Pearson's r: −0.2476
p−value = 0.1235
Cer 44:2;O2|Cer 20:1;O2/24:1
Mouse
Pearson's r: −0.2118
p−value = 0.1896
Rat
Pearson's r: 0.0649
p−value = 0.6831
Human
Pearson's r: −0.2566
p−value = 0.11
Cer 45:1;O2|Cer 20:0;O2/25:1
Mouse
Pearson's r: −0.346
p−value = 0.0287
Rat
Pearson's r: −0.0498
p−value = 0.7543
Human
Pearson's r: −0.2846
p−value = 0.0751
Cer 46:1;O2|Cer 20:0;O2/26:1
Mouse
Pearson's r: −0.1755
p−value = 0.2788
Rat
Pearson's r: 0.0424
p−value = 0.7899
Human
Pearson's r: −0.2964
p−value = 0.0633
Normalized intensity
Log2(Time)
Donor
1
2
3
4
5
6

CL 70:7|CL 16:1_18:1_16:1_20:4

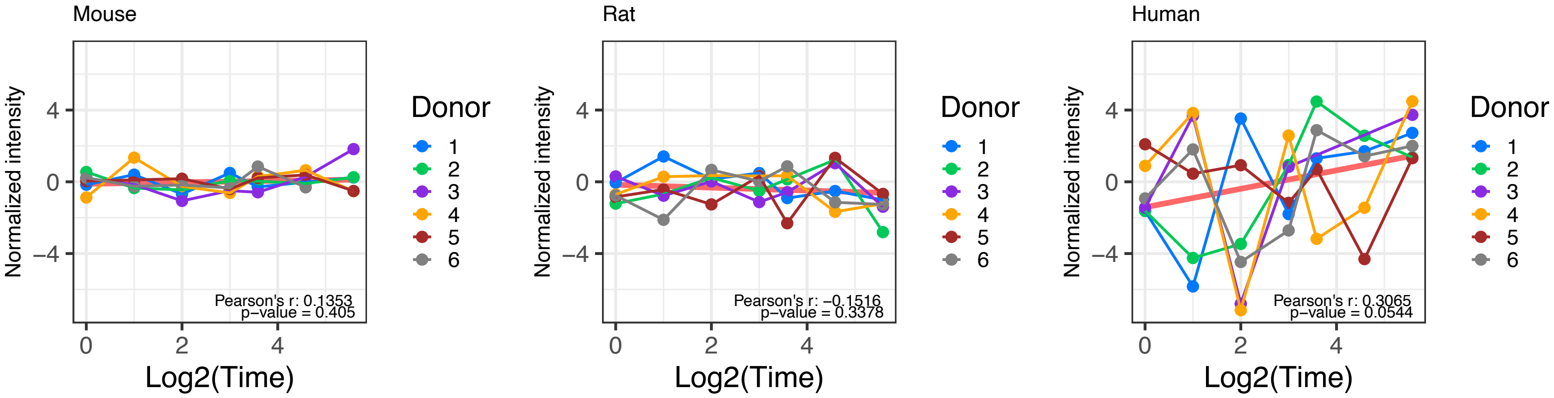

CL 72:10|CL 16:1_20:4_16:1_20:4

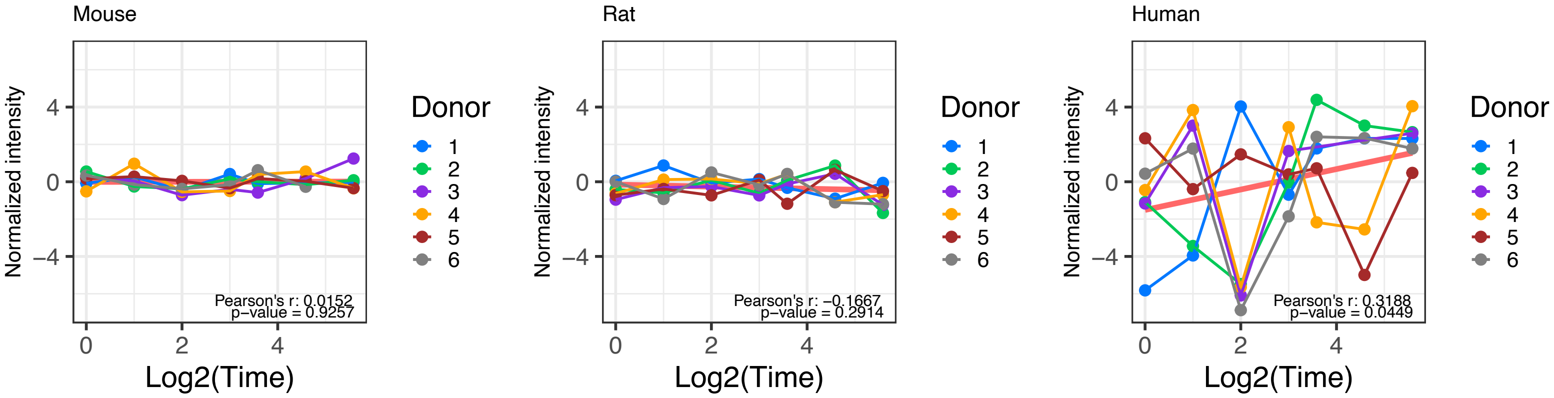

CL 72:7|CL 16:1_18:1_18:1_20:4

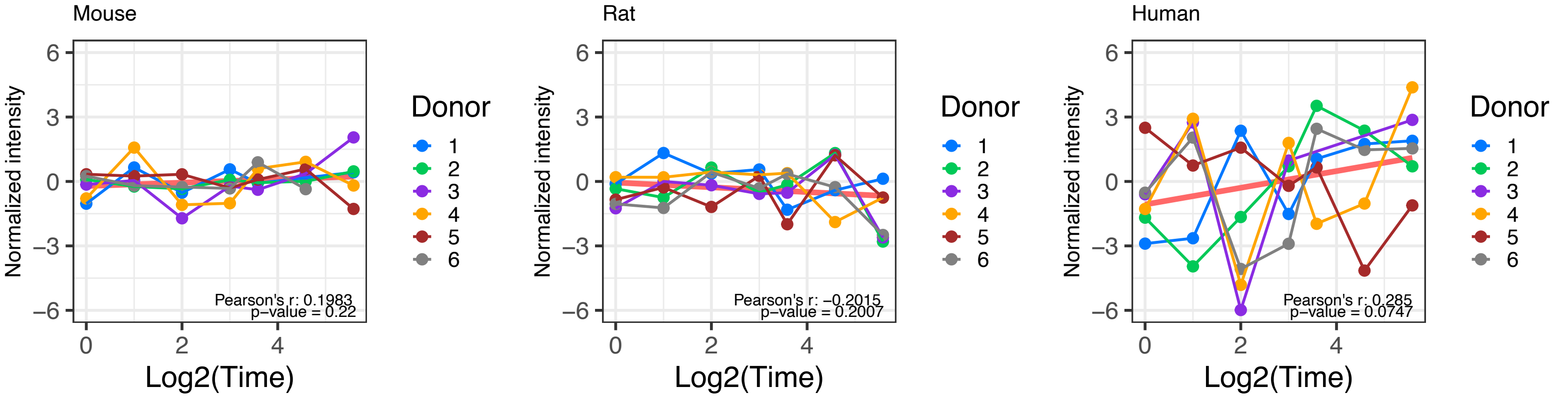

CL 72:8|CL 16:1_18:1_18:2_20:4

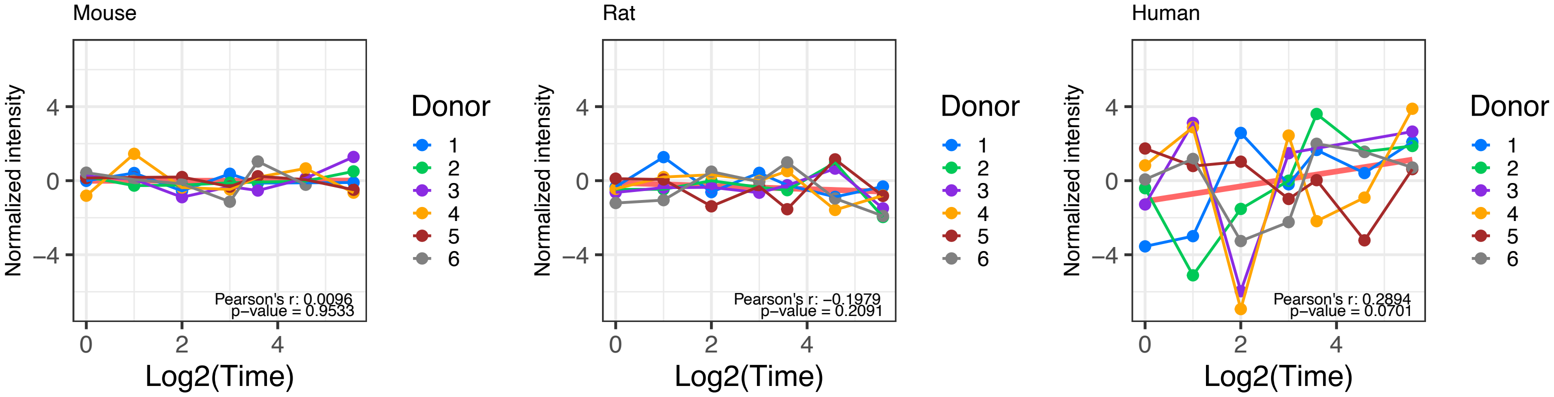

CL 74:10|CL 16:1_20:4_18:1_20:4

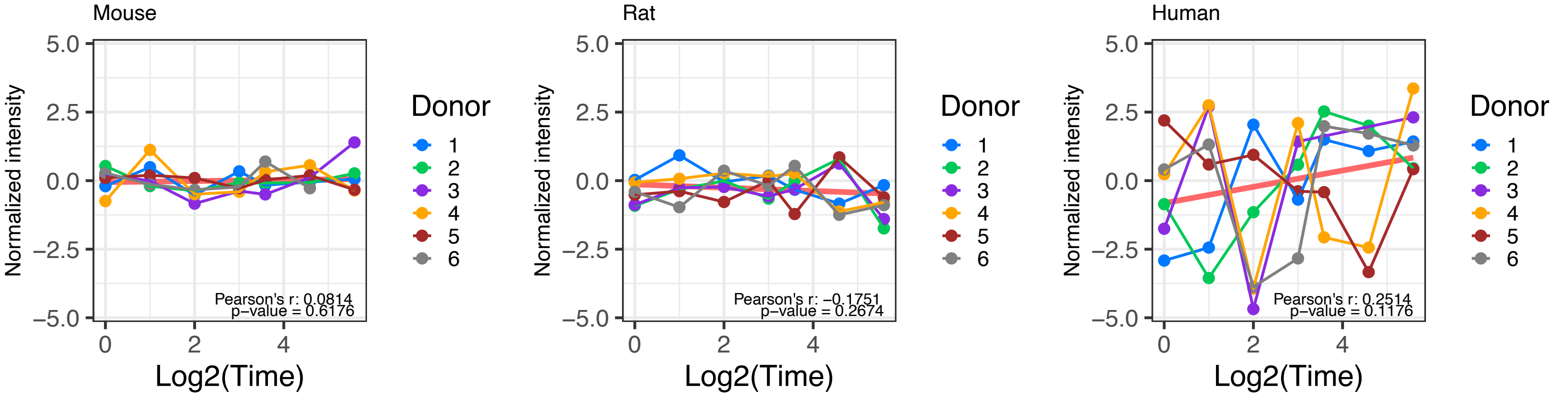

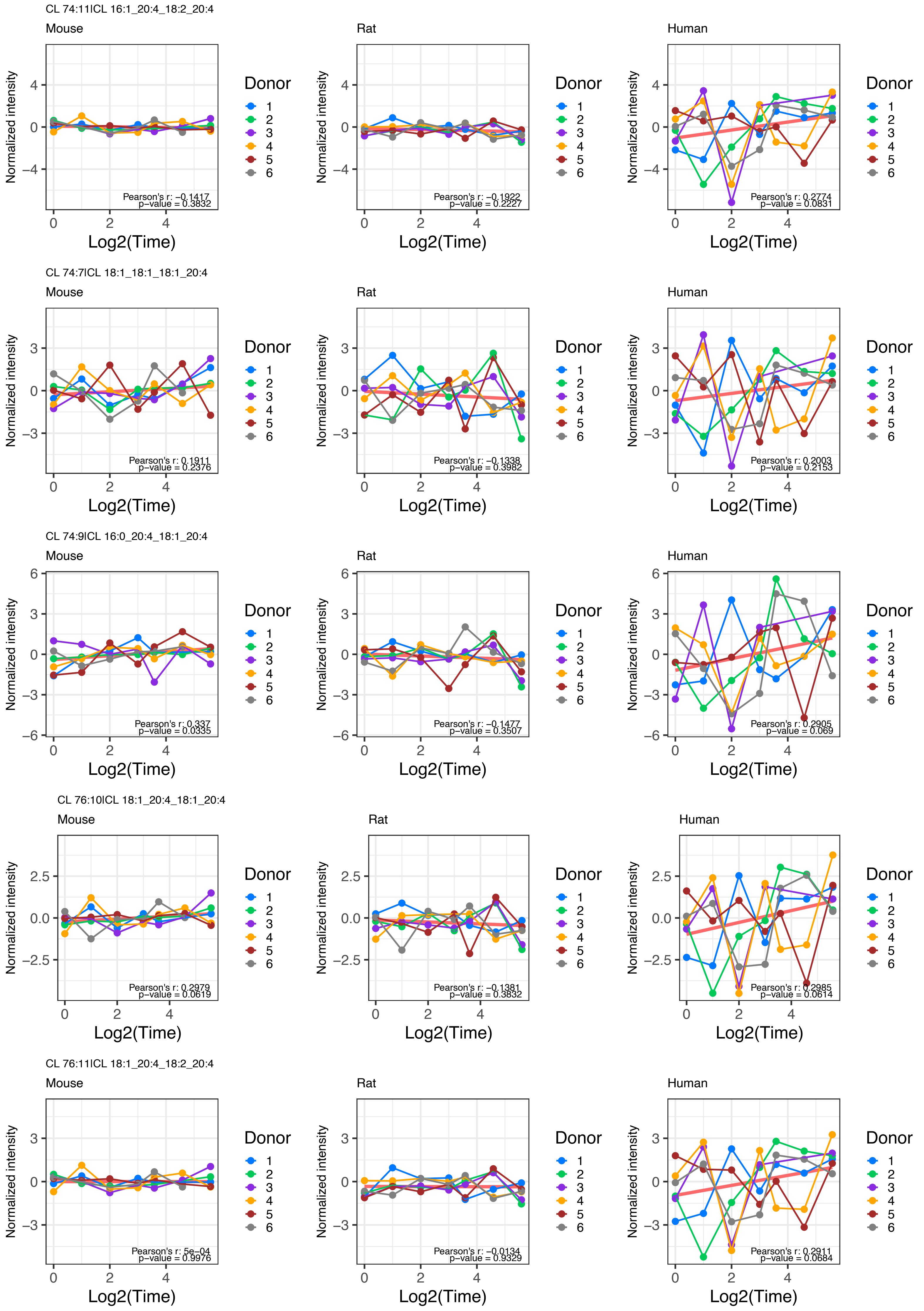

CL 74:11|CL 16:1_20:4_18:2_20:4
Mouse
Pearson's r: −0.1417
p−value = 0.3832
Rat
Pearson's r: −0.1922
p−value = 0.2227
Human
Pearson's r: 0.2774
p−value = 0.0831
CL 74:7|CL 18:1_18:1_18:1_20:4
Mouse
Pearson's r: 0.1911
p−value = 0.2376
Rat
Pearson's r: −0.1338
p−value = 0.3982
Human
Pearson's r: 0.2003
p−value = 0.2153
CL 74:9|CL 16:0_20:4_18:1_20:4
Mouse
Pearson's r: 0.337
p−value = 0.0335
Rat
Pearson's r: −0.1477
p−value = 0.3507
Human
Pearson's r: 0.2905
p−value = 0.069
CL 76:10|CL 18:1_20:4_18:1_20:4
Mouse
Pearson's r: 0.2979
p−value = 0.0619
Rat
Pearson's r: −0.1381
p−value = 0.3832
Human
Pearson's r: 0.2985
p−value = 0.0614
CL 76:11|CL 18:1_20:4_18:2_20:4
Mouse
Pearson's r: 5e−04
p−value = 0.9976
Rat
Pearson's r: −0.0134
p−value = 0.9329
Human
Pearson's r: 0.2911
p−value = 0.0684
Normalized intensity
Log2(Time)
Donor
1
2
3
4
5
6

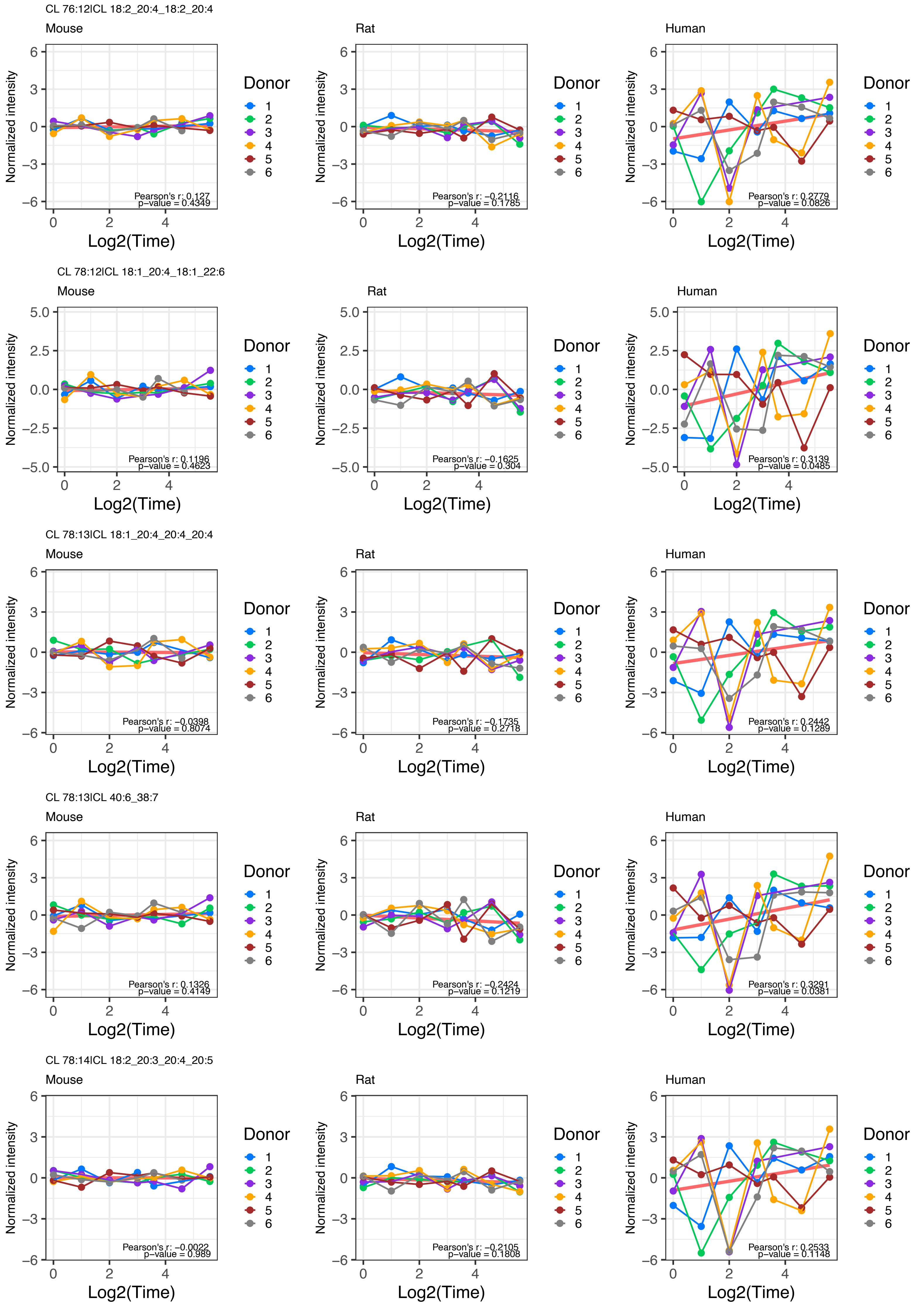
CL 76:12|CL 18:2_20:4_18:2_20:4
Mouse
Pearson's r: 0.127
p−value = 0.4349
Rat
Pearson's r: −0.2116
p−value = 0.1785
Human
Pearson's r: 0.2779
p−value = 0.0826
CL 78:12|CL 18:1_20:4_18:1_22:6
Mouse
Pearson's r: 0.1196
p−value = 0.4623
Rat
Pearson's r: −0.1625
p−value = 0.304
Human
Pearson's r: 0.3139
p−value = 0.0485
CL 78:13|CL 18:1_20:4_20:4_20:4
Mouse
Pearson's r: −0.0398
p−value = 0.8074
Rat
Pearson's r: −0.1735
p−value = 0.2718
Human
Pearson's r: 0.2442
p−value = 0.1289
CL 78:13|CL 40:6_38:7
Mouse
Pearson's r: 0.1326
p−value = 0.4149
Rat
Pearson's r: −0.2424
p−value = 0.1219
Human
Pearson's r: 0.3291
p−value = 0.0381
CL 78:14|CL 18:2_20:3_20:4_20:5
Mouse
Pearson's r: −0.0022
p−value = 0.989
Rat
Pearson's r: −0.2105
p−value = 0.1808
Human
Pearson's r: 0.2533
p−value = 0.1148
Normalized intensity
Log2(Time)
Donor
1
2
3
4
5
6

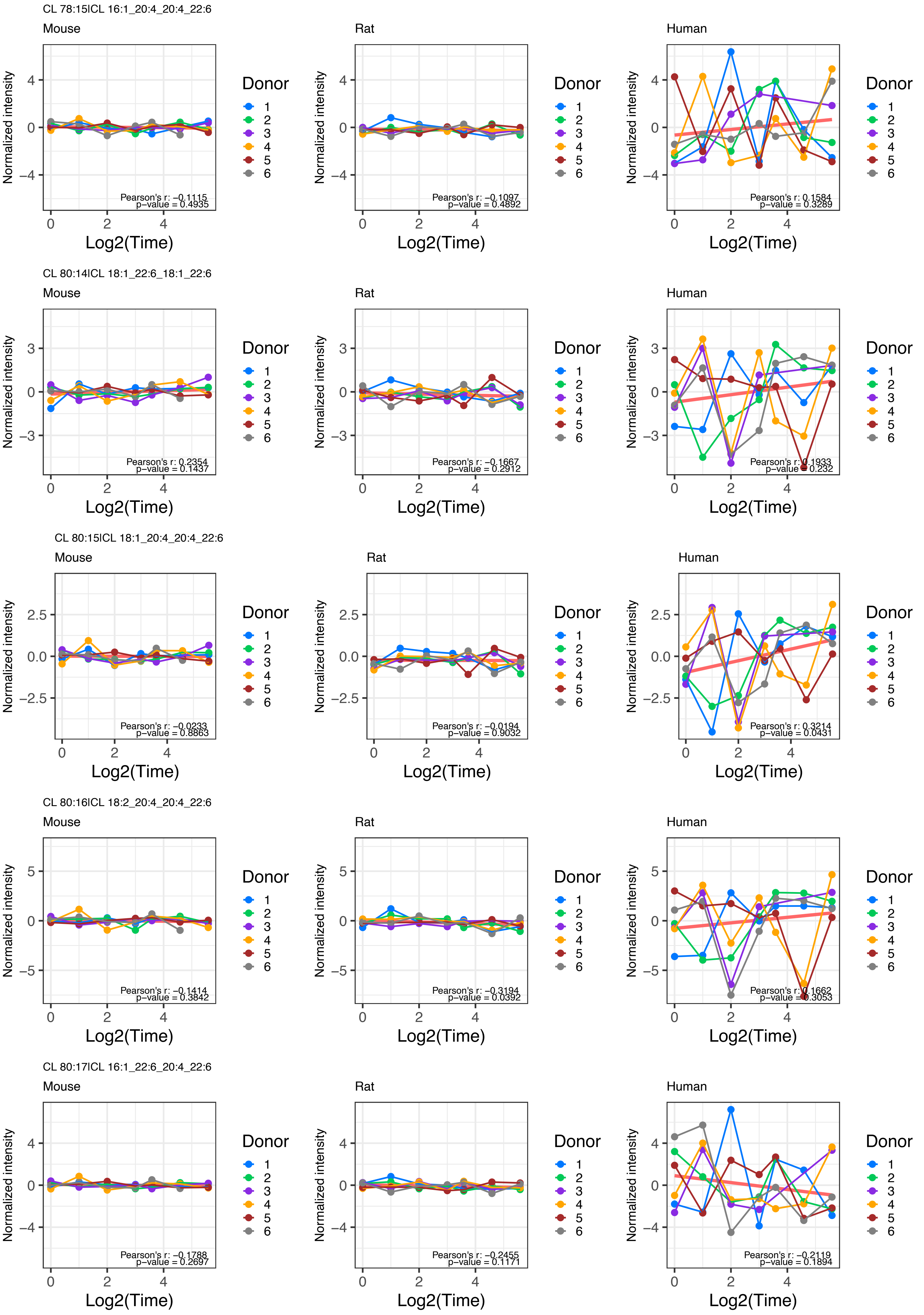
CL 78:15|CL 16:1_20:4_20:4_22:6
Mouse
Rat
Human
Normalized intensity
Log2(Time)
Donor
1
2
3
4
5
6
Pearson's r: −0.1115
p−value = 0.4935
Pearson's r: −0.1097
p−value = 0.4892
Pearson's r: 0.1584
p−value = 0.3289
CL 80:14|CL 18:1_22:6_18:1_22:6
Pearson's r: 0.2354
p−value = 0.1437
Pearson's r: −0.1667
p−value = 0.2912
Pearson's r: 0.1933
p−value = 0.232
CL 80:15|CL 18:1_20:4_20:4_22:6
Pearson's r: −0.0233
p−value = 0.8863
Pearson's r: −0.0194
p−value = 0.9032
Pearson's r: 0.3214
p−value = 0.0431
CL 80:16|CL 18:2_20:4_20:4_22:6
Pearson's r: −0.1414
p−value = 0.3842
Pearson's r: −0.3194
p−value = 0.0392
Pearson's r: 0.1662
p−value = 0.3053
CL 80:17|CL 16:1_22:6_20:4_22:6
Pearson's r: −0.1788
p−value = 0.2697
Pearson's r: −0.2455
p−value = 0.1171
Pearson's r: −0.2119
p−value = 0.1894

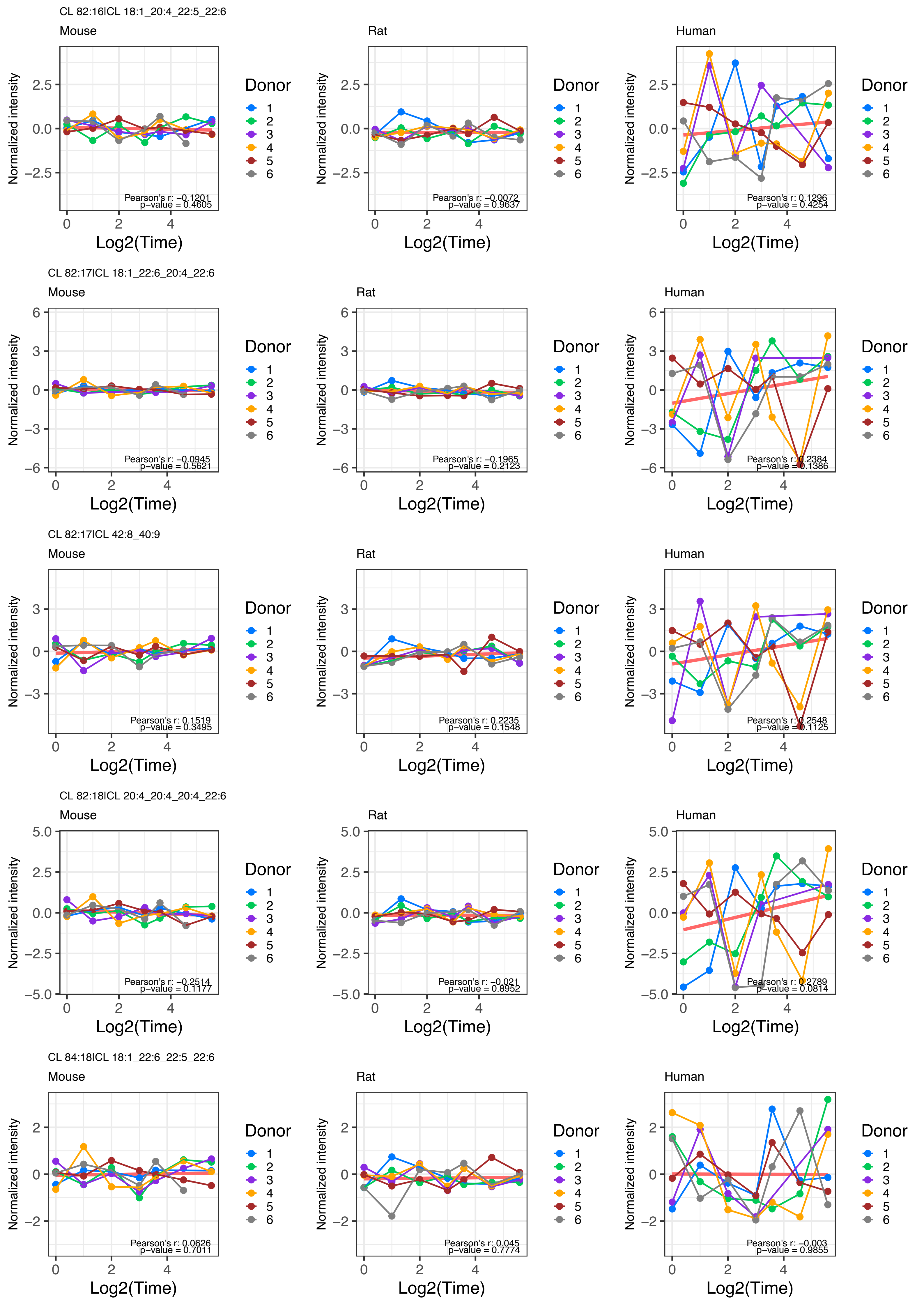

CL 82:16|CL 18:1_20:4_22:5_22:6
Mouse
Pearson's r: −0.1201
p−value = 0.4605
Rat
Pearson's r: −0.0072
p−value = 0.9637
Human
Pearson's r: 0.1296
p−value = 0.4254
CL 82:17|CL 18:1_22:6_20:4_22:6
Mouse
Pearson's r: −0.0945
p−value = 0.5621
Rat
Pearson's r: −0.1965
p−value = 0.2123
Human
Pearson's r: 0.2384
p−value = 0.1386
CL 82:17|CL 42:8_40:9
Mouse
Pearson's r: 0.1519
p−value = 0.3495
Rat
Pearson's r: 0.2235
p−value = 0.1548
Human
Pearson's r: 0.2548
p−value = 0.1125
CL 82:18|CL 20:4_20:4_20:4_22:6
Mouse
Pearson's r: −0.2514
p−value = 0.1177
Rat
Pearson's r: −0.021
p−value = 0.8952
Human
Pearson's r: 0.2789
p−value = 0.0814
CL 84:18|CL 18:1_22:6_22:5_22:6
Mouse
Pearson's r: 0.0626
p−value = 0.7011
Rat
Pearson's r: 0.045
p−value = 0.7774
Human
Pearson's r: −0.003
p−value = 0.9855
Normalized intensity
Log2(Time)
Donor
1
2
3
4
5
6

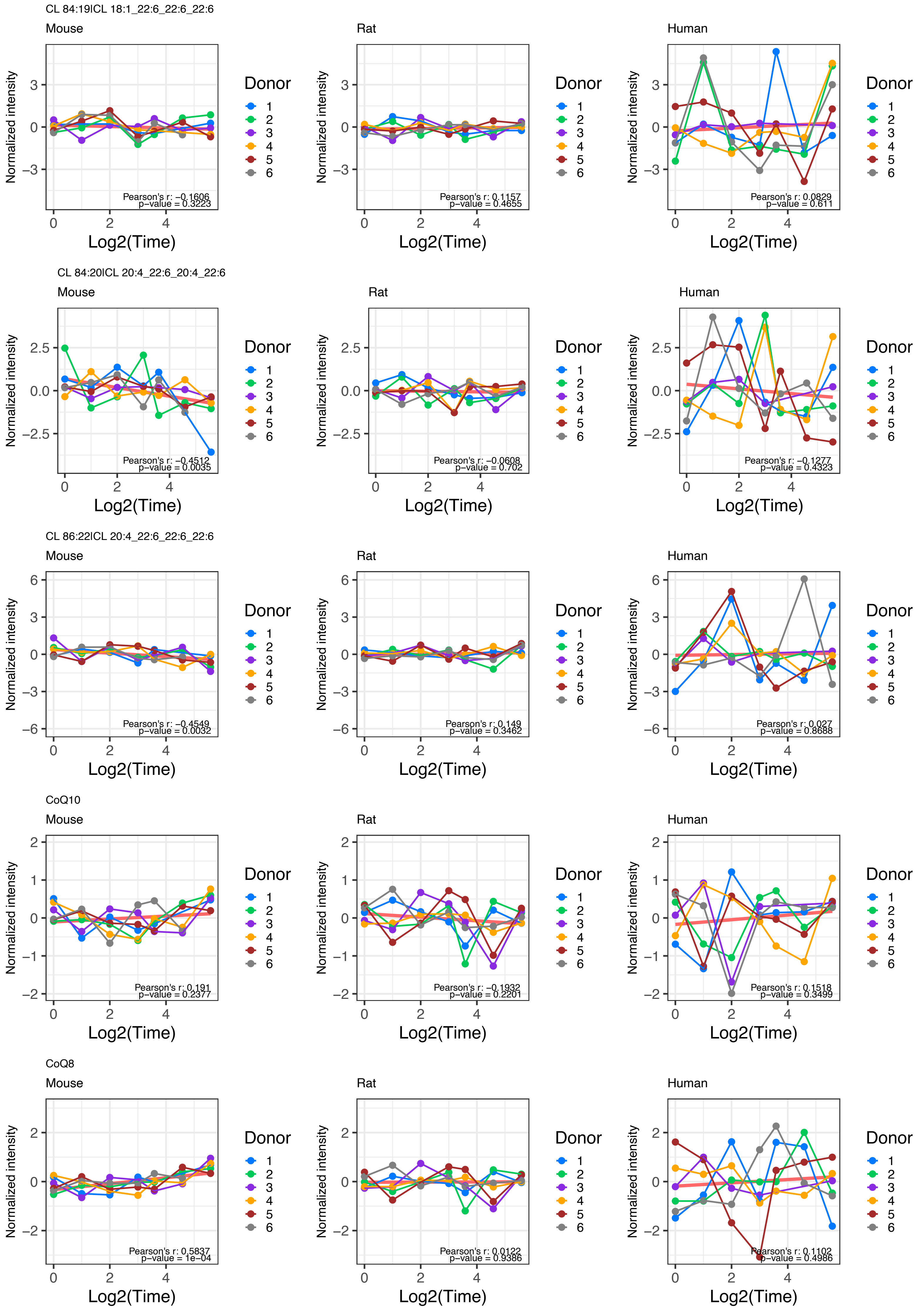
CL 84:19|CL 18:1_22:6_22:6_22:6
Mouse
Rat
Human
Normalized intensity
Log2(Time)
Donor
1
2
3
4
5
6
Pearson's r: −0.1606
p−value = 0.3223
Pearson's r: 0.1157
p−value = 0.4655
Pearson's r: 0.0829
p−value = 0.611
CL 84:20|CL 20:4_22:6_20:4_22:6
Mouse
Rat
Human
Pearson's r: −0.4512
p−value = 0.0035
Pearson's r: −0.0608
p−value = 0.702
Pearson's r: −0.1277
p−value = 0.4323
CL 86:22|CL 20:4_22:6_22:6_22:6
Mouse
Rat
Human
Pearson's r: −0.4549
p−value = 0.0032
Pearson's r: 0.149
p−value = 0.3462
Pearson's r: 0.027
p−value = 0.8688
CoQ10
Mouse
Rat
Human
Pearson's r: 0.191
p−value = 0.2377
Pearson's r: −0.1932
p−value = 0.2201
Pearson's r: 0.1518
p−value = 0.3499
CoQ8
Mouse
Rat
Human
Pearson's r: 0.5837
p−value = 1e−04
Pearson's r: 0.0122
p−value = 0.9386
Pearson's r: 0.1102
p−value = 0.4986

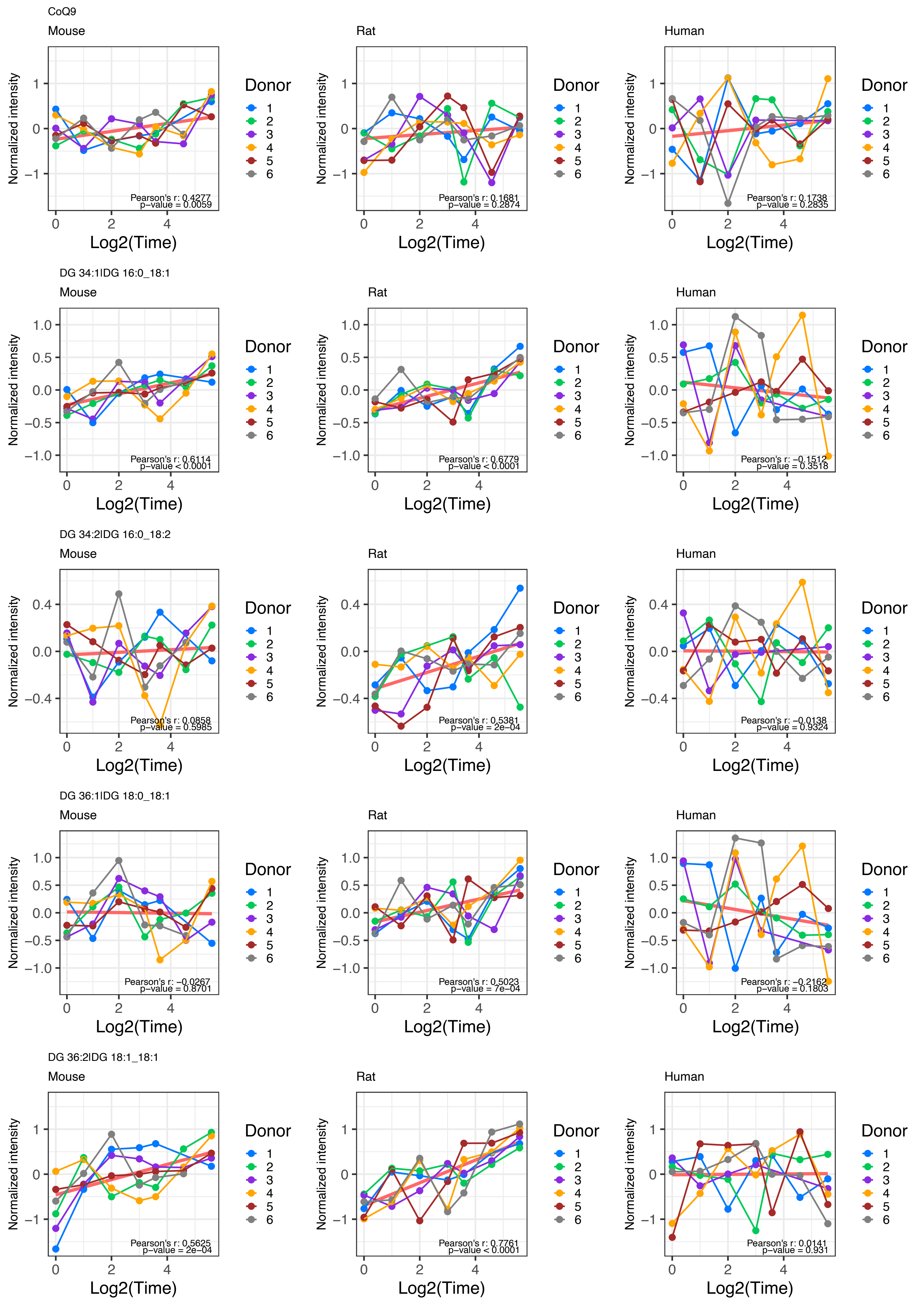
CoQ9
Mouse
Rat
Human
Pearson's r: 0.4277
p-value = 0.0059
Pearson's r: 0.1681
p-value = 0.2874
Pearson's r: 0.1738
p-value = 0.2835
DG 34:1|DG 16:0_18:1
Mouse
Rat
Human
Pearson's r: 0.6114
p-value < 0.0001
Pearson's r: 0.6779
p-value < 0.0001
Pearson's r: −0.1512
p-value = 0.3518
DG 34:2|DG 16:0_18:2
Mouse
Rat
Human
Pearson's r: 0.0858
p-value = 0.5985
Pearson's r: 0.5381
p-value = 2e−04
Pearson's r: −0.0138
p-value = 0.9324
DG 36:1|DG 18:0_18:1
Mouse
Rat
Human
Pearson's r: −0.0267
p-value = 0.8701
Pearson's r: 0.5023
p-value = 7e−04
Pearson's r: −0.2162
p-value = 0.1803
DG 36:2|DG 18:1_18:1
Mouse
Rat
Human
Pearson's r: 0.5625
p-value = 2e−04
Pearson's r: 0.7761
p-value < 0.0001
Pearson's r: 0.0141
p-value = 0.931
Normalized intensity
Log2(Time)
Donor
1
2
3
4
5
6

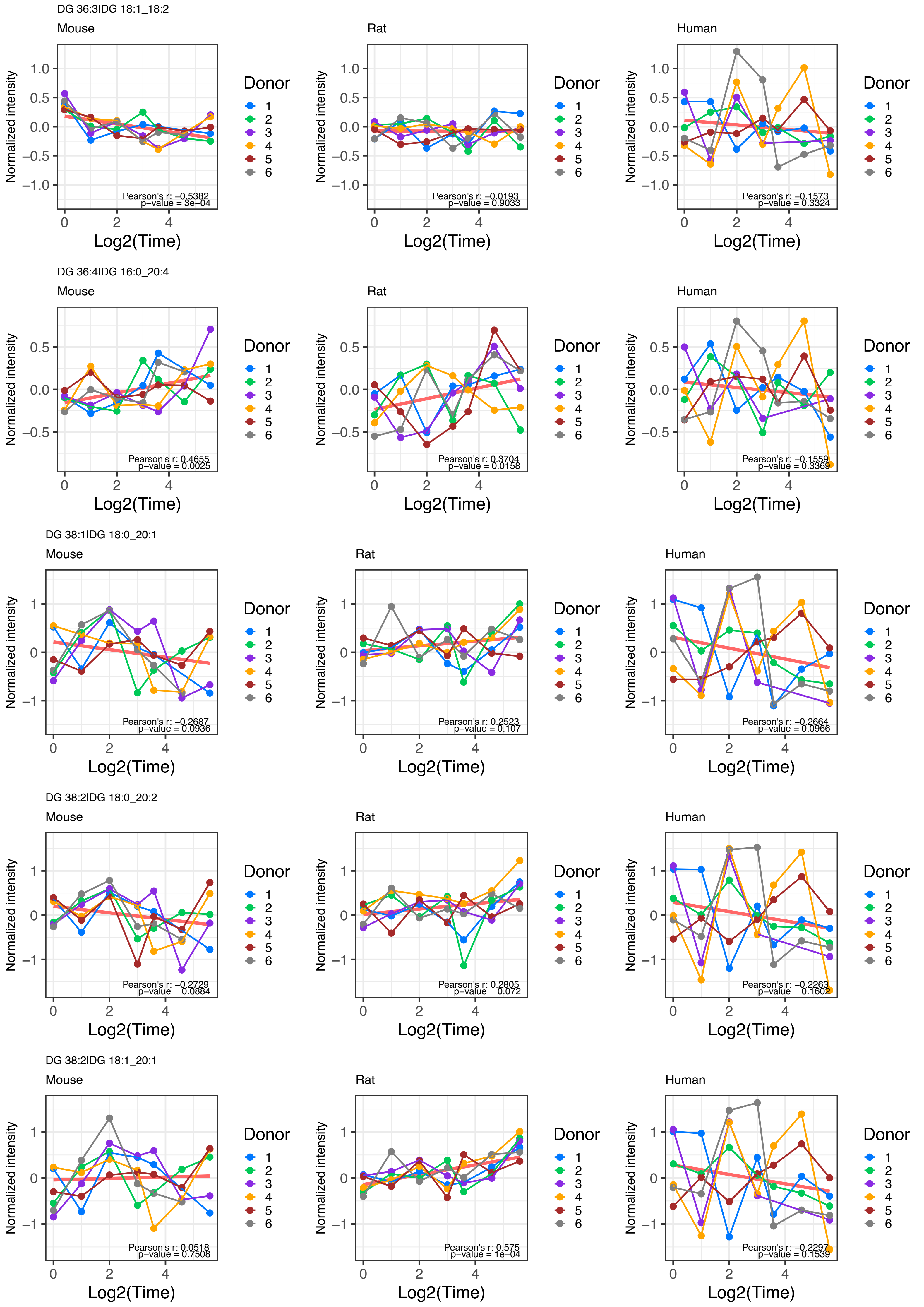
DG 36:3|DG 18:1_18:2
Mouse
Pearson's r: −0.5382
p−value = 3e−04
Rat
Pearson's r: −0.0193
p−value = 0.9033
Human
Pearson's r: −0.1573
p−value = 0.3324
DG 36:4|DG 16:0_20:4
Mouse
Pearson's r: 0.4655
p−value = 0.0025
Rat
Pearson's r: 0.3704
p−value = 0.0158
Human
Pearson's r: −0.1559
p−value = 0.3369
DG 38:1|DG 18:0_20:1
Mouse
Pearson's r: −0.2687
p−value = 0.0936
Rat
Pearson's r: 0.2523
p−value = 0.107
Human
Pearson's r: −0.2664
p−value = 0.0966
DG 38:2|DG 18:0_20:2
Mouse
Pearson's r: −0.2729
p−value = 0.0884
Rat
Pearson's r: 0.2805
p−value = 0.072
Human
Pearson's r: −0.2263
p−value = 0.1602
DG 38:2|DG 18:1_20:1
Mouse
Pearson's r: 0.0518
p−value = 0.7508
Rat
Pearson's r: 0.575
p−value = 1e−04
Human
Pearson's r: −0.2297
p−value = 0.1539
Normalized intensity
Log2(Time)
Donor
1
2
3
4
5
6

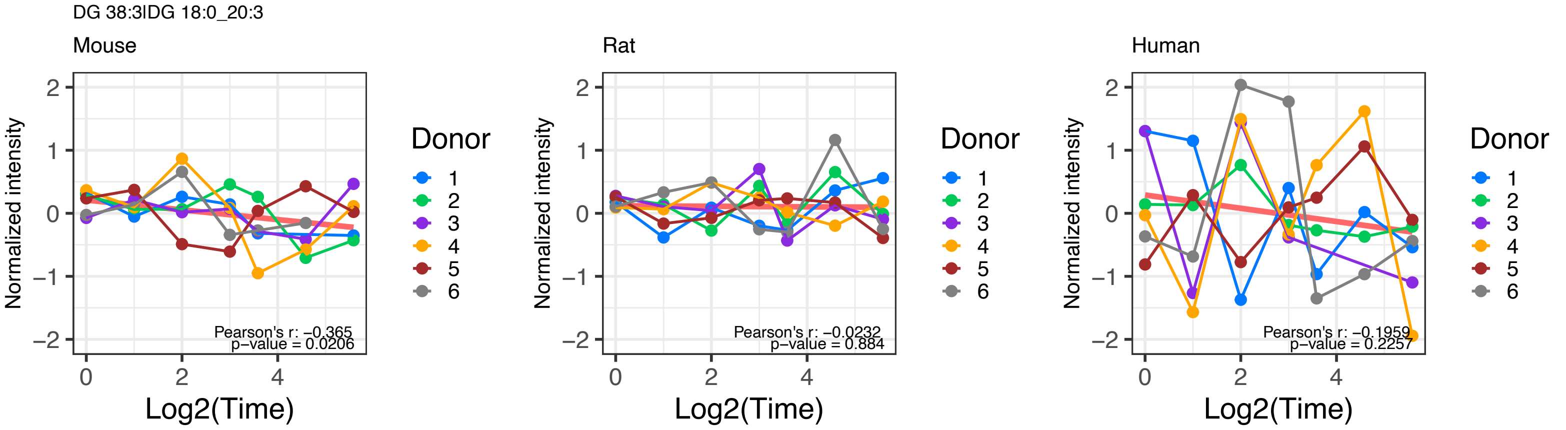
DG 38:3|DG 18:0_20:3
Mouse
Rat
Human
Normalized intensity
Log2(Time)
Donor
1
2
3
4
5
6
Pearson's r: −0.365
p−value = 0.0206
Pearson's r: −0.0232
p−value = 0.884
Pearson's r: −0.1959
p−value = 0.2257

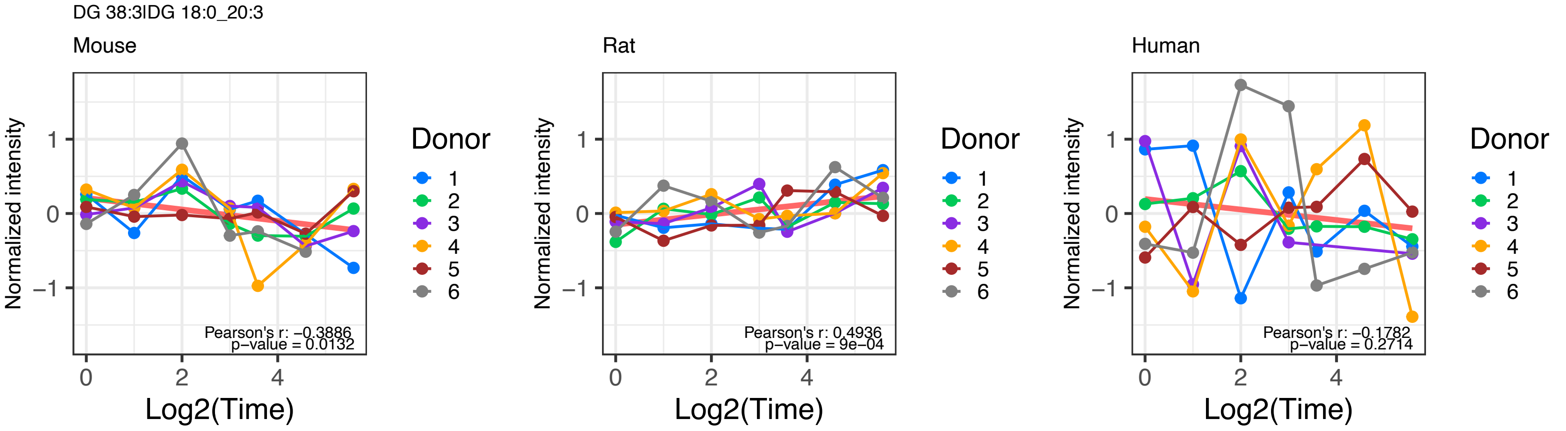
DG 38:3|DG 18:0_20:3
Mouse
Rat
Human
Normalized intensity
Log2(Time)
Donor
1
2
3
4
5
6
Pearson's r: −0.3886
p−value = 0.0132
Pearson's r: 0.4936
p−value = 9e−04
Pearson's r: −0.1782
p−value = 0.2714

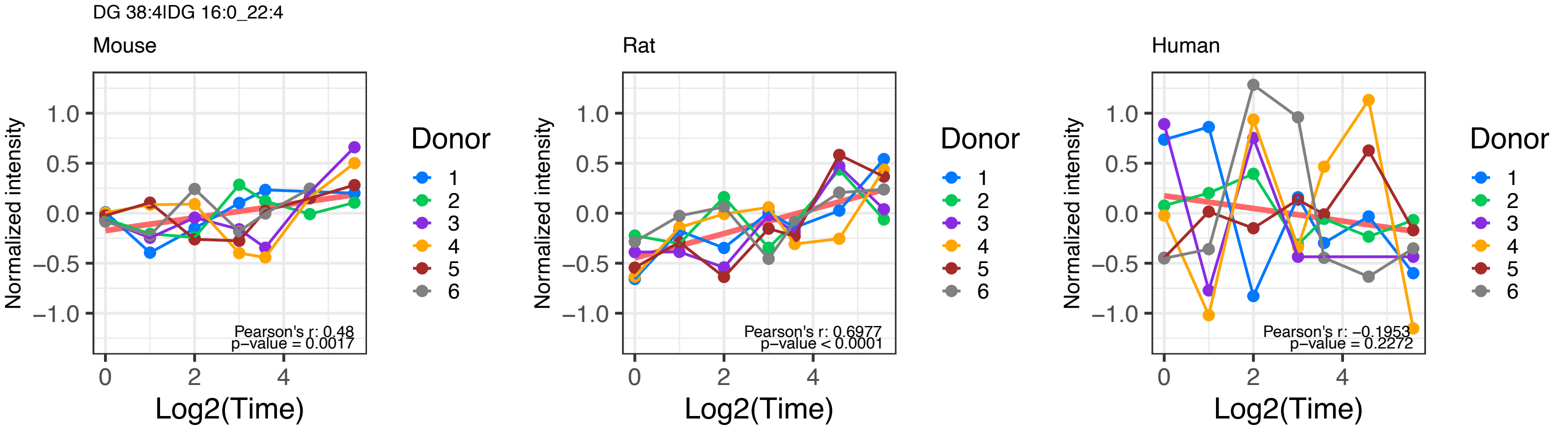
DG 38:4|DG 16:0_22:4
Mouse
Rat
Human
Normalized intensity
Log2(Time)
Donor
1
2
3
4
5
6
Pearson's r: 0.48
p−value = 0.0017
Pearson's r: 0.6977
p−value < 0.0001
Pearson's r: −0.1953
p−value = 0.2272

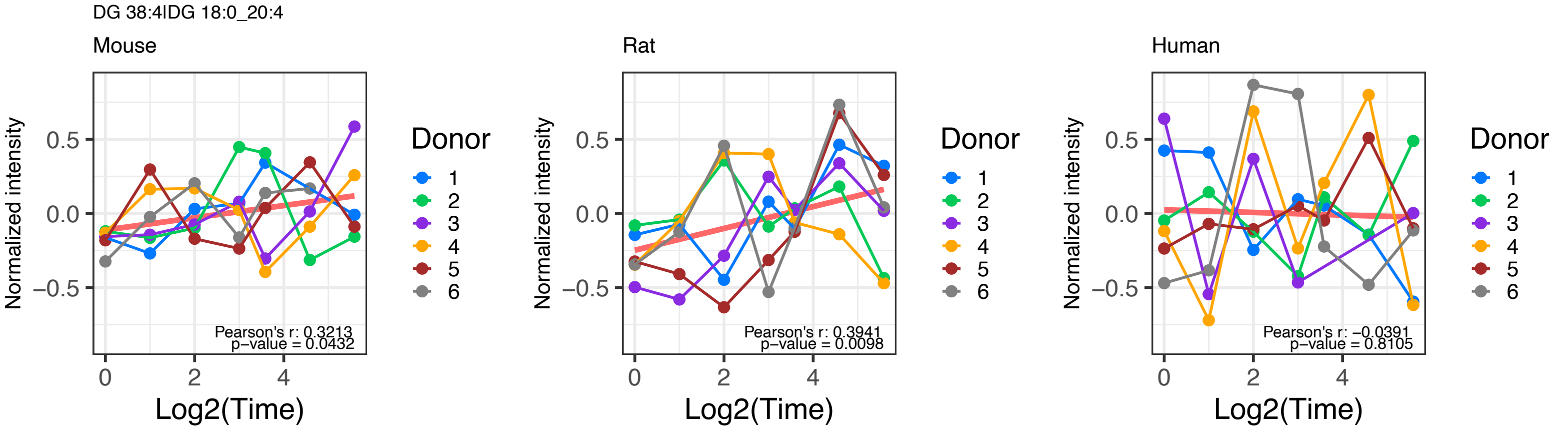
DG 38:4|DG 18:0_20:4
Mouse
Rat
Human
Normalized intensity
Log2(Time)
Donor
1
2
3
4
5
6
Pearson's r: 0.3213
p−value = 0.0432
Pearson's r: 0.3941
p−value = 0.0098
Pearson's r: −0.0391
p−value = 0.8105

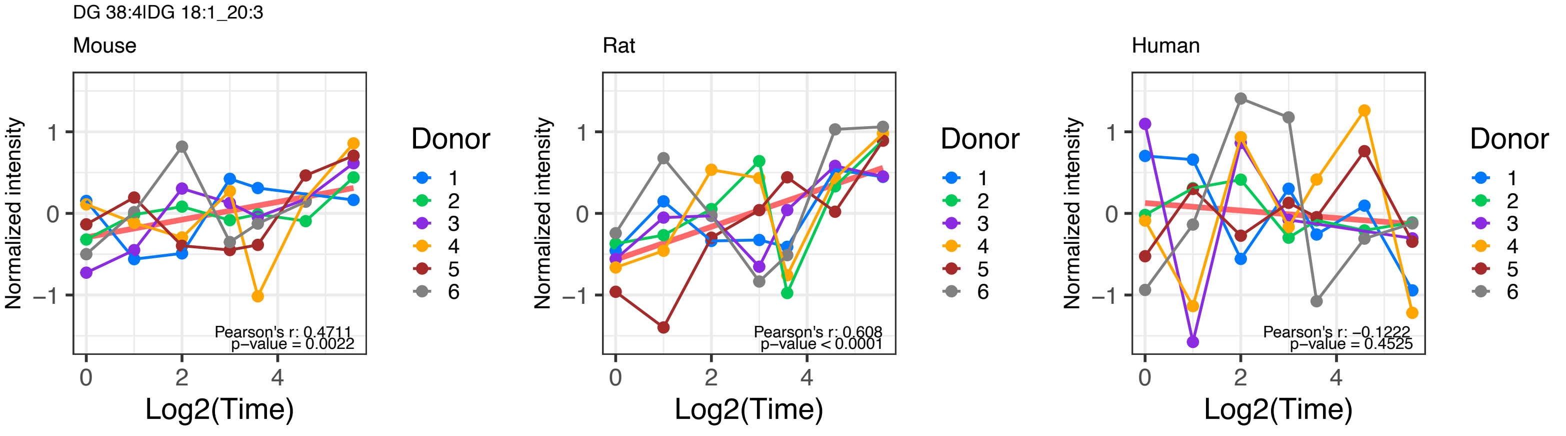
DG 38:4|DG 18:1_20:3
Mouse
Rat
Human
Normalized intensity
Log2(Time)
Donor
1
2
3
4
5
6
Pearson's r: 0.4711
p−value = 0.0022
Pearson's r: 0.608
p−value < 0.0001
Pearson's r: −0.1222
p−value = 0.4525

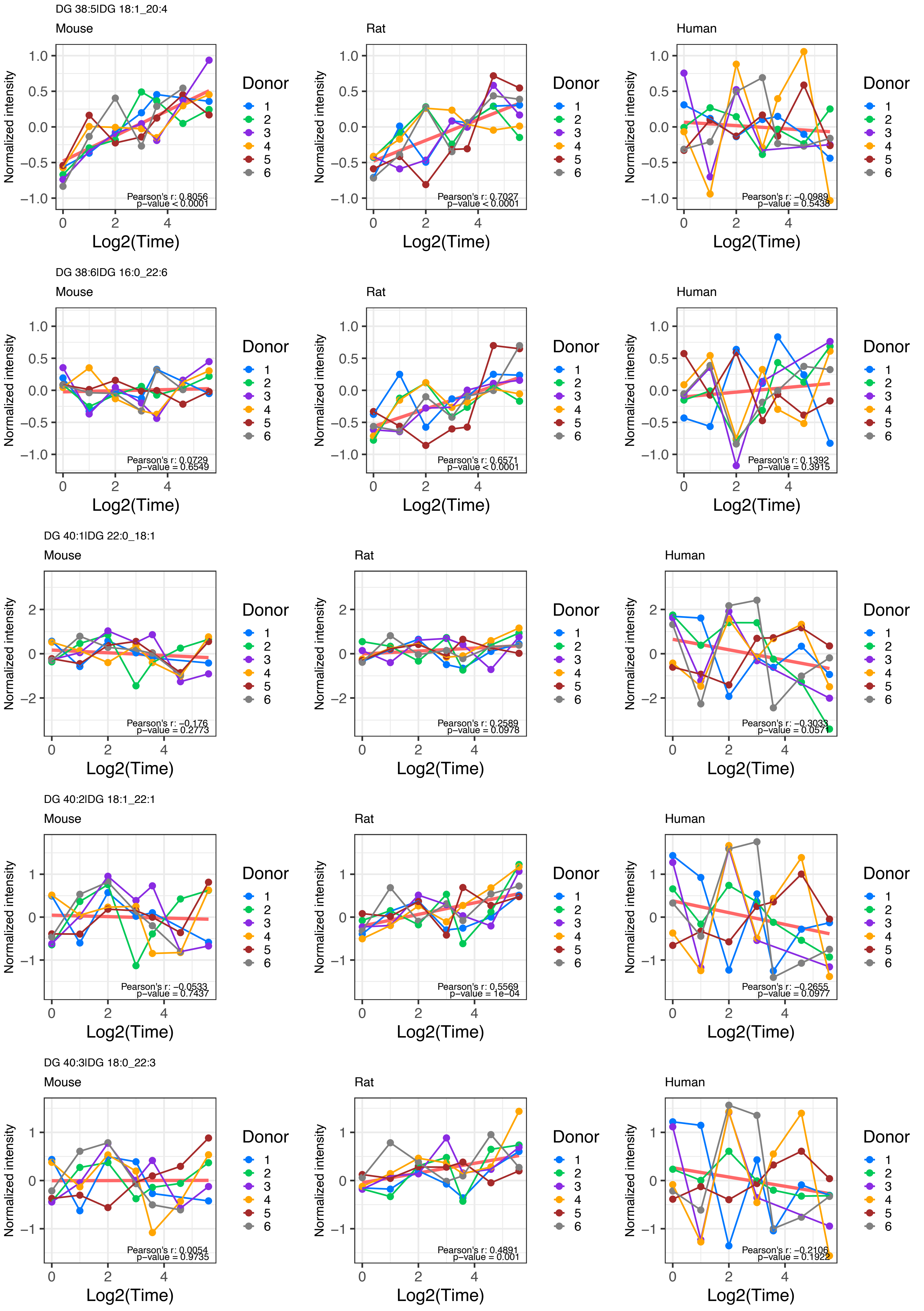

DG 38:5|DG 18:1_20:4
Mouse
Pearson's r: 0.8056
p−value < 0.0001
Rat
Pearson's r: 0.7027
p−value < 0.0001
Human
Pearson's r: −0.0989
p−value = 0.5438
DG 38:6|DG 16:0_22:6
Mouse
Pearson's r: 0.0729
p−value = 0.6549
Rat
Pearson's r: 0.6571
p−value < 0.0001
Human
Pearson's r: 0.1392
p−value = 0.3915
DG 40:1|DG 22:0_18:1
Mouse
Pearson's r: −0.176
p−value = 0.2773
Rat
Pearson's r: 0.2589
p−value = 0.0978
Human
Pearson's r: −0.3033
p−value = 0.0571
DG 40:2|DG 18:1_22:1
Mouse
Pearson's r: −0.0533
p−value = 0.7437
Rat
Pearson's r: 0.5569
p−value = 1e−04
Human
Pearson's r: −0.2655
p−value = 0.0977
DG 40:3|DG 18:0_22:3
Mouse
Pearson's r: 0.0054
p−value = 0.9735
Rat
Pearson's r: 0.4891
p−value = 0.001
Human
Pearson's r: −0.2106
p−value = 0.1922
Normalized intensity
Log2(Time)
Donor
1
2
3
4
5
6

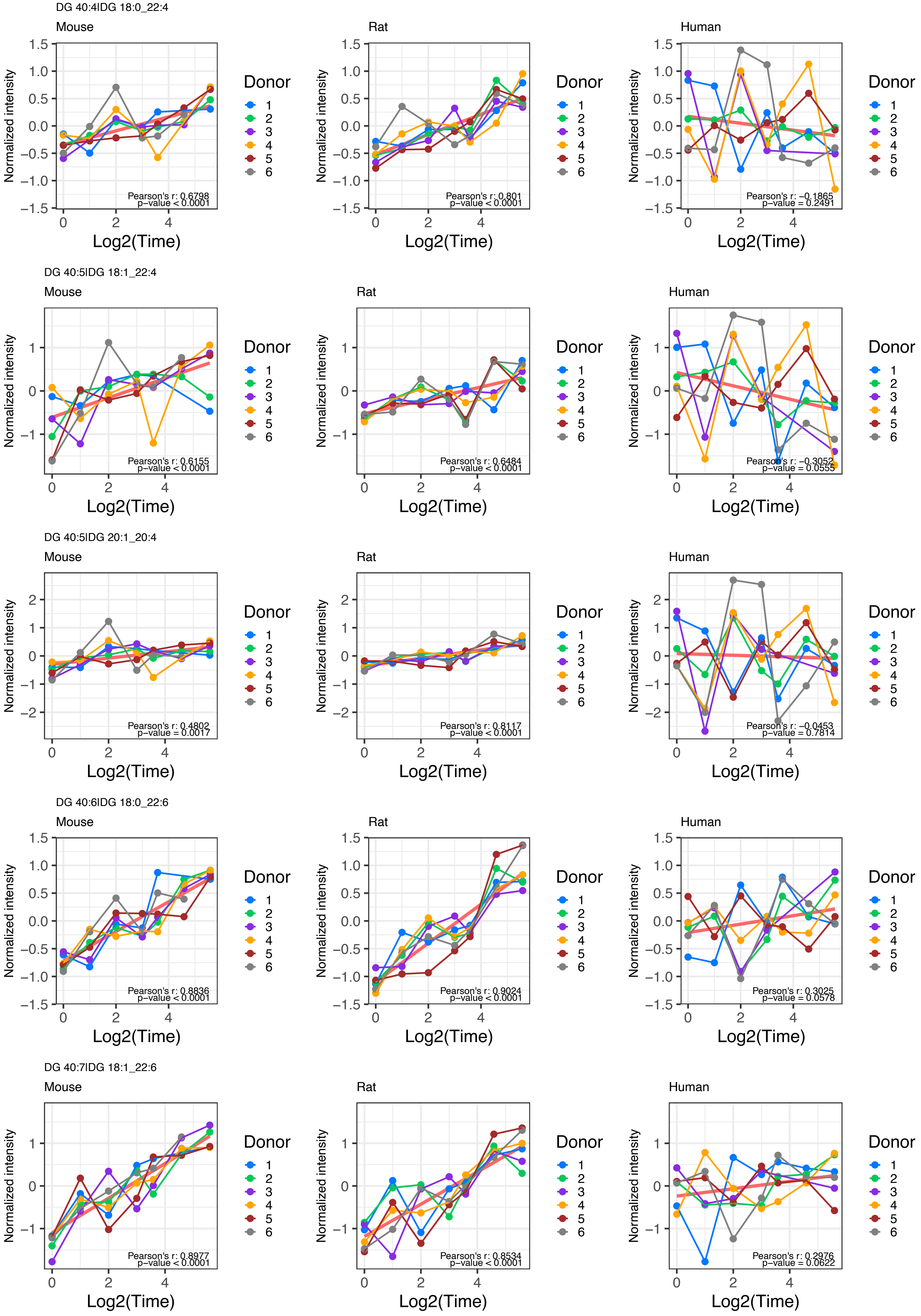
DG 40:4|DG 18:0_22:4
Mouse
Pearson's r: 0.6798
p−value < 0.0001
Rat
Pearson's r: 0.801
p−value < 0.0001
Human
Pearson's r: −0.1865
p−value = 0.2491
DG 40:5|DG 18:1_22:4
Mouse
Pearson's r: 0.6155
p−value < 0.0001
Rat
Pearson's r: 0.6484
p−value < 0.0001
Human
Pearson's r: −0.3052
p−value = 0.0555
DG 40:5|DG 20:1_20:4
Mouse
Pearson's r: 0.4802
p−value = 0.0017
Rat
Pearson's r: 0.8117
p−value < 0.0001
Human
Pearson's r: −0.0453
p−value = 0.7814
DG 40:6|DG 18:0_22:6
Mouse
Pearson's r: 0.8836
p−value < 0.0001
Rat
Pearson's r: 0.9024
p−value < 0.0001
Human
Pearson's r: 0.3025
p−value = 0.0578
DG 40:7|DG 18:1_22:6
Mouse
Pearson's r: 0.8977
p−value < 0.0001
Rat
Pearson's r: 0.8534
p−value < 0.0001
Human
Pearson's r: 0.2976
p−value = 0.0622
Normalized intensity
Log2(Time)
Donor
1
2
3
4
5
6

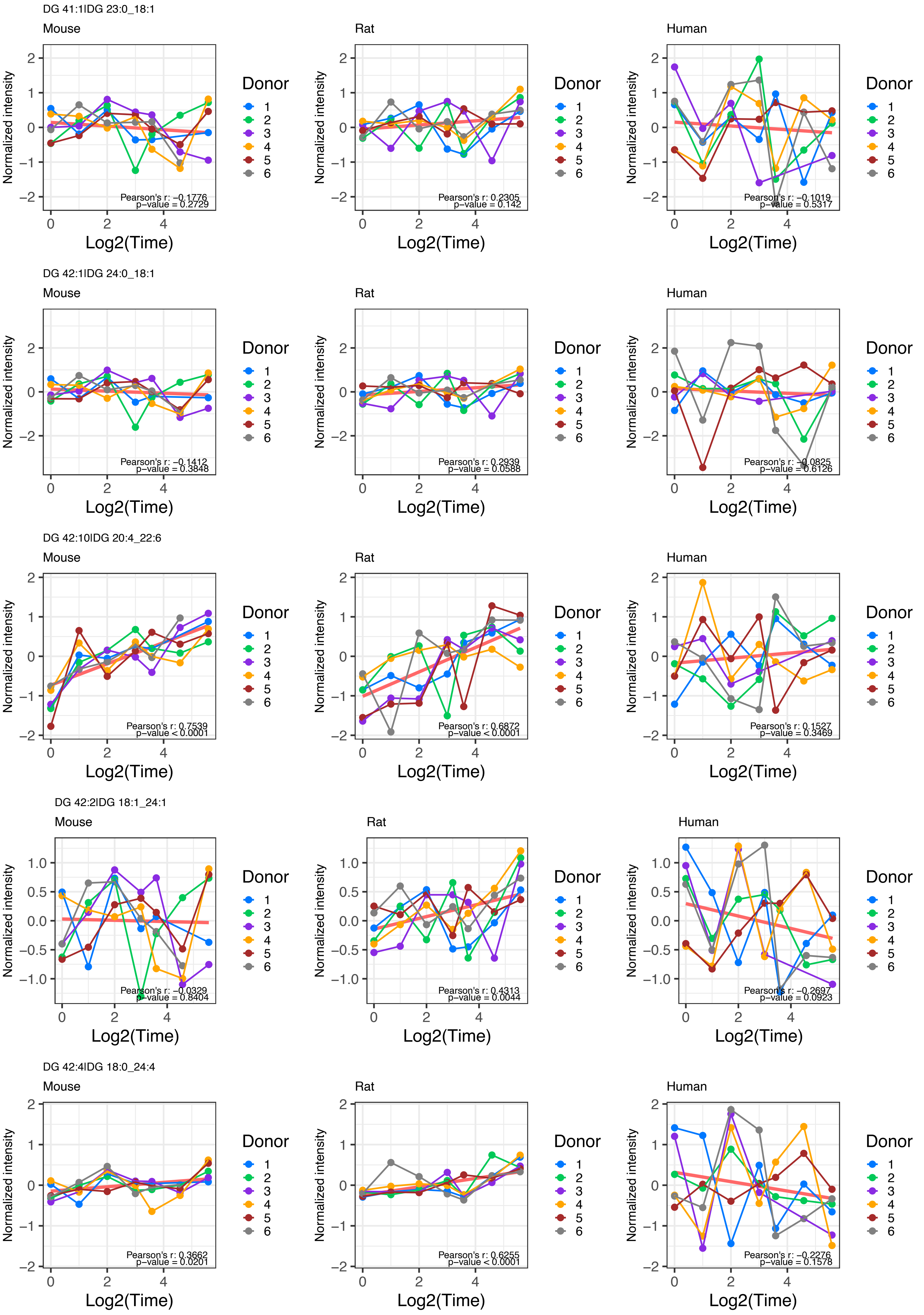

DG 41:1|DG 23:0_18:1
Mouse
Rat
Human
Normalized intensity
Log2(Time)
Donor
1
2
3
4
5
6
Pearson's r: −0.1776
p−value = 0.2729
Pearson's r: 0.2305
p−value = 0.142
Pearson's r: −0.1019
p−value = 0.5317
DG 42:1|DG 24:0_18:1
Pearson's r: −0.1412
p−value = 0.3848
Pearson's r: 0.2939
p−value = 0.0588
Pearson's r: −0.0825
p−value = 0.6126
DG 42:10|DG 20:4_22:6
Pearson's r: 0.7539
p−value < 0.0001
Pearson's r: 0.6872
p−value < 0.0001
Pearson's r: 0.1527
p−value = 0.3469
DG 42:2|DG 18:1_24:1
Pearson's r: −0.0329
p−value = 0.8404
Pearson's r: 0.4313
p−value = 0.0044
Pearson's r: −0.2697
p−value = 0.0923
DG 42:4|DG 18:0_24:4
Pearson's r: 0.3662
p−value = 0.0201
Pearson's r: 0.6255
p−value < 0.0001
Pearson's r: −0.2276
p−value = 0.1578

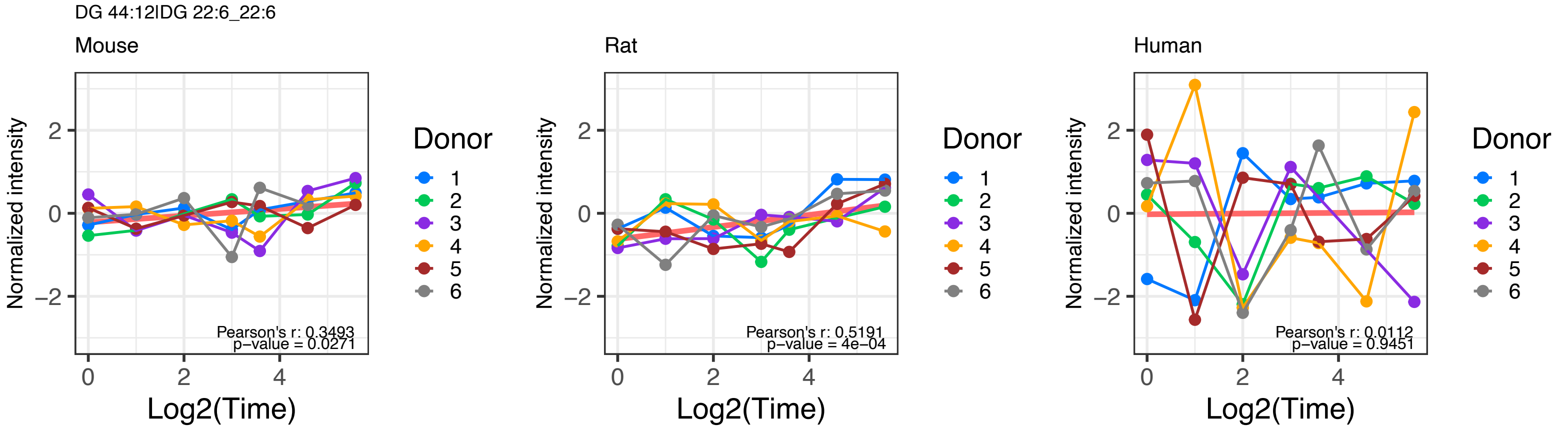
DG 44:12|DG 22:6_22:6
Mouse
Rat
Human
Normalized intensity
Log2(Time)
Donor
1
2
3
4
5
6
Pearson's r: 0.3493
p−value = 0.0271
Pearson's r: 0.5191
p−value = 4e−04
Pearson's r: 0.0112
p−value = 0.9451

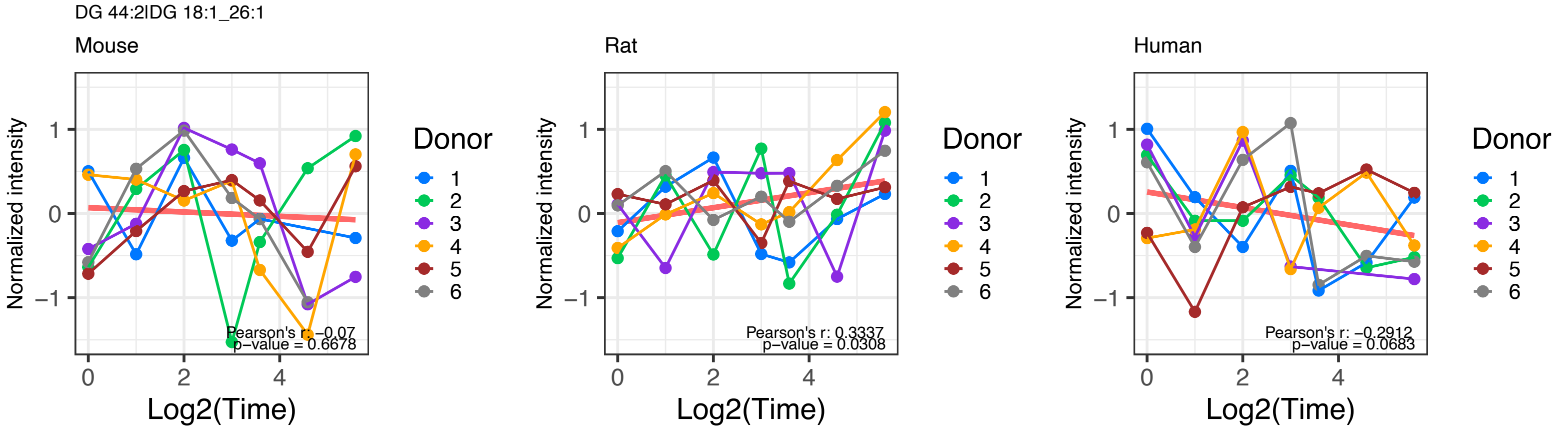
DG 44:2|DG 18:1_26:1
Mouse
Rat
Human
Normalized intensity
Log2(Time)
Donor
1
2
3
4
5
6
Pearson's r: −0.07
p−value = 0.6678
Pearson's r: 0.3337
p−value = 0.0308
Pearson's r: −0.2912
p−value = 0.0683

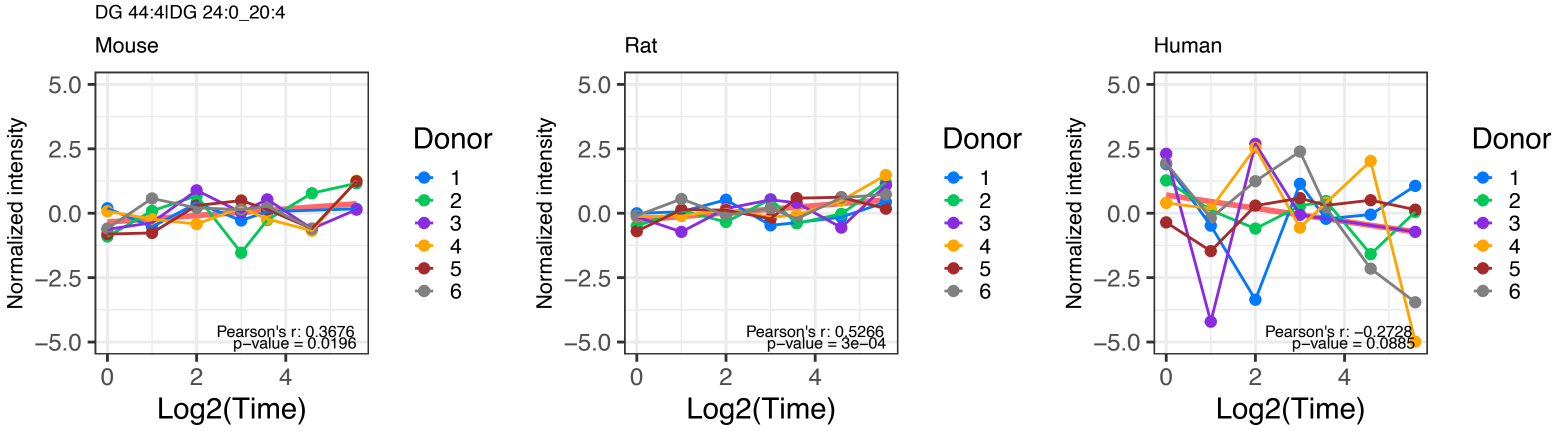
DG 44:4|DG 24:0_20:4
Mouse
Rat
Human
Normalized intensity
Log2(Time)
Donor
1
2
3
4
5
6
Pearson's r: 0.3676
p−value = 0.0196
Pearson's r: 0.5266
p−value = 3e−04
Pearson's r: −0.2728
p−value = 0.0885

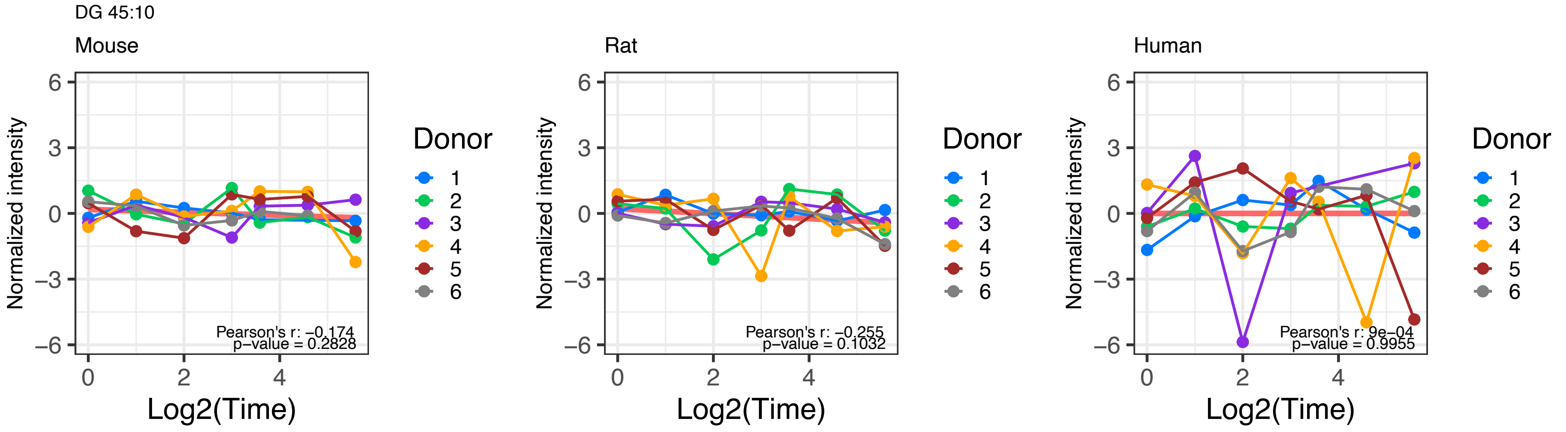
DG 45:10
Mouse
Rat
Human
Normalized intensity
Log2(Time)
Donor
1
2
3
4
5
6
Pearson's r: −0.174
p−value = 0.2828
Pearson's r: −0.255
p−value = 0.1032
Pearson's r: 9e−04
p−value = 0.9955

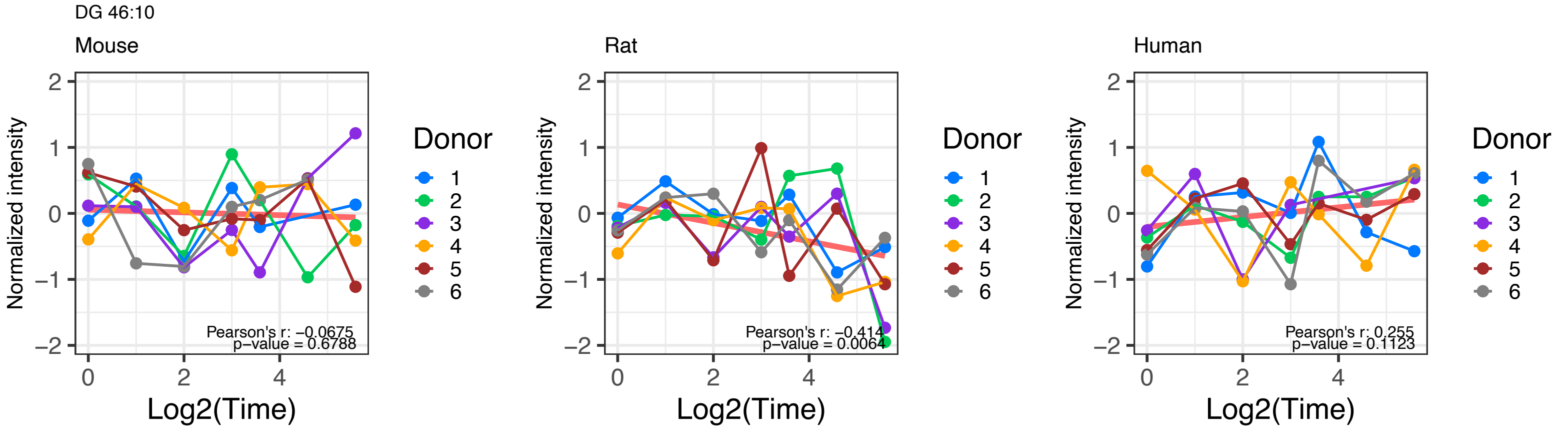
DG 46:10
Mouse
Rat
Human
Normalized intensity
Log2(Time)
Donor
1
2
3
4
5
6
Pearson's r: −0.0675
p−value = 0.6788
Pearson's r: −0.414
p−value = 0.0064
Pearson's r: 0.255
p−value = 0.1123

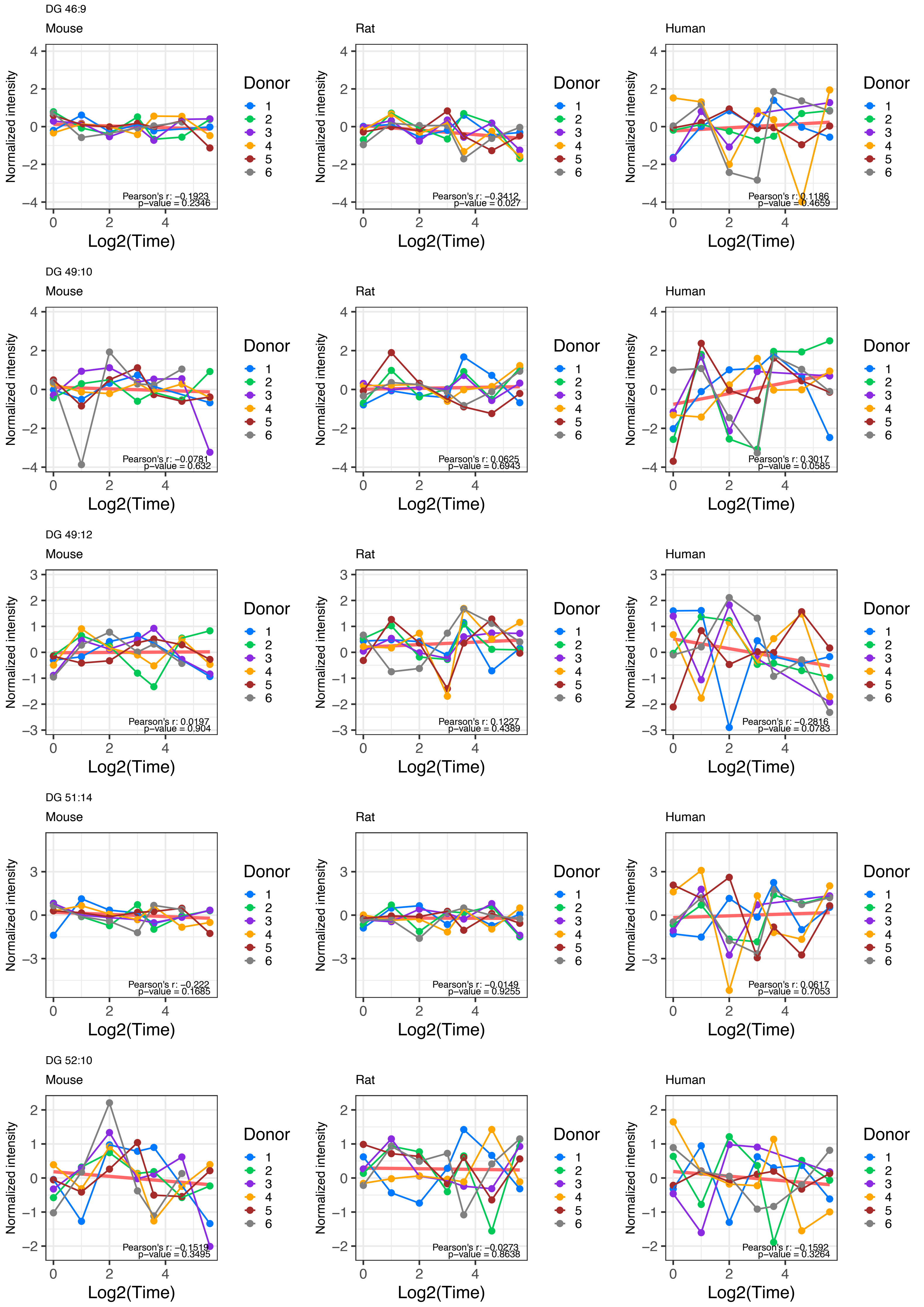
DG 46:9
Mouse
Pearson's r: −0.1923
p−value = 0.2346
Rat
Pearson's r: −0.3412
p−value = 0.027
Human
Pearson's r: 0.1186
p−value = 0.4659
DG 49:10
Mouse
Pearson's r: −0.0781
p−value = 0.632
Rat
Pearson's r: 0.0625
p−value = 0.6943
Human
Pearson's r: 0.3017
p−value = 0.0585
DG 49:12
Mouse
Pearson's r: 0.0197
p−value = 0.904
Rat
Pearson's r: 0.1227
p−value = 0.4389
Human
Pearson's r: −0.2816
p−value = 0.0783
DG 51:14
Mouse
Pearson's r: −0.222
p−value = 0.1685
Rat
Pearson's r: −0.0149
p−value = 0.9255
Human
Pearson's r: 0.0617
p−value = 0.7053
DG 52:10
Mouse
Pearson's r: −0.1519
p−value = 0.3495
Rat
Pearson's r: −0.0273
p−value = 0.8638
Human
Pearson's r: −0.1592
p−value = 0.3264
Normalized intensity
Log2(Time)
Donor
1
2
3
4
5
6

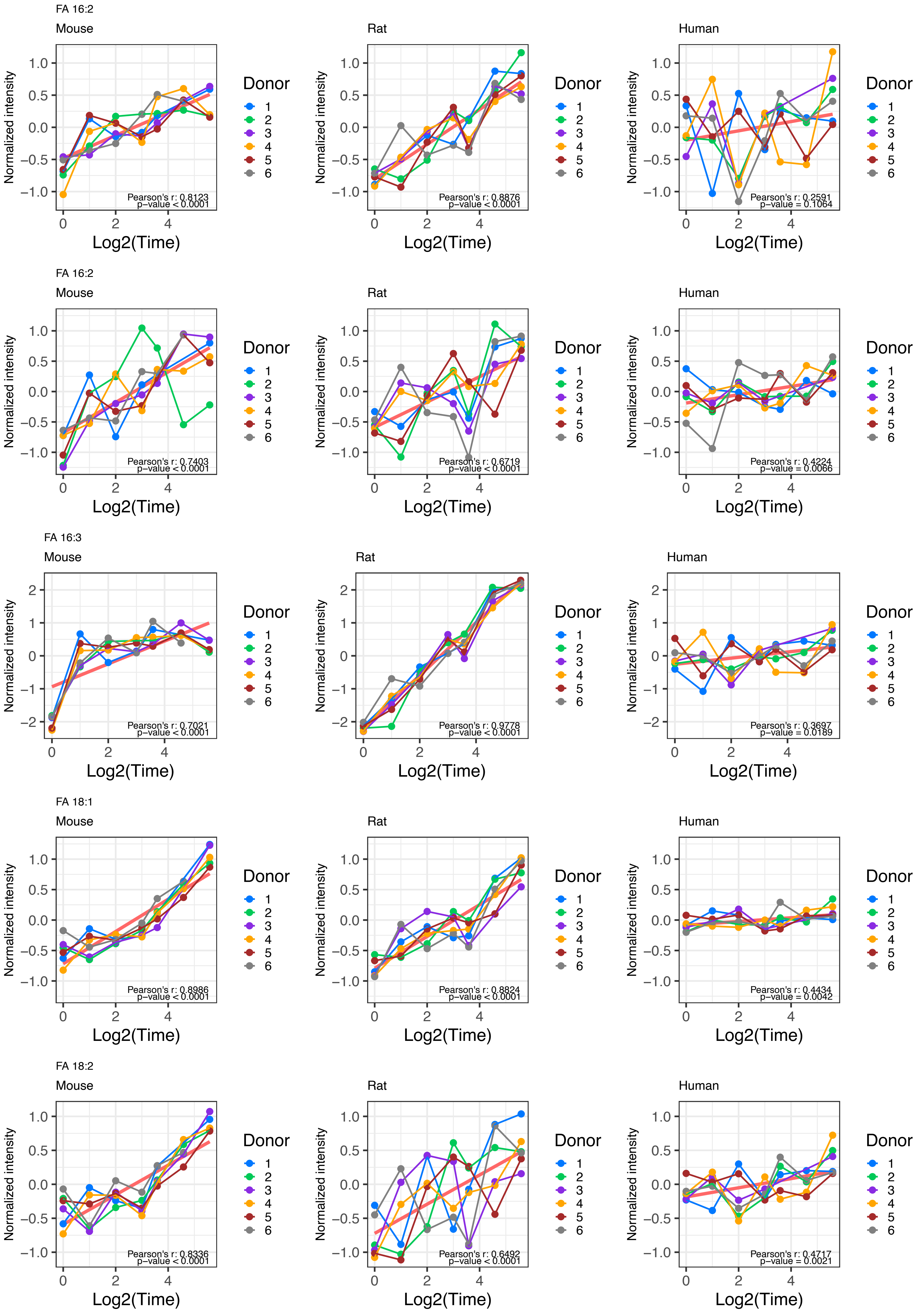
FA 16:2
Mouse
Pearson's r: 0.8123
p−value < 0.0001
Rat
Pearson's r: 0.8876
p−value < 0.0001
Human
Pearson's r: 0.2591
p−value = 0.1064
FA 16:2
Mouse
Pearson's r: 0.7403
p−value < 0.0001
Rat
Pearson's r: 0.6719
p−value < 0.0001
Human
Pearson's r: 0.4224
p−value = 0.0066
FA 16:3
Mouse
Pearson's r: 0.7021
p−value < 0.0001
Rat
Pearson's r: 0.9778
p−value < 0.0001
Human
Pearson's r: 0.3697
p−value = 0.0189
FA 18:1
Mouse
Pearson's r: 0.8986
p−value < 0.0001
Rat
Pearson's r: 0.8824
p−value < 0.0001
Human
Pearson's r: 0.4434
p−value = 0.0042
FA 18:2
Mouse
Pearson's r: 0.8336
p−value < 0.0001
Rat
Pearson's r: 0.6492
p−value < 0.0001
Human
Pearson's r: 0.4717
p−value = 0.0021
Normalized intensity
Log2(Time)
Donor
1
2
3
4
5
6

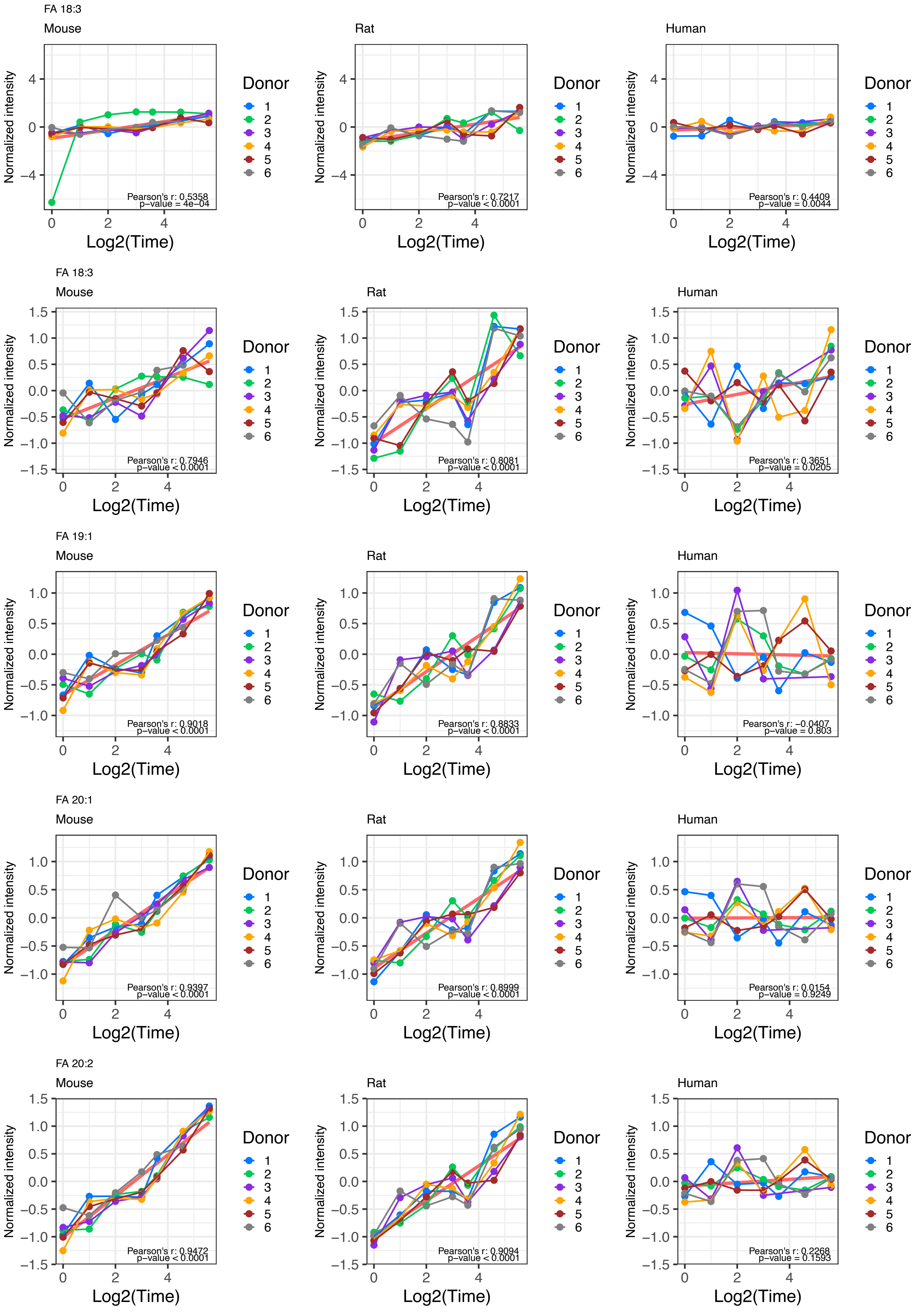
FA 18:3
Mouse
Rat
Human
Normalized intensity
Log2(Time)
Donor
1
2
3
4
5
6
Pearson's r: 0.5358
p−value = 4e−04
Pearson's r: 0.7217
p−value < 0.0001
Pearson's r: 0.4409
p−value = 0.0044
FA 18:3
Mouse
Rat
Human
Pearson's r: 0.7946
p−value < 0.0001
Pearson's r: 0.8081
p−value < 0.0001
Pearson's r: 0.3651
p−value = 0.0205
FA 19:1
Mouse
Rat
Human
Pearson's r: 0.9018
p−value < 0.0001
Pearson's r: 0.8833
p−value < 0.0001
Pearson's r: −0.0407
p−value = 0.803
FA 20:1
Mouse
Rat
Human
Pearson's r: 0.9397
p−value < 0.0001
Pearson's r: 0.8999
p−value < 0.0001
Pearson's r: 0.0154
p−value = 0.9249
FA 20:2
Mouse
Rat
Human
Pearson's r: 0.9472
p−value < 0.0001
Pearson's r: 0.9094
p−value < 0.0001
Pearson's r: 0.2268
p−value = 0.1593

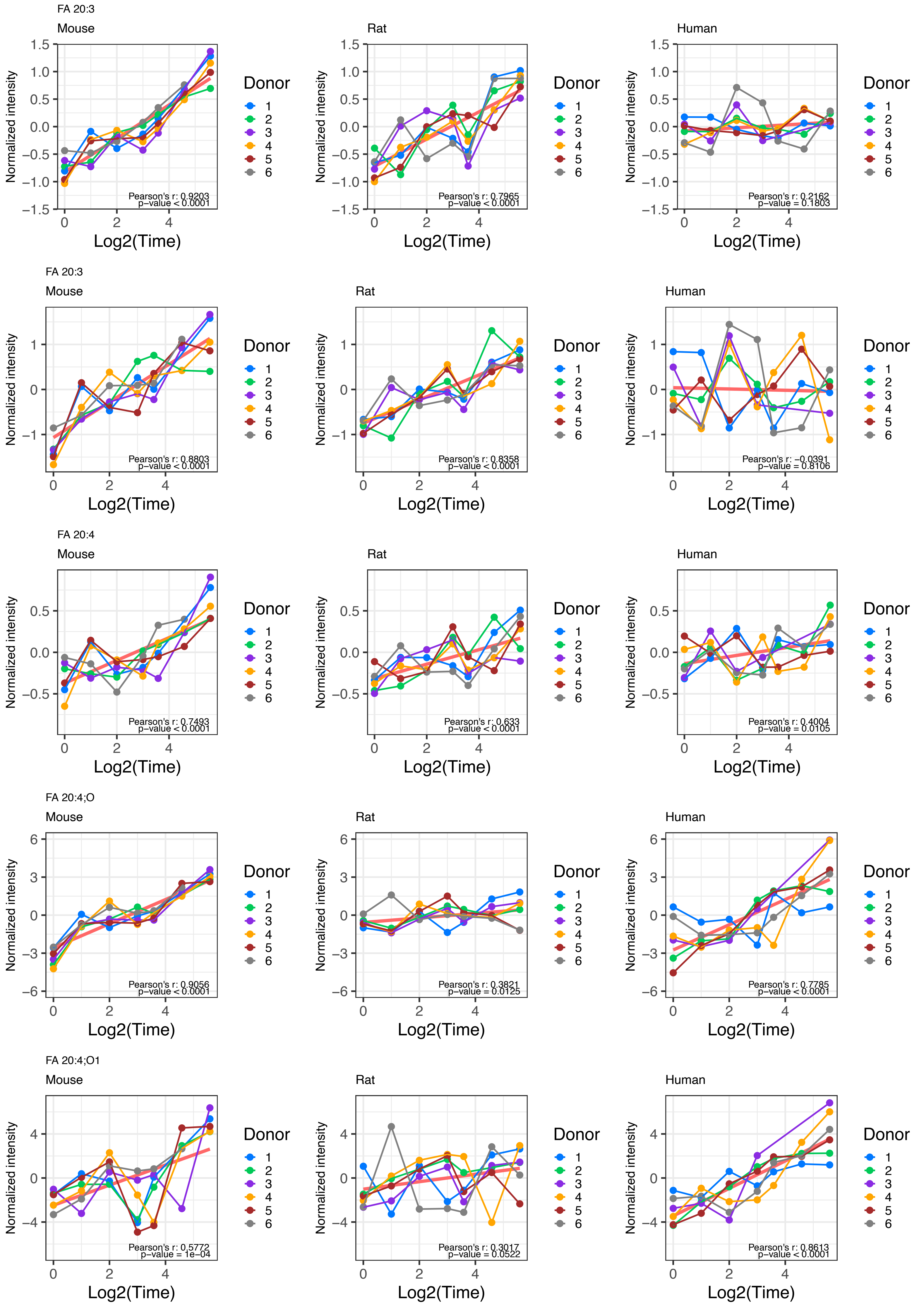

FA 20:3
Mouse
Rat
Human
Normalized intensity
Log2(Time)
Donor
1
2
3
4
5
6
Pearson's r: 0.9203
p−value < 0.0001
Pearson's r: 0.7965
p−value < 0.0001
Pearson's r: 0.2162
p−value = 0.1803
FA 20:3
Mouse
Rat
Human
Pearson's r: 0.8803
p−value < 0.0001
Pearson's r: 0.8358
p−value < 0.0001
Pearson's r: −0.0391
p−value = 0.8106
FA 20:4
Mouse
Rat
Human
Pearson's r: 0.7493
p−value < 0.0001
Pearson's r: 0.633
p−value < 0.0001
Pearson's r: 0.4004
p−value = 0.0105
FA 20:4;O
Mouse
Rat
Human
Pearson's r: 0.9056
p−value < 0.0001
Pearson's r: 0.3821
p−value = 0.0125
Pearson's r: 0.7785
p−value < 0.0001
FA 20:4;O1
Mouse
Rat
Human
Pearson's r: 0.5772
p−value = 1e−04
Pearson's r: 0.3017
p−value = 0.0522
Pearson's r: 0.8613
p−value < 0.0001

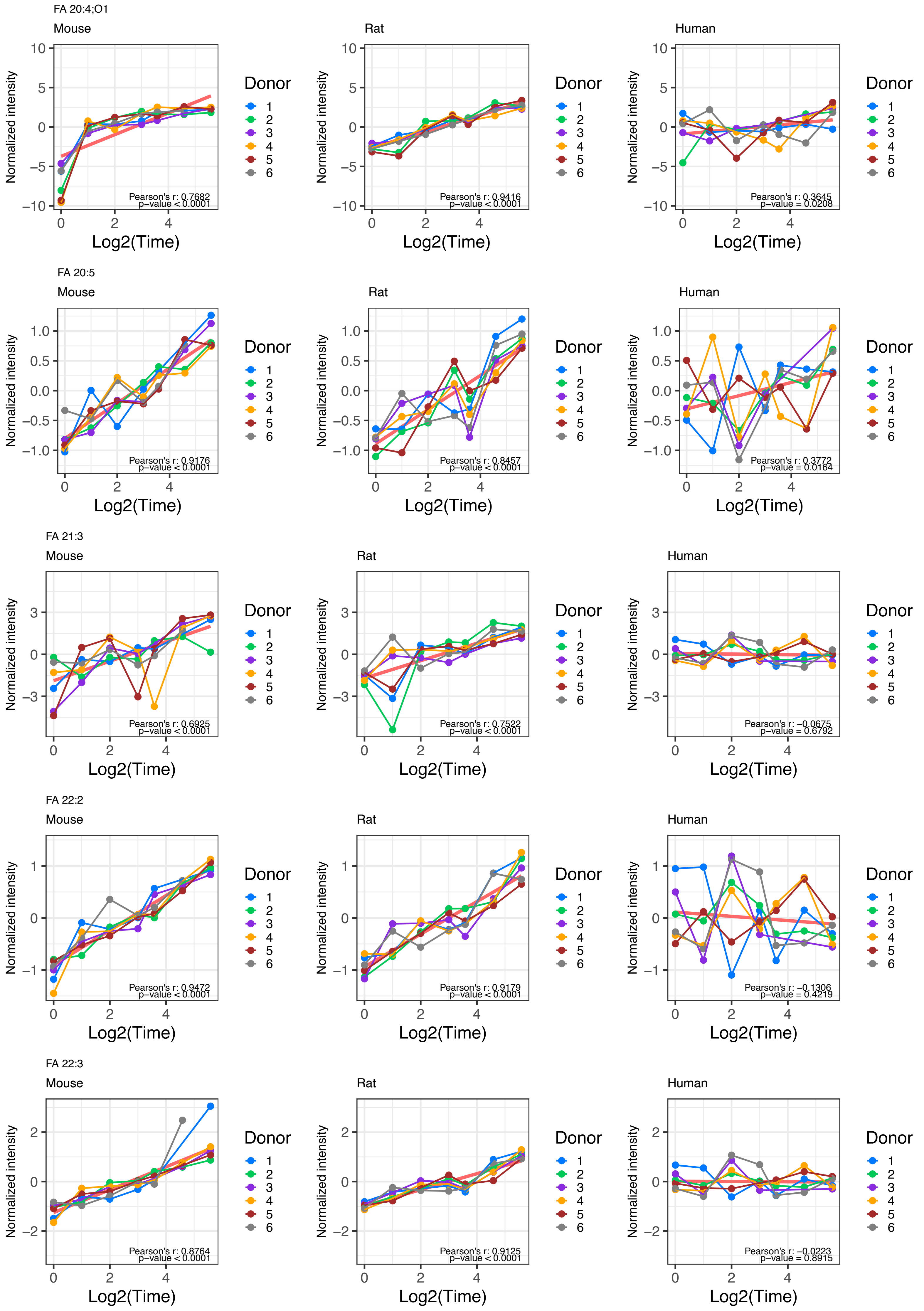
FA 20:4;O1
Mouse
Pearson's r: 0.7682
p−value < 0.0001
Rat
Pearson's r: 0.9416
p−value < 0.0001
Human
Pearson's r: 0.3645
p−value = 0.0208
FA 20:5
Mouse
Pearson's r: 0.9176
p−value < 0.0001
Rat
Pearson's r: 0.8457
p−value < 0.0001
Human
Pearson's r: 0.3772
p−value = 0.0164
FA 21:3
Mouse
Pearson's r: 0.6925
p−value < 0.0001
Rat
Pearson's r: 0.7522
p−value < 0.0001
Human
Pearson's r: −0.0675
p−value = 0.6792
FA 22:2
Mouse
Pearson's r: 0.9472
p−value < 0.0001
Rat
Pearson's r: 0.9179
p−value < 0.0001
Human
Pearson's r: −0.1306
p−value = 0.4219
FA 22:3
Mouse
Pearson's r: 0.8764
p−value < 0.0001
Rat
Pearson's r: 0.9125
p−value < 0.0001
Human
Pearson's r: −0.0223
p−value = 0.8915
Normalized intensity
Log2(Time)
Donor
1
2
3
4
5
6

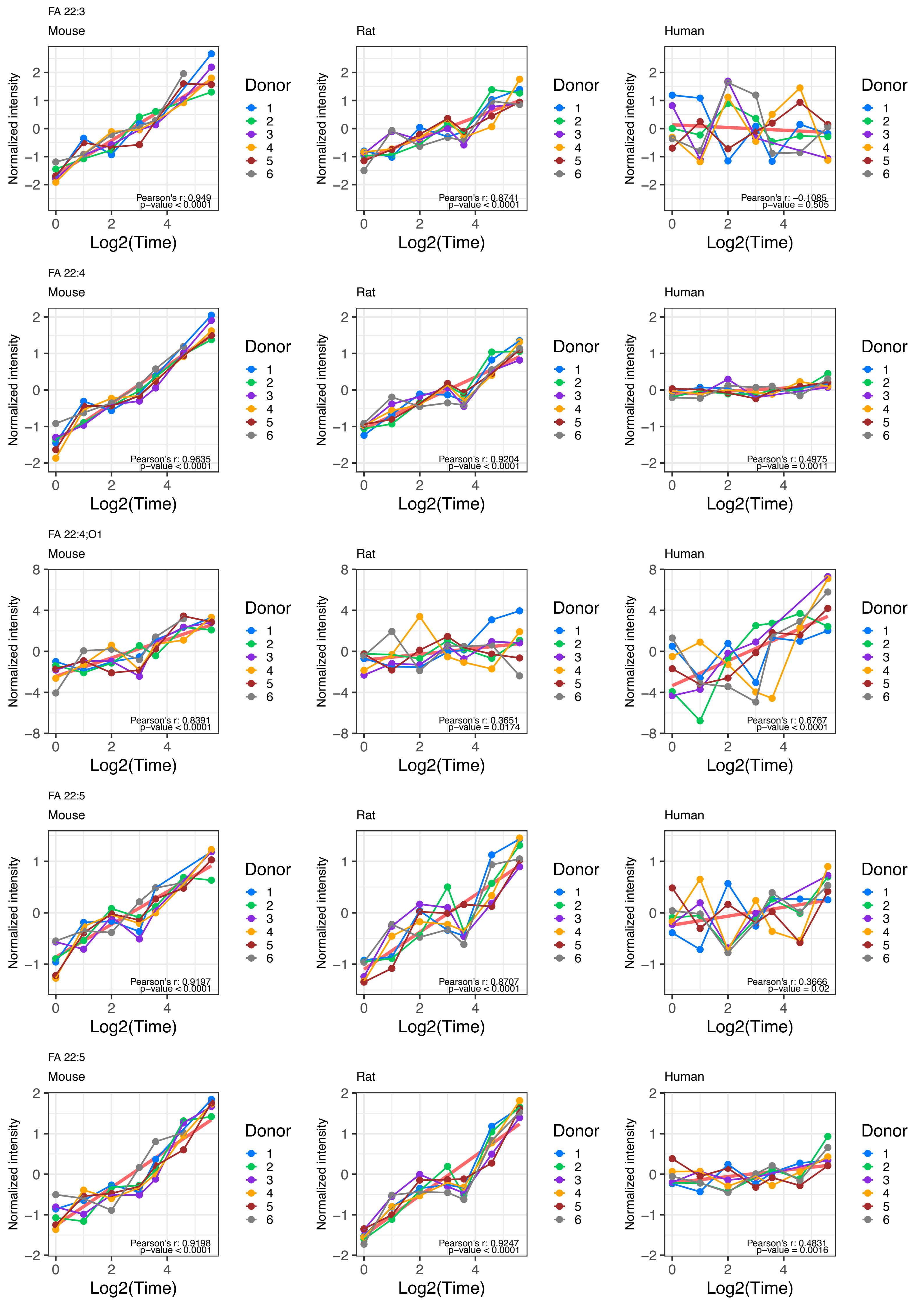
FA 22:3
Mouse
Pearson's r: 0.949
p−value < 0.0001
Rat
Pearson's r: 0.8741
p−value < 0.0001
Human
Pearson's r: −0.1085
p−value = 0.505
FA 22:4
Mouse
Pearson's r: 0.9635
p−value < 0.0001
Rat
Pearson's r: 0.9204
p−value < 0.0001
Human
Pearson's r: 0.4975
p−value = 0.0011
FA 22:4;O1
Mouse
Pearson's r: 0.8391
p−value < 0.0001
Rat
Pearson's r: 0.3651
p−value = 0.0174
Human
Pearson's r: 0.6767
p−value < 0.0001
FA 22:5
Mouse
Pearson's r: 0.9197
p−value < 0.0001
Rat
Pearson's r: 0.8707
p−value < 0.0001
Human
Pearson's r: 0.3666
p−value = 0.02
FA 22:5
Mouse
Pearson's r: 0.9198
p−value < 0.0001
Rat
Pearson's r: 0.9247
p−value < 0.0001
Human
Pearson's r: 0.4831
p−value = 0.0016
Normalized intensity
Log2(Time)
Donor
1
2
3
4
5
6

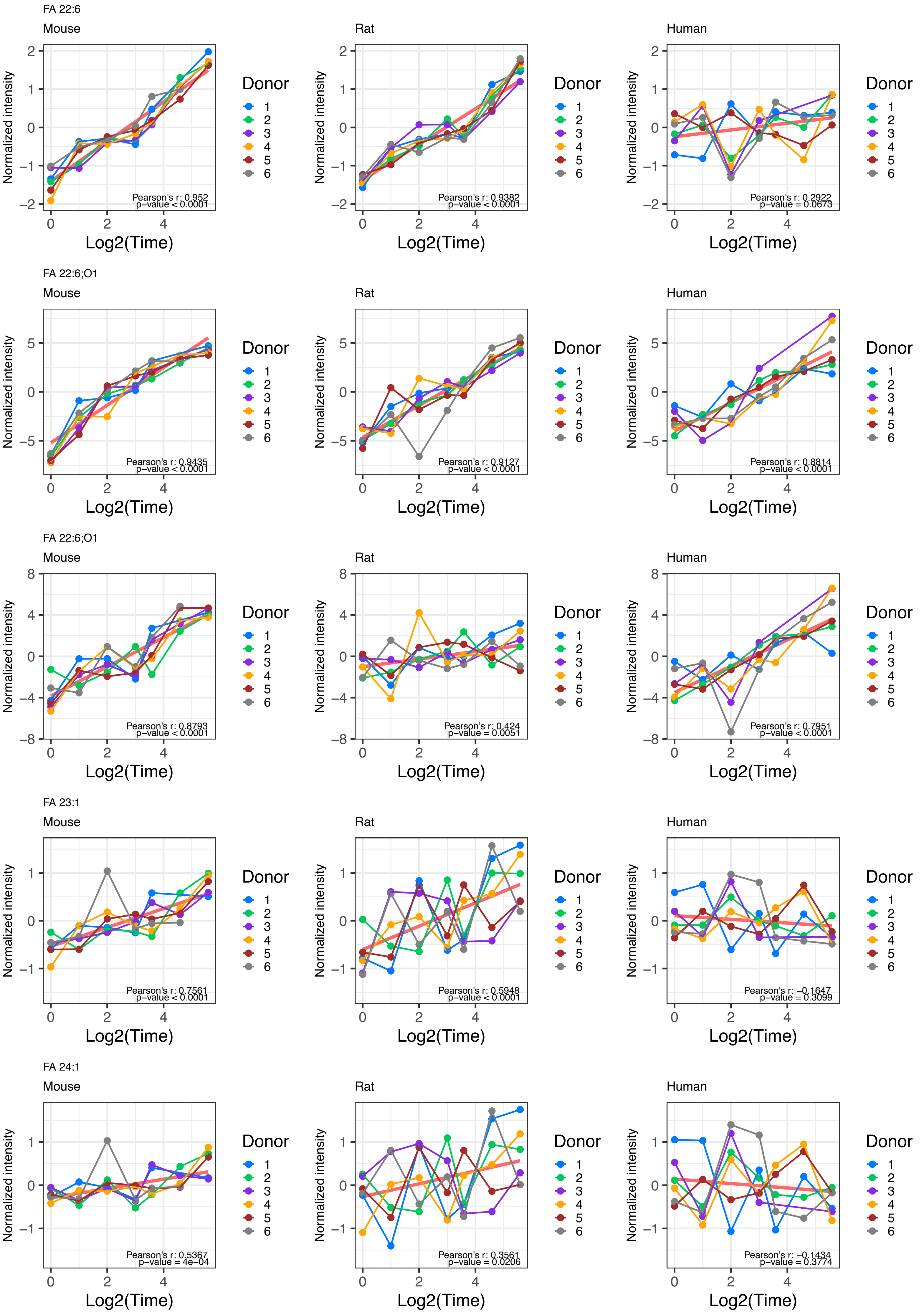
FA 22:6
Mouse
Pearson's r: 0.952
p−value < 0.0001
Rat
Pearson's r: 0.9382
p−value < 0.0001
Human
Pearson's r: 0.2922
p−value = 0.0673
FA 22:6;O1
Mouse
Pearson's r: 0.9435
p−value < 0.0001
Rat
Pearson's r: 0.9127
p−value < 0.0001
Human
Pearson's r: 0.8814
p−value < 0.0001
FA 22:6;O1
Mouse
Pearson's r: 0.8793
p−value < 0.0001
Rat
Pearson's r: 0.424
p−value = 0.0051
Human
Pearson's r: 0.7951
p−value < 0.0001
FA 23:1
Mouse
Pearson's r: 0.7561
p−value < 0.0001
Rat
Pearson's r: 0.5948
p−value < 0.0001
Human
Pearson's r: −0.1647
p−value = 0.3099
FA 24:1
Mouse
Pearson's r: 0.5367
p−value = 4e−04
Rat
Pearson's r: 0.3561
p−value = 0.0206
Human
Pearson's r: −0.1434
p−value = 0.3774
Normalized intensity
Log2(Time)
Donor
1
2
3
4
5
6

FA 24:1(2OH)

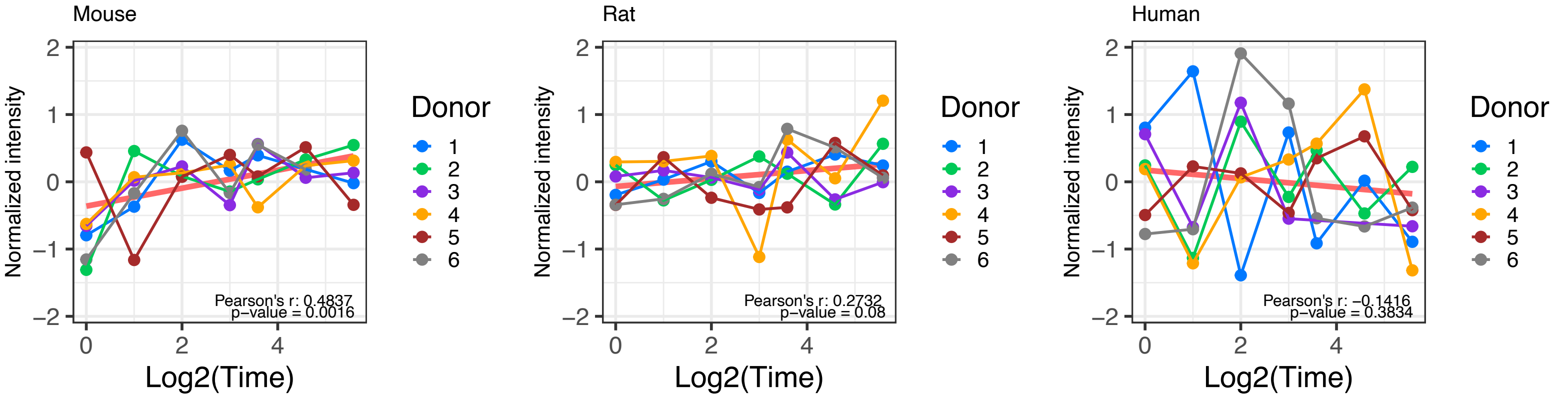

FA 24:2

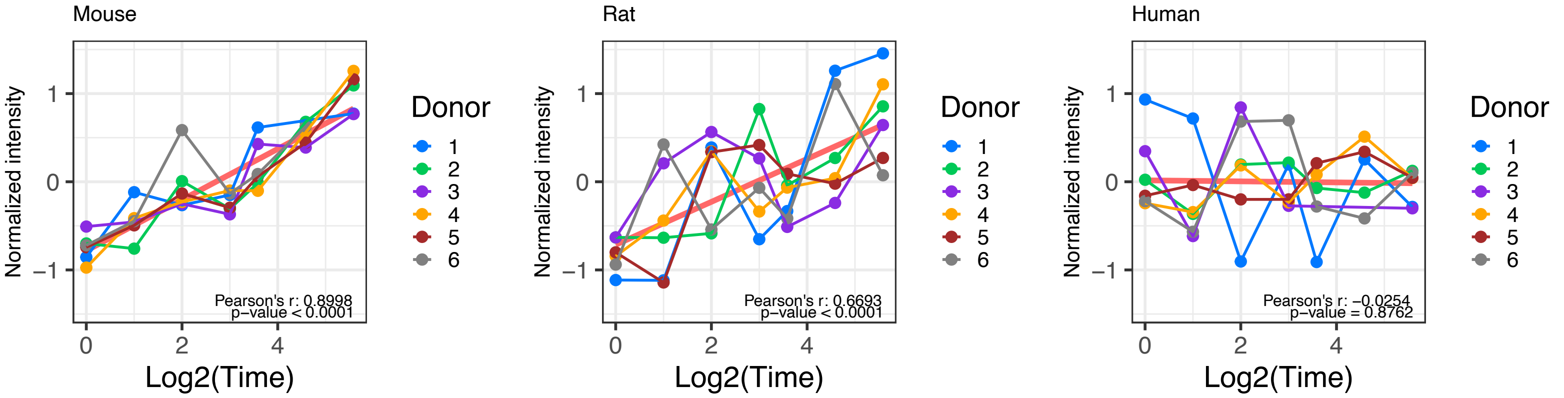

FA 24:3

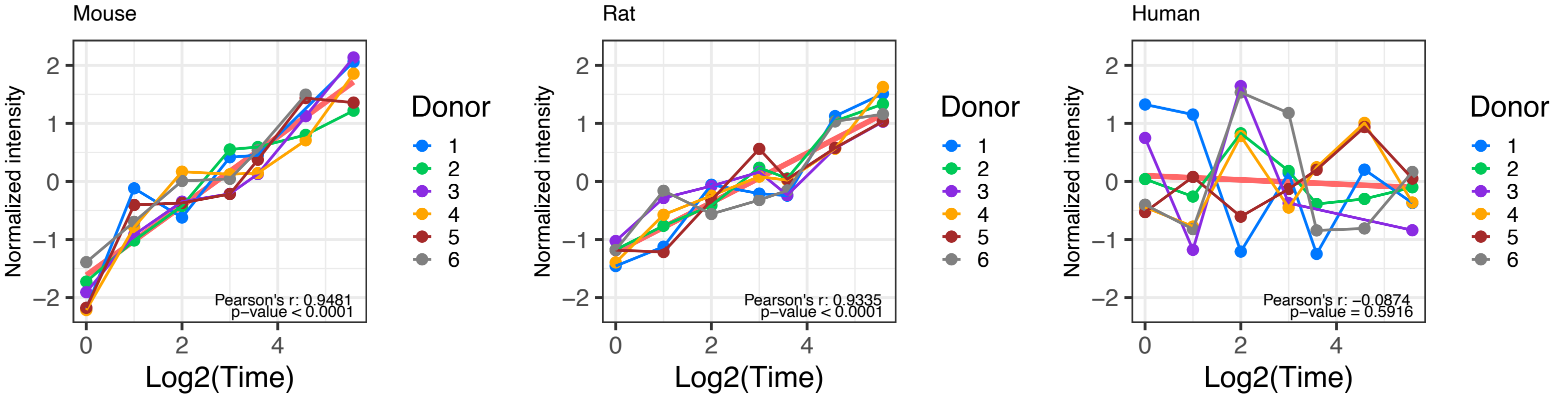

FA 24:4

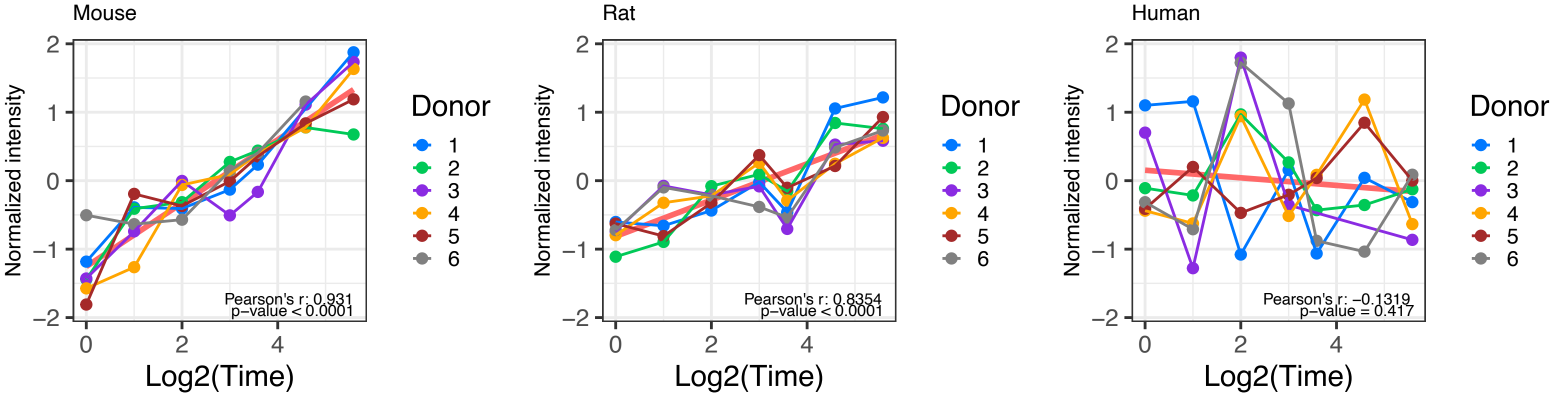

FA 24:5

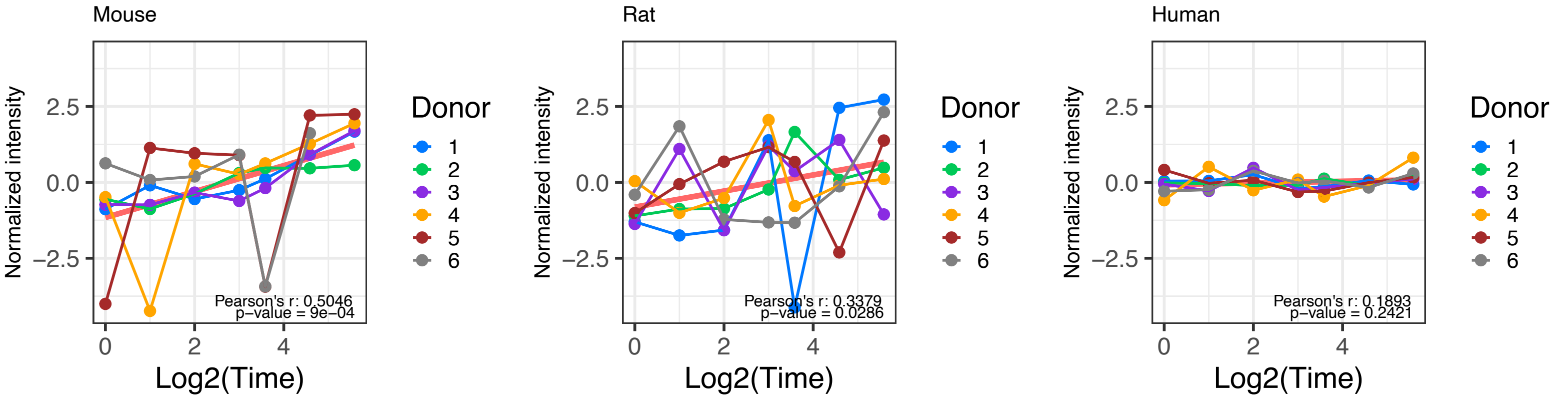

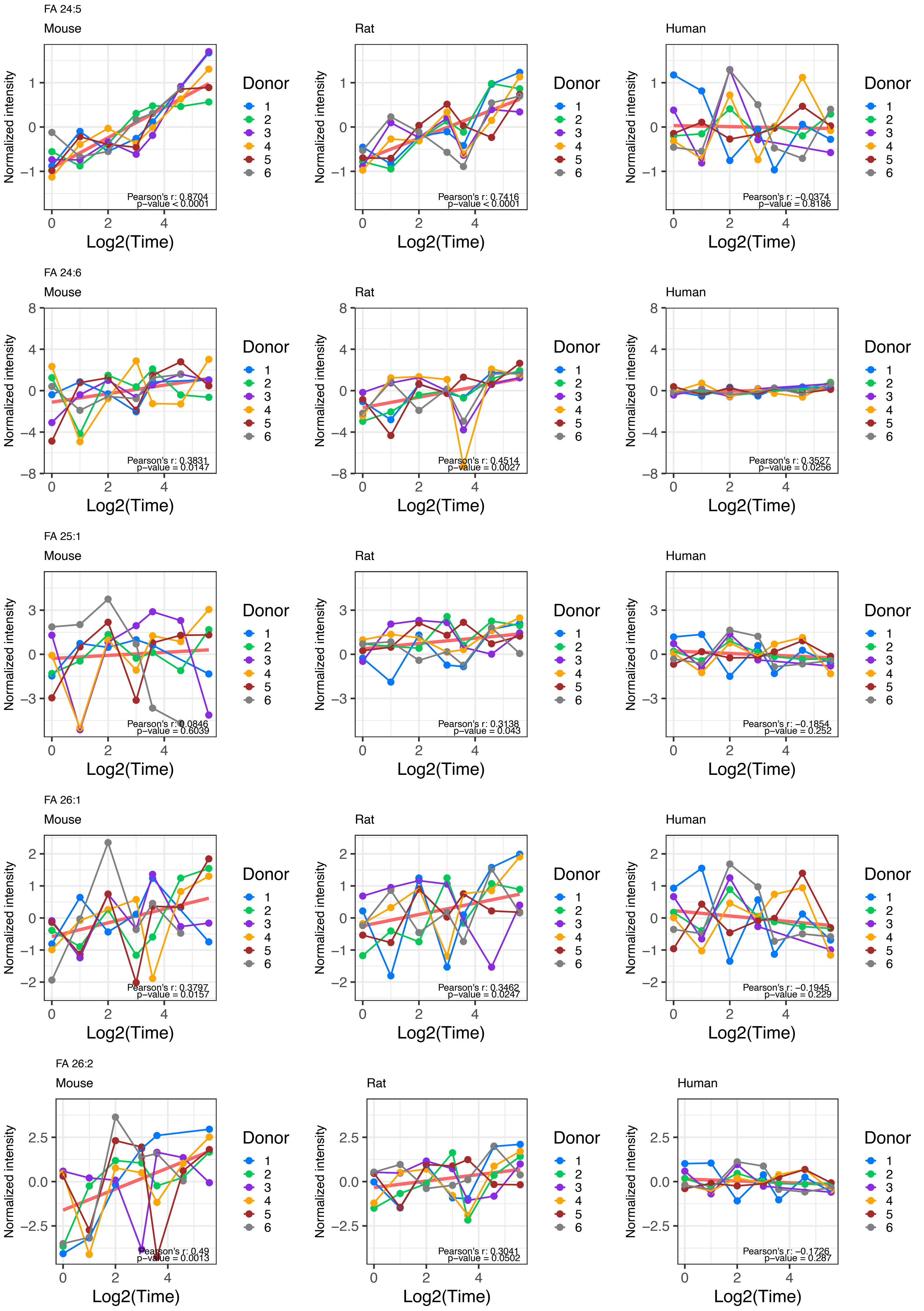
FA 24:5
Mouse
Pearson's r: 0.8704
p−value < 0.0001
Rat
Pearson's r: 0.7416
p−value < 0.0001
Human
Pearson's r: −0.0374
p−value = 0.8186
FA 24:6
Mouse
Pearson's r: 0.3831
p−value = 0.0147
Rat
Pearson's r: 0.4514
p−value = 0.0027
Human
Pearson's r: 0.3527
p−value = 0.0256
FA 25:1
Mouse
Pearson's r: 0.0846
p−value = 0.6039
Rat
Pearson's r: 0.3138
p−value = 0.043
Human
Pearson's r: −0.1854
p−value = 0.252
FA 26:1
Mouse
Pearson's r: 0.3797
p−value = 0.0157
Rat
Pearson's r: 0.3462
p−value = 0.0247
Human
Pearson's r: −0.1945
p−value = 0.229
FA 26:2
Mouse
Pearson's r: 0.49
p−value = 0.0013
Rat
Pearson's r: 0.3041
p−value = 0.0502
Human
Pearson's r: −0.1726
p−value = 0.287
Normalized intensity
Log2(Time)
Donor
1
2
3
4
5
6

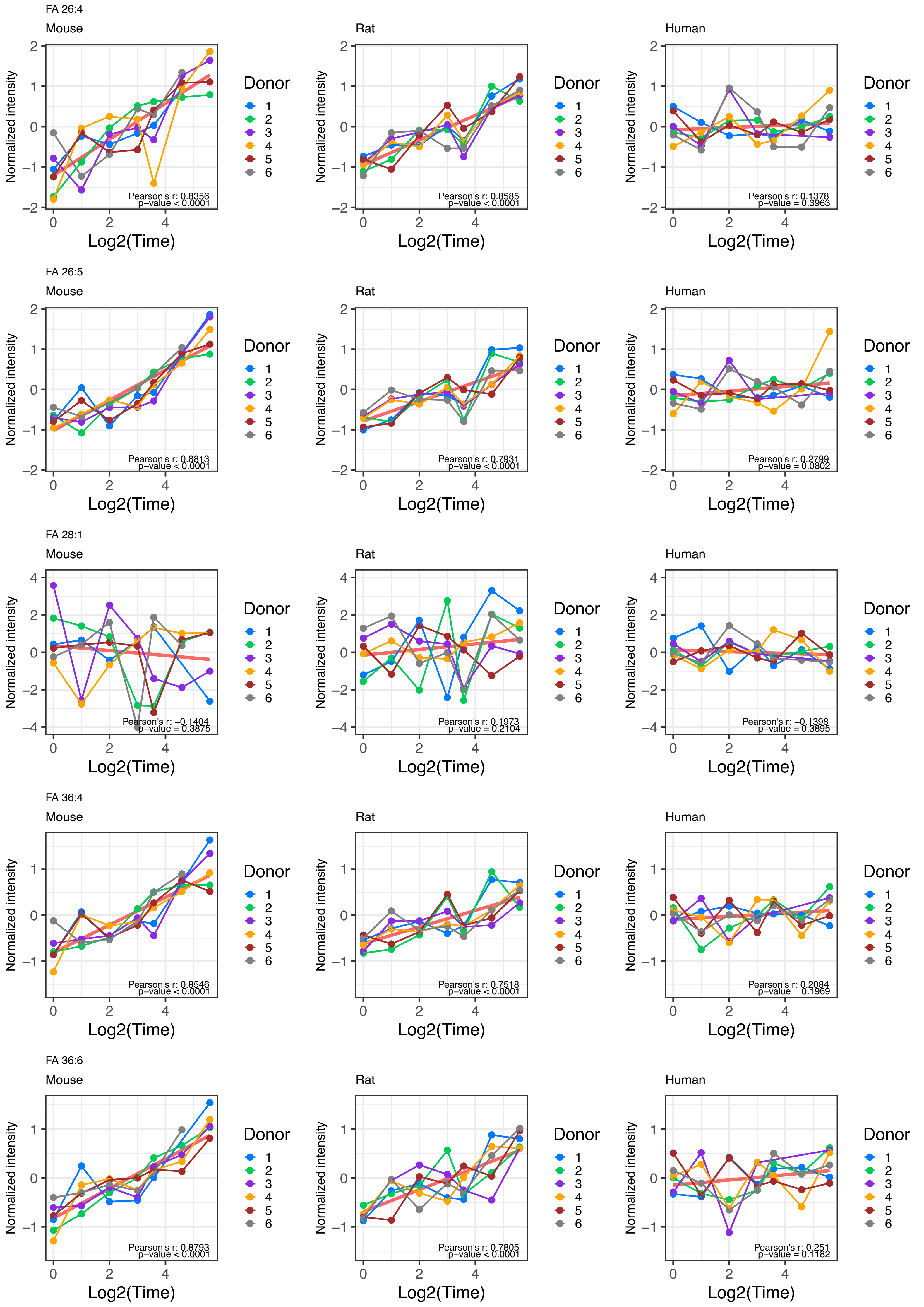
FA 26:4
Mouse
Rat
Human
Normalized intensity
Log2(Time)
Donor
1
2
3
4
5
6
Pearson's r: 0.8356
p−value < 0.0001
Pearson's r: 0.8585
p−value < 0.0001
Pearson's r: 0.1378
p−value = 0.3963
FA 26:5
Mouse
Rat
Human
Pearson's r: 0.8813
p−value < 0.0001
Pearson's r: 0.7931
p−value < 0.0001
Pearson's r: 0.2799
p−value = 0.0802
FA 28:1
Mouse
Rat
Human
Pearson's r: −0.1404
p−value = 0.3875
Pearson's r: 0.1973
p−value = 0.2104
Pearson's r: −0.1398
p−value = 0.3895
FA 36:4
Mouse
Rat
Human
Pearson's r: 0.8546
p−value < 0.0001
Pearson's r: 0.7518
p−value < 0.0001
Pearson's r: 0.2084
p−value = 0.1969
FA 36:6
Mouse
Rat
Human
Pearson's r: 0.8793
p−value < 0.0001
Pearson's r: 0.7805
p−value < 0.0001
Pearson's r: 0.251
p−value = 0.1182

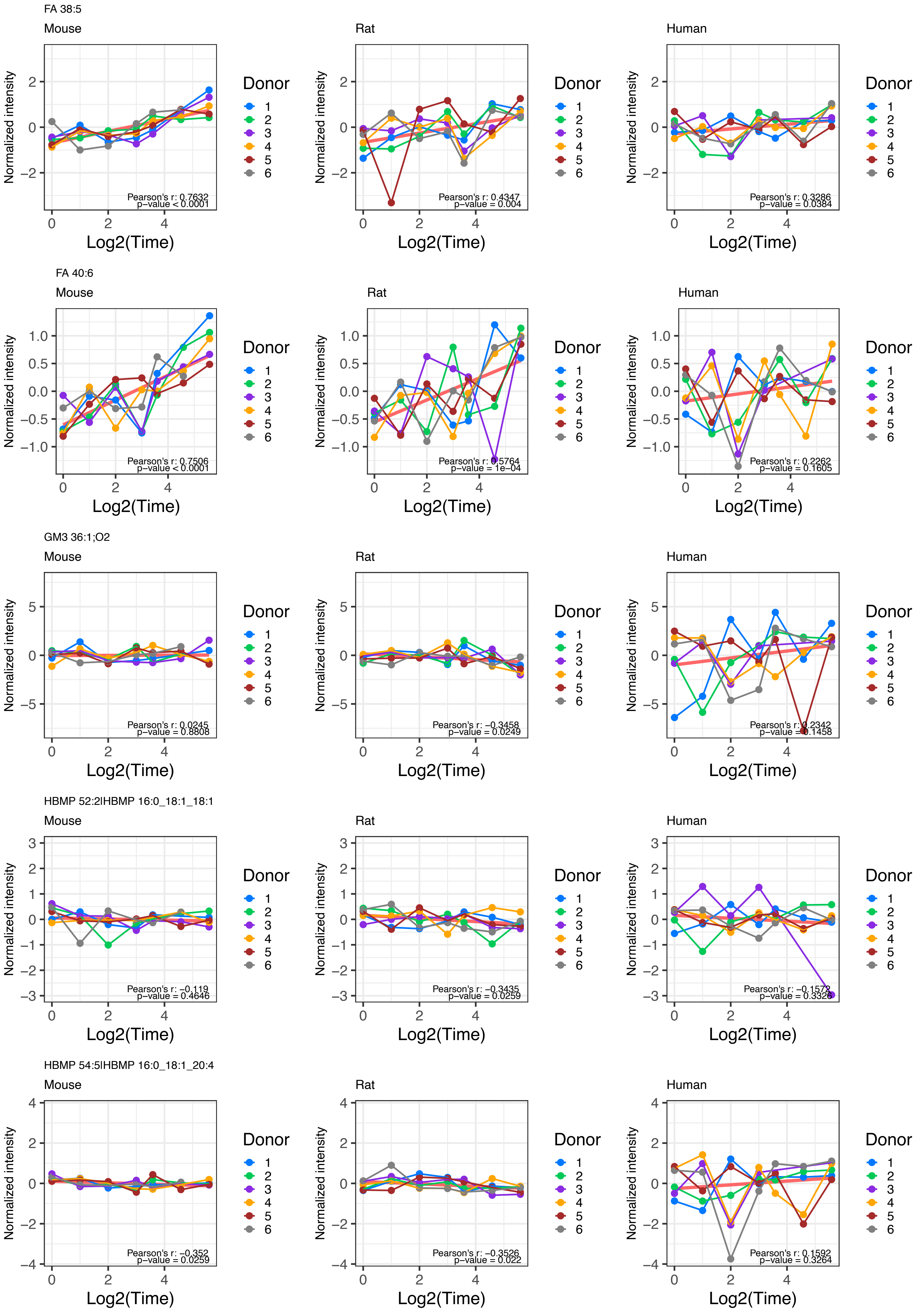

FA 38:5
Mouse
Pearson's r: 0.7632
p−value < 0.0001
Rat
Pearson's r: 0.4347
p−value = 0.004
Human
Pearson's r: 0.3286
p−value = 0.0384
FA 40:6
Mouse
Pearson's r: 0.7506
p−value < 0.0001
Rat
Pearson's r: 0.5764
p−value = 1e−04
Human
Pearson's r: 0.2262
p−value = 0.1605
GM3 36:1;O2
Mouse
Pearson's r: 0.0245
p−value = 0.8808
Rat
Pearson's r: −0.3458
p−value = 0.0249
Human
Pearson's r: 0.2342
p−value = 0.1458
HBMP 52:2|HBMP 16:0_18:1_18:1
Mouse
Pearson's r: −0.119
p−value = 0.4646
Rat
Pearson's r: −0.3435
p−value = 0.0259
Human
Pearson's r: −0.1572
p−value = 0.3326
HBMP 54:5|HBMP 16:0_18:1_20:4
Mouse
Pearson's r: −0.352
p−value = 0.0259
Rat
Pearson's r: −0.3526
p−value = 0.022
Human
Pearson's r: 0.1592
p−value = 0.3264
Normalized intensity
Log2(Time)
Donor
1
2
3
4
5
6

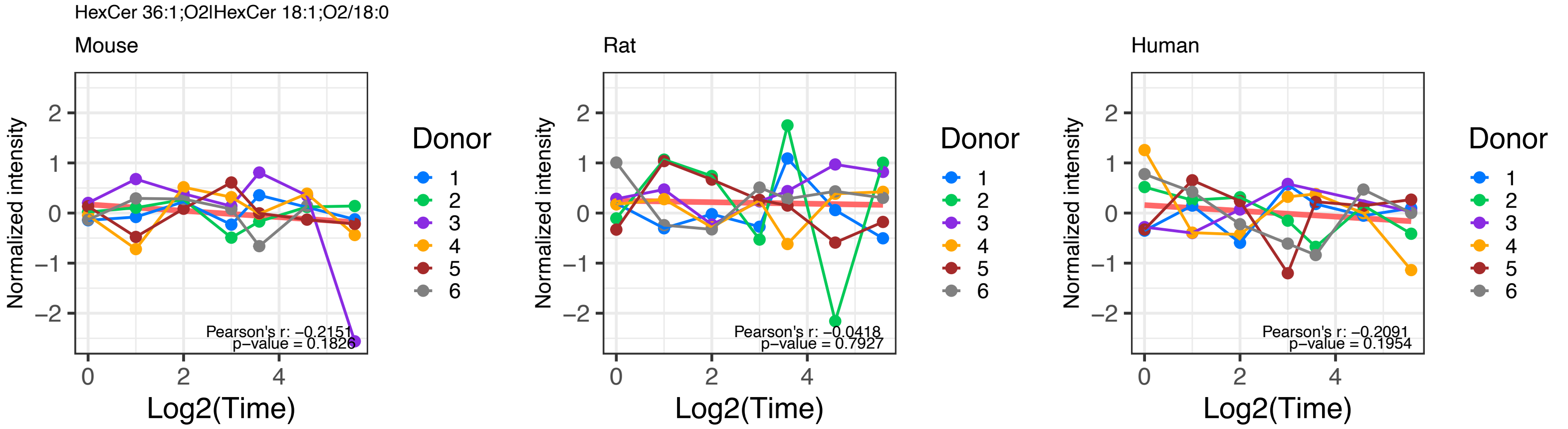
HexCer 36:1;O2|HexCer 18:1;O2/18:0
Mouse
Rat
Human
Normalized intensity
Log2(Time)
Donor
Pearson's r: −0.2151
p−value = 0.1826
Pearson's r: −0.0418
p−value = 0.7927
Pearson's r: −0.2091
p−value = 0.1954

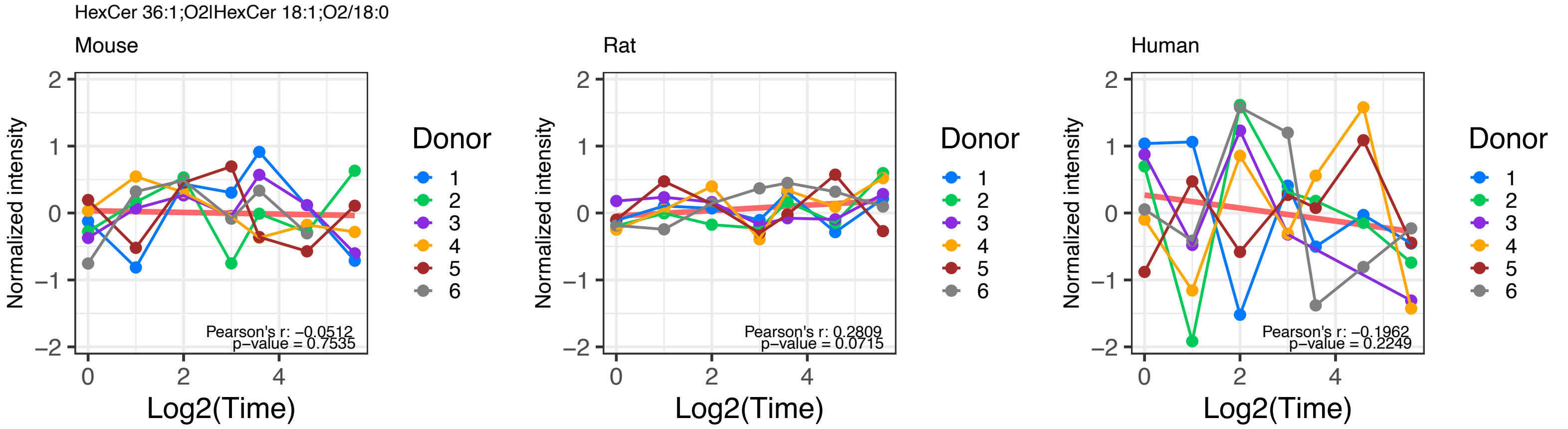
HexCer 36:1;O2|HexCer 18:1;O2/18:0
Mouse
Rat
Human
Normalized intensity
Log2(Time)
Donor
Pearson's r: −0.0512
p−value = 0.7535
Pearson's r: 0.2809
p−value = 0.0715
Pearson's r: −0.1962
p−value = 0.2249

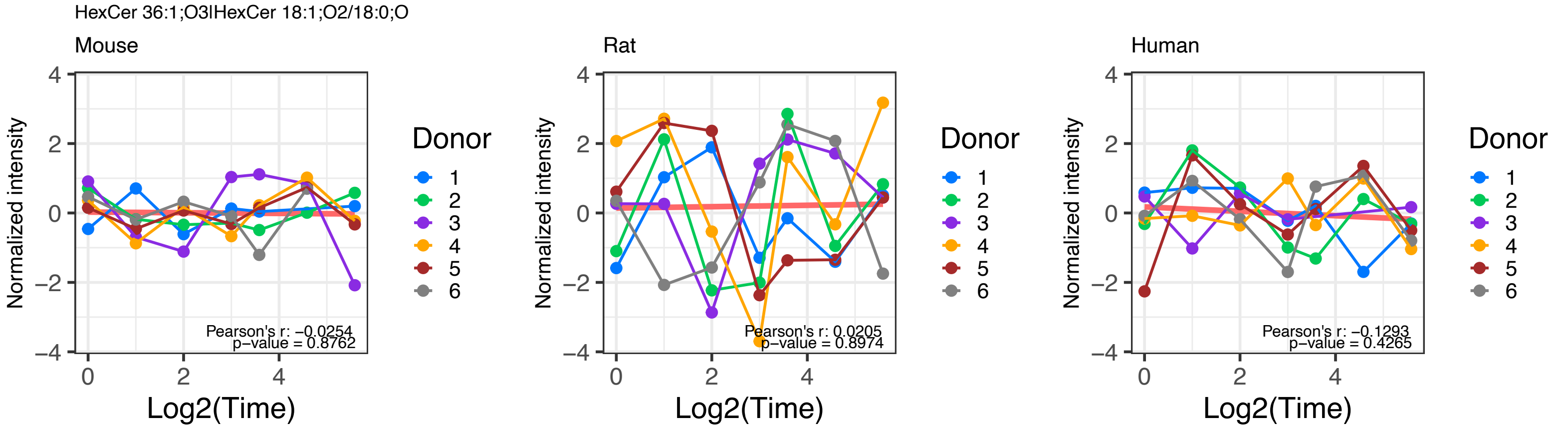
HexCer 36:1;O3|HexCer 18:1;O2/18:0;O
Mouse
Rat
Human
Normalized intensity
Log2(Time)
Donor
Pearson's r: −0.0254
p−value = 0.8762
Pearson's r: 0.0205
p−value = 0.8974
Pearson's r: −0.1293
p−value = 0.4265

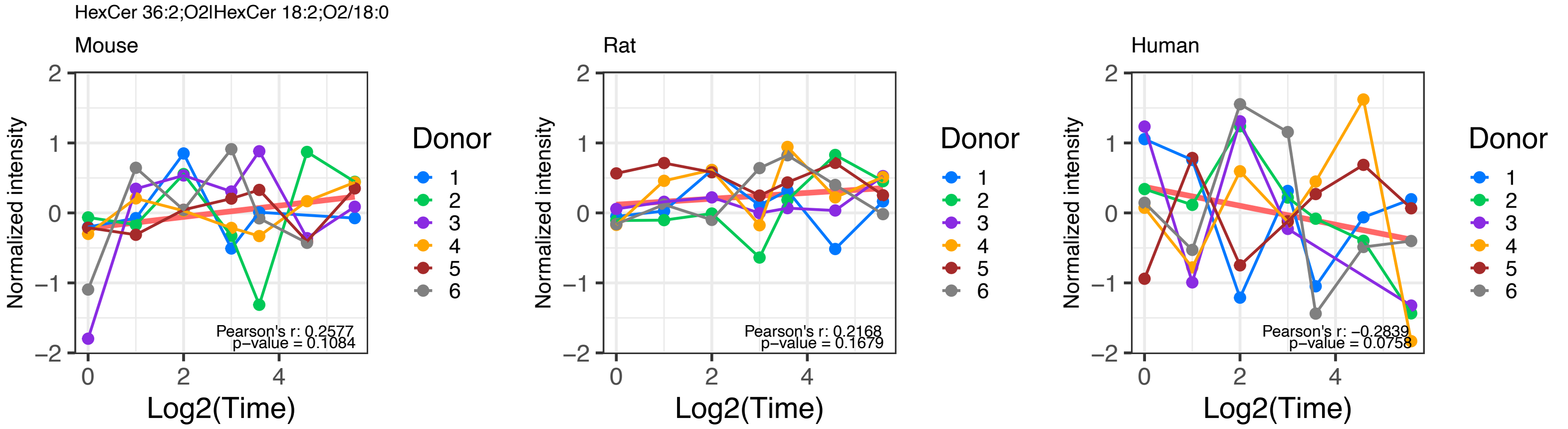
HexCer 36:2;O2|HexCer 18:2;O2/18:0
Mouse
Rat
Human
Normalized intensity
Log2(Time)
Donor
Pearson's r: 0.2577
p−value = 0.1084
Pearson's r: 0.2168
p−value = 0.1679
Pearson's r: −0.2839
p−value = 0.0758

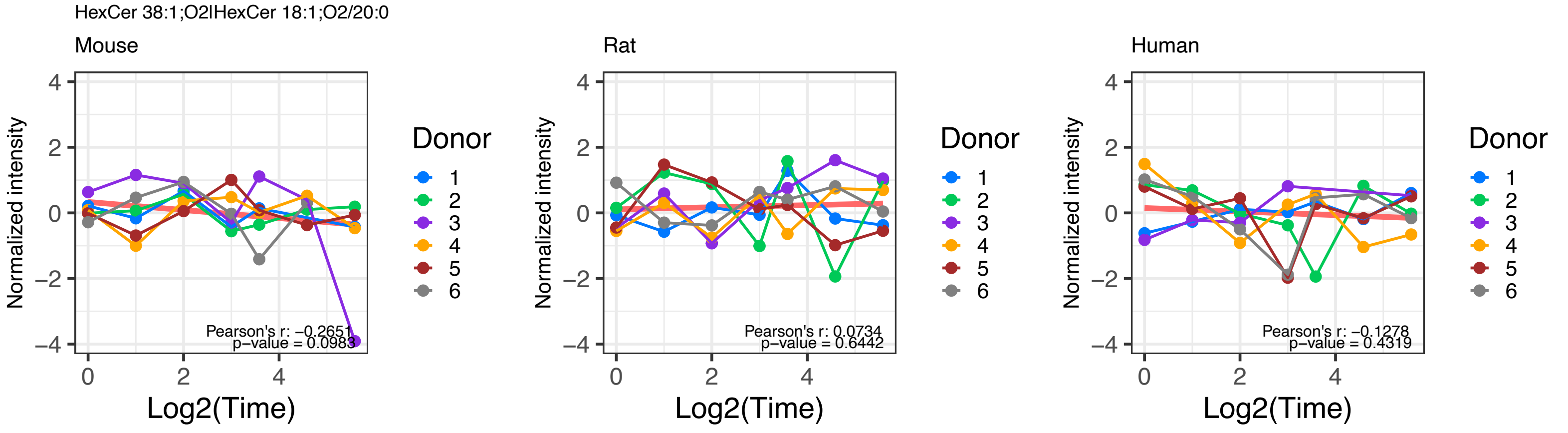
HexCer 38:1;O2|HexCer 18:1;O2/20:0
Mouse
Rat
Human
Normalized intensity
Log2(Time)
Donor
Pearson's r: −0.2651
p−value = 0.0983
Pearson's r: 0.0734
p−value = 0.6442
Pearson's r: −0.1278
p−value = 0.4319

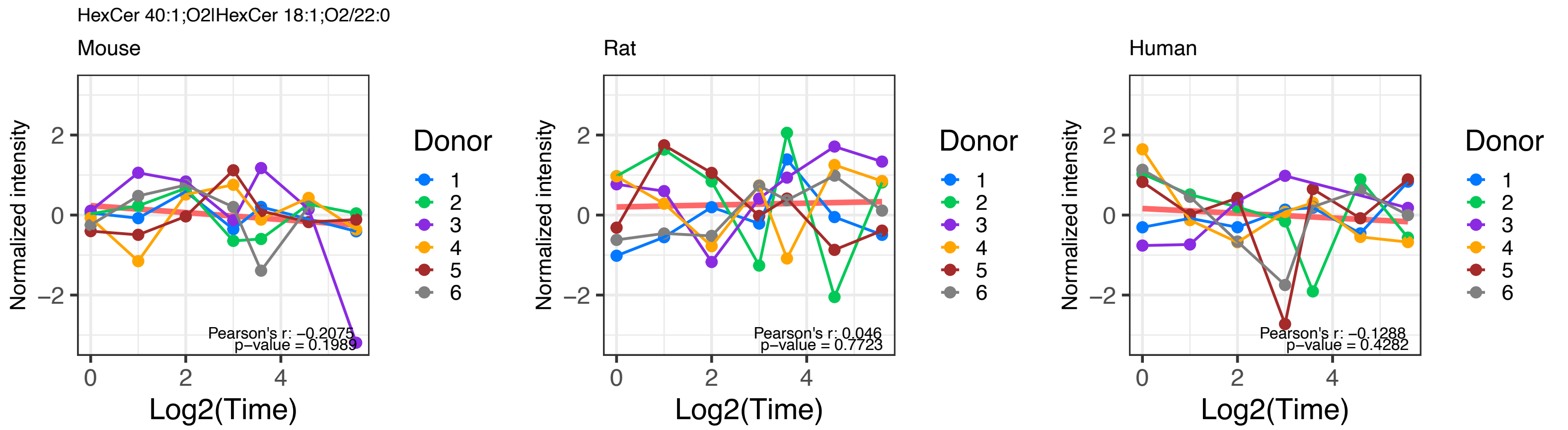
HexCer 40:1;O2|HexCer 18:1;O2/22:0
Mouse
Rat
Human
Normalized intensity
Log2(Time)
Donor
1
2
3
4
5
6
Pearson's r: −0.2075
p−value = 0.1989
Pearson's r: 0.046
p−value = 0.7723
Pearson's r: −0.1288
p−value = 0.4282

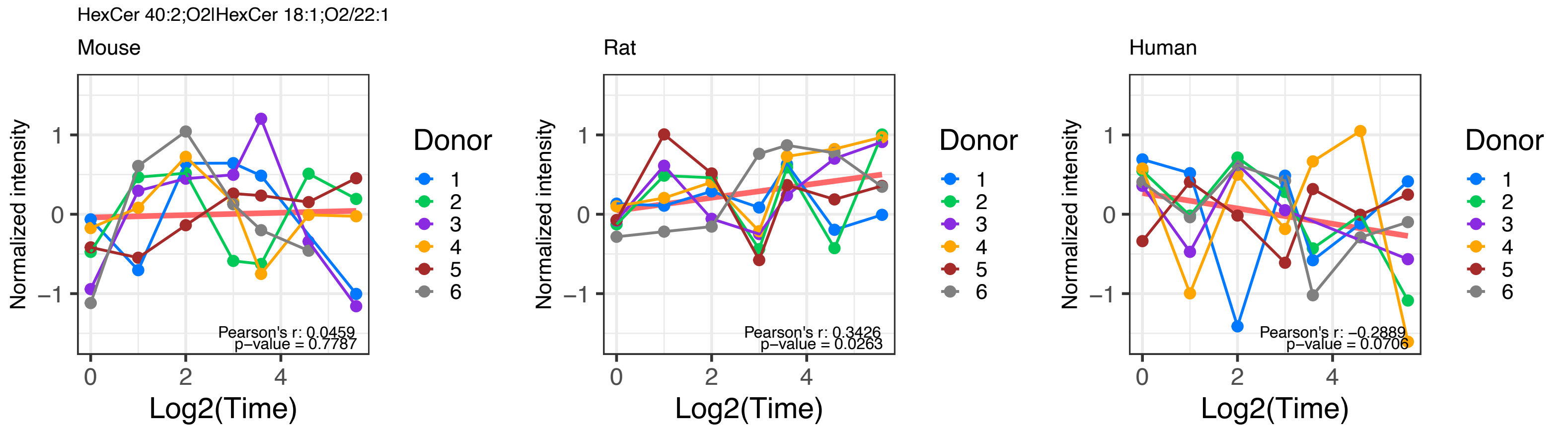
HexCer 40:2;O2|HexCer 18:1;O2/22:1
Mouse
Rat
Human
Normalized intensity
Log2(Time)
Donor
1
2
3
4
5
6
Pearson's r: 0.0459
p−value = 0.7787
Pearson's r: 0.3426
p−value = 0.0263
Pearson's r: −0.2889
p−value = 0.0706

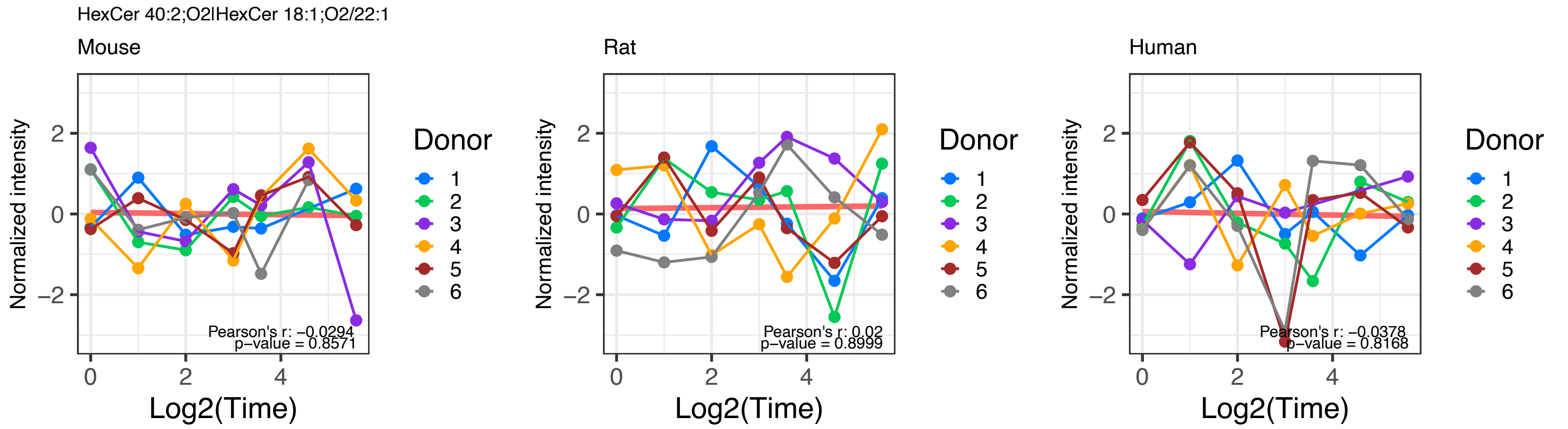
HexCer 40:2;O2|HexCer 18:1;O2/22:1
Mouse
Rat
Human
Normalized intensity
Log2(Time)
Donor
1
2
3
4
5
6
Pearson's r: −0.0294
p−value = 0.8571
Pearson's r: 0.02
p−value = 0.8999
Pearson's r: −0.0378
p−value = 0.8168

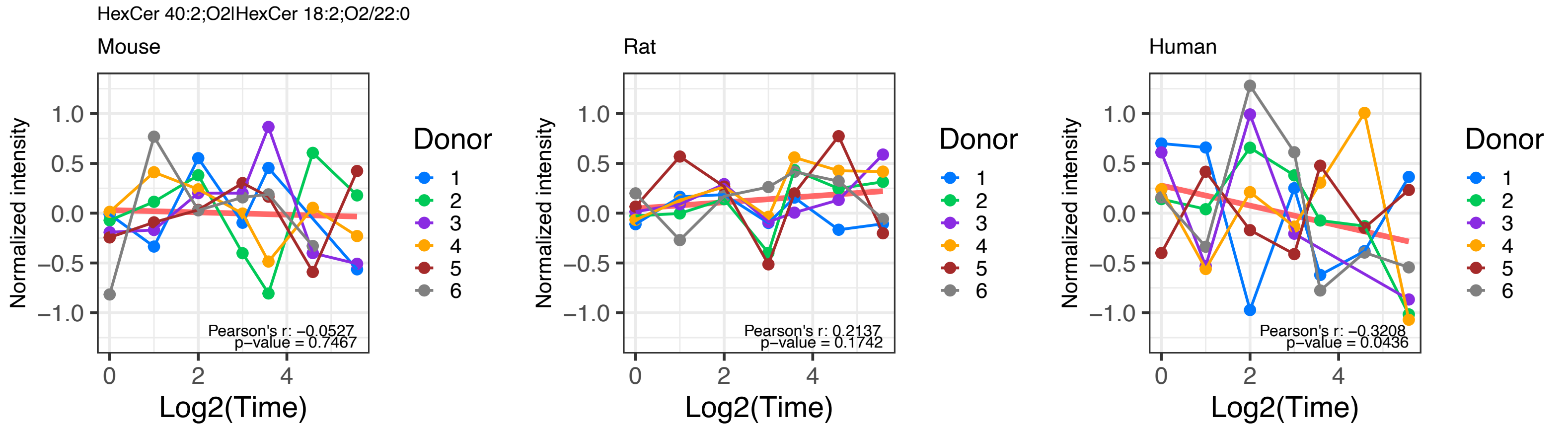
HexCer 40:2;O2|HexCer 18:2;O2/22:0
Mouse
Rat
Human
Normalized intensity
Log2(Time)
Donor
1
2
3
4
5
6
Pearson's r: −0.0527
p−value = 0.7467
Pearson's r: 0.2137
p−value = 0.1742
Pearson's r: −0.3208
p−value = 0.0436

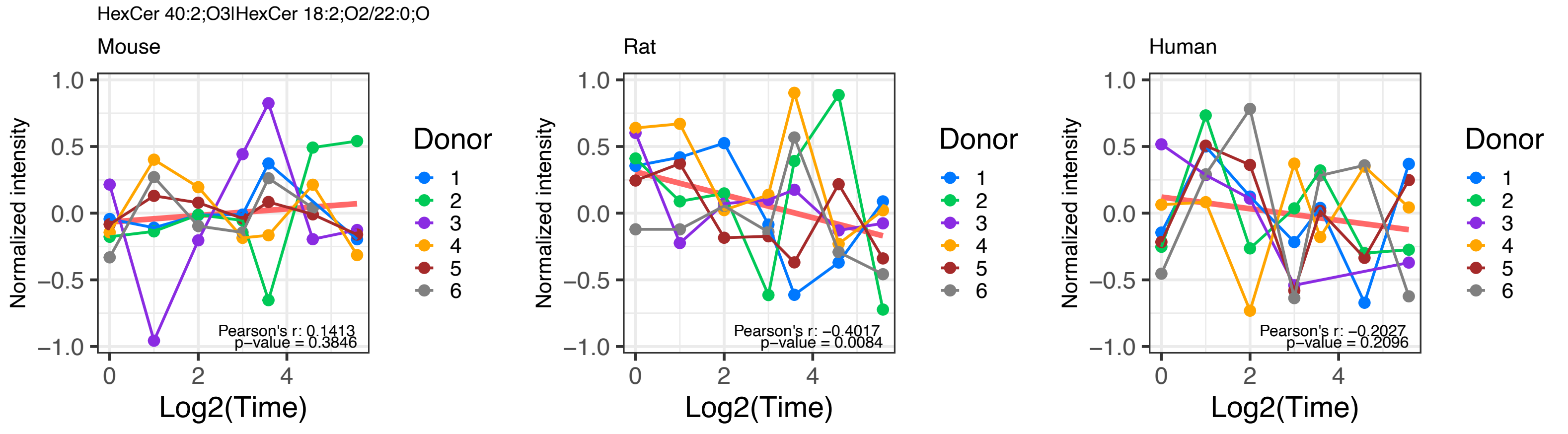
HexCer 40:2;O3|HexCer 18:2;O2/22:0;O
Mouse
Rat
Human
Normalized intensity
Log2(Time)
Donor
1
2
3
4
5
6
Pearson's r: 0.1413
p−value = 0.3846
Pearson's r: −0.4017
p−value = 0.0084
Pearson's r: −0.2027
p−value = 0.2096

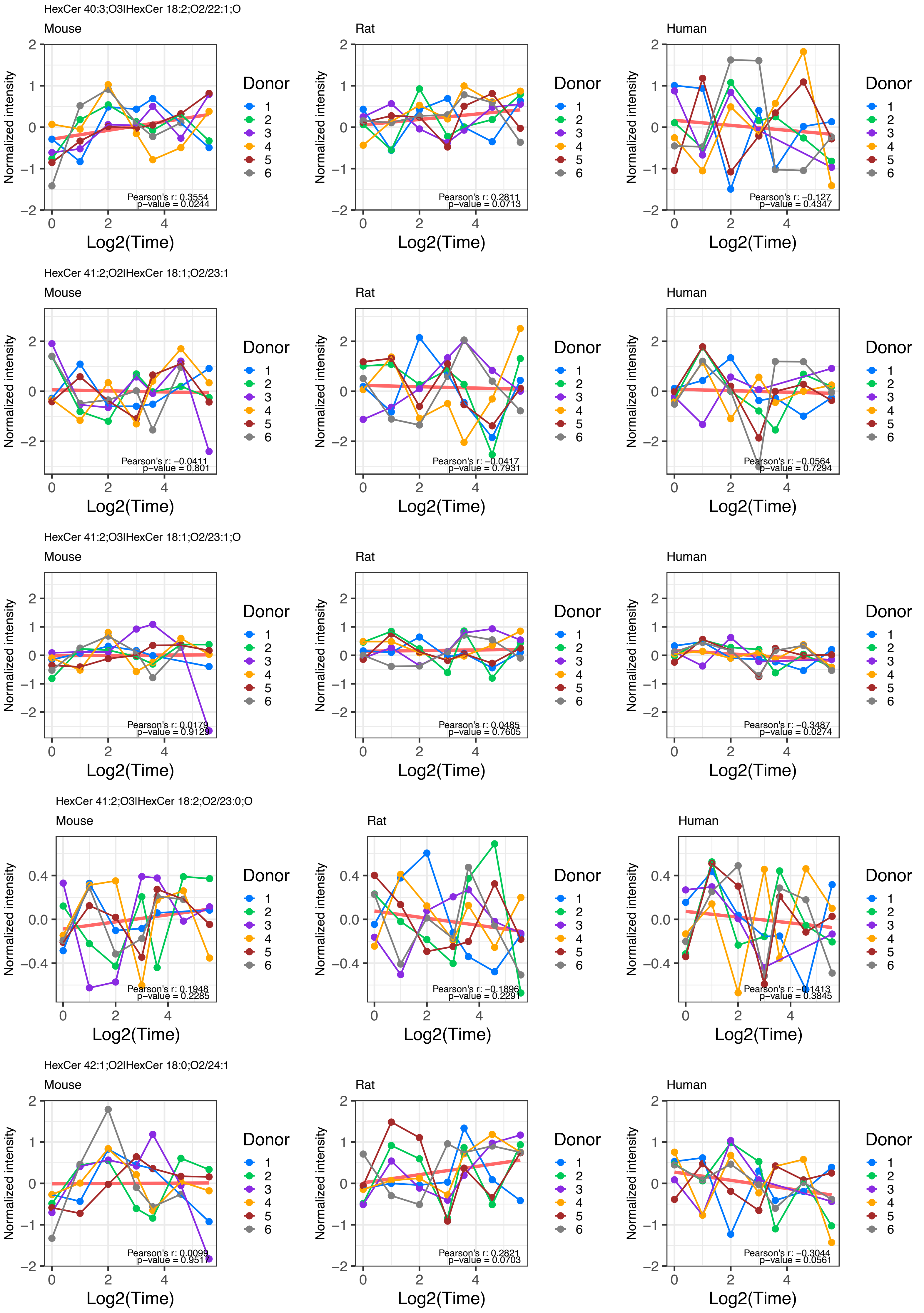

HexCer 40:3;O3|HexCer 18:2;O2/22:1;O
Mouse
Pearson's r: 0.3554
p−value = 0.0244
Rat
Pearson's r: 0.2811
p−value = 0.0713
Human
Pearson's r: −0.127
p−value = 0.4347
HexCer 41:2;O2|HexCer 18:1;O2/23:1
Mouse
Pearson's r: −0.0411
p−value = 0.801
Rat
Pearson's r: −0.0417
p−value = 0.7931
Human
Pearson's r: −0.0564
p−value = 0.7294
HexCer 41:2;O3|HexCer 18:1;O2/23:1;O
Mouse
Pearson's r: 0.0179
p−value = 0.9129
Rat
Pearson's r: 0.0485
p−value = 0.7605
Human
Pearson's r: −0.3487
p−value = 0.0274
HexCer 41:2;O3|HexCer 18:2;O2/23:0;O
Mouse
Pearson's r: 0.1948
p−value = 0.2285
Rat
Pearson's r: −0.1896
p−value = 0.2291
Human
Pearson's r: −0.1413
p−value = 0.3845
HexCer 42:1;O2|HexCer 18:0;O2/24:1
Mouse
Pearson's r: 0.0099
p−value = 0.9517
Rat
Pearson's r: 0.2821
p−value = 0.0703
Human
Pearson's r: −0.3044
p−value = 0.0561
Normalized intensity
Log2(Time)
Donor
1
2
3
4
5
6

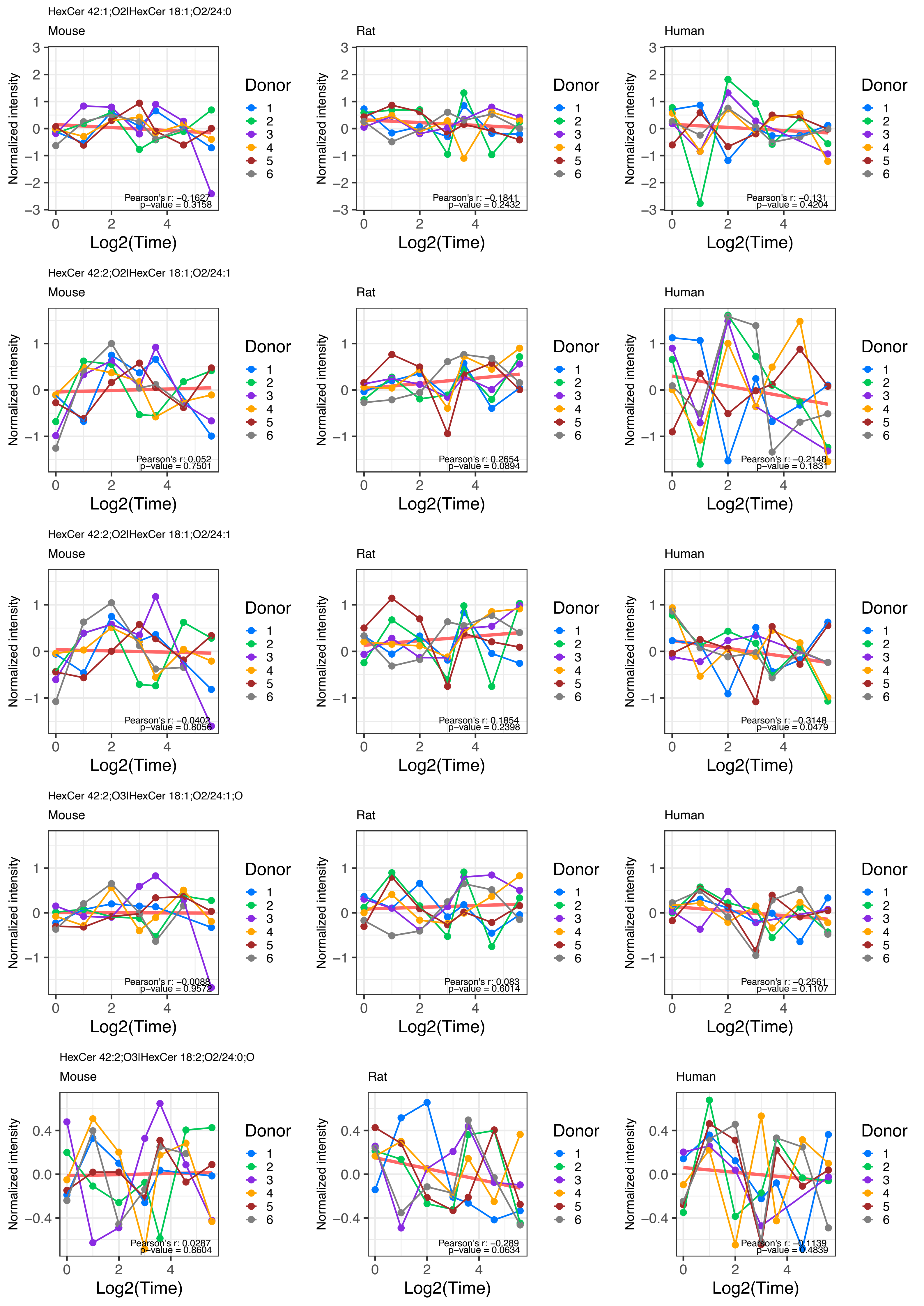

HexCer 42:1;O2|HexCer 18:1;O2/24:0
Mouse
Pearson's r: −0.1627
p−value = 0.3158
Rat
Pearson's r: −0.1841
p−value = 0.2432
Human
Pearson's r: −0.131
p−value = 0.4204
HexCer 42:2;O2|HexCer 18:1;O2/24:1
Mouse
Pearson's r: 0.052
p−value = 0.7501
Rat
Pearson's r: 0.2654
p−value = 0.0894
Human
Pearson's r: −0.2148
p−value = 0.1831
HexCer 42:2;O2|HexCer 18:1;O2/24:1
Mouse
Pearson's r: −0.0402
p−value = 0.8056
Rat
Pearson's r: 0.1854
p−value = 0.2398
Human
Pearson's r: −0.3148
p−value = 0.0479
HexCer 42:2;O3|HexCer 18:1;O2/24:1;O
Mouse
Pearson's r: −0.0088
p−value = 0.9572
Rat
Pearson's r: 0.083
p−value = 0.6014
Human
Pearson's r: −0.2561
p−value = 0.1107
HexCer 42:2;O3|HexCer 18:2;O2/24:0;O
Mouse
Pearson's r: 0.0287
p−value = 0.8604
Rat
Pearson's r: −0.289
p−value = 0.0634
Human
Pearson's r: −0.1139
p−value = 0.4839
Normalized intensity
Log2(Time)
Donor
1
2
3
4
5
6

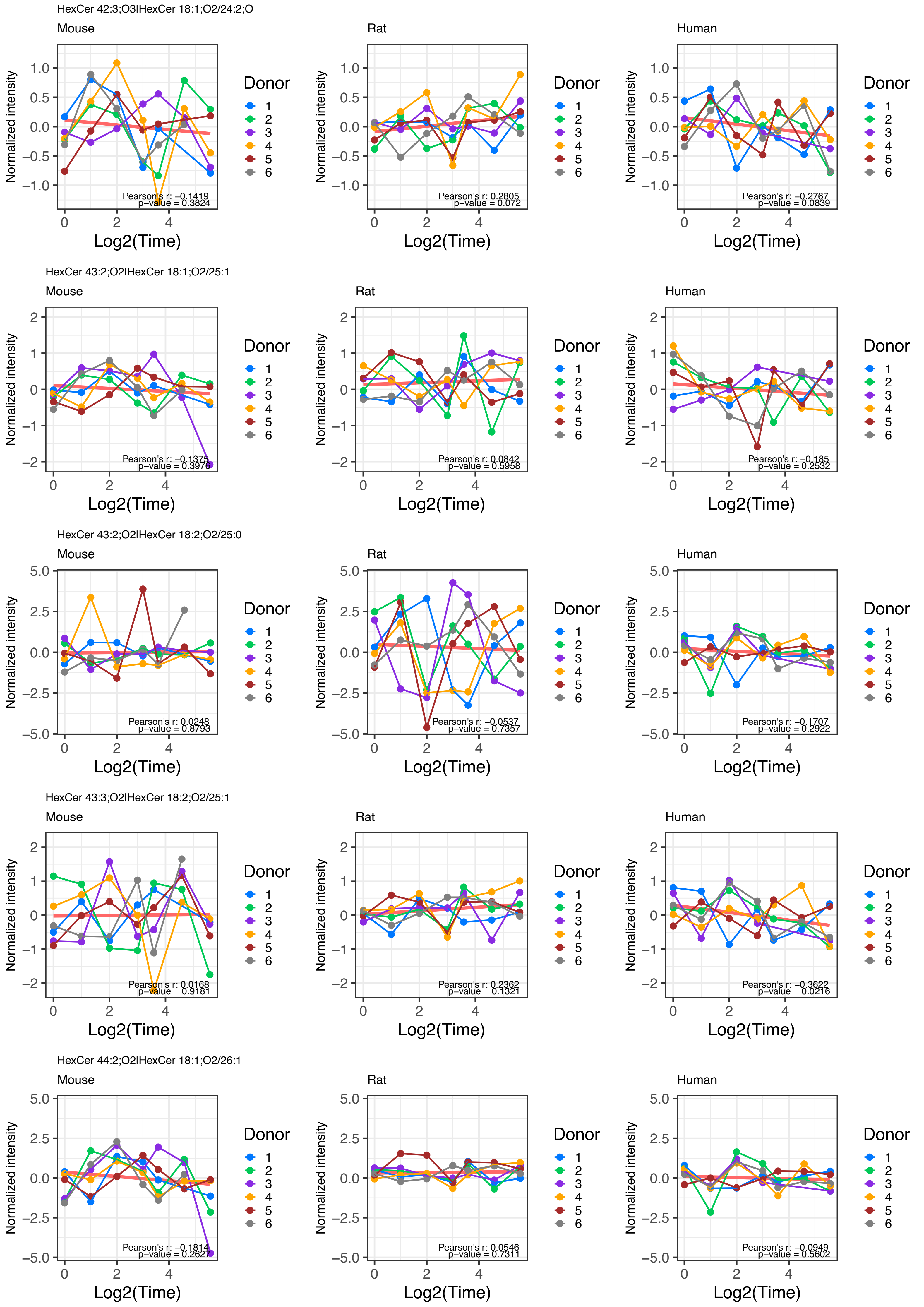
HexCer 42:3;O3|HexCer 18:1;O2/24:2;O
Mouse
Pearson's r: −0.1419
p−value = 0.3824
Rat
Pearson's r: 0.2805
p−value = 0.072
Human
Pearson's r: −0.2767
p−value = 0.0839
HexCer 43:2;O2|HexCer 18:1;O2/25:1
Mouse
Pearson's r: −0.1375
p−value = 0.3976
Rat
Pearson's r: 0.0842
p−value = 0.5958
Human
Pearson's r: −0.185
p−value = 0.2532
HexCer 43:2;O2|HexCer 18:2;O2/25:0
Mouse
Pearson's r: 0.0248
p−value = 0.8793
Rat
Pearson's r: −0.0537
p−value = 0.7357
Human
Pearson's r: −0.1707
p−value = 0.2922
HexCer 43:3;O2|HexCer 18:2;O2/25:1
Mouse
Pearson's r: 0.0168
p−value = 0.9181
Rat
Pearson's r: 0.2362
p−value = 0.1321
Human
Pearson's r: −0.3622
p−value = 0.0216
HexCer 44:2;O2|HexCer 18:1;O2/26:1
Mouse
Pearson's r: −0.1814
p−value = 0.2627
Rat
Pearson's r: 0.0546
p−value = 0.7311
Human
Pearson's r: −0.0949
p−value = 0.5602
Normalized intensity
Log2(Time)
Donor
1
2
3
4
5
6

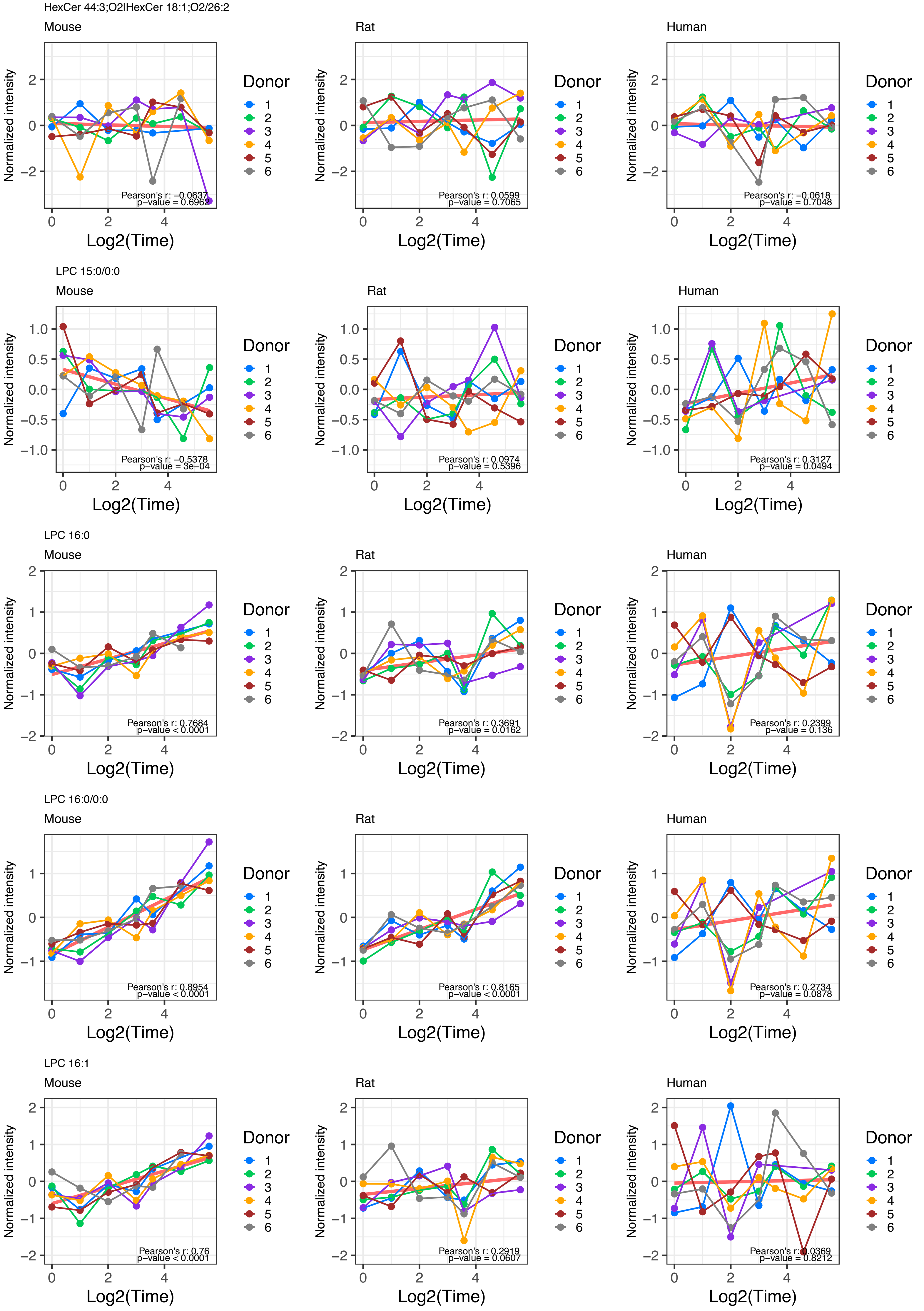

HexCer 44:3;O2|HexCer 18:1;O2/26:2
Mouse
Pearson's r: −0.0637
p−value = 0.6962
Rat
Pearson's r: 0.0599
p−value = 0.7065
Human
Pearson's r: −0.0618
p−value = 0.7048
LPC 15:0/0:0
Mouse
Pearson's r: −0.5378
p−value = 3e−04
Rat
Pearson's r: 0.0974
p−value = 0.5396
Human
Pearson's r: 0.3127
p−value = 0.0494
LPC 16:0
Mouse
Pearson's r: 0.7684
p−value < 0.0001
Rat
Pearson's r: 0.3691
p−value = 0.0162
Human
Pearson's r: 0.2399
p−value = 0.136
LPC 16:0/0:0
Mouse
Pearson's r: 0.8954
p−value < 0.0001
Rat
Pearson's r: 0.8165
p−value < 0.0001
Human
Pearson's r: 0.2734
p−value = 0.0878
LPC 16:1
Mouse
Pearson's r: 0.76
p−value < 0.0001
Rat
Pearson's r: 0.2919
p−value = 0.0607
Human
Pearson's r: 0.0369
p−value = 0.8212
Normalized intensity
Log2(Time)
Donor
1
2
3
4
5
6

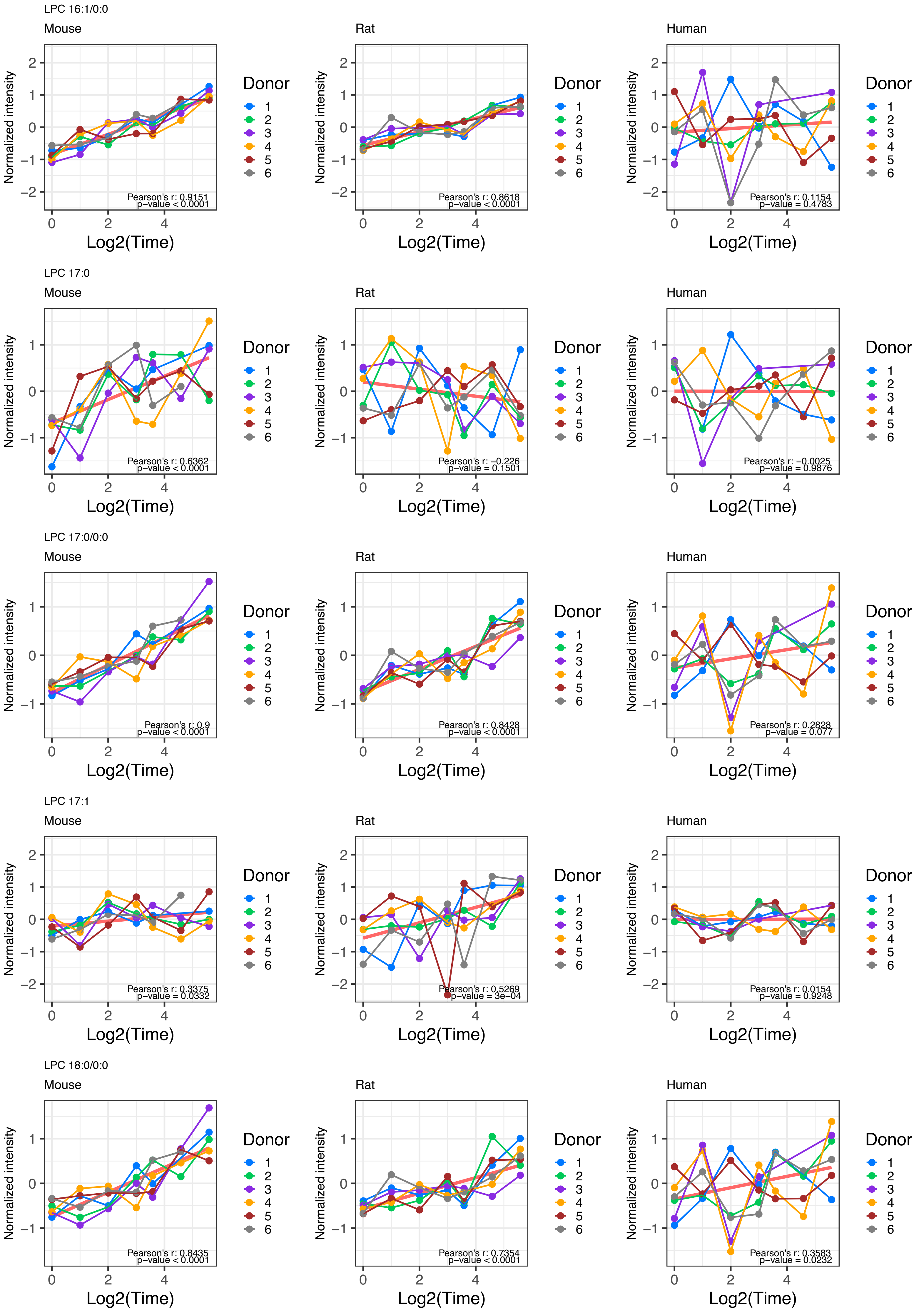
LPC 16:1/0:0
Mouse
Pearson's r: 0.9151
p−value < 0.0001
Rat
Pearson's r: 0.8618
p−value < 0.0001
Human
Pearson's r: 0.1154
p−value = 0.4783
LPC 17:0
Mouse
Pearson's r: 0.6362
p−value < 0.0001
Rat
Pearson's r: −0.226
p−value = 0.1501
Human
Pearson's r: −0.0025
p−value = 0.9876
LPC 17:0/0:0
Mouse
Pearson's r: 0.9
p−value < 0.0001
Rat
Pearson's r: 0.8428
p−value < 0.0001
Human
Pearson's r: 0.2828
p−value = 0.077
LPC 17:1
Mouse
Pearson's r: 0.3375
p−value = 0.0332
Rat
Pearson's r: 0.5269
p−value = 3e−04
Human
Pearson's r: 0.0154
p−value = 0.9248
LPC 18:0/0:0
Mouse
Pearson's r: 0.8435
p−value < 0.0001
Rat
Pearson's r: 0.7354
p−value < 0.0001
Human
Pearson's r: 0.3583
p−value = 0.0232
Normalized intensity
Log2(Time)
Donor
1
2
3
4
5
6

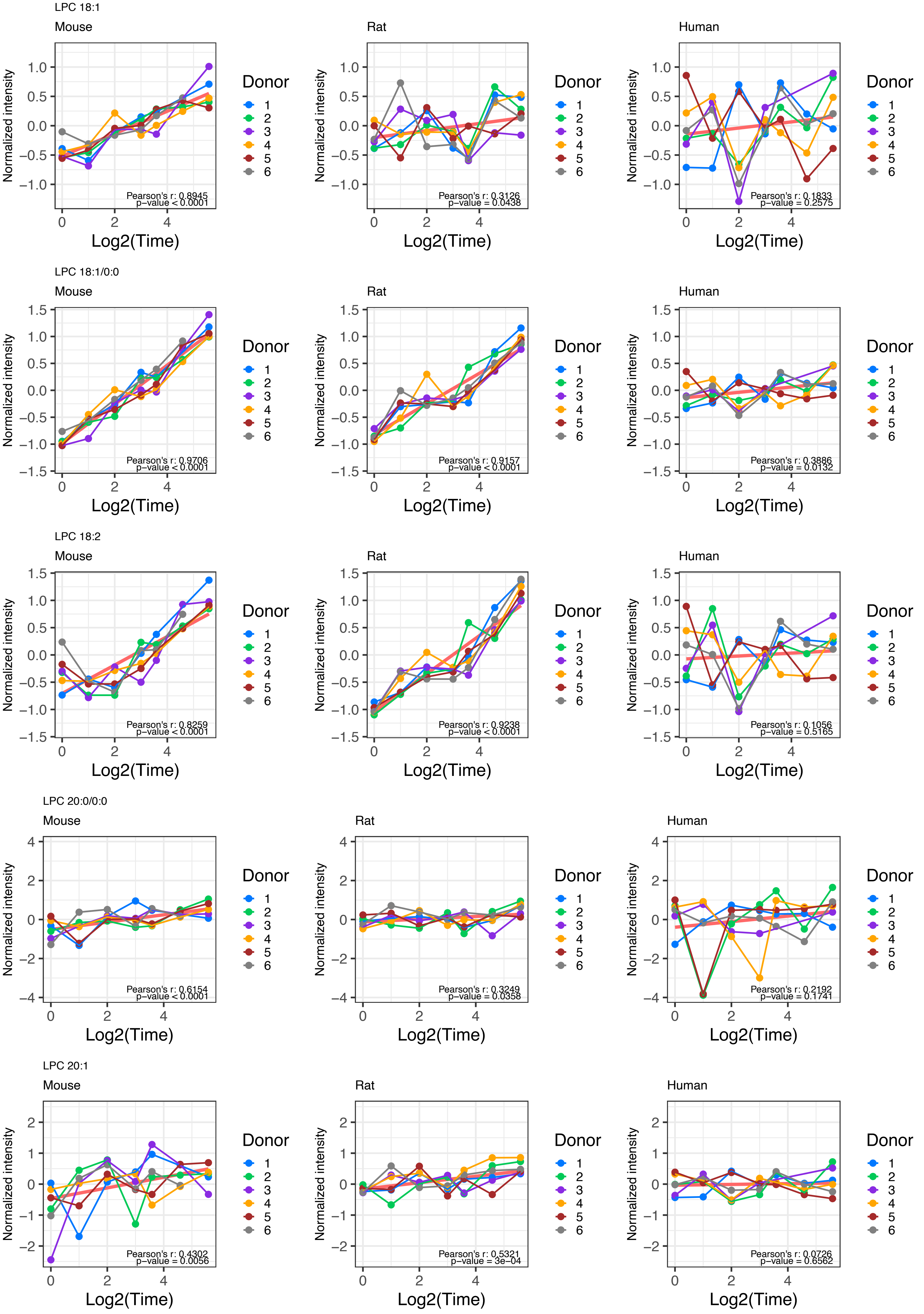

LPC 18:1
Mouse
Pearson's r: 0.8945
p−value < 0.0001
Rat
Pearson's r: 0.3126
p−value = 0.0438
Human
Pearson's r: 0.1833
p−value = 0.2575
LPC 18:1/0:0
Mouse
Pearson's r: 0.9706
p−value < 0.0001
Rat
Pearson's r: 0.9157
p−value < 0.0001
Human
Pearson's r: 0.3886
p−value = 0.0132
LPC 18:2
Mouse
Pearson's r: 0.8259
p−value < 0.0001
Rat
Pearson's r: 0.9238
p−value < 0.0001
Human
Pearson's r: 0.1056
p−value = 0.5165
LPC 20:0/0:0
Mouse
Pearson's r: 0.6154
p−value < 0.0001
Rat
Pearson's r: 0.3249
p−value = 0.0358
Human
Pearson's r: 0.2192
p−value = 0.1741
LPC 20:1
Mouse
Pearson's r: 0.4302
p−value = 0.0056
Rat
Pearson's r: 0.5321
p−value = 3e−04
Human
Pearson's r: 0.0726
p−value = 0.6562
Normalized intensity
Log2(Time)
Donor
1
2
3
4
5
6

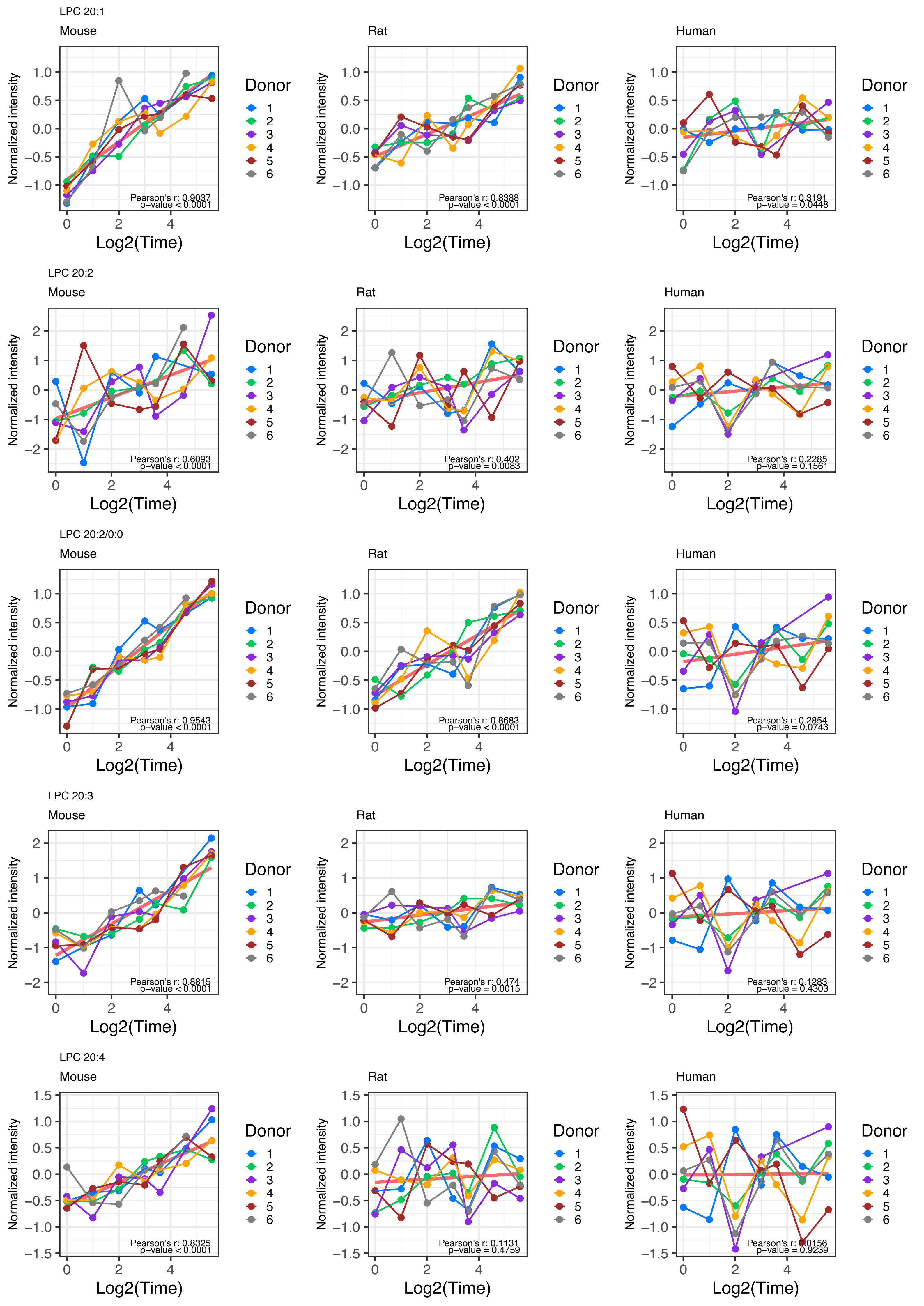
LPC 20:1
Mouse
Pearson's r: 0.9037
p−value < 0.0001
Rat
Pearson's r: 0.8388
p−value < 0.0001
Human
Pearson's r: 0.3191
p−value = 0.0448
LPC 20:2
Mouse
Pearson's r: 0.6093
p−value < 0.0001
Rat
Pearson's r: 0.402
p−value = 0.0083
Human
Pearson's r: 0.2285
p−value = 0.1561
LPC 20:2/0:0
Mouse
Pearson's r: 0.9543
p−value < 0.0001
Rat
Pearson's r: 0.8683
p−value < 0.0001
Human
Pearson's r: 0.2854
p−value = 0.0743
LPC 20:3
Mouse
Pearson's r: 0.8815
p−value < 0.0001
Rat
Pearson's r: 0.474
p−value = 0.0015
Human
Pearson's r: 0.1283
p−value = 0.4303
LPC 20:4
Mouse
Pearson's r: 0.8325
p−value < 0.0001
Rat
Pearson's r: 0.1131
p−value = 0.4759
Human
Pearson's r: 0.0156
p−value = 0.9239
Normalized intensity
Log2(Time)
Donor
1
2
3
4
5
6

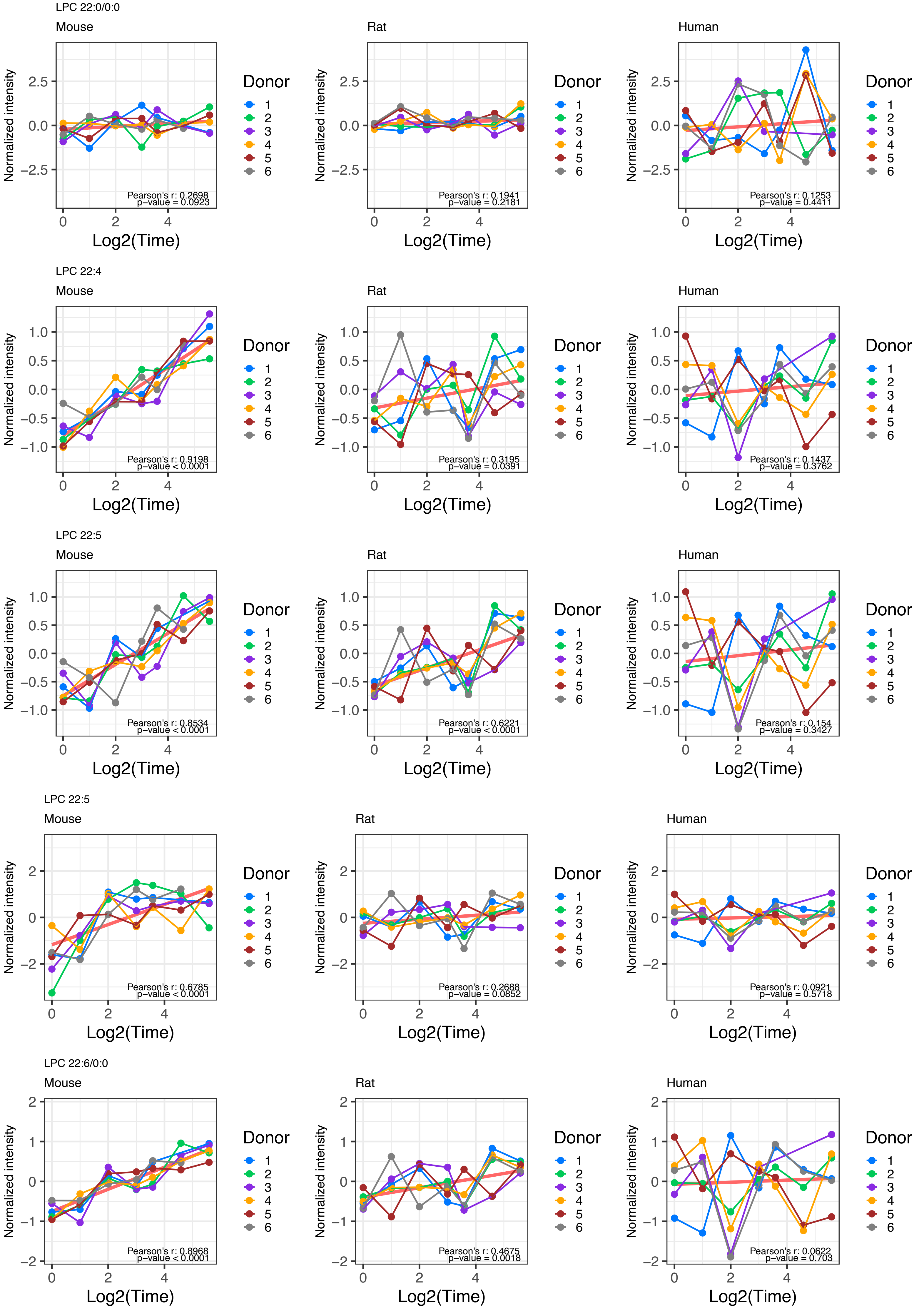
LPC 22:0/0:0
Mouse
Pearson's r: 0.2698
p−value = 0.0923
Rat
Pearson's r: 0.1941
p−value = 0.2181
Human
Pearson's r: 0.1253
p−value = 0.4411
LPC 22:4
Mouse
Pearson's r: 0.9198
p−value < 0.0001
Rat
Pearson's r: 0.3195
p−value = 0.0391
Human
Pearson's r: 0.1437
p−value = 0.3762
LPC 22:5
Mouse
Pearson's r: 0.8534
p−value < 0.0001
Rat
Pearson's r: 0.6221
p−value < 0.0001
Human
Pearson's r: 0.154
p−value = 0.3427
LPC 22:5
Mouse
Pearson's r: 0.6785
p−value < 0.0001
Rat
Pearson's r: 0.2688
p−value = 0.0852
Human
Pearson's r: 0.0921
p−value = 0.5718
LPC 22:6/0:0
Mouse
Pearson's r: 0.8968
p−value < 0.0001
Rat
Pearson's r: 0.4675
p−value = 0.0018
Human
Pearson's r: 0.0622
p−value = 0.703
Normalized intensity
Log2(Time)
Donor
1
2
3
4
5
6

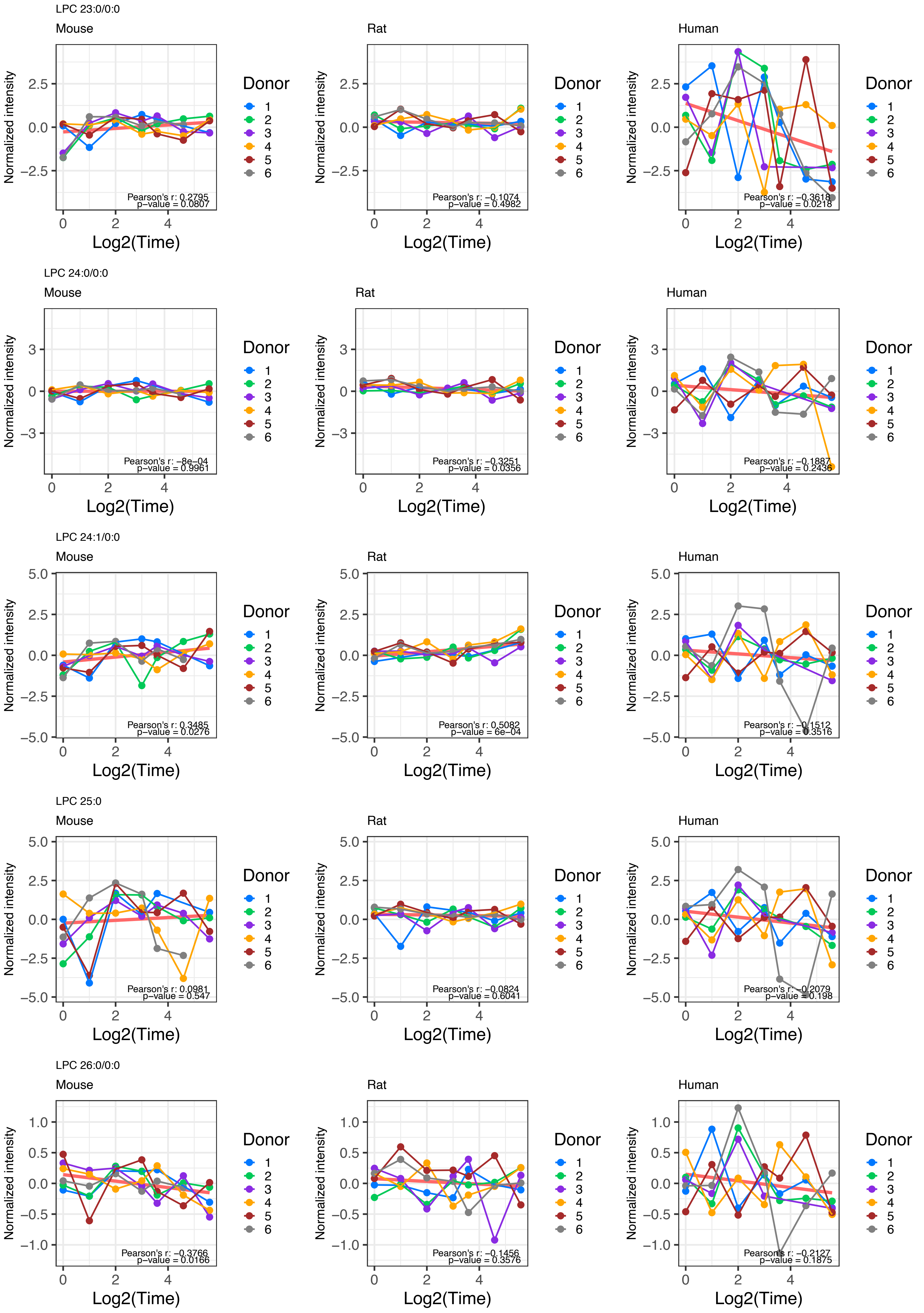
LPC 23:0/0:0
Mouse
Rat
Human
Normalized intensity
Log2(Time)
Donor
1
2
3
4
5
6
Pearson's r: 0.2795
p−value = 0.0807
Pearson's r: −0.1074
p−value = 0.4982
Pearson's r: −0.3618
p−value = 0.0218
LPC 24:0/0:0
Pearson's r: −8e−04
p−value = 0.9961
Pearson's r: −0.3251
p−value = 0.0356
Pearson's r: −0.1887
p−value = 0.2436
LPC 24:1/0:0
Pearson's r: 0.3485
p−value = 0.0276
Pearson's r: 0.5082
p−value = 6e−04
Pearson's r: −0.1512
p−value = 0.3516
LPC 25:0
Pearson's r: 0.0981
p−value = 0.547
Pearson's r: −0.0824
p−value = 0.6041
Pearson's r: −0.2079
p−value = 0.198
LPC 26:0/0:0
Pearson's r: −0.3766
p−value = 0.0166
Pearson's r: −0.1456
p−value = 0.3576
Pearson's r: −0.2127
p−value = 0.1875

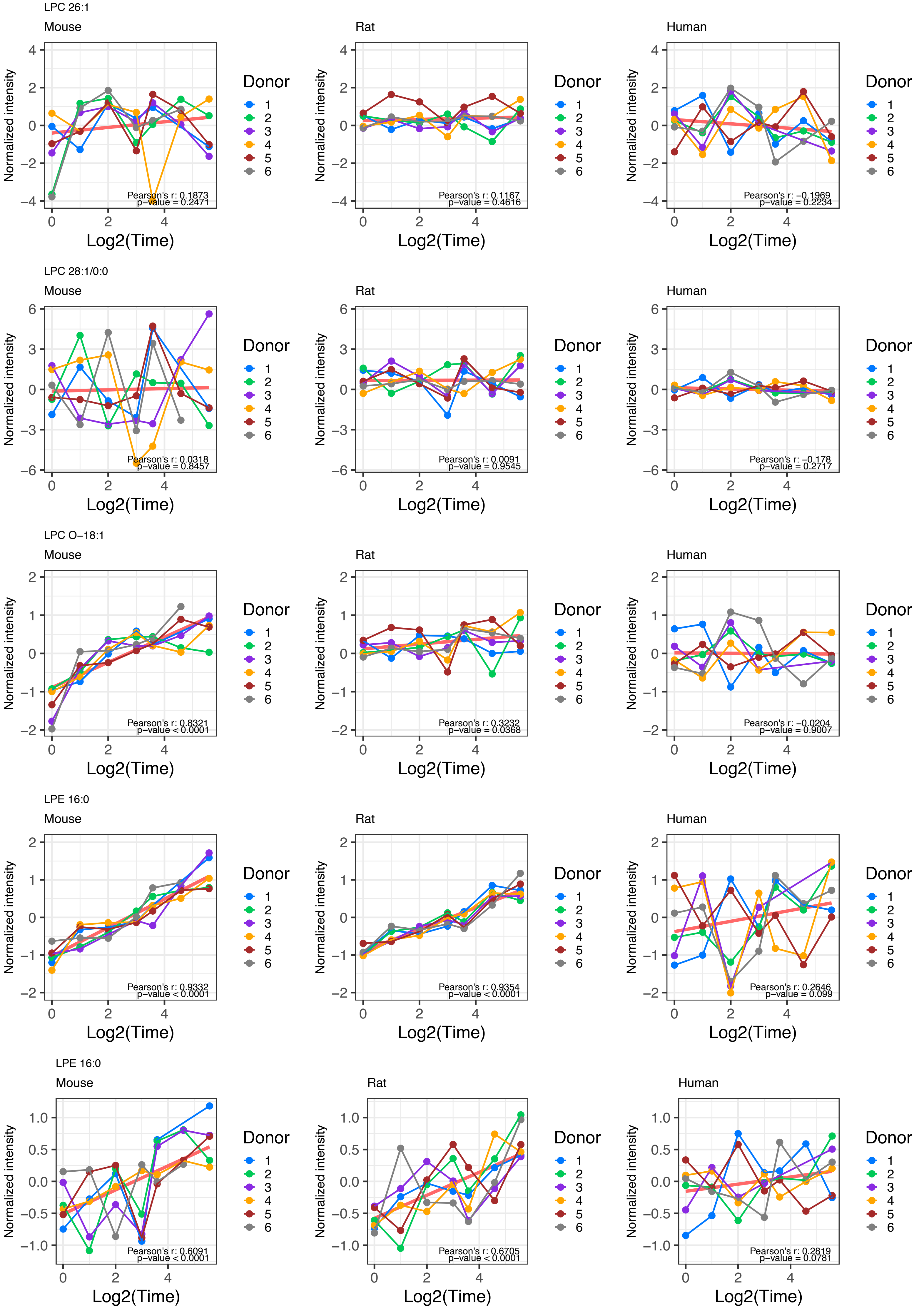

LPC 26:1
Mouse
Pearson's r: 0.1873
p−value = 0.2471
Rat
Pearson's r: 0.1167
p−value = 0.4616
Human
Pearson's r: −0.1969
p−value = 0.2234
LPC 28:1/0:0
Mouse
Pearson's r: 0.0318
p−value = 0.8457
Rat
Pearson's r: 0.0091
p−value = 0.9545
Human
Pearson's r: −0.178
p−value = 0.2717
LPC O−18:1
Mouse
Pearson's r: 0.8321
p−value < 0.0001
Rat
Pearson's r: 0.3232
p−value = 0.0368
Human
Pearson's r: −0.0204
p−value = 0.9007
LPE 16:0
Mouse
Pearson's r: 0.9332
p−value < 0.0001
Rat
Pearson's r: 0.9354
p−value < 0.0001
Human
Pearson's r: 0.2646
p−value = 0.099
LPE 16:0
Mouse
Pearson's r: 0.6091
p−value < 0.0001
Rat
Pearson's r: 0.6705
p−value < 0.0001
Human
Pearson's r: 0.2819
p−value = 0.0781
Normalized intensity
Log2(Time)
Donor
1
2
3
4
5
6

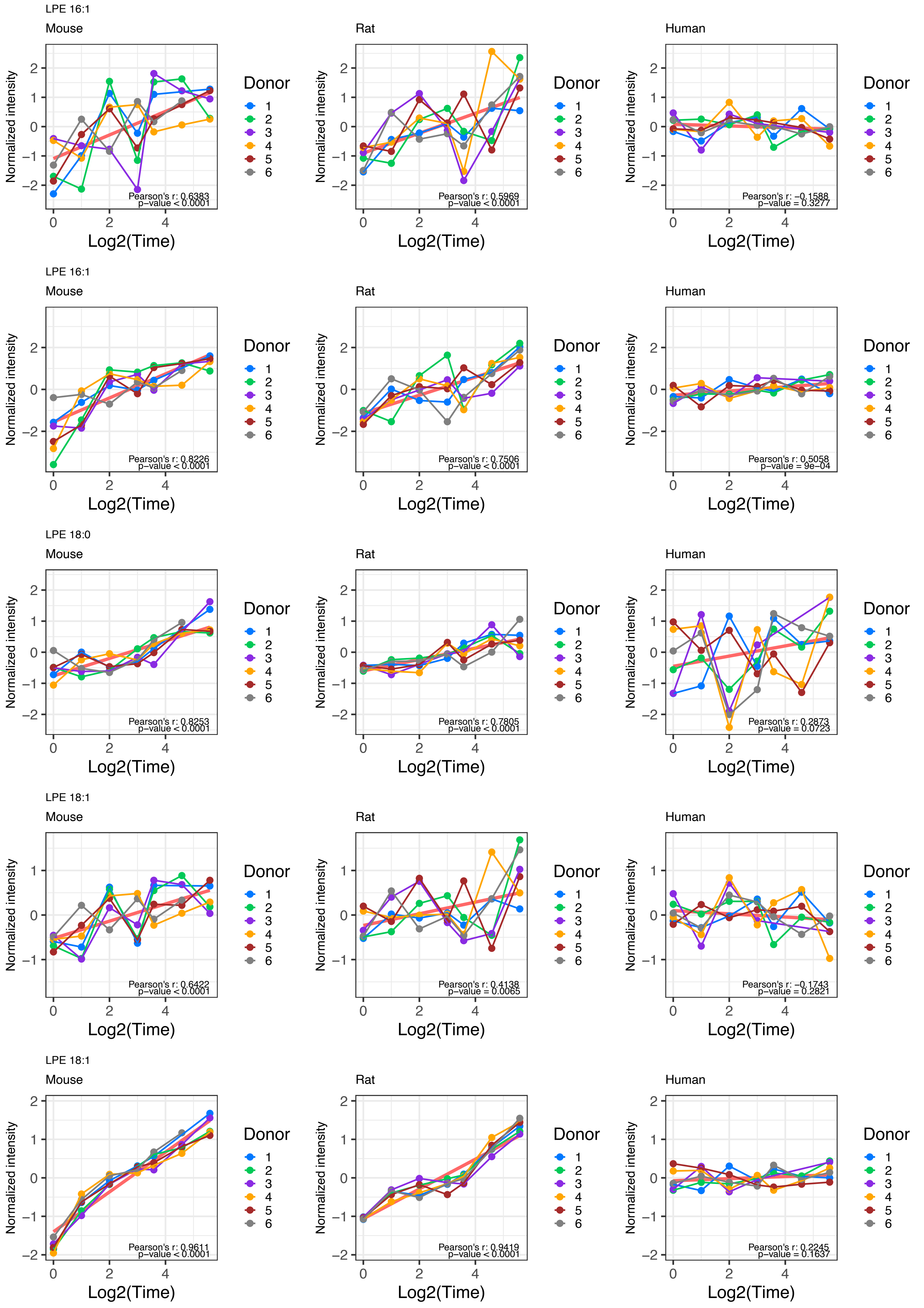

LPE 16:1
Mouse
Pearson's r: 0.6383
p−value < 0.0001
Rat
Pearson's r: 0.5969
p−value < 0.0001
Human
Pearson's r: −0.1588
p−value = 0.3277
LPE 16:1
Mouse
Pearson's r: 0.8226
p−value < 0.0001
Rat
Pearson's r: 0.7506
p−value < 0.0001
Human
Pearson's r: 0.5058
p−value = 9e−04
LPE 18:0
Mouse
Pearson's r: 0.8253
p−value < 0.0001
Rat
Pearson's r: 0.7805
p−value < 0.0001
Human
Pearson's r: 0.2873
p−value = 0.0723
LPE 18:1
Mouse
Pearson's r: 0.6422
p−value < 0.0001
Rat
Pearson's r: 0.4138
p−value = 0.0065
Human
Pearson's r: −0.1743
p−value = 0.2821
LPE 18:1
Mouse
Pearson's r: 0.9611
p−value < 0.0001
Rat
Pearson's r: 0.9419
p−value < 0.0001
Human
Pearson's r: 0.2245
p−value = 0.1637
Normalized intensity
Log2(Time)
Donor
1
2
3
4
5
6

LPE 18:2

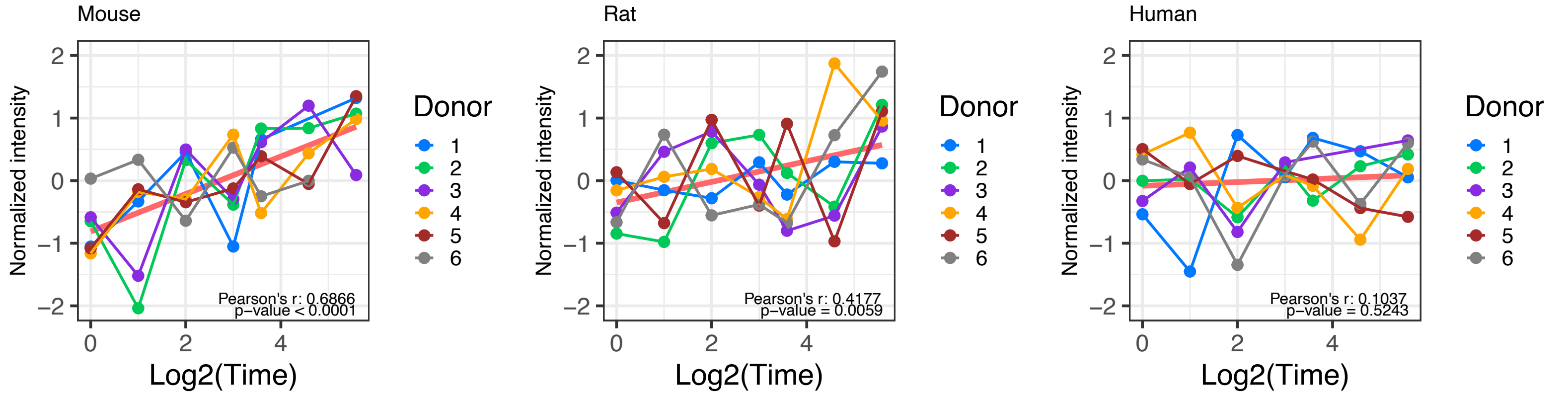

LPE 20:1

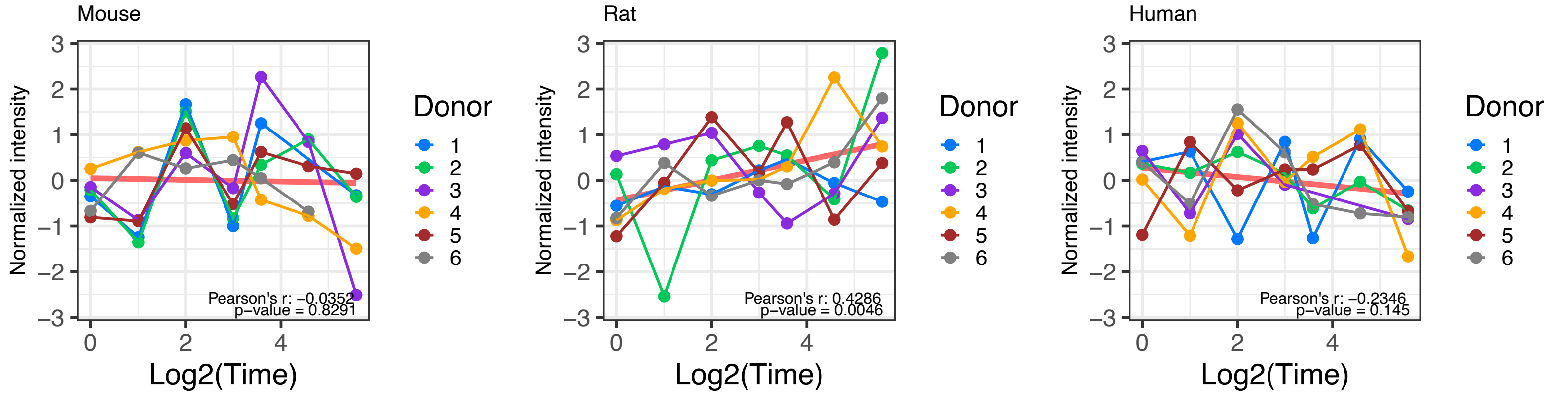

LPE 20:2

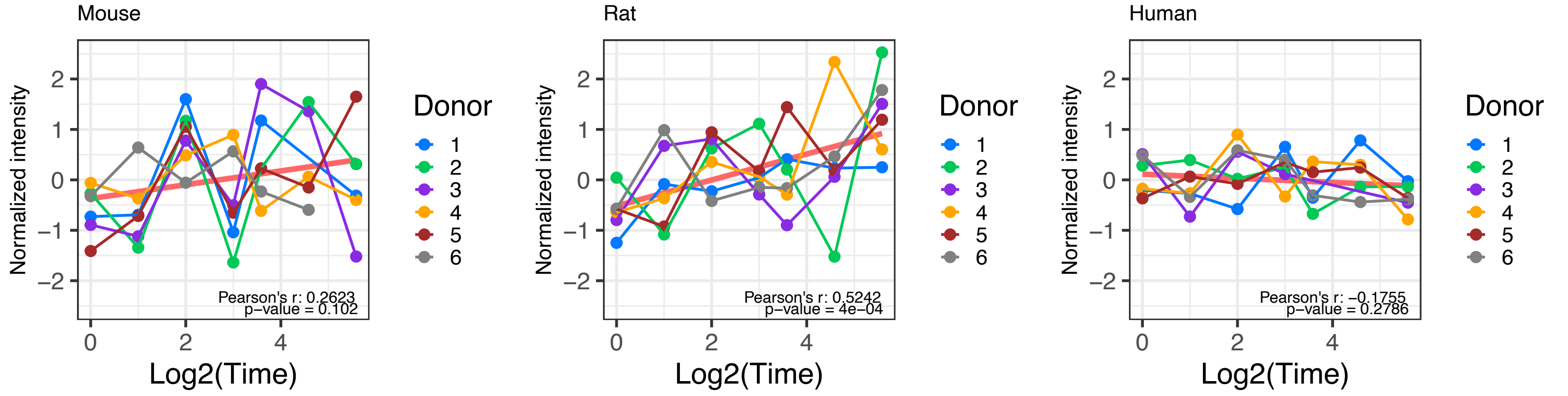

LPE 20:2

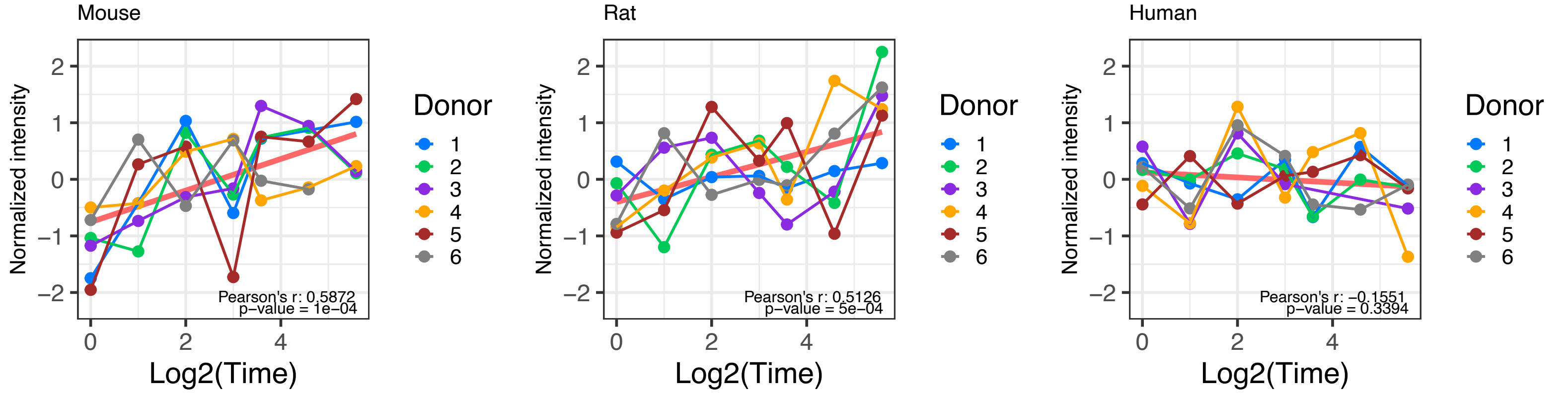

LPE 20:3

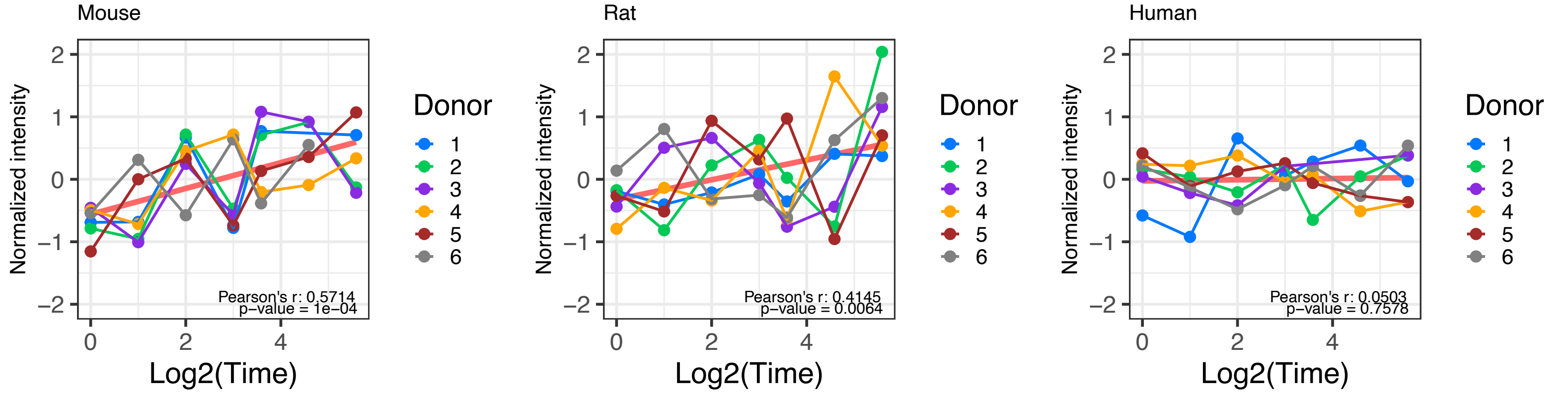

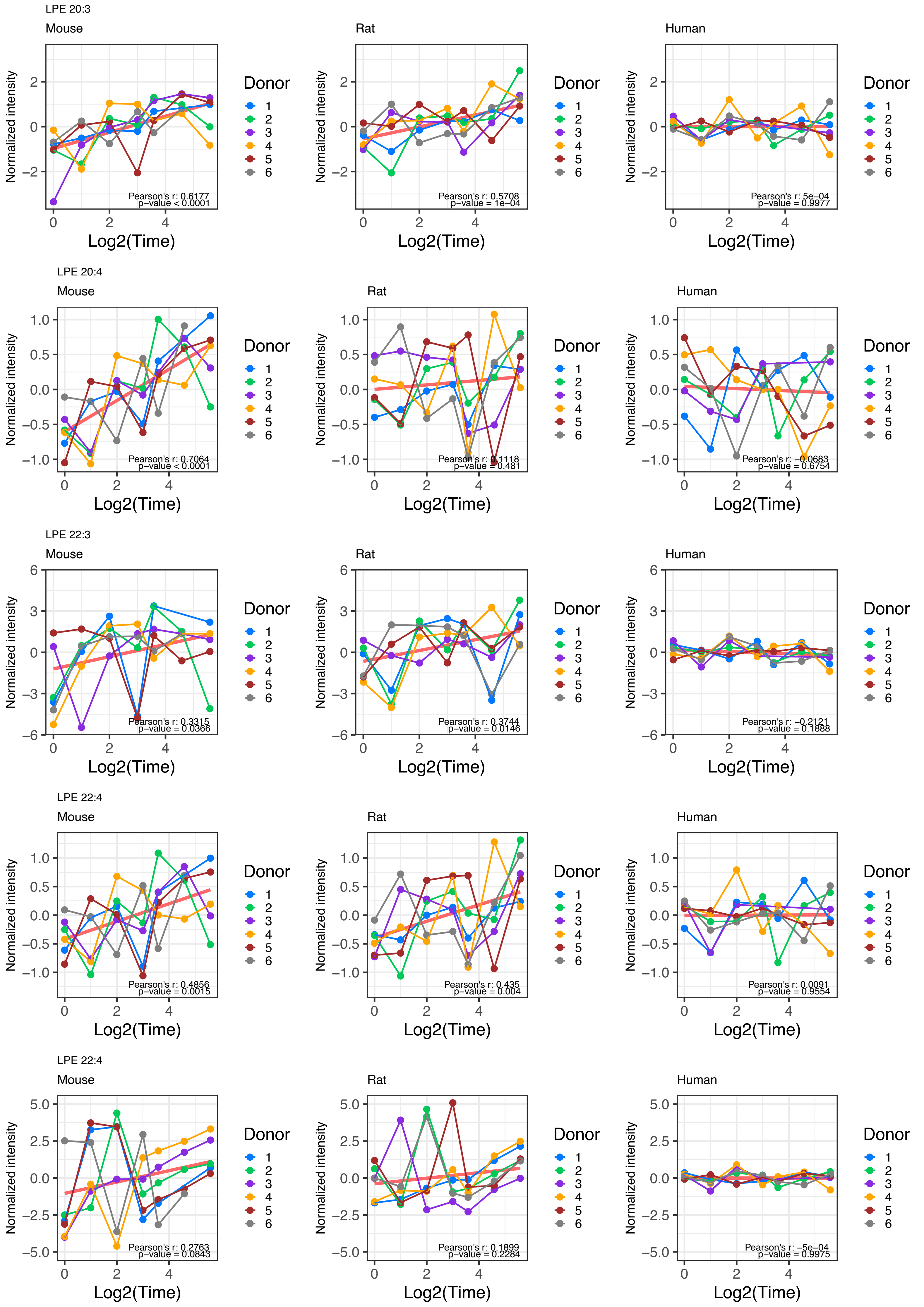
LPE 20:3
Mouse
Rat
Human
Normalized intensity
Log2(Time)
Donor
1
2
3
4
5
6
Pearson's r: 0.6177
p−value < 0.0001
Pearson's r: 0.5708
p−value = 1e−04
Pearson's r: 5e−04
p−value = 0.9977
LPE 20:4
Mouse
Rat
Human
Pearson's r: 0.7064
p−value < 0.0001
Pearson's r: 0.1118
p−value = 0.481
Pearson's r: −0.0683
p−value = 0.6754
LPE 22:3
Mouse
Rat
Human
Pearson's r: 0.3315
p−value = 0.0366
Pearson's r: 0.3744
p−value = 0.0146
Pearson's r: −0.2121
p−value = 0.1888
LPE 22:4
Mouse
Rat
Human
Pearson's r: 0.4856
p−value = 0.0015
Pearson's r: 0.435
p−value = 0.004
Pearson's r: 0.0091
p−value = 0.9554
LPE 22:4
Mouse
Rat
Human
Pearson's r: 0.2763
p−value = 0.0843
Pearson's r: 0.1899
p−value = 0.2284
Pearson's r: −5e−04
p−value = 0.9975

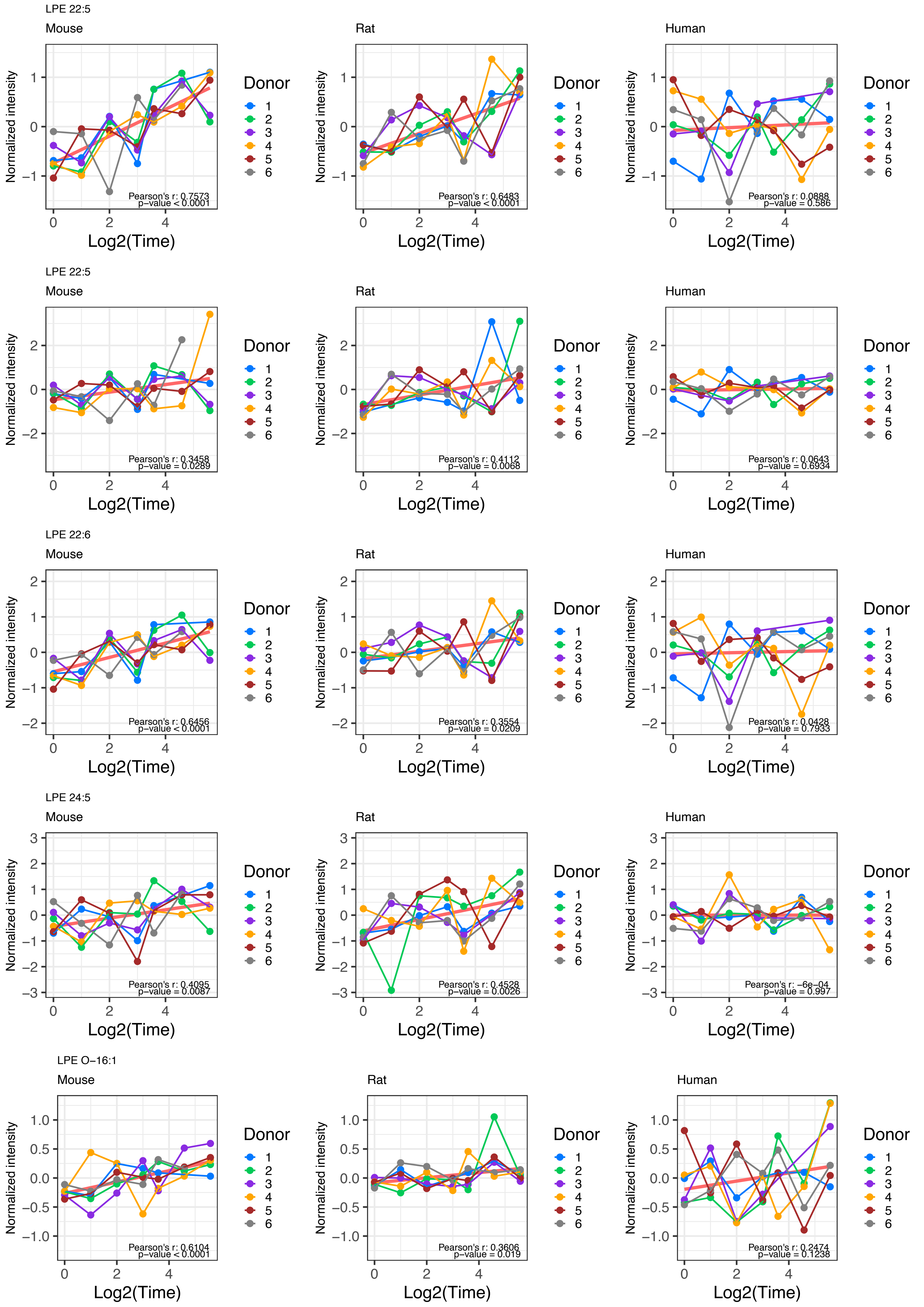
LPE 22:5
Mouse
Pearson's r: 0.7573
p−value < 0.0001
Rat
Pearson's r: 0.6483
p−value < 0.0001
Human
Pearson's r: 0.0888
p−value = 0.586
LPE 22:5
Mouse
Pearson's r: 0.3458
p−value = 0.0289
Rat
Pearson's r: 0.4112
p−value = 0.0068
Human
Pearson's r: 0.0643
p−value = 0.6934
LPE 22:6
Mouse
Pearson's r: 0.6456
p−value < 0.0001
Rat
Pearson's r: 0.3554
p−value = 0.0209
Human
Pearson's r: 0.0428
p−value = 0.7933
LPE 24:5
Mouse
Pearson's r: 0.4095
p−value = 0.0087
Rat
Pearson's r: 0.4528
p−value = 0.0026
Human
Pearson's r: −6e−04
p−value = 0.997
LPE O−16:1
Mouse
Pearson's r: 0.6104
p−value < 0.0001
Rat
Pearson's r: 0.3606
p−value = 0.019
Human
Pearson's r: 0.2474
p−value = 0.1238
Normalized intensity
Log2(Time)
Donor
1
2
3
4
5
6

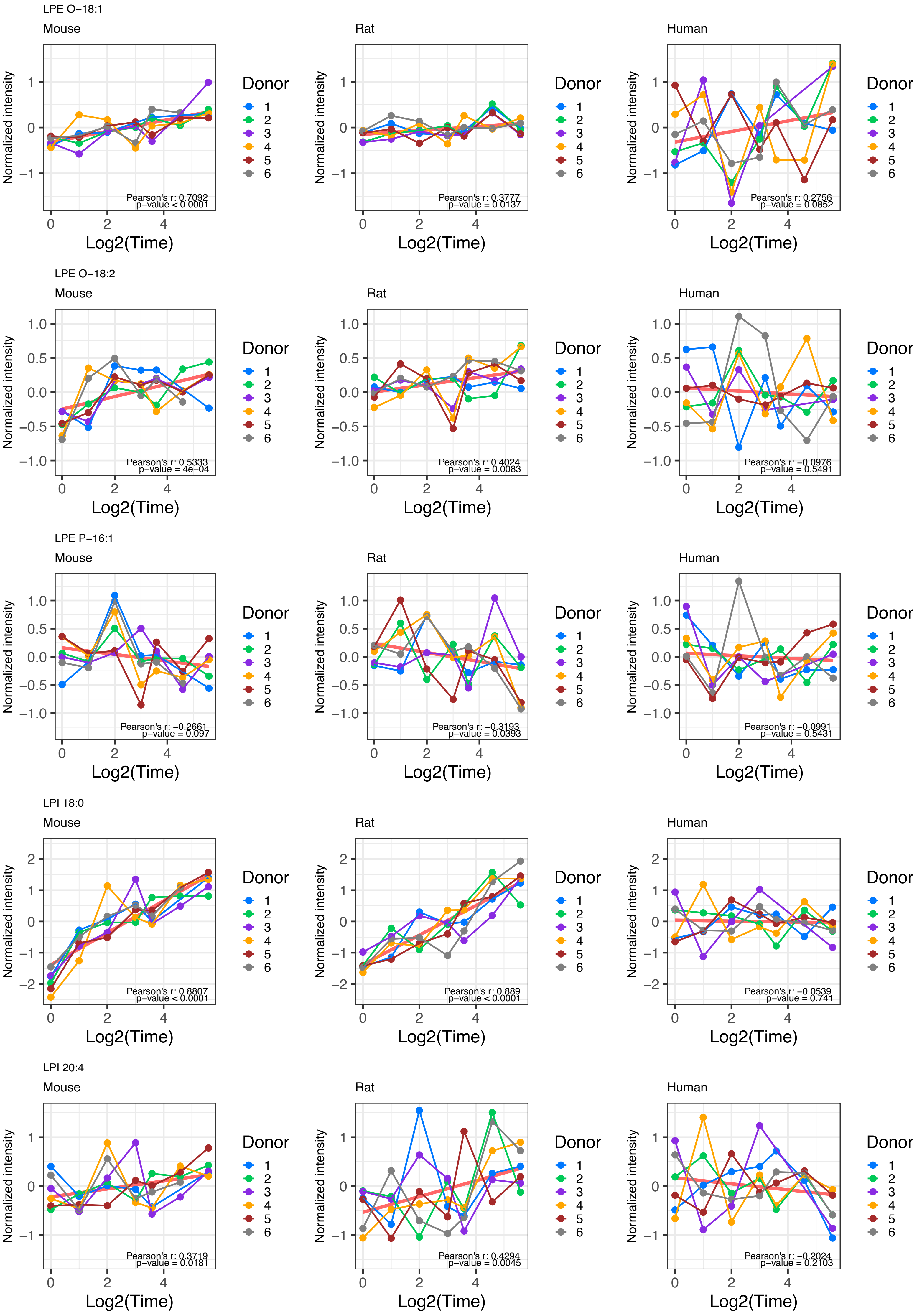
LPE O−18:1
Mouse
Pearson's r: 0.7092
p−value < 0.0001
Rat
Pearson's r: 0.3777
p−value = 0.0137
Human
Pearson's r: 0.2756
p−value = 0.0852
LPE O−18:2
Mouse
Pearson's r: 0.5333
p−value = 4e−04
Rat
Pearson's r: 0.4024
p−value = 0.0083
Human
Pearson's r: −0.0976
p−value = 0.5491
LPE P−16:1
Mouse
Pearson's r: −0.2661
p−value = 0.097
Rat
Pearson's r: −0.3193
p−value = 0.0393
Human
Pearson's r: −0.0991
p−value = 0.5431
LPI 18:0
Mouse
Pearson's r: 0.8807
p−value < 0.0001
Rat
Pearson's r: 0.889
p−value < 0.0001
Human
Pearson's r: −0.0539
p−value = 0.741
LPI 20:4
Mouse
Pearson's r: 0.3719
p−value = 0.0181
Rat
Pearson's r: 0.4294
p−value = 0.0045
Human
Pearson's r: −0.2024
p−value = 0.2103
Normalized intensity
Log2(Time)
Donor
1
2
3
4
5
6

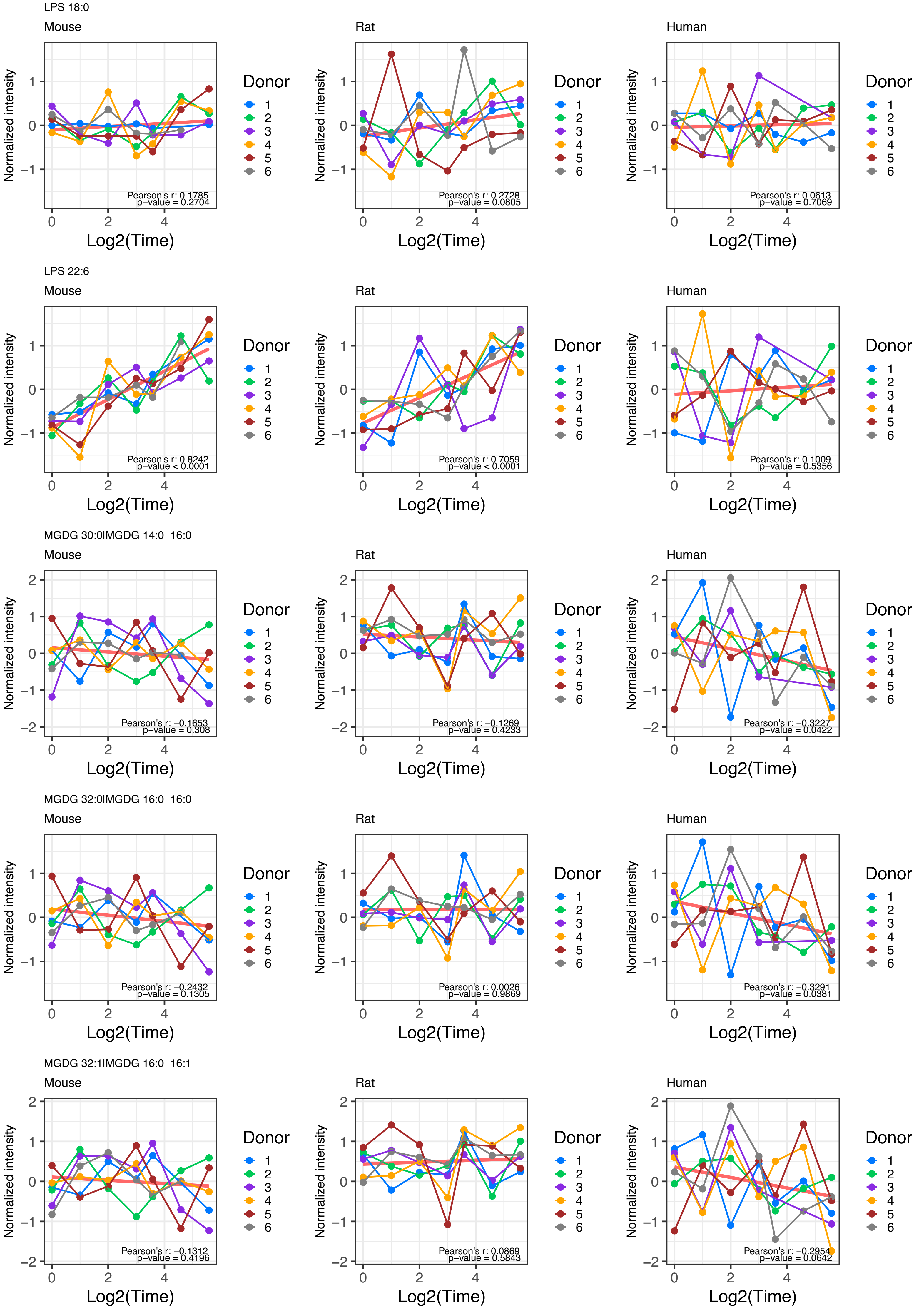
LPS 18:0
Mouse
Rat
Human
Normalized intensity
Log2(Time)
Donor
1
2
3
4
5
6
Pearson's r: 0.1785
p−value = 0.2704
Pearson's r: 0.2728
p−value = 0.0805
Pearson's r: 0.0613
p−value = 0.7069
LPS 22:6
Pearson's r: 0.8242
p−value < 0.0001
Pearson's r: 0.7059
p−value < 0.0001
Pearson's r: 0.1009
p−value = 0.5356
MGDG 30:0|MGDG 14:0_16:0
Pearson's r: −0.1653
p−value = 0.308
Pearson's r: −0.1269
p−value = 0.4233
Pearson's r: −0.3227
p−value = 0.0422
MGDG 32:0|MGDG 16:0_16:0
Pearson's r: −0.2432
p−value = 0.1305
Pearson's r: 0.0026
p−value = 0.9869
Pearson's r: −0.3291
p−value = 0.0381
MGDG 32:1|MGDG 16:0_16:1
Pearson's r: −0.1312
p−value = 0.4196
Pearson's r: 0.0869
p−value = 0.5843
Pearson's r: −0.2954
p−value = 0.0642

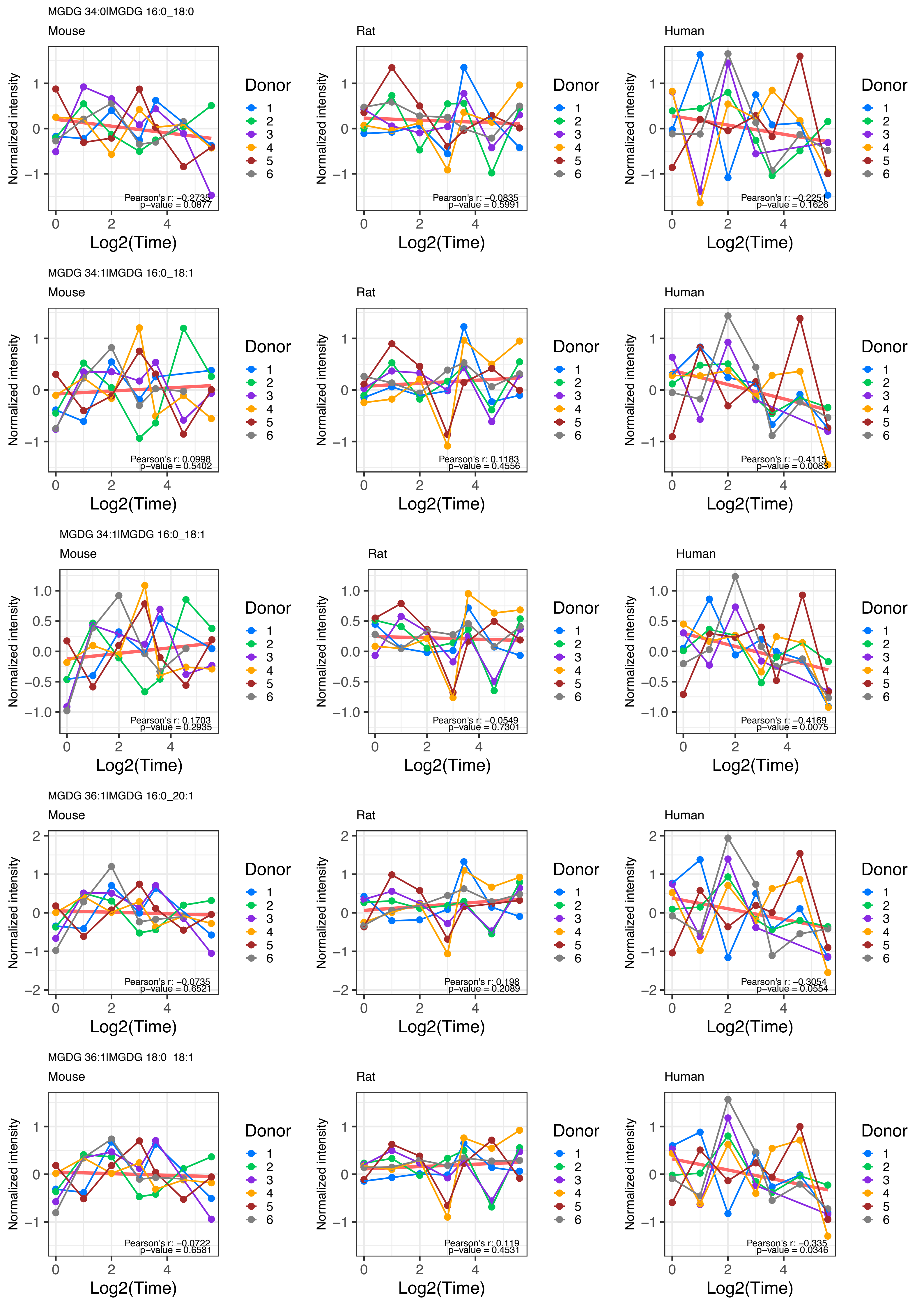
MGDG 34:0|MGDG 16:0_18:0
Mouse
Rat
Human
Normalized intensity
Log2(Time)
Donor
1
2
3
4
5
6
Pearson's r: −0.2735
p−value = 0.0877
Pearson's r: −0.0835
p−value = 0.5991
Pearson's r: −0.2251
p−value = 0.1626
MGDG 34:1|MGDG 16:0_18:1
Pearson's r: 0.0998
p−value = 0.5402
Pearson's r: 0.1183
p−value = 0.4556
Pearson's r: −0.4115
p−value = 0.0083
MGDG 34:1|MGDG 16:0_18:1
Pearson's r: 0.1703
p−value = 0.2935
Pearson's r: −0.0549
p−value = 0.7301
Pearson's r: −0.4169
p−value = 0.0075
MGDG 36:1|MGDG 16:0_20:1
Pearson's r: −0.0735
p−value = 0.6521
Pearson's r: 0.198
p−value = 0.2089
Pearson's r: −0.3054
p−value = 0.0554
MGDG 36:1|MGDG 18:0_18:1
Pearson's r: −0.0722
p−value = 0.6581
Pearson's r: 0.119
p−value = 0.4531
Pearson's r: −0.335
p−value = 0.0346

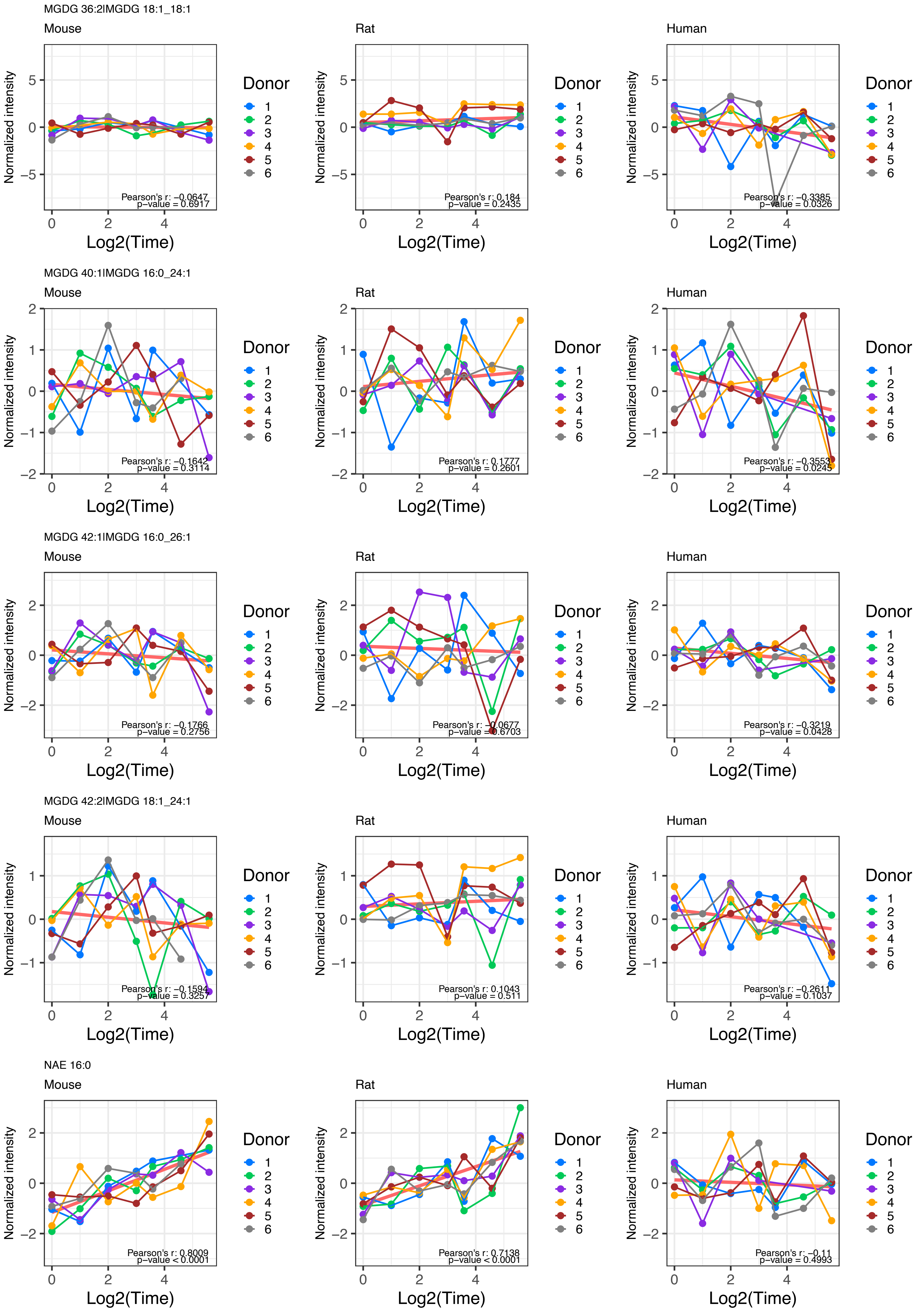

MGDG 36:2|MGDG 18:1_18:1
Mouse
Pearson's r: −0.0647
p−value = 0.6917
Rat
Pearson's r: 0.184
p−value = 0.2435
Human
Pearson's r: −0.3385
p−value = 0.0326
MGDG 40:1|MGDG 16:0_24:1
Mouse
Pearson's r: −0.1642
p−value = 0.3114
Rat
Pearson's r: 0.1777
p−value = 0.2601
Human
Pearson's r: −0.3553
p−value = 0.0245
MGDG 42:1|MGDG 16:0_26:1
Mouse
Pearson's r: −0.1766
p−value = 0.2756
Rat
Pearson's r: −0.0677
p−value = 0.6703
Human
Pearson's r: −0.3219
p−value = 0.0428
MGDG 42:2|MGDG 18:1_24:1
Mouse
Pearson's r: −0.1594
p−value = 0.3257
Rat
Pearson's r: 0.1043
p−value = 0.511
Human
Pearson's r: −0.2611
p−value = 0.1037
NAE 16:0
Mouse
Pearson's r: 0.8009
p−value < 0.0001
Rat
Pearson's r: 0.7138
p−value < 0.0001
Human
Pearson's r: −0.11
p−value = 0.4993
Normalized intensity
Log2(Time)
Donor
1
2
3
4
5
6

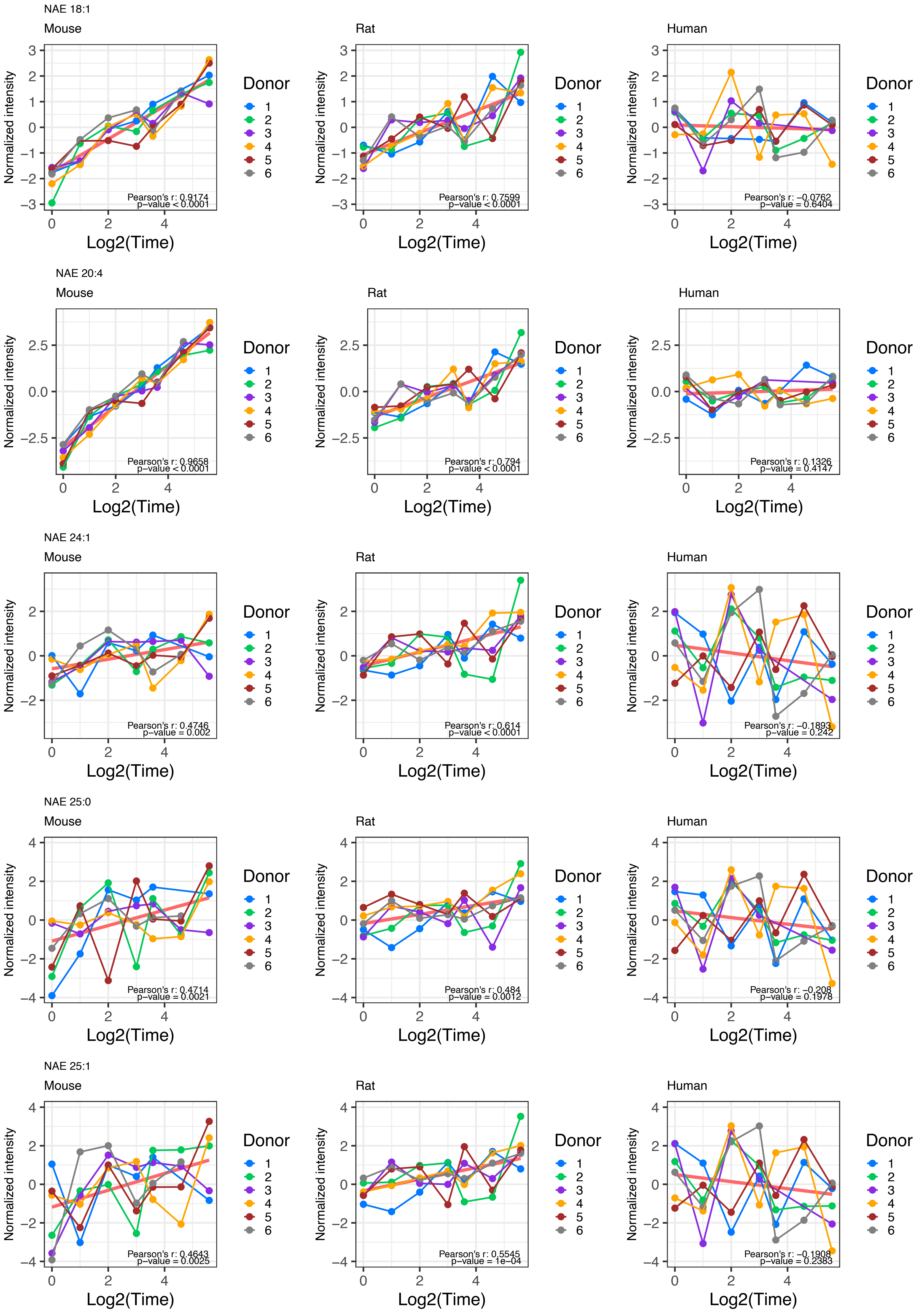
NAE 18:1
Mouse
Pearson's r: 0.9174
p−value < 0.0001
Rat
Pearson's r: 0.7599
p−value < 0.0001
Human
Pearson's r: −0.0762
p−value = 0.6404
NAE 20:4
Mouse
Pearson's r: 0.9658
p−value < 0.0001
Rat
Pearson's r: 0.794
p−value < 0.0001
Human
Pearson's r: 0.1326
p−value = 0.4147
NAE 24:1
Mouse
Pearson's r: 0.4746
p−value = 0.002
Rat
Pearson's r: 0.614
p−value < 0.0001
Human
Pearson's r: −0.1893
p−value = 0.242
NAE 25:0
Mouse
Pearson's r: 0.4714
p−value = 0.0021
Rat
Pearson's r: 0.484
p−value = 0.0012
Human
Pearson's r: −0.208
p−value = 0.1978
NAE 25:1
Mouse
Pearson's r: 0.4643
p−value = 0.0025
Rat
Pearson's r: 0.5545
p−value = 1e−04
Human
Pearson's r: −0.1908
p−value = 0.2383
Normalized intensity
Log2(Time)
Donor
1
2
3
4
5
6

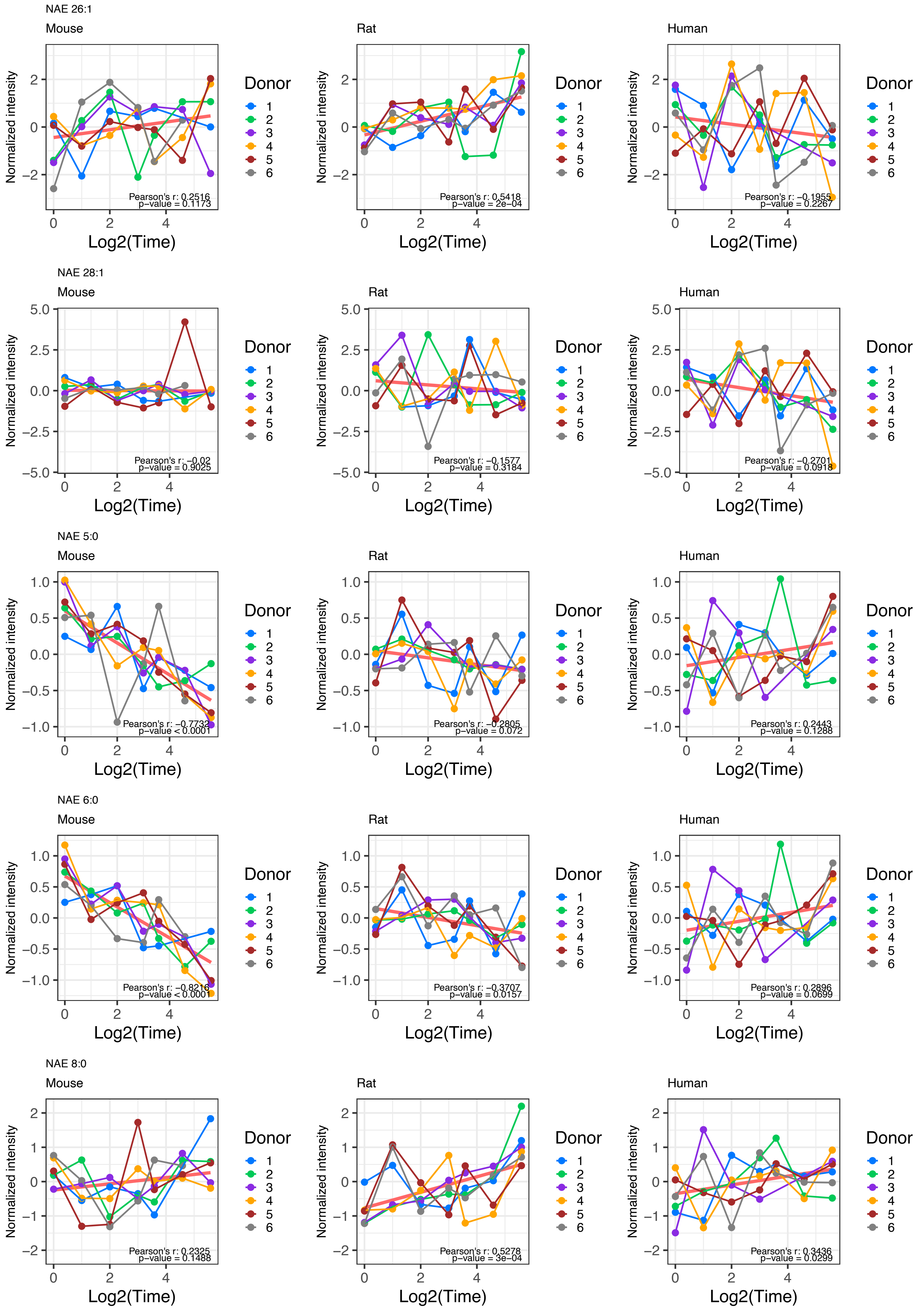
NAE 26:1
Mouse
Pearson's r: 0.2516
p−value = 0.1173
Rat
Pearson's r: 0.5418
p−value = 2e−04
Human
Pearson's r: −0.1955
p−value = 0.2267
NAE 28:1
Mouse
Pearson's r: −0.02
p−value = 0.9025
Rat
Pearson's r: −0.1577
p−value = 0.3184
Human
Pearson's r: −0.2701
p−value = 0.0918
NAE 5:0
Mouse
Pearson's r: −0.7732
p−value < 0.0001
Rat
Pearson's r: −0.2805
p−value = 0.072
Human
Pearson's r: 0.2443
p−value = 0.1288
NAE 6:0
Mouse
Pearson's r: −0.8216
p−value < 0.0001
Rat
Pearson's r: −0.3707
p−value = 0.0157
Human
Pearson's r: 0.2896
p−value = 0.0699
NAE 8:0
Mouse
Pearson's r: 0.2325
p−value = 0.1488
Rat
Pearson's r: 0.5278
p−value = 3e−04
Human
Pearson's r: 0.3436
p−value = 0.0299
Normalized intensity
Log2(Time)
Donor
1
2
3
4
5
6

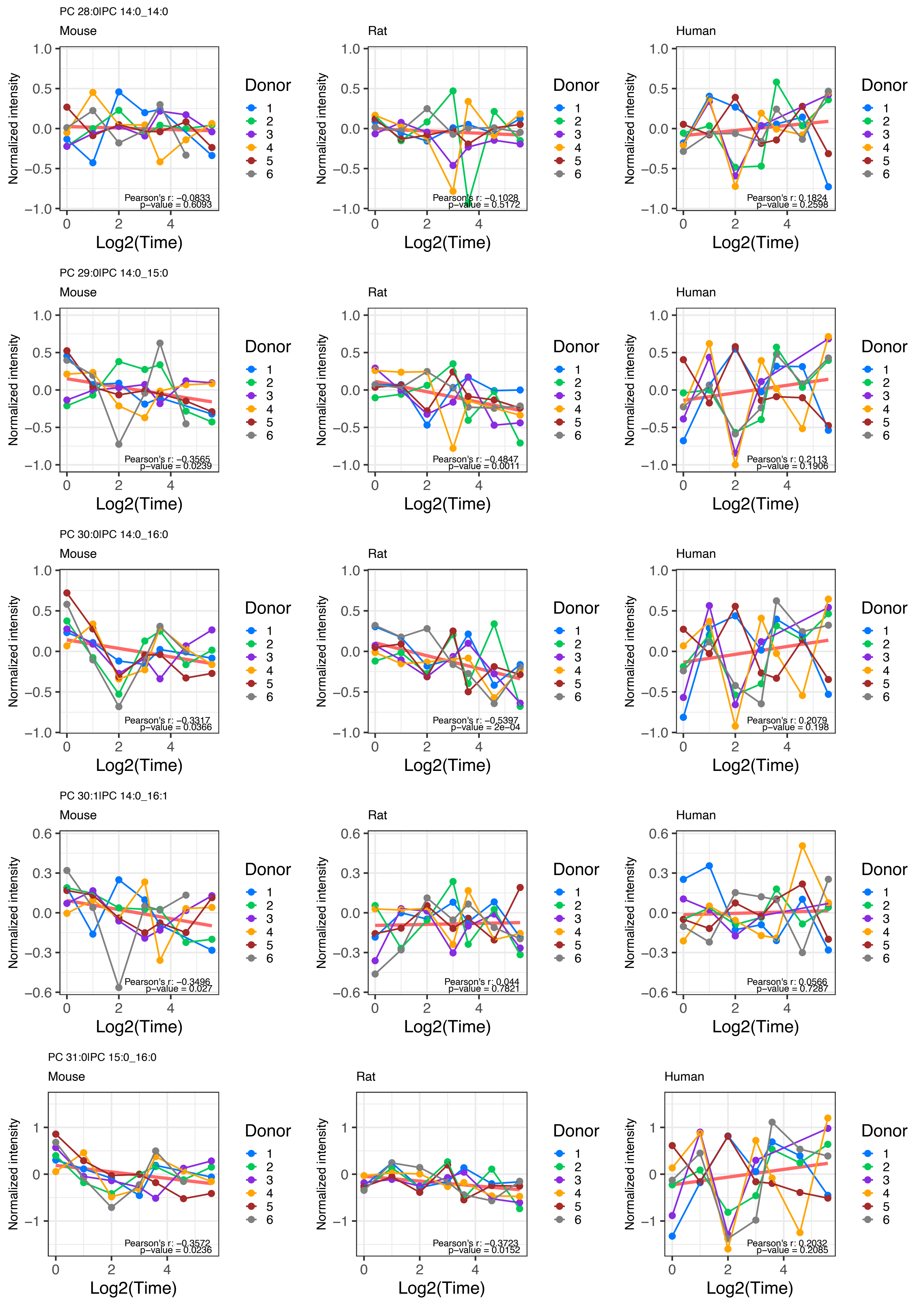
PC 28:0|PC 14:0_14:0
Mouse
Pearson's r: −0.0833
p−value = 0.6093
Rat
Pearson's r: −0.1028
p−value = 0.5172
Human
Pearson's r: 0.1824
p−value = 0.2598
PC 29:0|PC 14:0_15:0
Mouse
Pearson's r: −0.3565
p−value = 0.0239
Rat
Pearson's r: −0.4847
p−value = 0.0011
Human
Pearson's r: 0.2113
p−value = 0.1906
PC 30:0|PC 14:0_16:0
Mouse
Pearson's r: −0.3317
p−value = 0.0366
Rat
Pearson's r: −0.5397
p−value = 2e−04
Human
Pearson's r: 0.2079
p−value = 0.198
PC 30:1|PC 14:0_16:1
Mouse
Pearson's r: −0.3496
p−value = 0.027
Rat
Pearson's r: 0.044
p−value = 0.7821
Human
Pearson's r: 0.0566
p−value = 0.7287
PC 31:0|PC 15:0_16:0
Mouse
Pearson's r: −0.3572
p−value = 0.0236
Rat
Pearson's r: −0.3723
p−value = 0.0152
Human
Pearson's r: 0.2032
p−value = 0.2085
Normalized intensity
Log2(Time)
Donor
1
2
3
4
5
6

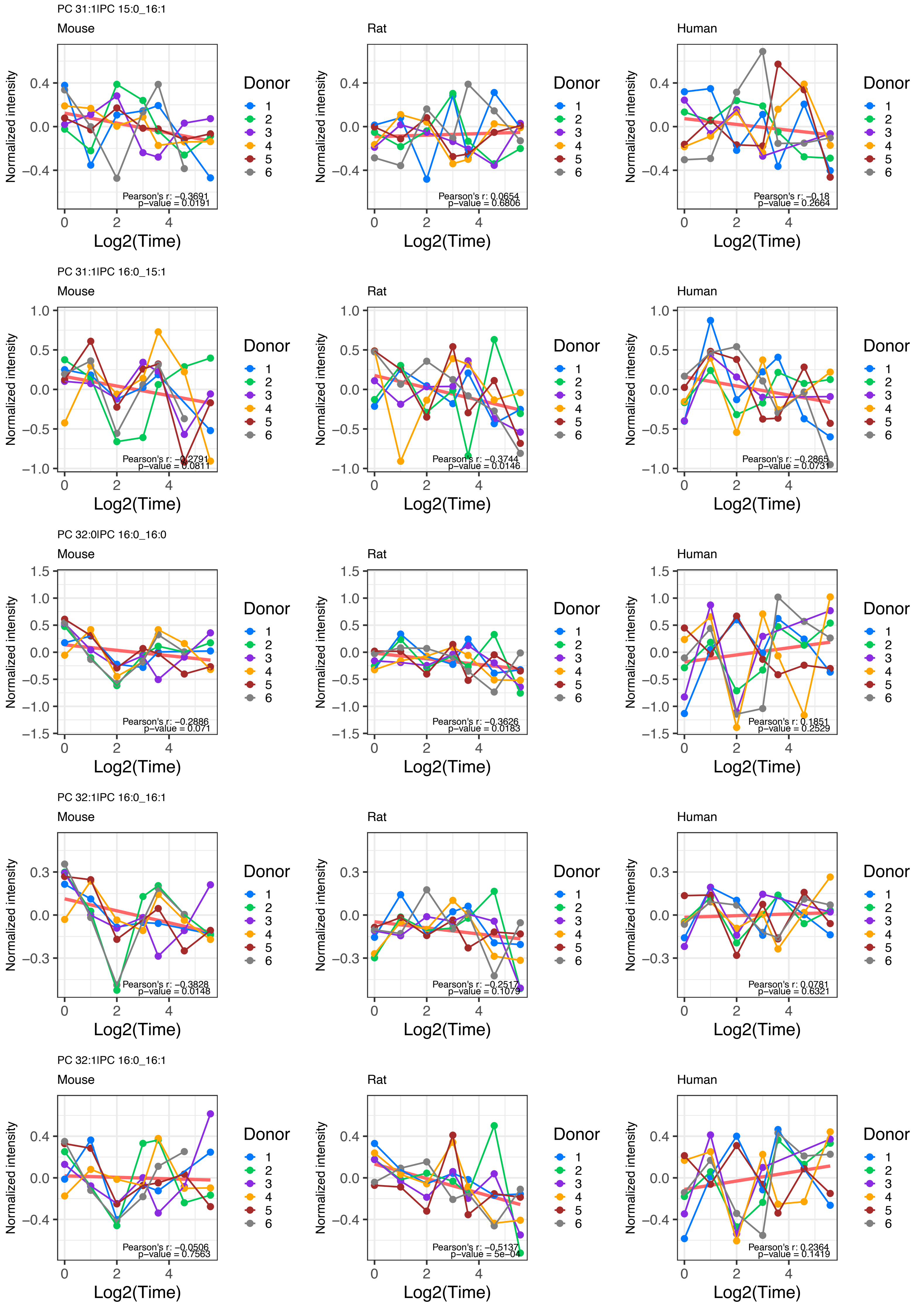
PC 31:1|PC 15:0_16:1
Mouse
Pearson's r: −0.3691
p−value = 0.0191
Rat
Pearson's r: 0.0654
p−value = 0.6806
Human
Pearson's r: −0.18
p−value = 0.2664
PC 31:1|PC 16:0_15:1
Mouse
Pearson's r: −0.2791
p−value = 0.0811
Rat
Pearson's r: −0.3744
p−value = 0.0146
Human
Pearson's r: −0.2865
p−value = 0.0731
PC 32:0|PC 16:0_16:0
Mouse
Pearson's r: −0.2886
p−value = 0.071
Rat
Pearson's r: −0.3626
p−value = 0.0183
Human
Pearson's r: 0.1851
p−value = 0.2529
PC 32:1|PC 16:0_16:1
Mouse
Pearson's r: −0.3828
p−value = 0.0148
Rat
Pearson's r: −0.2517
p−value = 0.1079
Human
Pearson's r: 0.0781
p−value = 0.6321
PC 32:1|PC 16:0_16:1
Mouse
Pearson's r: −0.0506
p−value = 0.7563
Rat
Pearson's r: −0.5137
p−value = 5e−04
Human
Pearson's r: 0.2364
p−value = 0.1419
Normalized intensity
Log2(Time)
Donor
1
2
3
4
5
6

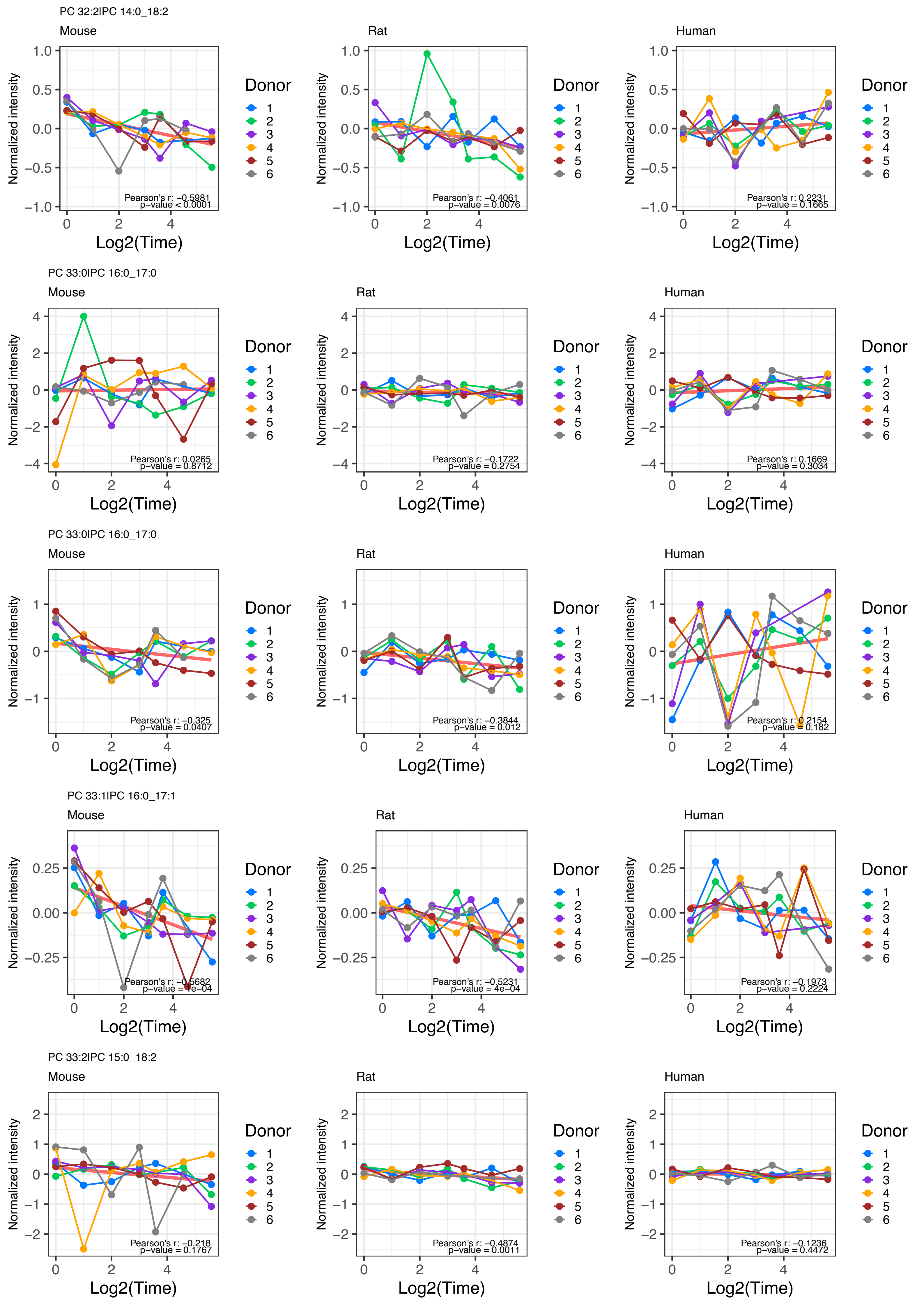

PC 32:2|PC 14:0_18:2
Mouse
Rat
Human
Normalized intensity
Log2(Time)
Donor
1
2
3
4
5
6
Pearson's r: −0.5981
p−value < 0.0001
Pearson's r: −0.4061
p−value = 0.0076
Pearson's r: 0.2231
p−value = 0.1665
PC 33:0|PC 16:0_17:0
Mouse
Rat
Human
Pearson's r: 0.0265
p−value = 0.8712
Pearson's r: −0.1722
p−value = 0.2754
Pearson's r: 0.1669
p−value = 0.3034
PC 33:0|PC 16:0_17:0
Mouse
Rat
Human
Pearson's r: −0.325
p−value = 0.0407
Pearson's r: −0.3844
p−value = 0.012
Pearson's r: 0.2154
p−value = 0.182
PC 33:1|PC 16:0_17:1
Mouse
Rat
Human
Pearson's r: −0.5682
Pearson's r: −0.5231
p−value = 4e−04
Pearson's r: −0.1973
p−value = 0.2224
PC 33:2|PC 15:0_18:2
Mouse
Rat
Human
Pearson's r: −0.218
p−value = 0.1767
Pearson's r: −0.4874
p−value = 0.0011
Pearson's r: −0.1236
p−value = 0.4472

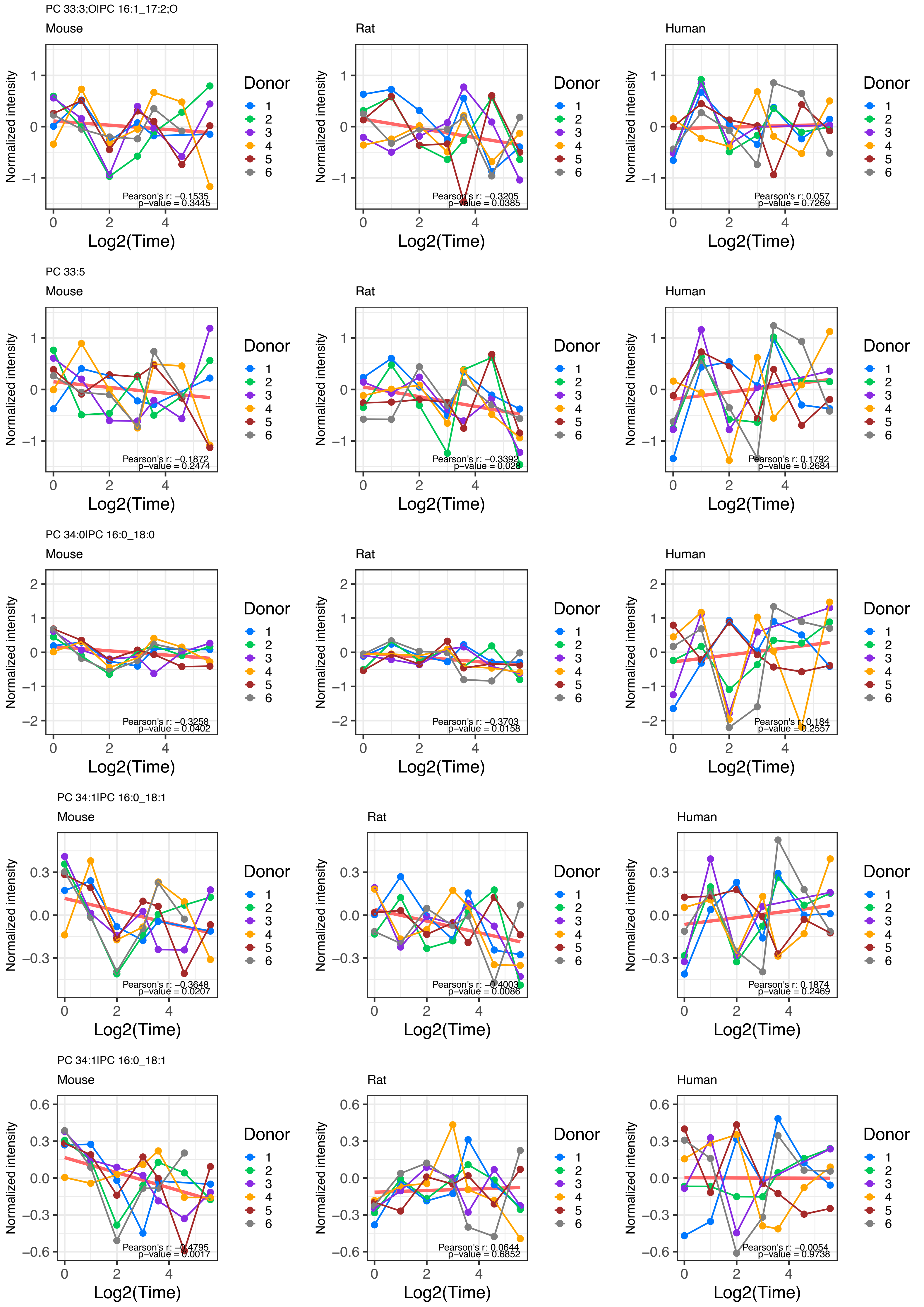
PC 33:3;O|PC 16:1_17:2;O
Mouse
Pearson's r: −0.1535
p−value = 0.3445
Rat
Pearson's r: −0.3205
p−value = 0.0385
Human
Pearson's r: 0.057
p−value = 0.7269
PC 33:5
Mouse
Pearson's r: −0.1872
p−value = 0.2474
Rat
Pearson's r: −0.3392
p−value = 0.028
Human
Pearson's r: 0.1792
p−value = 0.2684
PC 34:0|PC 16:0_18:0
Mouse
Pearson's r: −0.3258
p−value = 0.0402
Rat
Pearson's r: −0.3703
p−value = 0.0158
Human
Pearson's r: 0.184
p−value = 0.2557
PC 34:1|PC 16:0_18:1
Mouse
Pearson's r: −0.3648
p−value = 0.0207
Rat
Pearson's r: −0.4003
p−value = 0.0086
Human
Pearson's r: 0.1874
p−value = 0.2469
PC 34:1|PC 16:0_18:1
Mouse
Pearson's r: −0.4795
p−value = 0.0017
Rat
Pearson's r: 0.0644
p−value = 0.6852
Human
Pearson's r: −0.0054
p−value = 0.9738
Normalized intensity
Log2(Time)
Donor
1
2
3
4
5
6

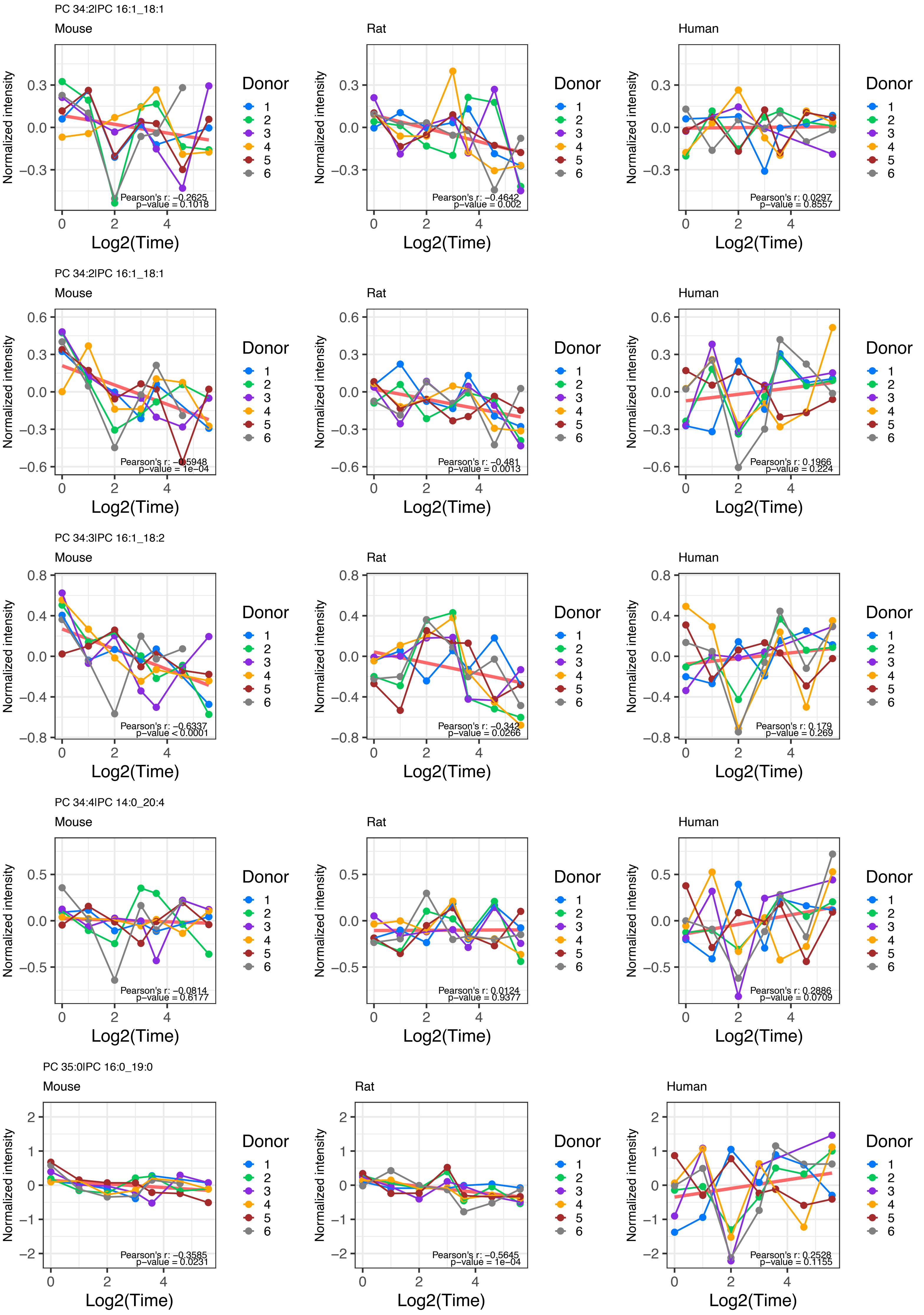
PC 34:2|PC 16:1_18:1
Mouse
Pearson's r: −0.2625
p−value = 0.1018
Rat
Pearson's r: −0.4642
p−value = 0.002
Human
Pearson's r: 0.0297
p−value = 0.8557
PC 34:2|PC 16:1_18:1
Mouse
Pearson's r: −0.5948
p−value = 1e−04
Rat
Pearson's r: −0.481
p−value = 0.0013
Human
Pearson's r: 0.1966
p−value = 0.224
PC 34:3|PC 16:1_18:2
Mouse
Pearson's r: −0.6337
p−value < 0.0001
Rat
Pearson's r: −0.342
p−value = 0.0266
Human
Pearson's r: 0.179
p−value = 0.269
PC 34:4|PC 14:0_20:4
Mouse
Pearson's r: −0.0814
p−value = 0.6177
Rat
Pearson's r: 0.0124
p−value = 0.9377
Human
Pearson's r: 0.2886
p−value = 0.0709
PC 35:0|PC 16:0_19:0
Mouse
Pearson's r: −0.3585
p−value = 0.0231
Rat
Pearson's r: −0.5645
p−value = 1e−04
Human
Pearson's r: 0.2528
p−value = 0.1155
Normalized intensity
Log2(Time)
Donor
1
2
3
4
5
6

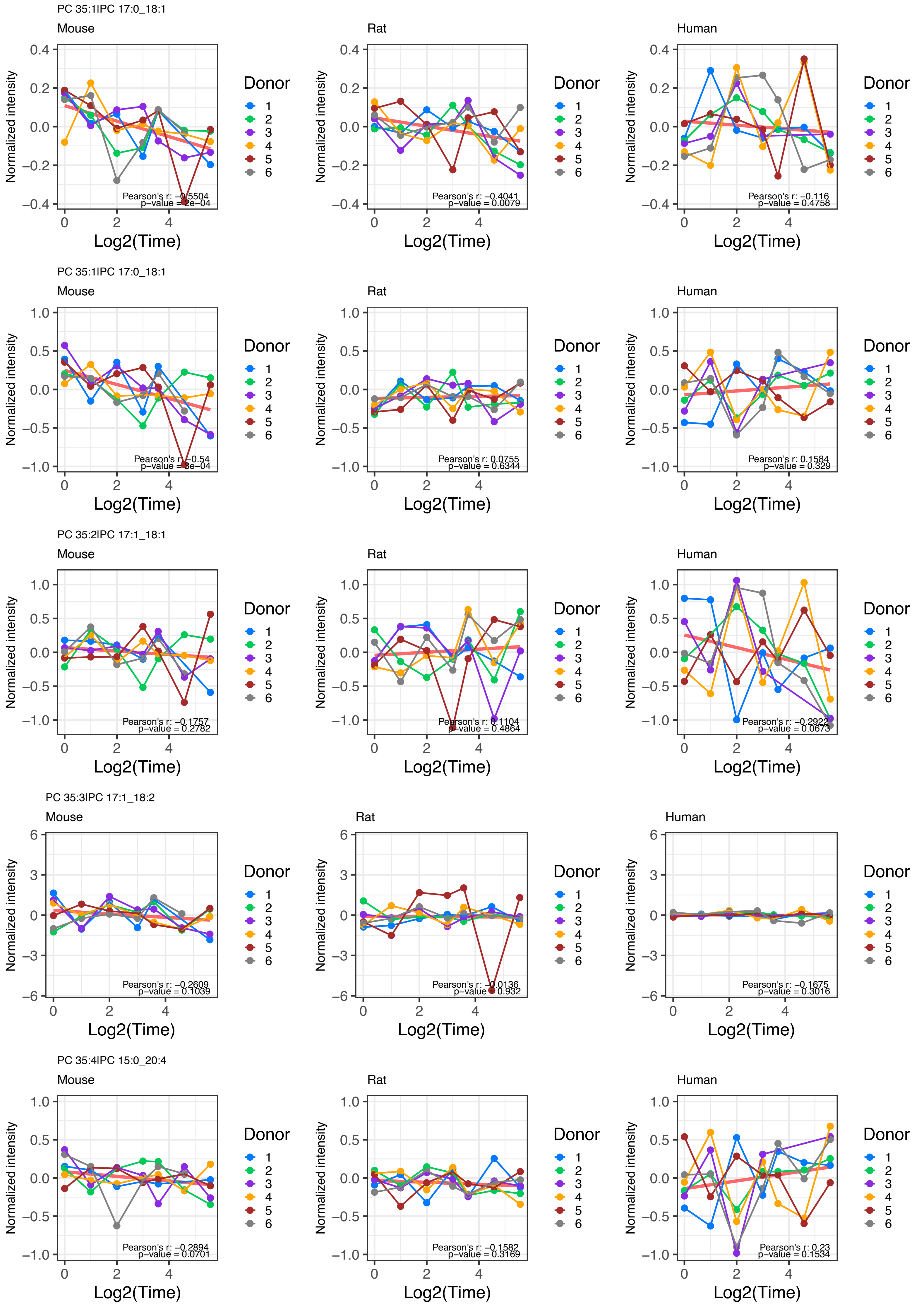
PC 35:1|PC 17:0_18:1
Mouse
Rat
Human
Normalized intensity
Log2(Time)
Donor
1
2
3
4
5
6
Pearson's r: −0.5504
p−value = 2e−04
Pearson's r: −0.4041
p−value = 0.0079
Pearson's r: −0.116
p−value = 0.4758
PC 35:1|PC 17:0_18:1
Pearson's r: −0.54
p−value = 3e−04
Pearson's r: 0.0755
p−value = 0.6344
Pearson's r: 0.1584
p−value = 0.329
PC 35:2|PC 17:1_18:1
Pearson's r: −0.1757
p−value = 0.2782
Pearson's r: 0.1104
p−value = 0.4864
Pearson's r: −0.2922
p−value = 0.0673
PC 35:3|PC 17:1_18:2
Pearson's r: −0.2609
p−value = 0.1039
Pearson's r: −0.0136
p−value = 0.932
Pearson's r: −0.1675
p−value = 0.3016
PC 35:4|PC 15:0_20:4
Pearson's r: −0.2894
p−value = 0.0701
Pearson's r: −0.1582
p−value = 0.3169
Pearson's r: 0.23
p−value = 0.1534

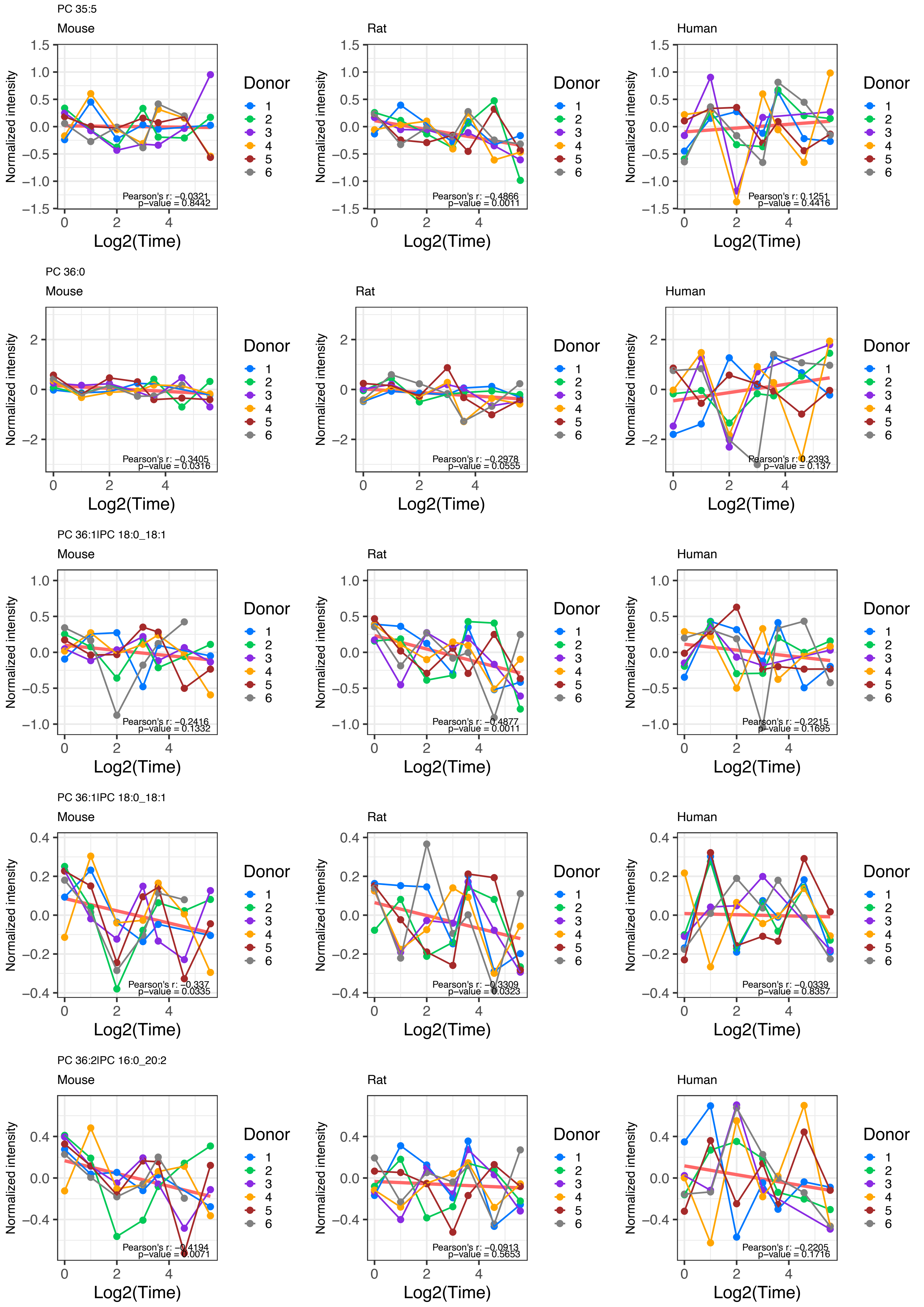
PC 35:5
Mouse
Rat
Human
Normalized intensity
Log2(Time)
Donor
1
2
3
4
5
6
Pearson's r: −0.0321
p−value = 0.8442
Pearson's r: −0.4866
p−value = 0.0011
Pearson's r: 0.1251
p−value = 0.4416
PC 36:0
Mouse
Rat
Human
Pearson's r: −0.3405
p−value = 0.0316
Pearson's r: −0.2978
p−value = 0.0555
Pearson's r: 0.2393
p−value = 0.137
PC 36:1|PC 18:0_18:1
Mouse
Rat
Human
Pearson's r: −0.2416
p−value = 0.1332
Pearson's r: −0.4877
p−value = 0.0011
Pearson's r: −0.2215
p−value = 0.1695
PC 36:1|PC 18:0_18:1
Mouse
Rat
Human
Pearson's r: −0.337
p−value = 0.0335
Pearson's r: −0.3309
p−value = 0.0323
Pearson's r: −0.0339
p−value = 0.8357
PC 36:2|PC 16:0_20:2
Mouse
Rat
Human
Pearson's r: −0.4194
p−value = 0.0071
Pearson's r: −0.0913
p−value = 0.5653
Pearson's r: −0.2205
p−value = 0.1716

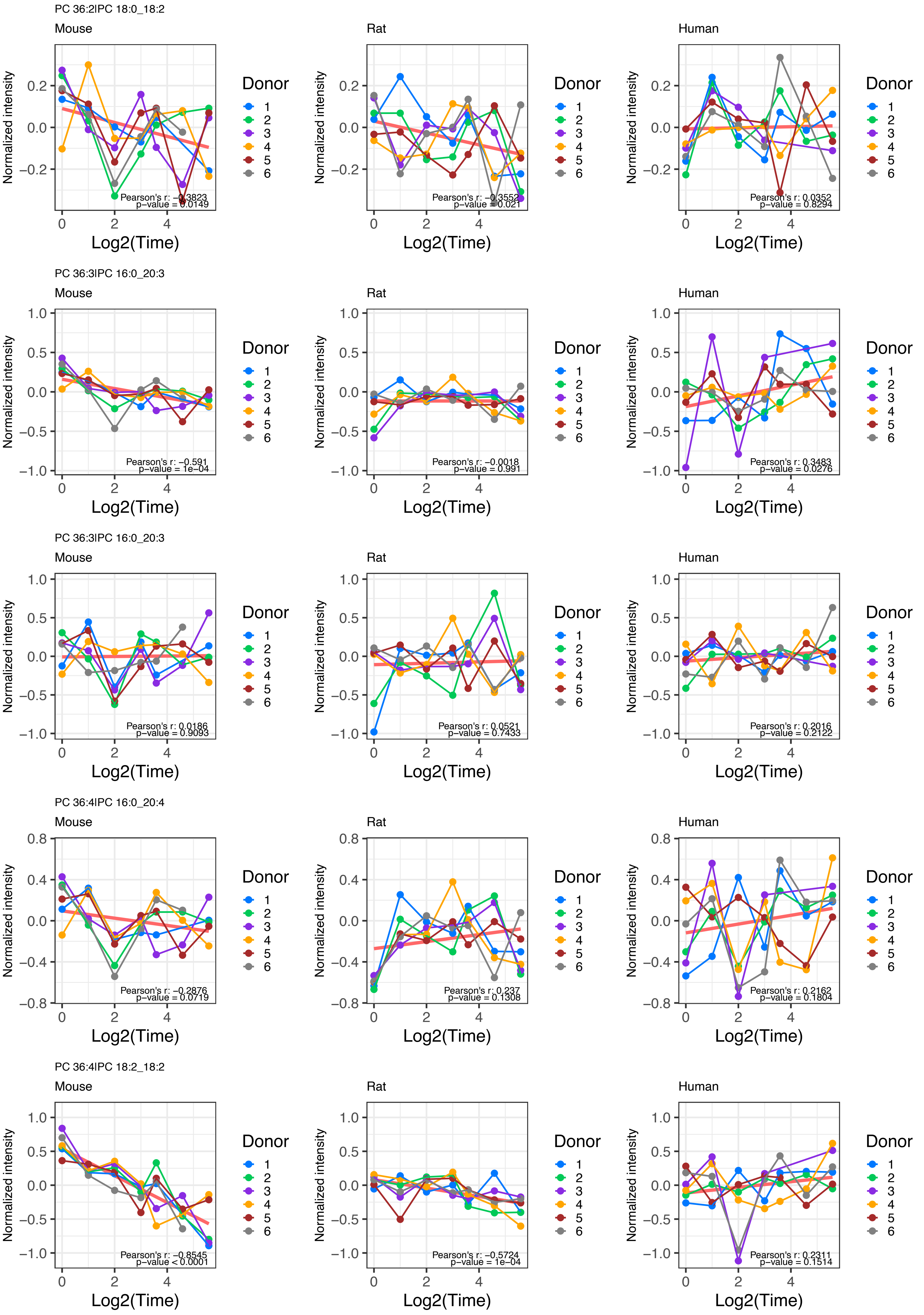
PC 36:2|PC 18:0_18:2
Mouse
Pearson's r: −0.3823
p−value = 0.0149
Rat
Pearson's r: −0.3552
p−value = 0.021
Human
Pearson's r: 0.0352
p−value = 0.8294
PC 36:3|PC 16:0_20:3
Mouse
Pearson's r: −0.591
p−value = 1e−04
Rat
Pearson's r: −0.0018
p−value = 0.991
Human
Pearson's r: 0.3483
p−value = 0.0276
PC 36:3|PC 16:0_20:3
Mouse
Pearson's r: 0.0186
p−value = 0.9093
Rat
Pearson's r: 0.0521
p−value = 0.7433
Human
Pearson's r: 0.2016
p−value = 0.2122
PC 36:4|PC 16:0_20:4
Mouse
Pearson's r: −0.2876
p−value = 0.0719
Rat
Pearson's r: 0.237
p−value = 0.1308
Human
Pearson's r: 0.2162
p−value = 0.1804
PC 36:4|PC 18:2_18:2
Mouse
Pearson's r: −0.8545
p−value < 0.0001
Rat
Pearson's r: −0.5724
p−value = 1e−04
Human
Pearson's r: 0.2311
p−value = 0.1514
Normalized intensity
Log2(Time)
Donor
1
2
3
4
5
6

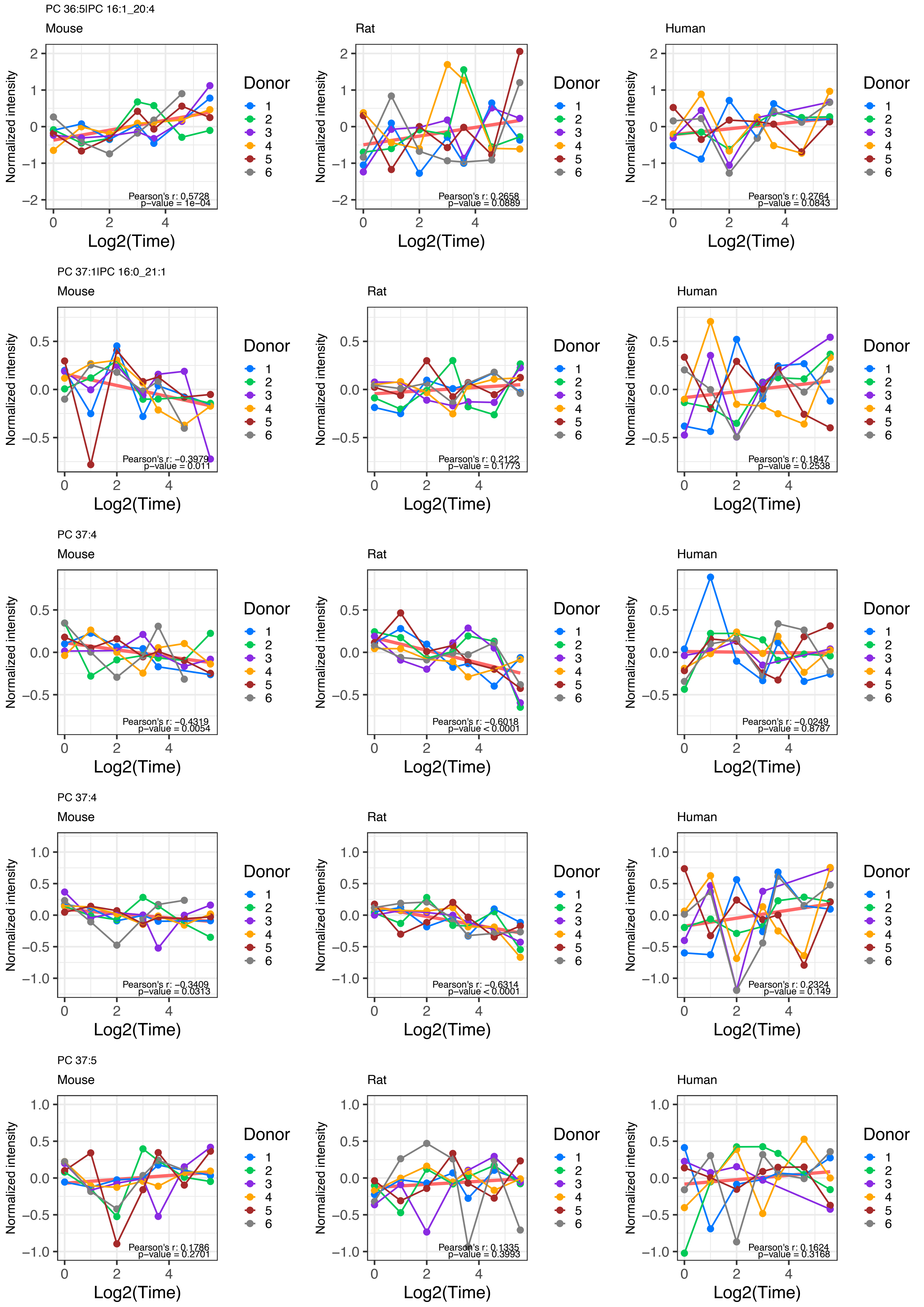
PC 36:5|PC 16:1_20:4
Mouse
Pearson's r: 0.5728
p−value = 1e−04
Rat
Pearson's r: 0.2658
p−value = 0.0889
Human
Pearson's r: 0.2764
p−value = 0.0843
PC 37:1|PC 16:0_21:1
Mouse
Pearson's r: −0.3979
p−value = 0.011
Rat
Pearson's r: 0.2122
p−value = 0.1773
Human
Pearson's r: 0.1847
p−value = 0.2538
PC 37:4
Mouse
Pearson's r: −0.4319
p−value = 0.0054
Rat
Pearson's r: −0.6018
p−value < 0.0001
Human
Pearson's r: −0.0249
p−value = 0.8787
PC 37:4
Mouse
Pearson's r: −0.3409
p−value = 0.0313
Rat
Pearson's r: −0.6314
p−value < 0.0001
Human
Pearson's r: 0.2324
p−value = 0.149
PC 37:5
Mouse
Pearson's r: 0.1786
p−value = 0.2701
Rat
Pearson's r: 0.1335
p−value = 0.3993
Human
Pearson's r: 0.1624
p−value = 0.3168
Normalized intensity
Log2(Time)
Donor
1
2
3
4
5
6

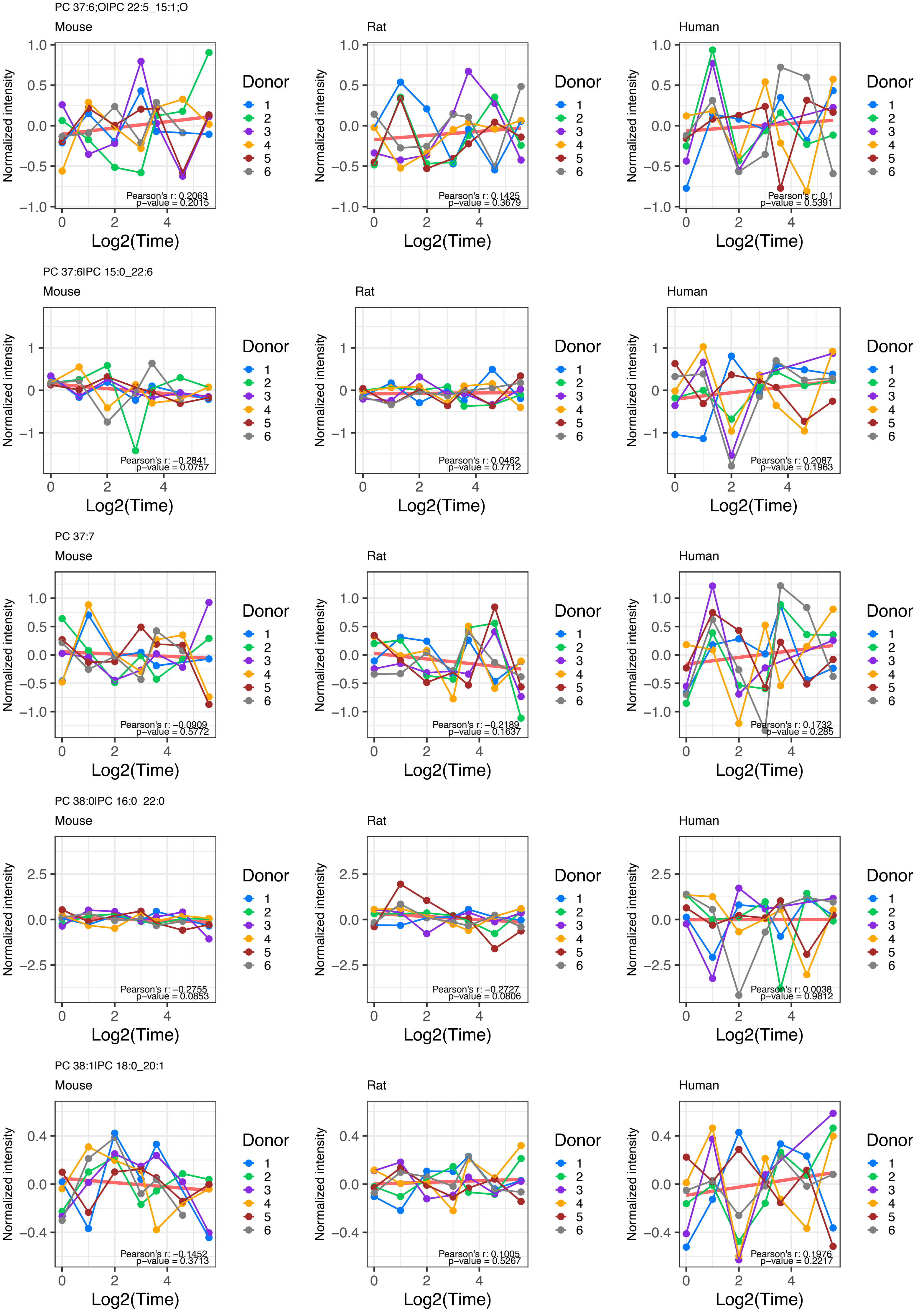
PC 37:6;O|PC 22:5_15:1;O
Mouse
Pearson's r: 0.2063
p−value = 0.2015
Rat
Pearson's r: 0.1425
p−value = 0.3679
Human
Pearson's r: 0.1
p−value = 0.5391
PC 37:6|PC 15:0_22:6
Mouse
Pearson's r: −0.2841
p−value = 0.0757
Rat
Pearson's r: 0.0462
p−value = 0.7712
Human
Pearson's r: 0.2087
p−value = 0.1963
PC 37:7
Mouse
Pearson's r: −0.0909
p−value = 0.5772
Rat
Pearson's r: −0.2189
p−value = 0.1637
Human
Pearson's r: 0.1732
p−value = 0.285
PC 38:0|PC 16:0_22:0
Mouse
Pearson's r: −0.2755
p−value = 0.0853
Rat
Pearson's r: −0.2727
p−value = 0.0806
Human
Pearson's r: 0.0038
p−value = 0.9812
PC 38:1|PC 18:0_20:1
Mouse
Pearson's r: −0.1452
p−value = 0.3713
Rat
Pearson's r: 0.1005
p−value = 0.5267
Human
Pearson's r: 0.1976
p−value = 0.2217
Normalized intensity
Log2(Time)
Donor
1
2
3
4
5
6

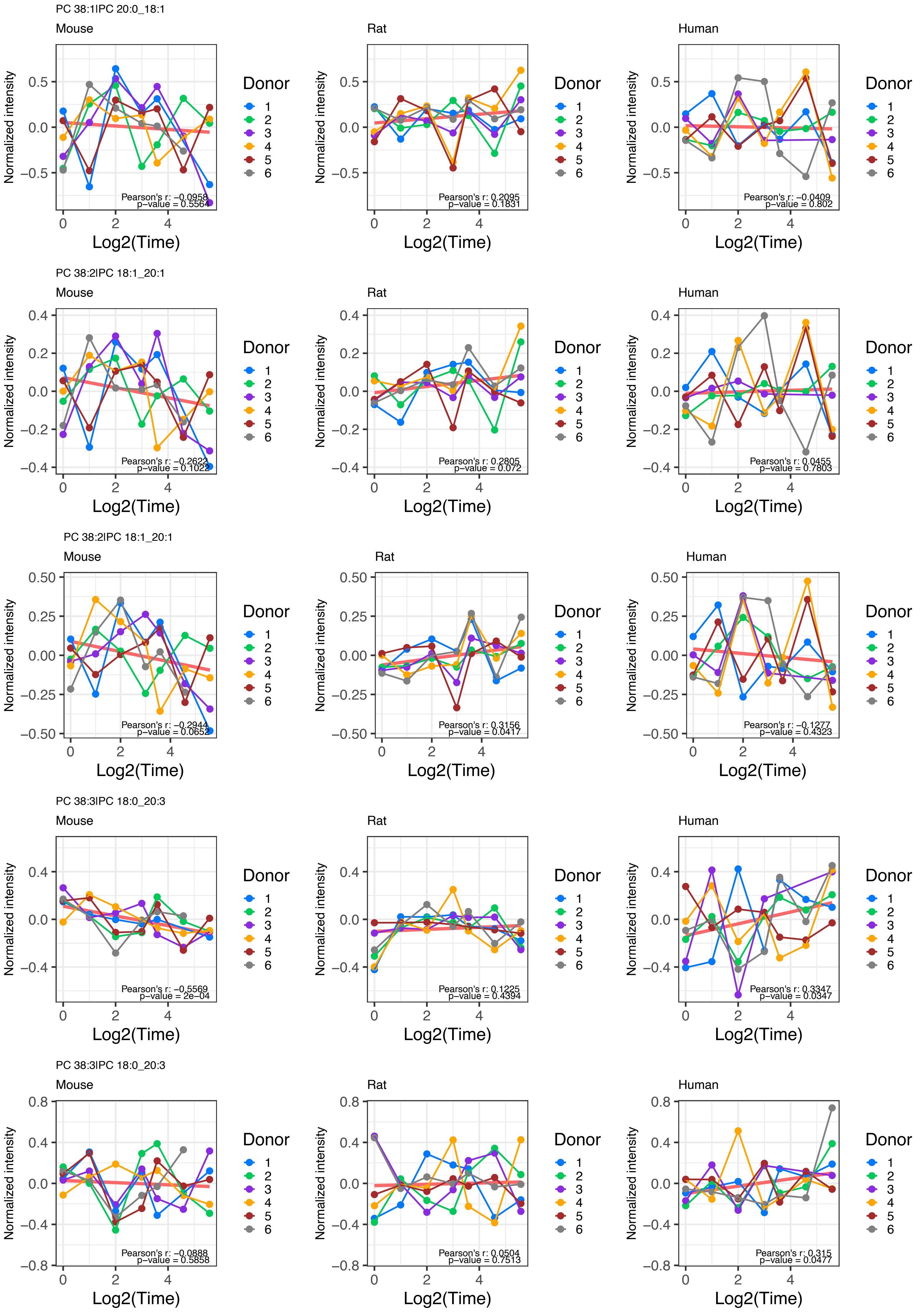

PC 38:1|PC 20:0_18:1
Mouse
Pearson's r: −0.0958
p−value = 0.5564
Rat
Pearson's r: 0.2095
p−value = 0.1831
Human
Pearson's r: −0.0409
p−value = 0.802
PC 38:2|PC 18:1_20:1
Mouse
Pearson's r: −0.2622
p−value = 0.1022
Rat
Pearson's r: 0.2805
p−value = 0.072
Human
Pearson's r: 0.0455
p−value = 0.7803
PC 38:2|PC 18:1_20:1
Mouse
Pearson's r: −0.2944
p−value = 0.0652
Rat
Pearson's r: 0.3156
p−value = 0.0417
Human
Pearson's r: −0.1277
p−value = 0.4323
PC 38:3|PC 18:0_20:3
Mouse
Pearson's r: −0.5569
p−value = 2e−04
Rat
Pearson's r: 0.1225
p−value = 0.4394
Human
Pearson's r: 0.3347
p−value = 0.0347
PC 38:3|PC 18:0_20:3
Mouse
Pearson's r: −0.0888
p−value = 0.5858
Rat
Pearson's r: 0.0504
p−value = 0.7513
Human
Pearson's r: 0.315
p−value = 0.0477
Normalized intensity
Log2(Time)
Donor
1
2
3
4
5
6

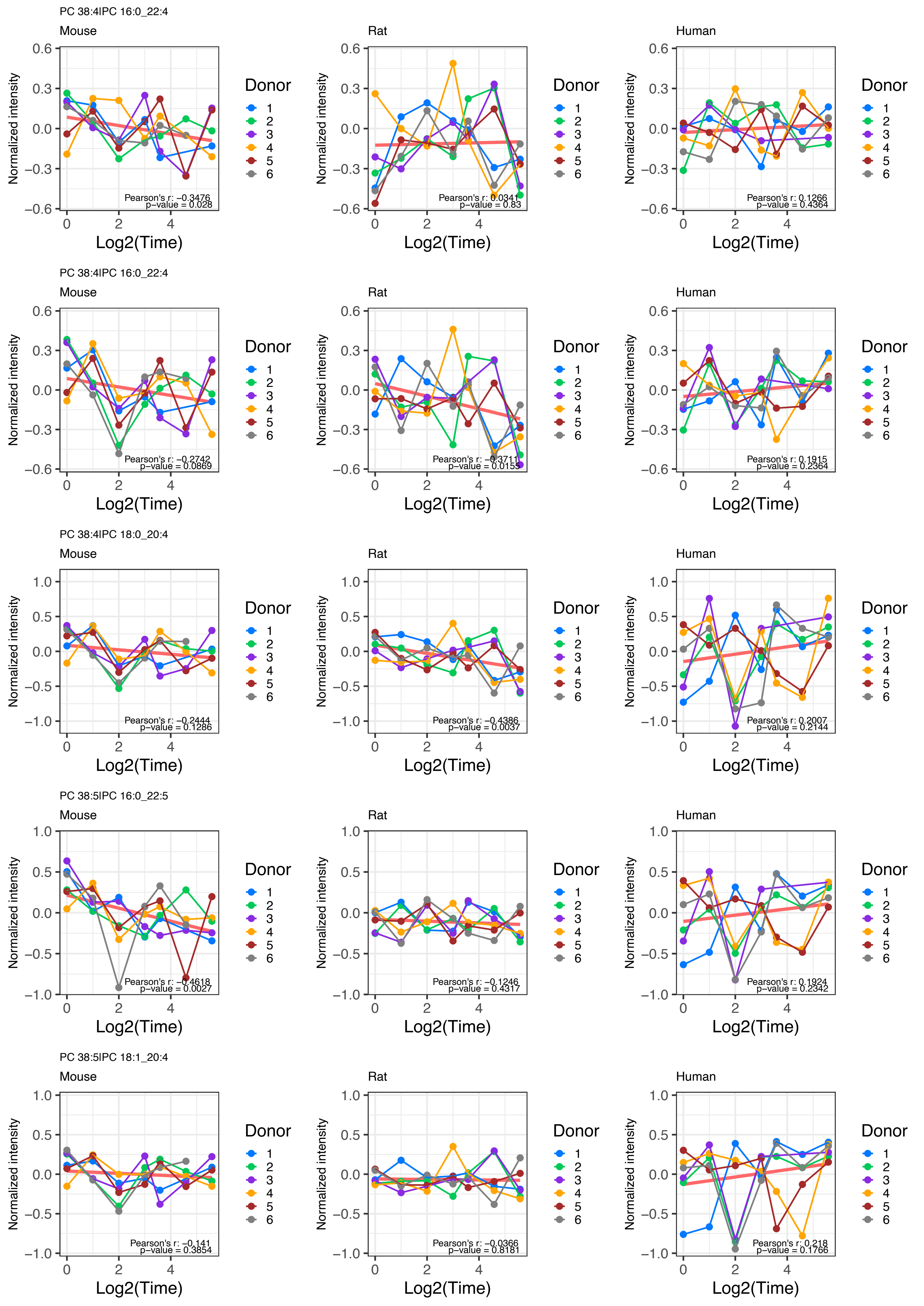
PC 38:4|PC 16:0_22:4
Mouse
Pearson's r: −0.3476
p−value = 0.028
Rat
Pearson's r: 0.0341
p−value = 0.83
Human
Pearson's r: 0.1266
p−value = 0.4364
PC 38:4|PC 16:0_22:4
Mouse
Pearson's r: −0.2742
p−value = 0.0869
Rat
Pearson's r: −0.3711
p−value = 0.0155
Human
Pearson's r: 0.1915
p−value = 0.2364
PC 38:4|PC 18:0_20:4
Mouse
Pearson's r: −0.2444
p−value = 0.1286
Rat
Pearson's r: −0.4386
p−value = 0.0037
Human
Pearson's r: 0.2007
p−value = 0.2144
PC 38:5|PC 16:0_22:5
Mouse
Pearson's r: −0.4618
p−value = 0.0027
Rat
Pearson's r: −0.1246
p−value = 0.4317
Human
Pearson's r: 0.1924
p−value = 0.2342
PC 38:5|PC 18:1_20:4
Mouse
Pearson's r: −0.141
p−value = 0.3854
Rat
Pearson's r: −0.0366
p−value = 0.8181
Human
Pearson's r: 0.218
p−value = 0.1766
Normalized intensity
Log2(Time)
Donor
1
2
3
4
5
6

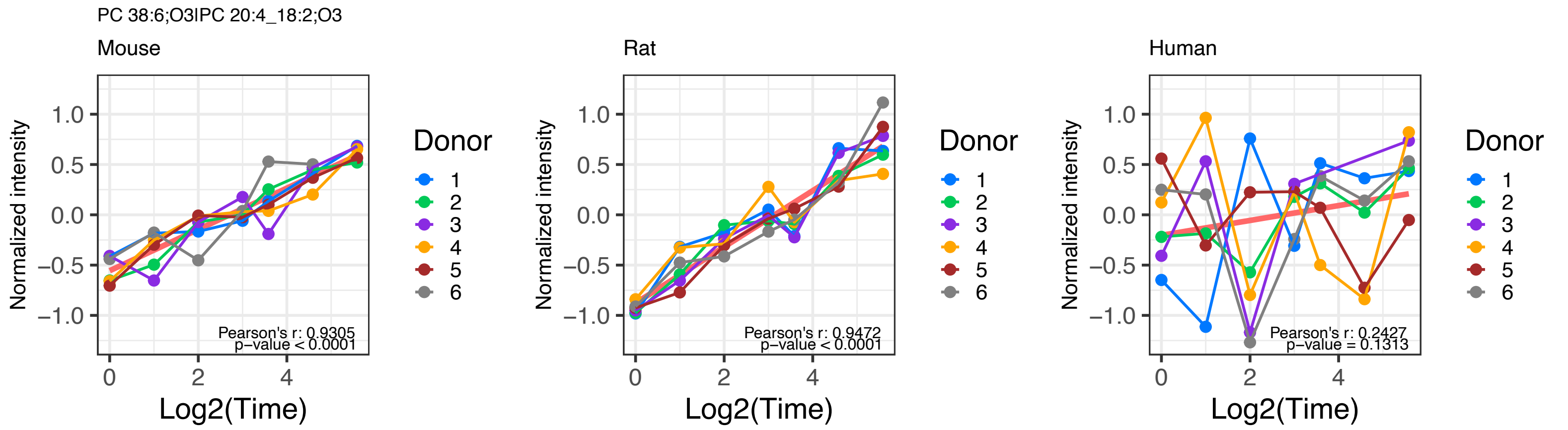
PC 38:6;O3|PC 20:4_18:2;O3
Mouse
Rat
Human
Normalized intensity
Log2(Time)
Donor
1
2
3
4
5
6
Pearson's r: 0.9305
p−value < 0.0001
Pearson's r: 0.9472
p−value < 0.0001
Pearson's r: 0.2427
p−value = 0.1313

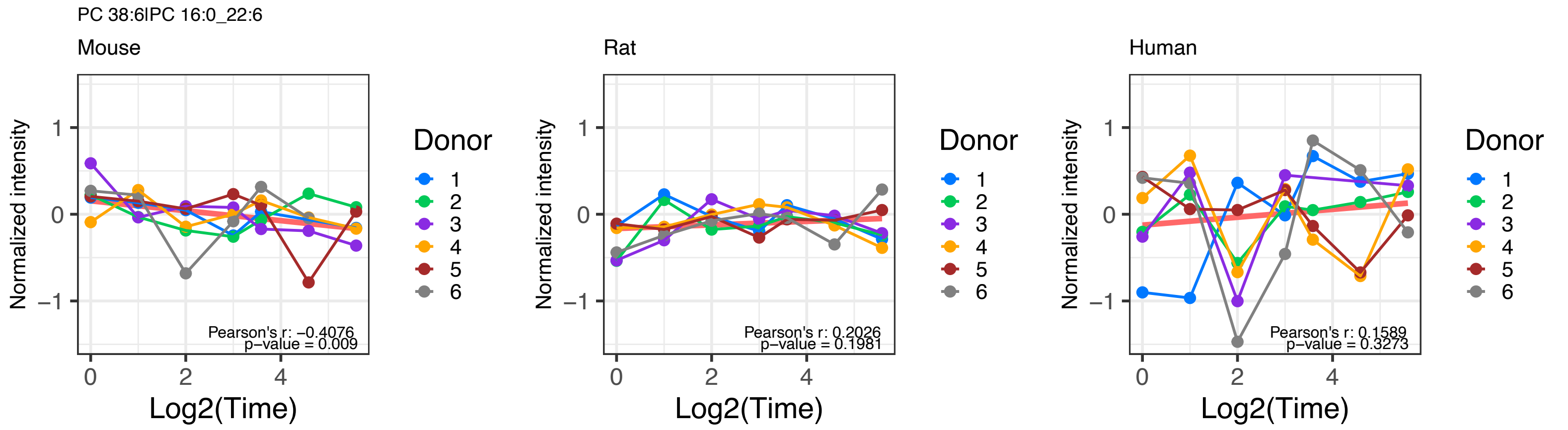
PC 38:6|PC 16:0_22:6
Mouse
Rat
Human
Normalized intensity
Log2(Time)
Donor
1
2
3
4
5
6
Pearson's r: −0.4076
p−value = 0.009
Pearson's r: 0.2026
p−value = 0.1981
Pearson's r: 0.1589
p−value = 0.3273

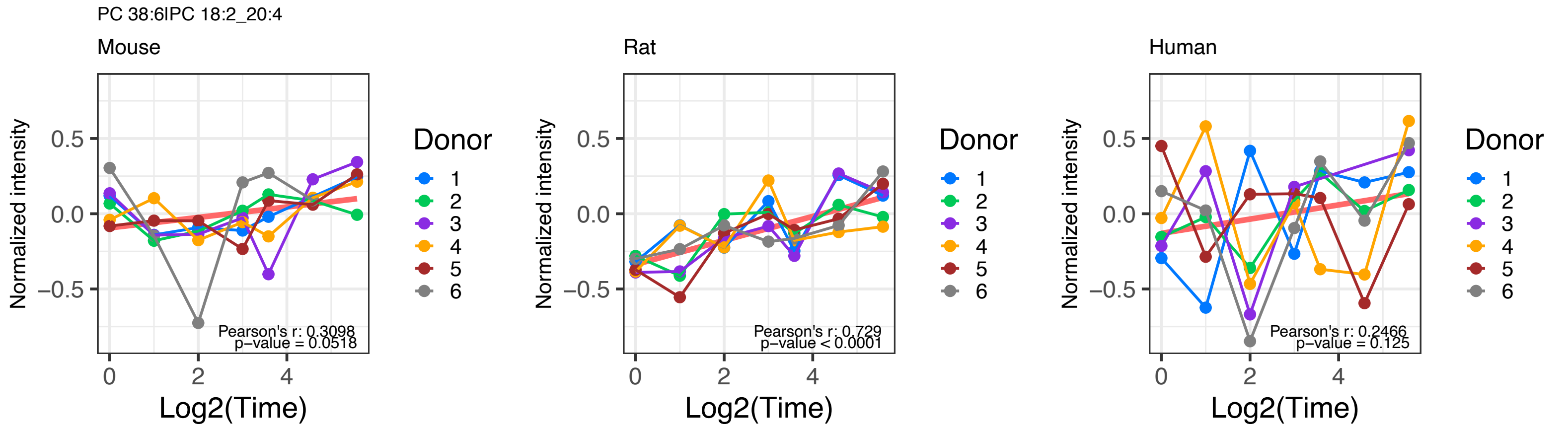
PC 38:6|PC 18:2_20:4
Mouse
Rat
Human
Normalized intensity
Log2(Time)
Donor
1
2
3
4
5
6
Pearson's r: 0.3098
p−value = 0.0518
Pearson's r: 0.729
p−value < 0.0001
Pearson's r: 0.2466
p−value = 0.125

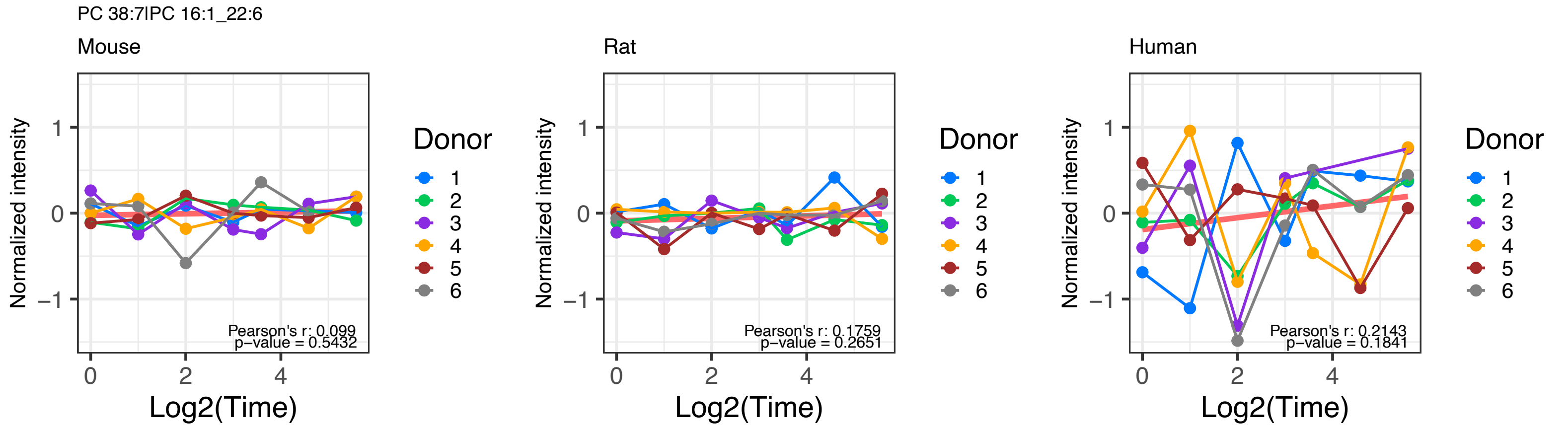
PC 38:7|PC 16:1_22:6
Mouse
Rat
Human
Normalized intensity
Log2(Time)
Donor
1
2
3
4
5
6
Pearson's r: 0.099
p−value = 0.5432
Pearson's r: 0.1759
p−value = 0.2651
Pearson's r: 0.2143
p−value = 0.1841

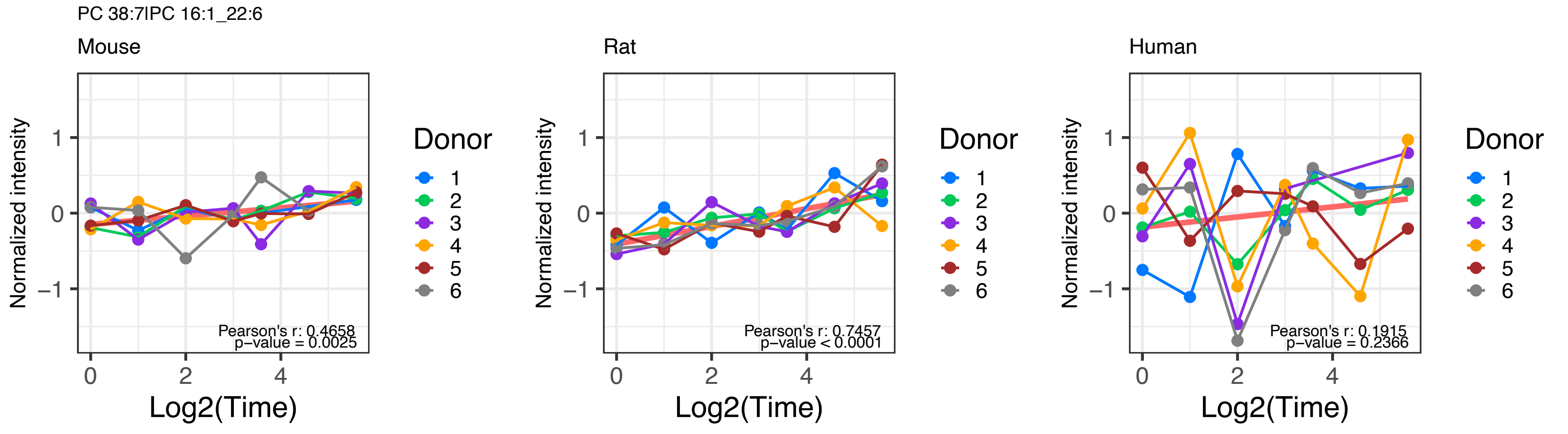
PC 38:7|PC 16:1_22:6
Mouse
Rat
Human
Normalized intensity
Log2(Time)
Donor
1
2
3
4
5
6
Pearson's r: 0.4658
p−value = 0.0025
Pearson's r: 0.7457
p−value < 0.0001
Pearson's r: 0.1915
p−value = 0.2366

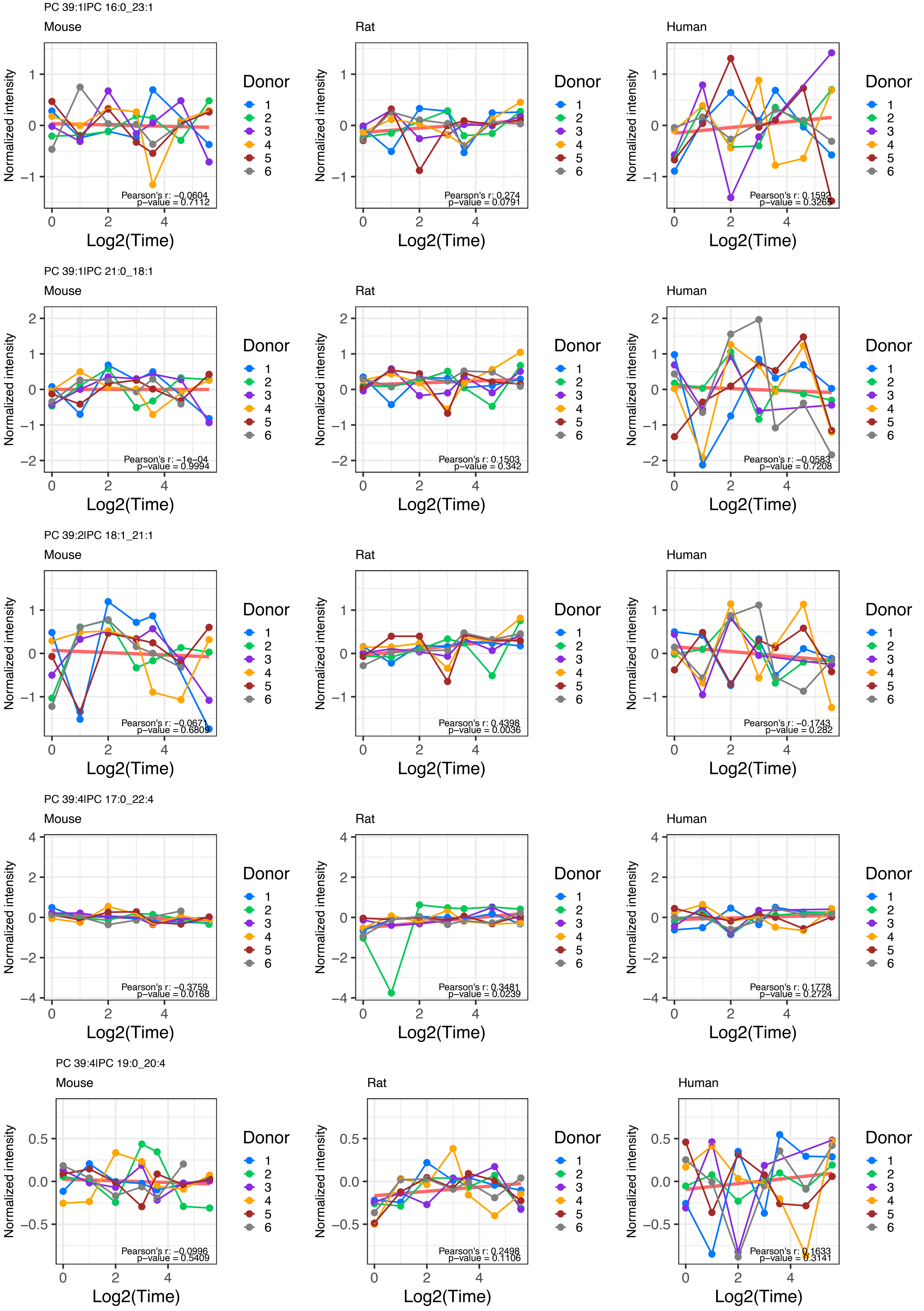

PC 39:1|PC 16:0_23:1
Mouse
Rat
Human
Normalized intensity
Log2(Time)
Donor
1
2
3
4
5
6
Pearson's r: −0.0604
p−value = 0.7112
Pearson's r: 0.274
p−value = 0.0791
Pearson's r: 0.1592
p−value = 0.3265
PC 39:1|PC 21:0_18:1
Pearson's r: −1e−04
p−value = 0.9994
Pearson's r: 0.1503
p−value = 0.342
Pearson's r: −0.0583
p−value = 0.7208
PC 39:2|PC 18:1_21:1
Pearson's r: −0.0671
p−value = 0.6809
Pearson's r: 0.4398
p−value = 0.0036
Pearson's r: −0.1743
p−value = 0.282
PC 39:4|PC 17:0_22:4
Pearson's r: −0.3759
p−value = 0.0168
Pearson's r: 0.3481
p−value = 0.0239
Pearson's r: 0.1778
p−value = 0.2724
PC 39:4|PC 19:0_20:4
Pearson's r: −0.0996
p−value = 0.5409
Pearson's r: 0.2498
p−value = 0.1106
Pearson's r: 0.1633
p−value = 0.3141

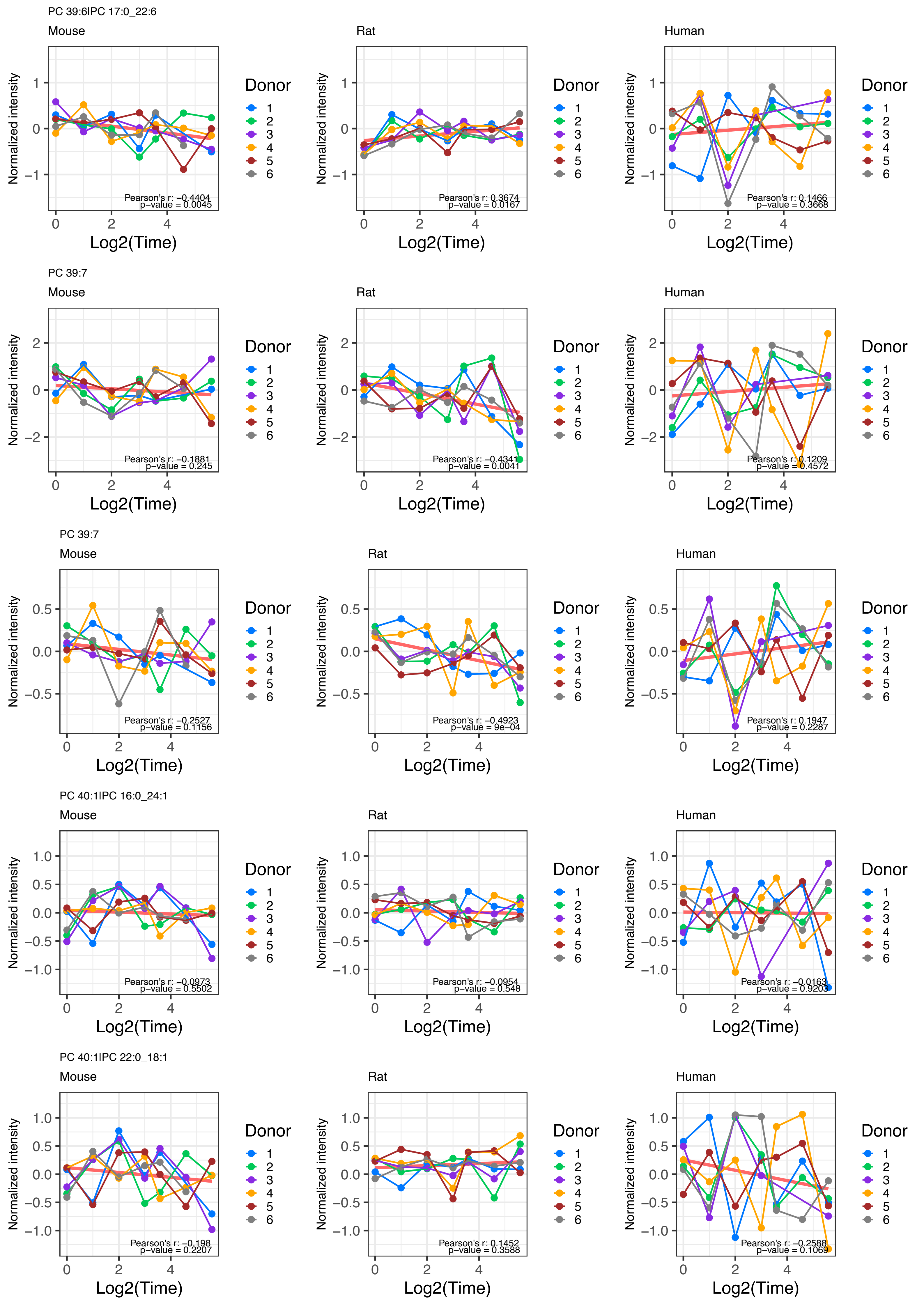

PC 39:6|PC 17:0_22:6
Mouse
Pearson's r: −0.4404
p−value = 0.0045
Rat
Pearson's r: 0.3674
p−value = 0.0167
Human
Pearson's r: 0.1466
p−value = 0.3668
PC 39:7
Mouse
Pearson's r: −0.1881
p−value = 0.245
Rat
Pearson's r: −0.4341
p−value = 0.0041
Human
Pearson's r: 0.1209
p−value = 0.4572
PC 39:7
Mouse
Pearson's r: −0.2527
p−value = 0.1156
Rat
Pearson's r: −0.4923
p−value = 9e−04
Human
Pearson's r: 0.1947
p−value = 0.2287
PC 40:1|PC 16:0_24:1
Mouse
Pearson's r: −0.0973
p−value = 0.5502
Rat
Pearson's r: −0.0954
p−value = 0.548
Human
Pearson's r: −0.0163
p−value = 0.9203
PC 40:1|PC 22:0_18:1
Mouse
Pearson's r: −0.198
p−value = 0.2207
Rat
Pearson's r: 0.1452
p−value = 0.3588
Human
Pearson's r: −0.2588
p−value = 0.1069
Normalized intensity
Log2(Time)
Donor
1
2
3
4
5
6

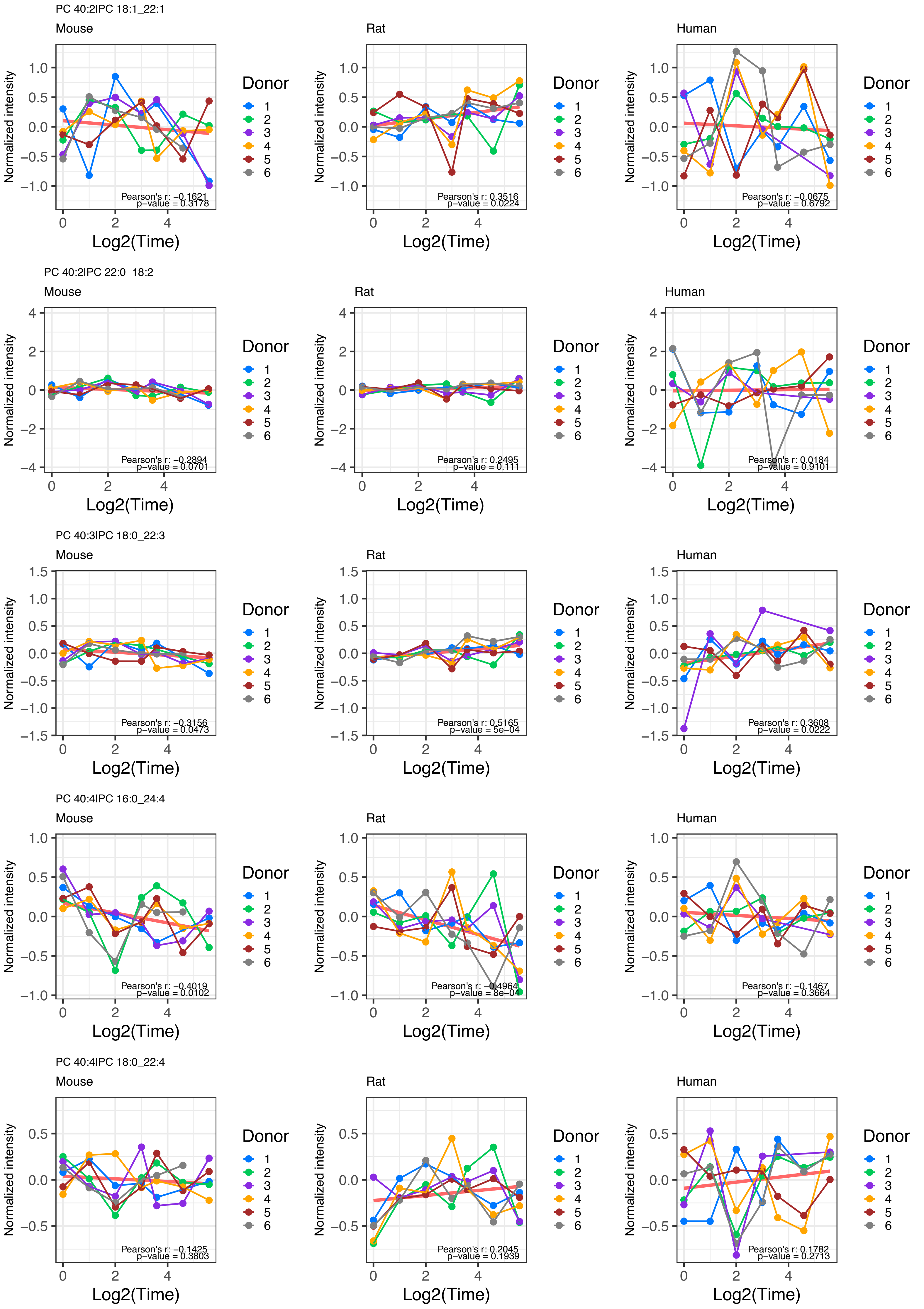
PC 40:2|PC 18:1_22:1
Mouse
Pearson's r: −0.1621
p−value = 0.3178
Rat
Pearson's r: 0.3516
p−value = 0.0224
Human
Pearson's r: −0.0675
p−value = 0.6792
PC 40:2|PC 22:0_18:2
Mouse
Pearson's r: −0.2894
p−value = 0.0701
Rat
Pearson's r: 0.2495
p−value = 0.111
Human
Pearson's r: 0.0184
p−value = 0.9101
PC 40:3|PC 18:0_22:3
Mouse
Pearson's r: −0.3156
p−value = 0.0473
Rat
Pearson's r: 0.5165
p−value = 5e−04
Human
Pearson's r: 0.3608
p−value = 0.0222
PC 40:4|PC 16:0_24:4
Mouse
Pearson's r: −0.4019
p−value = 0.0102
Rat
Pearson's r: −0.4964
p−value = 8e−04
Human
Pearson's r: −0.1467
p−value = 0.3664
PC 40:4|PC 18:0_22:4
Mouse
Pearson's r: −0.1425
p−value = 0.3803
Rat
Pearson's r: 0.2045
p−value = 0.1939
Human
Pearson's r: 0.1782
p−value = 0.2713
Normalized intensity
Log2(Time)
Donor
1
2
3
4
5
6

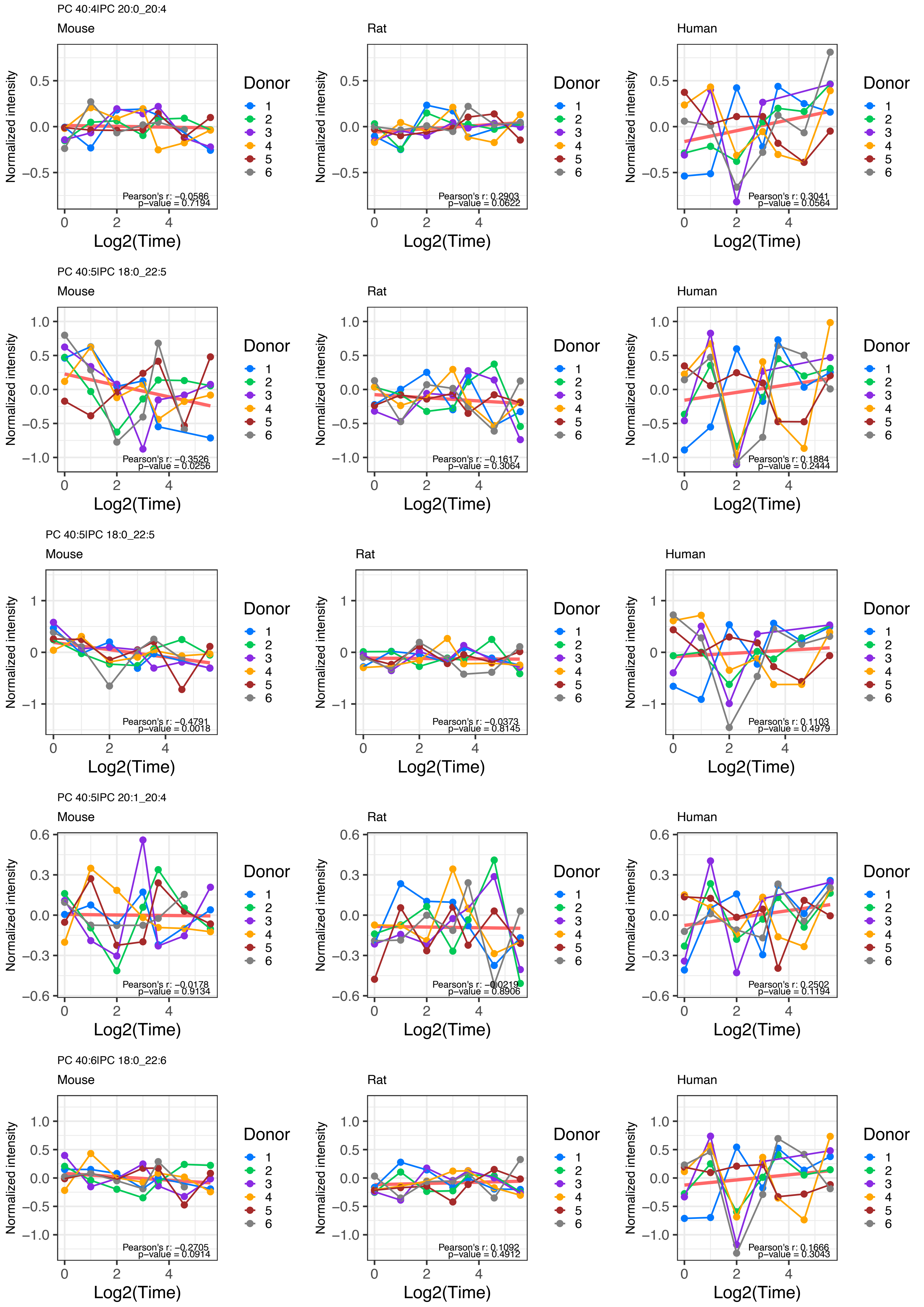
PC 40:4|PC 20:0_20:4
Mouse
Rat
Human
Normalized intensity
Log2(Time)
Donor
1
2
3
4
5
6
Pearson's r: −0.0586
p−value = 0.7194
Pearson's r: 0.2903
p−value = 0.0622
Pearson's r: 0.3041
p−value = 0.0564
PC 40:5|PC 18:0_22:5
Pearson's r: −0.3526
p−value = 0.0256
Pearson's r: −0.1617
p−value = 0.3064
Pearson's r: 0.1884
p−value = 0.2444
PC 40:5|PC 18:0_22:5
Pearson's r: −0.4791
p−value = 0.0018
Pearson's r: −0.0373
p−value = 0.8145
Pearson's r: 0.1103
p−value = 0.4979
PC 40:5|PC 20:1_20:4
Pearson's r: −0.0178
p−value = 0.9134
Pearson's r: −0.0219
p−value = 0.8906
Pearson's r: 0.2502
p−value = 0.1194
PC 40:6|PC 18:0_22:6
Pearson's r: −0.2705
p−value = 0.0914
Pearson's r: 0.1092
p−value = 0.4912
Pearson's r: 0.1666
p−value = 0.3043

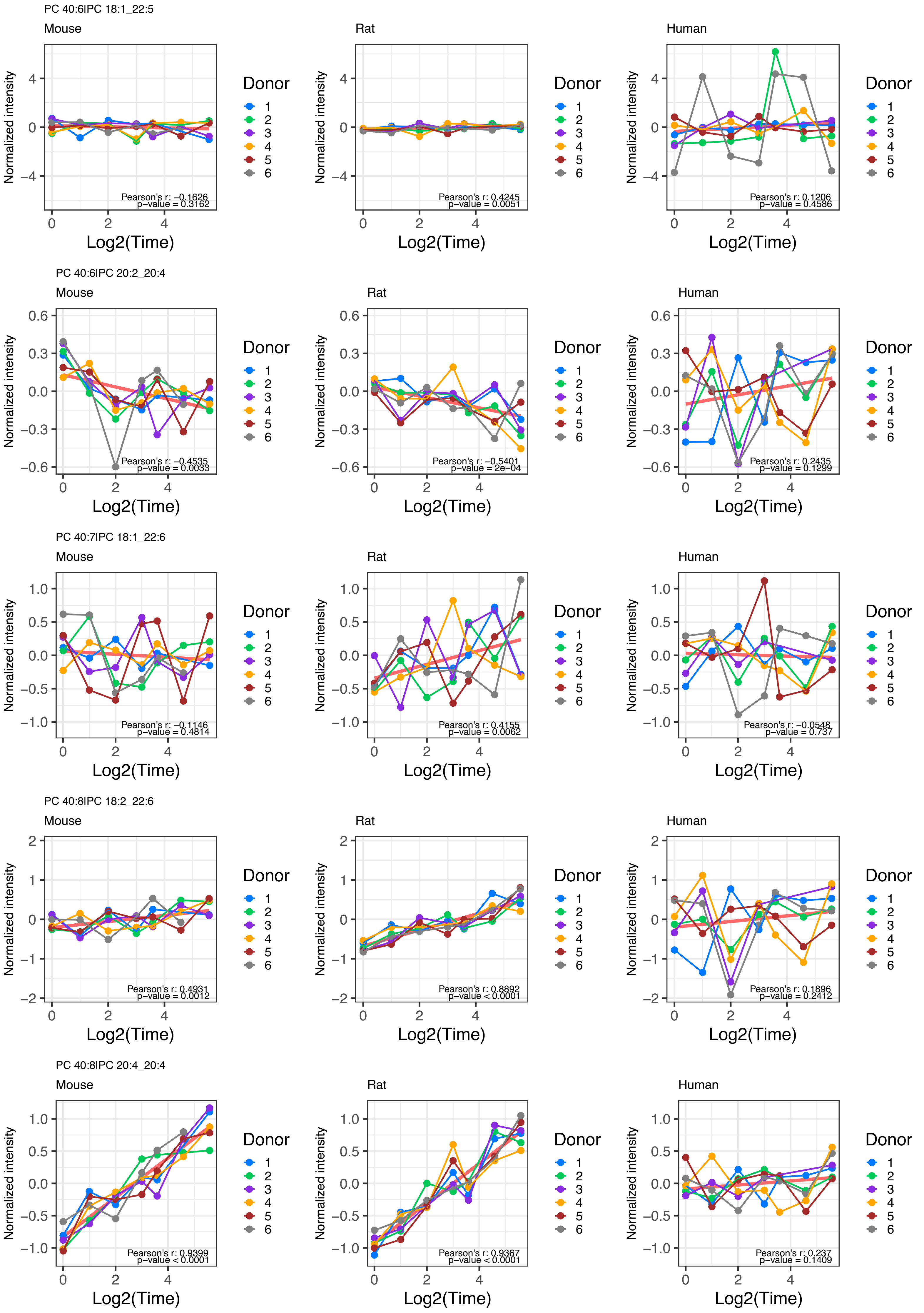

PC 40:6|PC 18:1_22:5
Mouse
Rat
Human
Normalized intensity
Log2(Time)
Donor
1
2
3
4
5
6
Pearson's r: −0.1626
p−value = 0.3162
Pearson's r: 0.4245
p−value = 0.0051
Pearson's r: 0.1206
p−value = 0.4586
PC 40:6|PC 20:2_20:4
Mouse
Rat
Human
Pearson's r: −0.4535
p−value = 0.0033
Pearson's r: −0.5401
p−value = 2e−04
Pearson's r: 0.2435
p−value = 0.1299
PC 40:7|PC 18:1_22:6
Mouse
Rat
Human
Pearson's r: −0.1146
p−value = 0.4814
Pearson's r: 0.4155
p−value = 0.0062
Pearson's r: −0.0548
p−value = 0.737
PC 40:8|PC 18:2_22:6
Mouse
Rat
Human
Pearson's r: 0.4931
p−value = 0.0012
Pearson's r: 0.8892
p−value < 0.0001
Pearson's r: 0.1896
p−value = 0.2412
PC 40:8|PC 20:4_20:4
Mouse
Rat
Human
Pearson's r: 0.9399
p−value < 0.0001
Pearson's r: 0.9367
p−value < 0.0001
Pearson's r: 0.237
p−value = 0.1409

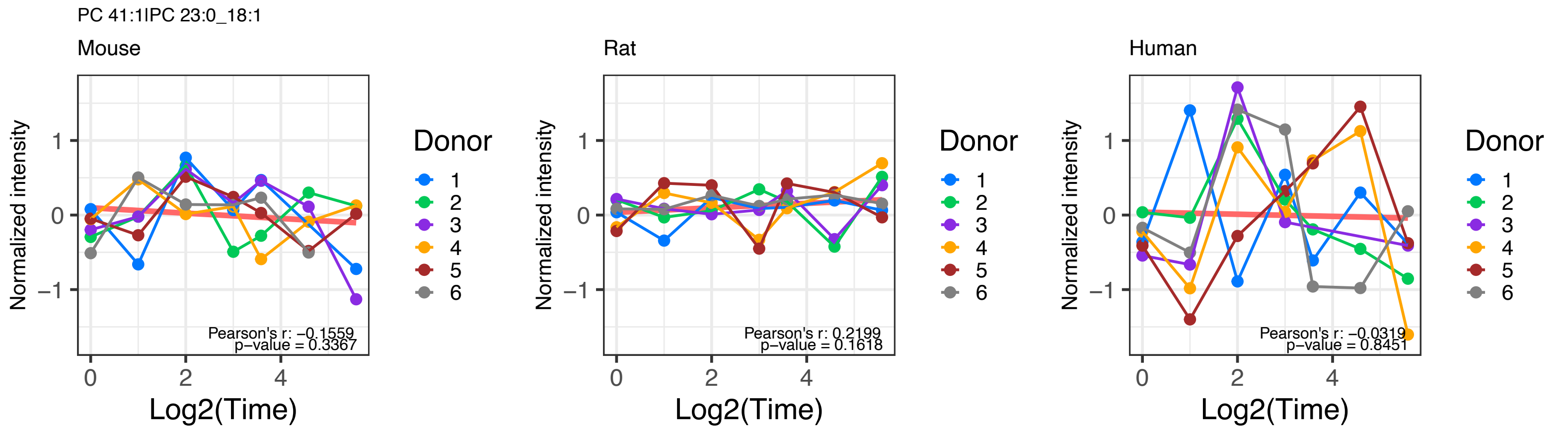
PC 41:1|PC 23:0_18:1
Mouse
Rat
Human
Normalized intensity
Log2(Time)
Donor
Pearson's r: −0.1559
p−value = 0.3367
Pearson's r: 0.2199
p−value = 0.1618
Pearson's r: −0.0319
p−value = 0.8451

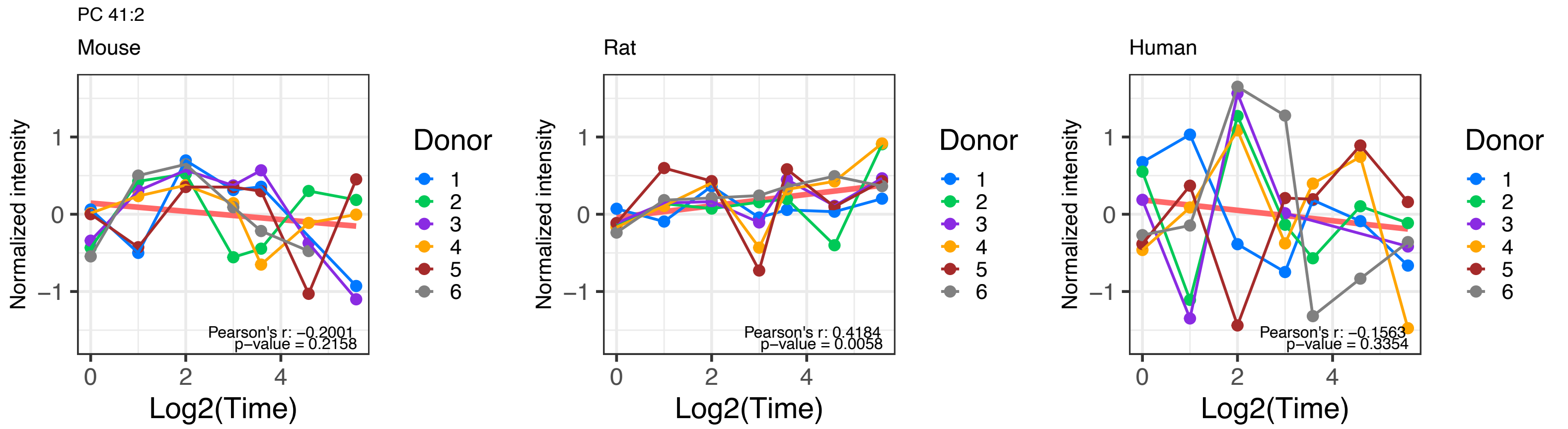
PC 41:2
Mouse
Rat
Human
Normalized intensity
Log2(Time)
Donor
Pearson's r: −0.2001
p−value = 0.2158
Pearson's r: 0.4184
p−value = 0.0058
Pearson's r: −0.1563
p−value = 0.3354

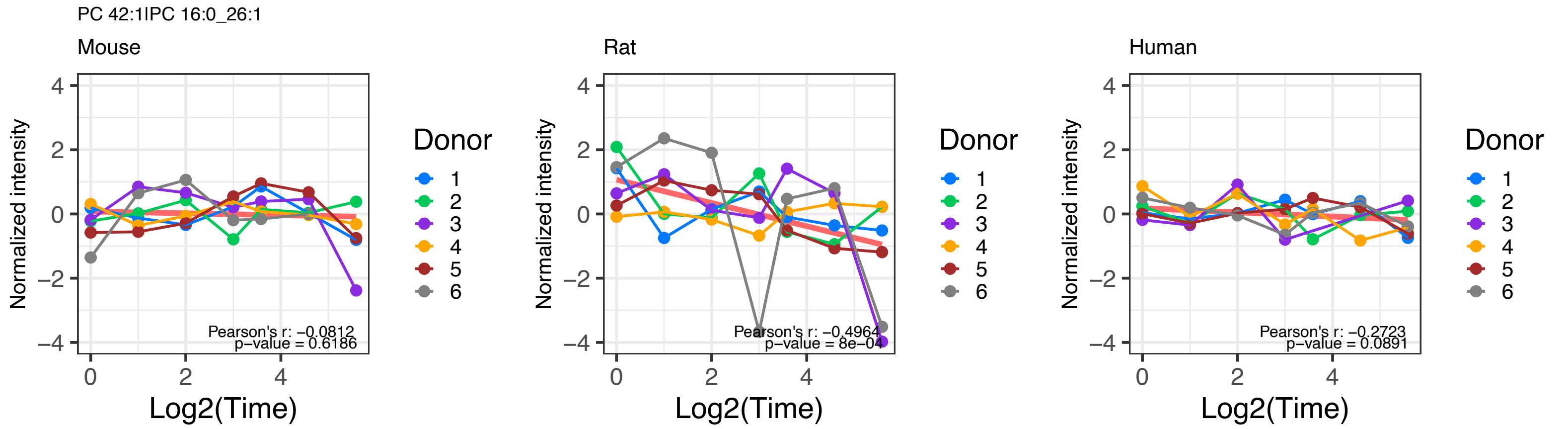
PC 42:1|PC 16:0_26:1
Mouse
Rat
Human
Normalized intensity
Log2(Time)
Donor
Pearson's r: −0.0812
p−value = 0.6186
Pearson's r: −0.4964
p−value = 8e−04
Pearson's r: −0.2723
p−value = 0.0891

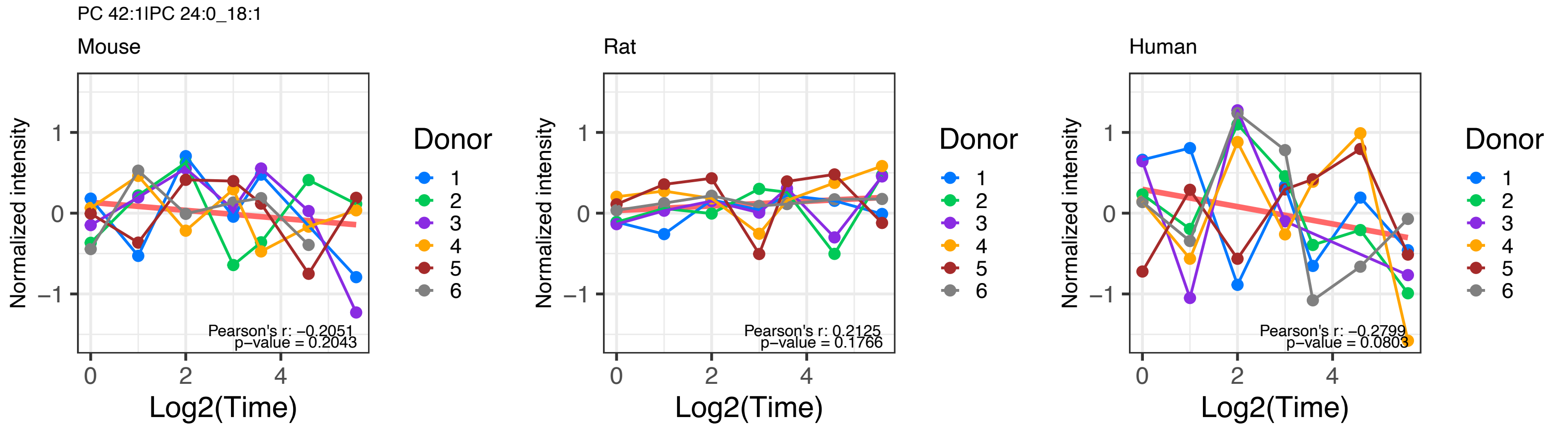
PC 42:1|PC 24:0_18:1
Mouse
Rat
Human
Normalized intensity
Log2(Time)
Donor
Pearson's r: −0.2051
p−value = 0.2043
Pearson's r: 0.2125
p−value = 0.1766
Pearson's r: −0.2799
p−value = 0.0803

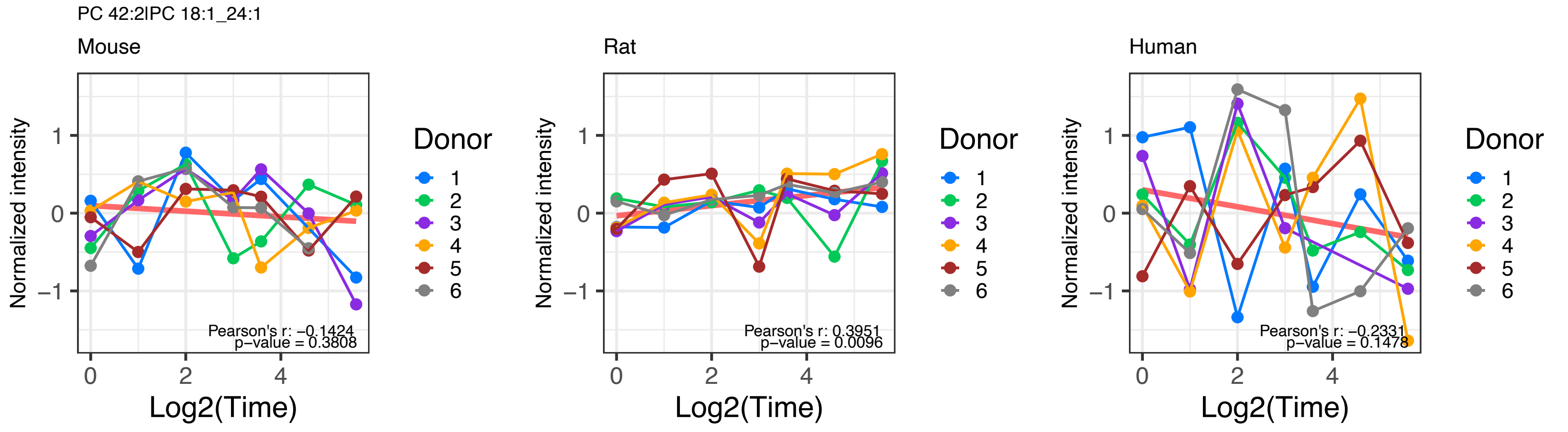
PC 42:2|PC 18:1_24:1
Mouse
Rat
Human
Normalized intensity
Log2(Time)
Donor
Pearson's r: −0.1424
p−value = 0.3808
Pearson's r: 0.3951
p−value = 0.0096
Pearson's r: −0.2331
p−value = 0.1478

PC 42:2|PC 24:0_18:2

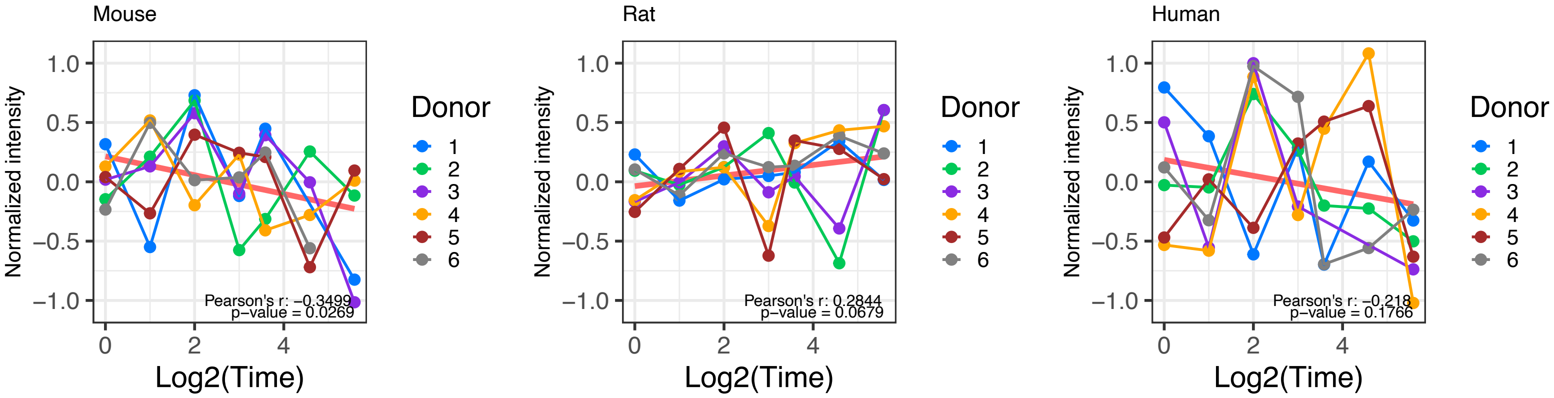

PC 42:3

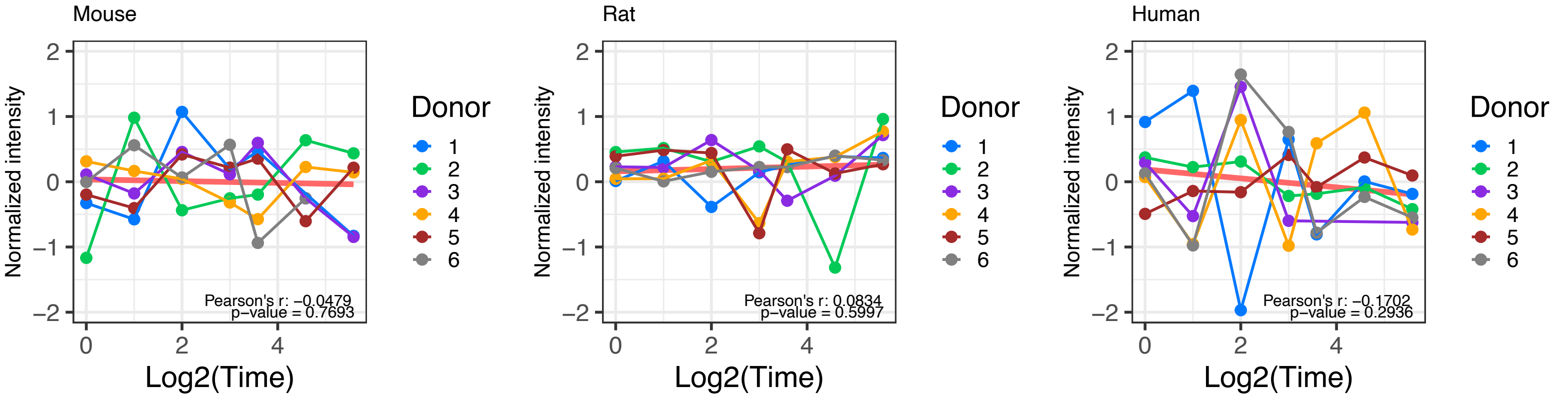

PC 42:4|PC 16:0_26:4

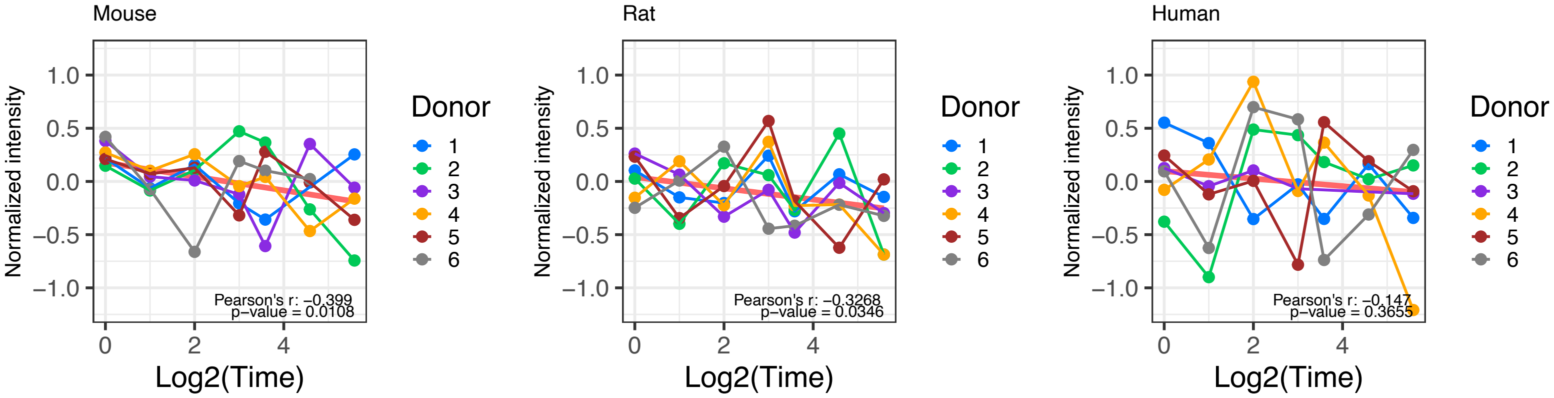

PC 42:4|PC 18:0_24:4

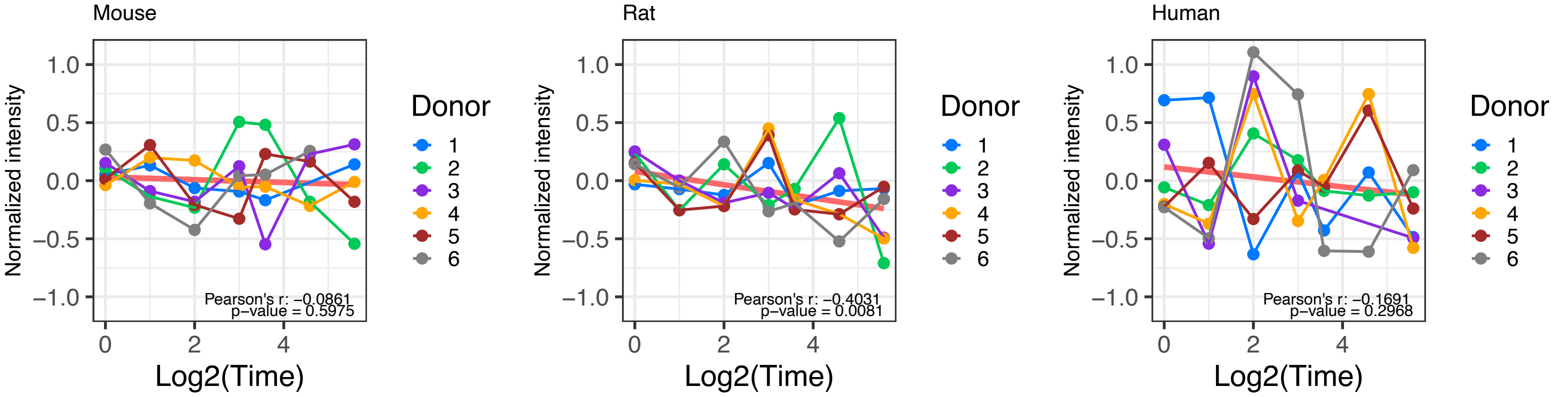

PC 42:4|PC 22:0_20:4

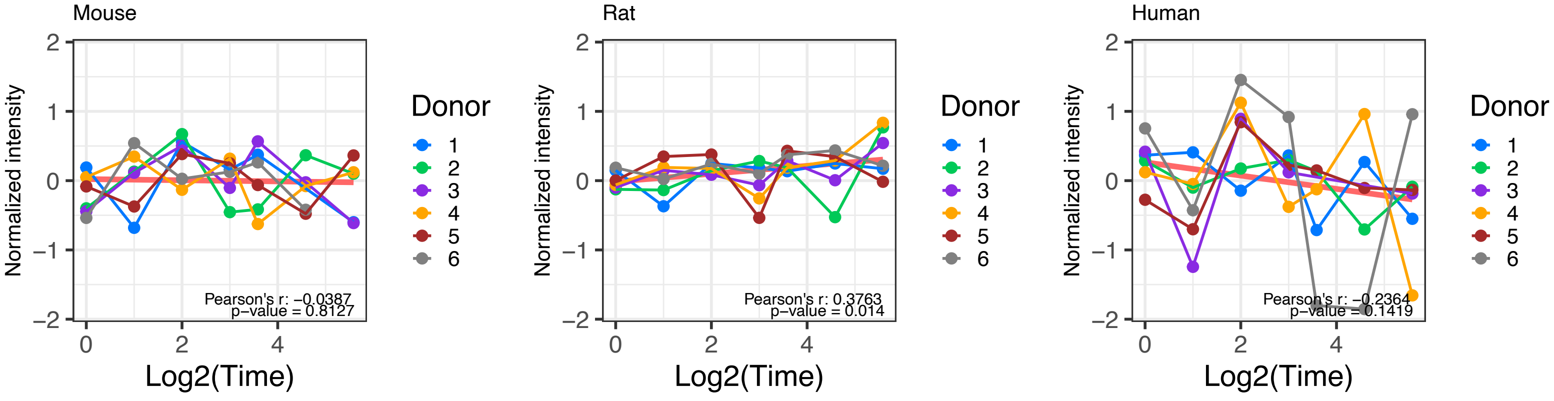

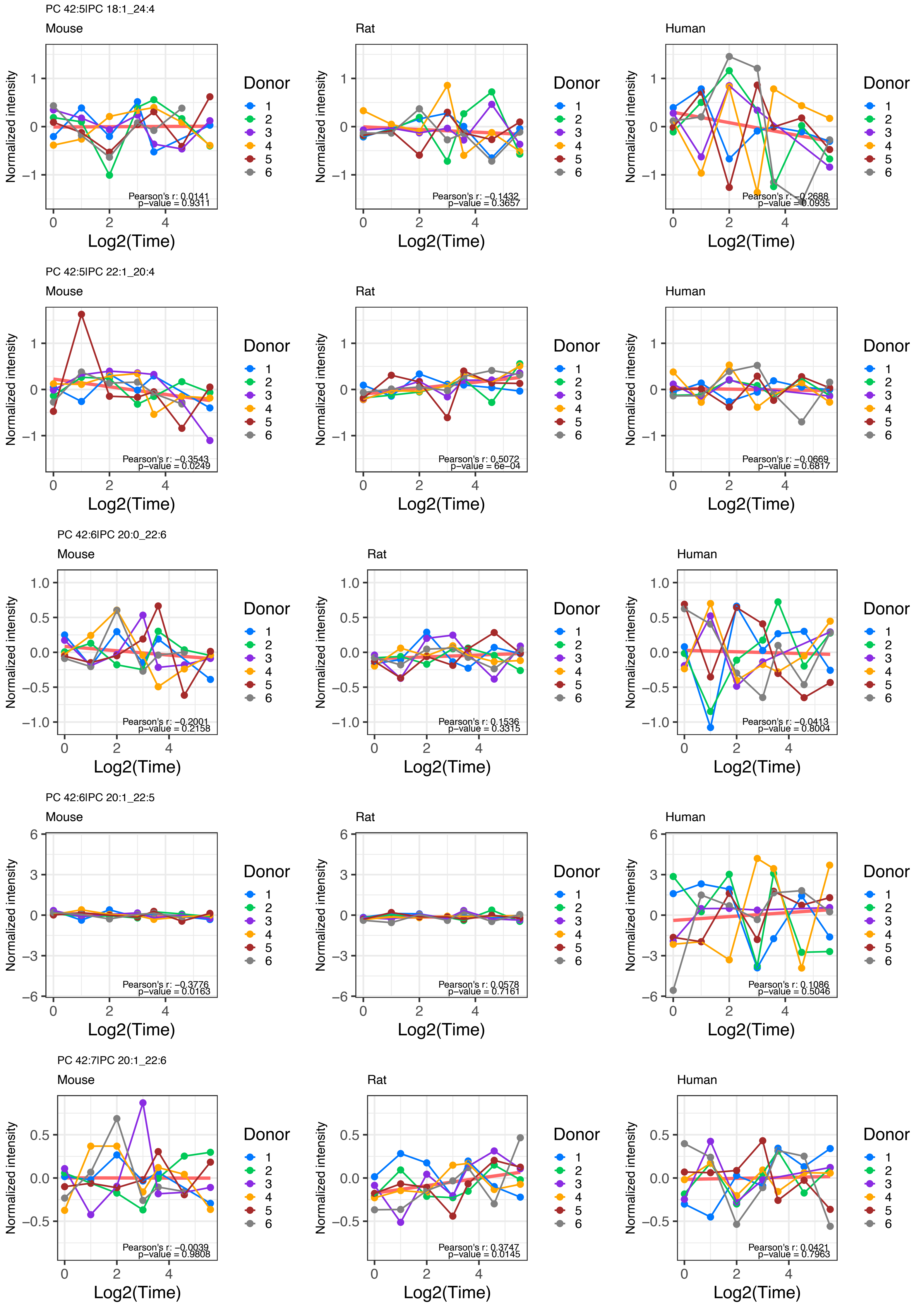
PC 42:5|PC 18:1_24:4
Mouse
Rat
Human
Normalized intensity
Log2(Time)
Donor
1
2
3
4
5
6
Pearson's r: 0.0141
p−value = 0.9311
Pearson's r: −0.1432
p−value = 0.3657
Pearson's r: −0.2688
p−value = 0.0935
PC 42:5|PC 22:1_20:4
Pearson's r: −0.3543
p−value = 0.0249
Pearson's r: 0.5072
p−value = 6e−04
Pearson's r: −0.0669
p−value = 0.6817
PC 42:6|PC 20:0_22:6
Pearson's r: −0.2001
p−value = 0.2158
Pearson's r: 0.1536
p−value = 0.3315
Pearson's r: −0.0413
p−value = 0.8004
PC 42:6|PC 20:1_22:5
Pearson's r: −0.3776
p−value = 0.0163
Pearson's r: 0.0578
p−value = 0.7161
Pearson's r: 0.1086
p−value = 0.5046
PC 42:7|PC 20:1_22:6
Pearson's r: −0.0039
p−value = 0.9808
Pearson's r: 0.3747
p−value = 0.0145
Pearson's r: 0.0421
p−value = 0.7963

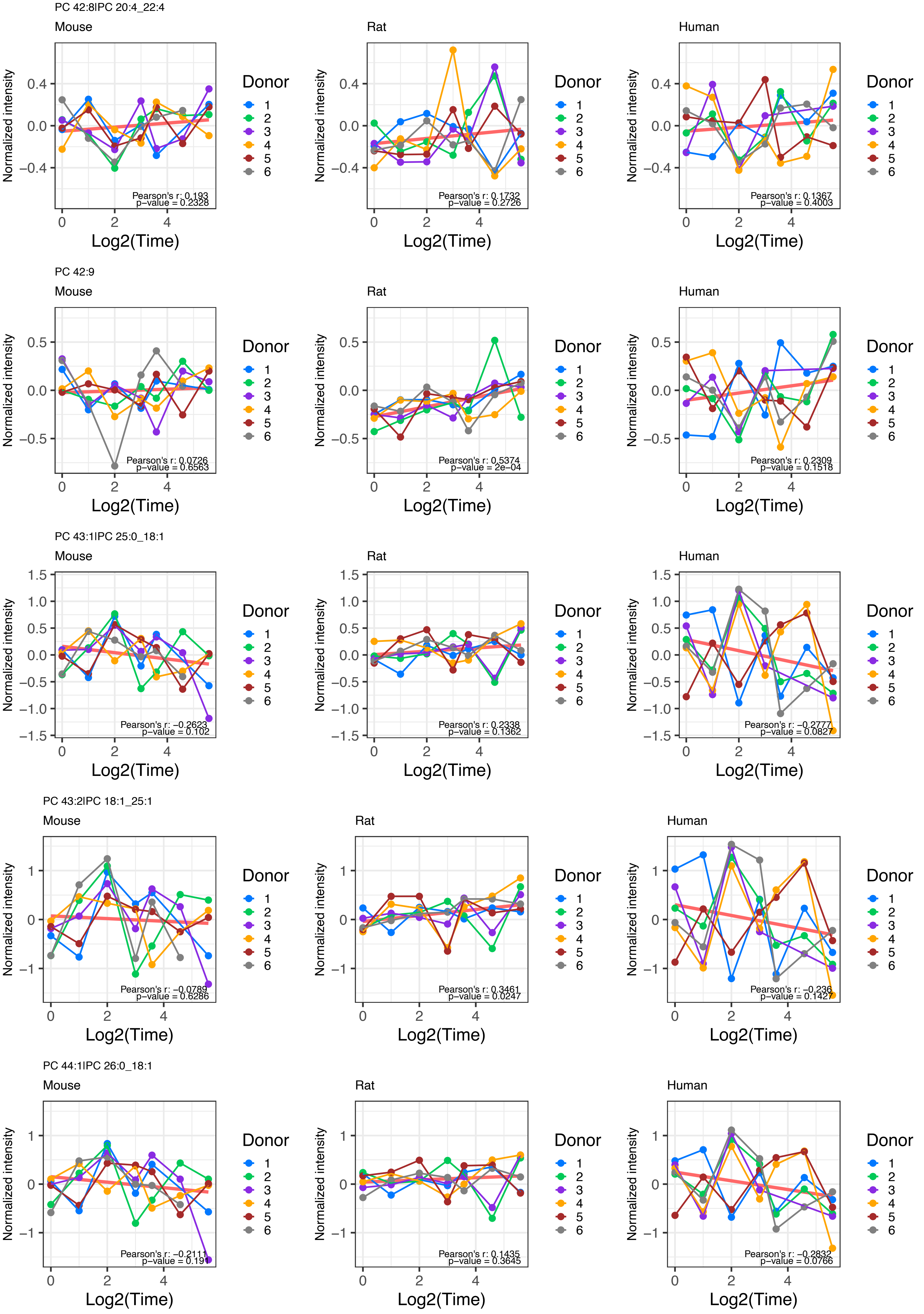
PC 42:8|PC 20:4_22:4
Mouse
Pearson's r: 0.193
p−value = 0.2328
Rat
Pearson's r: 0.1732
p−value = 0.2726
Human
Pearson's r: 0.1367
p−value = 0.4003
PC 42:9
Mouse
Pearson's r: 0.0726
p−value = 0.6563
Rat
Pearson's r: 0.5374
p−value = 2e−04
Human
Pearson's r: 0.2309
p−value = 0.1518
PC 43:1|PC 25:0_18:1
Mouse
Pearson's r: −0.2623
p−value = 0.102
Rat
Pearson's r: 0.2338
p−value = 0.1362
Human
Pearson's r: −0.2777
p−value = 0.0827
PC 43:2|PC 18:1_25:1
Mouse
Pearson's r: −0.0789
p−value = 0.6286
Rat
Pearson's r: 0.3461
p−value = 0.0247
Human
Pearson's r: −0.236
p−value = 0.1427
PC 44:1|PC 26:0_18:1
Mouse
Pearson's r: −0.2111
p−value = 0.191
Rat
Pearson's r: 0.1435
p−value = 0.3645
Human
Pearson's r: −0.2832
p−value = 0.0766
Normalized intensity
Log2(Time)
Donor
1
2
3
4
5
6

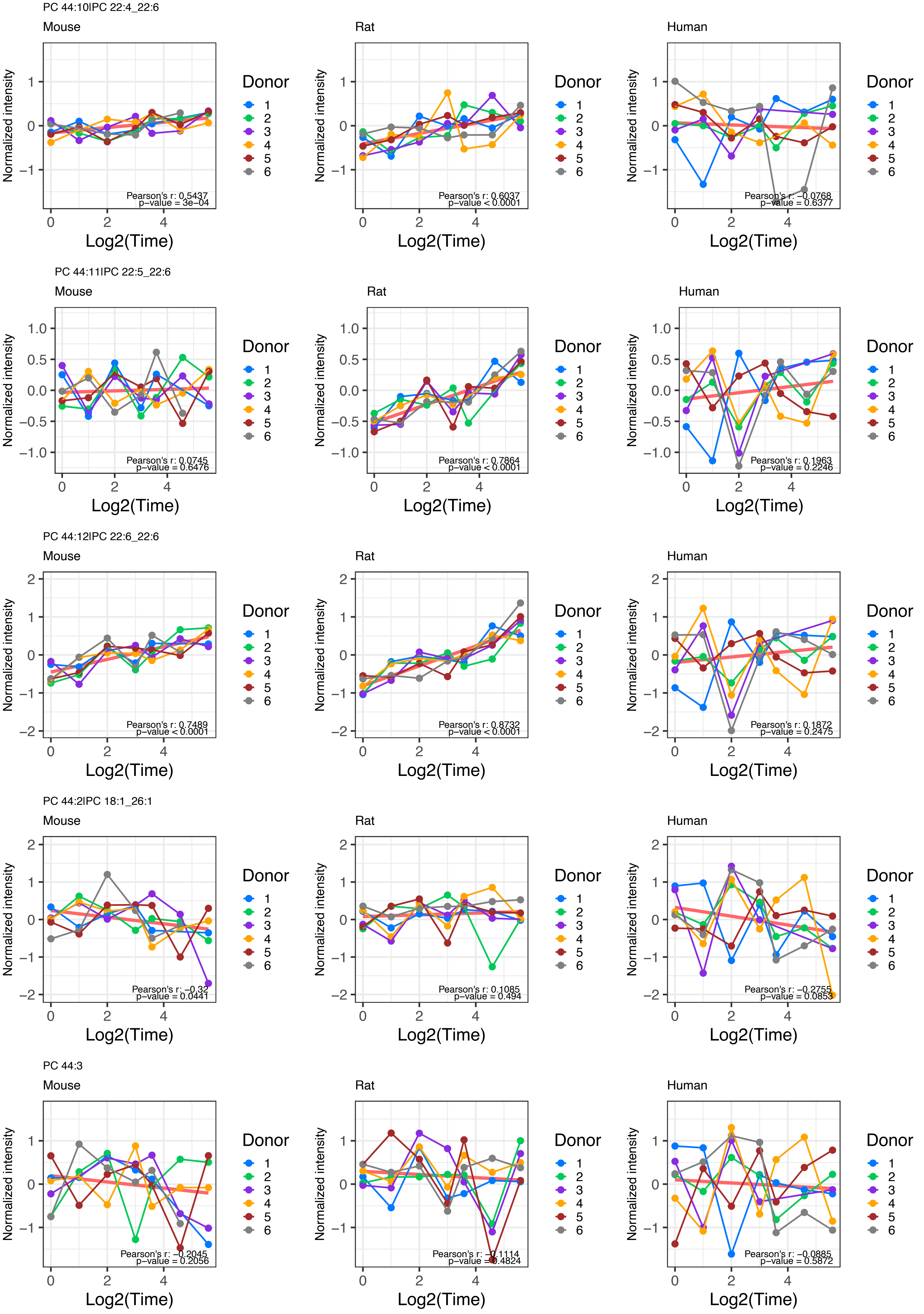

PC 44:10|PC 22:4_22:6
Mouse
Pearson's r: 0.5437
p−value = 3e−04
Rat
Pearson's r: 0.6037
p−value < 0.0001
Human
Pearson's r: −0.0768
p−value = 0.6377
PC 44:11|PC 22:5_22:6
Mouse
Pearson's r: 0.0745
p−value = 0.6476
Rat
Pearson's r: 0.7864
p−value < 0.0001
Human
Pearson's r: 0.1963
p−value = 0.2246
PC 44:12|PC 22:6_22:6
Mouse
Pearson's r: 0.7489
p−value < 0.0001
Rat
Pearson's r: 0.8732
p−value < 0.0001
Human
Pearson's r: 0.1872
p−value = 0.2475
PC 44:2|PC 18:1_26:1
Mouse
Pearson's r: −0.32
p−value = 0.0441
Rat
Pearson's r: 0.1085
p−value = 0.494
Human
Pearson's r: −0.2755
p−value = 0.0853
PC 44:3
Mouse
Pearson's r: −0.2045
p−value = 0.2056
Rat
Pearson's r: −0.1114
p−value = 0.4824
Human
Pearson's r: −0.0885
p−value = 0.5872
Normalized intensity
Log2(Time)
Donor
1
2
3
4
5
6

PC 44:4

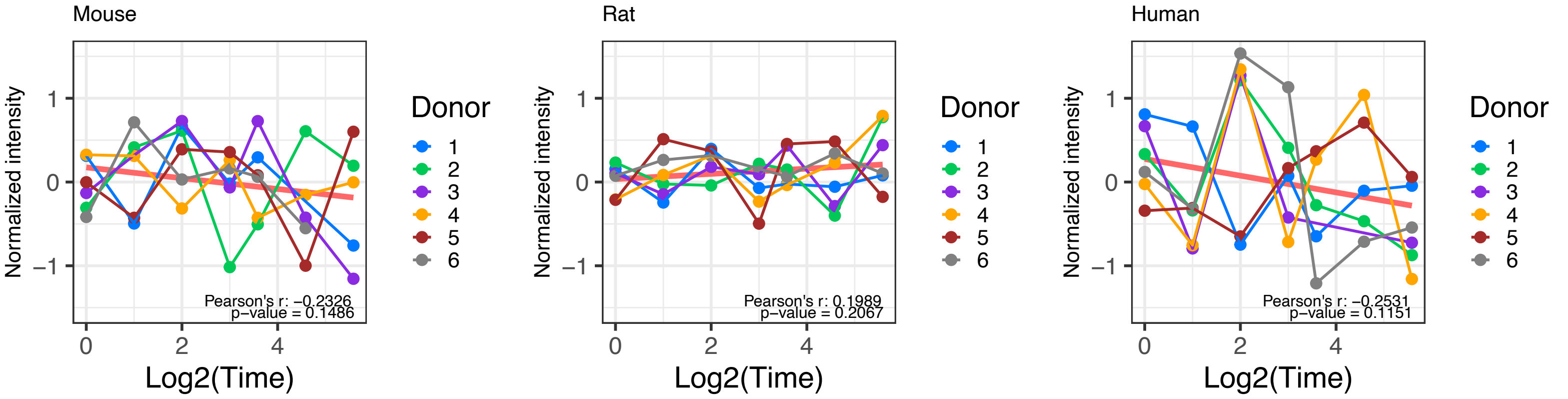

PC 44:5

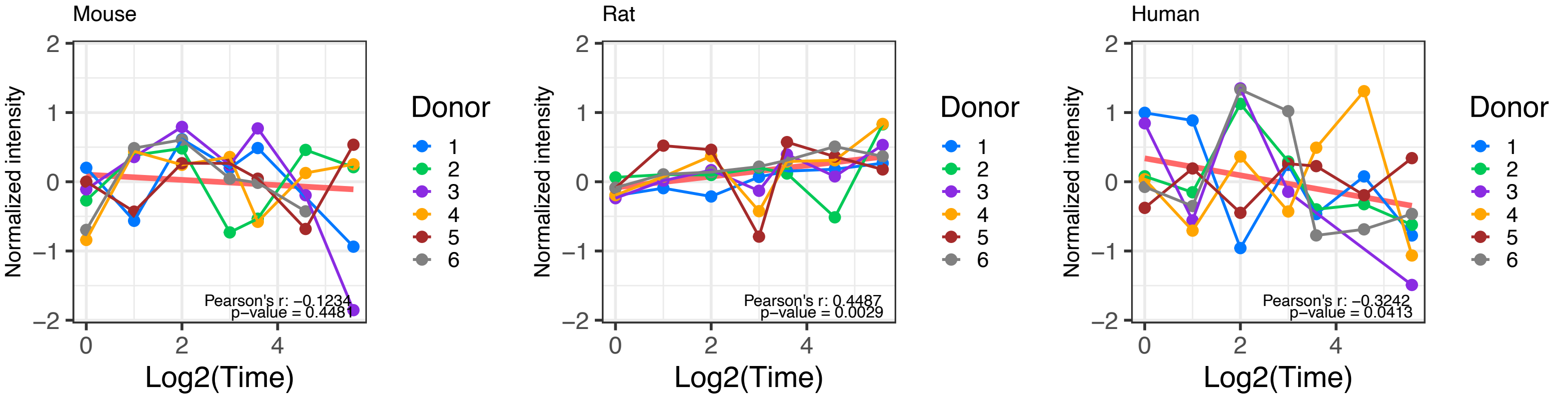

PC 44:5

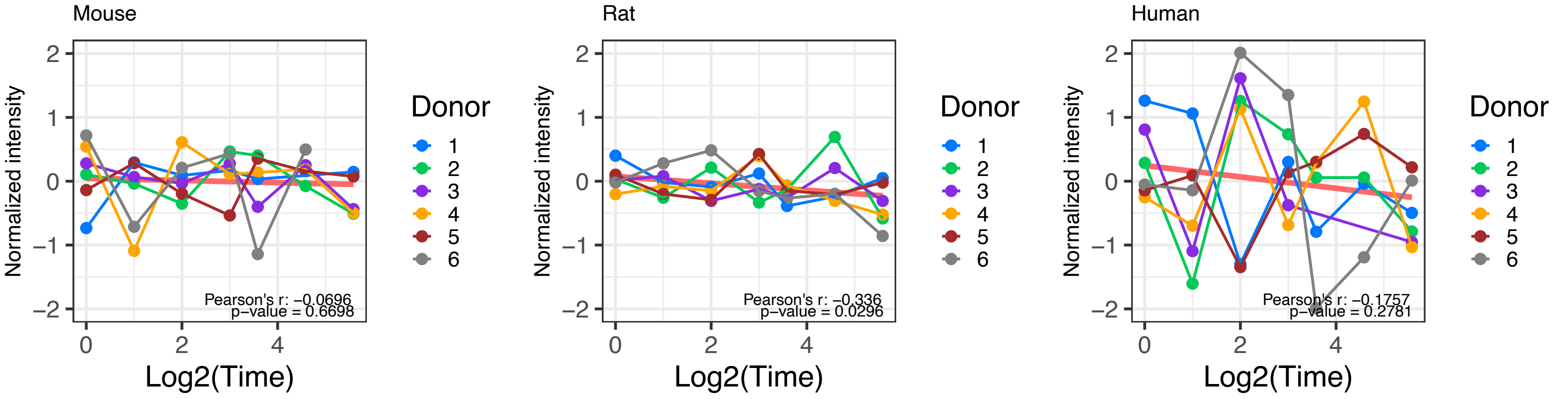

PC 44:7

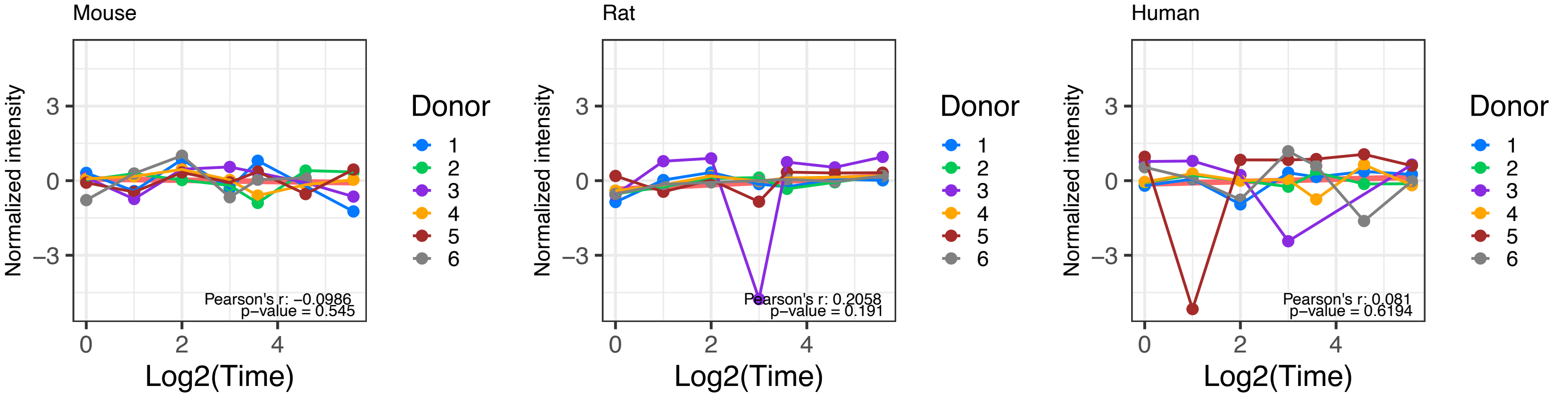

PC 44:8|PC 20:4_24:4

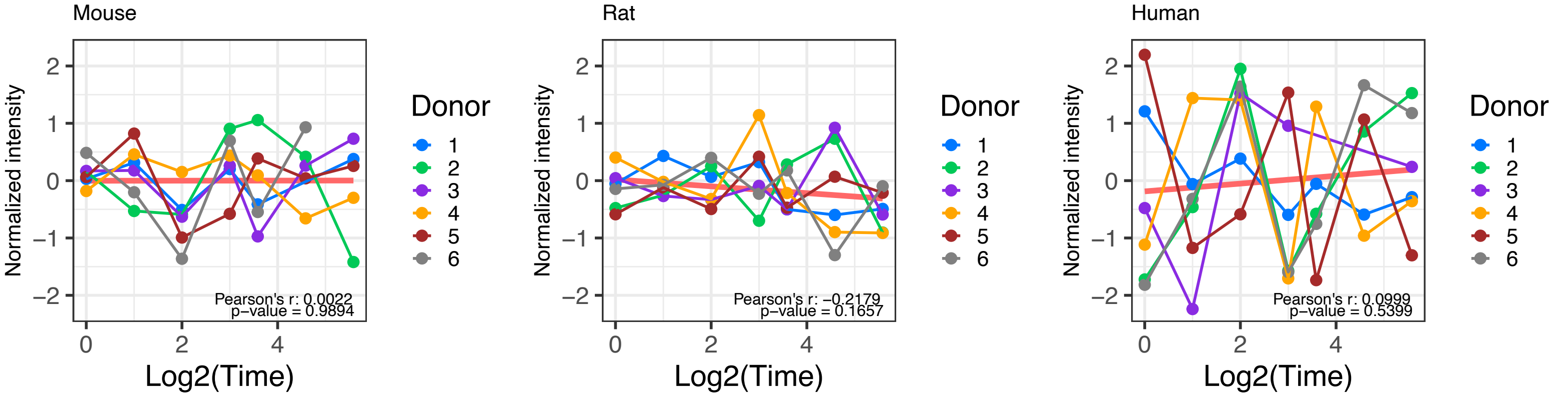

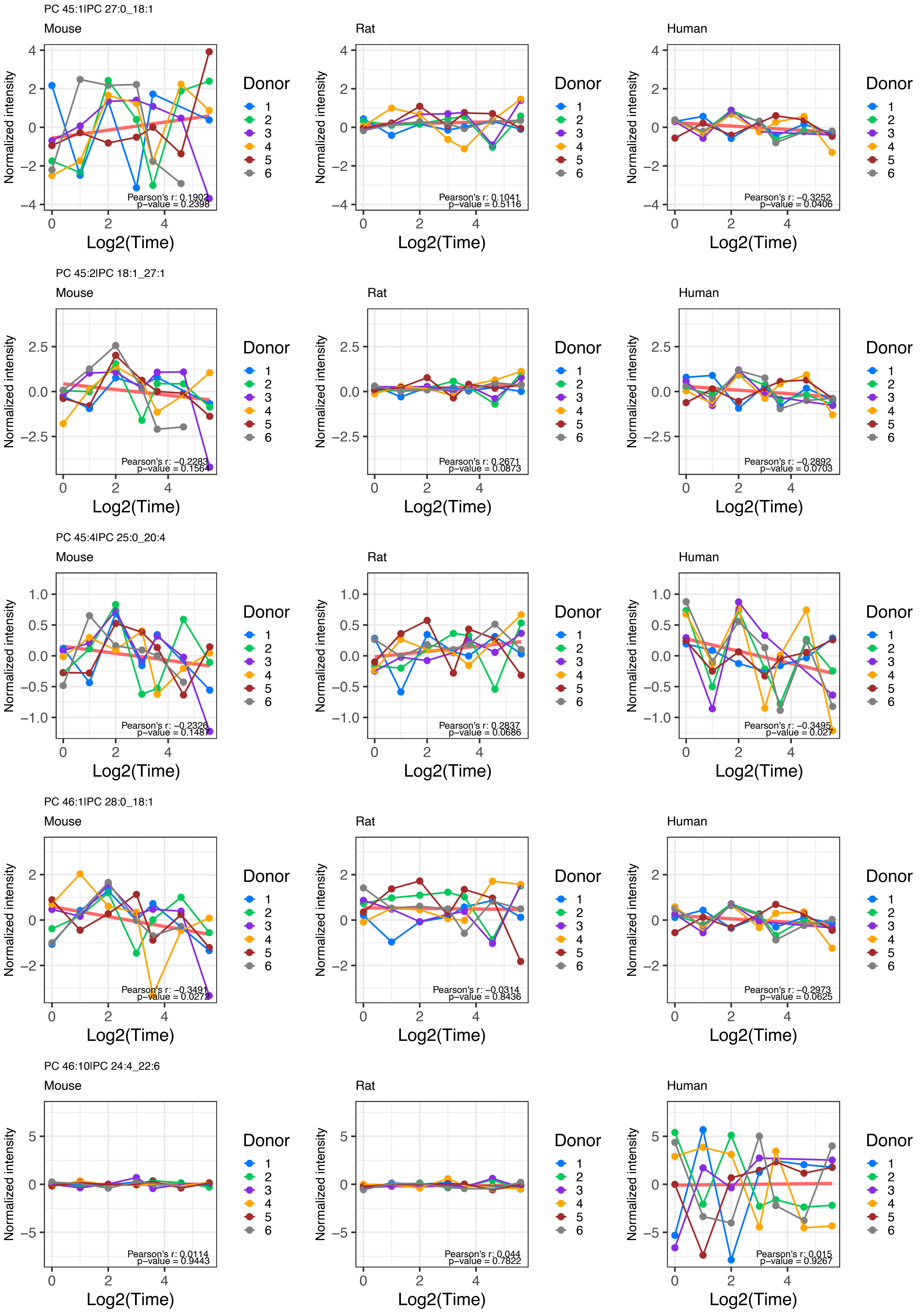
PC 45:1|PC 27:0_18:1
Mouse
Rat
Human
Normalized intensity
Log2(Time)
Donor
1
2
3
4
5
6
Pearson's r: 0.1902
p−value = 0.2398
Pearson's r: 0.1041
p−value = 0.5116
Pearson's r: −0.3252
p−value = 0.0406
PC 45:2|PC 18:1_27:1
Pearson's r: −0.2283
p−value = 0.1564
Pearson's r: 0.2671
p−value = 0.0873
Pearson's r: −0.2892
p−value = 0.0703
PC 45:4|PC 25:0_20:4
Pearson's r: −0.2326
p−value = 0.1487
Pearson's r: 0.2837
p−value = 0.0686
Pearson's r: −0.3495
p−value = 0.027
PC 46:1|PC 28:0_18:1
Pearson's r: −0.3491
p−value = 0.0272
Pearson's r: −0.0314
p−value = 0.8436
Pearson's r: −0.2973
p−value = 0.0625
PC 46:10|PC 24:4_22:6
Pearson's r: 0.0114
p−value = 0.9443
Pearson's r: 0.044
p−value = 0.7822
Pearson's r: 0.015
p−value = 0.9267

PC 46:2|PC 18:1_28:1

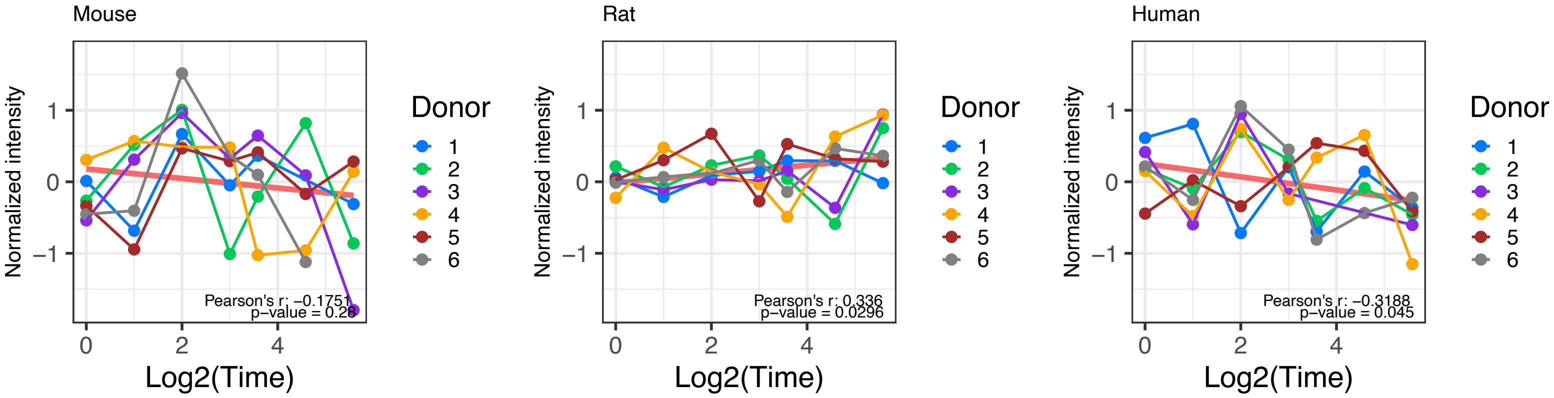

PC 46:4|PC 18:2_28:2

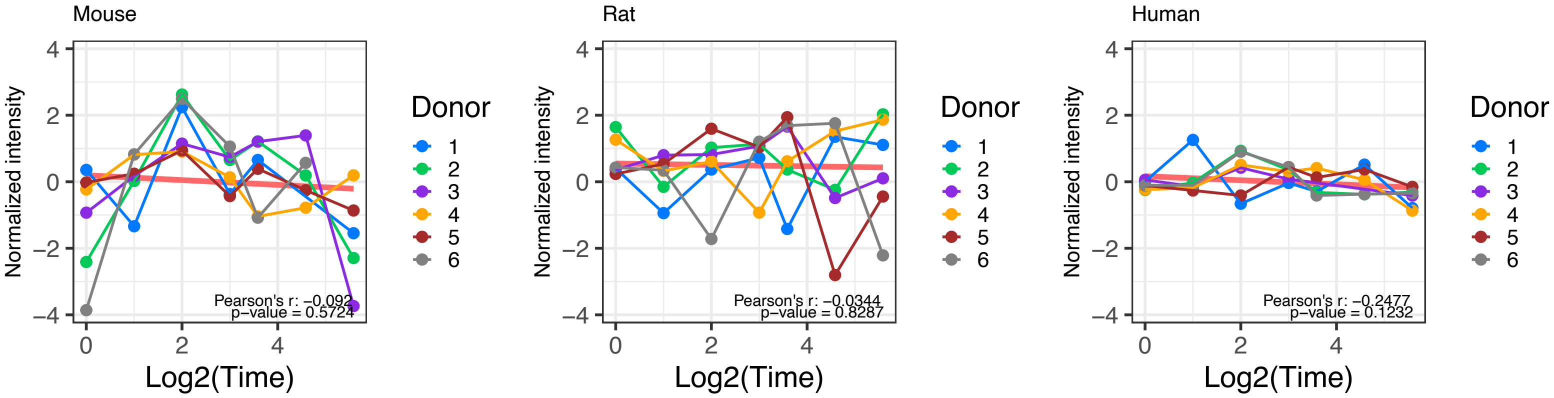

PC 46:4|PC 26:0_20:4

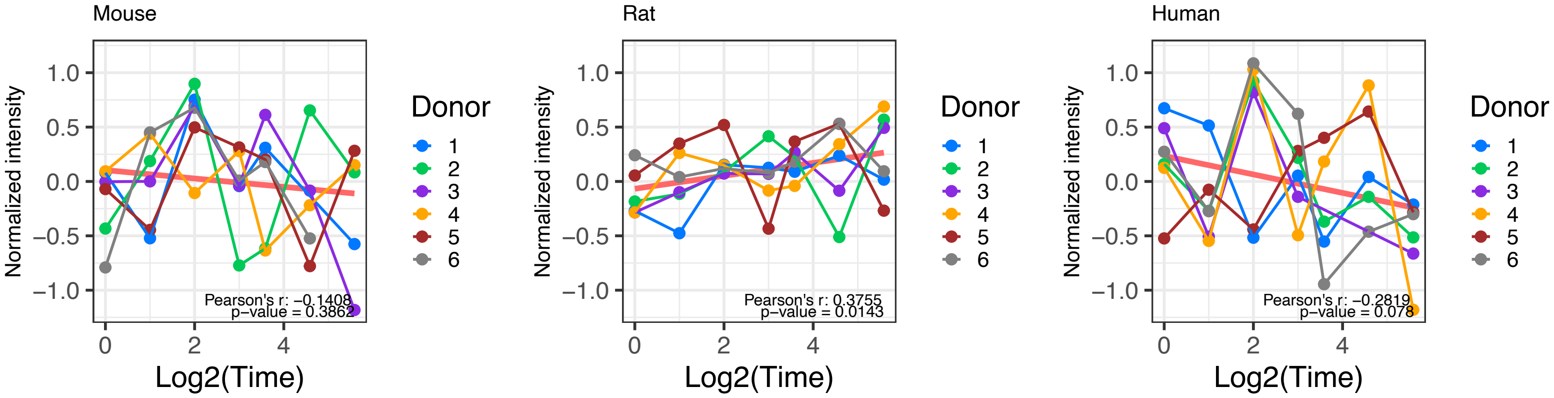

PC 46:5

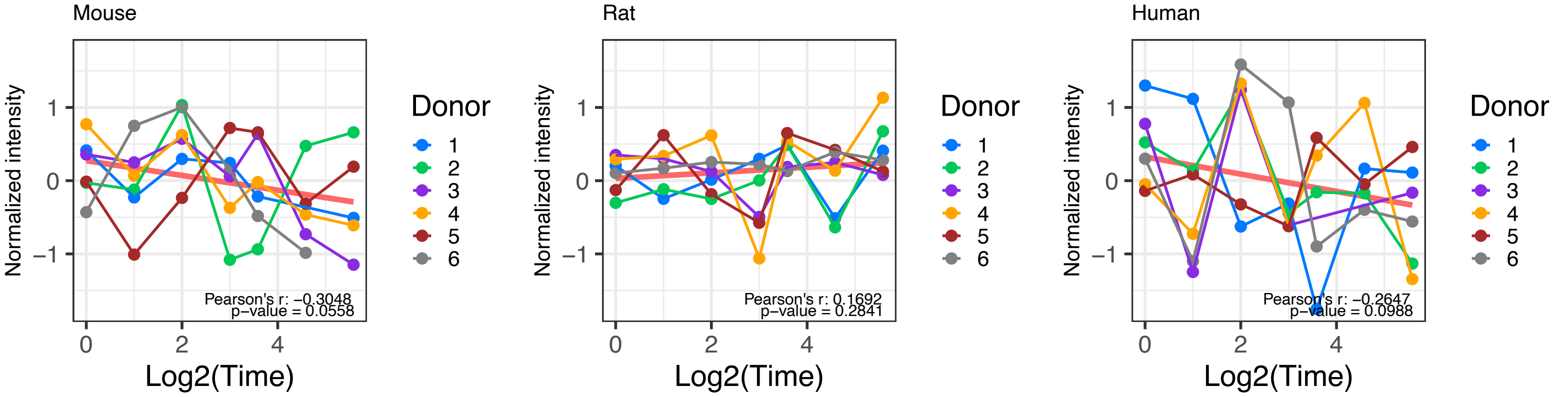

PC 47:2|PC 18:1_29:1

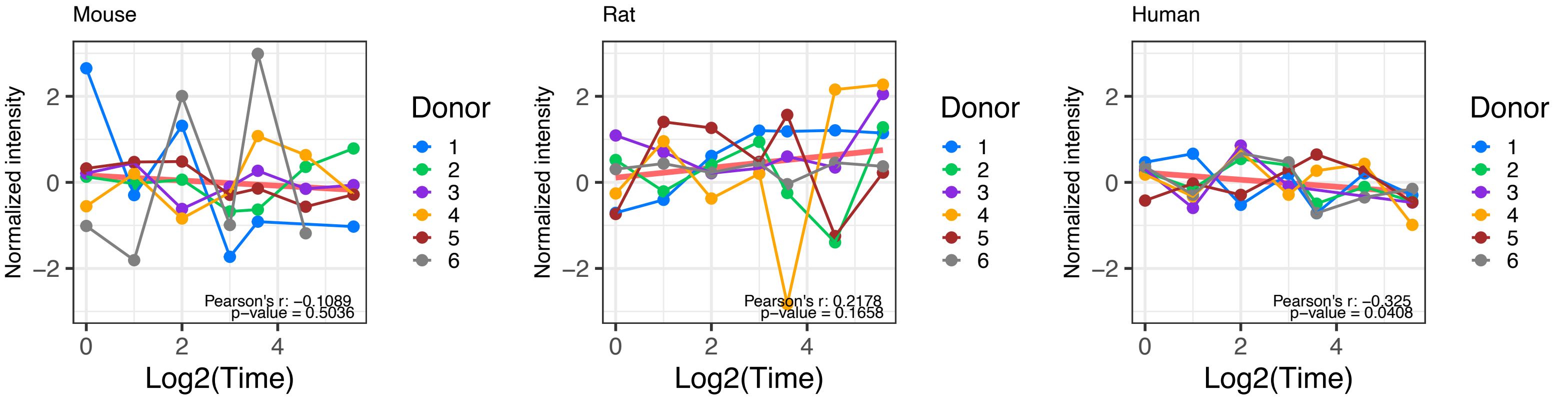

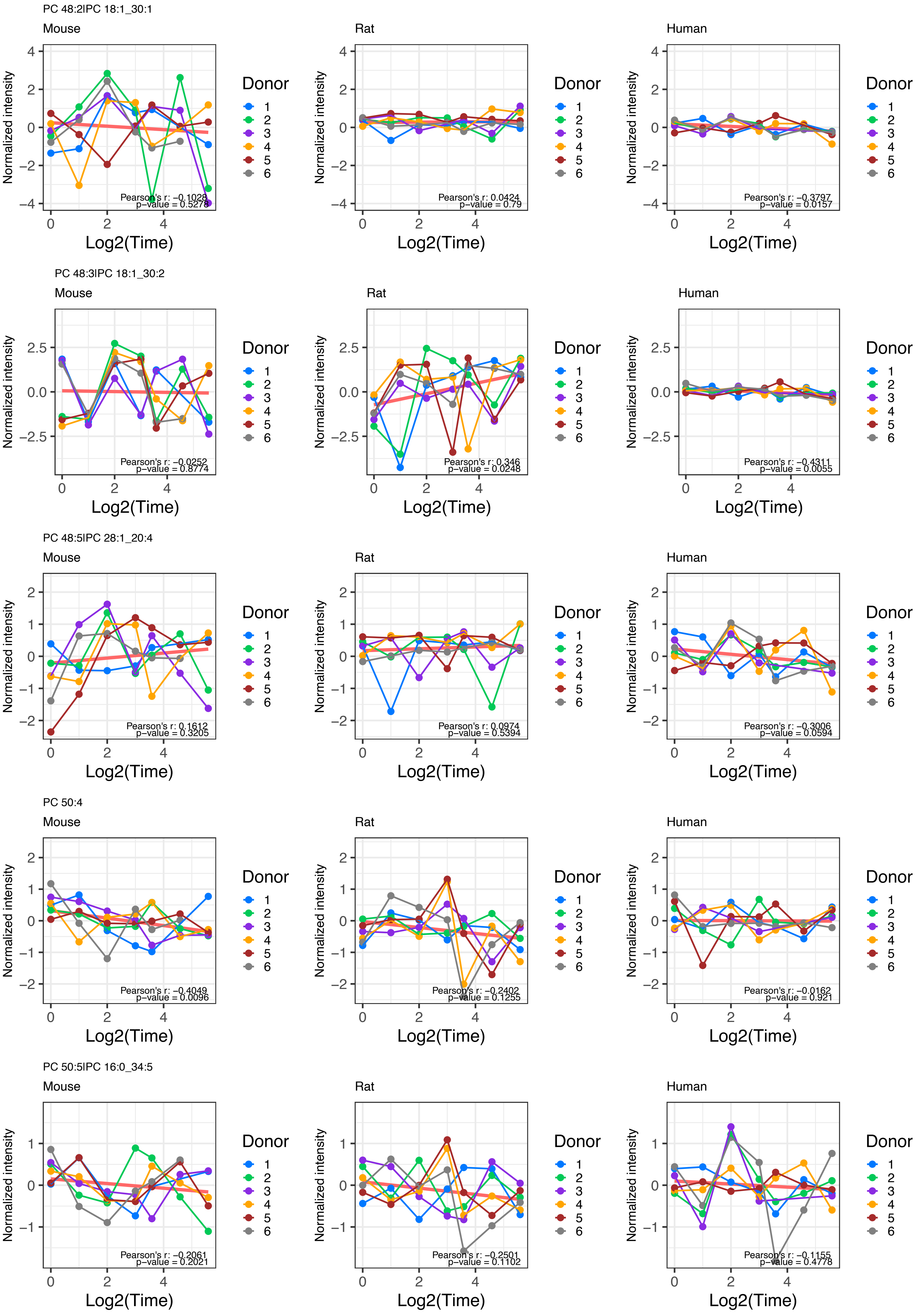
PC 48:2|PC 18:1_30:1
Mouse
Pearson's r: −0.1028
p−value = 0.5278
Rat
Pearson's r: 0.0424
p−value = 0.79
Human
Pearson's r: −0.3797
p−value = 0.0157
PC 48:3|PC 18:1_30:2
Mouse
Pearson's r: −0.0252
p−value = 0.8774
Rat
Pearson's r: 0.346
p−value = 0.0248
Human
Pearson's r: −0.4311
p−value = 0.0055
PC 48:5|PC 28:1_20:4
Mouse
Pearson's r: 0.1612
p−value = 0.3205
Rat
Pearson's r: 0.0974
p−value = 0.5394
Human
Pearson's r: −0.3006
p−value = 0.0594
PC 50:4
Mouse
Pearson's r: −0.4049
p−value = 0.0096
Rat
Pearson's r: −0.2402
p−value = 0.1255
Human
Pearson's r: −0.0162
p−value = 0.921
PC 50:5|PC 16:0_34:5
Mouse
Pearson's r: −0.2061
p−value = 0.2021
Rat
Pearson's r: −0.2501
p−value = 0.1102
Human
Pearson's r: −0.1155
p−value = 0.4778
Normalized intensity
Log2(Time)
Donor
1
2
3
4
5
6

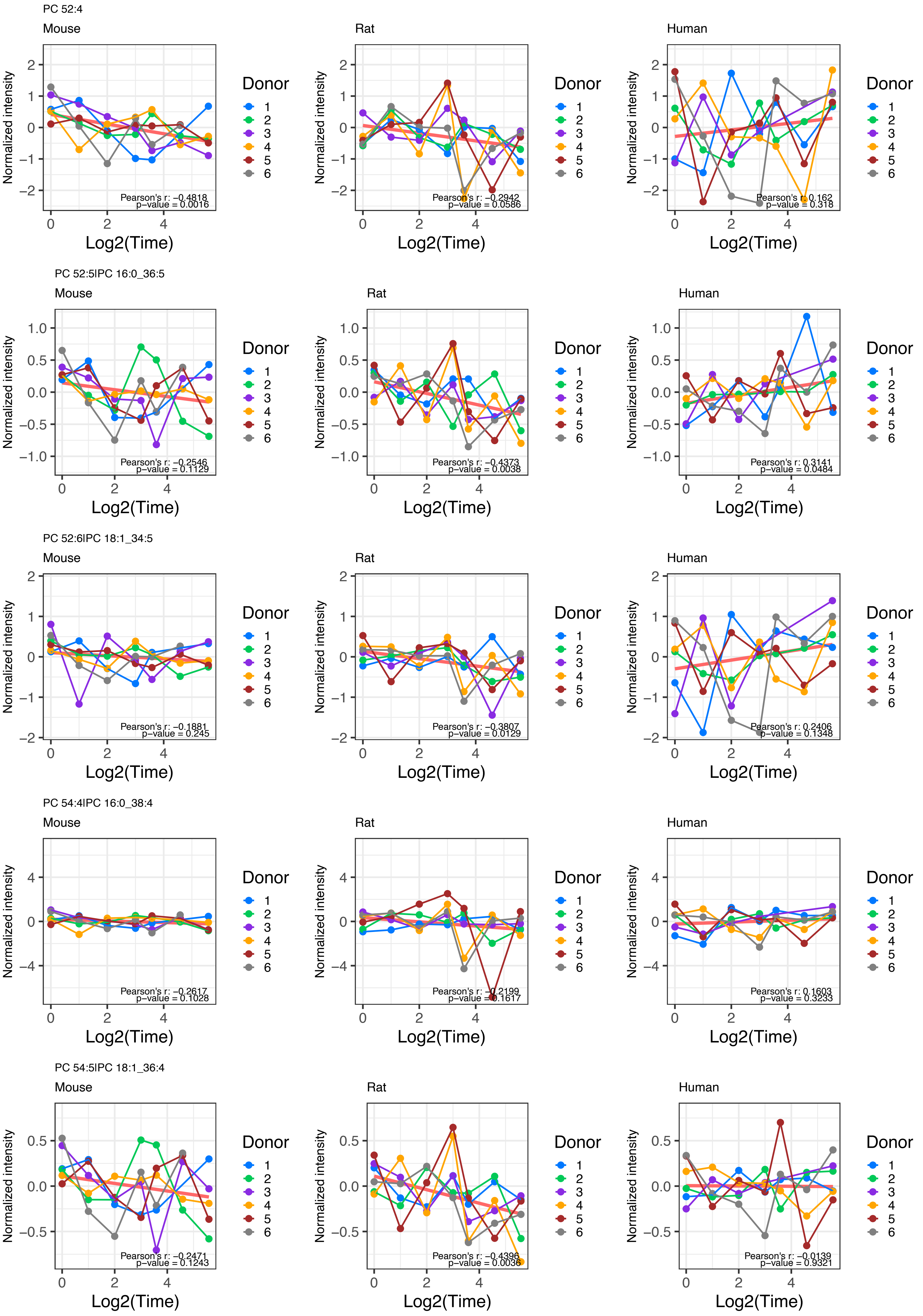
PC 52:4
Mouse
Pearson's r: −0.4818
p−value = 0.0016
Rat
Pearson's r: −0.2942
p−value = 0.0586
Human
Pearson's r: 0.162
p−value = 0.318
PC 52:5|PC 16:0_36:5
Mouse
Pearson's r: −0.2546
p−value = 0.1129
Rat
Pearson's r: −0.4373
p−value = 0.0038
Human
Pearson's r: 0.3141
p−value = 0.0484
PC 52:6|PC 18:1_34:5
Mouse
Pearson's r: −0.1881
p−value = 0.245
Rat
Pearson's r: −0.3807
p−value = 0.0129
Human
Pearson's r: 0.2406
p−value = 0.1348
PC 54:4|PC 16:0_38:4
Mouse
Pearson's r: −0.2617
p−value = 0.1028
Rat
Pearson's r: −0.2199
p−value = 0.1617
Human
Pearson's r: 0.1603
p−value = 0.3233
PC 54:5|PC 18:1_36:4
Mouse
Pearson's r: −0.2471
p−value = 0.1243
Rat
Pearson's r: −0.4396
p−value = 0.0036
Human
Pearson's r: −0.0139
p−value = 0.9321
Normalized intensity
Log2(Time)
Donor
1
2
3
4
5
6

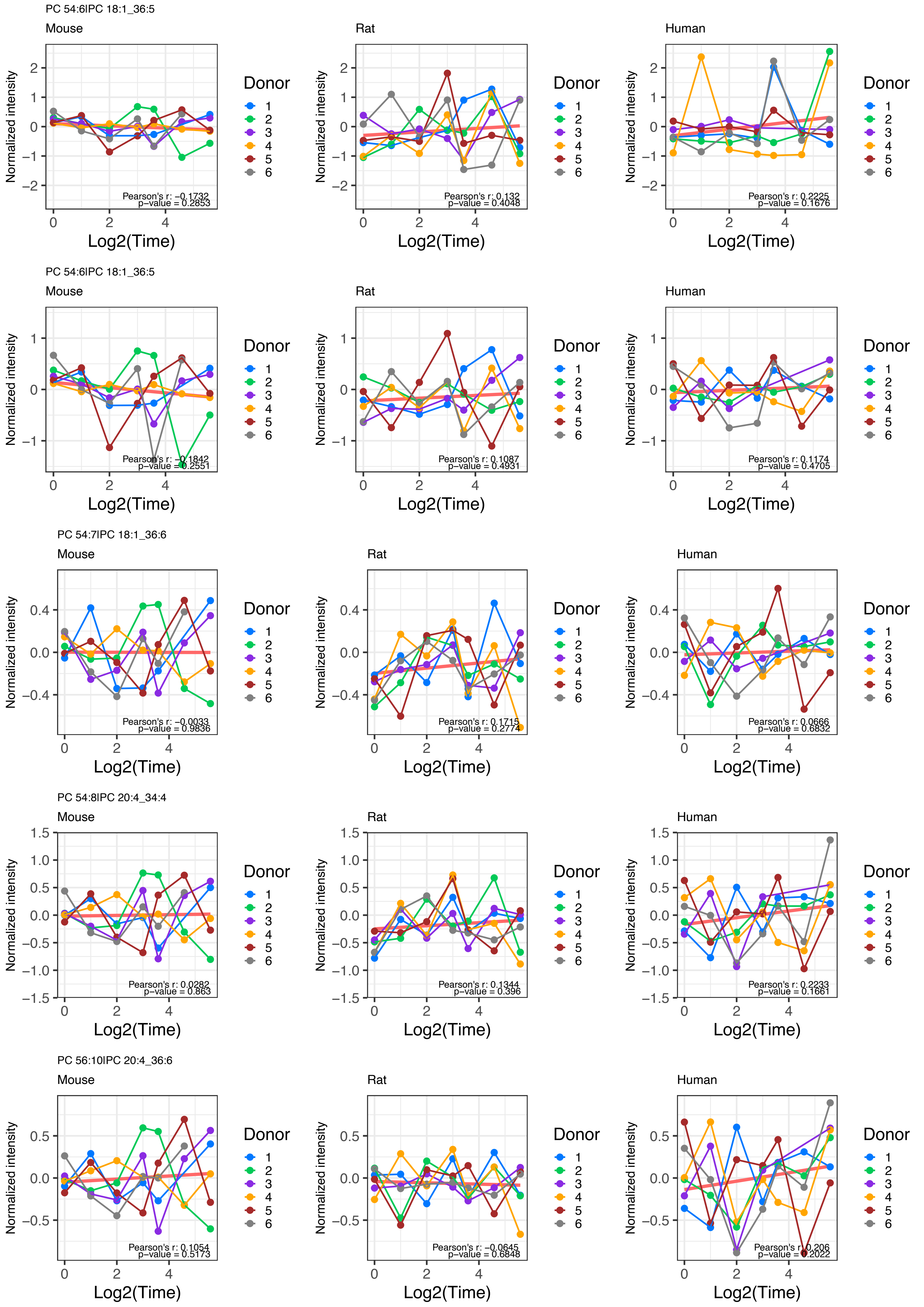
PC 54:6|PC 18:1_36:5
Mouse
Pearson's r: −0.1732
p−value = 0.2853
Rat
Pearson's r: 0.132
p−value = 0.4048
Human
Pearson's r: 0.2225
p−value = 0.1676
PC 54:6|PC 18:1_36:5
Mouse
Pearson's r: −0.1842
p−value = 0.2551
Rat
Pearson's r: 0.1087
p−value = 0.4931
Human
Pearson's r: 0.1174
p−value = 0.4705
PC 54:7|PC 18:1_36:6
Mouse
Pearson's r: −0.0033
p−value = 0.9836
Rat
Pearson's r: 0.1715
p−value = 0.2774
Human
Pearson's r: 0.0666
p−value = 0.6832
PC 54:8|PC 20:4_34:4
Mouse
Pearson's r: 0.0282
p−value = 0.863
Rat
Pearson's r: 0.1344
p−value = 0.396
Human
Pearson's r: 0.2233
p−value = 0.1661
PC 56:10|PC 20:4_36:6
Mouse
Pearson's r: 0.1054
p−value = 0.5173
Rat
Pearson's r: −0.0645
p−value = 0.6848
Human
Pearson's r: 0.206
p−value = 0.2022
Normalized intensity
Log2(Time)
Donor
1
2
3
4
5
6

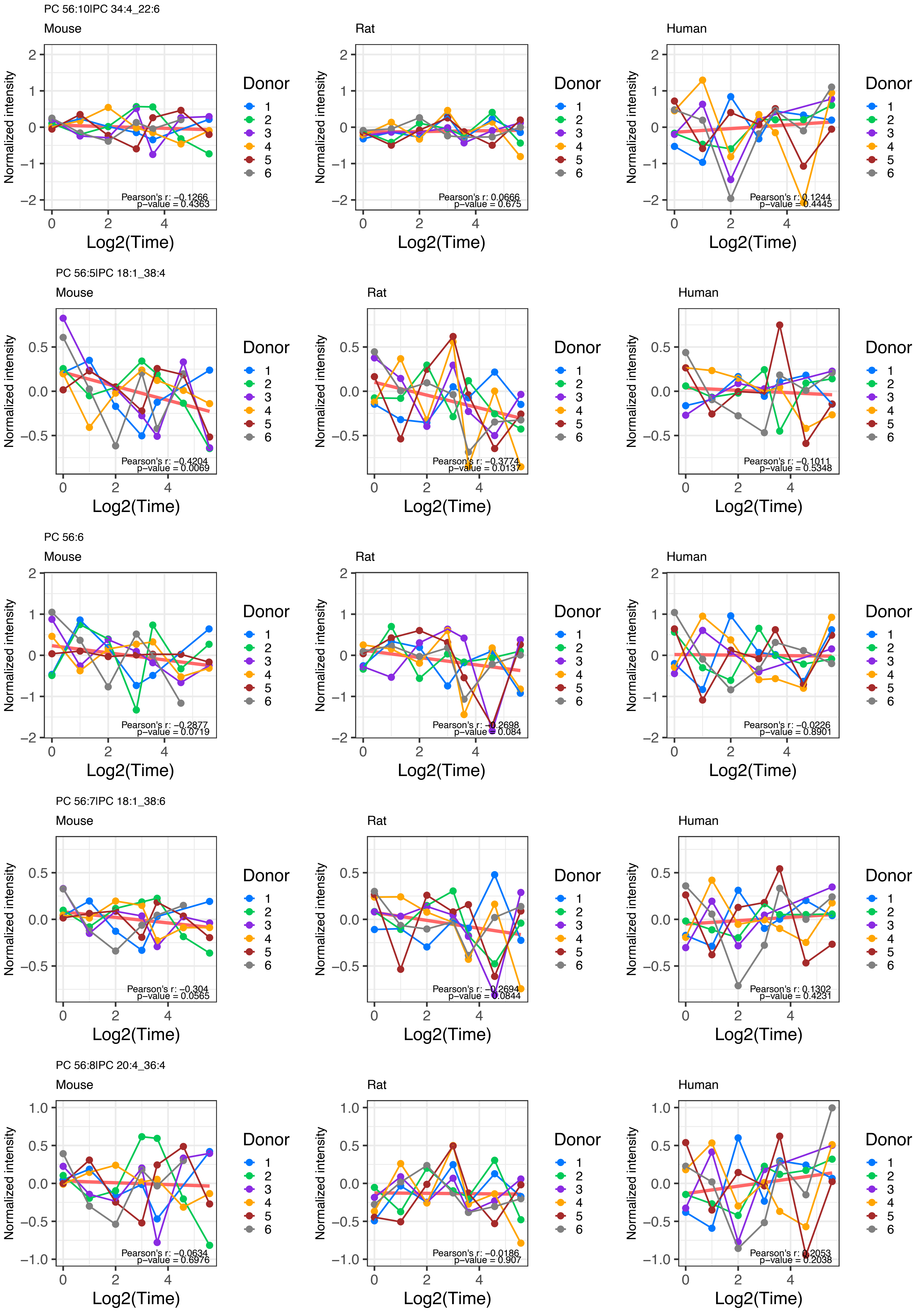
PC 56:10|PC 34:4_22:6
Mouse
Pearson's r: −0.1266
p−value = 0.4363
Rat
Pearson's r: 0.0666
p−value = 0.675
Human
Pearson's r: 0.1244
p−value = 0.4445
PC 56:5|PC 18:1_38:4
Mouse
Pearson's r: −0.4204
p−value = 0.0069
Rat
Pearson's r: −0.3774
p−value = 0.0137
Human
Pearson's r: −0.1011
p−value = 0.5348
PC 56:6
Mouse
Pearson's r: −0.2877
p−value = 0.0719
Rat
Pearson's r: −0.2698
p−value = 0.084
Human
Pearson's r: −0.0226
p−value = 0.8901
PC 56:7|PC 18:1_38:6
Mouse
Pearson's r: −0.304
p−value = 0.0565
Rat
Pearson's r: −0.2694
p−value = 0.0844
Human
Pearson's r: 0.1302
p−value = 0.4231
PC 56:8|PC 20:4_36:4
Mouse
Pearson's r: −0.0634
p−value = 0.6976
Rat
Pearson's r: −0.0186
p−value = 0.907
Human
Pearson's r: 0.2053
p−value = 0.2038
Normalized intensity
Log2(Time)
Donor
1
2
3
4
5
6

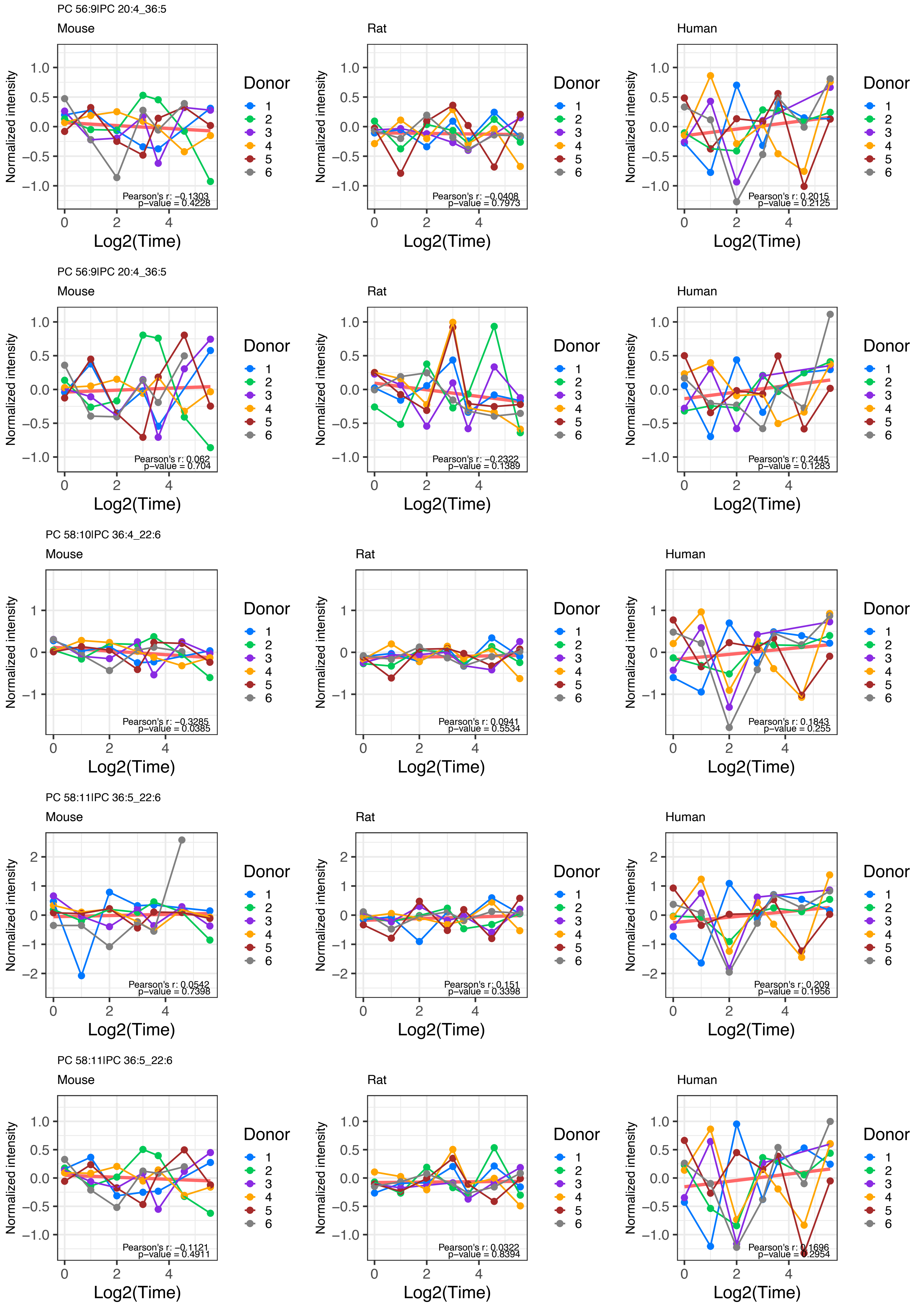

PC 56:9|PC 20:4_36:5
Mouse
Pearson's r: −0.1303
p−value = 0.4228
Rat
Pearson's r: −0.0408
p−value = 0.7973
Human
Pearson's r: 0.2015
p−value = 0.2125
PC 56:9|PC 20:4_36:5
Mouse
Pearson's r: 0.062
p−value = 0.704
Rat
Pearson's r: −0.2322
p−value = 0.1389
Human
Pearson's r: 0.2445
p−value = 0.1283
PC 58:10|PC 36:4_22:6
Mouse
Pearson's r: −0.3285
p−value = 0.0385
Rat
Pearson's r: 0.0941
p−value = 0.5534
Human
Pearson's r: 0.1843
p−value = 0.255
PC 58:11|PC 36:5_22:6
Mouse
Pearson's r: 0.0542
p−value = 0.7398
Rat
Pearson's r: 0.151
p−value = 0.3398
Human
Pearson's r: 0.209
p−value = 0.1956
PC 58:11|PC 36:5_22:6
Mouse
Pearson's r: −0.1121
p−value = 0.4911
Rat
Pearson's r: 0.0322
p−value = 0.8394
Human
Pearson's r: 0.1696
p−value = 0.2954
Normalized intensity
Log2(Time)
Donor
1
2
3
4
5
6

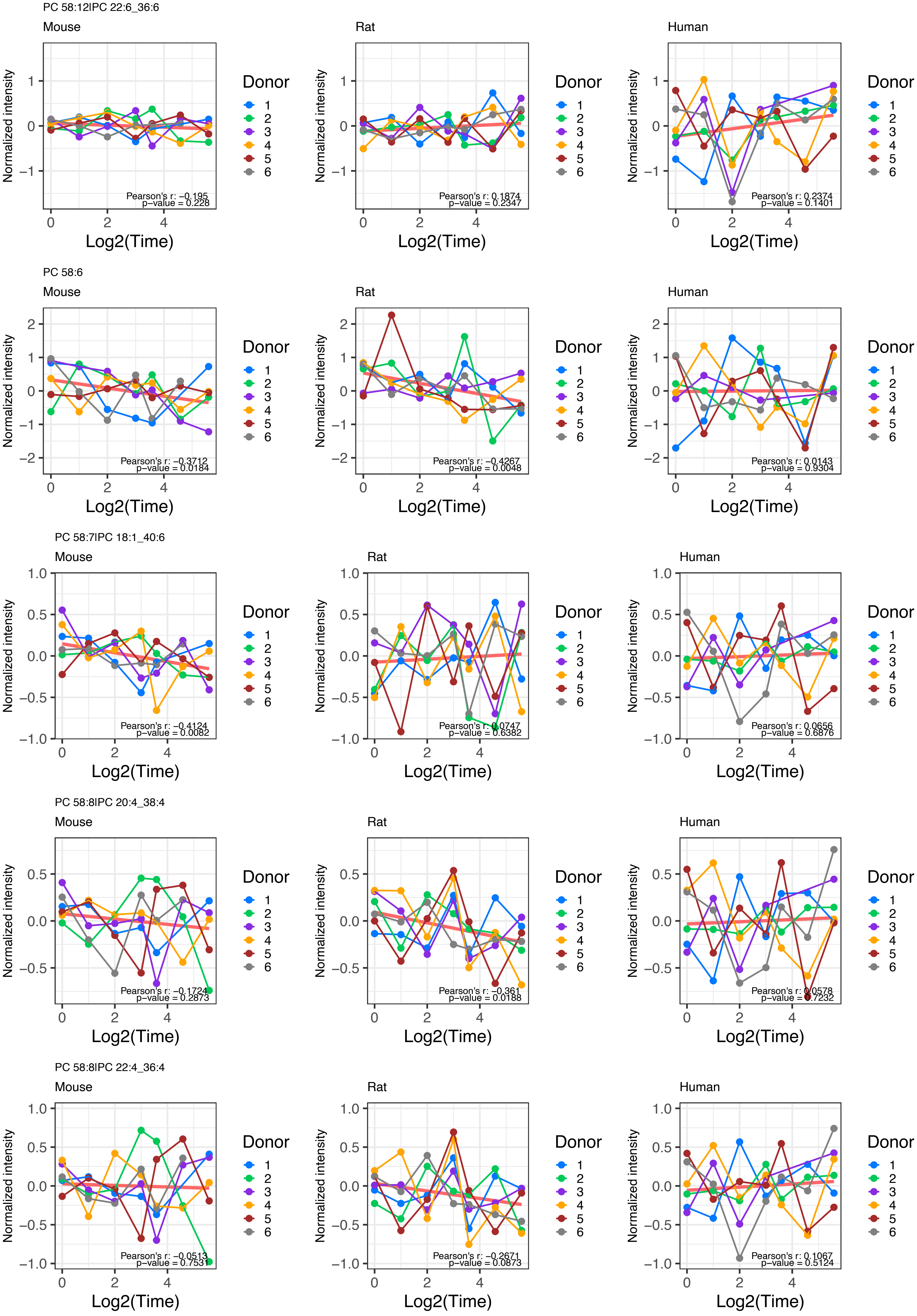
PC 58:12|PC 22:6_36:6
Mouse
Pearson's r: −0.195
p−value = 0.228
Rat
Pearson's r: 0.1874
p−value = 0.2347
Human
Pearson's r: 0.2374
p−value = 0.1401
PC 58:6
Mouse
Pearson's r: −0.3712
p−value = 0.0184
Rat
Pearson's r: −0.4267
p−value = 0.0048
Human
Pearson's r: 0.0143
p−value = 0.9304
PC 58:7|PC 18:1_40:6
Mouse
Pearson's r: −0.4124
p−value = 0.0082
Rat
Pearson's r: 0.0747
p−value = 0.6382
Human
Pearson's r: 0.0656
p−value = 0.6876
PC 58:8|PC 20:4_38:4
Mouse
Pearson's r: −0.1724
p−value = 0.2873
Rat
Pearson's r: −0.361
p−value = 0.0188
Human
Pearson's r: 0.0578
p−value = 0.7232
PC 58:8|PC 22:4_36:4
Mouse
Pearson's r: −0.0513
p−value = 0.7531
Rat
Pearson's r: −0.2671
p−value = 0.0873
Human
Pearson's r: 0.1067
p−value = 0.5124
Normalized intensity
Log2(Time)
Donor
1
2
3
4
5
6

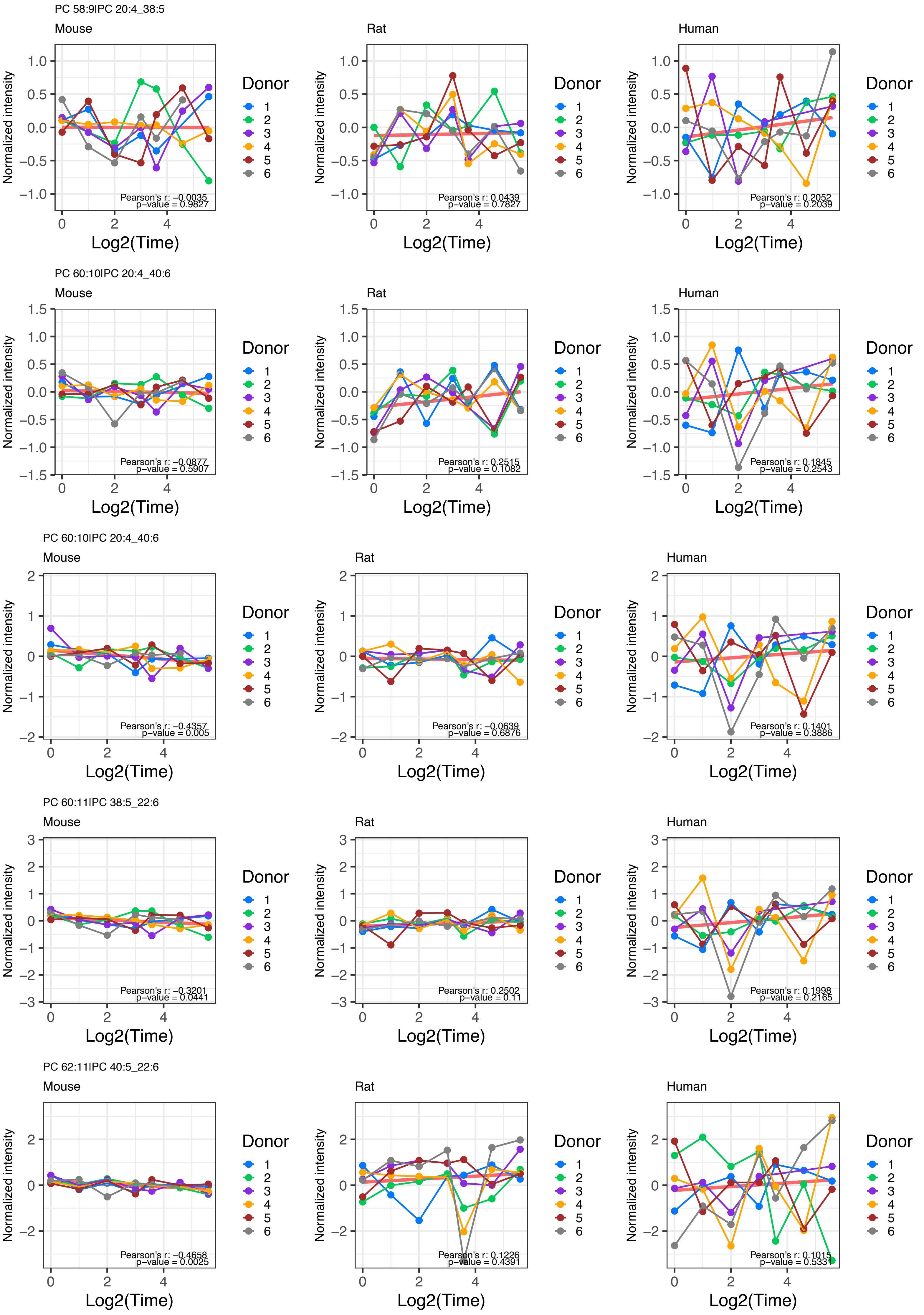
PC 58:9|PC 20:4_38:5
Mouse
Pearson's r: −0.0035
p−value = 0.9827
Rat
Pearson's r: 0.0439
p−value = 0.7827
Human
Pearson's r: 0.2052
p−value = 0.2039
PC 60:10|PC 20:4_40:6
Mouse
Pearson's r: −0.0877
p−value = 0.5907
Rat
Pearson's r: 0.2515
p−value = 0.1082
Human
Pearson's r: 0.1845
p−value = 0.2543
PC 60:10|PC 20:4_40:6
Mouse
Pearson's r: −0.4357
p−value = 0.005
Rat
Pearson's r: −0.0639
p−value = 0.6876
Human
Pearson's r: 0.1401
p−value = 0.3886
PC 60:11|PC 38:5_22:6
Mouse
Pearson's r: −0.3201
p−value = 0.0441
Rat
Pearson's r: 0.2502
p−value = 0.11
Human
Pearson's r: 0.1998
p−value = 0.2165
PC 62:11|PC 40:5_22:6
Mouse
Pearson's r: −0.4658
p−value = 0.0025
Rat
Pearson's r: 0.1226
p−value = 0.4391
Human
Pearson's r: 0.1015
p−value = 0.5331
Normalized intensity
Log2(Time)
Donor
1
2
3
4
5
6

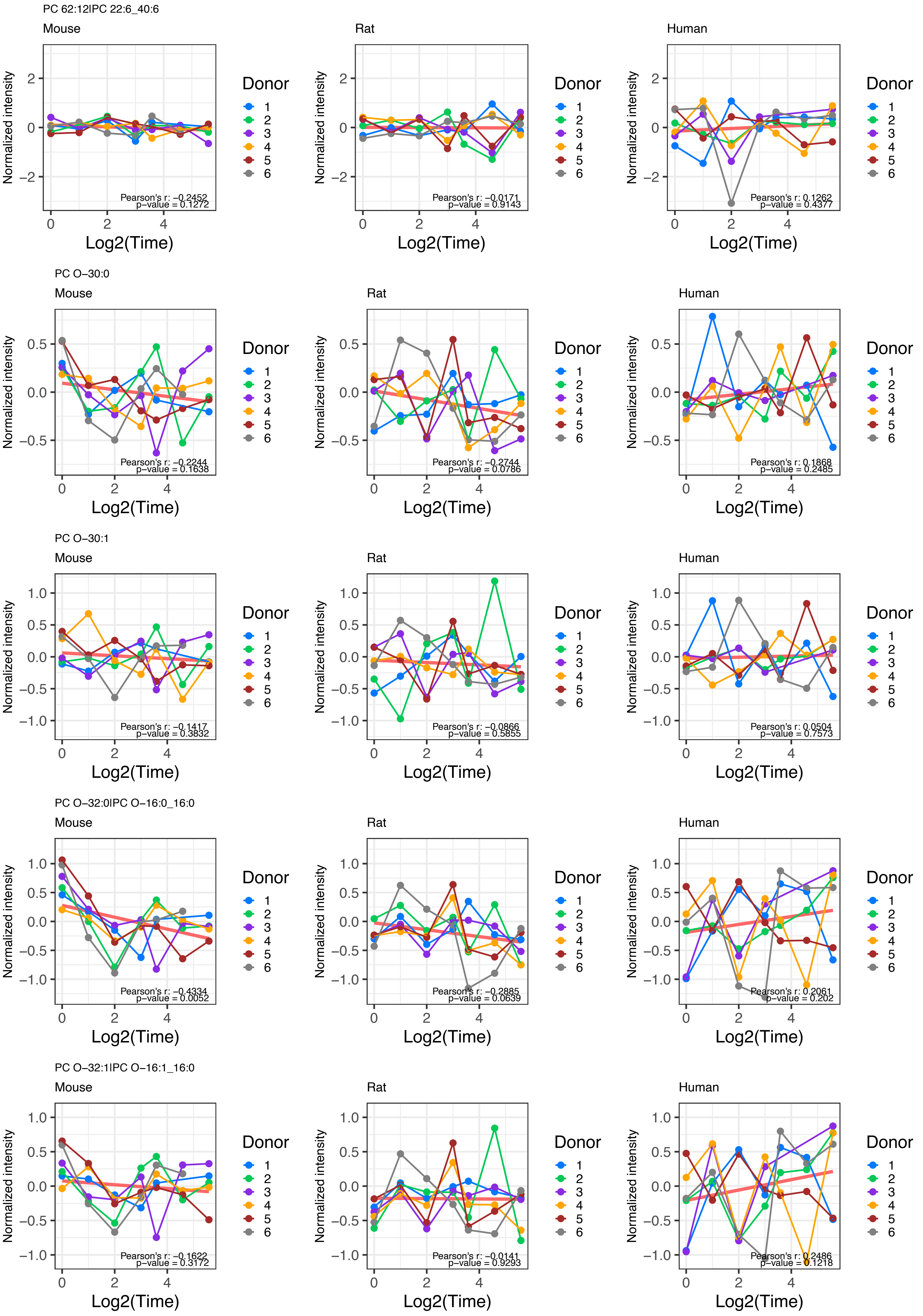

PC 62:12|PC 22:6_40:6
Mouse
Rat
Human
Normalized intensity
Log2(Time)
Donor
1
2
3
4
5
6
Pearson's r: −0.2452
p−value = 0.1272
Pearson's r: −0.0171
p−value = 0.9143
Pearson's r: 0.1262
p−value = 0.4377
PC O−30:0
Pearson's r: −0.2244
p−value = 0.1638
Pearson's r: −0.2744
p−value = 0.0786
Pearson's r: 0.1868
p−value = 0.2485
PC O−30:1
Pearson's r: −0.1417
p−value = 0.3832
Pearson's r: −0.0866
p−value = 0.5855
Pearson's r: 0.0504
p−value = 0.7573
PC O−32:0|PC O−16:0_16:0
Pearson's r: −0.4334
p−value = 0.0052
Pearson's r: −0.2885
p−value = 0.0639
Pearson's r: 0.2061
p−value = 0.202
PC O−32:1|PC O−16:1_16:0
Pearson's r: −0.1622
p−value = 0.3172
Pearson's r: −0.0141
p−value = 0.9293
Pearson's r: 0.2486
p−value = 0.1218

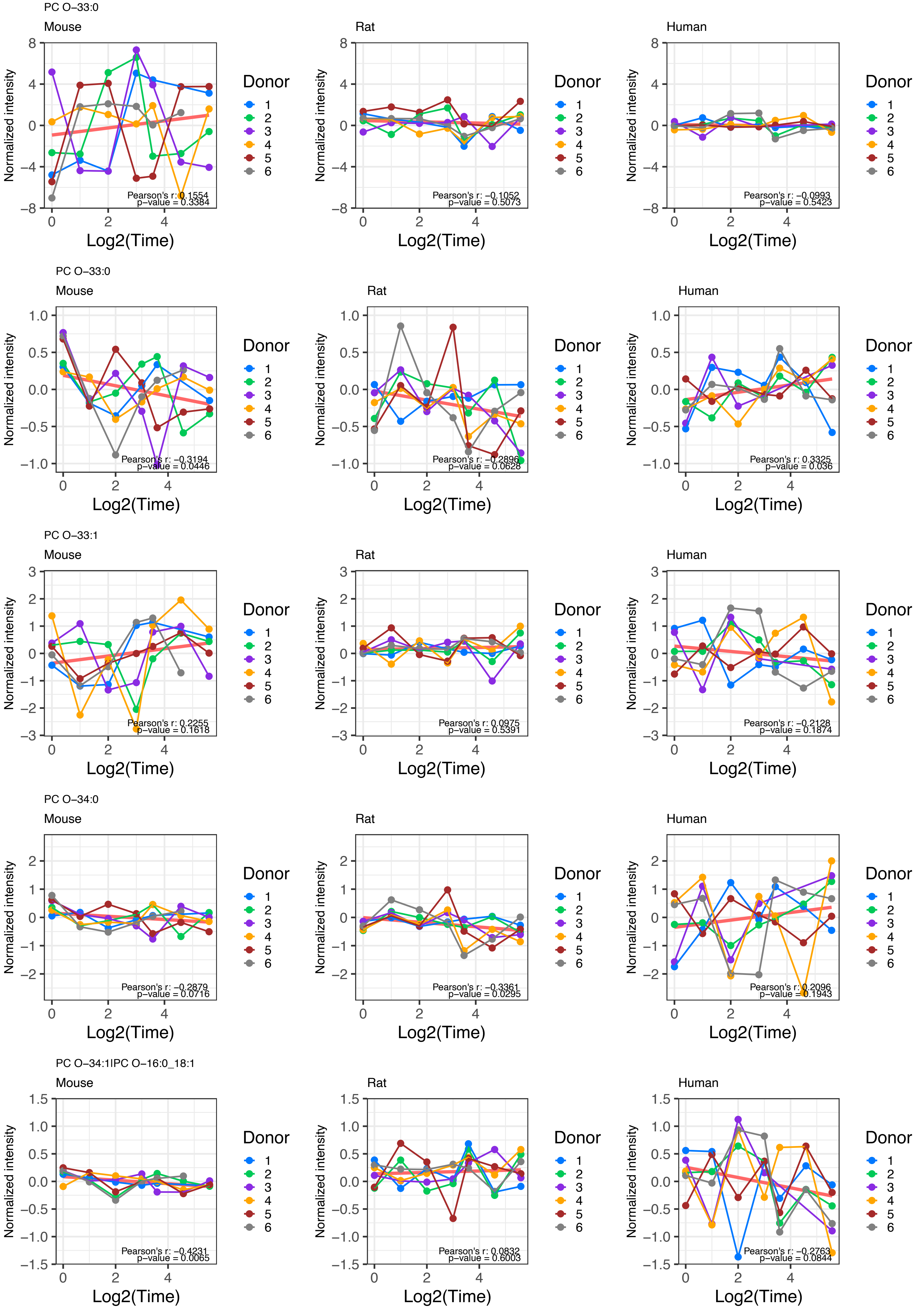

PC O−33:0
Mouse
Rat
Human
Normalized intensity
Log2(Time)
Donor
1
2
3
4
5
6
Pearson's r: 0.1554
p−value = 0.3384
Pearson's r: −0.1052
p−value = 0.5073
Pearson's r: −0.0993
p−value = 0.5423
PC O−33:0
Mouse
Rat
Human
Pearson's r: −0.3194
p−value = 0.0446
Pearson's r: −0.2896
p−value = 0.0628
Pearson's r: 0.3325
p−value = 0.036
PC O−33:1
Mouse
Rat
Human
Pearson's r: 0.2255
p−value = 0.1618
Pearson's r: 0.0975
p−value = 0.5391
Pearson's r: −0.2128
p−value = 0.1874
PC O−34:0
Mouse
Rat
Human
Pearson's r: −0.2879
p−value = 0.0716
Pearson's r: −0.3361
p−value = 0.0295
Pearson's r: 0.2096
p−value = 0.1943
PC O−34:1|PC O−16:0_18:1
Mouse
Rat
Human
Pearson's r: −0.4231
p−value = 0.0065
Pearson's r: 0.0832
p−value = 0.6003
Pearson's r: −0.2763
p−value = 0.0844

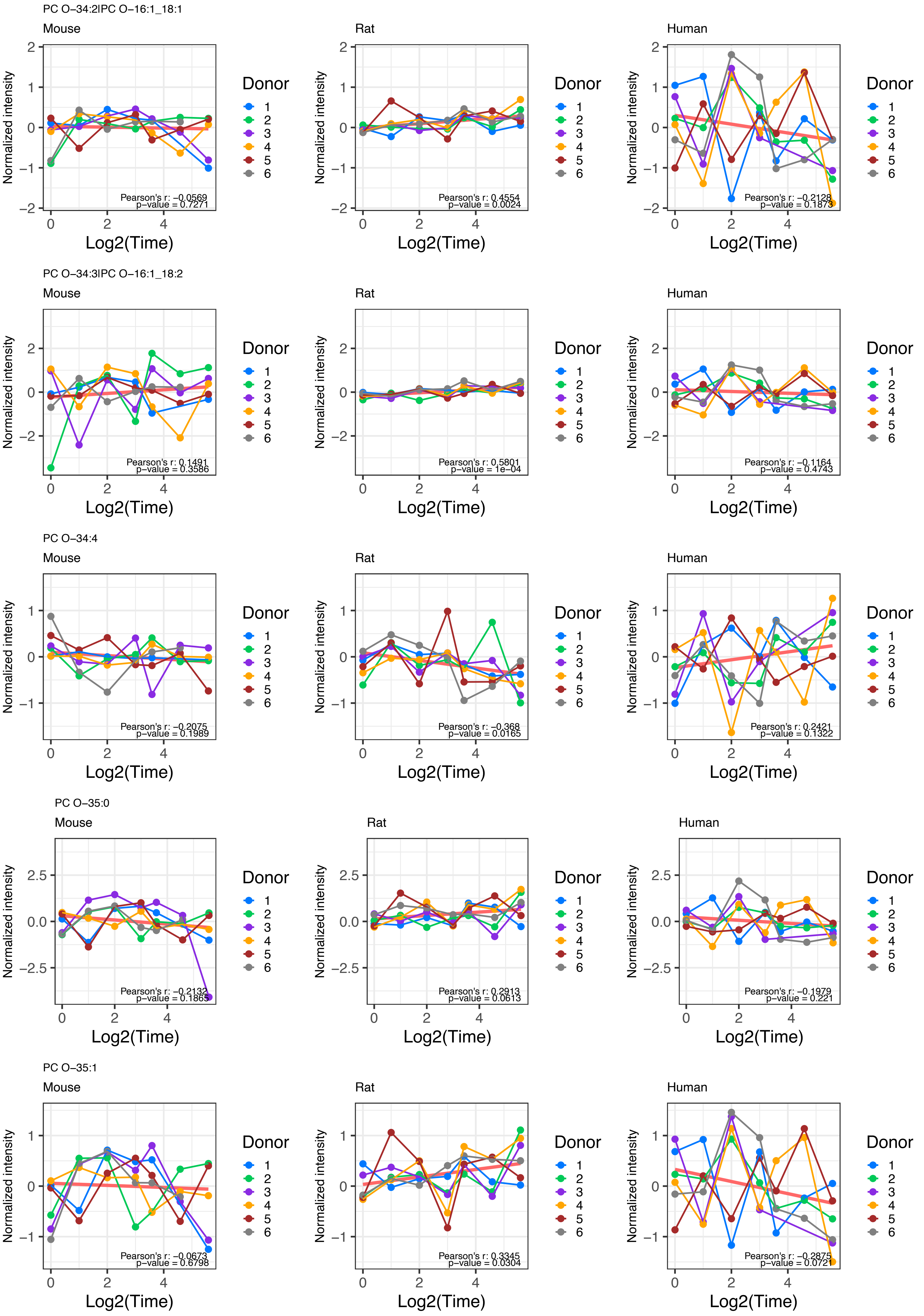
PC O-34:2|PC O-16:1_18:1
Mouse
Pearson's r: −0.0569
p−value = 0.7271
Rat
Pearson's r: 0.4554
p−value = 0.0024
Human
Pearson's r: −0.2128
p−value = 0.1873
PC O-34:3|PC O-16:1_18:2
Mouse
Pearson's r: 0.1491
p−value = 0.3586
Rat
Pearson's r: 0.5801
p−value = 1e−04
Human
Pearson's r: −0.1164
p−value = 0.4743
PC O−34:4
Mouse
Pearson's r: −0.2075
p−value = 0.1989
Rat
Pearson's r: −0.368
p−value = 0.0165
Human
Pearson's r: 0.2421
p−value = 0.1322
PC O−35:0
Mouse
Pearson's r: −0.2132
p−value = 0.1865
Rat
Pearson's r: 0.2913
p−value = 0.0613
Human
Pearson's r: −0.1979
p−value = 0.221
PC O−35:1
Mouse
Pearson's r: −0.0673
p−value = 0.6798
Rat
Pearson's r: 0.3345
p−value = 0.0304
Human
Pearson's r: −0.2875
p−value = 0.0721
Normalized intensity
Log2(Time)
Donor
1
2
3
4
5
6

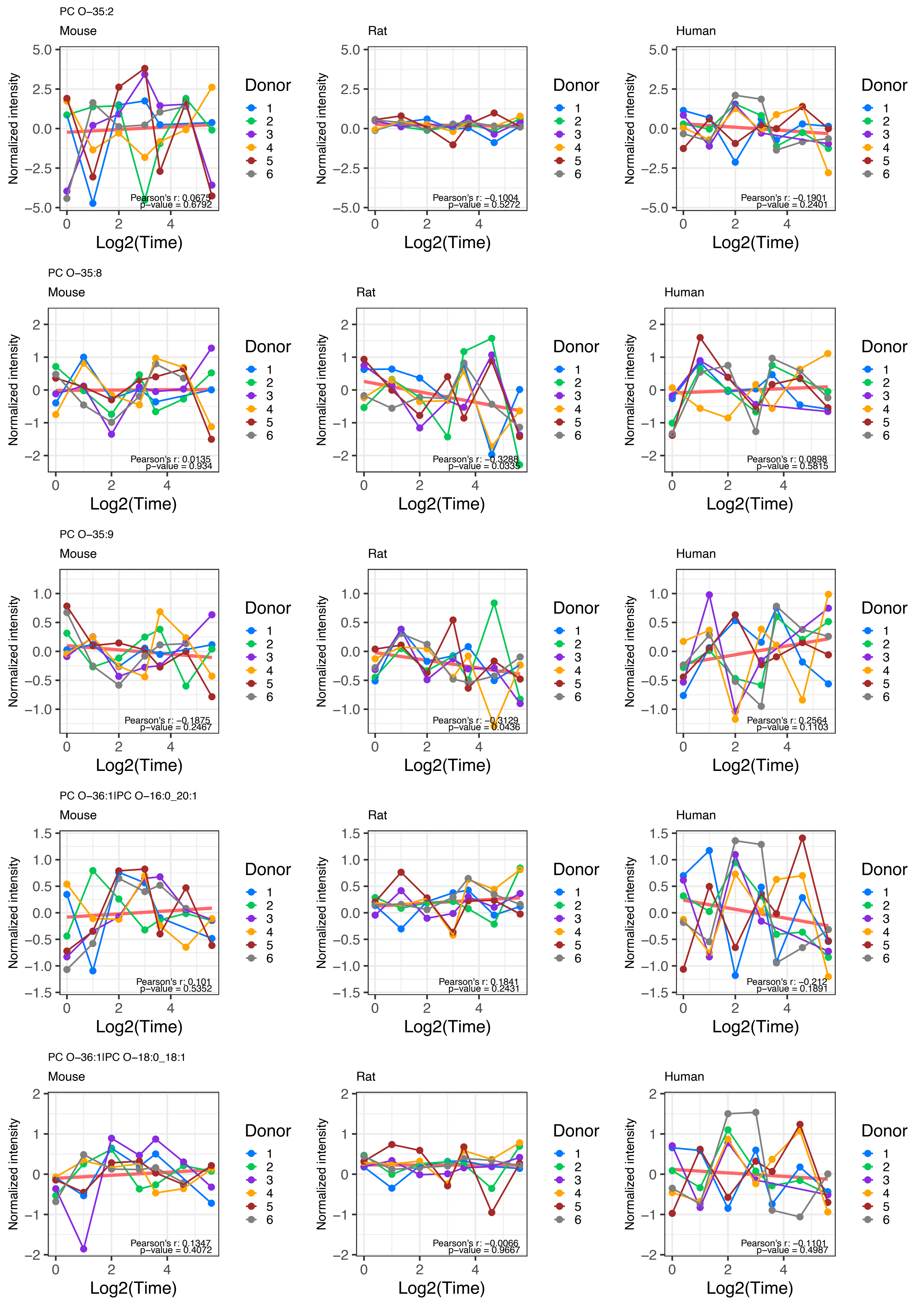

PC O−35:2
Mouse
Pearson's r: 0.0675
p−value = 0.6792
Rat
Pearson's r: −0.1004
p−value = 0.5272
Human
Pearson's r: −0.1901
p−value = 0.2401
PC O−35:8
Mouse
Pearson's r: 0.0135
p−value = 0.934
Rat
Pearson's r: −0.3288
p−value = 0.0335
Human
Pearson's r: 0.0898
p−value = 0.5815
PC O−35:9
Mouse
Pearson's r: −0.1875
p−value = 0.2467
Rat
Pearson's r: −0.3129
p−value = 0.0436
Human
Pearson's r: 0.2564
p−value = 0.1103
PC O−36:1|PC O−16:0_20:1
Mouse
Pearson's r: 0.101
p−value = 0.5352
Rat
Pearson's r: 0.1841
p−value = 0.2431
Human
Pearson's r: −0.212
p−value = 0.1891
PC O−36:1|PC O−18:0_18:1
Mouse
Pearson's r: 0.1347
p−value = 0.4072
Rat
Pearson's r: −0.0066
p−value = 0.9667
Human
Pearson's r: −0.1101
p−value = 0.4987
Normalized intensity
Log2(Time)
Donor
1
2
3
4
5
6

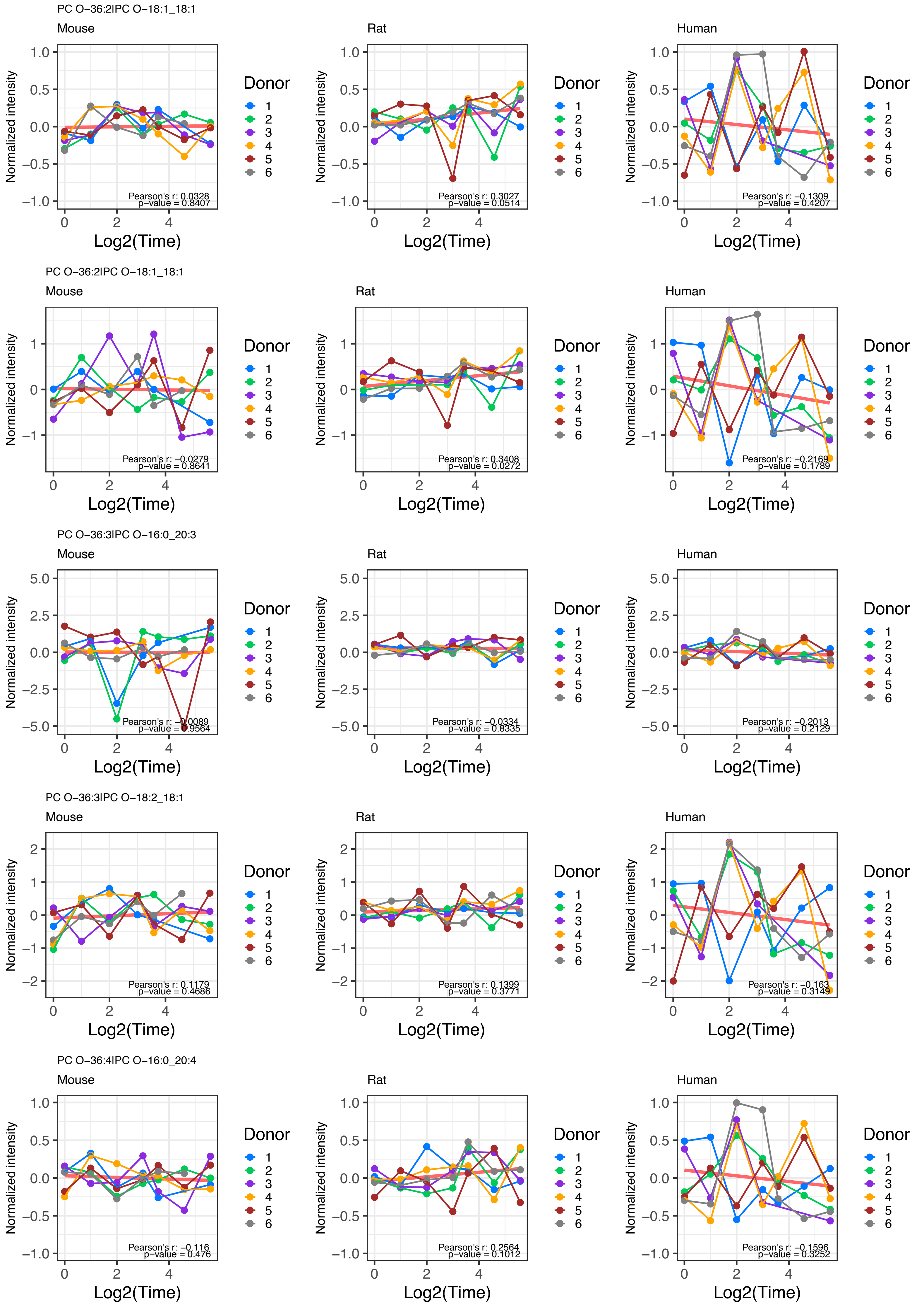
PC O−36:2|PC O−18:1_18:1
Mouse
Normalized intensity
Log2(Time)
Pearson's r: 0.0328
p−value = 0.8407
Rat
Normalized intensity
Log2(Time)
Pearson's r: 0.3027
p−value = 0.0514
Human
Normalized intensity
Log2(Time)
Pearson's r: −0.1309
p−value = 0.4207
Donor
1
2
3
4
5
6
PC O−36:2|PC O−18:1_18:1
Mouse
Pearson's r: −0.0279
p−value = 0.8641
Rat
Pearson's r: 0.3408
p−value = 0.0272
Human
Pearson's r: −0.2169
p−value = 0.1789
PC O−36:3|PC O−16:0_20:3
Mouse
Pearson's r: −0.0089
p−value = 0.9564
Rat
Pearson's r: −0.0334
p−value = 0.8335
Human
Pearson's r: −0.2013
p−value = 0.2129
PC O−36:3|PC O−18:2_18:1
Mouse
Pearson's r: 0.1179
p−value = 0.4686
Rat
Pearson's r: 0.1399
p−value = 0.3771
Human
Pearson's r: −0.163
p−value = 0.3149
PC O−36:4|PC O−16:0_20:4
Mouse
Pearson's r: −0.116
p−value = 0.476
Rat
Pearson's r: 0.2564
p−value = 0.1012
Human
Pearson's r: −0.1596
p−value = 0.3252

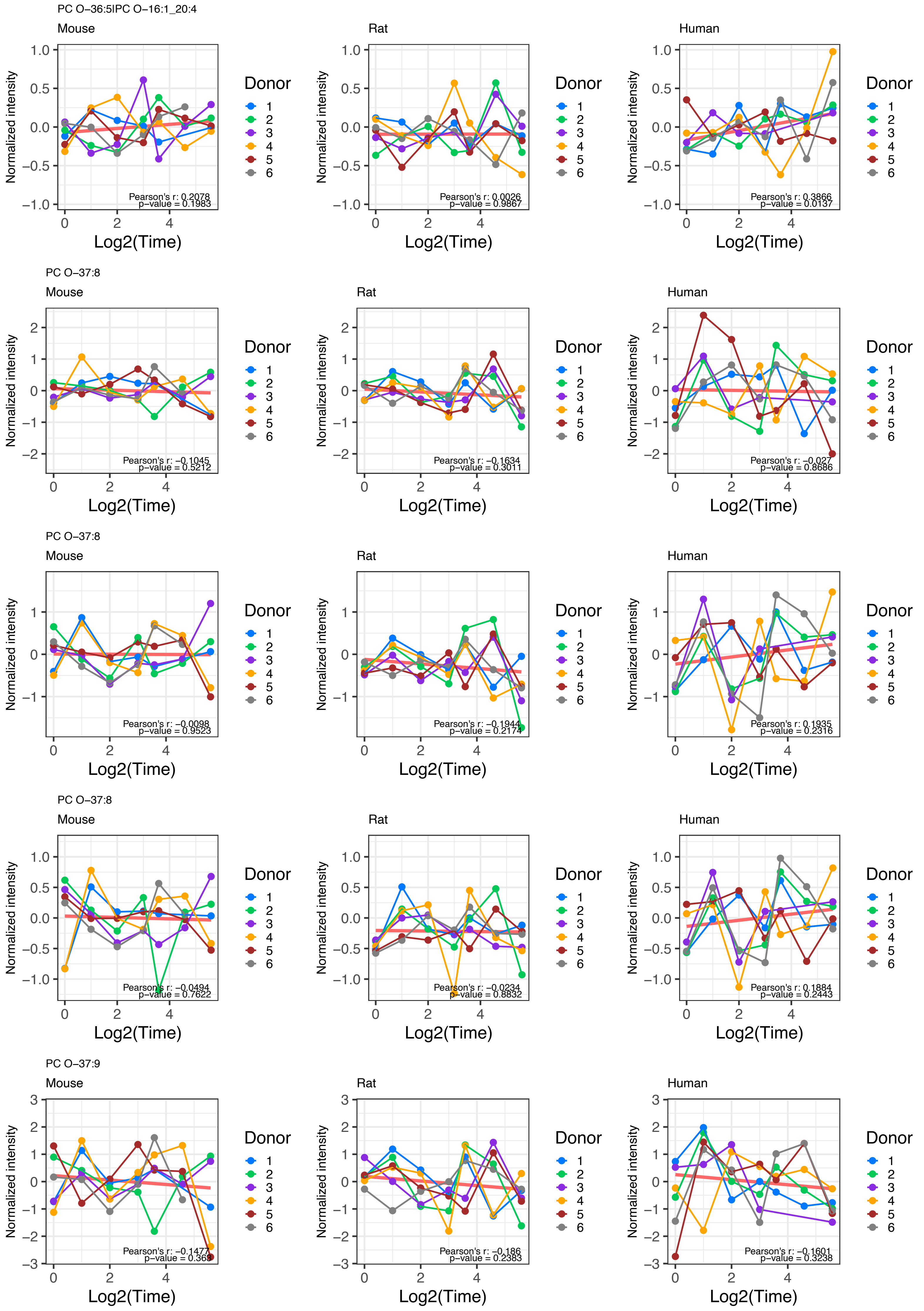
PC O−36:5|PC O−16:1_20:4
Mouse
Pearson's r: 0.2078
p−value = 0.1983
Rat
Pearson's r: 0.0026
p−value = 0.9867
Human
Pearson's r: 0.3866
p−value = 0.0137
PC O−37:8
Mouse
Pearson's r: −0.1045
p−value = 0.5212
Rat
Pearson's r: −0.1634
p−value = 0.3011
Human
Pearson's r: −0.027
p−value = 0.8686
PC O−37:8
Mouse
Pearson's r: −0.0098
p−value = 0.9523
Rat
Pearson's r: −0.1944
p−value = 0.2174
Human
Pearson's r: 0.1935
p−value = 0.2316
PC O−37:8
Mouse
Pearson's r: −0.0494
p−value = 0.7622
Rat
Pearson's r: −0.0234
p−value = 0.8832
Human
Pearson's r: 0.1884
p−value = 0.2443
PC O−37:9
Mouse
Pearson's r: −0.1477
p−value = 0.368
Rat
Pearson's r: −0.186
p−value = 0.2383
Human
Pearson's r: −0.1601
p−value = 0.3238
Normalized intensity
Log2(Time)
Donor
1
2
3
4
5
6

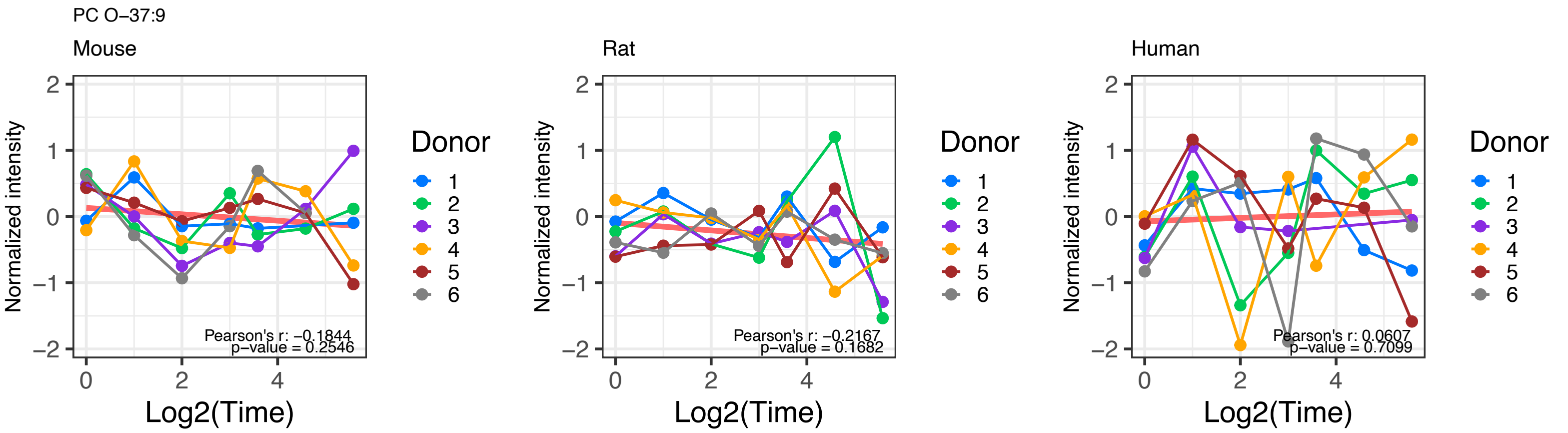
PC O−37:9
Mouse
Rat
Human
Normalized intensity
Log2(Time)
Donor
1
2
3
4
5
6
Pearson's r: −0.1844
p−value = 0.2546
Pearson's r: −0.2167
p−value = 0.1682
Pearson's r: 0.0607
p−value = 0.7099

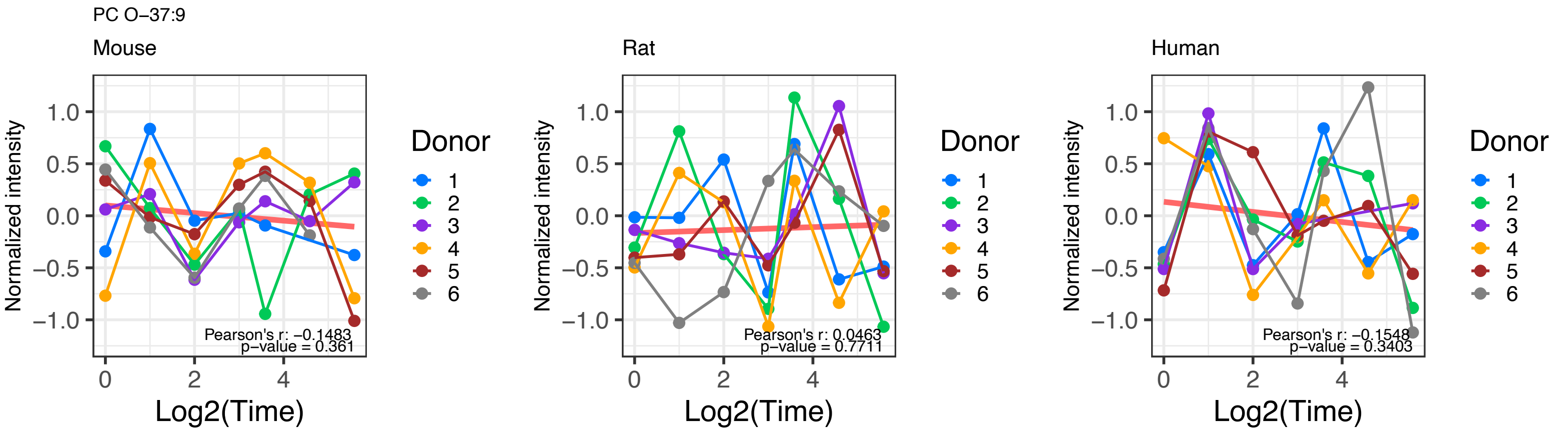
PC O−37:9
Mouse
Rat
Human
Normalized intensity
Log2(Time)
Donor
1
2
3
4
5
6
Pearson's r: −0.1483
p−value = 0.361
Pearson's r: 0.0463
p−value = 0.7711
Pearson's r: −0.1548
p−value = 0.3403

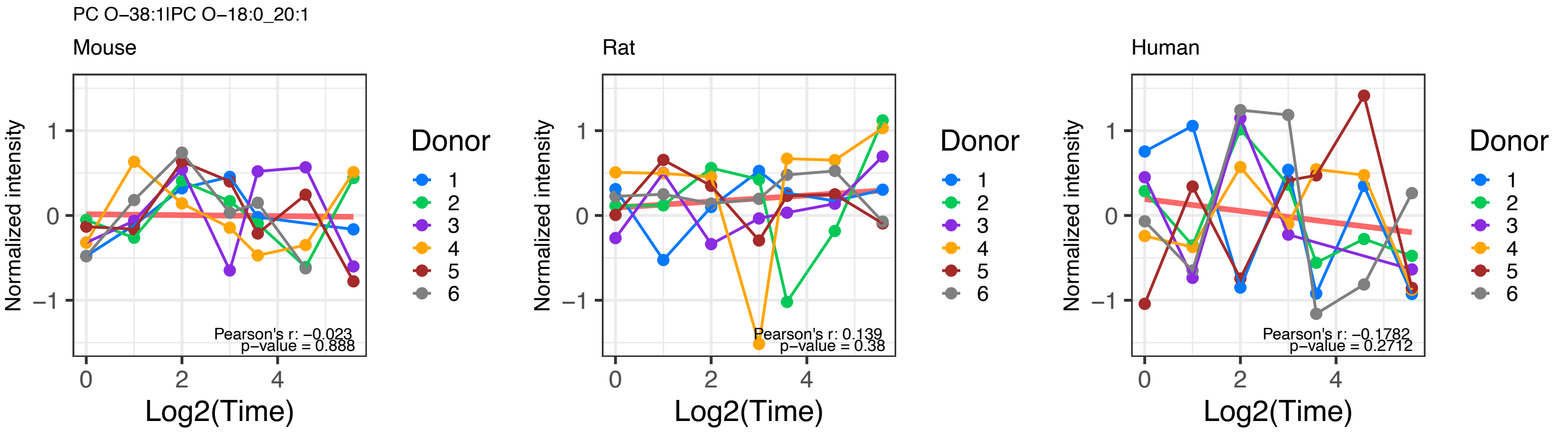
PC O−38:1|PC O−18:0_20:1
Mouse
Rat
Human
Normalized intensity
Log2(Time)
Donor
1
2
3
4
5
6
Pearson's r: −0.023
p−value = 0.888
Pearson's r: 0.139
p−value = 0.38
Pearson's r: −0.1782
p−value = 0.2712

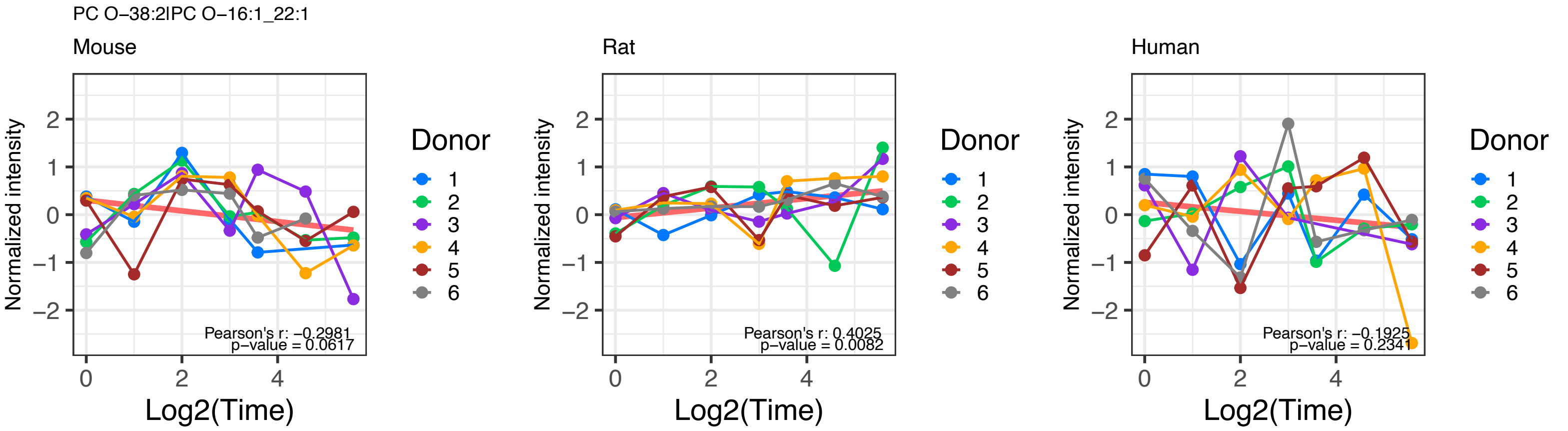
PC O−38:2|PC O−16:1_22:1
Mouse
Rat
Human
Normalized intensity
Log2(Time)
Donor
1
2
3
4
5
6
Pearson's r: −0.2981
p−value = 0.0617
Pearson's r: 0.4025
p−value = 0.0082
Pearson's r: −0.1925
p−value = 0.2341

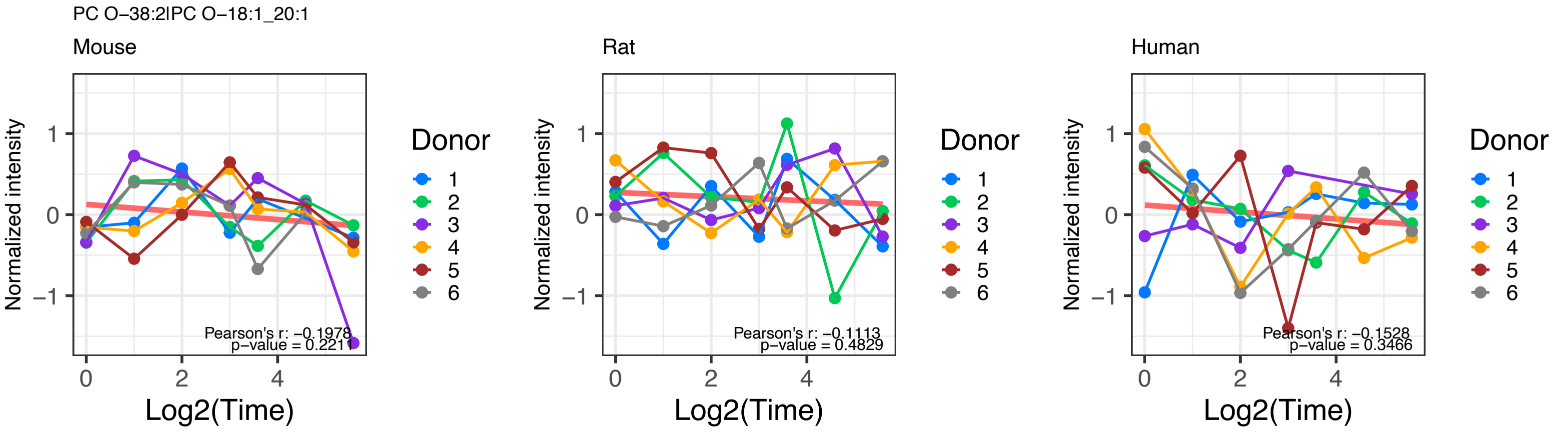
PC O−38:2|PC O−18:1_20:1
Mouse
Rat
Human
Normalized intensity
Log2(Time)
Donor
1
2
3
4
5
6
Pearson's r: −0.1978
p−value = 0.2211
Pearson's r: −0.1113
p−value = 0.4829
Pearson's r: −0.1528
p−value = 0.3466

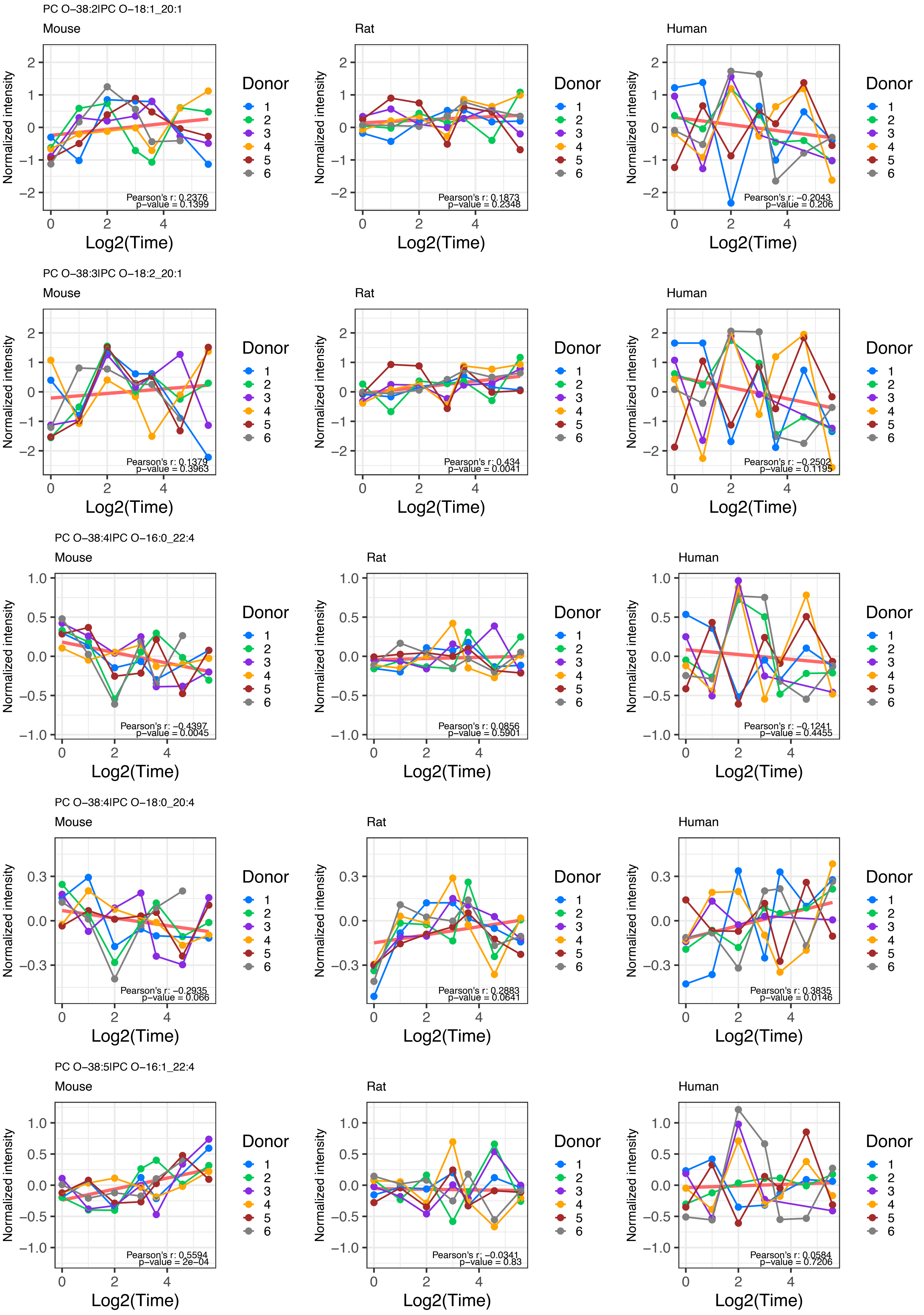
PC O−38:2|PC O−18:1_20:1
Mouse
Pearson's r: 0.2376
p−value = 0.1399
Rat
Pearson's r: 0.1873
p−value = 0.2348
Human
Pearson's r: −0.2043
p−value = 0.206
PC O−38:3|PC O−18:2_20:1
Mouse
Pearson's r: 0.1379
p−value = 0.3963
Rat
Pearson's r: 0.434
p−value = 0.0041
Human
Pearson's r: −0.2502
p−value = 0.1195
PC O−38:4|PC O−16:0_22:4
Mouse
Pearson's r: −0.4397
p−value = 0.0045
Rat
Pearson's r: 0.0856
p−value = 0.5901
Human
Pearson's r: −0.1241
p−value = 0.4455
PC O−38:4|PC O−18:0_20:4
Mouse
Pearson's r: −0.2935
p−value = 0.066
Rat
Pearson's r: 0.2883
p−value = 0.0641
Human
Pearson's r: 0.3835
p−value = 0.0146
PC O−38:5|PC O−16:1_22:4
Mouse
Pearson's r: 0.5594
p−value = 2e−04
Rat
Pearson's r: −0.0341
p−value = 0.83
Human
Pearson's r: 0.0584
p−value = 0.7206
Normalized intensity
Log2(Time)
Donor
1
2
3
4
5
6

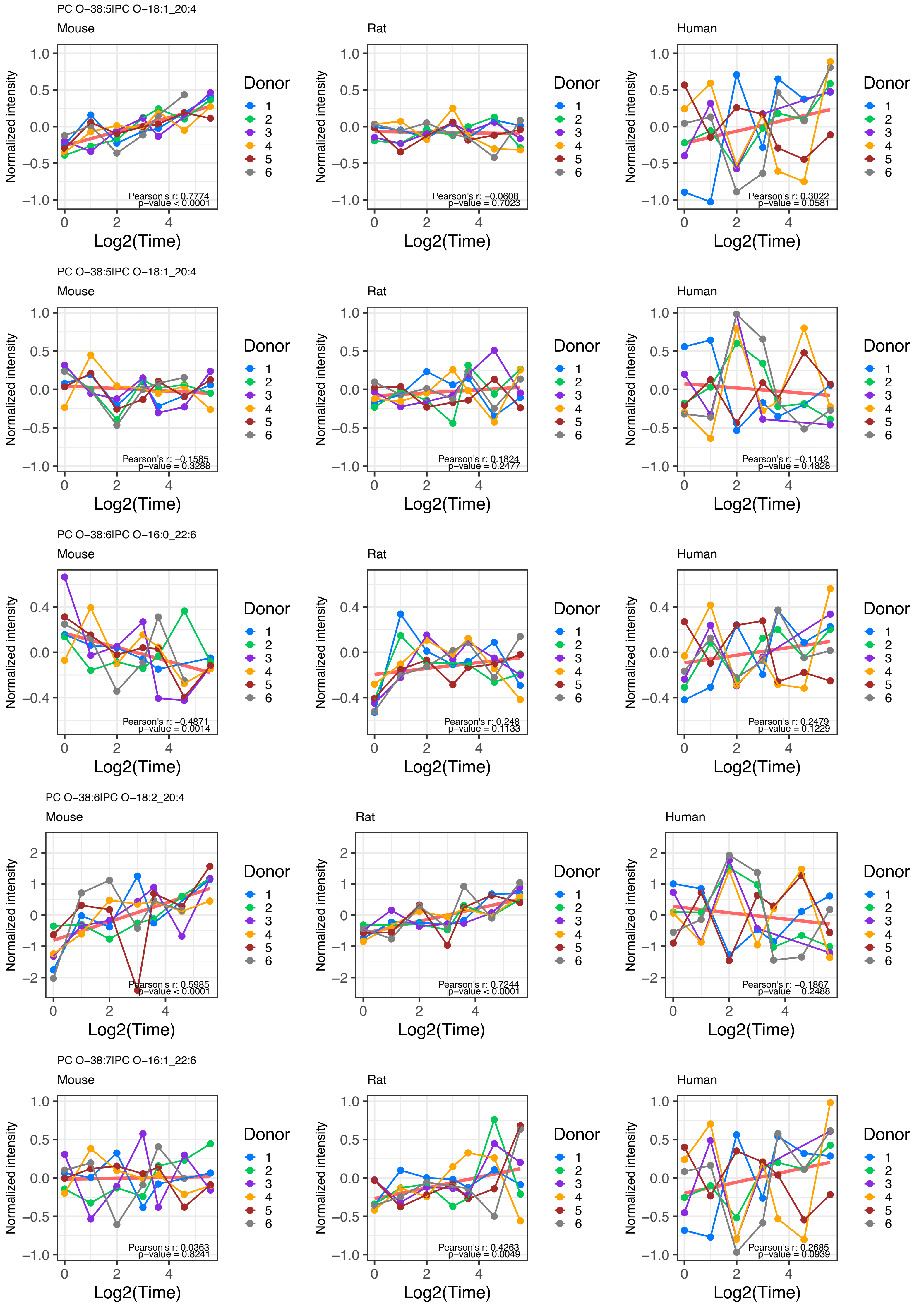
PC O−38:5|PC O−18:1_20:4
Mouse
Rat
Human
Pearson's r: 0.7774
p−value < 0.0001
Pearson's r: −0.0608
p−value = 0.7023
Pearson's r: 0.3022
p−value = 0.0581
PC O−38:5|PC O−18:1_20:4
Mouse
Rat
Human
Pearson's r: −0.1585
p−value = 0.3288
Pearson's r: 0.1824
p−value = 0.2477
Pearson's r: −0.1142
p−value = 0.4828
PC O−38:6|PC O−16:0_22:6
Mouse
Rat
Human
Pearson's r: −0.4871
p−value = 0.0014
Pearson's r: 0.248
p−value = 0.1133
Pearson's r: 0.2479
p−value = 0.1229
PC O−38:6|PC O−18:2_20:4
Mouse
Rat
Human
Pearson's r: 0.5985
p−value < 0.0001
Pearson's r: 0.7244
p−value < 0.0001
Pearson's r: −0.1867
p−value = 0.2488
PC O−38:7|PC O−16:1_22:6
Mouse
Rat
Human
Pearson's r: 0.0363
p−value = 0.8241
Pearson's r: 0.4263
p−value = 0.0049
Pearson's r: 0.2685
p−value = 0.0939
Normalized intensity
Log2(Time)
Donor
1
2
3
4
5
6

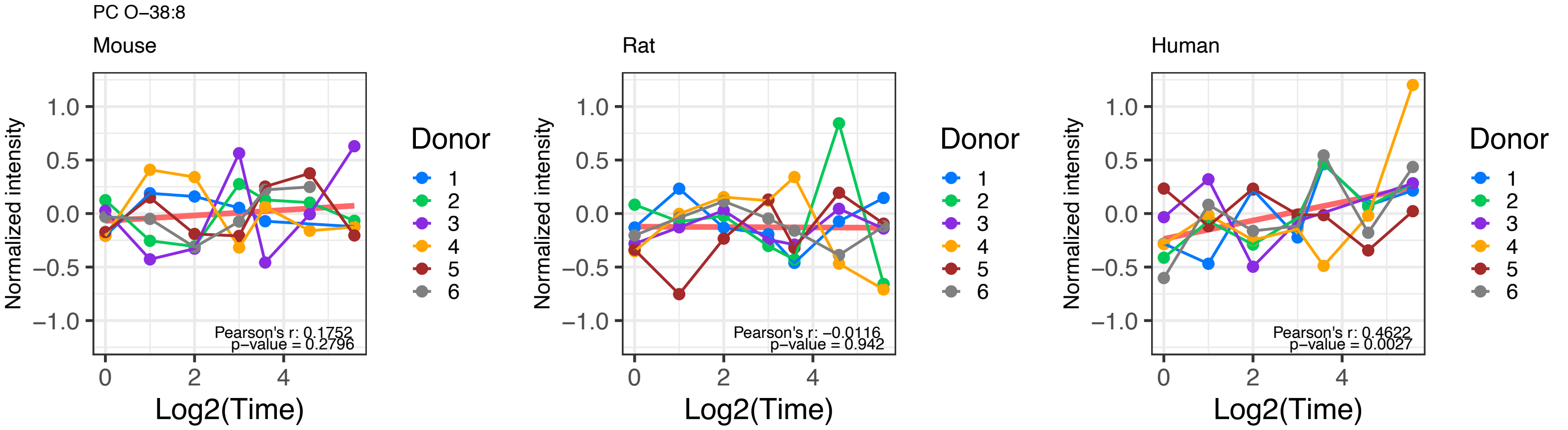
PC O−38:8
Mouse
Rat
Human
Normalized intensity
Log2(Time)
Donor
1
2
3
4
5
6
Pearson's r: 0.1752
p−value = 0.2796
Pearson's r: −0.0116
p−value = 0.942
Pearson's r: 0.4622
p−value = 0.0027

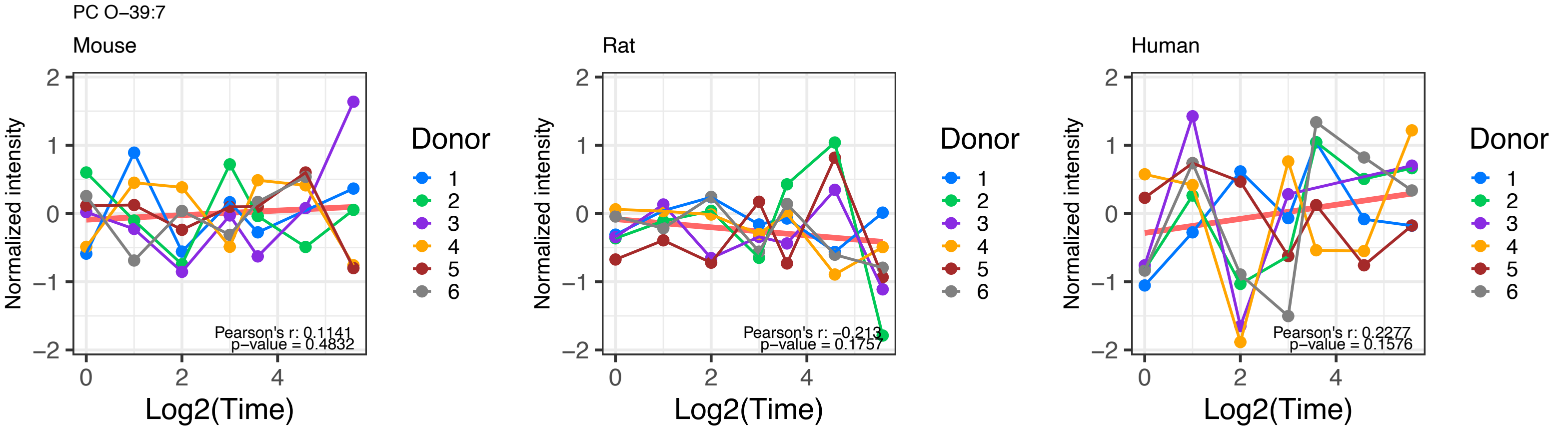
PC O−39:7
Mouse
Rat
Human
Normalized intensity
Log2(Time)
Donor
1
2
3
4
5
6
Pearson's r: 0.1141
p−value = 0.4832
Pearson's r: −0.213
p−value = 0.1757
Pearson's r: 0.2277
p−value = 0.1576

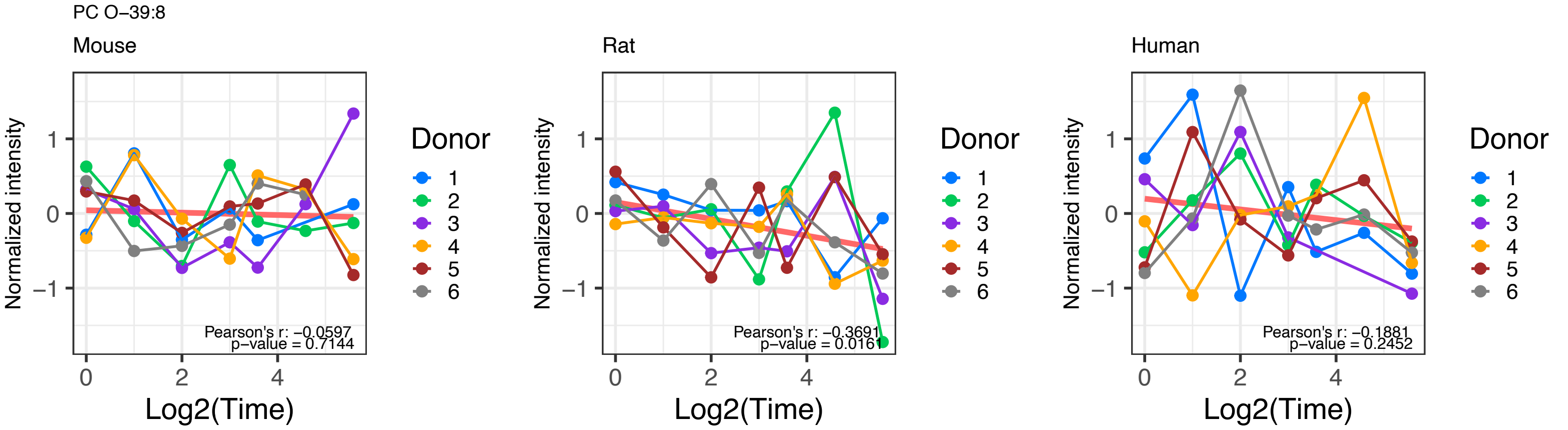
PC O−39:8
Mouse
Rat
Human
Normalized intensity
Log2(Time)
Donor
1
2
3
4
5
6
Pearson's r: −0.0597
p−value = 0.7144
Pearson's r: −0.3691
p−value = 0.0161
Pearson's r: −0.1881
p−value = 0.2452

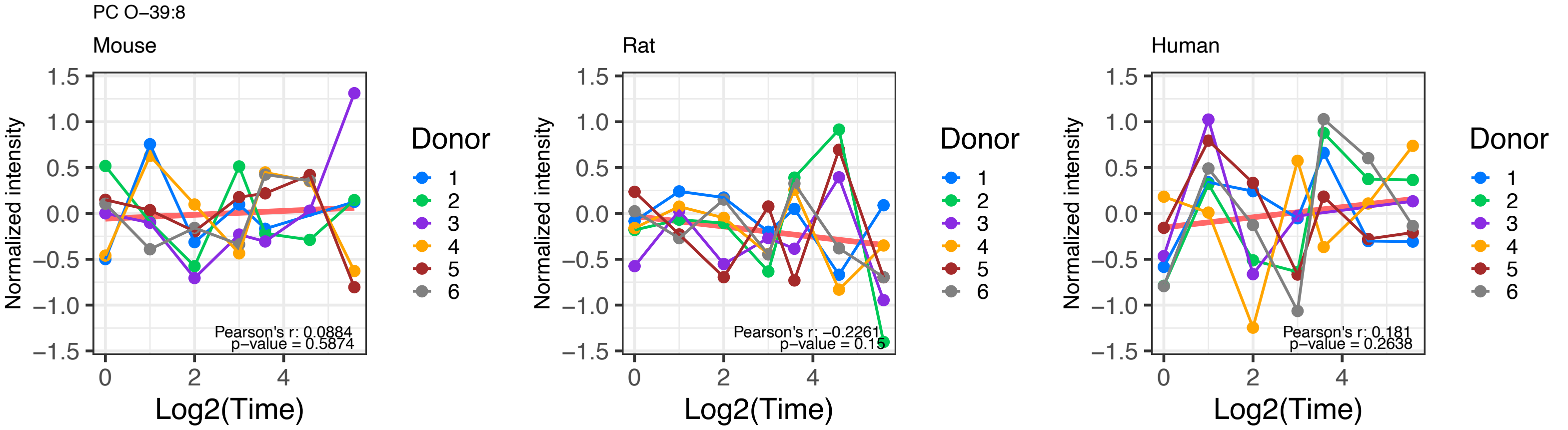
PC O−39:8
Mouse
Rat
Human
Normalized intensity
Log2(Time)
Donor
1
2
3
4
5
6
Pearson's r: 0.0884
p−value = 0.5874
Pearson's r: −0.2261
p−value = 0.15
Pearson's r: 0.181
p−value = 0.2638

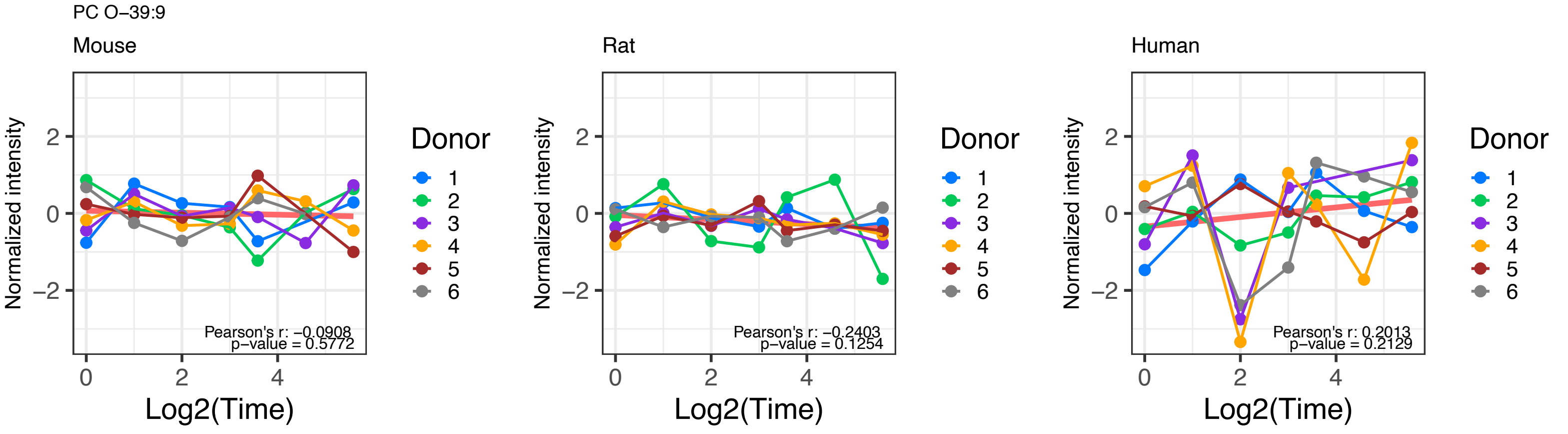
PC O−39:9
Mouse
Rat
Human
Normalized intensity
Log2(Time)
Donor
1
2
3
4
5
6
Pearson's r: −0.0908
p−value = 0.5772
Pearson's r: −0.2403
p−value = 0.1254
Pearson's r: 0.2013
p−value = 0.2129

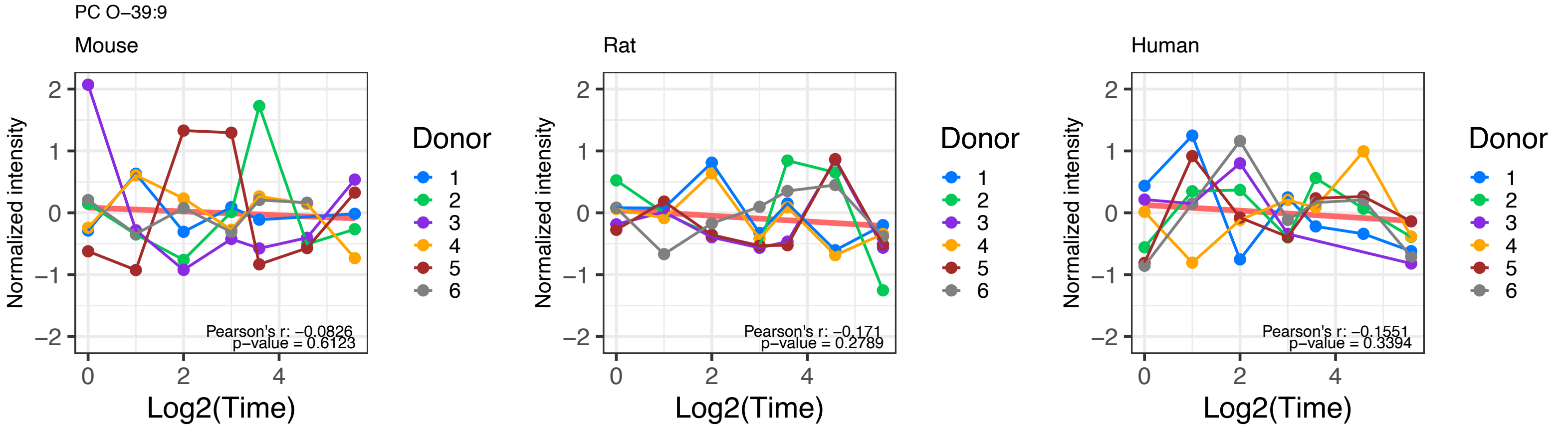
PC O−39:9
Mouse
Rat
Human
Normalized intensity
Log2(Time)
Donor
1
2
3
4
5
6
Pearson's r: −0.0826
p−value = 0.6123
Pearson's r: −0.171
p−value = 0.2789
Pearson's r: −0.1551
p−value = 0.3394

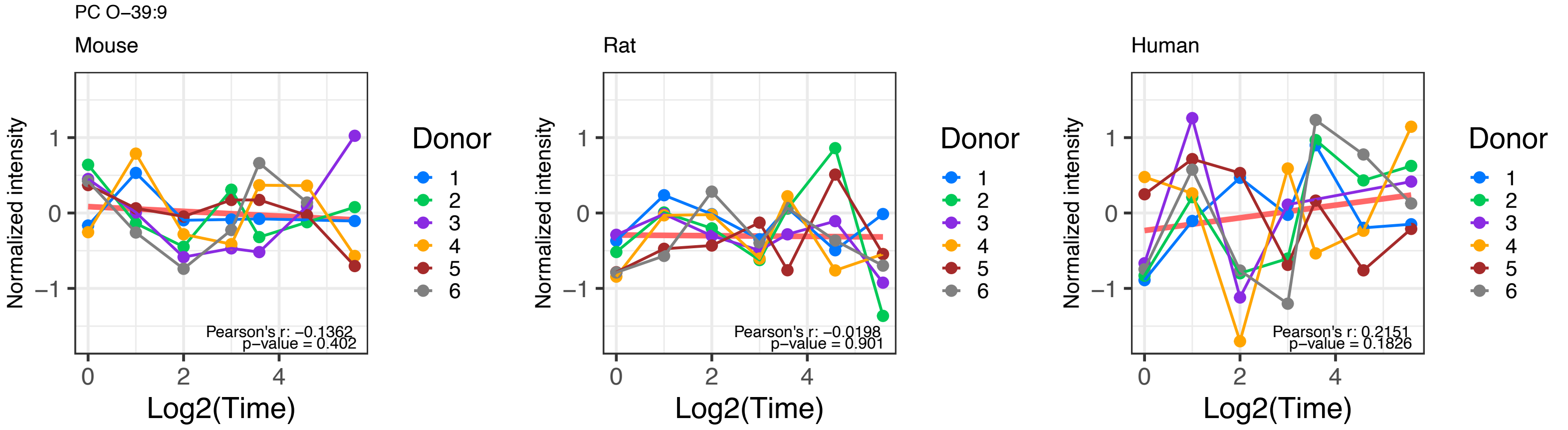
PC O−39:9
Mouse
Rat
Human
Normalized intensity
Log2(Time)
Donor
1
2
3
4
5
6
Pearson's r: −0.1362
p−value = 0.402
Pearson's r: −0.0198
p−value = 0.901
Pearson's r: 0.2151
p−value = 0.1826

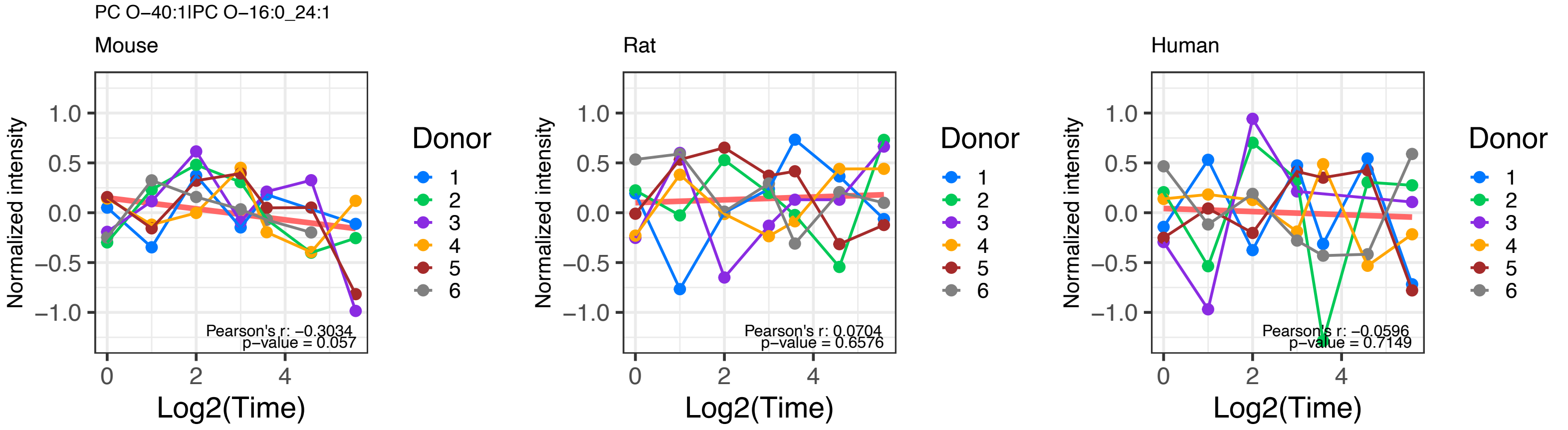
PC O−40:1|PC O−16:0_24:1
Mouse
Rat
Human
Normalized intensity
Log2(Time)
Donor
1
2
3
4
5
6
Pearson's r: −0.3034
p−value = 0.057
Pearson's r: 0.0704
p−value = 0.6576
Pearson's r: −0.0596
p−value = 0.7149

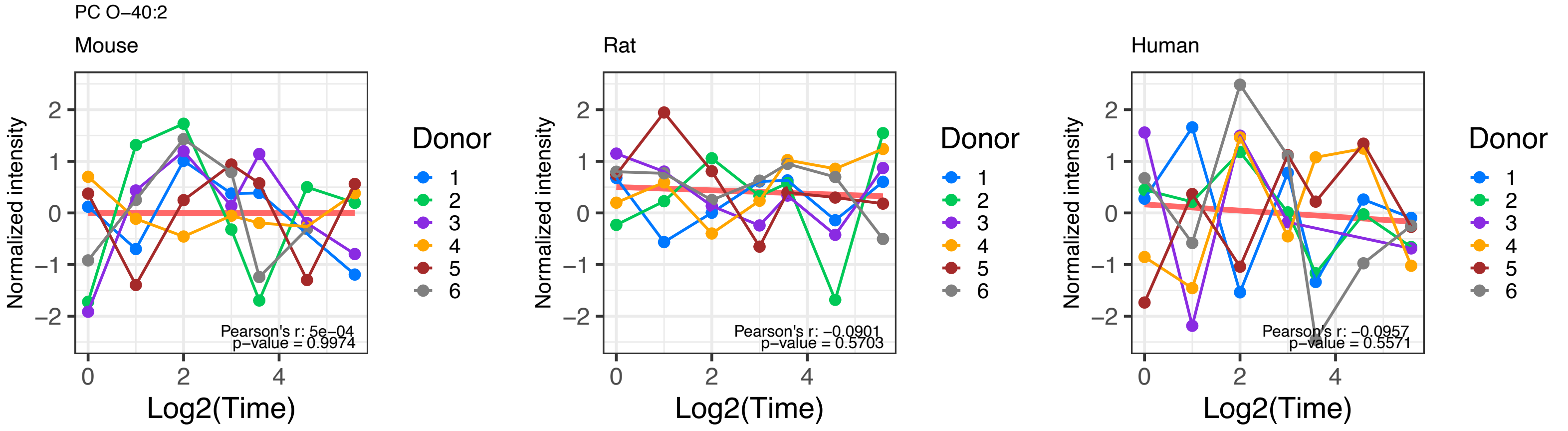
PC O−40:2
Mouse
Rat
Human
Normalized intensity
Log2(Time)
Donor
1
2
3
4
5
6
Pearson's r: 5e−04
p−value = 0.9974
Pearson's r: −0.0901
p−value = 0.5703
Pearson's r: −0.0957
p−value = 0.5571

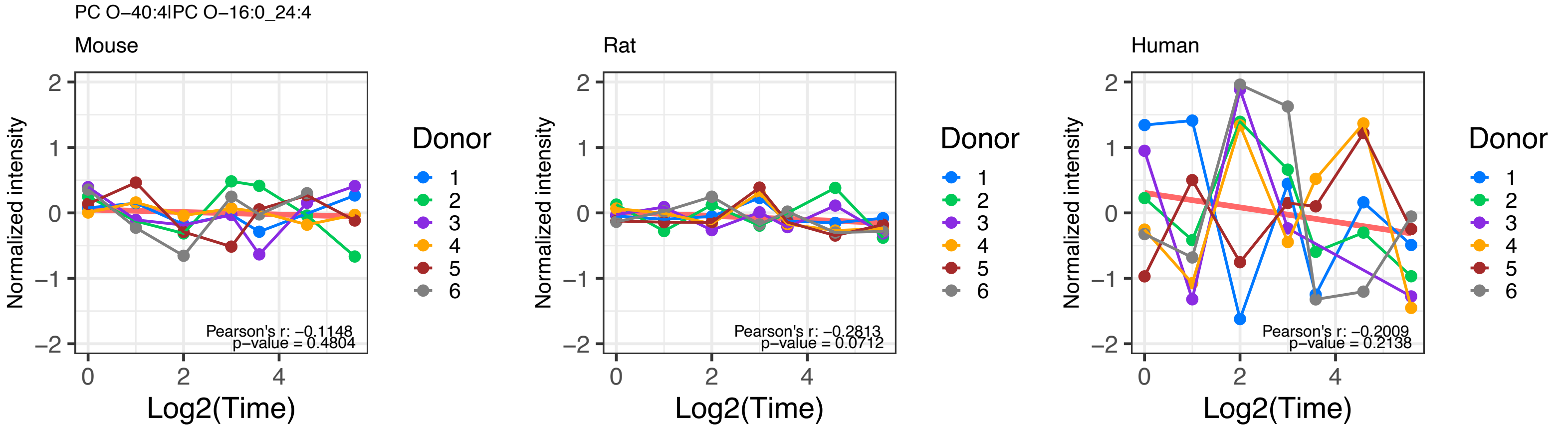
PC O−40:4|PC O−16:0_24:4
Mouse
Rat
Human
Normalized intensity
Log2(Time)
Donor
1
2
3
4
5
6
Pearson's r: −0.1148
p−value = 0.4804
Pearson's r: −0.2813
p−value = 0.0712
Pearson's r: −0.2009
p−value = 0.2138

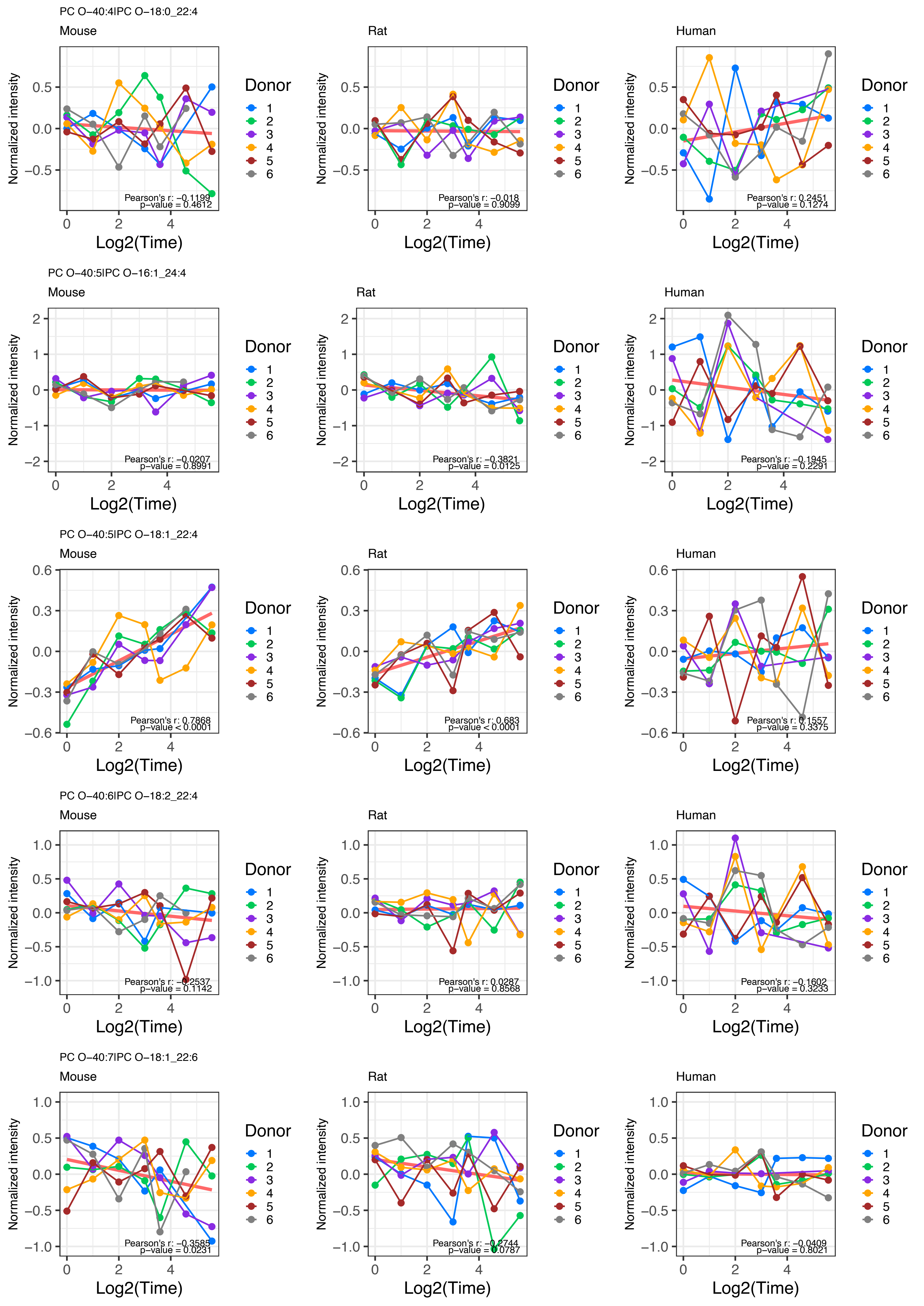

PC O−40:4|PC O−18:0_22:4
Mouse
Rat
Human
Normalized intensity
Log2(Time)
Donor
1
2
3
4
5
6
Pearson's r: −0.1199
p−value = 0.4612
Pearson's r: −0.018
p−value = 0.9099
Pearson's r: 0.2451
p−value = 0.1274
PC O−40:5|PC O−16:1_24:4
Pearson's r: −0.0207
p−value = 0.8991
Pearson's r: −0.3821
p−value = 0.0125
Pearson's r: −0.1945
p−value = 0.2291
PC O−40:5|PC O−18:1_22:4
Pearson's r: 0.7868
p−value < 0.0001
Pearson's r: 0.683
p−value < 0.0001
Pearson's r: 0.1557
p−value = 0.3375
PC O−40:6|PC O−18:2_22:4
Pearson's r: −0.2537
p−value = 0.1142
Pearson's r: 0.0287
p−value = 0.8568
Pearson's r: −0.1602
p−value = 0.3233
PC O−40:7|PC O−18:1_22:6
Pearson's r: −0.3585
p−value = 0.0231
Pearson's r: −0.2744
p−value = 0.0787
Pearson's r: −0.0409
p−value = 0.8021

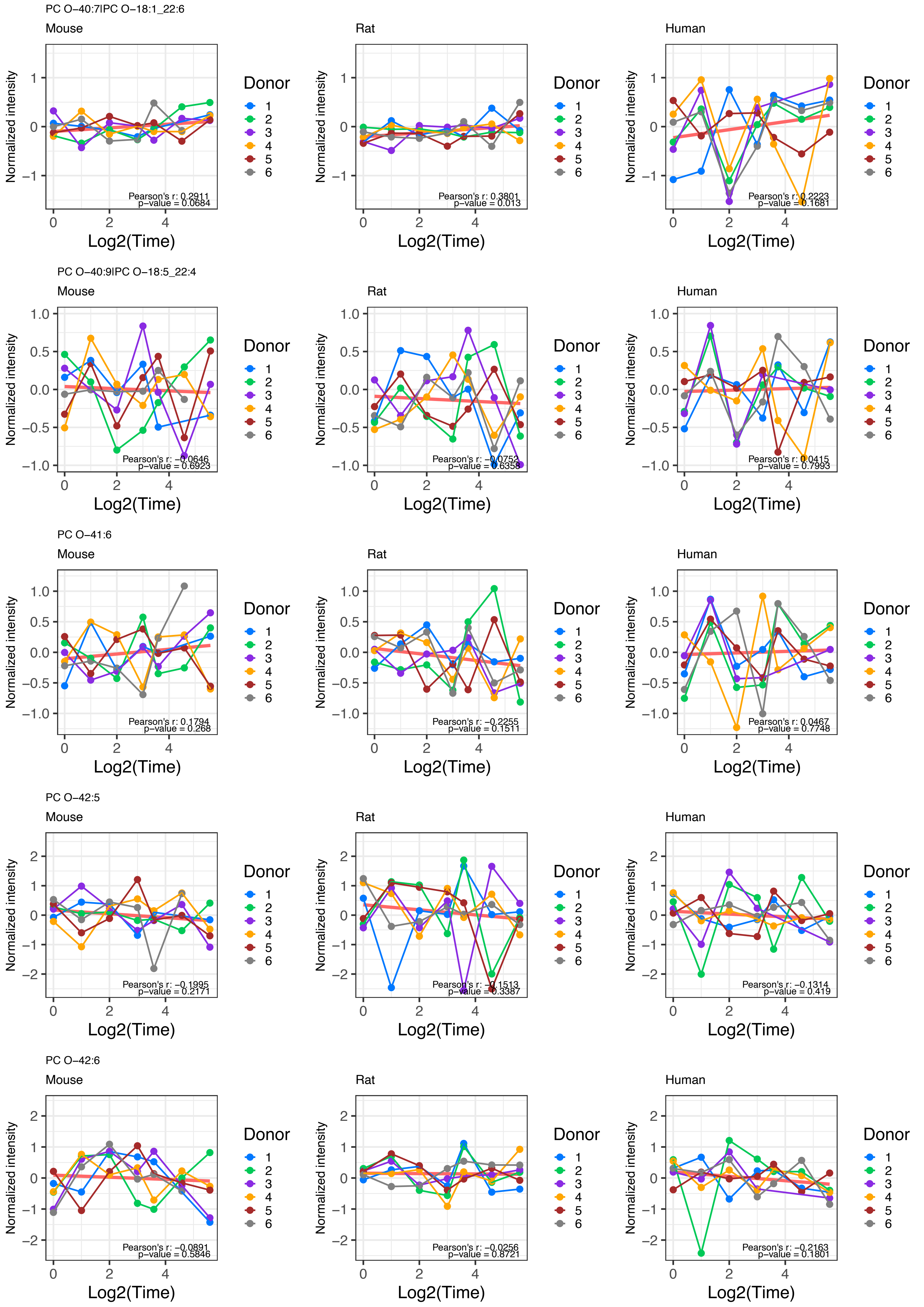
PC O−40:7|PC O−18:1_22:6
Mouse
Pearson's r: 0.2911
p−value = 0.0684
Rat
Pearson's r: 0.3801
p−value = 0.013
Human
Pearson's r: 0.2223
p−value = 0.1681
PC O−40:9|PC O−18:5_22:4
Mouse
Pearson's r: −0.0646
p−value = 0.6923
Rat
Pearson's r: −0.0752
p−value = 0.6358
Human
Pearson's r: 0.0415
p−value = 0.7993
PC O−41:6
Mouse
Pearson's r: 0.1794
p−value = 0.268
Rat
Pearson's r: −0.2255
p−value = 0.1511
Human
Pearson's r: 0.0467
p−value = 0.7748
PC O−42:5
Mouse
Pearson's r: −0.1995
p−value = 0.2171
Rat
Pearson's r: −0.1513
p−value = 0.3387
Human
Pearson's r: −0.1314
p−value = 0.419
PC O−42:6
Mouse
Pearson's r: −0.0891
p−value = 0.5846
Rat
Pearson's r: −0.0256
p−value = 0.8721
Human
Pearson's r: −0.2163
p−value = 0.1801
Normalized intensity
Log2(Time)
Donor
1
2
3
4
5
6

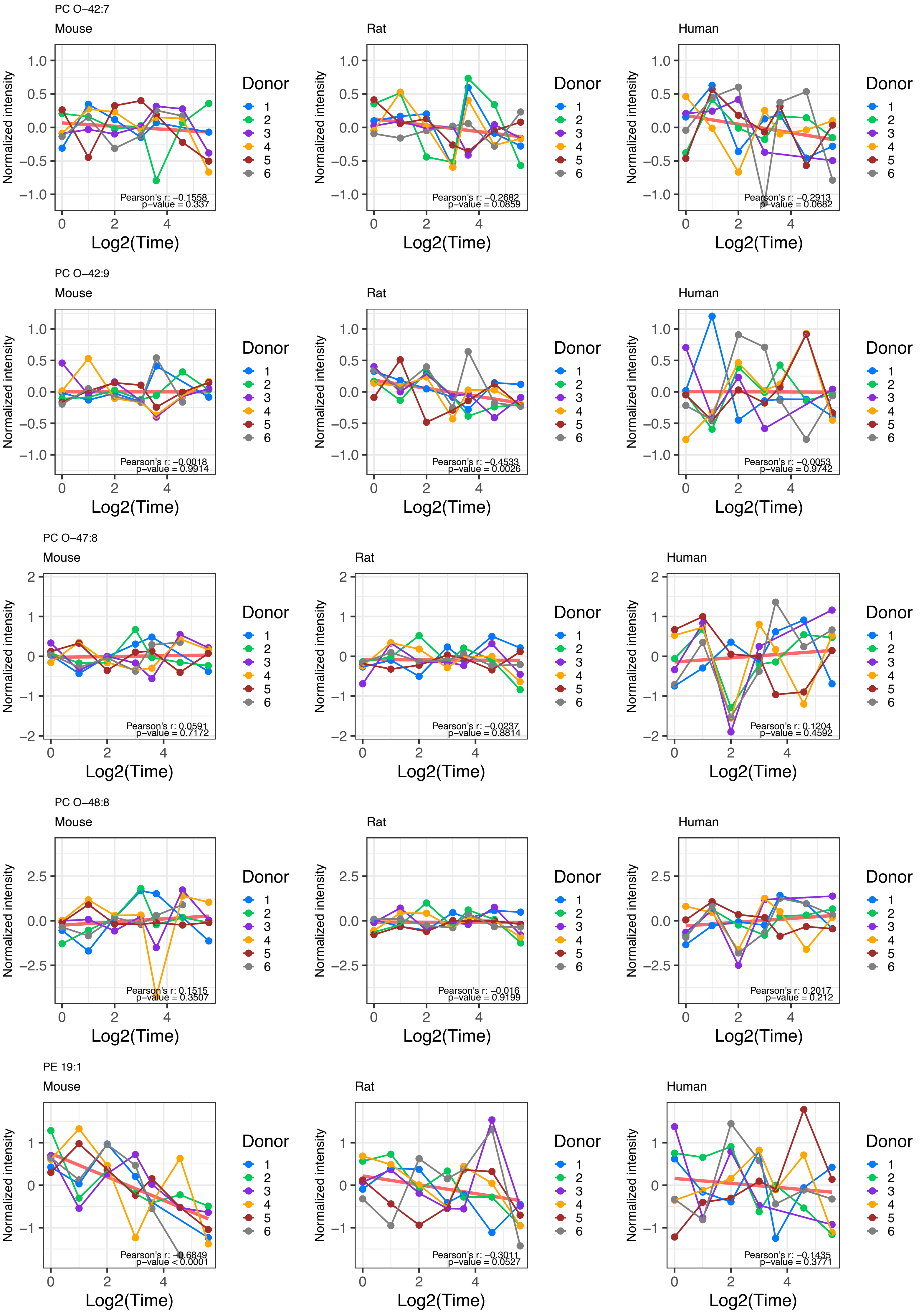
PC O−42:7
Mouse
Pearson's r: −0.1558
p−value = 0.337
Rat
Pearson's r: −0.2682
p−value = 0.0859
Human
Pearson's r: −0.2913
p−value = 0.0682
PC O−42:9
Mouse
Pearson's r: −0.0018
p−value = 0.9914
Rat
Pearson's r: −0.4533
p−value = 0.0026
Human
Pearson's r: −0.0053
p−value = 0.9742
PC O−47:8
Mouse
Pearson's r: 0.0591
p−value = 0.7172
Rat
Pearson's r: −0.0237
p−value = 0.8814
Human
Pearson's r: 0.1204
p−value = 0.4592
PC O−48:8
Mouse
Pearson's r: 0.1515
p−value = 0.3507
Rat
Pearson's r: −0.016
p−value = 0.9199
Human
Pearson's r: 0.2017
p−value = 0.212
PE 19:1
Mouse
Pearson's r: −0.6849
p−value < 0.0001
Rat
Pearson's r: −0.3011
p−value = 0.0527
Human
Pearson's r: −0.1435
p−value = 0.3771
Normalized intensity
Log2(Time)
Donor
1
2
3
4
5
6

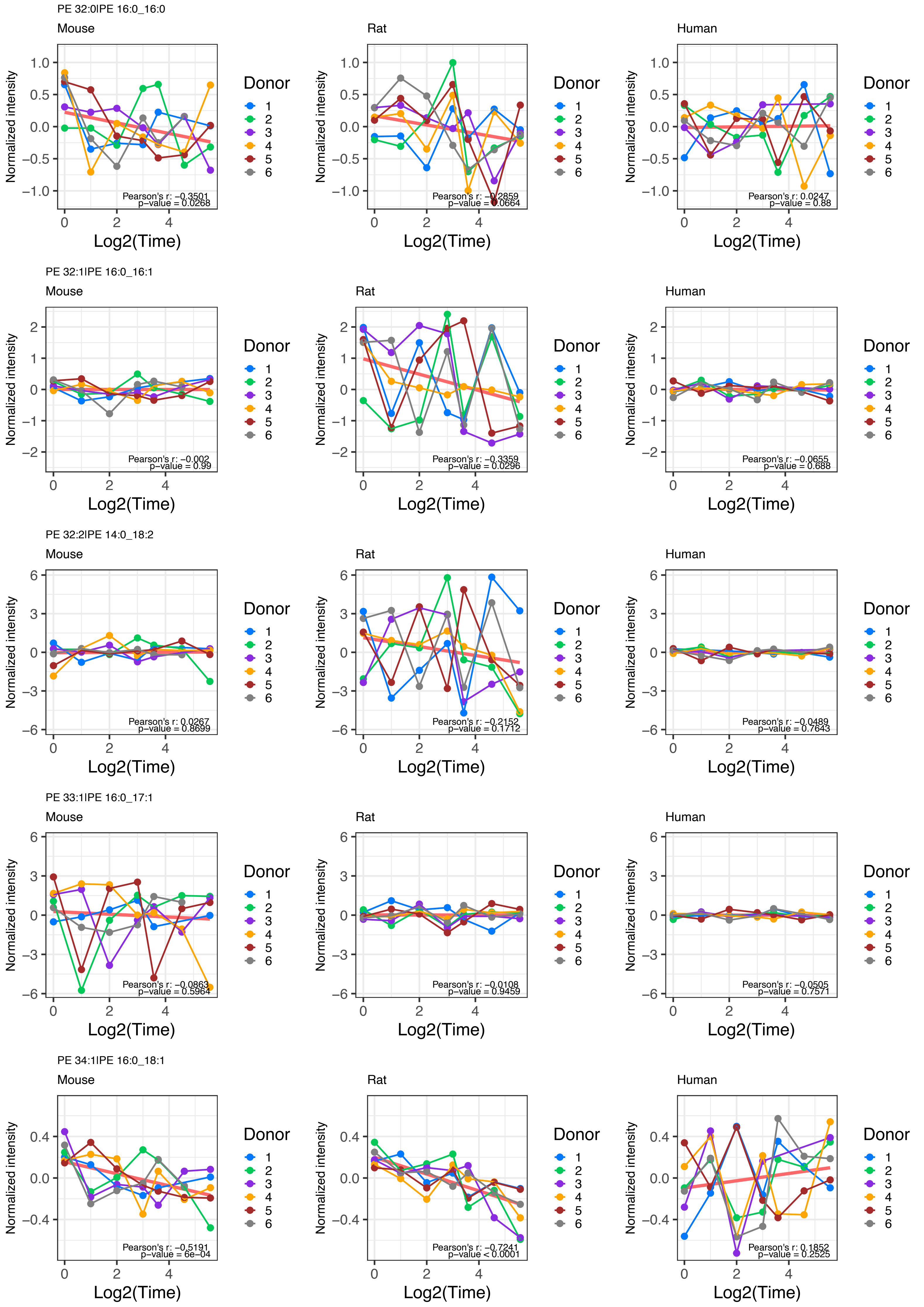
PE 32:0|PE 16:0_16:0
Mouse
Pearson's r: −0.3501
p−value = 0.0268
Rat
Pearson's r: −0.2859
p−value = 0.0664
Human
Pearson's r: 0.0247
p−value = 0.88
PE 32:1|PE 16:0_16:1
Mouse
Pearson's r: −0.002
p−value = 0.99
Rat
Pearson's r: −0.3359
p−value = 0.0296
Human
Pearson's r: −0.0655
p−value = 0.688
PE 32:2|PE 14:0_18:2
Mouse
Pearson's r: 0.0267
p−value = 0.8699
Rat
Pearson's r: −0.2152
p−value = 0.1712
Human
Pearson's r: −0.0489
p−value = 0.7643
PE 33:1|PE 16:0_17:1
Mouse
Pearson's r: −0.0863
p−value = 0.5964
Rat
Pearson's r: −0.0108
p−value = 0.9459
Human
Pearson's r: −0.0505
p−value = 0.7571
PE 34:1|PE 16:0_18:1
Mouse
Pearson's r: −0.5191
p−value = 6e−04
Rat
Pearson's r: −0.7241
p−value < 0.0001
Human
Pearson's r: 0.1852
p−value = 0.2525
Normalized intensity
Log2(Time)
Donor
1
2
3
4
5
6

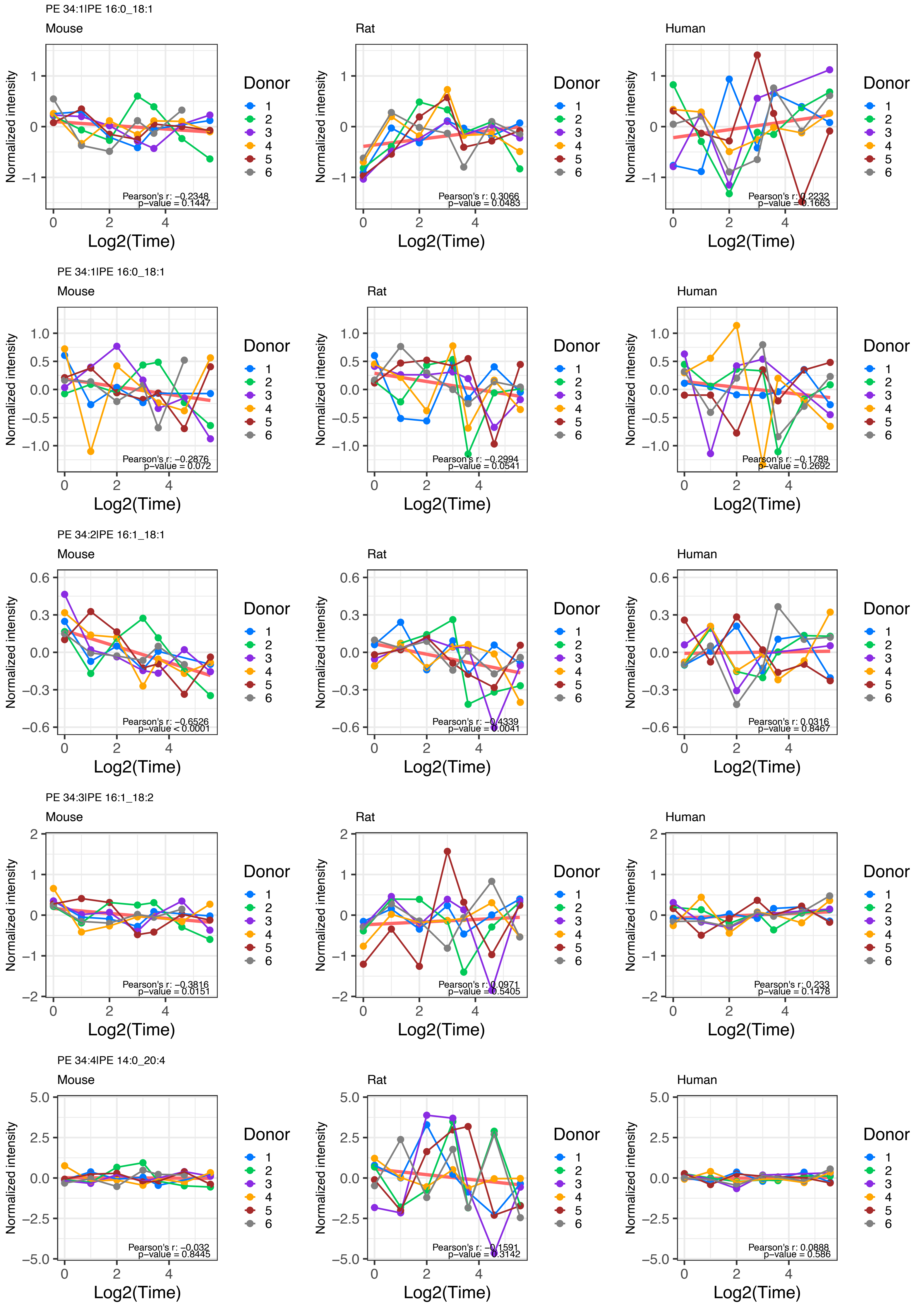

PE 34:1|PE 16:0_18:1
Mouse
Rat
Human
Pearson's r: −0.2348
p−value = 0.1447
Pearson's r: 0.3066
p−value = 0.0483
Pearson's r: 0.2232
p−value = 0.1663
PE 34:1|PE 16:0_18:1
Mouse
Rat
Human
Pearson's r: −0.2876
p−value = 0.072
Pearson's r: −0.2994
p−value = 0.0541
Pearson's r: −0.1789
p−value = 0.2692
PE 34:2|PE 16:1_18:1
Mouse
Rat
Human
Pearson's r: −0.6526
p−value < 0.0001
Pearson's r: −0.4339
p−value = 0.0041
Pearson's r: 0.0316
p−value = 0.8467
PE 34:3|PE 16:1_18:2
Mouse
Rat
Human
Pearson's r: −0.3816
p−value = 0.0151
Pearson's r: 0.0971
p−value = 0.5405
Pearson's r: 0.233
p−value = 0.1478
PE 34:4|PE 14:0_20:4
Mouse
Rat
Human
Pearson's r: −0.032
p−value = 0.8445
Pearson's r: −0.1591
p−value = 0.3142
Pearson's r: 0.0888
p−value = 0.586
Normalized intensity
Log2(Time)
Donor
1
2
3
4
5
6

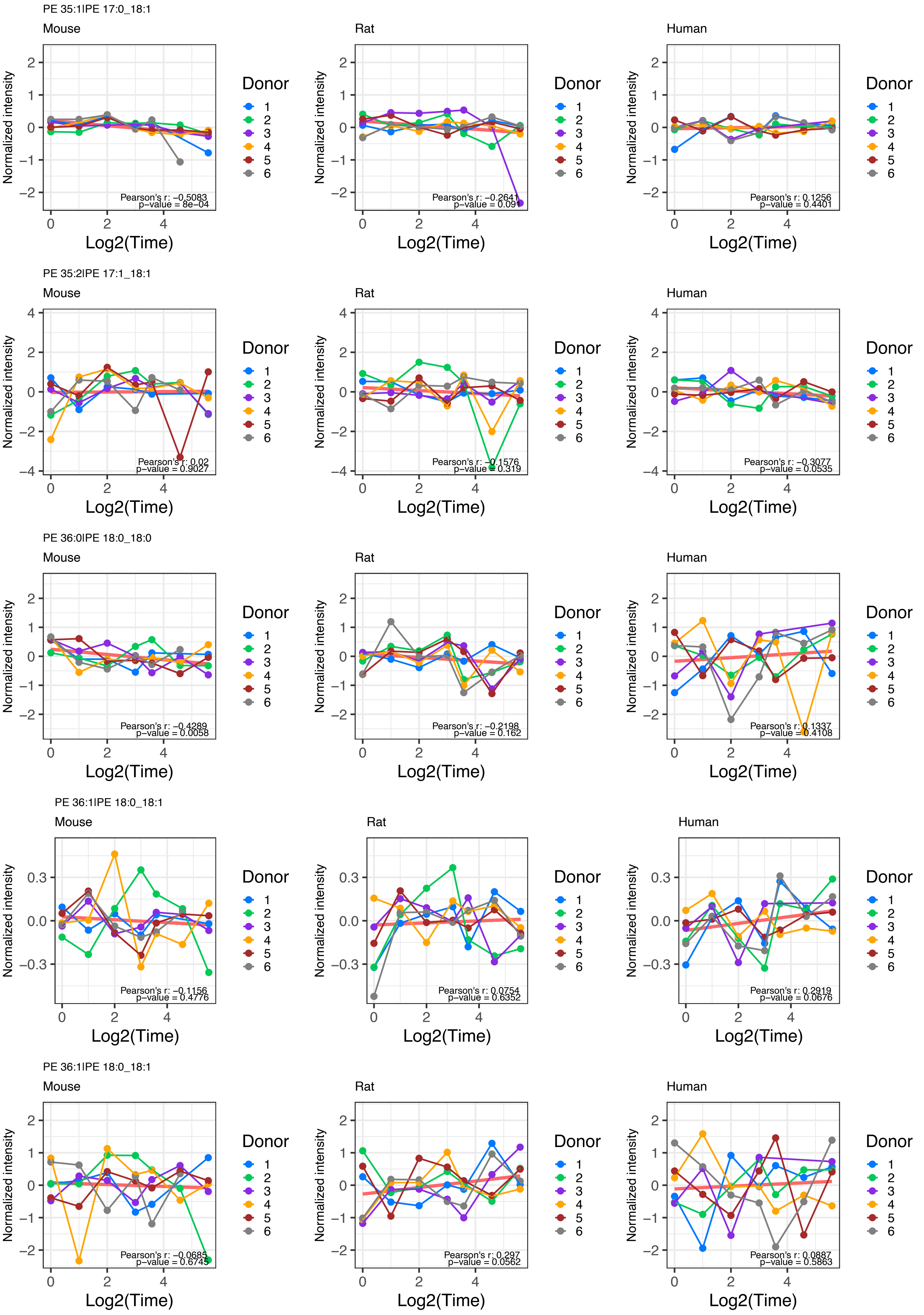

PE 35:1|PE 17:0_18:1
Mouse
Rat
Human
Normalized intensity
Log2(Time)
Donor
1
2
3
4
5
6
Pearson's r: −0.5083
p−value = 8e−04
Pearson's r: −0.2641
p−value = 0.091
Pearson's r: 0.1256
p−value = 0.4401
PE 35:2|PE 17:1_18:1
Mouse
Rat
Human
Pearson's r: 0.02
p−value = 0.9027
Pearson's r: −0.1576
p−value = 0.319
Pearson's r: −0.3077
p−value = 0.0535
PE 36:0|PE 18:0_18:0
Mouse
Rat
Human
Pearson's r: −0.4289
p−value = 0.0058
Pearson's r: −0.2198
p−value = 0.162
Pearson's r: 0.1337
p−value = 0.4108
PE 36:1|PE 18:0_18:1
Mouse
Rat
Human
Pearson's r: −0.1156
p−value = 0.4776
Pearson's r: 0.0754
p−value = 0.6352
Pearson's r: 0.2919
p−value = 0.0676
PE 36:1|PE 18:0_18:1
Mouse
Rat
Human
Pearson's r: −0.0685
p−value = 0.6745
Pearson's r: 0.297
p−value = 0.0562
Pearson's r: 0.0887
p−value = 0.5863

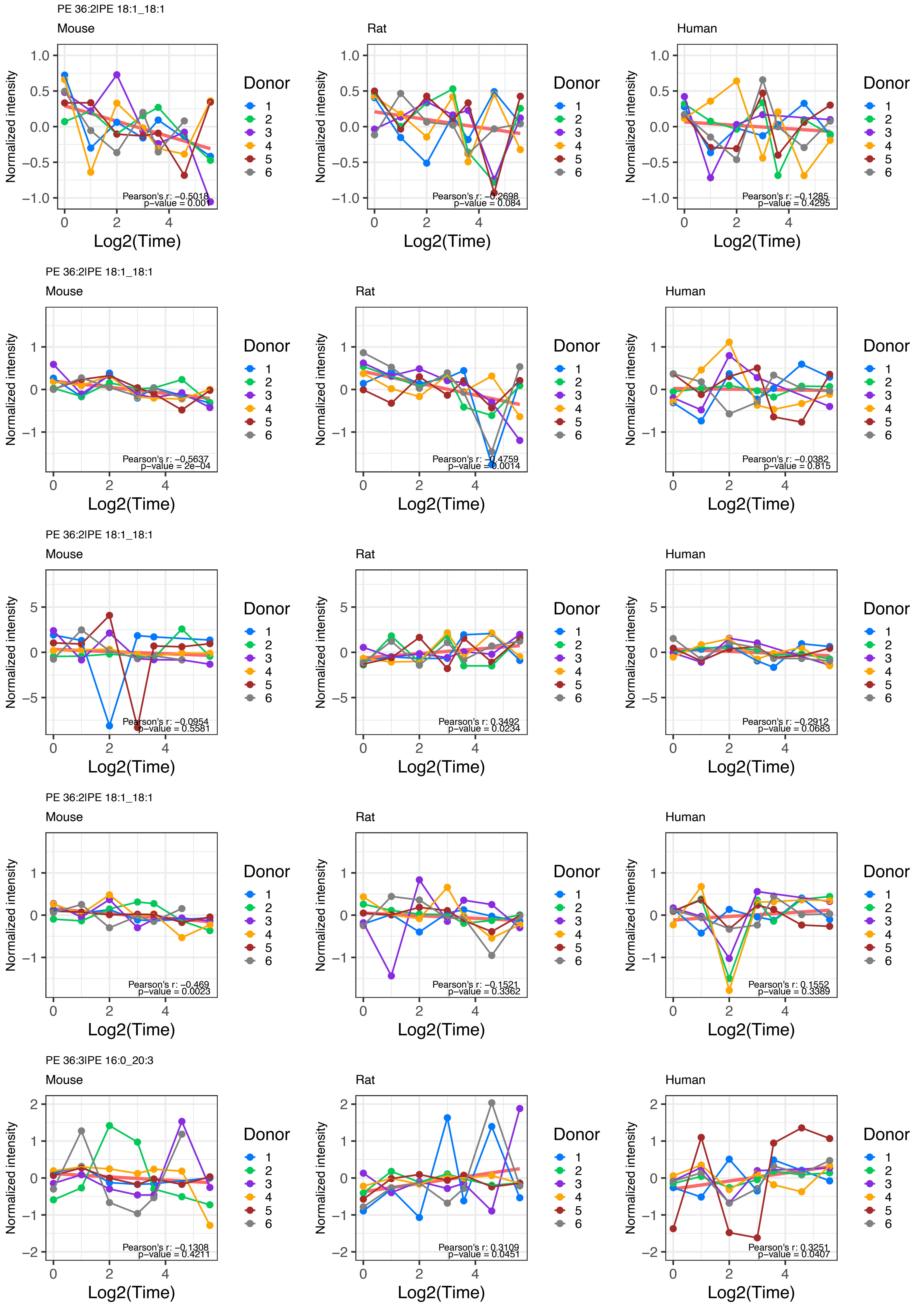

PE 36:2|PE 18:1_18:1
Mouse
Pearson's r: −0.5018
p−value = 0.001
Rat
Pearson's r: −0.2698
p−value = 0.084
Human
Pearson's r: −0.1285
p−value = 0.4295
PE 36:2|PE 18:1_18:1
Mouse
Pearson's r: −0.5637
p−value = 2e−04
Rat
Pearson's r: −0.4759
p−value = 0.0014
Human
Pearson's r: −0.0382
p−value = 0.815
PE 36:2|PE 18:1_18:1
Mouse
Pearson's r: −0.0954
p−value = 0.5581
Rat
Pearson's r: 0.3492
p−value = 0.0234
Human
Pearson's r: −0.2912
p−value = 0.0683
PE 36:2|PE 18:1_18:1
Mouse
Pearson's r: −0.469
p−value = 0.0023
Rat
Pearson's r: −0.1521
p−value = 0.3362
Human
Pearson's r: 0.1552
p−value = 0.3389
PE 36:3|PE 16:0_20:3
Mouse
Pearson's r: −0.1308
p−value = 0.4211
Rat
Pearson's r: 0.3109
p−value = 0.0451
Human
Pearson's r: 0.3251
p−value = 0.0407
Normalized intensity
Log2(Time)
Donor
1
2
3
4
5
6

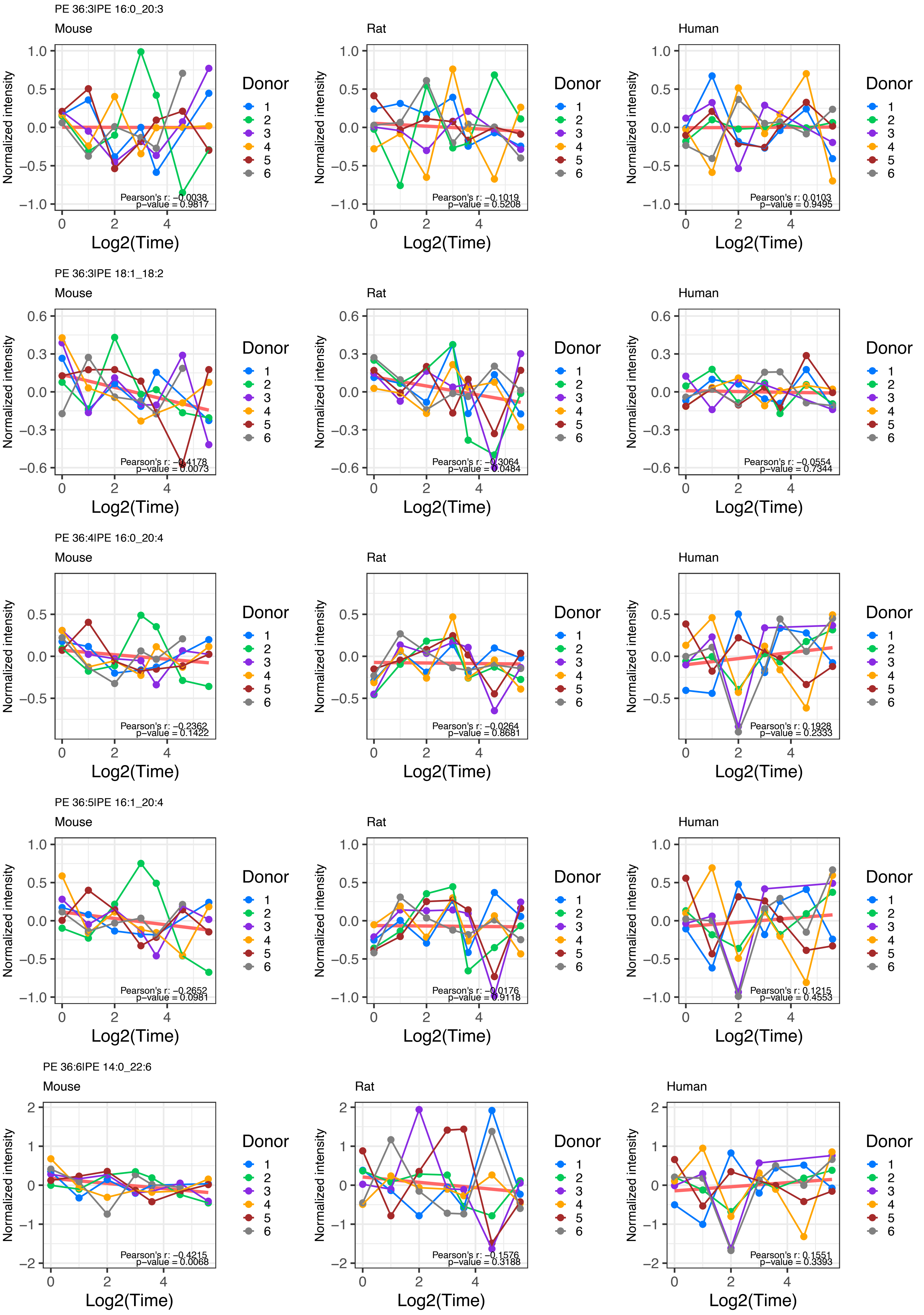
PE 36:3|PE 16:0_20:3
Mouse
Rat
Human
Normalized intensity
Log2(Time)
Donor
1
2
3
4
5
6
Pearson's r: −0.0038
p−value = 0.9817
Pearson's r: −0.1019
p−value = 0.5208
Pearson's r: 0.0103
p−value = 0.9495
PE 36:3|PE 18:1_18:2
Pearson's r: −0.4178
p−value = 0.0073
Pearson's r: −0.3064
p−value = 0.0484
Pearson's r: −0.0554
p−value = 0.7344
PE 36:4|PE 16:0_20:4
Pearson's r: −0.2362
p−value = 0.1422
Pearson's r: −0.0264
p−value = 0.8681
Pearson's r: 0.1928
p−value = 0.2333
PE 36:5|PE 16:1_20:4
Pearson's r: −0.2652
p−value = 0.0981
Pearson's r: −0.0176
p−value = 0.9118
Pearson's r: 0.1215
p−value = 0.4553
PE 36:6|PE 14:0_22:6
Pearson's r: −0.4215
p−value = 0.0068
Pearson's r: −0.1576
p−value = 0.3188
Pearson's r: 0.1551
p−value = 0.3393

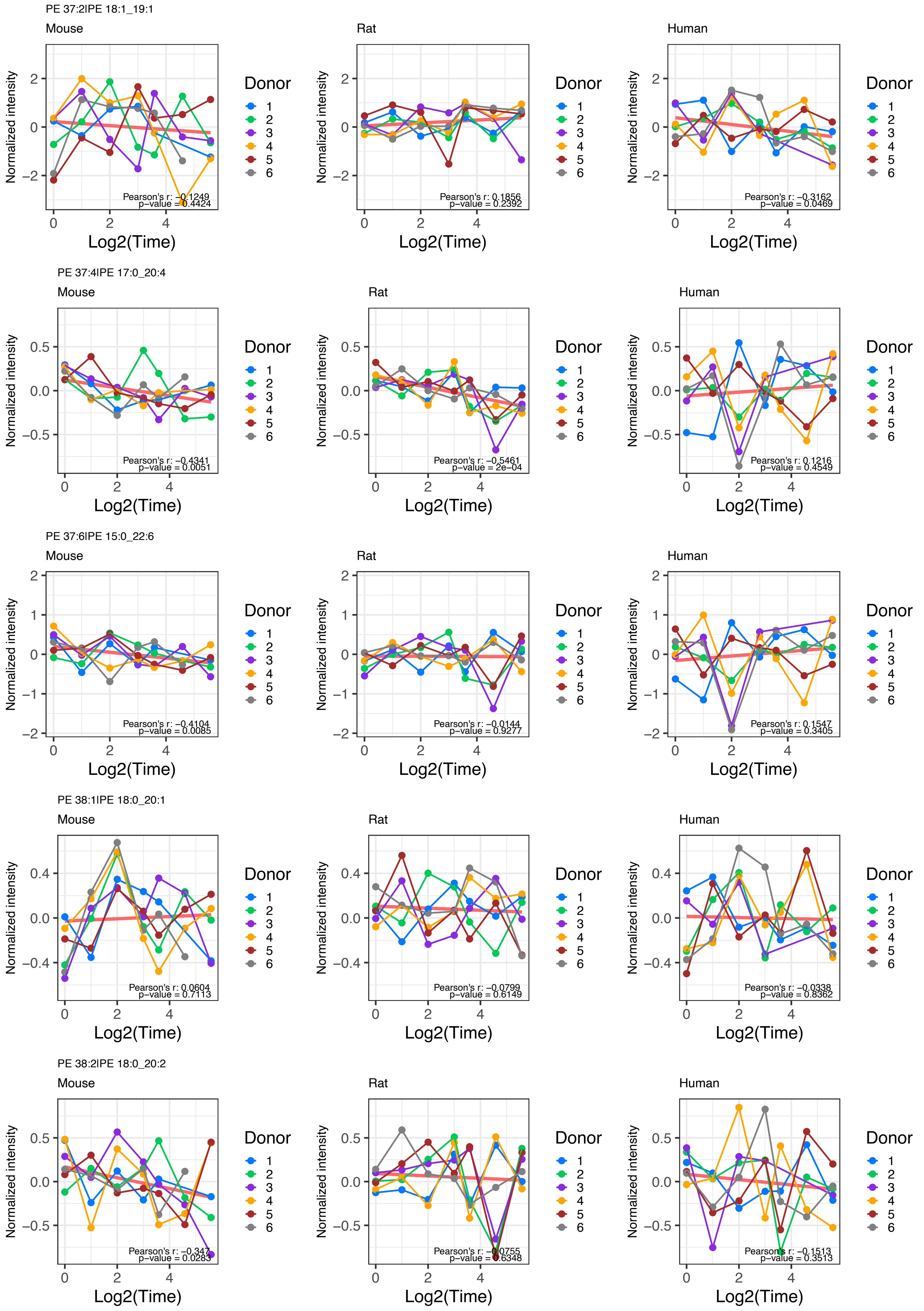

PE 37:2|PE 18:1_19:1
Mouse
Pearson's r: −0.1249
p−value = 0.4424
Rat
Pearson's r: 0.1856
p−value = 0.2392
Human
Pearson's r: −0.3162
p−value = 0.0469
PE 37:4|PE 17:0_20:4
Mouse
Pearson's r: −0.4341
p−value = 0.0051
Rat
Pearson's r: −0.5461
p−value = 2e−04
Human
Pearson's r: 0.1216
p−value = 0.4549
PE 37:6|PE 15:0_22:6
Mouse
Pearson's r: −0.4104
p−value = 0.0085
Rat
Pearson's r: −0.0144
p−value = 0.9277
Human
Pearson's r: 0.1547
p−value = 0.3405
PE 38:1|PE 18:0_20:1
Mouse
Pearson's r: 0.0604
p−value = 0.7113
Rat
Pearson's r: −0.0799
p−value = 0.6149
Human
Pearson's r: −0.0338
p−value = 0.8362
PE 38:2|PE 18:0_20:2
Mouse
Pearson's r: −0.347
p−value = 0.0283
Rat
Pearson's r: −0.0755
p−value = 0.6348
Human
Pearson's r: −0.1513
p−value = 0.3513
Normalized intensity
Log2(Time)
Donor
1
2
3
4
5
6

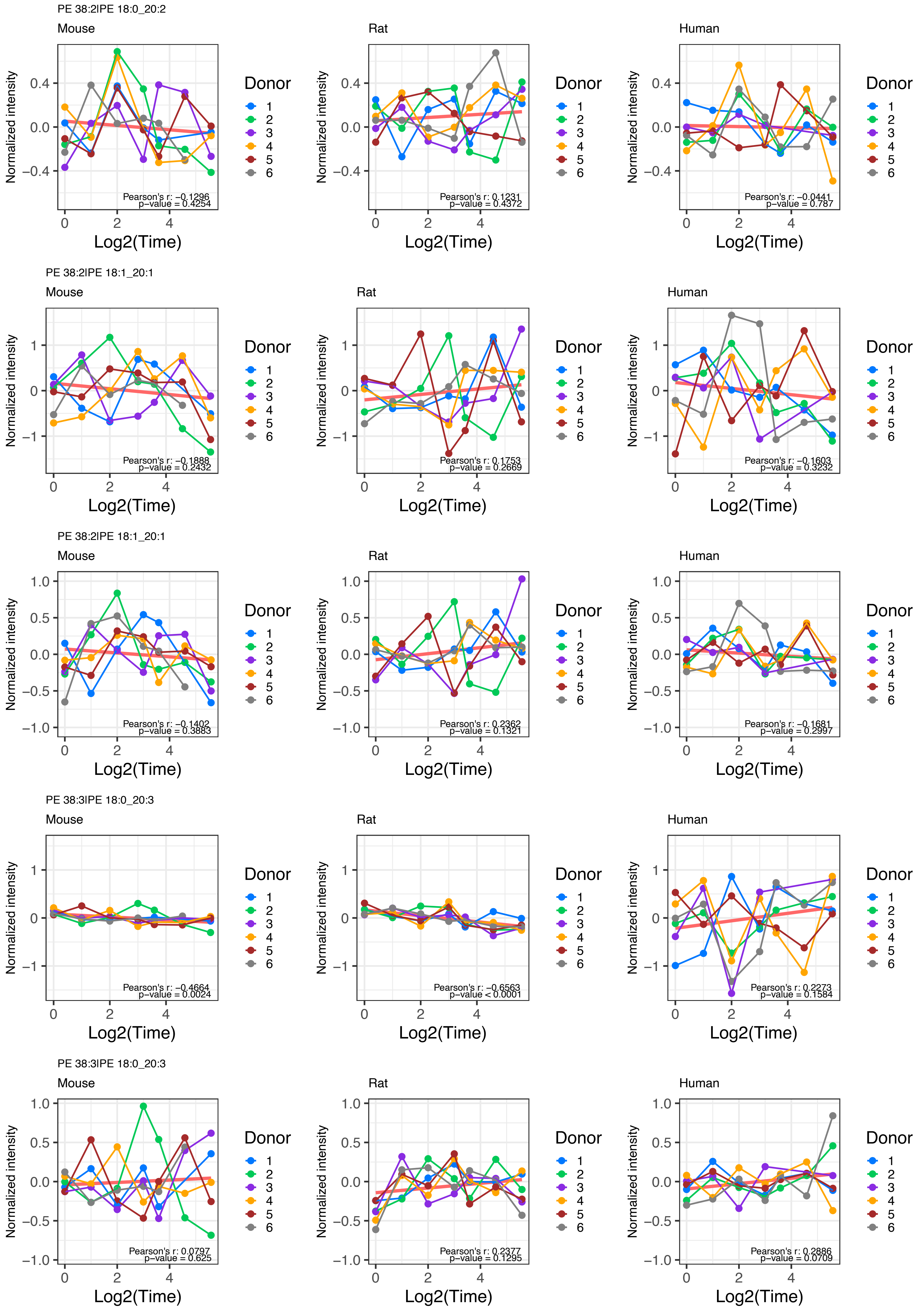
PE 38:2|PE 18:0_20:2
Mouse
Pearson's r: −0.1296
p−value = 0.4254
Rat
Pearson's r: 0.1231
p−value = 0.4372
Human
Pearson's r: −0.0441
p−value = 0.787
PE 38:2|PE 18:1_20:1
Mouse
Pearson's r: −0.1888
p−value = 0.2432
Rat
Pearson's r: 0.1753
p−value = 0.2669
Human
Pearson's r: −0.1603
p−value = 0.3232
PE 38:2|PE 18:1_20:1
Mouse
Pearson's r: −0.1402
p−value = 0.3883
Rat
Pearson's r: 0.2362
p−value = 0.1321
Human
Pearson's r: −0.1681
p−value = 0.2997
PE 38:3|PE 18:0_20:3
Mouse
Pearson's r: −0.4664
p−value = 0.0024
Rat
Pearson's r: −0.6563
p−value < 0.0001
Human
Pearson's r: 0.2273
p−value = 0.1584
PE 38:3|PE 18:0_20:3
Mouse
Pearson's r: 0.0797
p−value = 0.625
Rat
Pearson's r: 0.2377
p−value = 0.1295
Human
Pearson's r: 0.2886
p−value = 0.0709
Normalized intensity
Log2(Time)
Donor
1
2
3
4
5
6

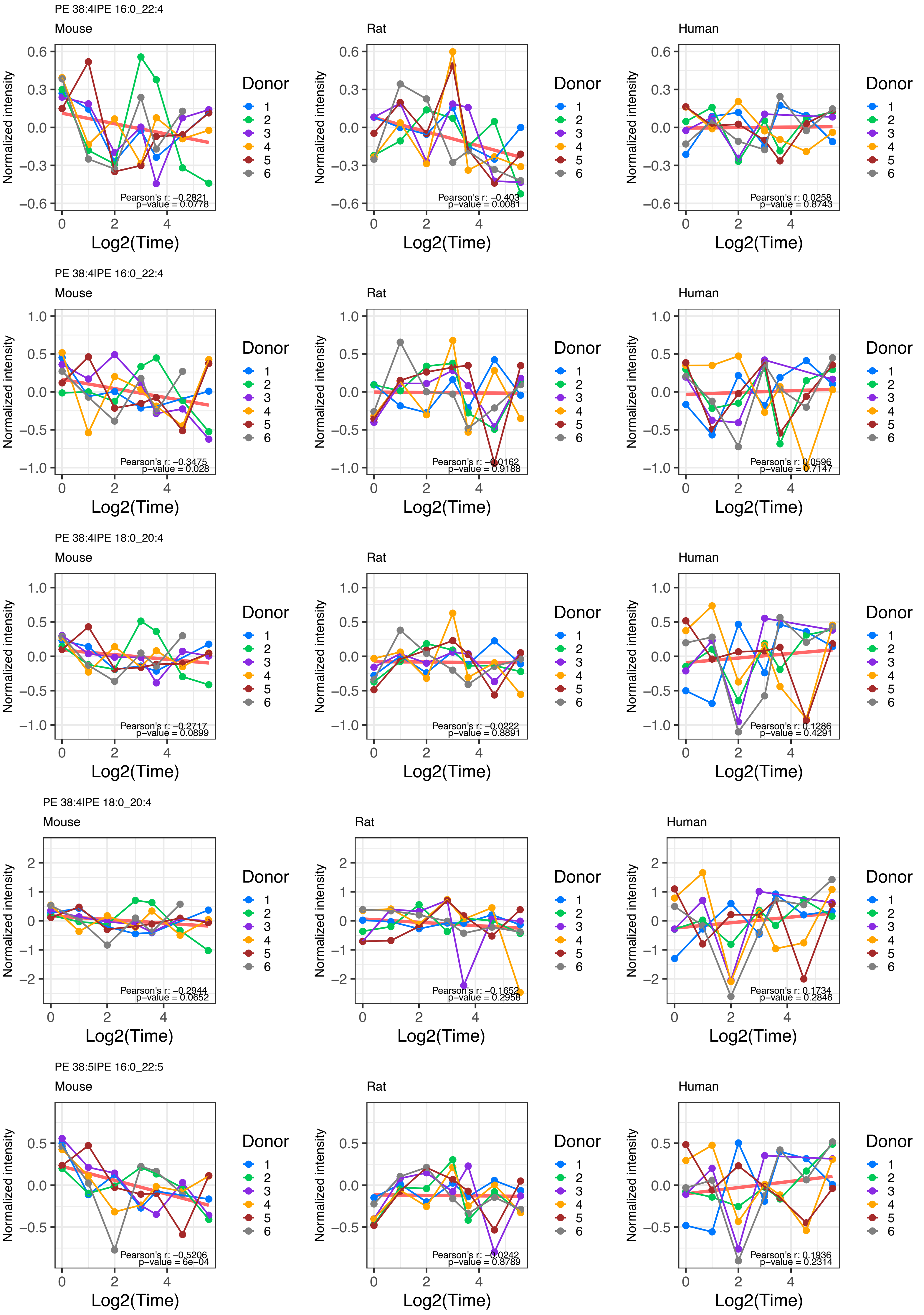

PE 38:4|PE 16:0_22:4
Mouse
Rat
Human
Normalized intensity
Log2(Time)
Donor
1
2
3
4
5
6
Pearson's r: −0.2821
p−value = 0.0778
Pearson's r: −0.403
p−value = 0.0081
Pearson's r: 0.0258
p−value = 0.8743
PE 38:4|PE 16:0_22:4
Mouse
Rat
Human
Pearson's r: −0.3475
p−value = 0.028
Pearson's r: −0.0162
p−value = 0.9188
Pearson's r: 0.0596
p−value = 0.7147
PE 38:4|PE 18:0_20:4
Mouse
Rat
Human
Pearson's r: −0.2717
p−value = 0.0899
Pearson's r: −0.0222
p−value = 0.8891
Pearson's r: 0.1286
p−value = 0.4291
PE 38:4|PE 18:0_20:4
Mouse
Rat
Human
Pearson's r: −0.2944
p−value = 0.0652
Pearson's r: −0.1652
p−value = 0.2958
Pearson's r: 0.1734
p−value = 0.2846
PE 38:5|PE 16:0_22:5
Mouse
Rat
Human
Pearson's r: −0.5206
p−value = 6e−04
Pearson's r: −0.0242
p−value = 0.8789
Pearson's r: 0.1936
p−value = 0.2314

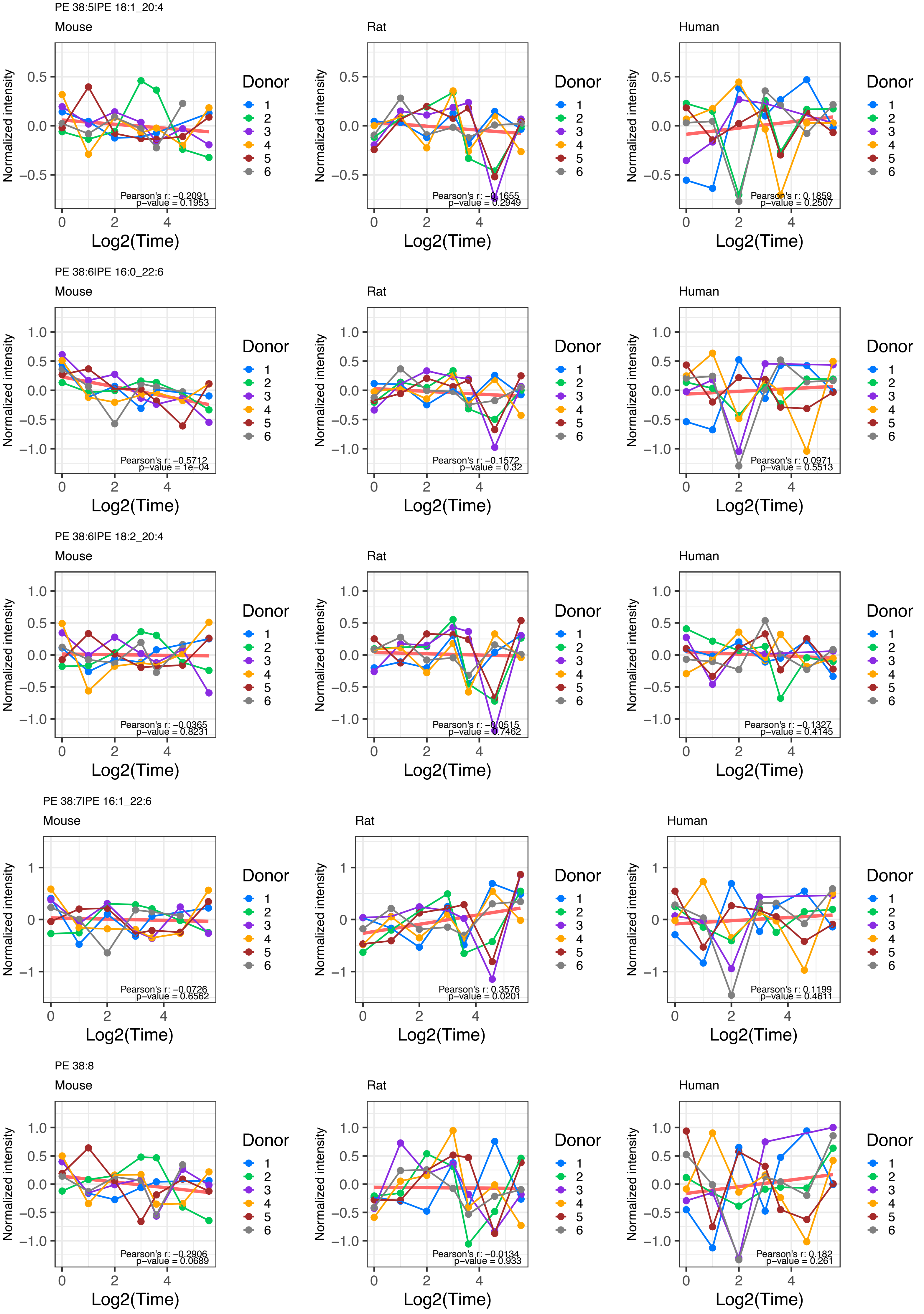

PE 38:5|PE 18:1_20:4
Mouse
Pearson's r: −0.2091
p−value = 0.1953
Rat
Pearson's r: −0.1655
p−value = 0.2949
Human
Pearson's r: 0.1859
p−value = 0.2507
PE 38:6|PE 16:0_22:6
Mouse
Pearson's r: −0.5712
p−value = 1e−04
Rat
Pearson's r: −0.1572
p−value = 0.32
Human
Pearson's r: 0.0971
p−value = 0.5513
PE 38:6|PE 18:2_20:4
Mouse
Pearson's r: −0.0365
p−value = 0.8231
Rat
Pearson's r: −0.0515
p−value = 0.7462
Human
Pearson's r: −0.1327
p−value = 0.4145
PE 38:7|PE 16:1_22:6
Mouse
Pearson's r: −0.0726
p−value = 0.6562
Rat
Pearson's r: 0.3576
p−value = 0.0201
Human
Pearson's r: 0.1199
p−value = 0.4611
PE 38:8
Mouse
Pearson's r: −0.2906
p−value = 0.0689
Rat
Pearson's r: −0.0134
p−value = 0.933
Human
Pearson's r: 0.182
p−value = 0.261
Normalized intensity
Log2(Time)
Donor
1
2
3
4
5
6

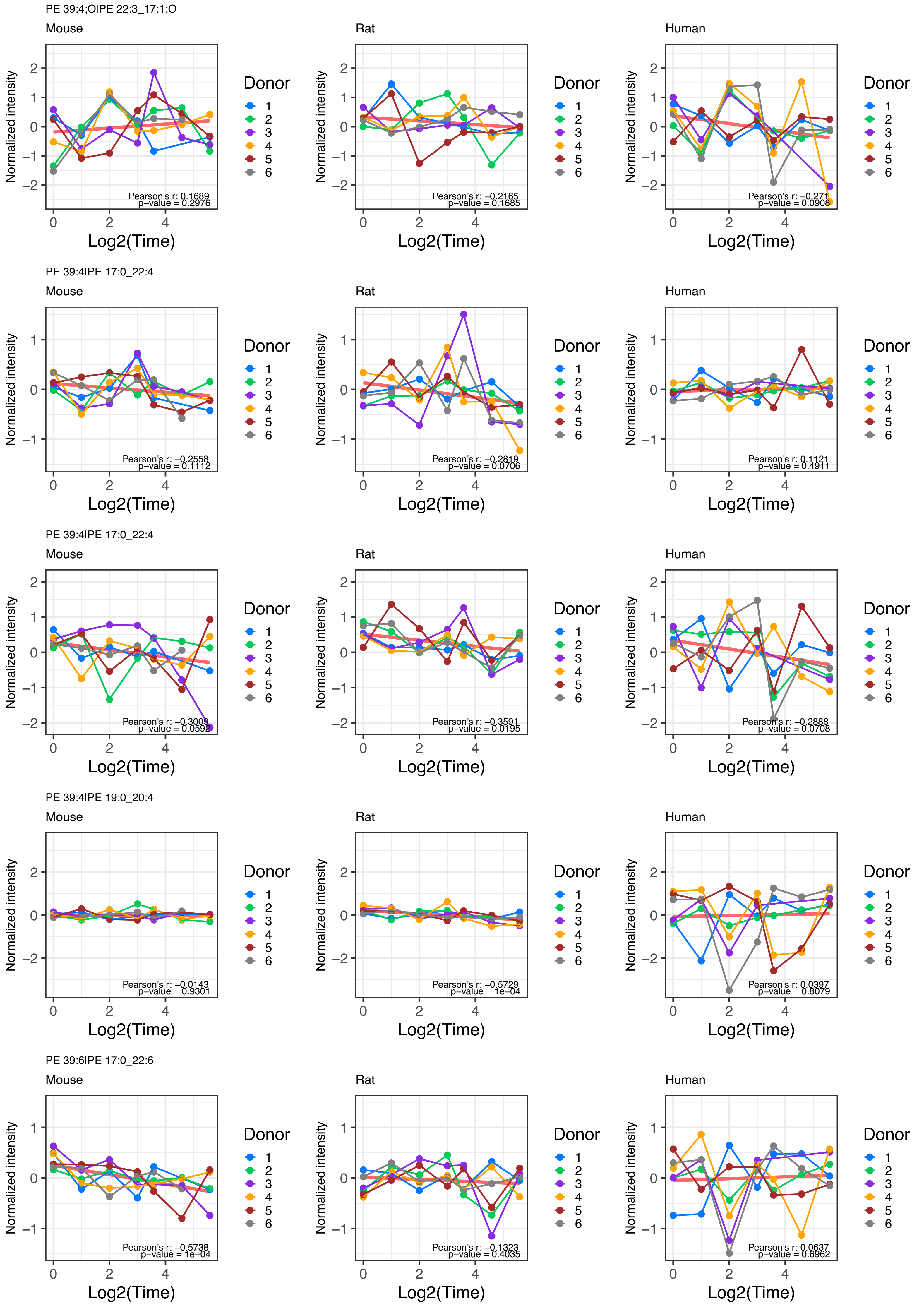
PE 39:4;O|PE 22:3_17:1;O
Mouse
Pearson's r: 0.1689
p−value = 0.2976
Rat
Pearson's r: −0.2165
p−value = 0.1685
Human
Pearson's r: −0.271
p−value = 0.0908
PE 39:4|PE 17:0_22:4
Mouse
Pearson's r: −0.2558
p−value = 0.1112
Rat
Pearson's r: −0.2819
p−value = 0.0706
Human
Pearson's r: 0.1121
p−value = 0.4911
PE 39:4|PE 17:0_22:4
Mouse
Pearson's r: −0.3009
p−value = 0.0592
Rat
Pearson's r: −0.3591
p−value = 0.0195
Human
Pearson's r: −0.2888
p−value = 0.0708
PE 39:4|PE 19:0_20:4
Mouse
Pearson's r: −0.0143
p−value = 0.9301
Rat
Pearson's r: −0.5729
p−value = 1e−04
Human
Pearson's r: 0.0397
p−value = 0.8079
PE 39:6|PE 17:0_22:6
Mouse
Pearson's r: −0.5738
p−value = 1e−04
Rat
Pearson's r: −0.1323
p−value = 0.4035
Human
Pearson's r: 0.0637
p−value = 0.6962
Normalized intensity
Log2(Time)
Donor
1
2
3
4
5
6

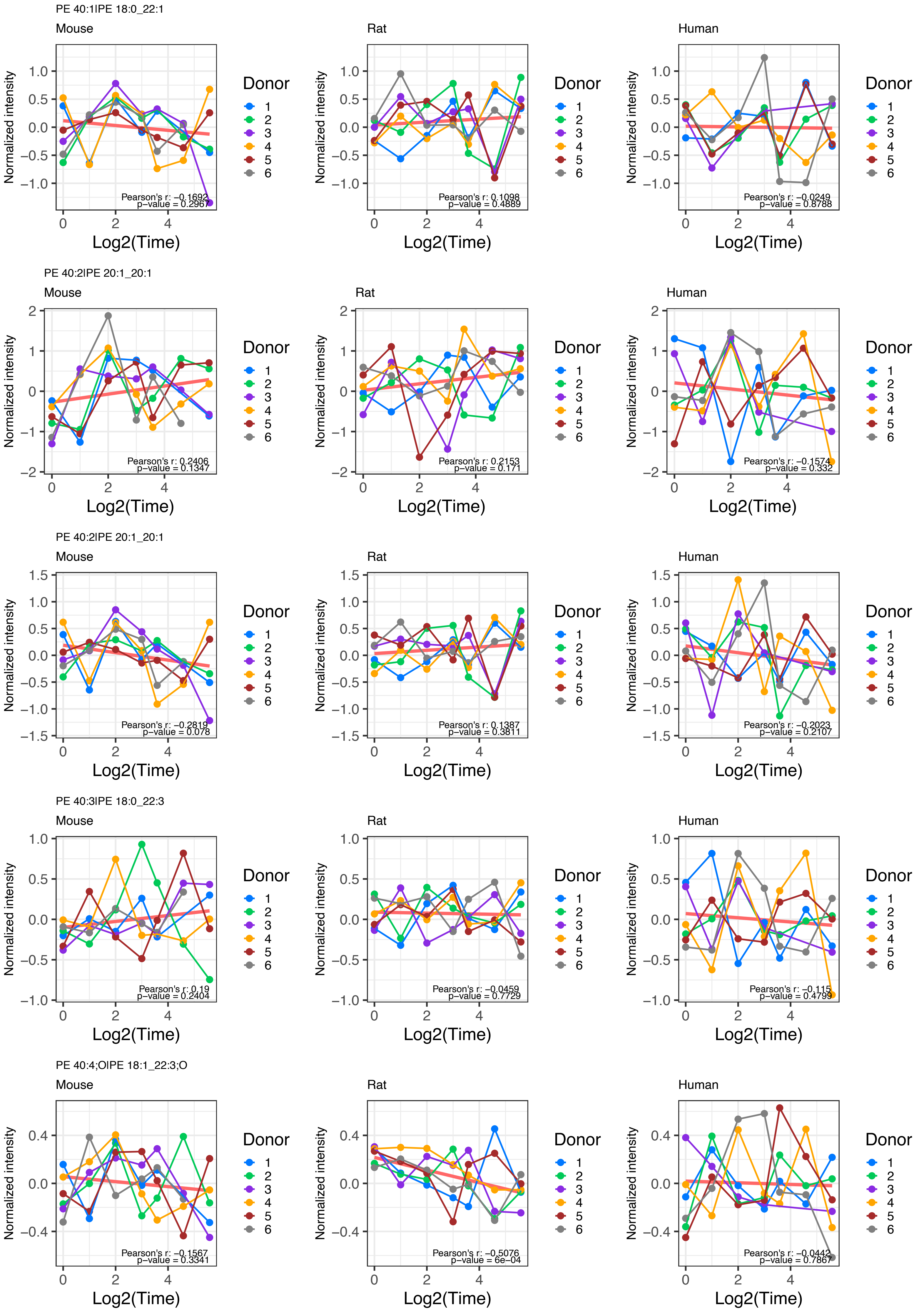

PE 40:1|PE 18:0_22:1
Mouse
Rat
Human
Pearson's r: −0.1692
p−value = 0.2967
Pearson's r: 0.1098
p−value = 0.4889
Pearson's r: −0.0249
p−value = 0.8788
PE 40:2|PE 20:1_20:1
Pearson's r: 0.2406
p−value = 0.1347
Pearson's r: 0.2153
p−value = 0.171
Pearson's r: −0.1574
p−value = 0.332
PE 40:2|PE 20:1_20:1
Pearson's r: −0.2819
p−value = 0.078
Pearson's r: 0.1387
p−value = 0.3811
Pearson's r: −0.2023
p−value = 0.2107
PE 40:3|PE 18:0_22:3
Pearson's r: 0.19
p−value = 0.2404
Pearson's r: −0.0459
p−value = 0.7729
Pearson's r: −0.115
p−value = 0.4799
PE 40:4;O|PE 18:1_22:3;O
Pearson's r: −0.1567
p−value = 0.3341
Pearson's r: −0.5076
p−value = 6e−04
Pearson's r: −0.0442
p−value = 0.7867
Normalized intensity
Log2(Time)
Donor
1
2
3
4
5
6

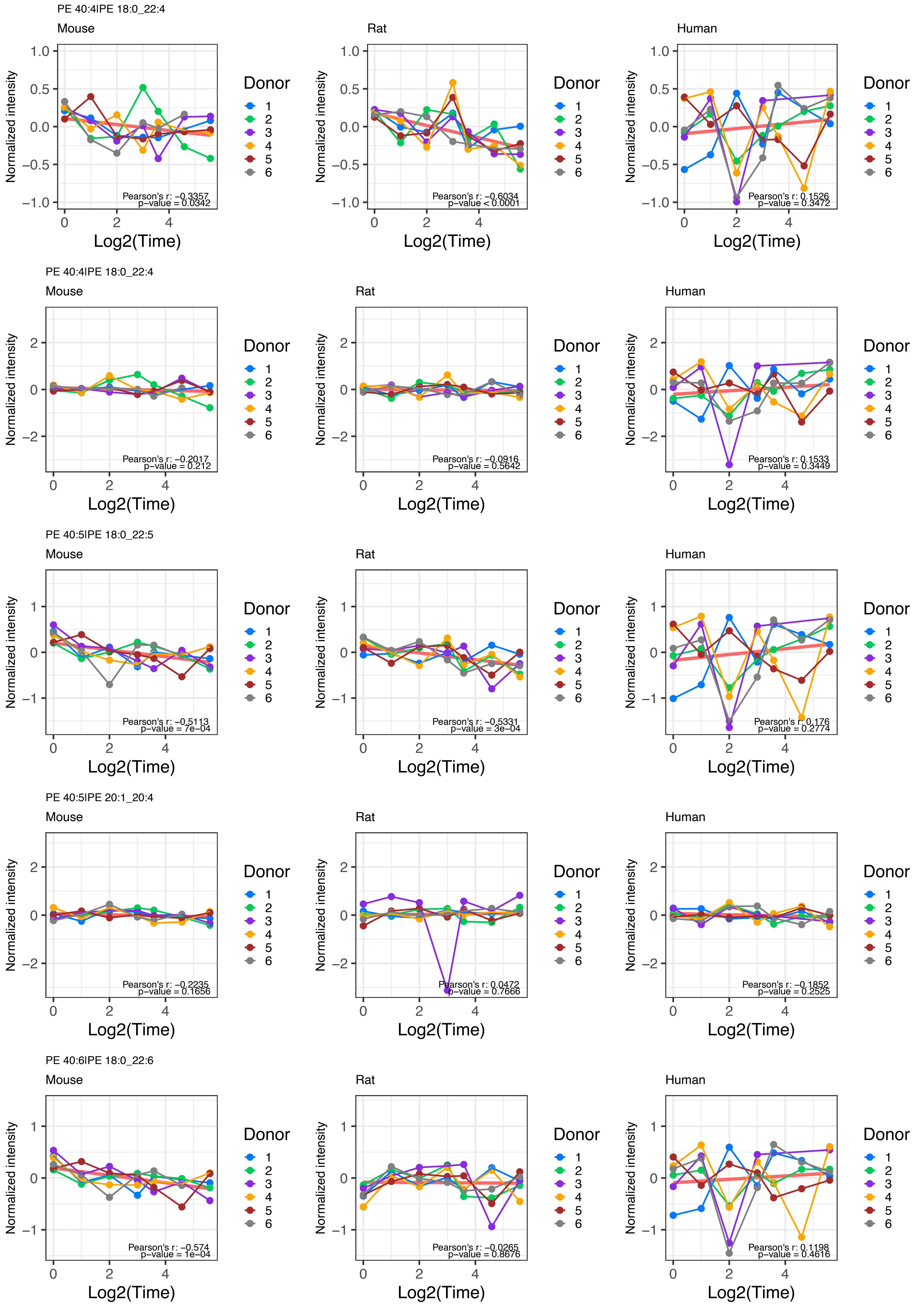
PE 40:4|PE 18:0_22:4
Mouse
Pearson's r: −0.3357
p−value = 0.0342
Rat
Pearson's r: −0.6034
p−value < 0.0001
Human
Pearson's r: 0.1526
p−value = 0.3472
PE 40:4|PE 18:0_22:4
Mouse
Pearson's r: −0.2017
p−value = 0.212
Rat
Pearson's r: −0.0916
p−value = 0.5642
Human
Pearson's r: 0.1533
p−value = 0.3449
PE 40:5|PE 18:0_22:5
Mouse
Pearson's r: −0.5113
p−value = 7e−04
Rat
Pearson's r: −0.5331
p−value = 3e−04
Human
Pearson's r: 0.176
p−value = 0.2774
PE 40:5|PE 20:1_20:4
Mouse
Pearson's r: −0.2235
p−value = 0.1656
Rat
Pearson's r: 0.0472
p−value = 0.7666
Human
Pearson's r: −0.1852
p−value = 0.2525
PE 40:6|PE 18:0_22:6
Mouse
Pearson's r: −0.574
p−value = 1e−04
Rat
Pearson's r: −0.0265
p−value = 0.8676
Human
Pearson's r: 0.1198
p−value = 0.4616
Normalized intensity
Log2(Time)
Donor
1
2
3
4
5
6

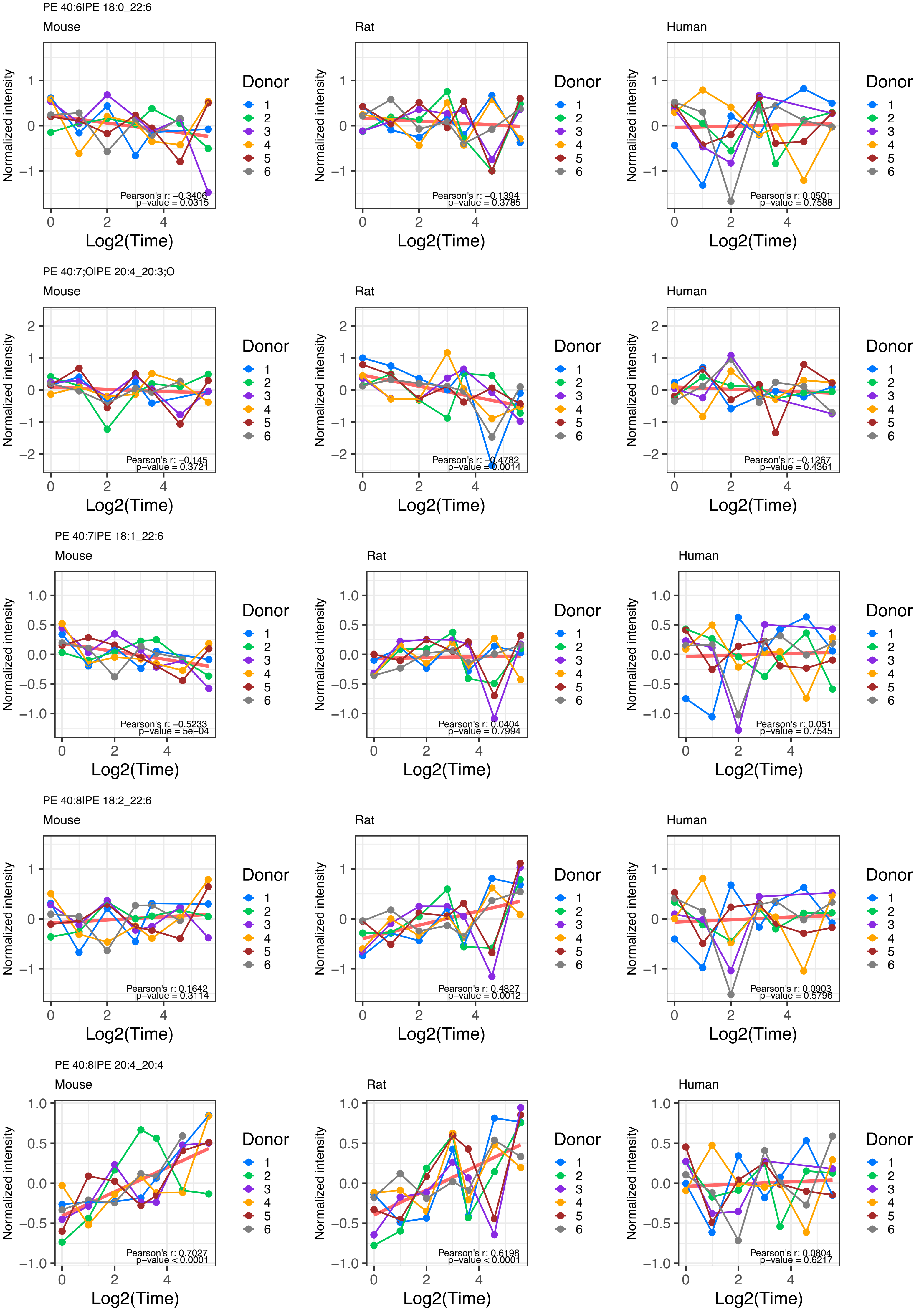

PE 40:6|PE 18:0_22:6
Mouse
Pearson's r: −0.3406
p−value = 0.0315
Rat
Pearson's r: −0.1394
p−value = 0.3785
Human
Pearson's r: 0.0501
p−value = 0.7588
PE 40:7;O|PE 20:4_20:3;O
Mouse
Pearson's r: −0.145
p−value = 0.3721
Rat
Pearson's r: −0.4782
p−value = 0.0014
Human
Pearson's r: −0.1267
p−value = 0.4361
PE 40:7|PE 18:1_22:6
Mouse
Pearson's r: −0.5233
p−value = 5e−04
Rat
Pearson's r: 0.0404
p−value = 0.7994
Human
Pearson's r: 0.051
p−value = 0.7545
PE 40:8|PE 18:2_22:6
Mouse
Pearson's r: 0.1642
p−value = 0.3114
Rat
Pearson's r: 0.4827
p−value = 0.0012
Human
Pearson's r: 0.0903
p−value = 0.5796
PE 40:8|PE 20:4_20:4
Mouse
Pearson's r: 0.7027
p−value < 0.0001
Rat
Pearson's r: 0.6198
p−value < 0.0001
Human
Pearson's r: 0.0804
p−value = 0.6217
Normalized intensity
Log2(Time)
Donor
1
2
3
4
5
6

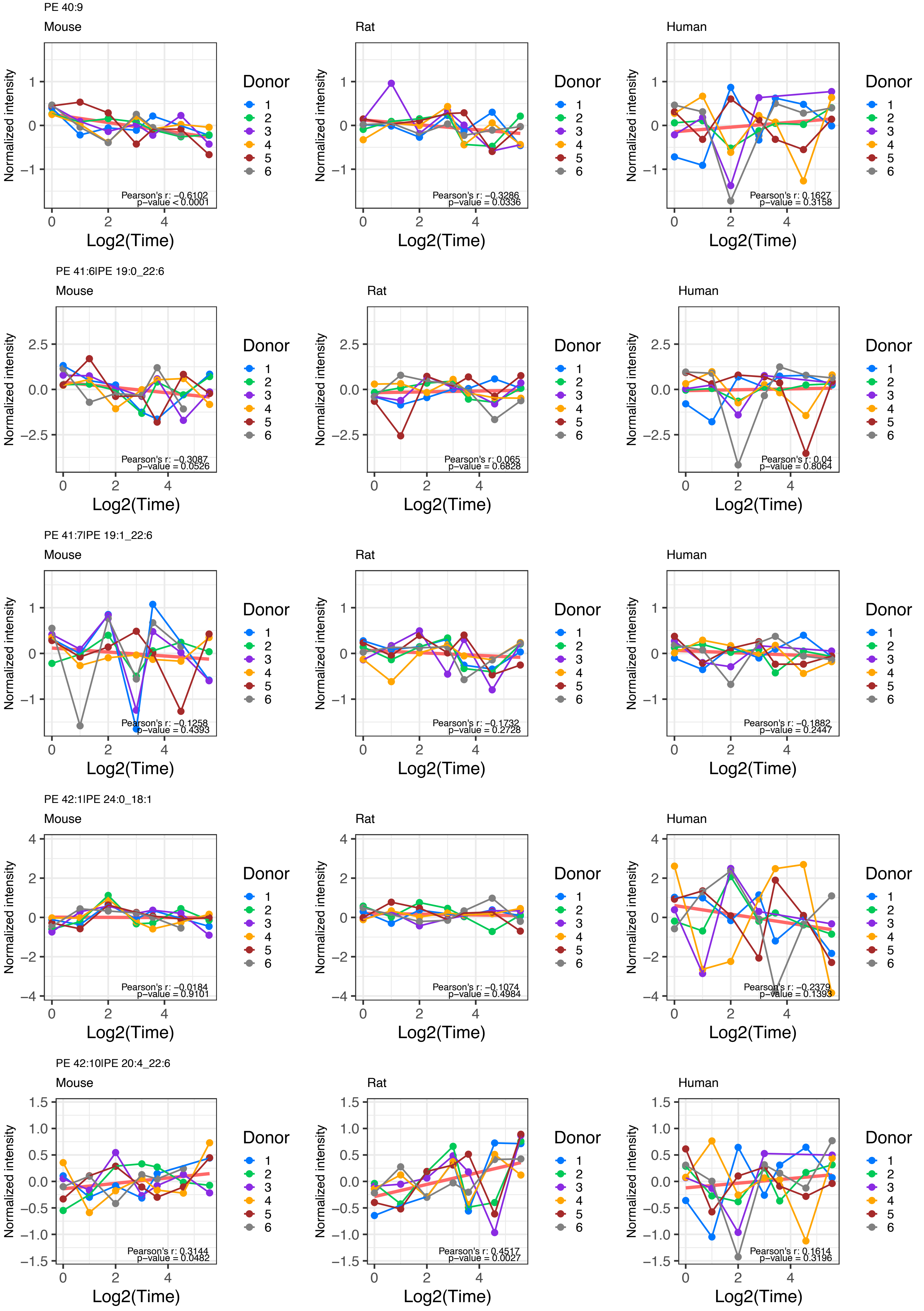
PE 40:9
Mouse
Pearson's r: −0.6102
p−value < 0.0001
Rat
Pearson's r: −0.3286
p−value = 0.0336
Human
Pearson's r: 0.1627
p−value = 0.3158
PE 41:6|PE 19:0_22:6
Mouse
Pearson's r: −0.3087
p−value = 0.0526
Rat
Pearson's r: 0.065
p−value = 0.6828
Human
Pearson's r: 0.04
p−value = 0.8064
PE 41:7|PE 19:1_22:6
Mouse
Pearson's r: −0.1258
p−value = 0.4393
Rat
Pearson's r: −0.1732
p−value = 0.2728
Human
Pearson's r: −0.1882
p−value = 0.2447
PE 42:1|PE 24:0_18:1
Mouse
Pearson's r: −0.0184
p−value = 0.9101
Rat
Pearson's r: −0.1074
p−value = 0.4984
Human
Pearson's r: −0.2379
p−value = 0.1393
PE 42:10|PE 20:4_22:6
Mouse
Pearson's r: 0.3144
p−value = 0.0482
Rat
Pearson's r: 0.4517
p−value = 0.0027
Human
Pearson's r: 0.1614
p−value = 0.3196
Normalized intensity
Log2(Time)
Donor
1
2
3
4
5
6

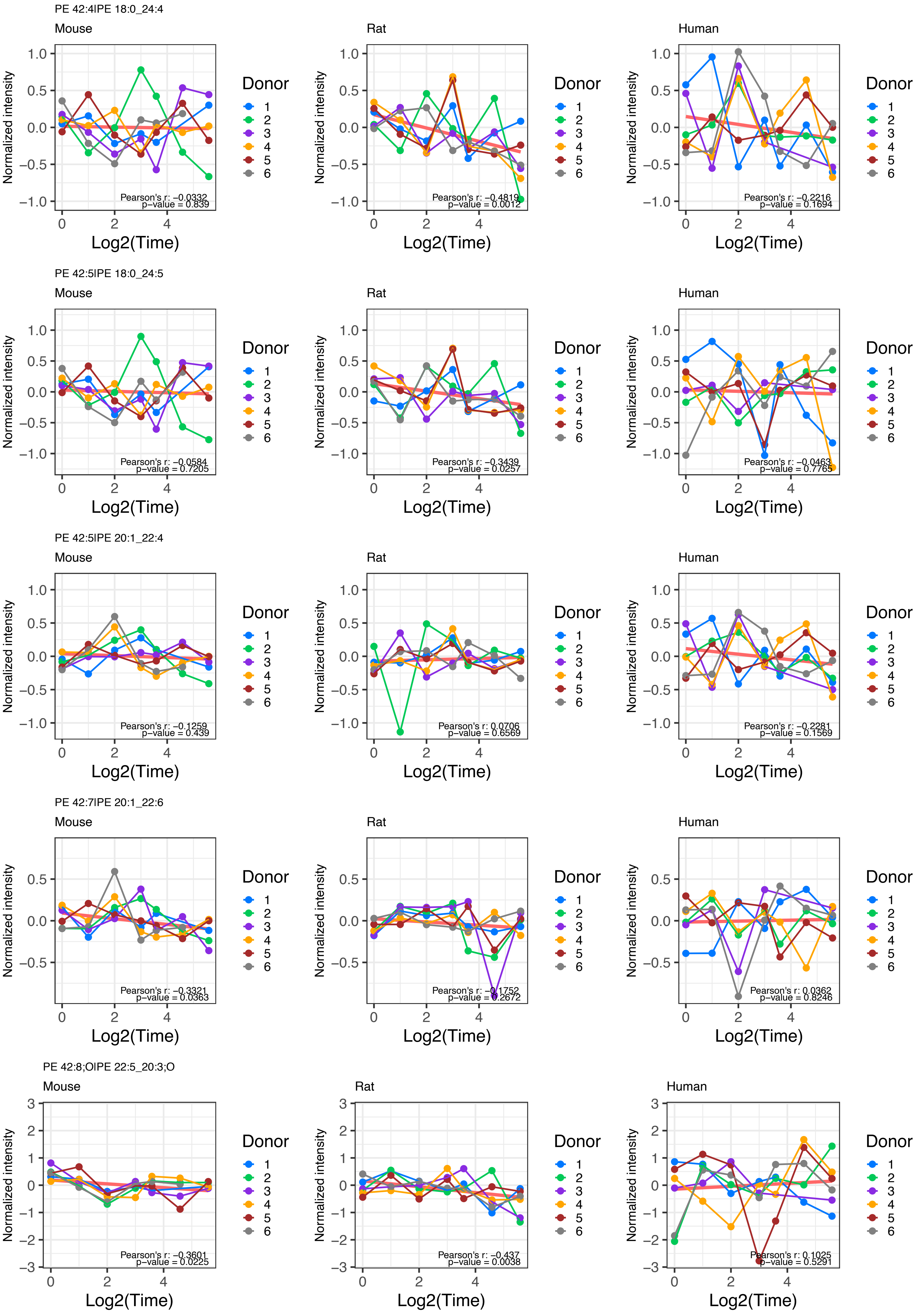

PE 42:4|PE 18:0_24:4
Mouse
Rat
Human
Pearson's r: −0.0332
p−value = 0.839
Pearson's r: −0.4819
p−value = 0.0012
Pearson's r: −0.2216
p−value = 0.1694
PE 42:5|PE 18:0_24:5
Mouse
Rat
Human
Pearson's r: −0.0584
p−value = 0.7205
Pearson's r: −0.3439
p−value = 0.0257
Pearson's r: −0.0463
p−value = 0.7765
PE 42:5|PE 20:1_22:4
Mouse
Rat
Human
Pearson's r: −0.1259
p−value = 0.439
Pearson's r: 0.0706
p−value = 0.6569
Pearson's r: −0.2281
p−value = 0.1569
PE 42:7|PE 20:1_22:6
Mouse
Rat
Human
Pearson's r: −0.3321
p−value = 0.0363
Pearson's r: −0.1752
p−value = 0.2672
Pearson's r: 0.0362
p−value = 0.8246
PE 42:8;O|PE 22:5_20:3;O
Mouse
Rat
Human
Pearson's r: −0.3601
p−value = 0.0225
Pearson's r: −0.437
p−value = 0.0038
Pearson's r: 0.1025
p−value = 0.5291
Normalized intensity
Log2(Time)
Donor
1
2
3
4
5
6

PE 42:8;O2|PE 20:4_22:4;O2

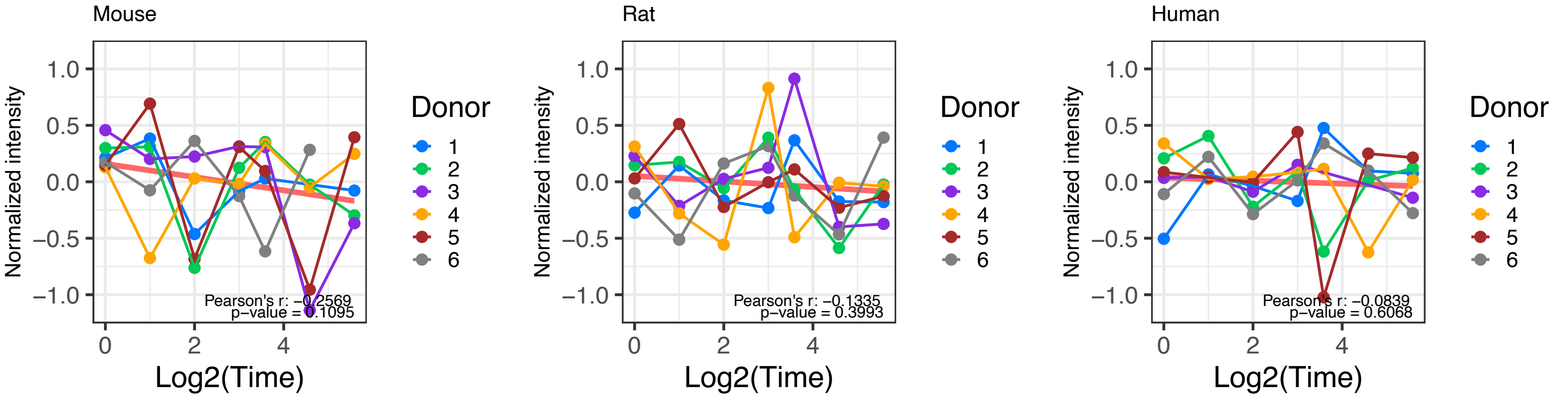

PE 42:8|PE 20:4_22:4

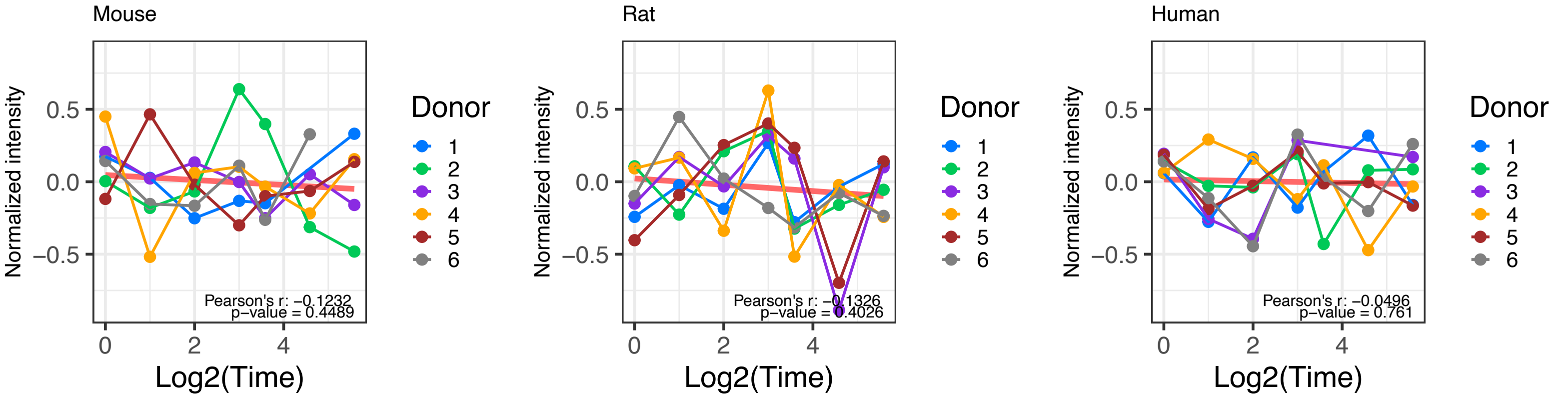

PE 42:9|PE 20:3_22:6

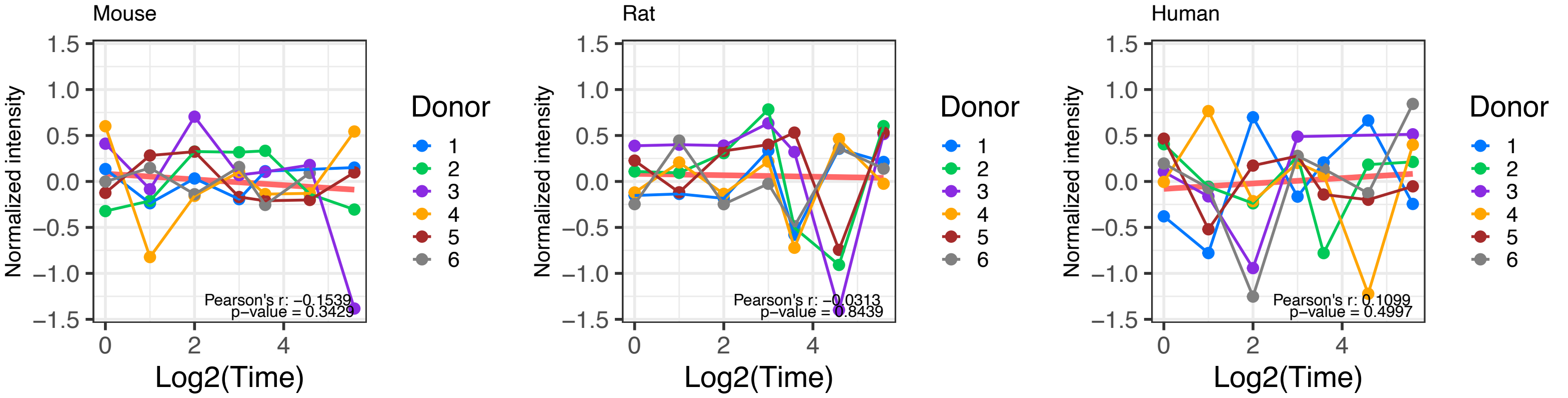

PE 42:9|PE 20:4_22:5

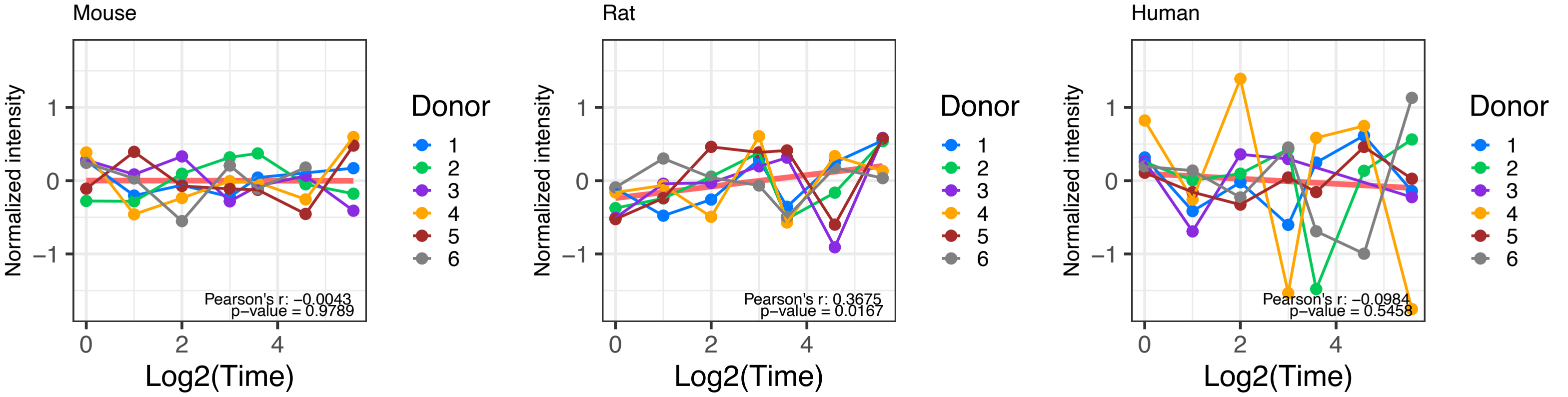

PE 44:10;O2|PE 22:6_22:4;O2

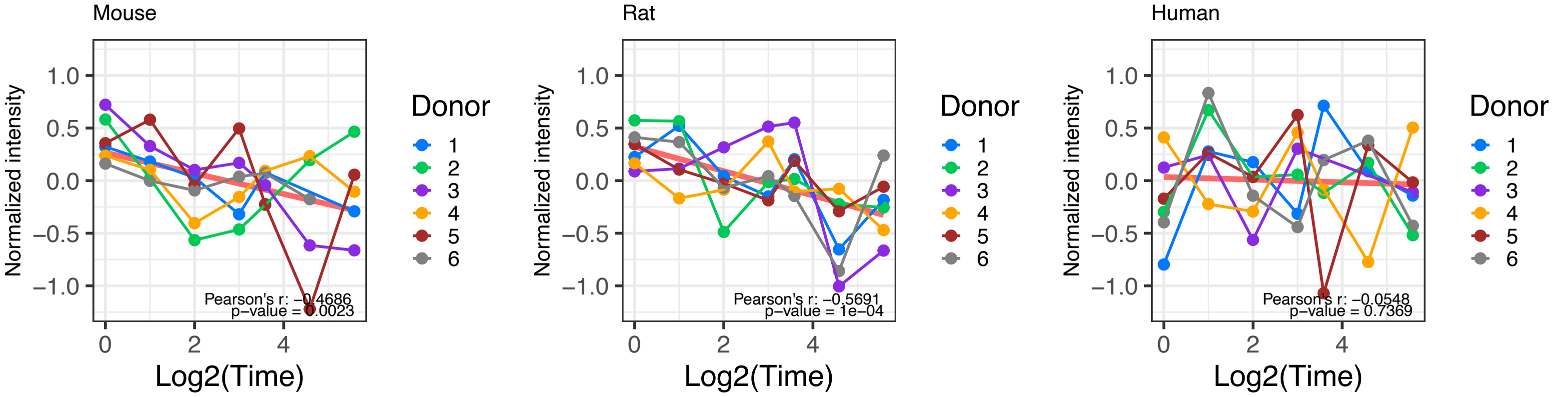

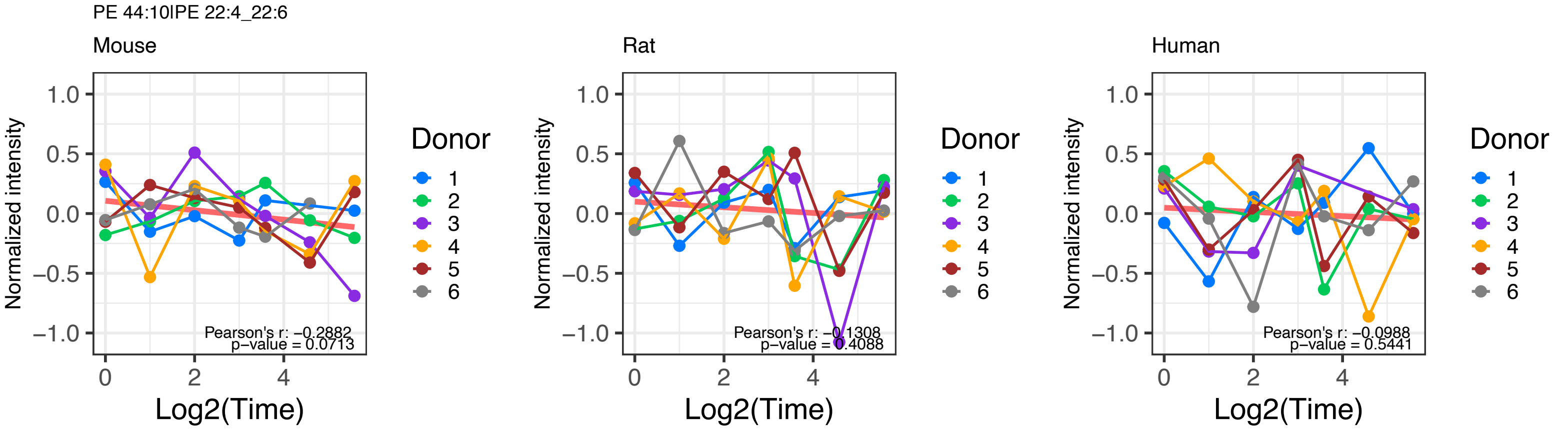
PE 44:10|PE 22:4_22:6
Mouse
Rat
Human
Normalized intensity
Log2(Time)
Donor
1
2
3
4
5
6
Pearson's r: −0.2882
p−value = 0.0713
Pearson's r: −0.1308
p−value = 0.4088
Pearson's r: −0.0988
p−value = 0.5441

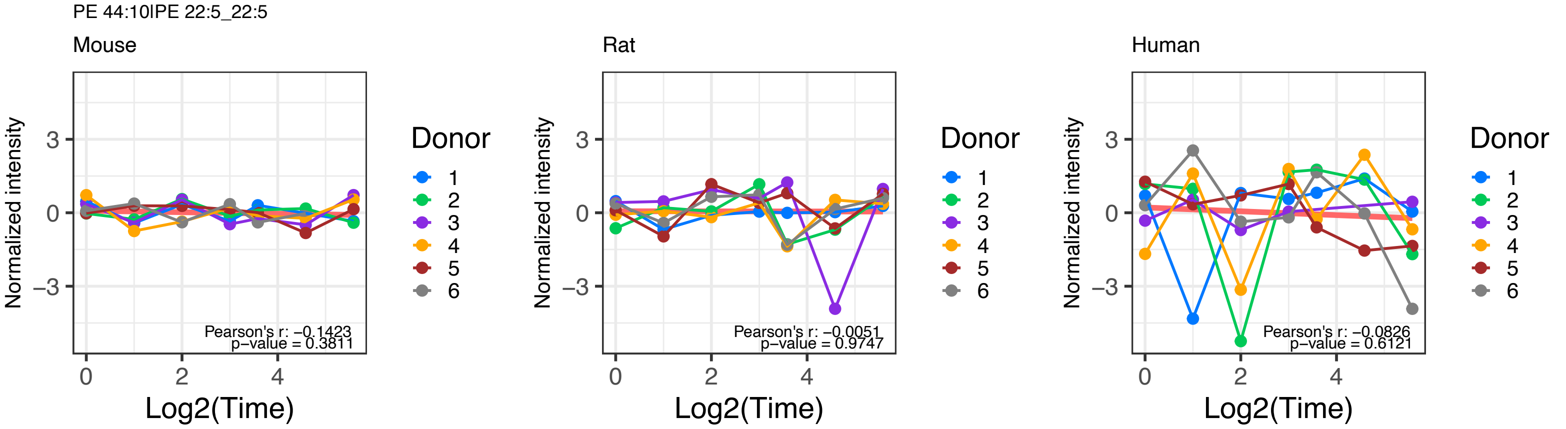
PE 44:10|PE 22:5_22:5
Mouse
Rat
Human
Normalized intensity
Log2(Time)
Donor
1
2
3
4
5
6
Pearson's r: −0.1423
p−value = 0.3811
Pearson's r: −0.0051
p−value = 0.9747
Pearson's r: −0.0826
p−value = 0.6121

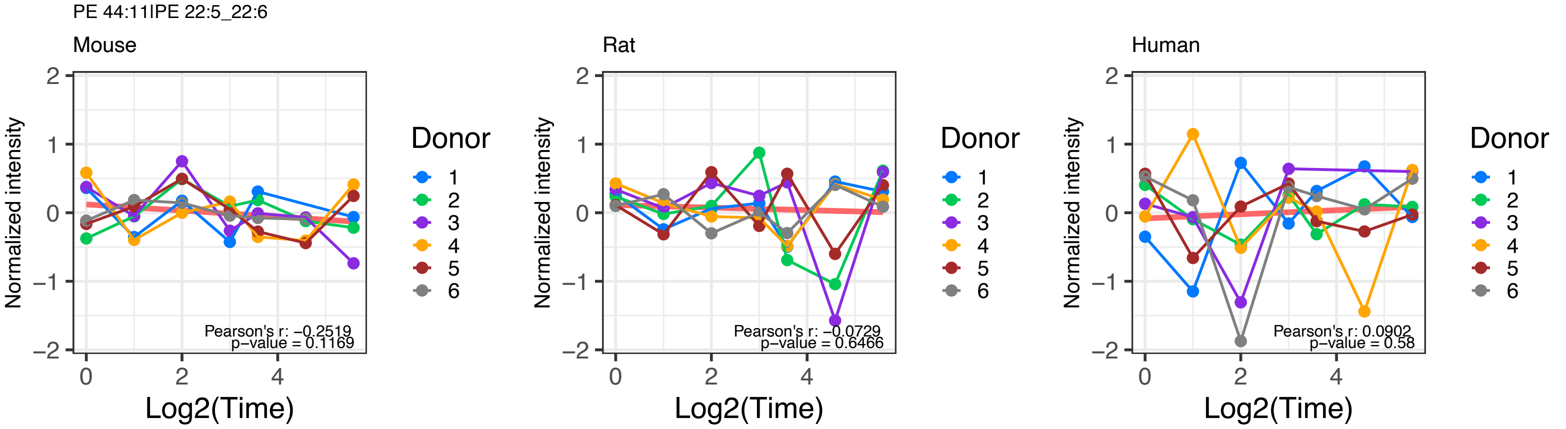
PE 44:11|PE 22:5_22:6
Mouse
Rat
Human
Normalized intensity
Log2(Time)
Donor
1
2
3
4
5
6
Pearson's r: −0.2519
p−value = 0.1169
Pearson's r: −0.0729
p−value = 0.6466
Pearson's r: 0.0902
p−value = 0.58

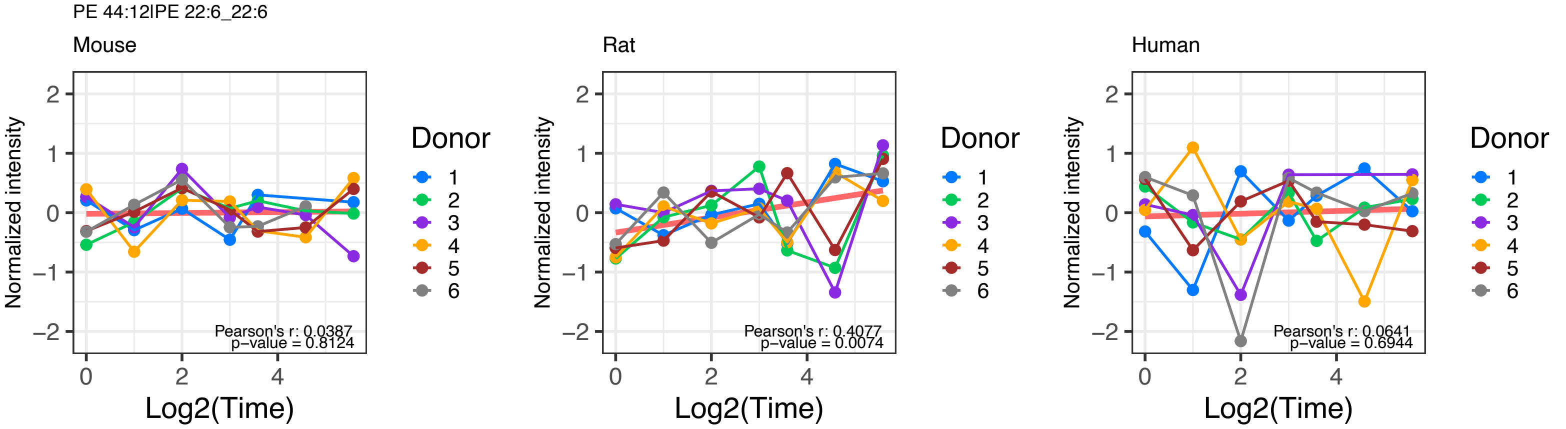
PE 44:12|PE 22:6_22:6
Mouse
Rat
Human
Normalized intensity
Log2(Time)
Donor
1
2
3
4
5
6
Pearson's r: 0.0387
p−value = 0.8124
Pearson's r: 0.4077
p−value = 0.0074
Pearson's r: 0.0641
p−value = 0.6944

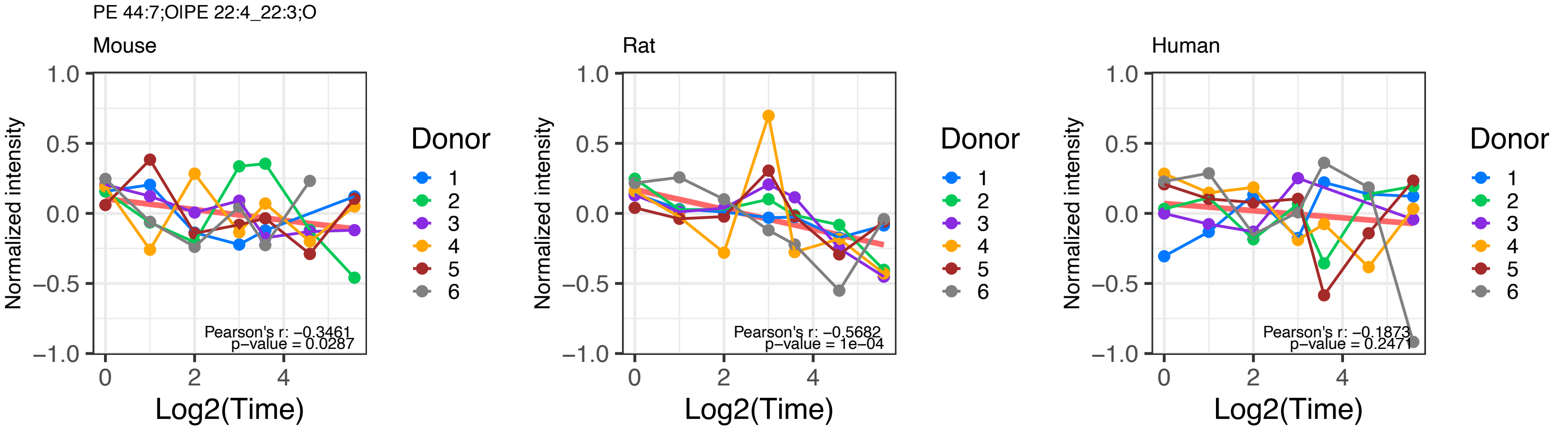
PE 44:7;O|PE 22:4_22:3;O
Mouse
Rat
Human
Normalized intensity
Log2(Time)
Donor
1
2
3
4
5
6
Pearson's r: −0.3461
p−value = 0.0287
Pearson's r: −0.5682
p−value = 1e−04
Pearson's r: −0.1873
p−value = 0.2471

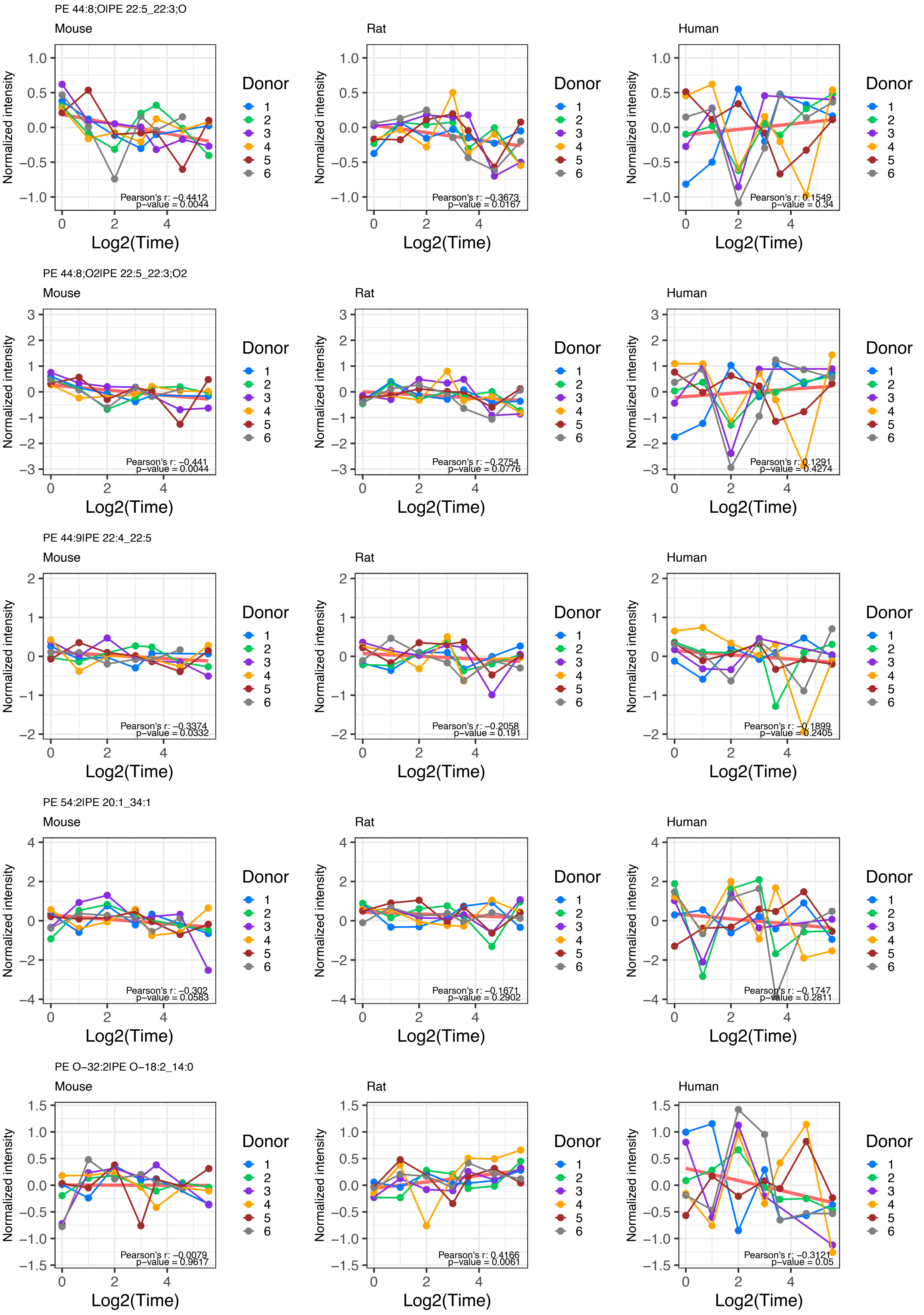
PE 44:8;O|PE 22:5_22:3;O
Mouse
Pearson's r: −0.4412
p−value = 0.0044
Rat
Pearson's r: −0.3673
p−value = 0.0167
Human
Pearson's r: 0.1549
p−value = 0.34
PE 44:8;O2|PE 22:5_22:3;O2
Mouse
Pearson's r: −0.441
p−value = 0.0044
Rat
Pearson's r: −0.2754
p−value = 0.0776
Human
Pearson's r: 0.1291
p−value = 0.4274
PE 44:9|PE 22:4_22:5
Mouse
Pearson's r: −0.3374
p−value = 0.0332
Rat
Pearson's r: −0.2058
p−value = 0.191
Human
Pearson's r: −0.1899
p−value = 0.2405
PE 54:2|PE 20:1_34:1
Mouse
Pearson's r: −0.302
p−value = 0.0583
Rat
Pearson's r: −0.1671
p−value = 0.2902
Human
Pearson's r: −0.1747
p−value = 0.2811
PE O−32:2|PE O−18:2_14:0
Mouse
Pearson's r: −0.0079
p−value = 0.9617
Rat
Pearson's r: 0.4166
p−value = 0.0061
Human
Pearson's r: −0.3121
p−value = 0.05
Normalized intensity
Log2(Time)
Donor
1
2
3
4
5
6

PE O−33:2|PE O−16:1_17:1

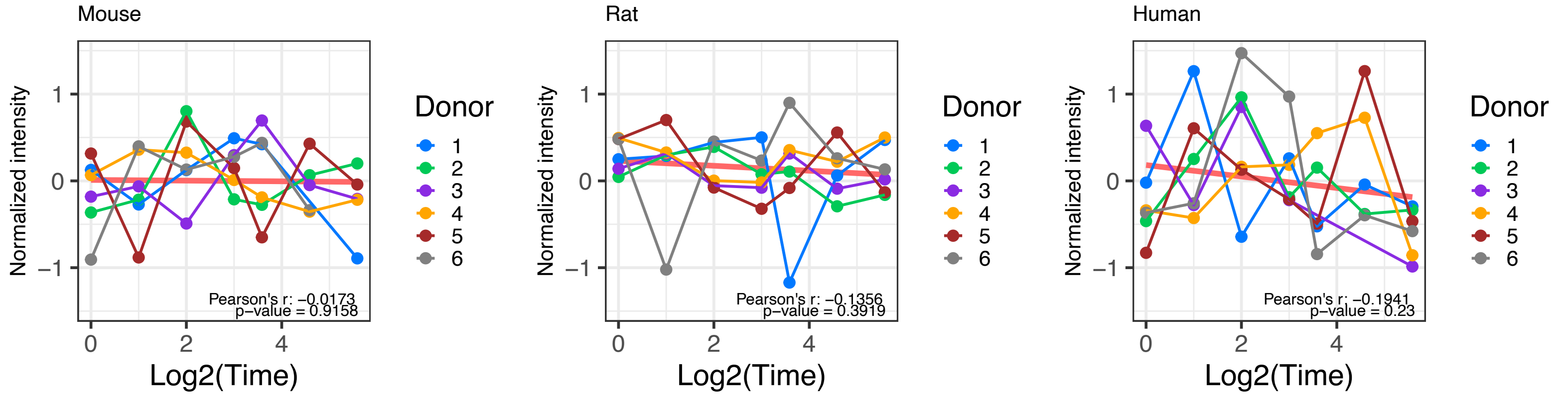

PE O−34:1|PE O−18:1_16:0

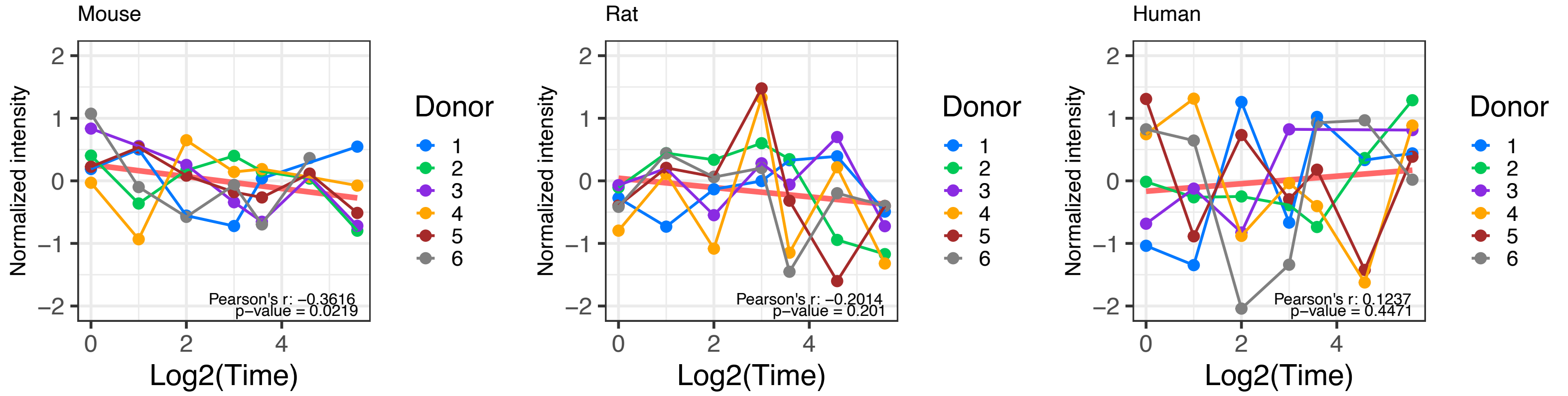

PE O−34:2|PE O−16:1_18:1

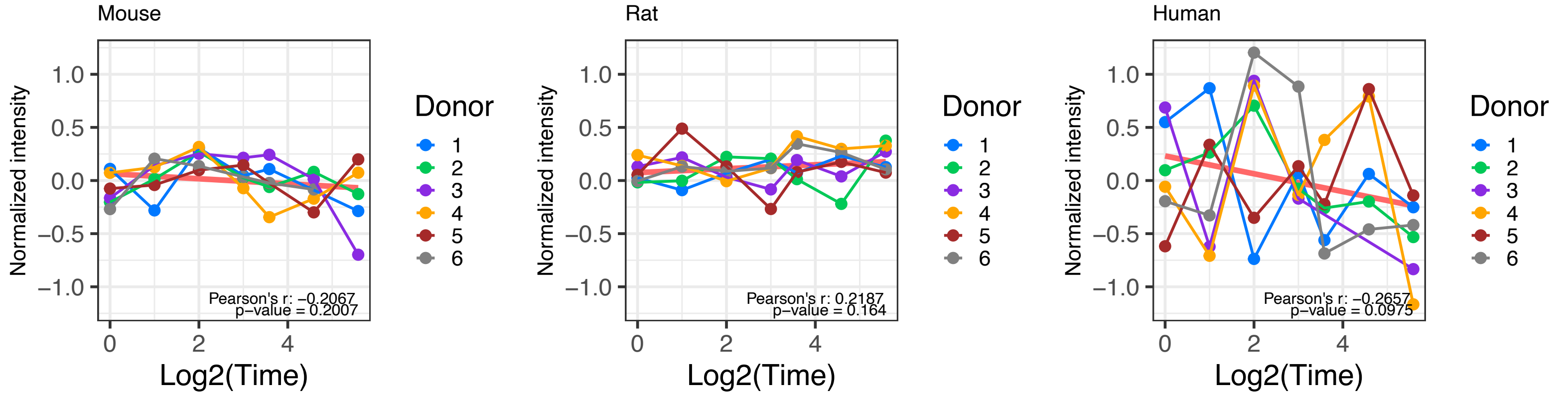

PE O−34:3|PE O−16:1_18:2

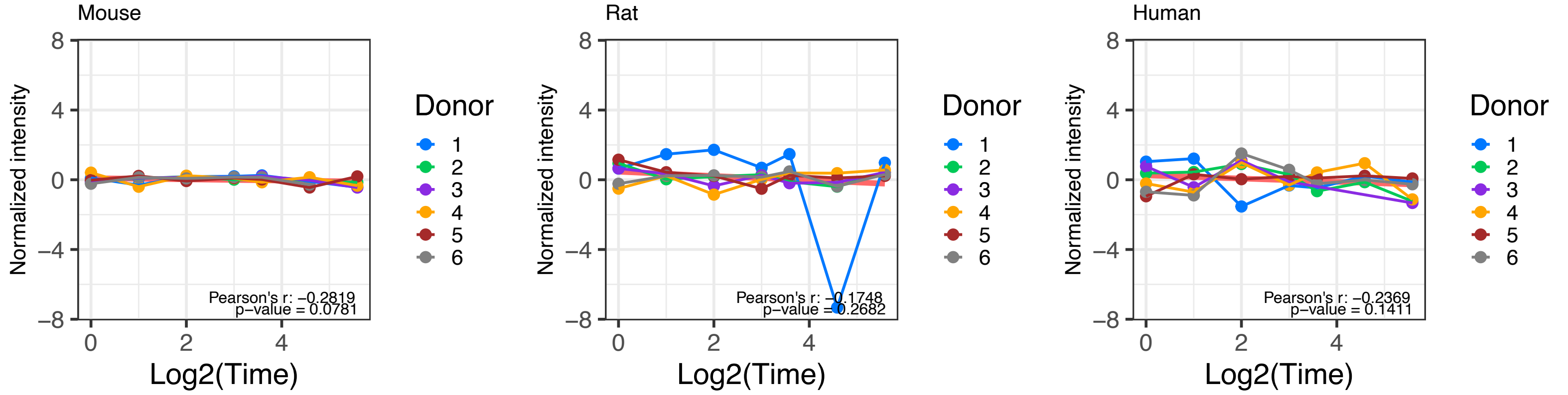

PE O−35:2|PE O−17:1_18:1

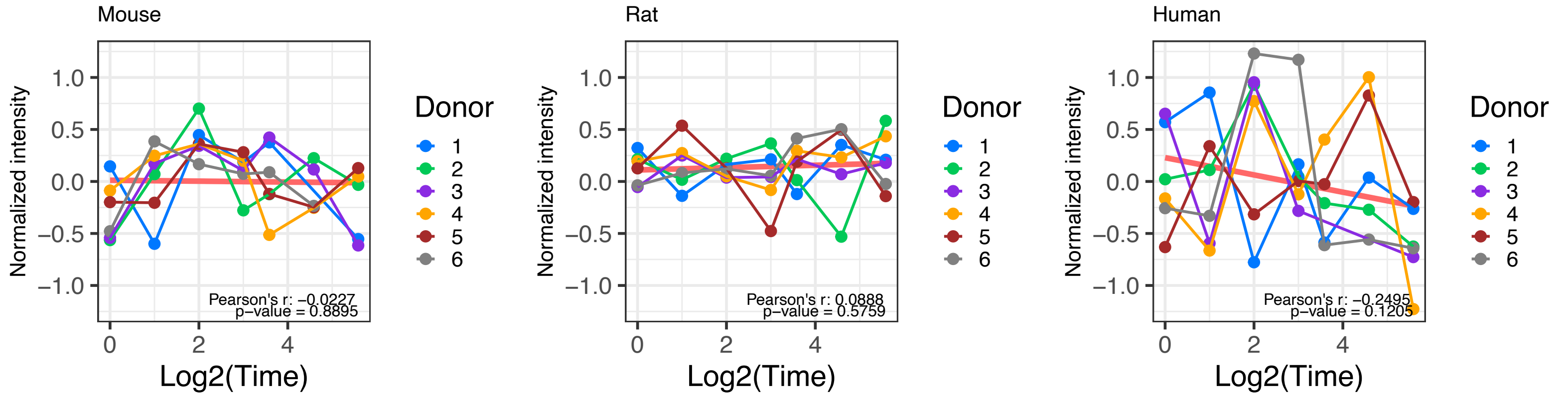

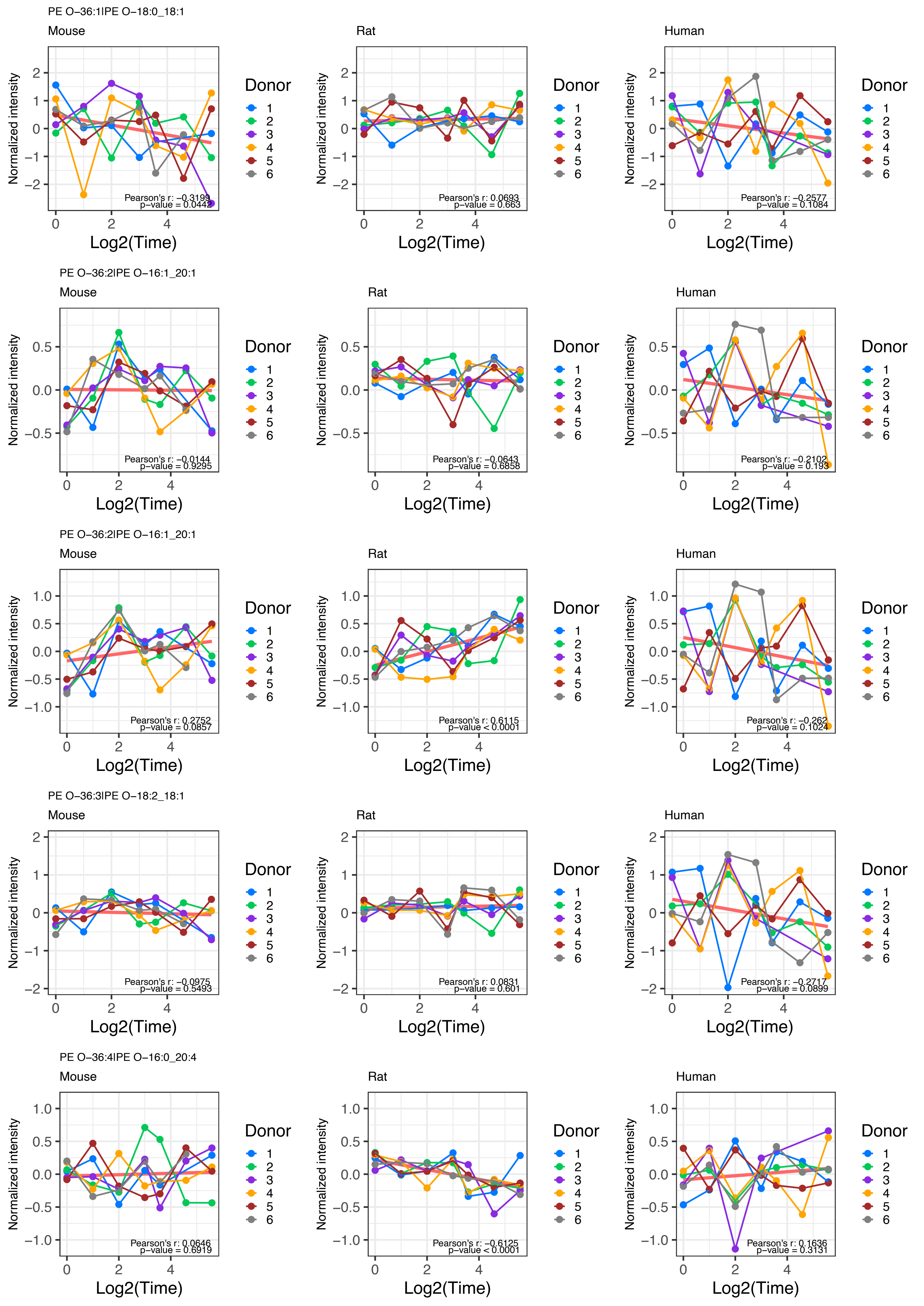

PE O-36:1|PE O-18:0_18:1
Mouse
Pearson's r: −0.3199
p−value = 0.0442
Rat
Pearson's r: 0.0693
p−value = 0.663
Human
Pearson's r: −0.2577
p−value = 0.1084
PE O-36:2|PE O-16:1_20:1
Mouse
Pearson's r: −0.0144
p−value = 0.9295
Rat
Pearson's r: −0.0643
p−value = 0.6858
Human
Pearson's r: −0.2102
p−value = 0.193
PE O-36:2|PE O-16:1_20:1
Mouse
Pearson's r: 0.2752
p−value = 0.0857
Rat
Pearson's r: 0.6115
p−value < 0.0001
Human
Pearson's r: −0.262
p−value = 0.1024
PE O-36:3|PE O-18:2_18:1
Mouse
Pearson's r: −0.0975
p−value = 0.5493
Rat
Pearson's r: 0.0831
p−value = 0.601
Human
Pearson's r: −0.2717
p−value = 0.0899
PE O-36:4|PE O-16:0_20:4
Mouse
Pearson's r: 0.0646
p−value = 0.6919
Rat
Pearson's r: −0.6125
p−value < 0.0001
Human
Pearson's r: 0.1636
p−value = 0.3131
Normalized intensity
Log2(Time)
Donor
1
2
3
4
5
6

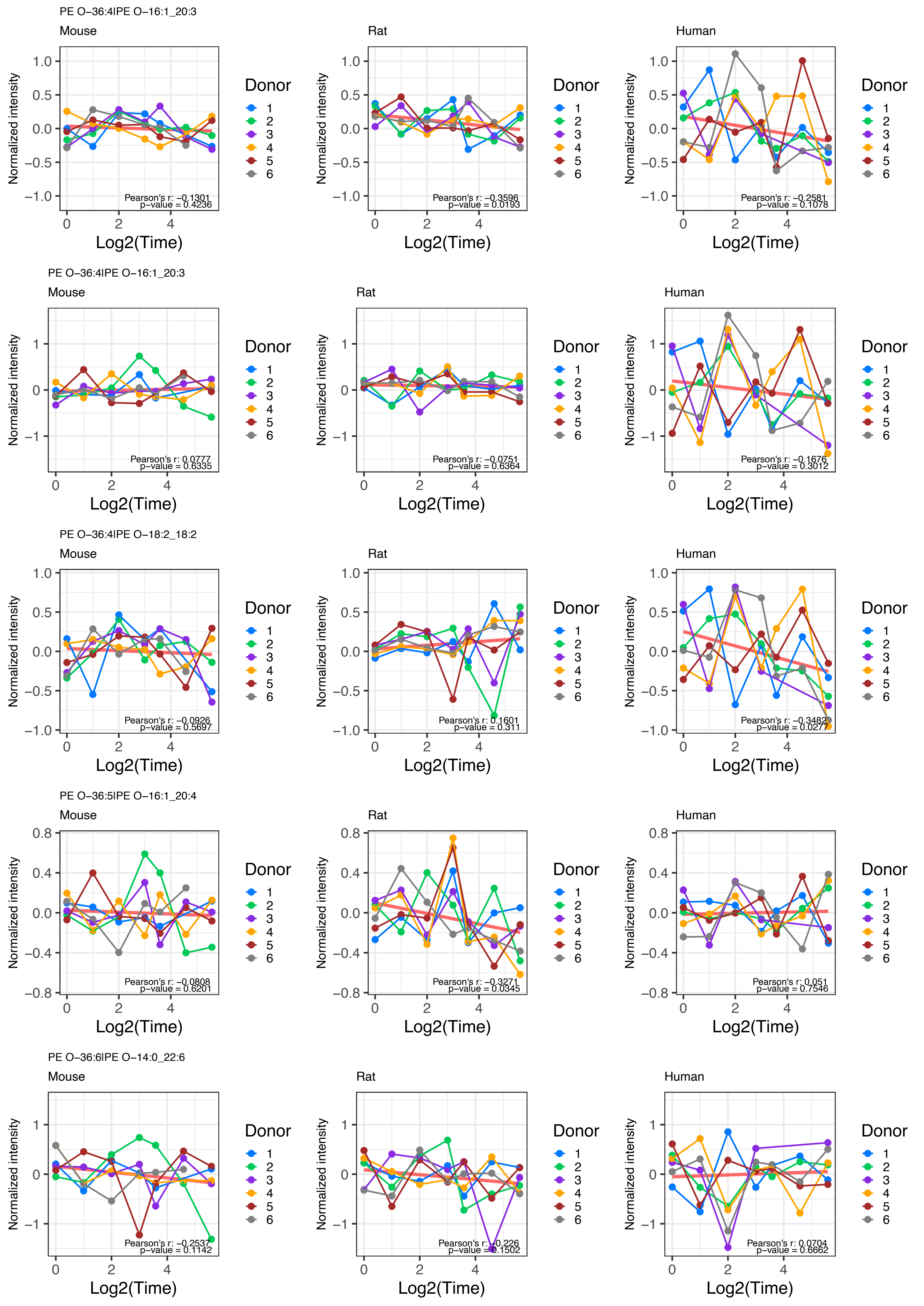

PE O−36:4|PE O−16:1_20:3
Mouse
Pearson's r: −0.1301
p−value = 0.4236
Rat
Pearson's r: −0.3596
p−value = 0.0193
Human
Pearson's r: −0.2581
p−value = 0.1078
PE O−36:4|PE O−16:1_20:3
Mouse
Pearson's r: 0.0777
p−value = 0.6335
Rat
Pearson's r: −0.0751
p−value = 0.6364
Human
Pearson's r: −0.1676
p−value = 0.3012
PE O−36:4|PE O−18:2_18:2
Mouse
Pearson's r: −0.0926
p−value = 0.5697
Rat
Pearson's r: 0.1601
p−value = 0.311
Human
Pearson's r: −0.3482
p−value = 0.0277
PE O−36:5|PE O−16:1_20:4
Mouse
Pearson's r: −0.0808
p−value = 0.6201
Rat
Pearson's r: −0.3271
p−value = 0.0345
Human
Pearson's r: 0.051
p−value = 0.7546
PE O−36:6|PE O−14:0_22:6
Mouse
Pearson's r: −0.2537
p−value = 0.1142
Rat
Pearson's r: −0.226
p−value = 0.1502
Human
Pearson's r: 0.0704
p−value = 0.6662
Normalized intensity
Log2(Time)
Donor
1
2
3
4
5
6

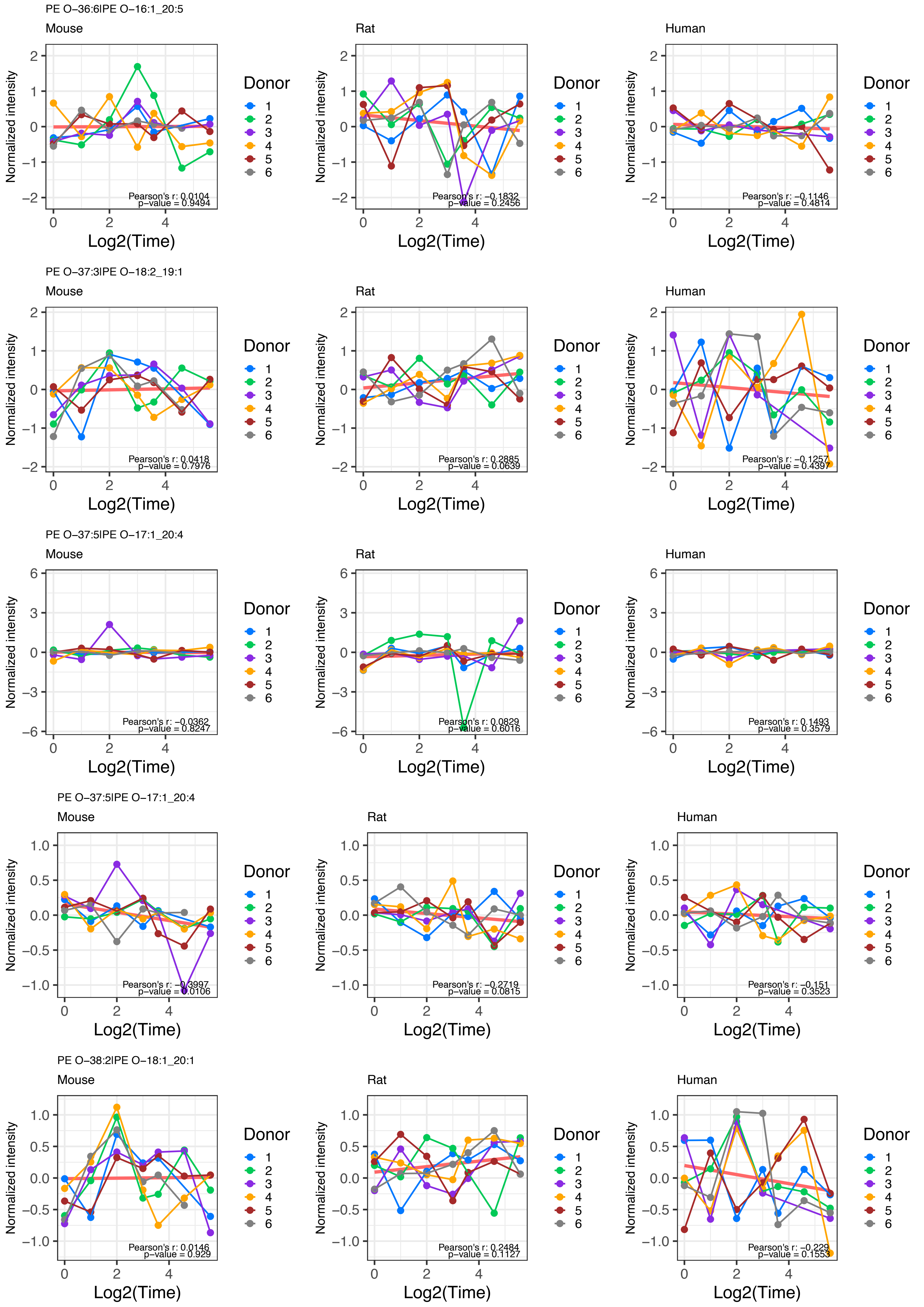
PE O−36:6|PE O−16:1_20:5
Mouse
Pearson's r: 0.0104
p−value = 0.9494
Rat
Pearson's r: −0.1832
p−value = 0.2456
Human
Pearson's r: −0.1146
p−value = 0.4814
PE O−37:3|PE O−18:2_19:1
Mouse
Pearson's r: 0.0418
p−value = 0.7976
Rat
Pearson's r: 0.2885
p−value = 0.0639
Human
Pearson's r: −0.1257
p−value = 0.4397
PE O−37:5|PE O−17:1_20:4
Mouse
Pearson's r: −0.0362
p−value = 0.8247
Rat
Pearson's r: 0.0829
p−value = 0.6016
Human
Pearson's r: 0.1493
p−value = 0.3579
PE O−37:5|PE O−17:1_20:4
Mouse
Pearson's r: −0.3997
p−value = 0.0106
Rat
Pearson's r: −0.2719
p−value = 0.0815
Human
Pearson's r: −0.151
p−value = 0.3523
PE O−38:2|PE O−18:1_20:1
Mouse
Pearson's r: 0.0146
p−value = 0.929
Rat
Pearson's r: 0.2484
p−value = 0.1127
Human
Pearson's r: −0.229
p−value = 0.1553
Normalized intensity
Log2(Time)
Donor
1
2
3
4
5
6

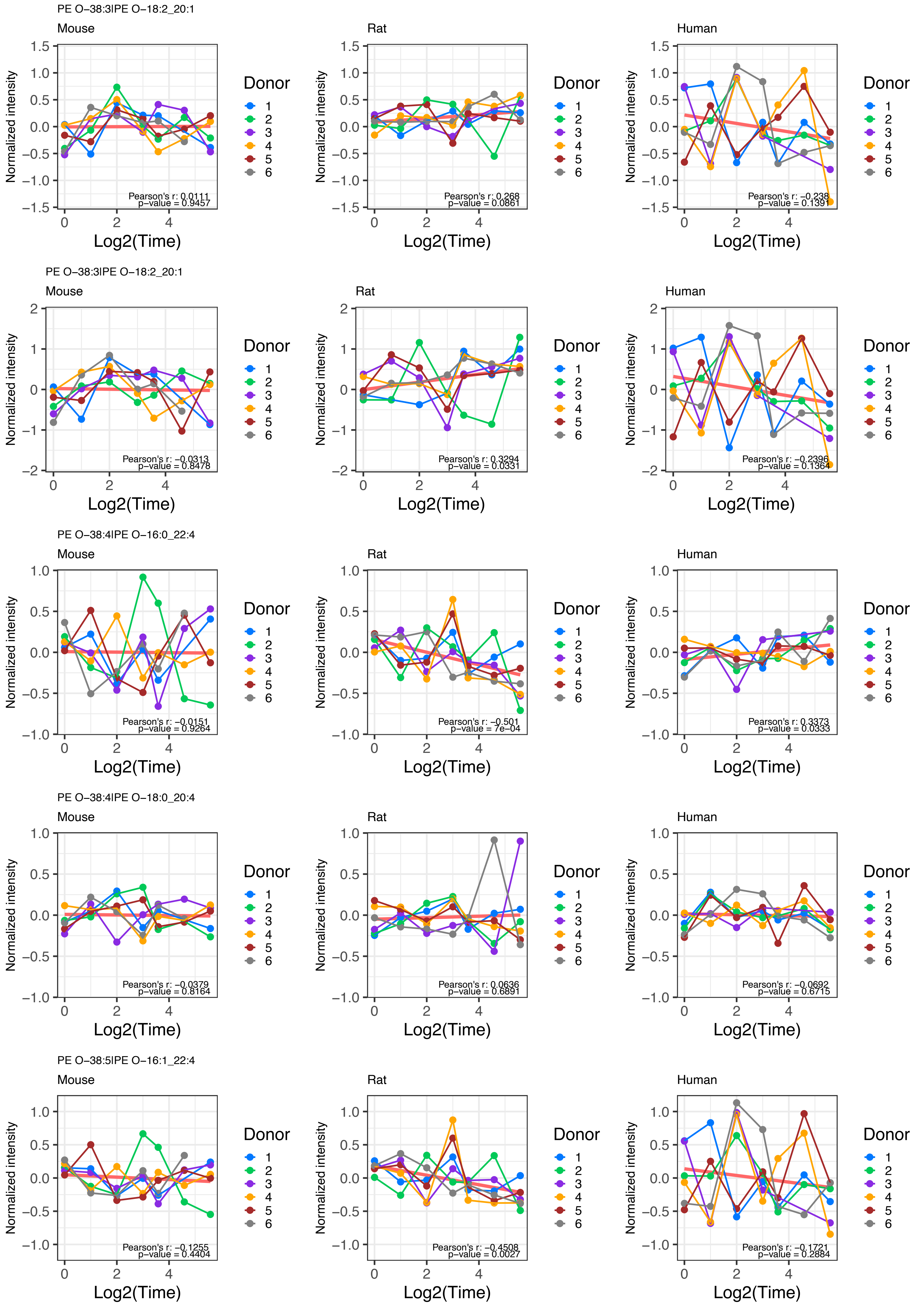
PE O−38:3|PE O−18:2_20:1
Mouse
Rat
Human
Normalized intensity
Log2(Time)
Donor
1
2
3
4
5
6
Pearson's r: 0.0111
p−value = 0.9457
Pearson's r: 0.268
p−value = 0.0861
Pearson's r: −0.238
p−value = 0.1391
PE O−38:3|PE O−18:2_20:1
Mouse
Rat
Human
Pearson's r: −0.0313
p−value = 0.8478
Pearson's r: 0.3294
p−value = 0.0331
Pearson's r: −0.2396
p−value = 0.1364
PE O−38:4|PE O−16:0_22:4
Mouse
Rat
Human
Pearson's r: −0.0151
p−value = 0.9264
Pearson's r: −0.501
p−value = 7e−04
Pearson's r: 0.3373
p−value = 0.0333
PE O−38:4|PE O−18:0_20:4
Mouse
Rat
Human
Pearson's r: −0.0379
p−value = 0.8164
Pearson's r: 0.0636
p−value = 0.6891
Pearson's r: −0.0692
p−value = 0.6715
PE O−38:5|PE O−16:1_22:4
Mouse
Rat
Human
Pearson's r: −0.1255
p−value = 0.4404
Pearson's r: −0.4508
p−value = 0.0027
Pearson's r: −0.1721
p−value = 0.2884

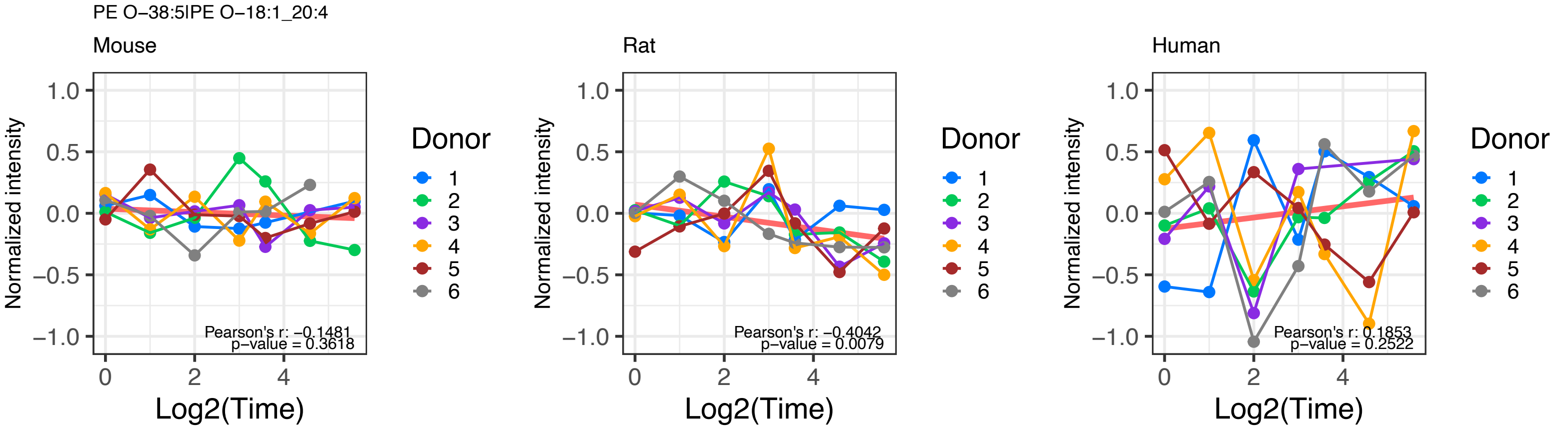
PE O−38:5|PE O−18:1_20:4
Mouse
Rat
Human
Normalized intensity
Log2(Time)
Donor
1
2
3
4
5
6
Pearson's r: −0.1481
p−value = 0.3618
Pearson's r: −0.4042
p−value = 0.0079
Pearson's r: 0.1853
p−value = 0.2522

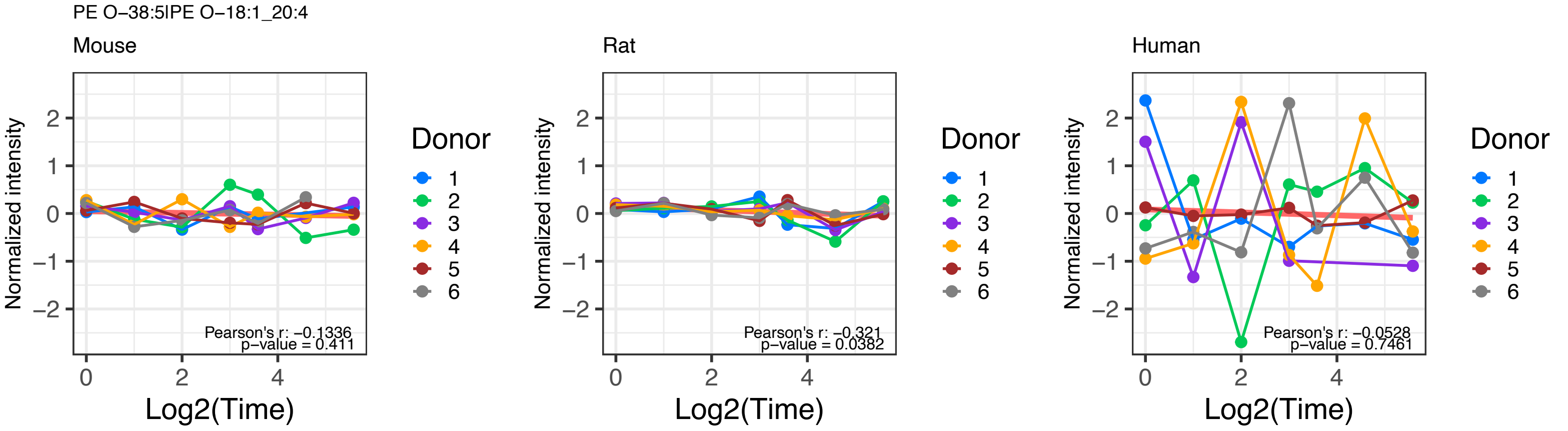
PE O−38:5|PE O−18:1_20:4
Mouse
Rat
Human
Normalized intensity
Log2(Time)
Donor
1
2
3
4
5
6
Pearson's r: −0.1336
p−value = 0.411
Pearson's r: −0.321
p−value = 0.0382
Pearson's r: −0.0528
p−value = 0.7461

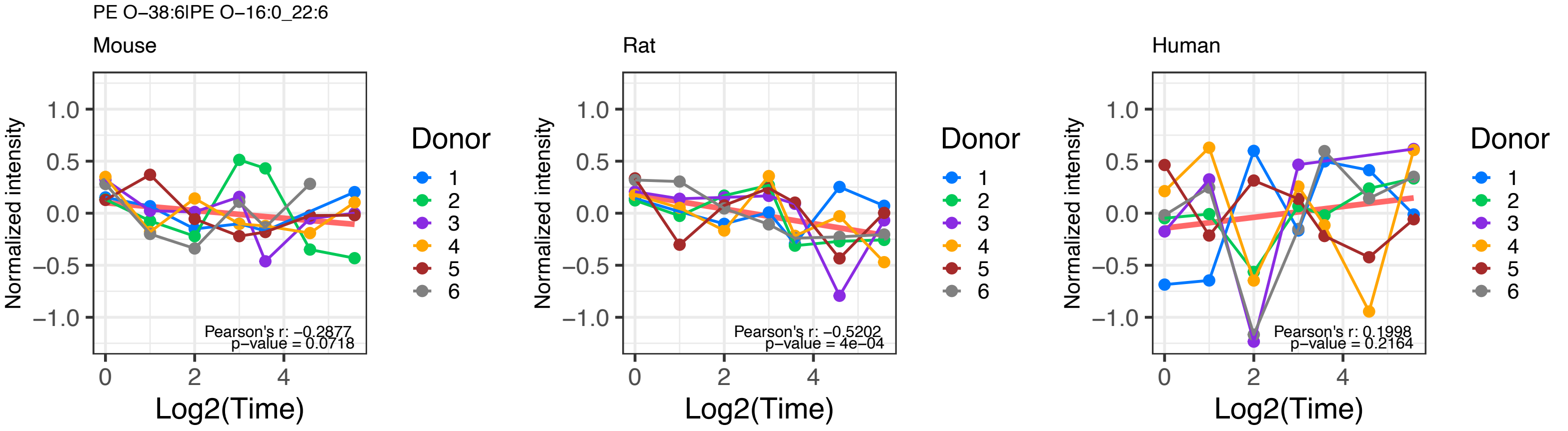
PE O−38:6|PE O−16:0_22:6
Mouse
Rat
Human
Normalized intensity
Log2(Time)
Donor
1
2
3
4
5
6
Pearson's r: −0.2877
p−value = 0.0718
Pearson's r: −0.5202
p−value = 4e−04
Pearson's r: 0.1998
p−value = 0.2164

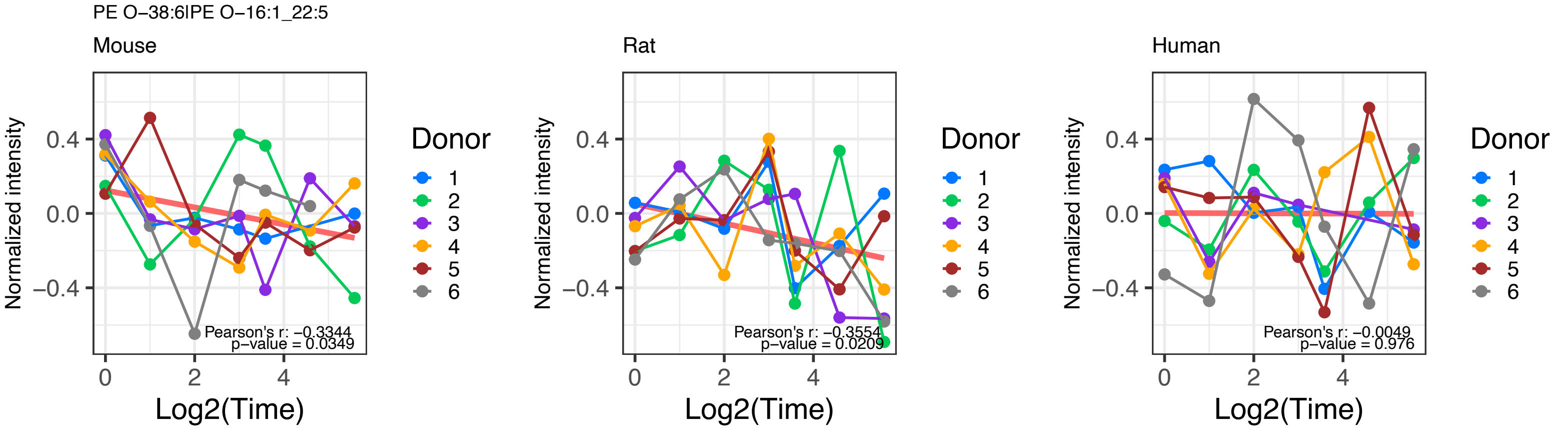
PE O−38:6|PE O−16:1_22:5
Mouse
Rat
Human
Normalized intensity
Log2(Time)
Donor
1
2
3
4
5
6
Pearson's r: −0.3344
p−value = 0.0349
Pearson's r: −0.3554
p−value = 0.0209
Pearson's r: −0.0049
p−value = 0.976

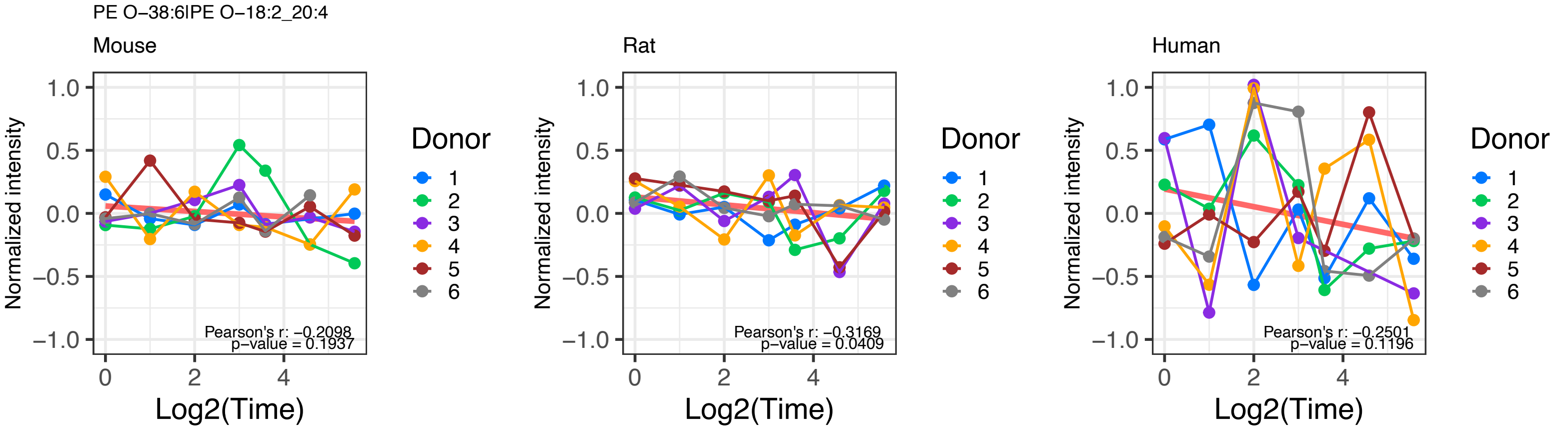
PE O−38:6|PE O−18:2_20:4
Mouse
Rat
Human
Normalized intensity
Log2(Time)
Donor
1
2
3
4
5
6
Pearson's r: −0.2098
p−value = 0.1937
Pearson's r: −0.3169
p−value = 0.0409
Pearson's r: −0.2501
p−value = 0.1196

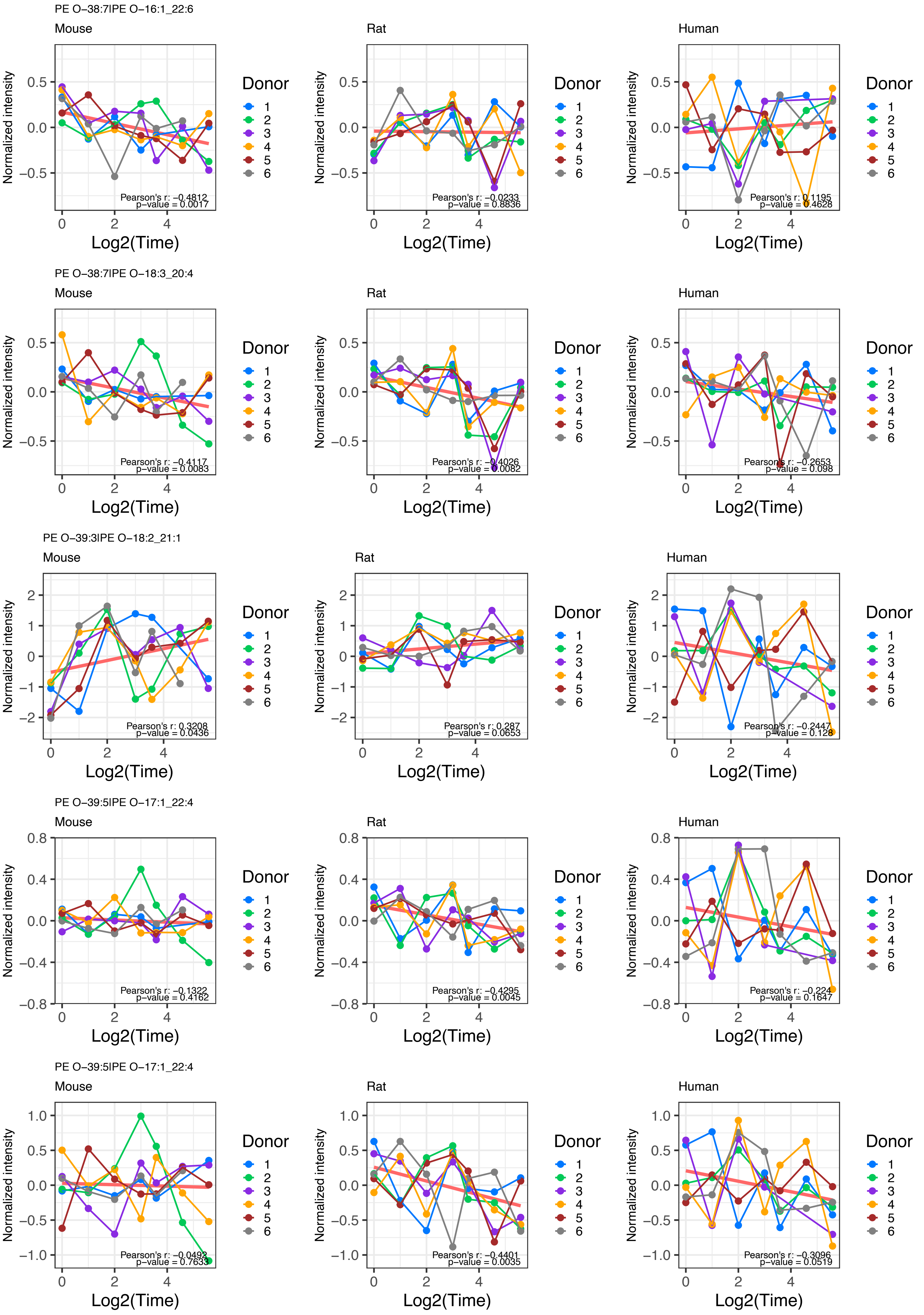
PE O−38:7|PE O−16:1_22:6
Mouse
Rat
Human
Pearson's r: −0.4812
p−value = 0.0017
Pearson's r: −0.0233
p−value = 0.8836
Pearson's r: 0.1195
p−value = 0.4628
PE O−38:7|PE O−18:3_20:4
Mouse
Rat
Human
Pearson's r: −0.4117
p−value = 0.0083
Pearson's r: −0.4026
p−value = 0.0082
Pearson's r: −0.2653
p−value = 0.098
PE O−39:3|PE O−18:2_21:1
Mouse
Rat
Human
Pearson's r: 0.3208
p−value = 0.0436
Pearson's r: 0.287
p−value = 0.0653
Pearson's r: −0.2447
p−value = 0.128
PE O−39:5|PE O−17:1_22:4
Mouse
Rat
Human
Pearson's r: −0.1322
p−value = 0.4162
Pearson's r: −0.4295
p−value = 0.0045
Pearson's r: −0.224
p−value = 0.1647
PE O−39:5|PE O−17:1_22:4
Mouse
Rat
Human
Pearson's r: −0.0492
p−value = 0.7633
Pearson's r: −0.4401
p−value = 0.0035
Pearson's r: −0.3096
p−value = 0.0519
Normalized intensity
Log2(Time)
Donor
1
2
3
4
5
6

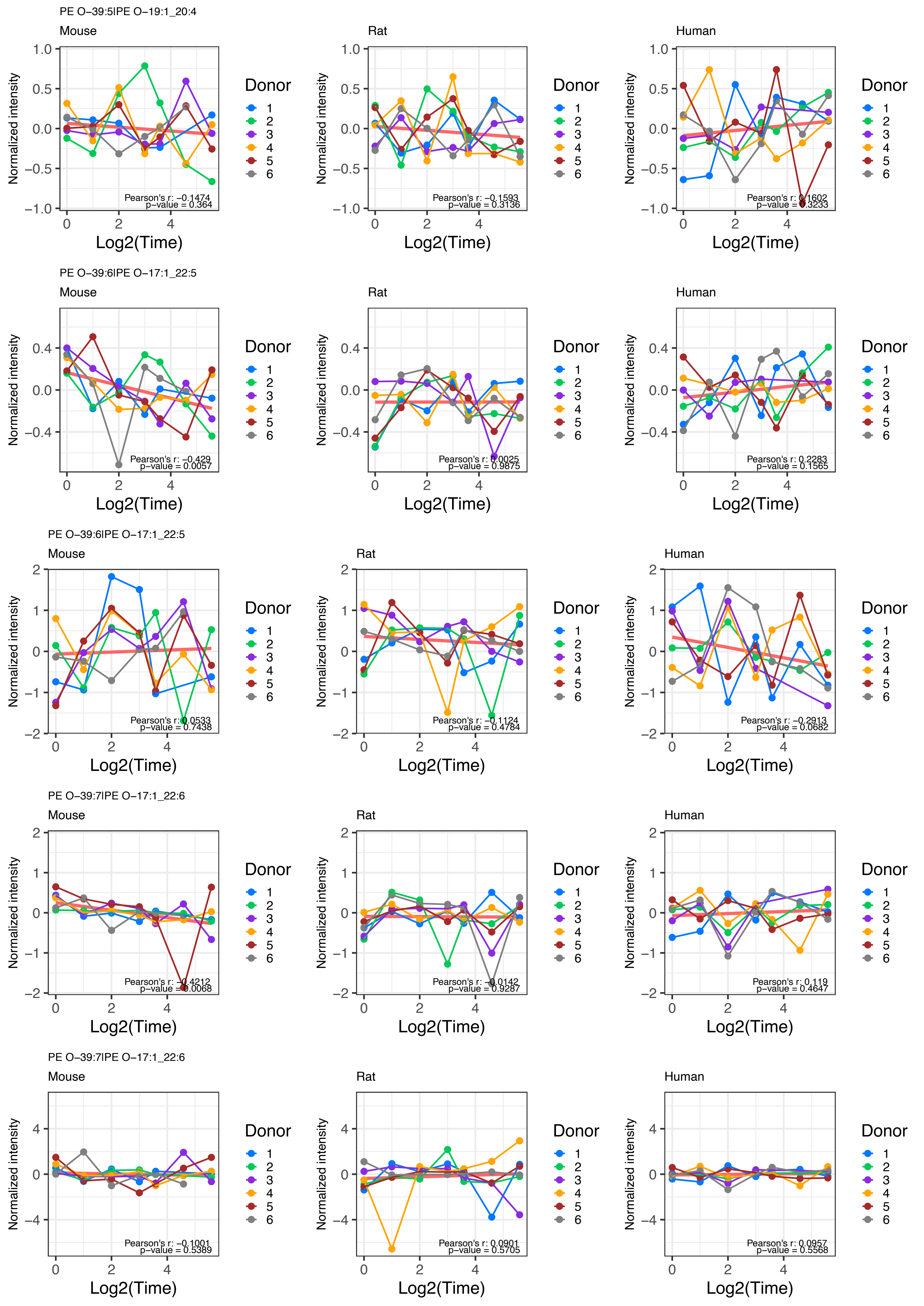
PE O−39:5|PE O−19:1_20:4
Mouse
Rat
Human
Normalized intensity
Log2(Time)
Donor
1
2
3
4
5
6
Pearson's r: −0.1474
p−value = 0.364
Pearson's r: −0.1593
p−value = 0.3136
Pearson's r: 0.1602
p−value = 0.3233
PE O−39:6|PE O−17:1_22:5
Pearson's r: −0.429
p−value = 0.0057
Pearson's r: 0.0025
p−value = 0.9875
Pearson's r: 0.2283
p−value = 0.1565
PE O−39:6|PE O−17:1_22:5
Pearson's r: 0.0533
p−value = 0.7438
Pearson's r: −0.1124
p−value = 0.4784
Pearson's r: −0.2913
p−value = 0.0682
PE O−39:7|PE O−17:1_22:6
Pearson's r: −0.4212
p−value = 0.0068
Pearson's r: −0.0142
p−value = 0.9287
Pearson's r: 0.119
p−value = 0.4647
PE O−39:7|PE O−17:1_22:6
Pearson's r: −0.1001
p−value = 0.5389
Pearson's r: 0.0901
p−value = 0.5705
Pearson's r: 0.0957
p−value = 0.5568

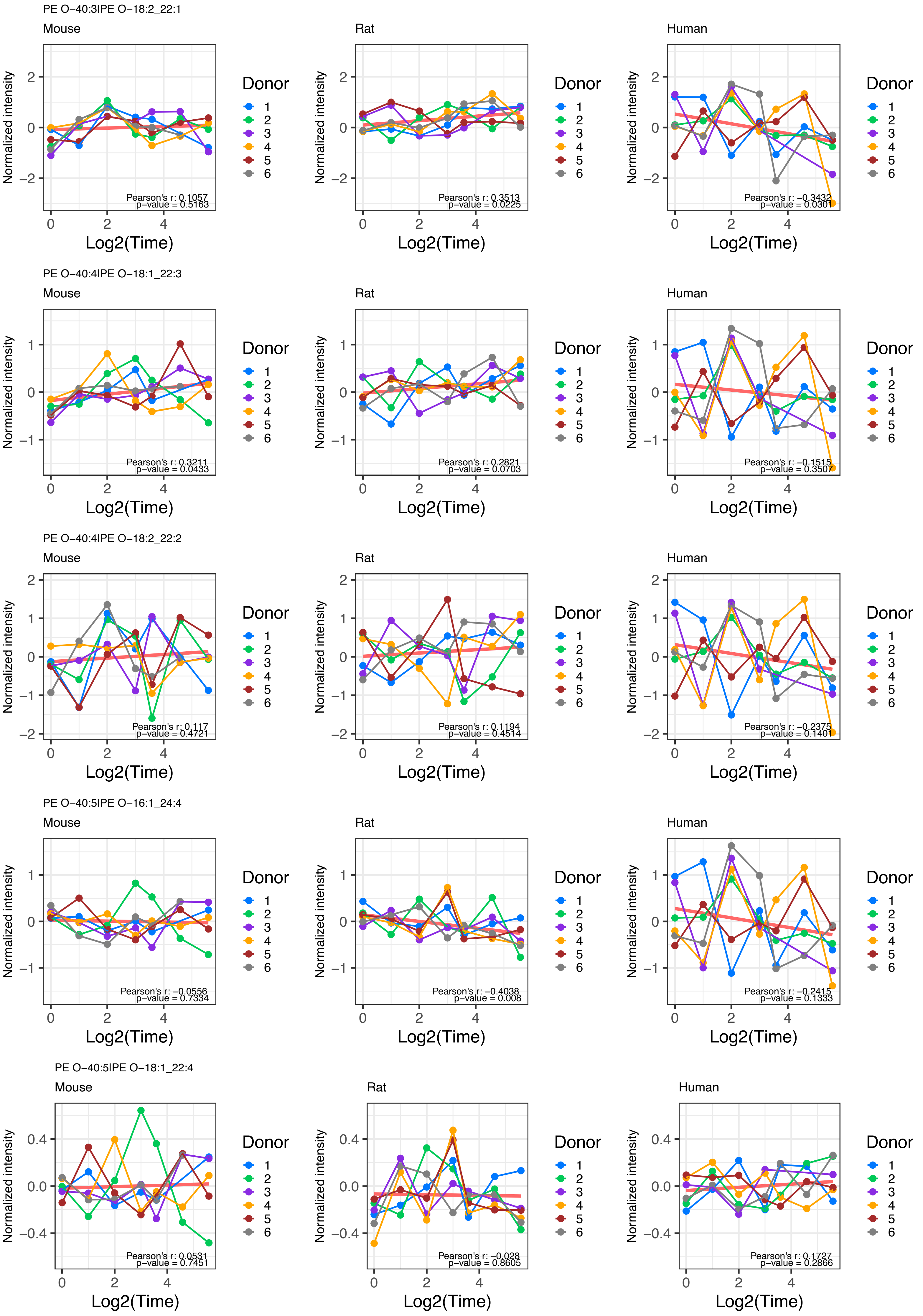
PE O−40:3|PE O−18:2_22:1
Mouse
Rat
Human
Normalized intensity
Log2(Time)
Donor
1
2
3
4
5
6
Pearson's r: 0.1057
p−value = 0.5163
Pearson's r: 0.3513
p−value = 0.0225
Pearson's r: −0.3432
p−value = 0.0301
PE O−40:4|PE O−18:1_22:3
Pearson's r: 0.3211
p−value = 0.0433
Pearson's r: 0.2821
p−value = 0.0703
Pearson's r: −0.1515
p−value = 0.3507
PE O−40:4|PE O−18:2_22:2
Pearson's r: 0.117
p−value = 0.4721
Pearson's r: 0.1194
p−value = 0.4514
Pearson's r: −0.2375
p−value = 0.1401
PE O−40:5|PE O−16:1_24:4
Pearson's r: −0.0556
p−value = 0.7334
Pearson's r: −0.4038
p−value = 0.008
Pearson's r: −0.2415
p−value = 0.1333
PE O−40:5|PE O−18:1_22:4
Pearson's r: 0.0531
p−value = 0.7451
Pearson's r: −0.028
p−value = 0.8605
Pearson's r: 0.1727
p−value = 0.2866

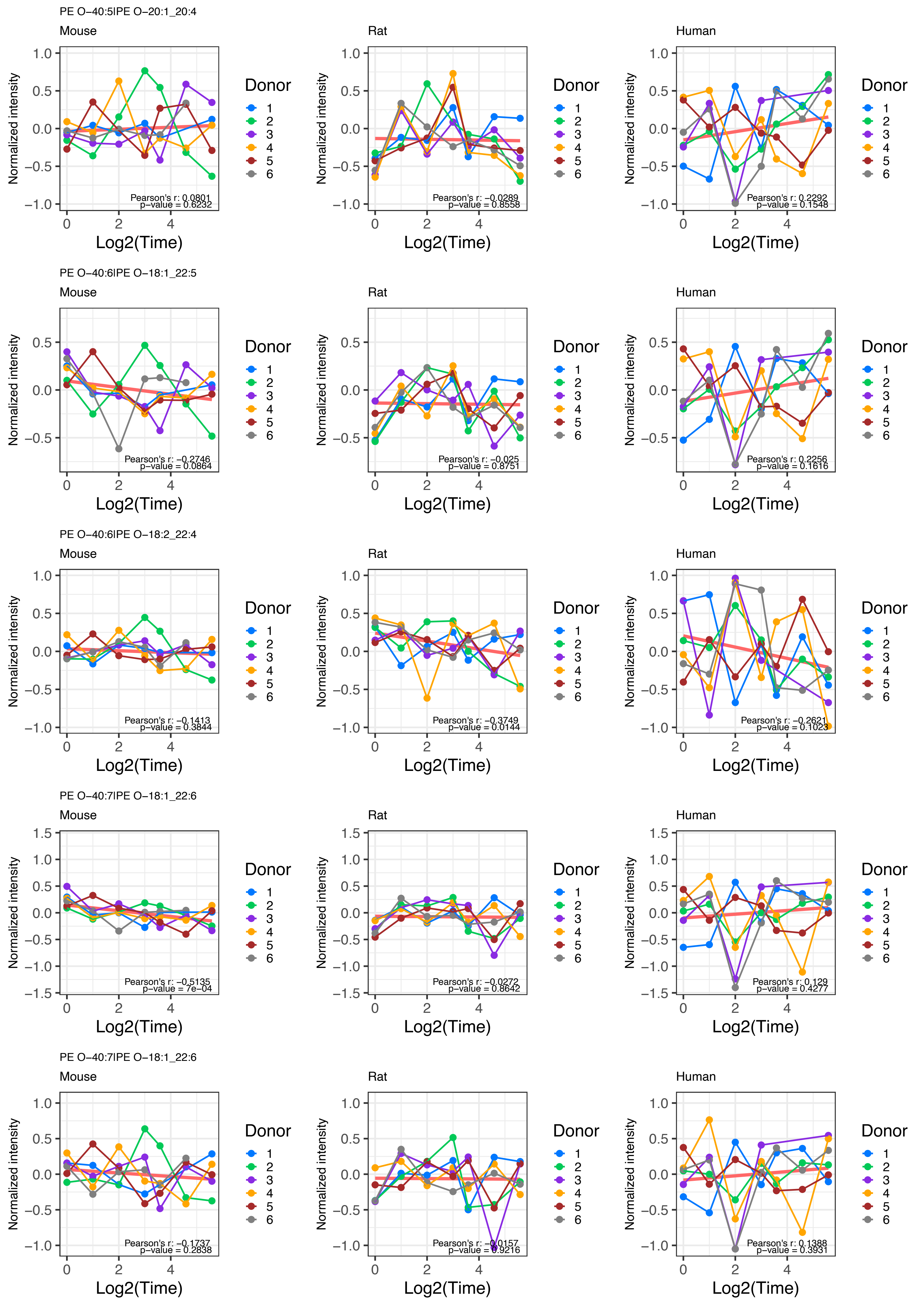
PE O−40:5|PE O−20:1_20:4
Mouse
Pearson's r: 0.0801
p−value = 0.6232
Rat
Pearson's r: −0.0289
p−value = 0.8558
Human
Pearson's r: 0.2292
p−value = 0.1548
PE O−40:6|PE O−18:1_22:5
Mouse
Pearson's r: −0.2746
p−value = 0.0864
Rat
Pearson's r: −0.025
p−value = 0.8751
Human
Pearson's r: 0.2256
p−value = 0.1616
PE O−40:6|PE O−18:2_22:4
Mouse
Pearson's r: −0.1413
p−value = 0.3844
Rat
Pearson's r: −0.3749
p−value = 0.0144
Human
Pearson's r: −0.2621
p−value = 0.1023
PE O−40:7|PE O−18:1_22:6
Mouse
Pearson's r: −0.5135
p−value = 7e−04
Rat
Pearson's r: −0.0272
p−value = 0.8642
Human
Pearson's r: 0.129
p−value = 0.4277
PE O−40:7|PE O−18:1_22:6
Mouse
Pearson's r: −0.1737
p−value = 0.2838
Rat
Pearson's r: −0.0157
p−value = 0.9216
Human
Pearson's r: 0.1388
p−value = 0.3931
Normalized intensity
Log2(Time)
Donor
1
2
3
4
5
6

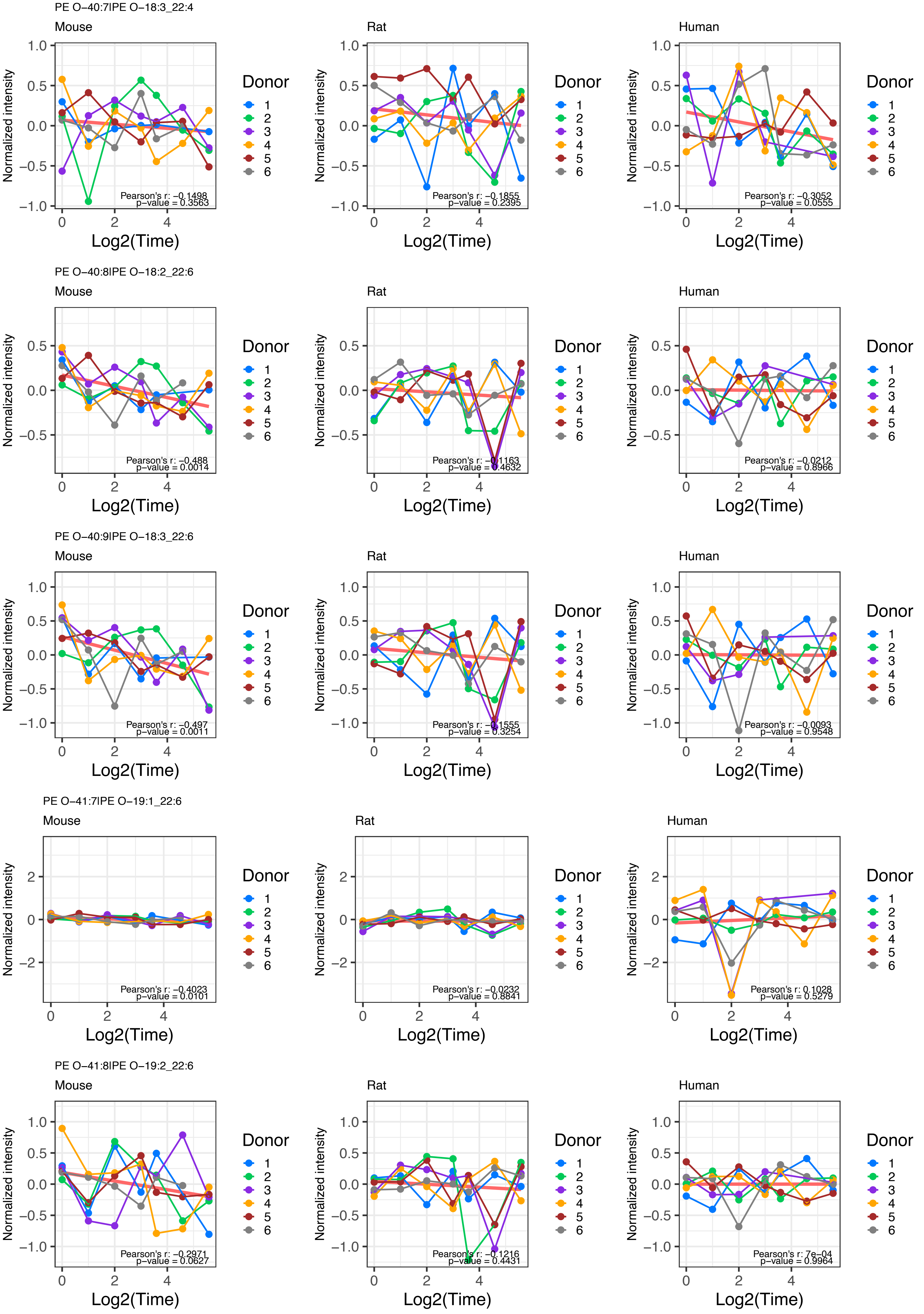

PE O−40:7|PE O−18:3_22:4
Mouse
Pearson's r: −0.1498
p−value = 0.3563
Rat
Pearson's r: −0.1855
p−value = 0.2395
Human
Pearson's r: −0.3052
p−value = 0.0555
PE O−40:8|PE O−18:2_22:6
Mouse
Pearson's r: −0.488
p−value = 0.0014
Rat
Pearson's r: −0.1163
p−value = 0.4632
Human
Pearson's r: −0.0212
p−value = 0.8966
PE O−40:9|PE O−18:3_22:6
Mouse
Pearson's r: −0.497
p−value = 0.0011
Rat
Pearson's r: −0.1555
p−value = 0.3254
Human
Pearson's r: −0.0093
p−value = 0.9548
PE O−41:7|PE O−19:1_22:6
Mouse
Pearson's r: −0.4023
p−value = 0.0101
Rat
Pearson's r: −0.0232
p−value = 0.8841
Human
Pearson's r: 0.1028
p−value = 0.5279
PE O−41:8|PE O−19:2_22:6
Mouse
Pearson's r: −0.2971
p−value = 0.0627
Rat
Pearson's r: −0.1216
p−value = 0.4431
Human
Pearson's r: 7e−04
p−value = 0.9964
Normalized intensity
Log2(Time)
Donor
1
2
3
4
5
6

PE O−42:5|PE O−18:1_24:4

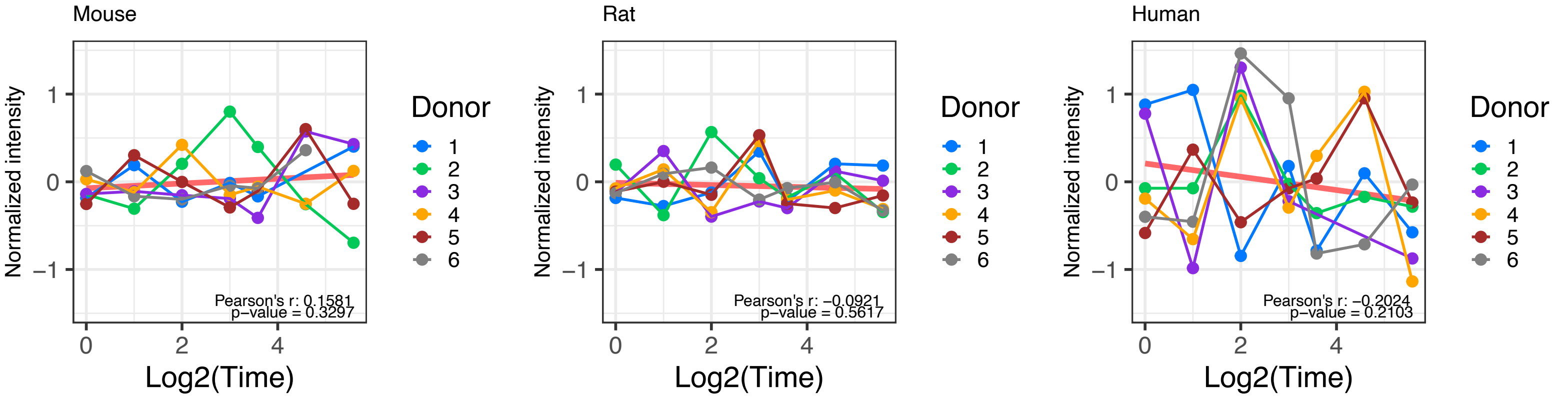

PE O−42:5|PE O−20:1_22:4

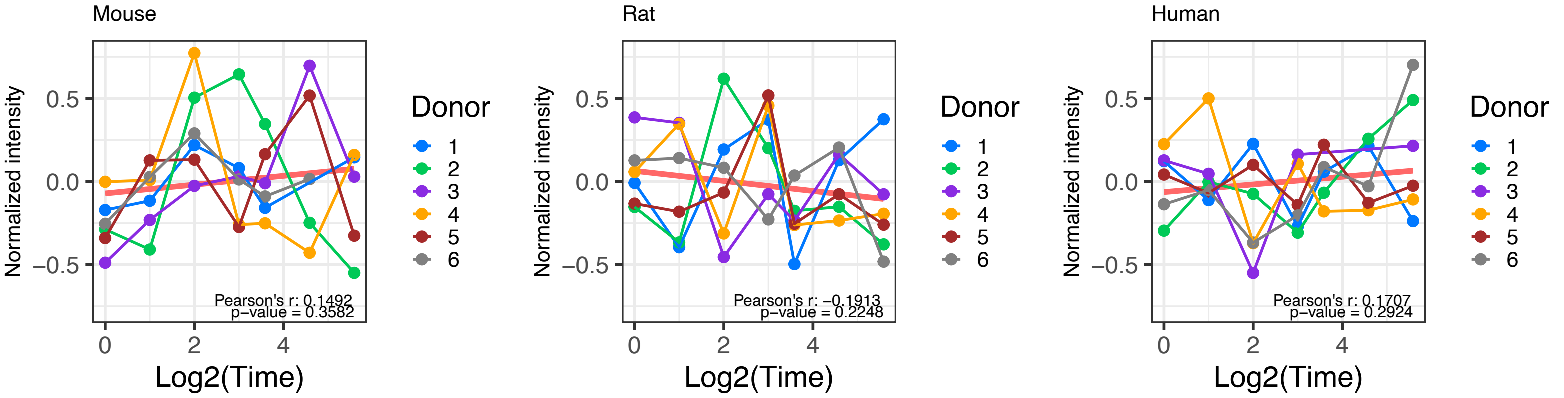

PE O−42:6|PE O−18:1_24:5

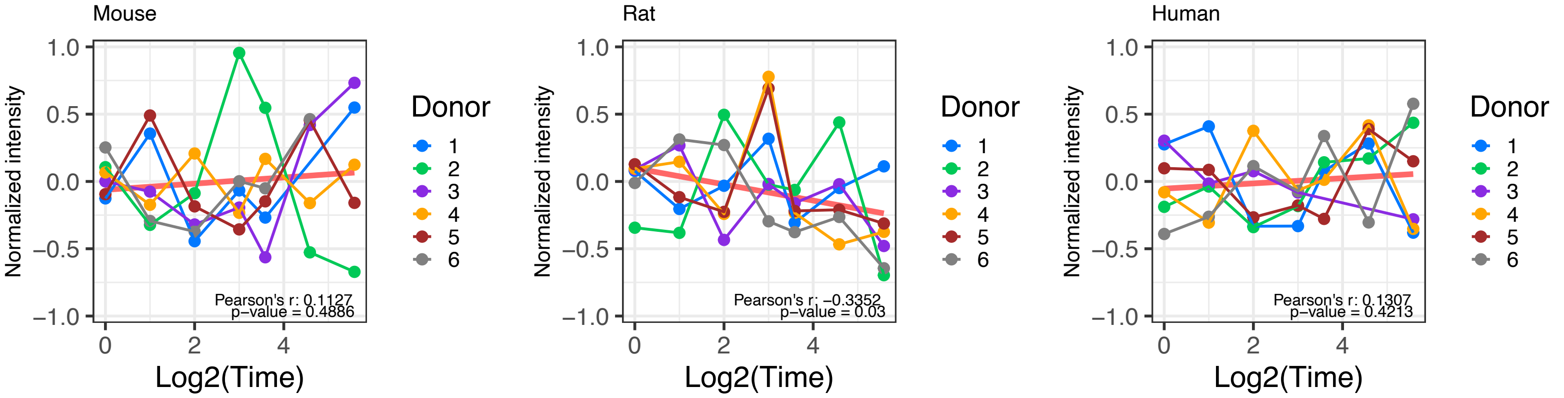

PE O−42:6|PE O−18:2_24:4

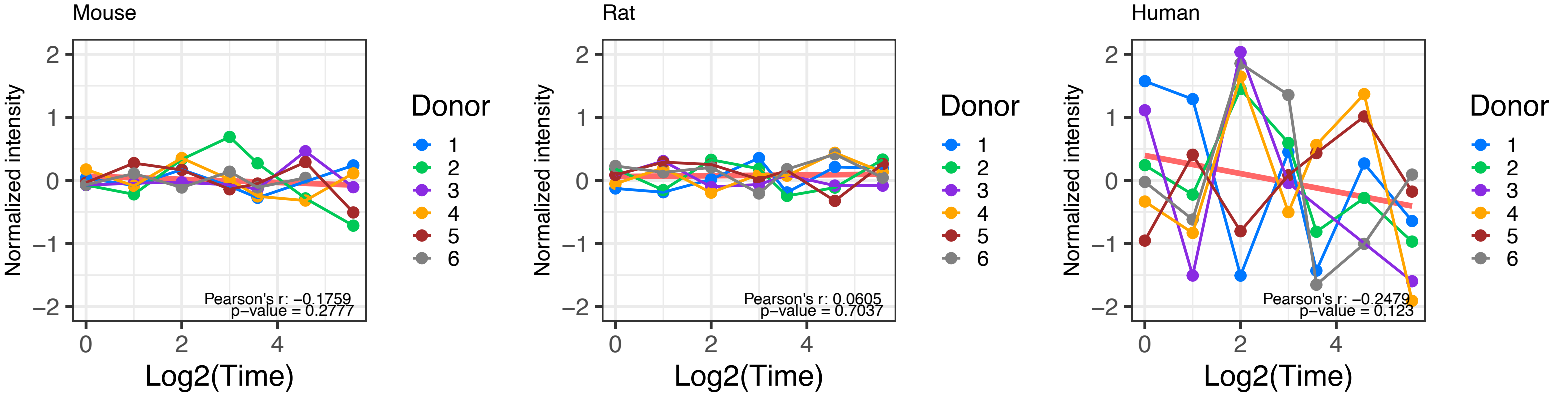

PE O−42:6|PE O−20:2_22:4

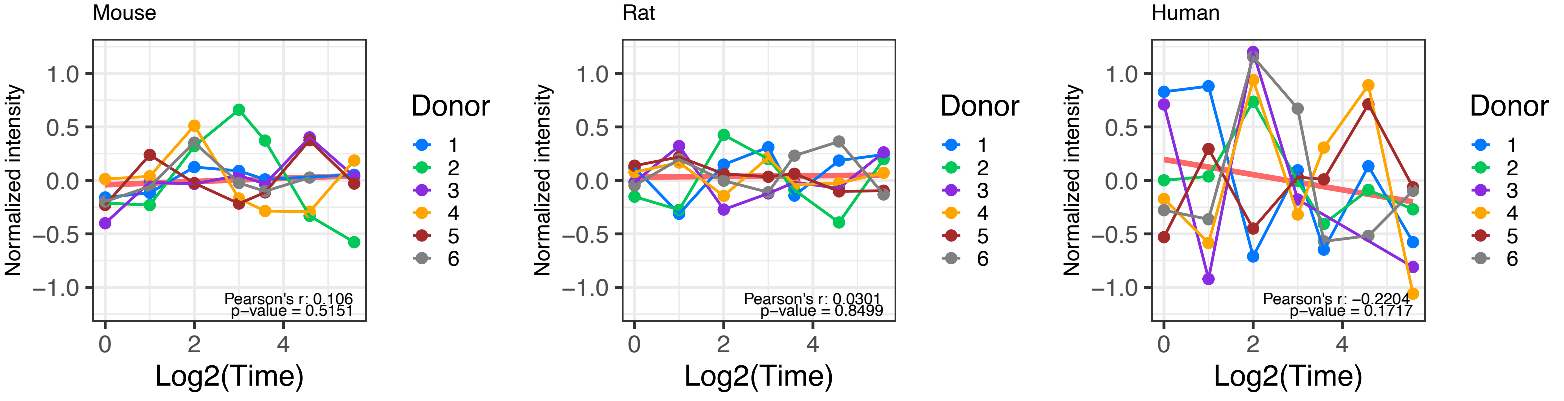

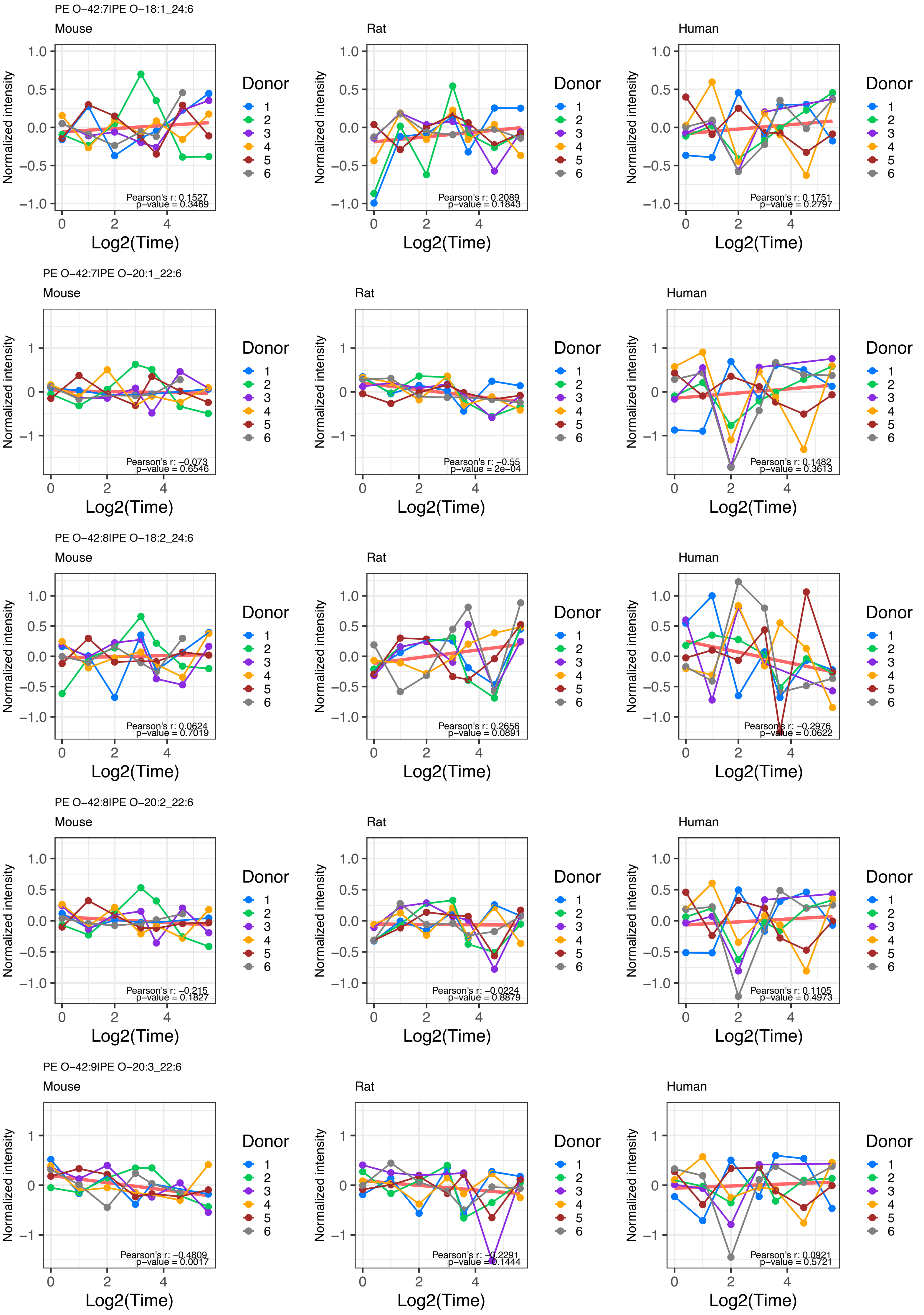
PE O−42:7lPE O−18:1_24:6
Mouse
Rat
Human
Normalized intensity
Log2(Time)
Donor
1
2
3
4
5
6
Pearson's r: 0.1527
p−value = 0.3469
Pearson's r: 0.2089
p−value = 0.1843
Pearson's r: 0.1751
p−value = 0.2797
PE O−42:7lPE O−20:1_22:6
Pearson's r: −0.073
p−value = 0.6546
Pearson's r: −0.55
p−value = 2e−04
Pearson's r: 0.1482
p−value = 0.3613
PE O−42:8lPE O−18:2_24:6
Pearson's r: 0.0624
p−value = 0.7019
Pearson's r: 0.2656
p−value = 0.0891
Pearson's r: −0.2976
p−value = 0.0622
PE O−42:8lPE O−20:2_22:6
Pearson's r: −0.215
p−value = 0.1827
Pearson's r: −0.0224
p−value = 0.8879
Pearson's r: 0.1105
p−value = 0.4973
PE O−42:9lPE O−20:3_22:6
Pearson's r: −0.4809
p−value = 0.0017
Pearson's r: −0.2291
p−value = 0.1444
Pearson's r: 0.0921
p−value = 0.5721

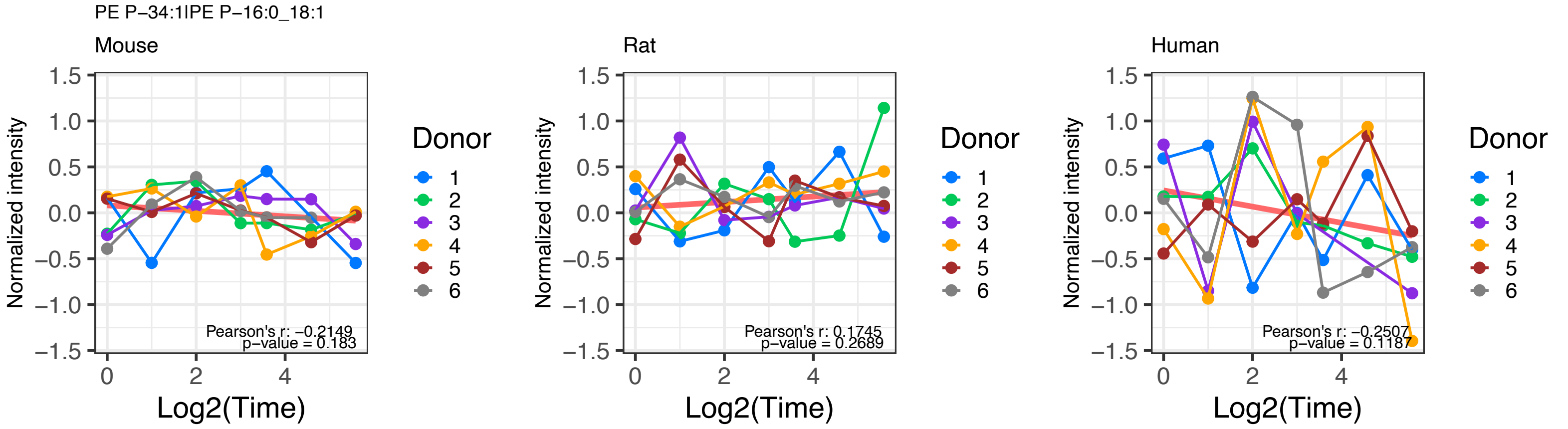
PE P−34:1|PE P−16:0_18:1
Mouse
Rat
Human
Normalized intensity
Log2(Time)
Donor
1
2
3
4
5
6
Pearson's r: −0.2149
p−value = 0.183
Pearson's r: 0.1745
p−value = 0.2689
Pearson's r: −0.2507
p−value = 0.1187

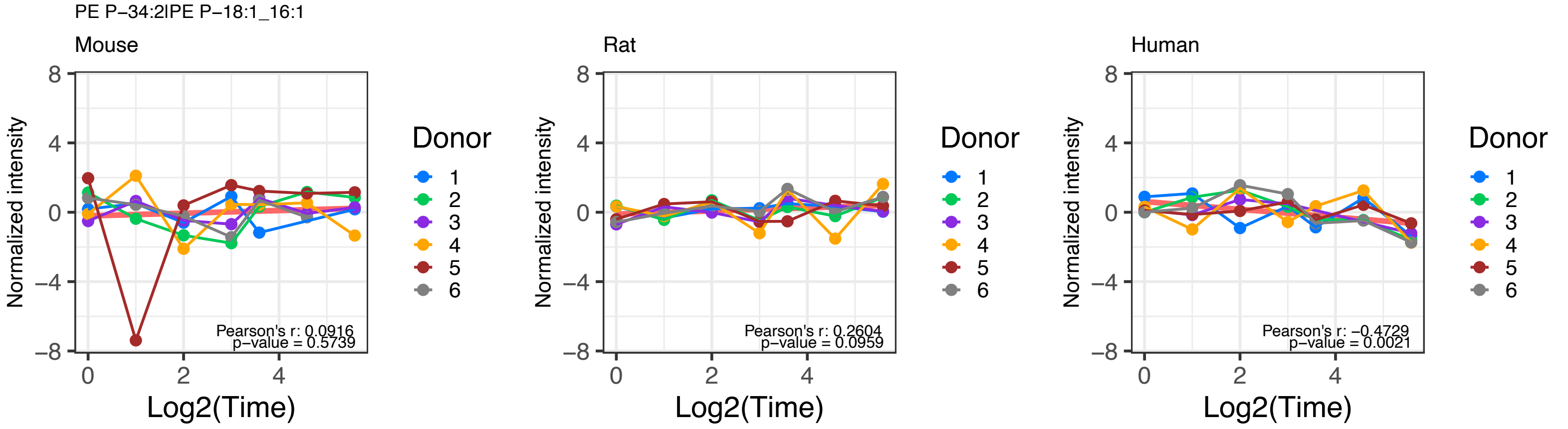
PE P−34:2|PE P−18:1_16:1
Mouse
Rat
Human
Normalized intensity
Log2(Time)
Donor
1
2
3
4
5
6
Pearson's r: 0.0916
p−value = 0.5739
Pearson's r: 0.2604
p−value = 0.0959
Pearson's r: −0.4729
p−value = 0.0021

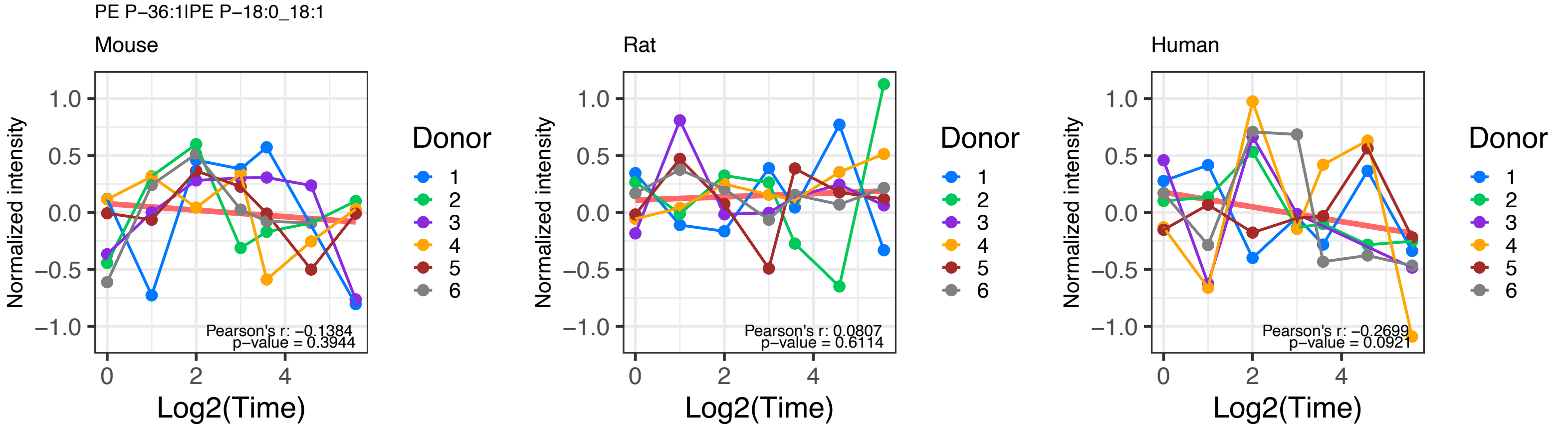
PE P−36:1|PE P−18:0_18:1
Mouse
Rat
Human
Normalized intensity
Log2(Time)
Donor
1
2
3
4
5
6
Pearson's r: −0.1384
p−value = 0.3944
Pearson's r: 0.0807
p−value = 0.6114
Pearson's r: −0.2699
p−value = 0.0921

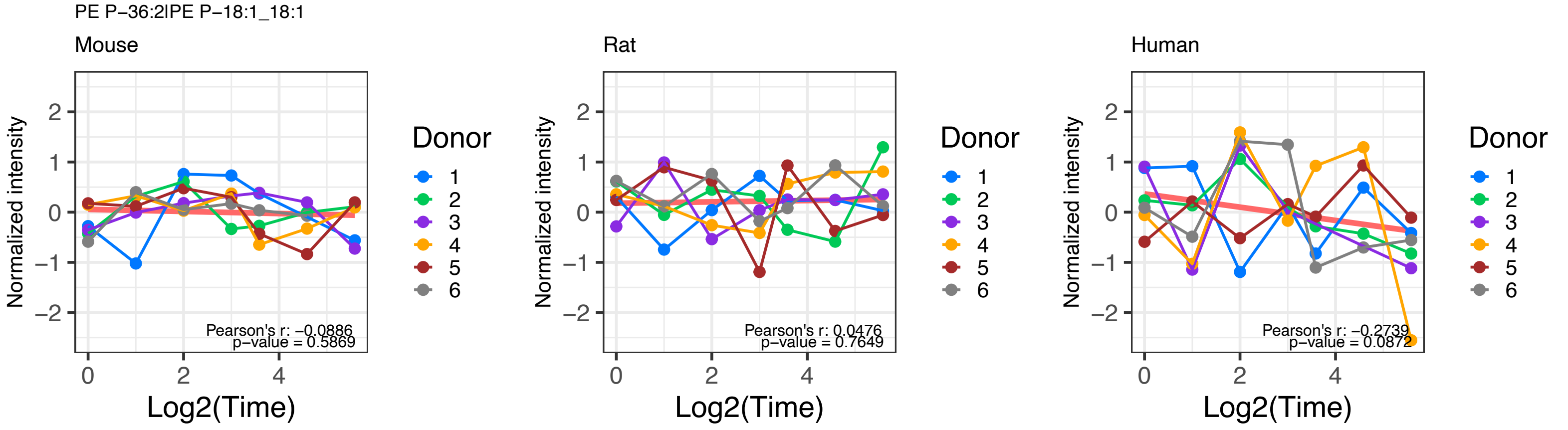
PE P−36:2|PE P−18:1_18:1
Mouse
Rat
Human
Normalized intensity
Log2(Time)
Donor
1
2
3
4
5
6
Pearson's r: −0.0886
p−value = 0.5869
Pearson's r: 0.0476
p−value = 0.7649
Pearson's r: −0.2739
p−value = 0.0872

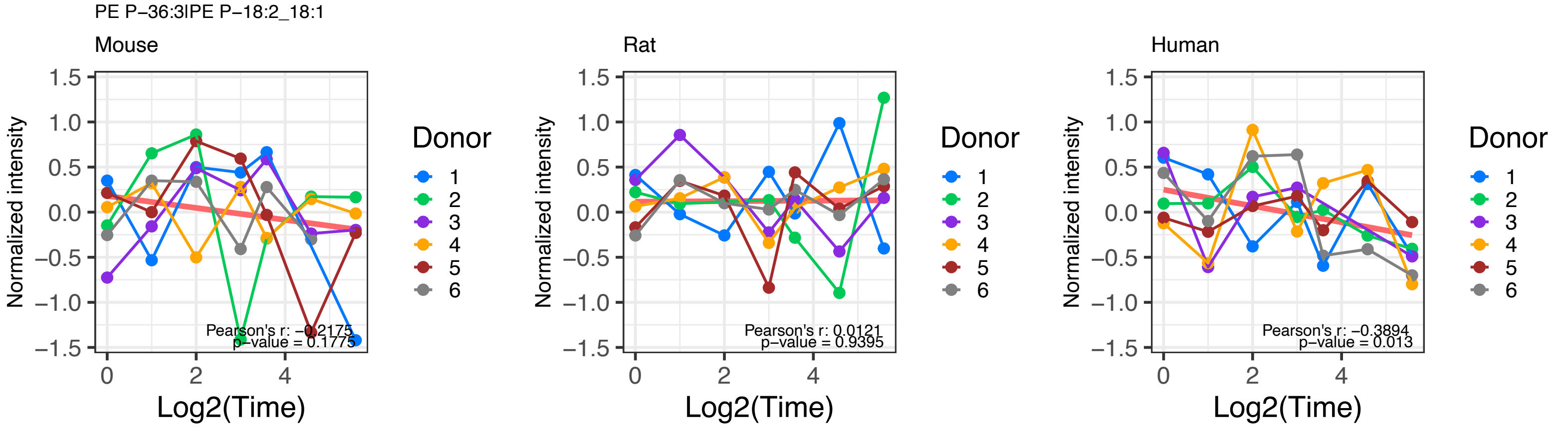
PE P−36:3|PE P−18:2_18:1
Mouse
Rat
Human
Normalized intensity
Log2(Time)
Donor
1
2
3
4
5
6
Pearson's r: −0.2175
p−value = 0.1775
Pearson's r: 0.0121
p−value = 0.9395
Pearson's r: −0.3894
p−value = 0.013

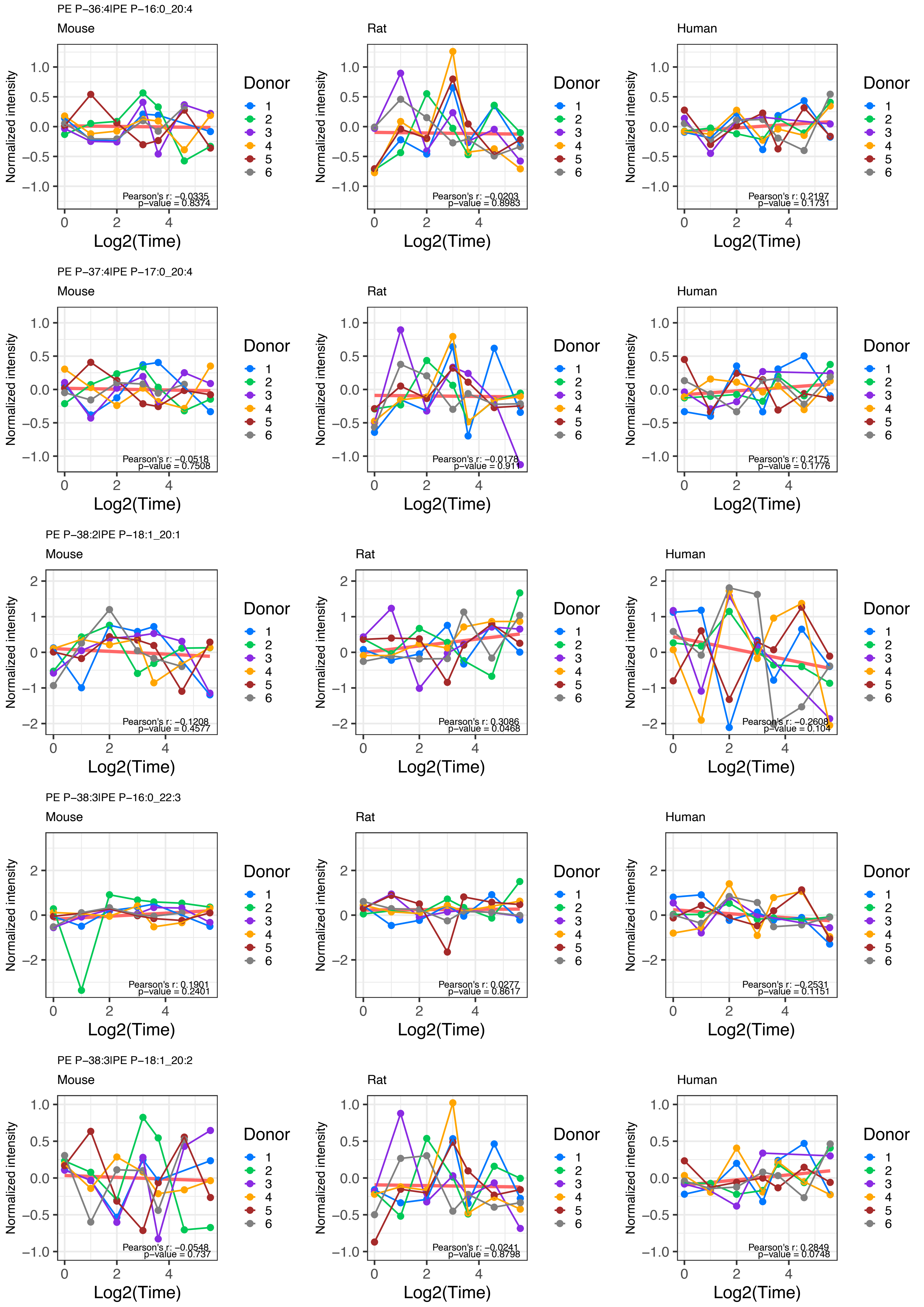
PE P−36:4|PE P−16:0_20:4
Mouse
Pearson's r: −0.0335
p−value = 0.8374
Rat
Pearson's r: −0.0203
p−value = 0.8983
Human
Pearson's r: 0.2197
p−value = 0.1731
PE P−37:4|PE P−17:0_20:4
Mouse
Pearson's r: −0.0518
p−value = 0.7508
Rat
Pearson's r: −0.0178
p−value = 0.911
Human
Pearson's r: 0.2175
p−value = 0.1776
PE P−38:2|PE P−18:1_20:1
Mouse
Pearson's r: −0.1208
p−value = 0.4577
Rat
Pearson's r: 0.3086
p−value = 0.0468
Human
Pearson's r: −0.2608
p−value = 0.104
PE P−38:3|PE P−16:0_22:3
Mouse
Pearson's r: 0.1901
p−value = 0.2401
Rat
Pearson's r: 0.0277
p−value = 0.8617
Human
Pearson's r: −0.2531
p−value = 0.1151
PE P−38:3|PE P−18:1_20:2
Mouse
Pearson's r: −0.0548
p−value = 0.737
Rat
Pearson's r: −0.0241
p−value = 0.8798
Human
Pearson's r: 0.2849
p−value = 0.0748
Normalized intensity
Log2(Time)
Donor
1
2
3
4
5
6

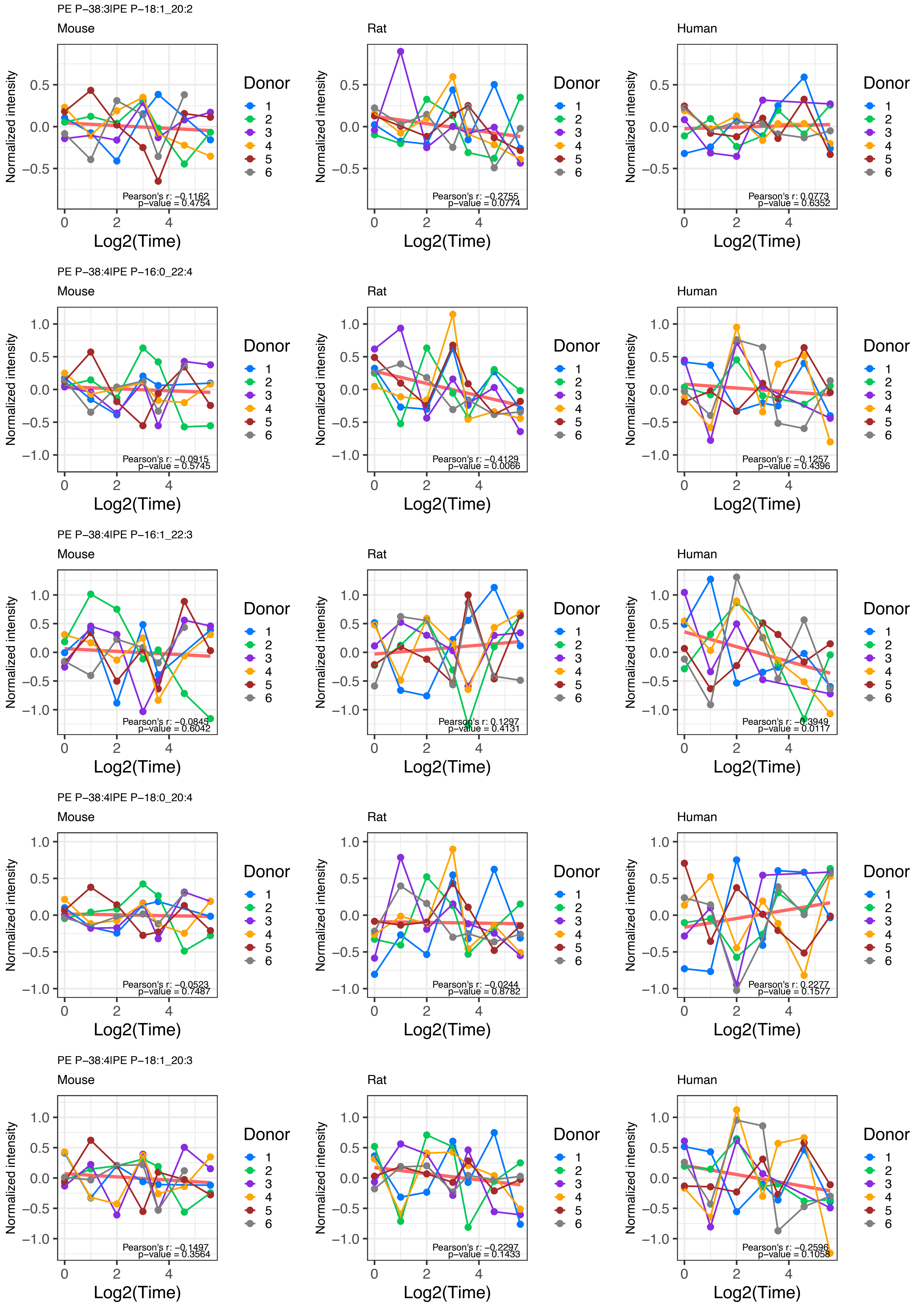

PE P−38:3|PE P−18:1_20:2
Mouse
Pearson's r: −0.1162
p−value = 0.4754
Rat
Pearson's r: −0.2755
p−value = 0.0774
Human
Pearson's r: 0.0773
p−value = 0.6352
PE P−38:4|PE P−16:0_22:4
Mouse
Pearson's r: −0.0915
p−value = 0.5745
Rat
Pearson's r: −0.4129
p−value = 0.0066
Human
Pearson's r: −0.1257
p−value = 0.4396
PE P−38:4|PE P−16:1_22:3
Mouse
Pearson's r: −0.0845
p−value = 0.6042
Rat
Pearson's r: 0.1297
p−value = 0.4131
Human
Pearson's r: −0.3949
p−value = 0.0117
PE P−38:4|PE P−18:0_20:4
Mouse
Pearson's r: −0.0523
p−value = 0.7487
Rat
Pearson's r: −0.0244
p−value = 0.8782
Human
Pearson's r: 0.2277
p−value = 0.1577
PE P−38:4|PE P−18:1_20:3
Mouse
Pearson's r: −0.1497
p−value = 0.3564
Rat
Pearson's r: −0.2297
p−value = 0.1433
Human
Pearson's r: −0.2596
p−value = 0.1058
Normalized intensity
Log2(Time)
Donor
1
2
3
4
5
6

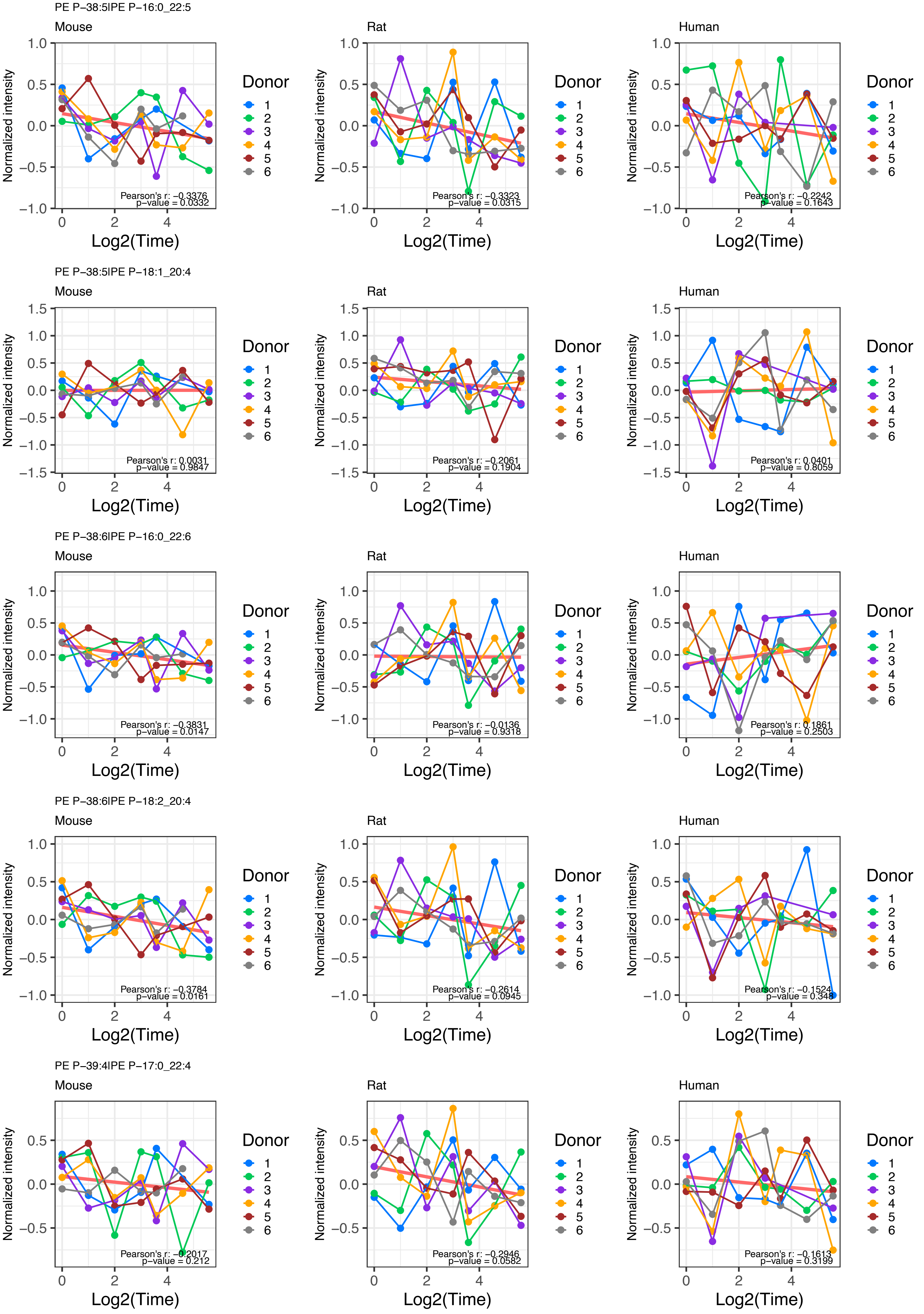
PE P−38:5|PE P−16:0_22:5
Mouse
Pearson's r: −0.3376
p−value = 0.0332
Rat
Pearson's r: −0.3323
p−value = 0.0315
Human
Pearson's r: −0.2242
p−value = 0.1643
PE P−38:5|PE P−18:1_20:4
Mouse
Pearson's r: 0.0031
p−value = 0.9847
Rat
Pearson's r: −0.2061
p−value = 0.1904
Human
Pearson's r: 0.0401
p−value = 0.8059
PE P−38:6|PE P−16:0_22:6
Mouse
Pearson's r: −0.3831
p−value = 0.0147
Rat
Pearson's r: −0.0136
p−value = 0.9318
Human
Pearson's r: 0.1861
p−value = 0.2503
PE P−38:6|PE P−18:2_20:4
Mouse
Pearson's r: −0.3784
p−value = 0.0161
Rat
Pearson's r: −0.2614
p−value = 0.0945
Human
Pearson's r: −0.1524
p−value = 0.348
PE P−39:4|PE P−17:0_22:4
Mouse
Pearson's r: −0.2017
p−value = 0.212
Rat
Pearson's r: −0.2946
p−value = 0.0582
Human
Pearson's r: −0.1613
p−value = 0.3199
Normalized intensity
Log2(Time)
Donor
1
2
3
4
5
6

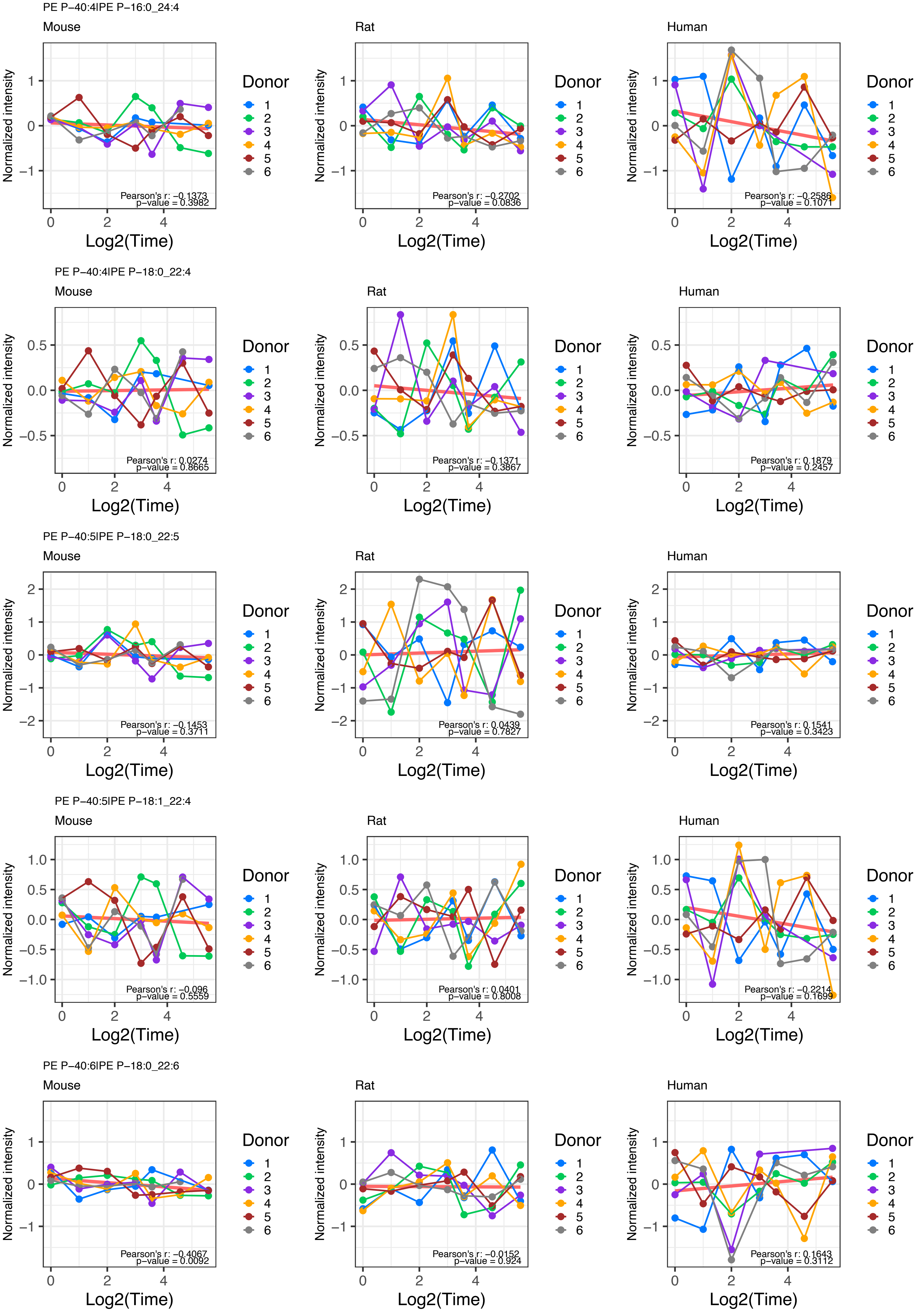
PE P−40:4|PE P−16:0_24:4
Mouse
Pearson's r: −0.1373
p−value = 0.3982
Rat
Pearson's r: −0.2702
p−value = 0.0836
Human
Pearson's r: −0.2586
p−value = 0.1071
PE P−40:4|PE P−18:0_22:4
Mouse
Pearson's r: 0.0274
p−value = 0.8665
Rat
Pearson's r: −0.1371
p−value = 0.3867
Human
Pearson's r: 0.1879
p−value = 0.2457
PE P−40:5|PE P−18:0_22:5
Mouse
Pearson's r: −0.1453
p−value = 0.3711
Rat
Pearson's r: 0.0439
p−value = 0.7827
Human
Pearson's r: 0.1541
p−value = 0.3423
PE P−40:5|PE P−18:1_22:4
Mouse
Pearson's r: −0.096
p−value = 0.5559
Rat
Pearson's r: 0.0401
p−value = 0.8008
Human
Pearson's r: −0.2214
p−value = 0.1699
PE P−40:6|PE P−18:0_22:6
Mouse
Pearson's r: −0.4067
p−value = 0.0092
Rat
Pearson's r: −0.0152
p−value = 0.924
Human
Pearson's r: 0.1643
p−value = 0.3112
Normalized intensity
Log2(Time)
Donor
1
2
3
4
5
6

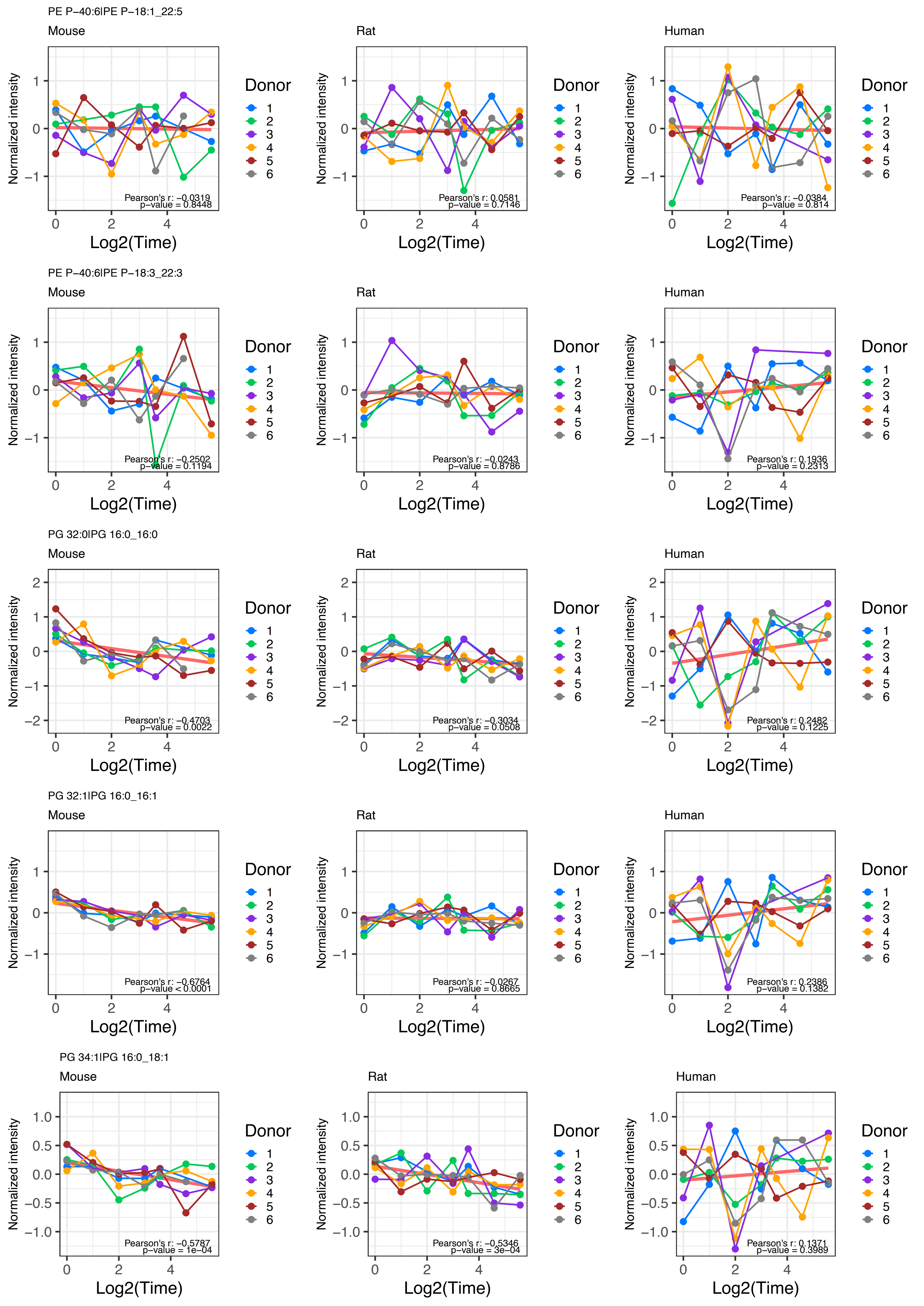
PE P−40:6|PE P−18:1_22:5
Mouse
Pearson's r: −0.0319
p−value = 0.8448
Rat
Pearson's r: 0.0581
p−value = 0.7146
Human
Pearson's r: −0.0384
p−value = 0.814
PE P−40:6|PE P−18:3_22:3
Mouse
Pearson's r: −0.2502
p−value = 0.1194
Rat
Pearson's r: −0.0243
p−value = 0.8786
Human
Pearson's r: 0.1936
p−value = 0.2313
PG 32:0|PG 16:0_16:0
Mouse
Pearson's r: −0.4703
p−value = 0.0022
Rat
Pearson's r: −0.3034
p−value = 0.0508
Human
Pearson's r: 0.2482
p−value = 0.1225
PG 32:1|PG 16:0_16:1
Mouse
Pearson's r: −0.6764
p−value < 0.0001
Rat
Pearson's r: −0.0267
p−value = 0.8665
Human
Pearson's r: 0.2386
p−value = 0.1382
PG 34:1|PG 16:0_18:1
Mouse
Pearson's r: −0.5787
p−value = 1e−04
Rat
Pearson's r: −0.5346
p−value = 3e−04
Human
Pearson's r: 0.1371
p−value = 0.3989
Normalized intensity
Log2(Time)
Donor
1
2
3
4
5
6

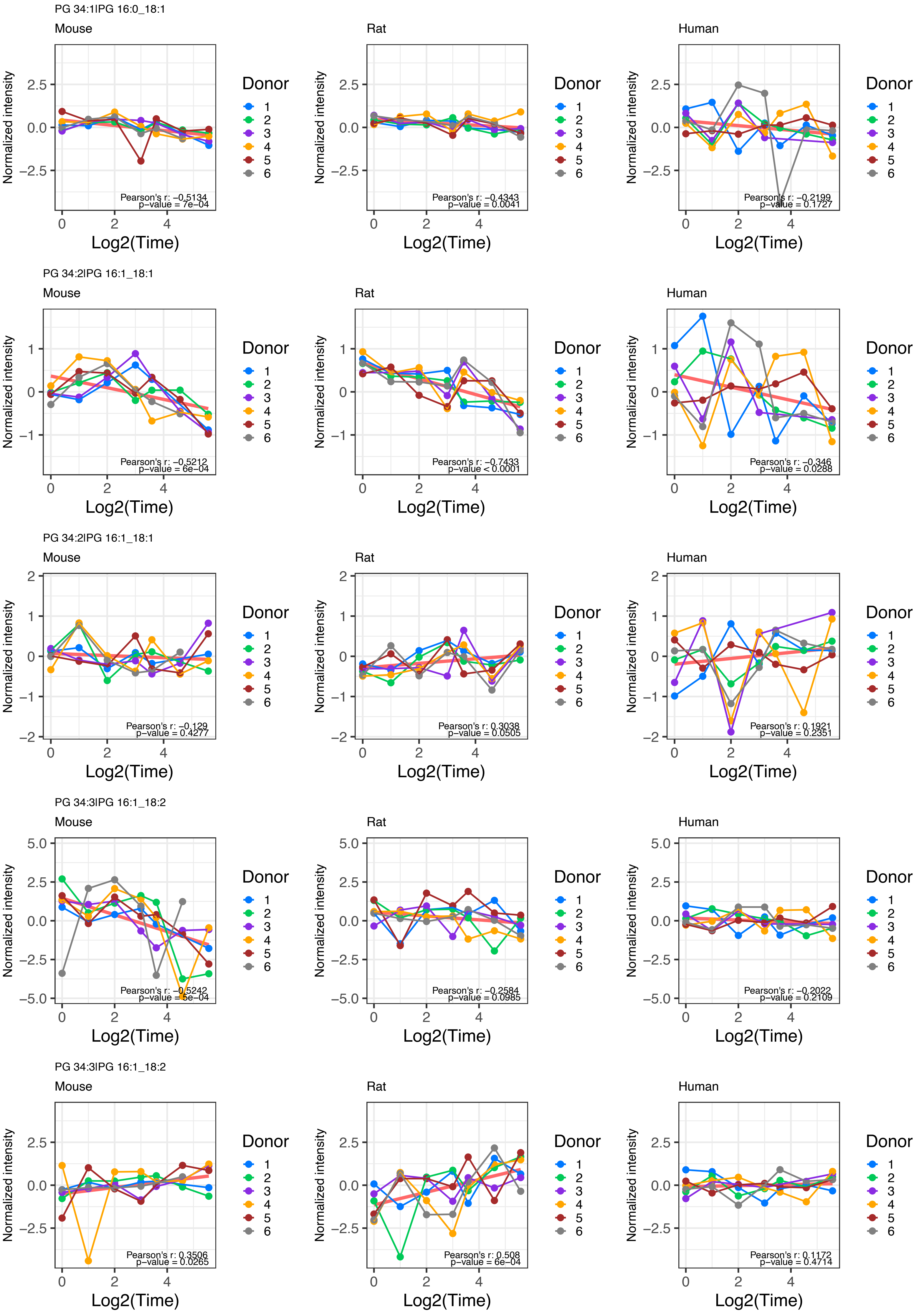
PG 34:1|PG 16:0_18:1
Mouse
Rat
Human
Normalized intensity
Log2(Time)
Donor
1
2
3
4
5
6
Pearson's r: −0.5134
p−value = 7e−04
Pearson's r: −0.4343
p−value = 0.0041
Pearson's r: −0.2199
p−value = 0.1727
PG 34:2|PG 16:1_18:1
Pearson's r: −0.5212
p−value = 6e−04
Pearson's r: −0.7433
p−value < 0.0001
Pearson's r: −0.346
p−value = 0.0288
PG 34:2|PG 16:1_18:1
Pearson's r: −0.129
p−value = 0.4277
Pearson's r: 0.3038
p−value = 0.0505
Pearson's r: 0.1921
p−value = 0.2351
PG 34:3|PG 16:1_18:2
Pearson's r: −0.5242
p−value = 5e−04
Pearson's r: −0.2584
p−value = 0.0985
Pearson's r: −0.2022
p−value = 0.2109
PG 34:3|PG 16:1_18:2
Pearson's r: 0.3506
p−value = 0.0265
Pearson's r: 0.508
p−value = 6e−04
Pearson's r: 0.1172
p−value = 0.4714

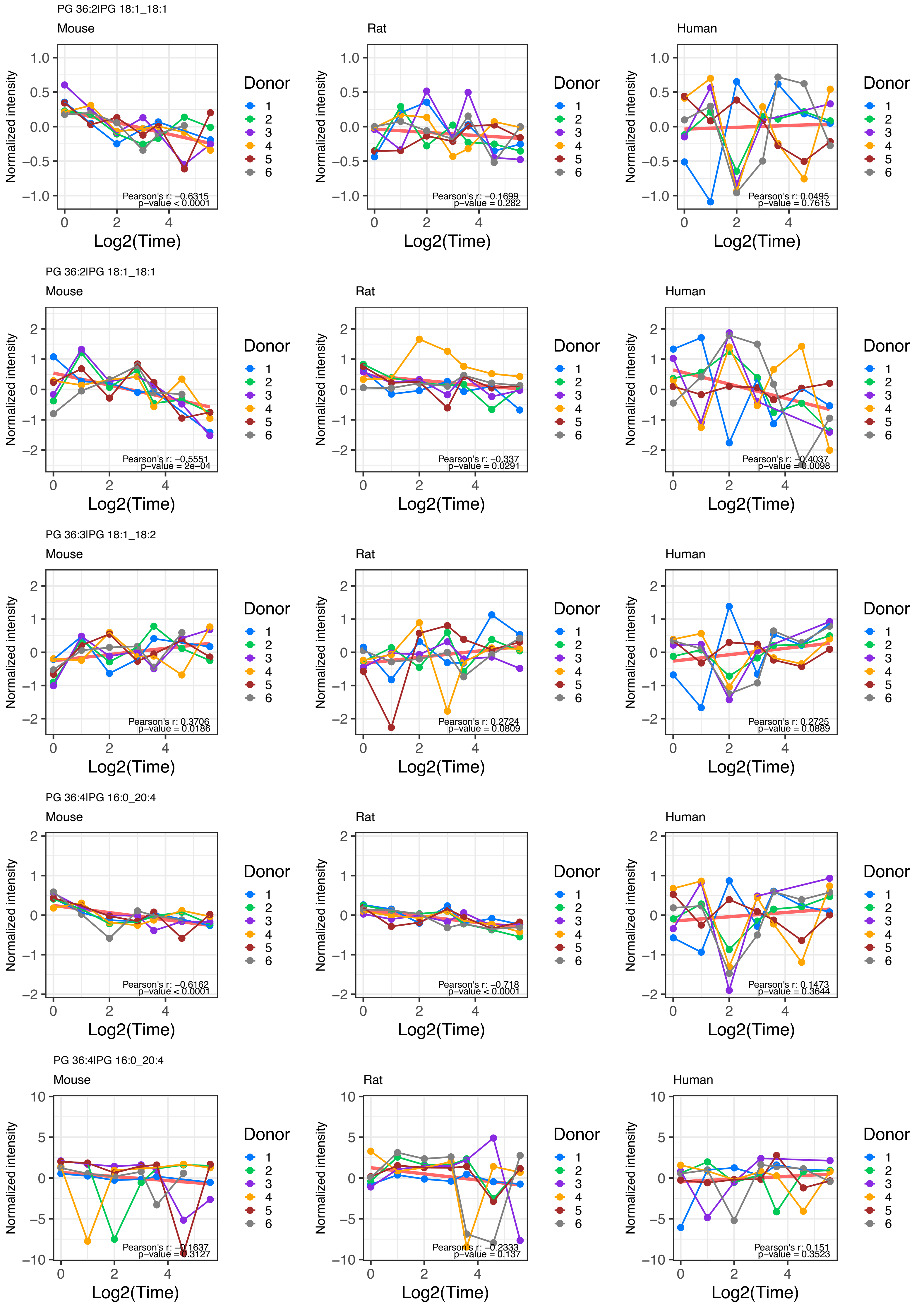
PG 36:2|PG 18:1_18:1
Mouse
Rat
Human
Normalized intensity
Log2(Time)
Donor
1
2
3
4
5
6
Pearson's r: −0.6315
p−value < 0.0001
Pearson's r: −0.1699
p−value = 0.282
Pearson's r: 0.0495
p−value = 0.7615
PG 36:2|PG 18:1_18:1
Pearson's r: −0.5551
p−value = 2e−04
Pearson's r: −0.337
p−value = 0.0291
Pearson's r: −0.4037
p−value = 0.0098
PG 36:3|PG 18:1_18:2
Pearson's r: 0.3706
p−value = 0.0186
Pearson's r: 0.2724
p−value = 0.0809
Pearson's r: 0.2725
p−value = 0.0889
PG 36:4|PG 16:0_20:4
Pearson's r: −0.6162
p−value < 0.0001
Pearson's r: −0.718
p−value < 0.0001
Pearson's r: 0.1473
p−value = 0.3644
PG 36:4|PG 16:0_20:4
Pearson's r: −0.1637
p−value = 0.3127
Pearson's r: −0.2333
p−value = 0.137
Pearson's r: 0.151
p−value = 0.3523

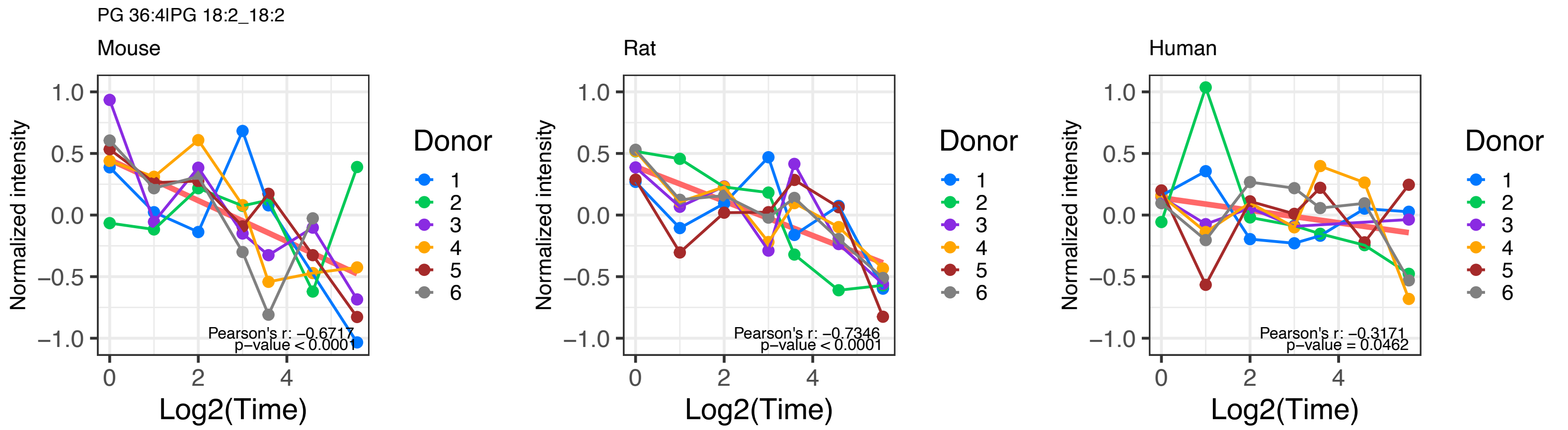
PG 36:4|PG 18:2_18:2
Mouse
Rat
Human
Normalized intensity
Log2(Time)
Donor
1
2
3
4
5
6
Pearson's r: −0.6717
p−value < 0.0001
Pearson's r: −0.7346
p−value < 0.0001
Pearson's r: −0.3171
p−value = 0.0462

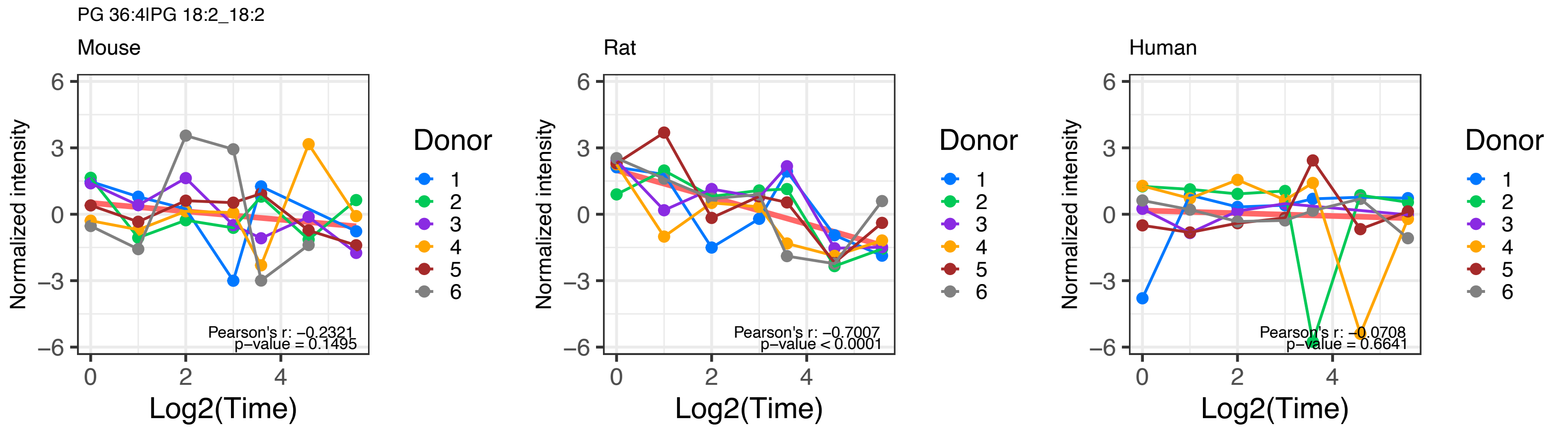
PG 36:4|PG 18:2_18:2
Mouse
Rat
Human
Normalized intensity
Log2(Time)
Donor
1
2
3
4
5
6
Pearson's r: −0.2321
p−value = 0.1495
Pearson's r: −0.7007
p−value < 0.0001
Pearson's r: −0.0708
p−value = 0.6641

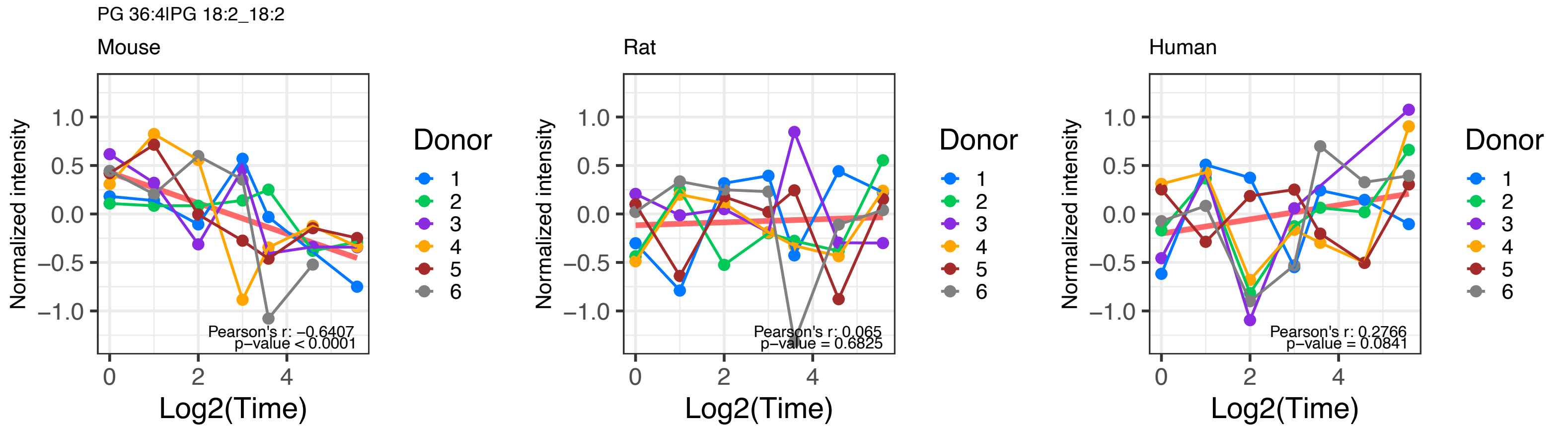
PG 36:4|PG 18:2_18:2
Mouse
Rat
Human
Normalized intensity
Log2(Time)
Donor
1
2
3
4
5
6
Pearson's r: −0.6407
p−value < 0.0001
Pearson's r: 0.065
p−value = 0.6825
Pearson's r: 0.2766
p−value = 0.0841

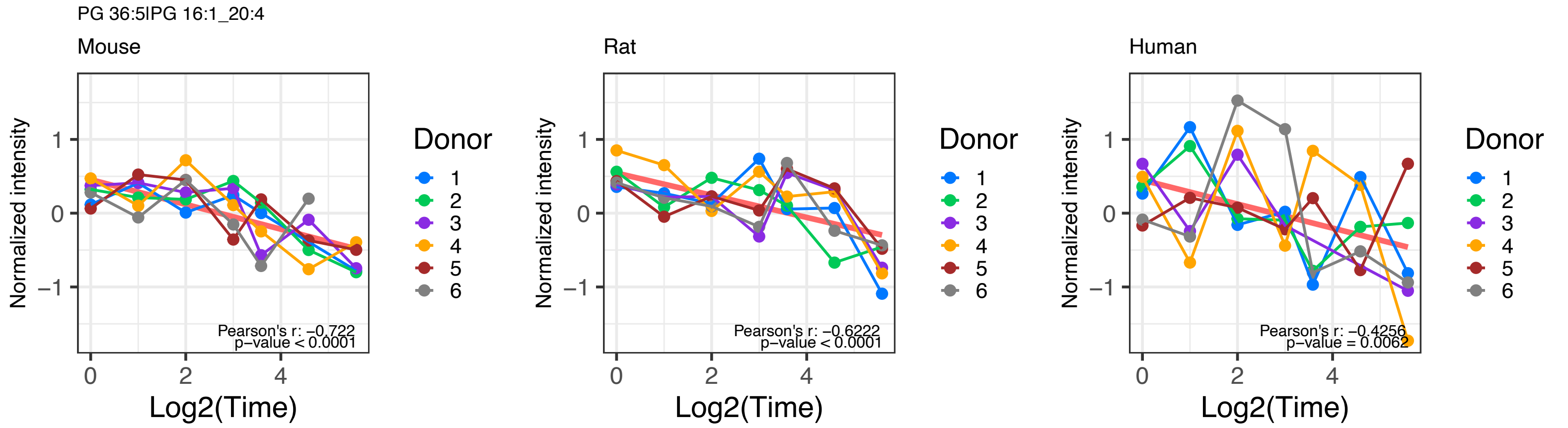
PG 36:5|PG 16:1_20:4
Mouse
Rat
Human
Normalized intensity
Log2(Time)
Donor
1
2
3
4
5
6
Pearson's r: −0.722
p−value < 0.0001
Pearson's r: −0.6222
p−value < 0.0001
Pearson's r: −0.4256
p−value = 0.0062

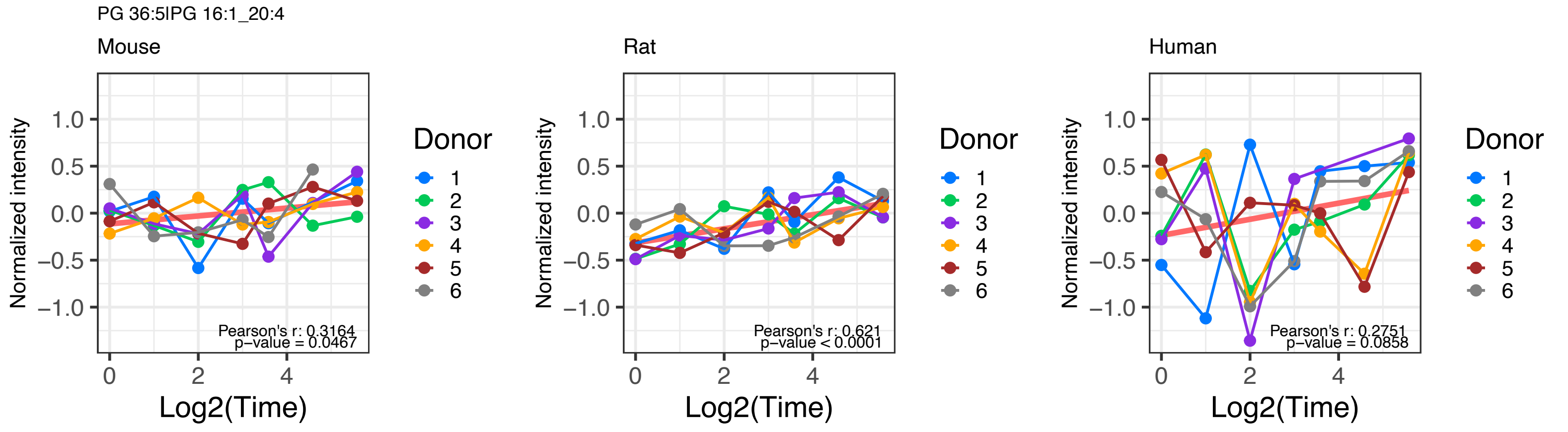
PG 36:5|PG 16:1_20:4
Mouse
Rat
Human
Normalized intensity
Log2(Time)
Donor
1
2
3
4
5
6
Pearson's r: 0.3164
p−value = 0.0467
Pearson's r: 0.621
p−value < 0.0001
Pearson's r: 0.2751
p−value = 0.0858

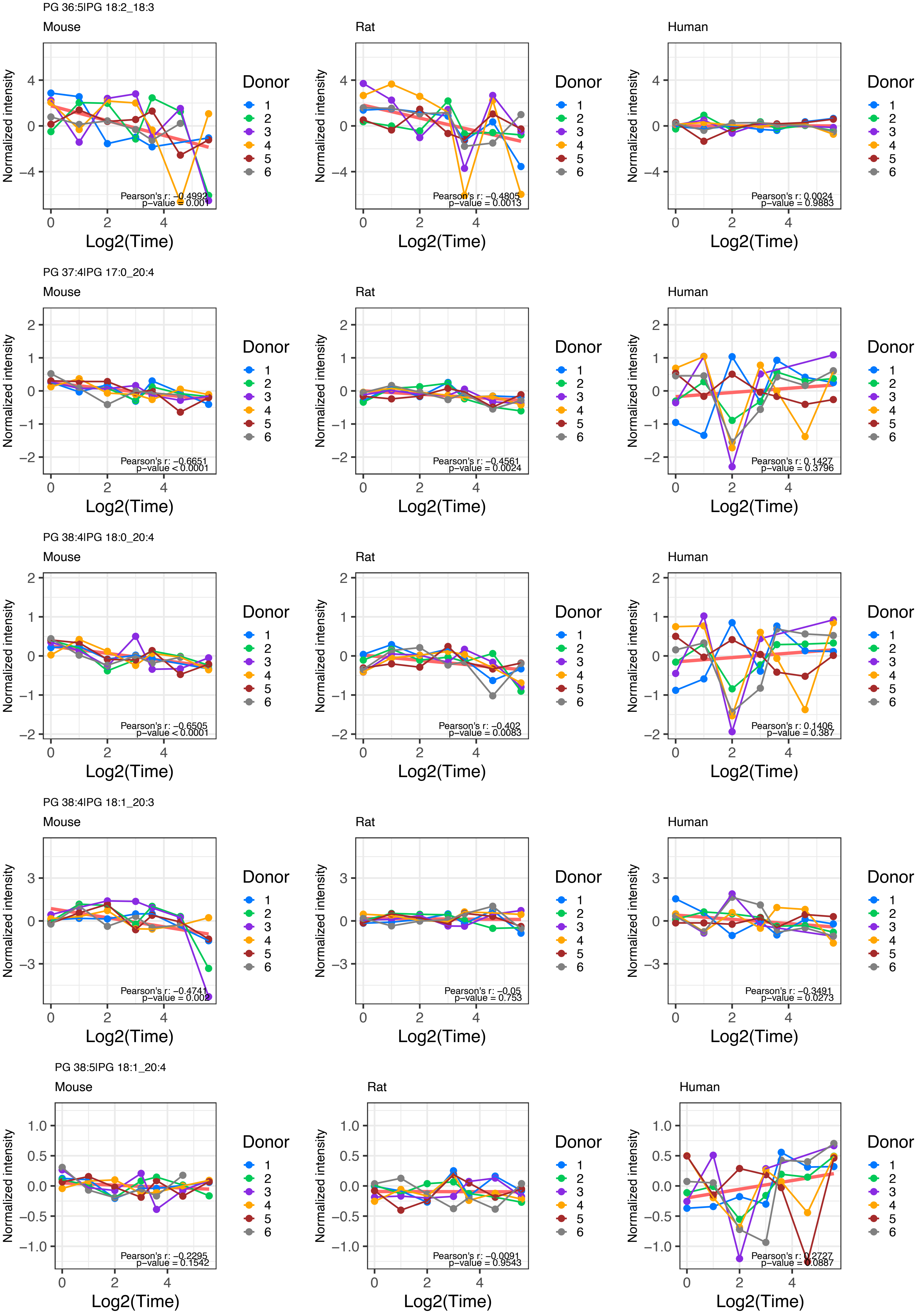
PG 36:5|PG 18:2_18:3
Mouse
Pearson's r: −0.4992
p−value = 0.001
Rat
Pearson's r: −0.4805
p−value = 0.0013
Human
Pearson's r: 0.0024
p−value = 0.9883
PG 37:4|PG 17:0_20:4
Mouse
Pearson's r: −0.6651
p−value < 0.0001
Rat
Pearson's r: −0.4561
p−value = 0.0024
Human
Pearson's r: 0.1427
p−value = 0.3796
PG 38:4|PG 18:0_20:4
Mouse
Pearson's r: −0.6505
p−value < 0.0001
Rat
Pearson's r: −0.402
p−value = 0.0083
Human
Pearson's r: 0.1406
p−value = 0.387
PG 38:4|PG 18:1_20:3
Mouse
Pearson's r: −0.4741
p−value = 0.002
Rat
Pearson's r: −0.05
p−value = 0.753
Human
Pearson's r: −0.3491
p−value = 0.0273
PG 38:5|PG 18:1_20:4
Mouse
Pearson's r: −0.2295
p−value = 0.1542
Rat
Pearson's r: −0.0091
p−value = 0.9543
Human
Pearson's r: 0.2727
p−value = 0.0887
Normalized intensity
Log2(Time)
Donor
1
2
3
4
5
6

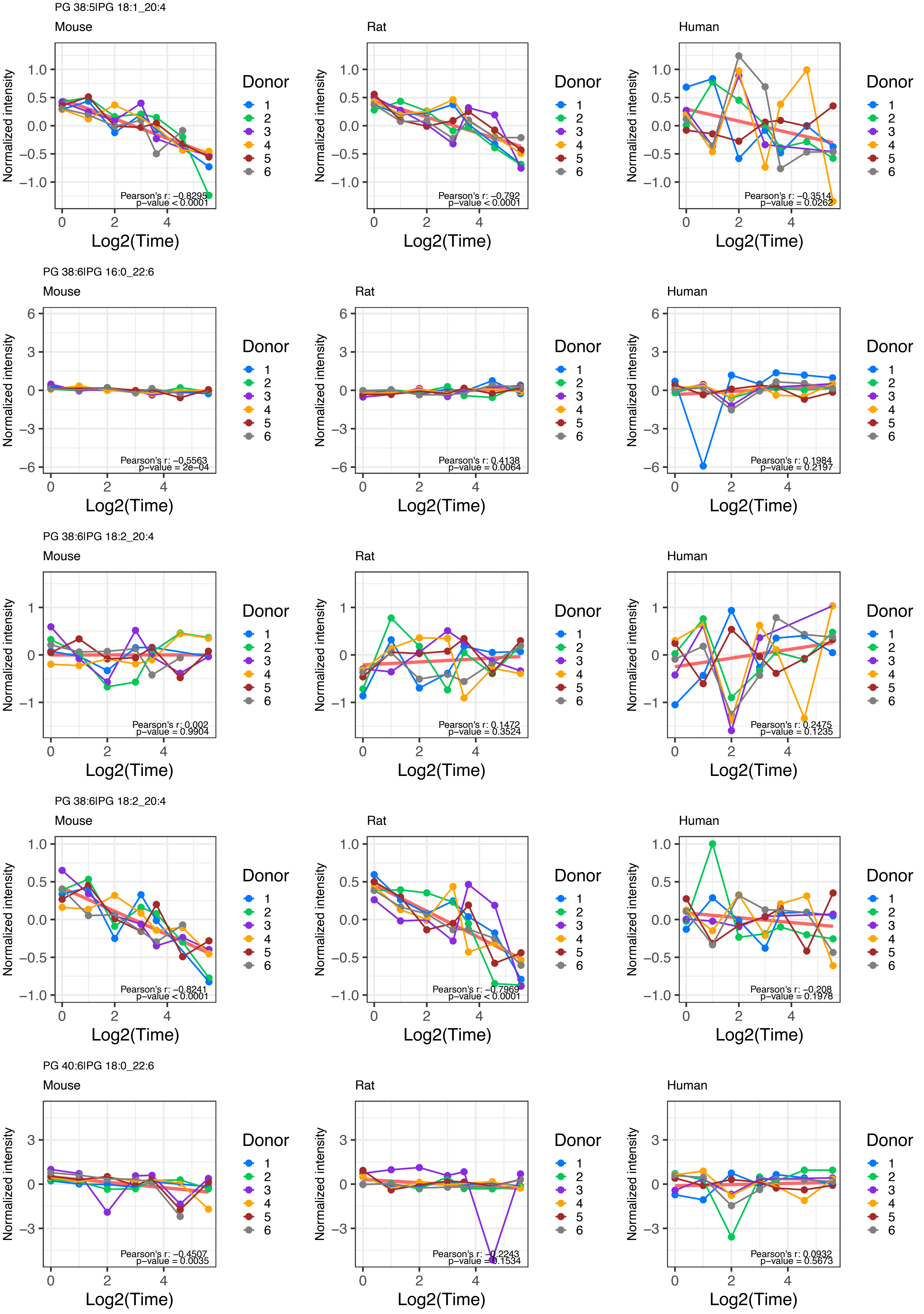
PG 38:5|PG 18:1_20:4
Mouse
Pearson's r: −0.8295
p−value < 0.0001
Rat
Pearson's r: −0.792
p−value < 0.0001
Human
Pearson's r: −0.3514
p−value = 0.0262
PG 38:6|PG 16:0_22:6
Mouse
Pearson's r: −0.5563
p−value = 2e−04
Rat
Pearson's r: 0.4138
p−value = 0.0064
Human
Pearson's r: 0.1984
p−value = 0.2197
PG 38:6|PG 18:2_20:4
Mouse
Pearson's r: 0.002
p−value = 0.9904
Rat
Pearson's r: 0.1472
p−value = 0.3524
Human
Pearson's r: 0.2475
p−value = 0.1235
PG 38:6|PG 18:2_20:4
Mouse
Pearson's r: −0.8241
p−value < 0.0001
Rat
Pearson's r: −0.7969
p−value < 0.0001
Human
Pearson's r: −0.208
p−value = 0.1978
PG 40:6|PG 18:0_22:6
Mouse
Pearson's r: −0.4507
p−value = 0.0035
Rat
Pearson's r: −0.2243
p−value = 0.1534
Human
Pearson's r: 0.0932
p−value = 0.5673
Normalized intensity
Log2(Time)
Donor
1
2
3
4
5
6

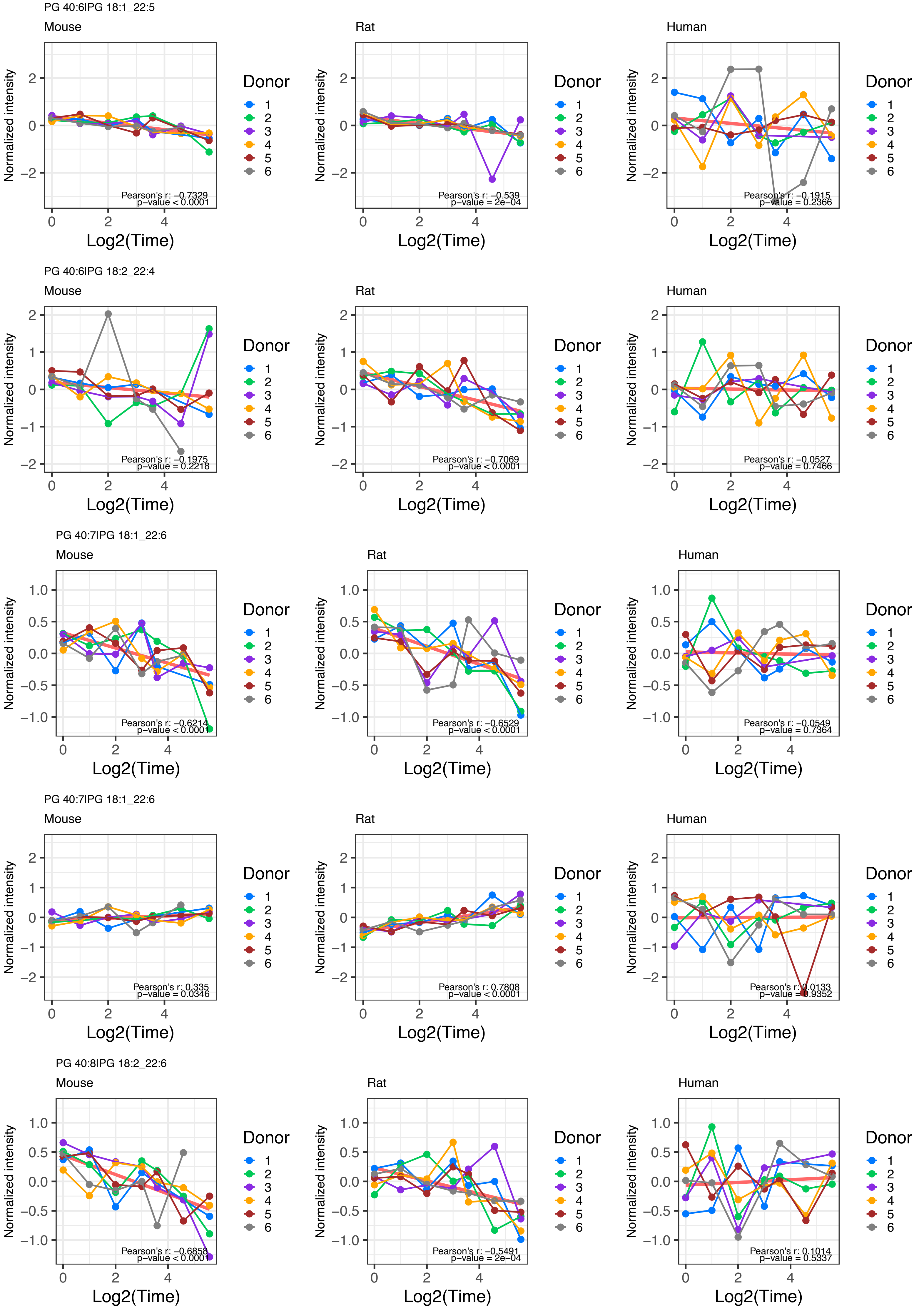

PG 40:6|PG 18:1_22:5
Mouse
Pearson's r: −0.7329
p−value < 0.0001
Rat
Pearson's r: −0.539
p−value = 2e−04
Human
Pearson's r: −0.1915
p−value = 0.2366
PG 40:6|PG 18:2_22:4
Mouse
Pearson's r: −0.1975
p−value = 0.2218
Rat
Pearson's r: −0.7069
p−value < 0.0001
Human
Pearson's r: −0.0527
p−value = 0.7466
PG 40:7|PG 18:1_22:6
Mouse
Pearson's r: −0.6214
p−value < 0.0001
Rat
Pearson's r: −0.6529
p−value < 0.0001
Human
Pearson's r: −0.0549
p−value = 0.7364
PG 40:7|PG 18:1_22:6
Mouse
Pearson's r: 0.335
p−value = 0.0346
Rat
Pearson's r: 0.7808
p−value < 0.0001
Human
Pearson's r: 0.0133
p−value = 0.9352
PG 40:8|PG 18:2_22:6
Mouse
Pearson's r: −0.6858
p−value < 0.0001
Rat
Pearson's r: −0.5491
p−value = 2e−04
Human
Pearson's r: 0.1014
p−value = 0.5337
Normalized intensity
Log2(Time)
Donor
1
2
3
4
5
6

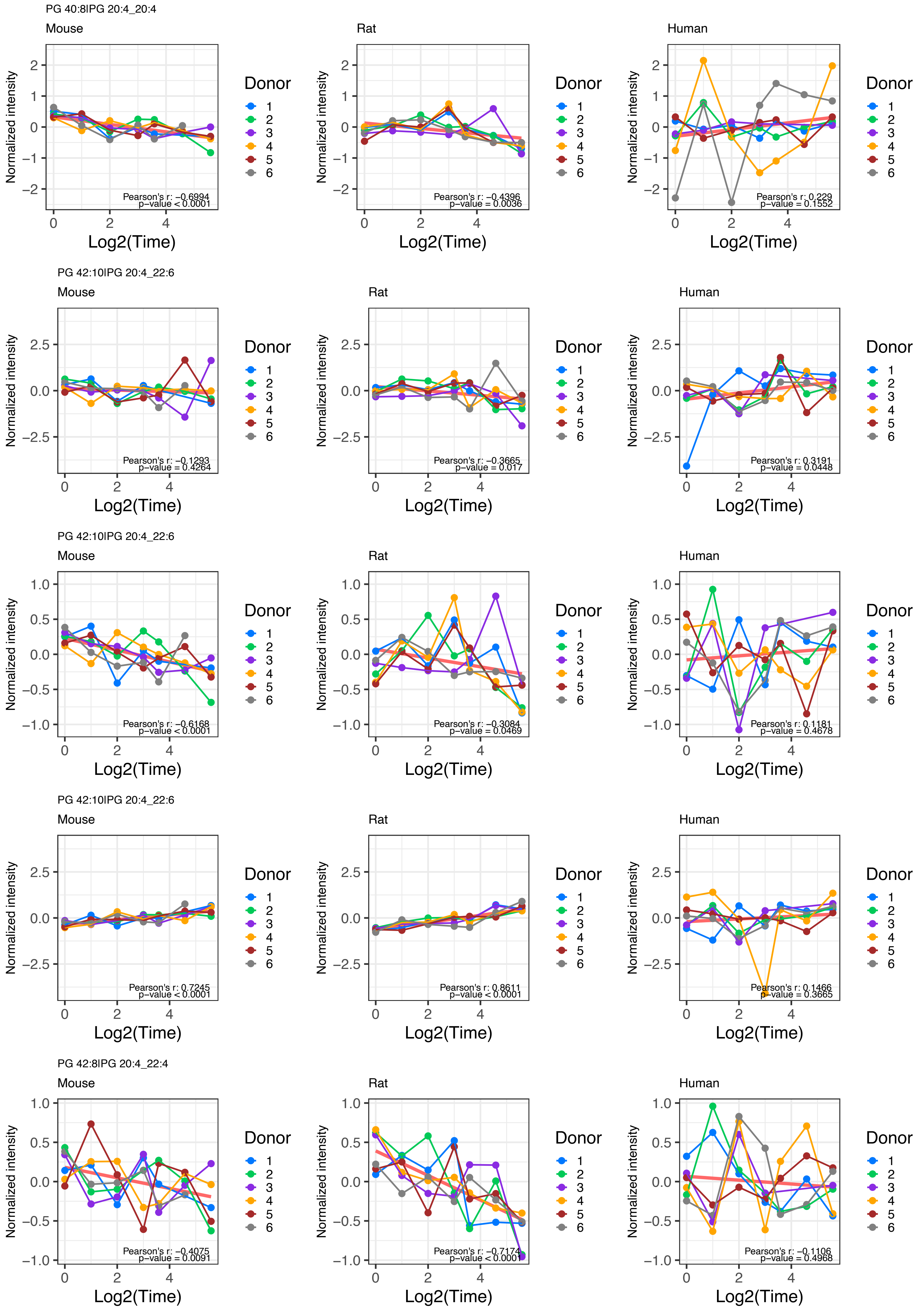

PG 40:8|PG 20:4_20:4
Mouse
Rat
Human
Normalized intensity
Log2(Time)
Donor
1
2
3
4
5
6
Pearson's r: −0.6994
p−value < 0.0001
Pearson's r: −0.4396
p−value = 0.0036
Pearson's r: 0.229
p−value = 0.1552
PG 42:10|PG 20:4_22:6
Mouse
Rat
Human
Pearson's r: −0.1293
p−value = 0.4264
Pearson's r: −0.3665
p−value = 0.017
Pearson's r: 0.3191
p−value = 0.0448
PG 42:10|PG 20:4_22:6
Mouse
Rat
Human
Pearson's r: −0.6168
p−value < 0.0001
Pearson's r: −0.3084
p−value = 0.0469
Pearson's r: 0.1181
p−value = 0.4678
PG 42:10|PG 20:4_22:6
Mouse
Rat
Human
Pearson's r: 0.7245
p−value < 0.0001
Pearson's r: 0.8611
p−value < 0.0001
Pearson's r: 0.1466
p−value = 0.3665
PG 42:8|PG 20:4_22:4
Mouse
Rat
Human
Pearson's r: −0.4075
p−value = 0.0091
Pearson's r: −0.7174
p−value < 0.0001
Pearson's r: −0.1106
p−value = 0.4968

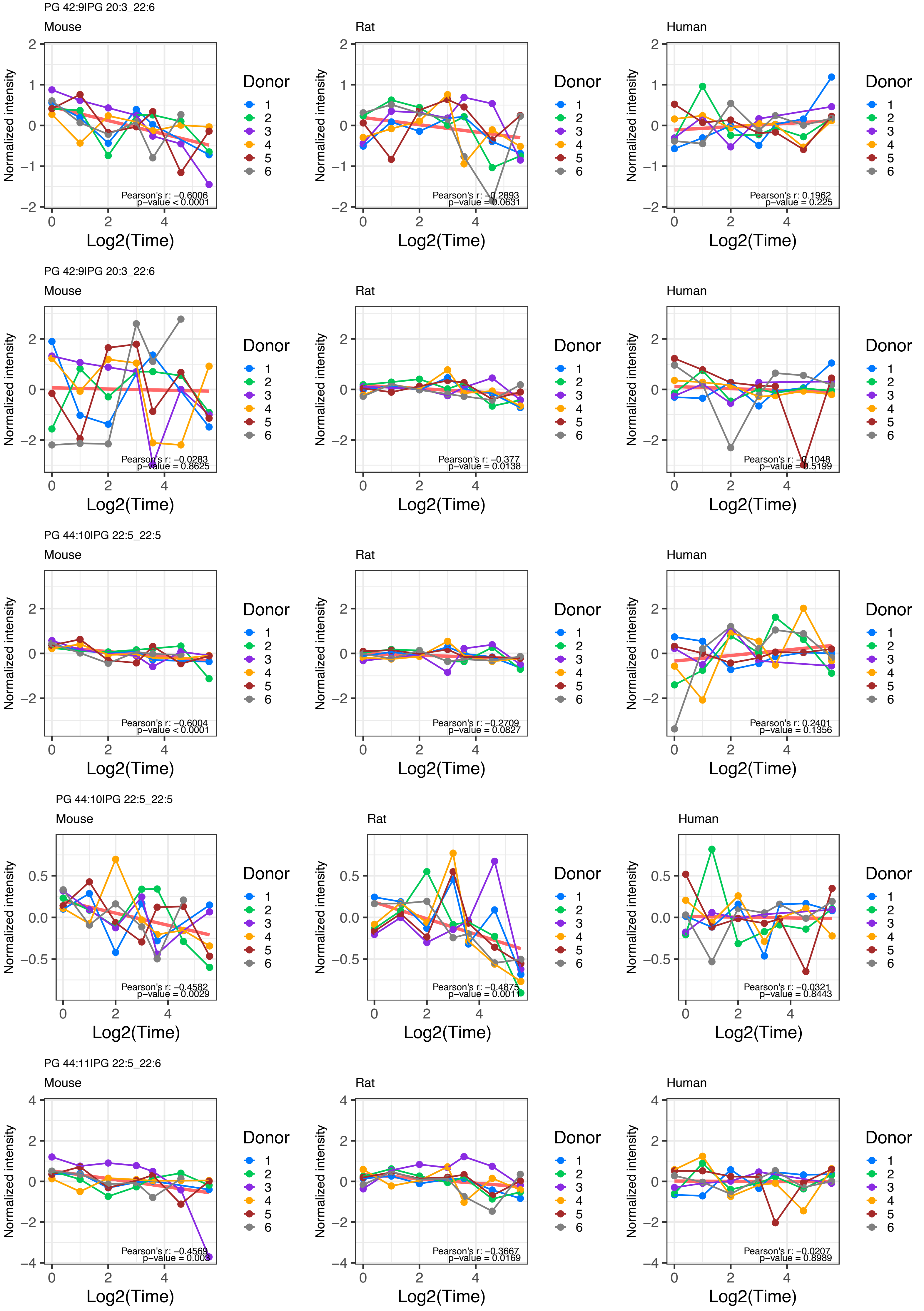

PG 42:9|PG 20:3_22:6
Mouse
Rat
Human
Normalized intensity
Log2(Time)
Donor
1
2
3
4
5
6
Pearson's r: −0.6006
p−value < 0.0001
Pearson's r: −0.2893
p−value = 0.0631
Pearson's r: 0.1962
p−value = 0.225
PG 42:9|PG 20:3_22:6
Mouse
Rat
Human
Pearson's r: −0.0283
p−value = 0.8625
Pearson's r: −0.377
p−value = 0.0138
Pearson's r: −0.1048
p−value = 0.5199
PG 44:10|PG 22:5_22:5
Mouse
Rat
Human
Pearson's r: −0.6004
p−value < 0.0001
Pearson's r: −0.2709
p−value = 0.0827
Pearson's r: 0.2401
p−value = 0.1356
PG 44:10|PG 22:5_22:5
Mouse
Rat
Human
Pearson's r: −0.4582
p−value = 0.0029
Pearson's r: −0.4875
p−value = 0.0011
Pearson's r: −0.0321
p−value = 0.8443
PG 44:11|PG 22:5_22:6
Mouse
Rat
Human
Pearson's r: −0.4569
p−value = 0.003
Pearson's r: −0.3667
p−value = 0.0169
Pearson's r: −0.0207
p−value = 0.8989

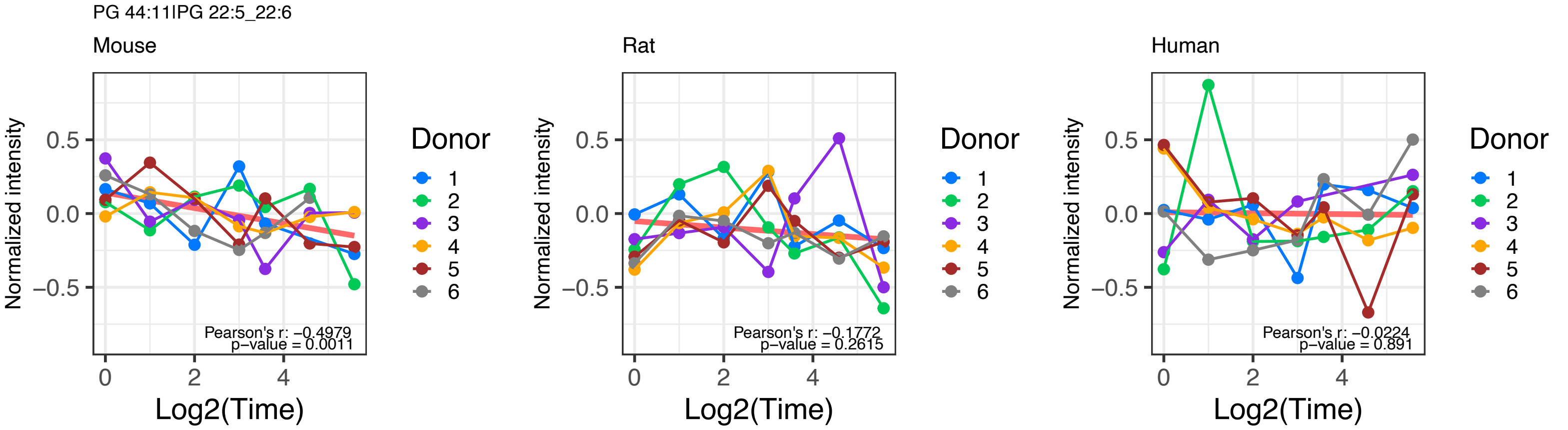
PG 44:11|PG 22:5_22:6
Mouse
Rat
Human
Normalized intensity
Log2(Time)
Donor
Pearson's r: −0.4979
p−value = 0.0011
Pearson's r: −0.1772
p−value = 0.2615
Pearson's r: −0.0224
p−value = 0.891

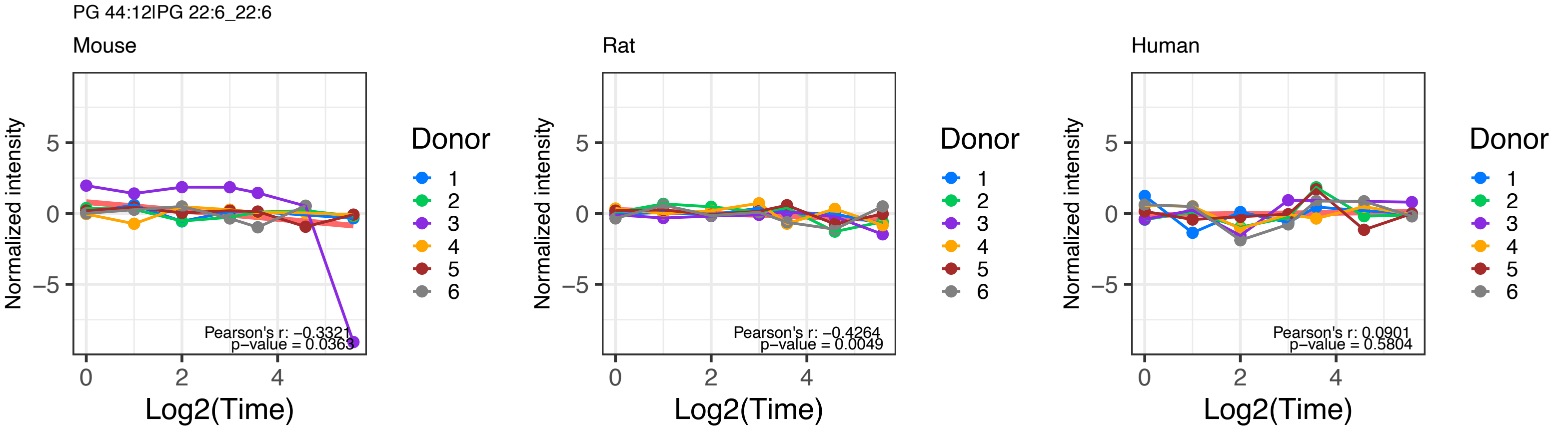
PG 44:12|PG 22:6_22:6
Mouse
Rat
Human
Normalized intensity
Log2(Time)
Donor
Pearson's r: −0.3321
p−value = 0.0363
Pearson's r: −0.4264
p−value = 0.0049
Pearson's r: 0.0901
p−value = 0.5804

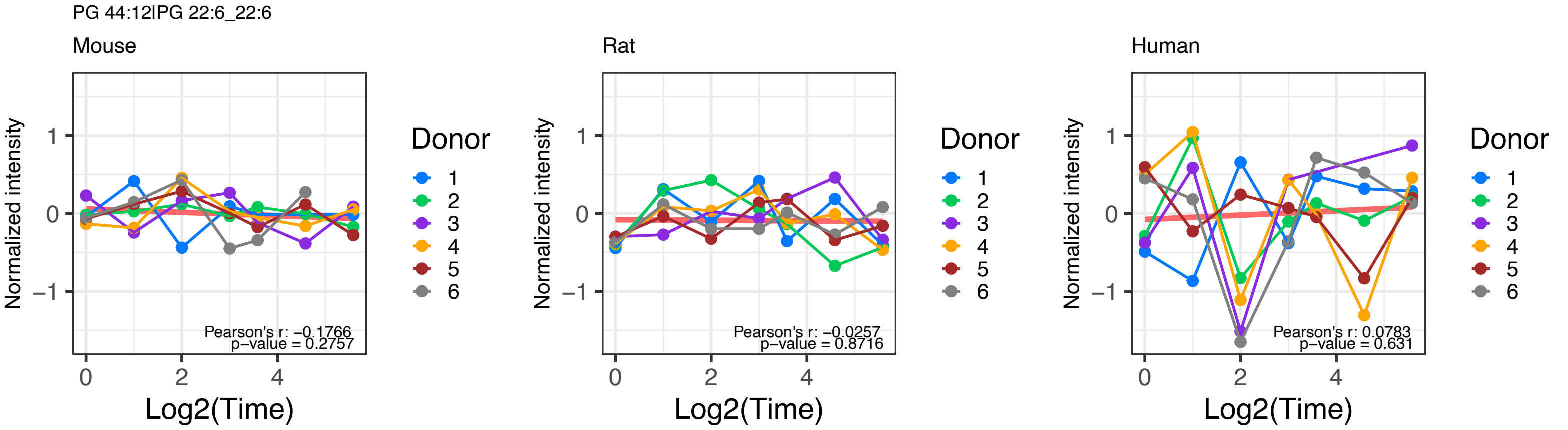
PG 44:12|PG 22:6_22:6
Mouse
Rat
Human
Normalized intensity
Log2(Time)
Donor
Pearson's r: −0.1766
p−value = 0.2757
Pearson's r: −0.0257
p−value = 0.8716
Pearson's r: 0.0783
p−value = 0.631

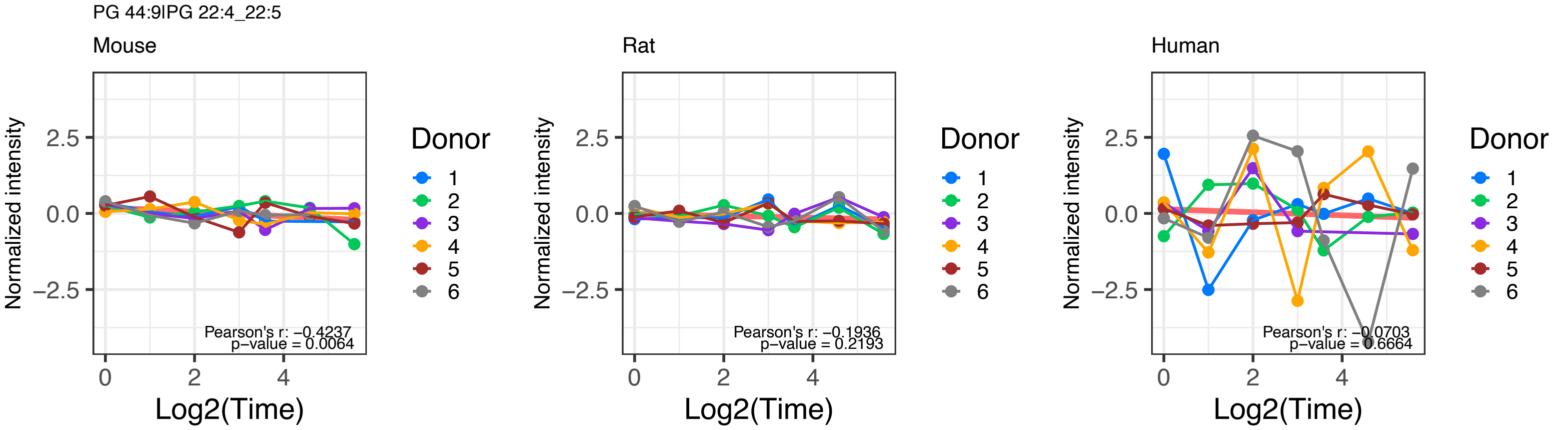
PG 44:9|PG 22:4_22:5
Mouse
Rat
Human
Normalized intensity
Log2(Time)
Donor
Pearson's r: −0.4237
p−value = 0.0064
Pearson's r: −0.1936
p−value = 0.2193
Pearson's r: −0.0703
p−value = 0.6664

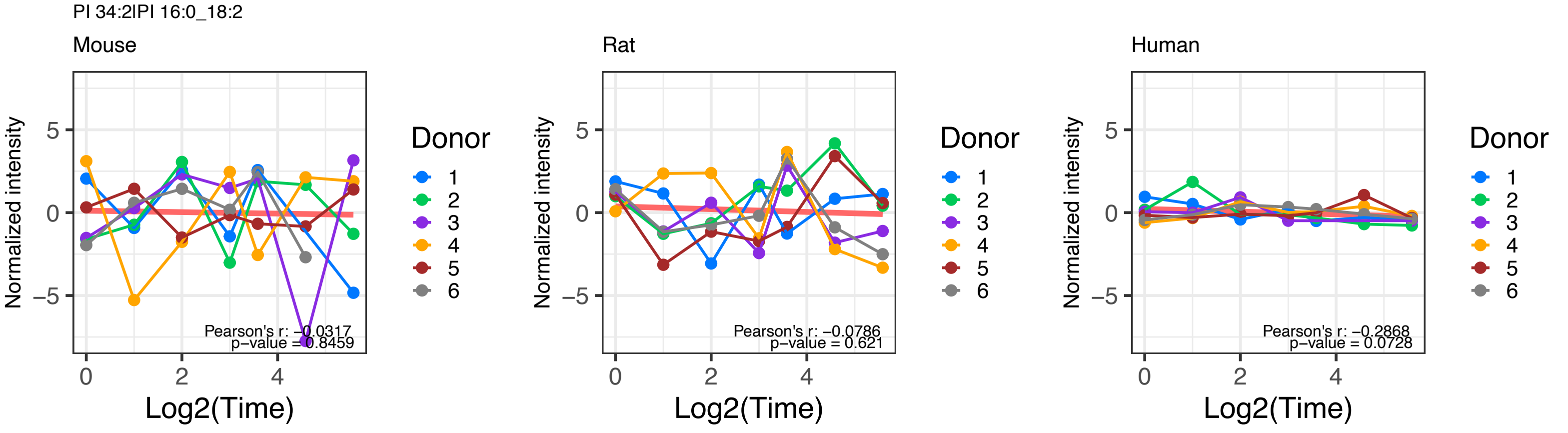
PI 34:2|PI 16:0_18:2
Mouse
Rat
Human
Normalized intensity
Log2(Time)
Donor
Pearson's r: −0.0317
p−value = 0.8459
Pearson's r: −0.0786
p−value = 0.621
Pearson's r: −0.2868
p−value = 0.0728

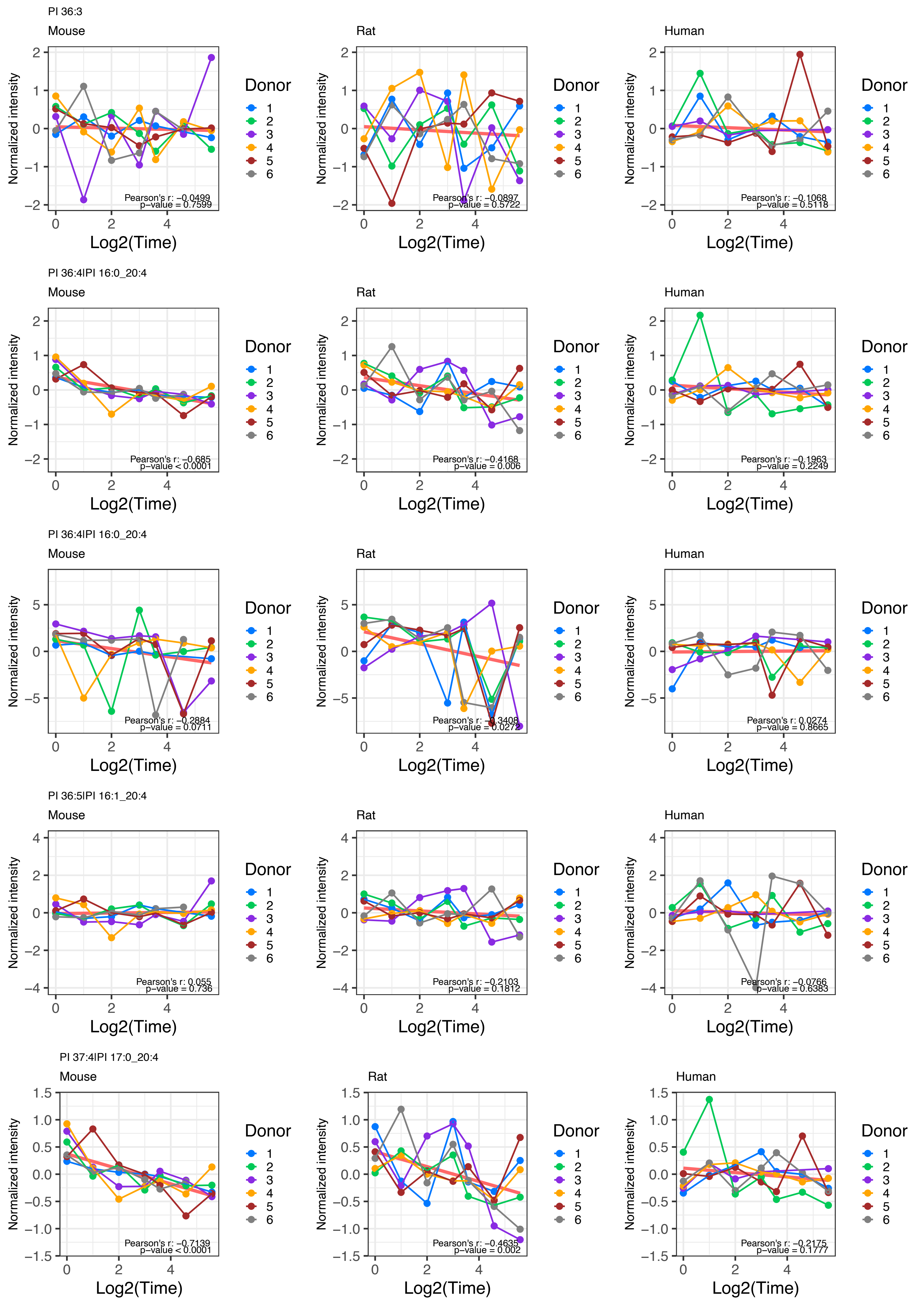
PI 36:3
Mouse
Pearson's r: −0.0499
p−value = 0.7599
Rat
Pearson's r: −0.0897
p−value = 0.5722
Human
Pearson's r: −0.1068
p−value = 0.5118
PI 36:4|PI 16:0_20:4
Mouse
Pearson's r: −0.685
p−value < 0.0001
Rat
Pearson's r: −0.4168
p−value = 0.006
Human
Pearson's r: −0.1963
p−value = 0.2249
PI 36:4|PI 16:0_20:4
Mouse
Pearson's r: −0.2884
p−value = 0.0711
Rat
Pearson's r: −0.3408
p−value = 0.0272
Human
Pearson's r: 0.0274
p−value = 0.8665
PI 36:5|PI 16:1_20:4
Mouse
Pearson's r: 0.055
p−value = 0.736
Rat
Pearson's r: −0.2103
p−value = 0.1812
Human
Pearson's r: −0.0766
p−value = 0.6383
PI 37:4|PI 17:0_20:4
Mouse
Pearson's r: −0.7139
p−value < 0.0001
Rat
Pearson's r: −0.4635
p−value = 0.002
Human
Pearson's r: −0.2175
p−value = 0.1777
Normalized intensity
Log2(Time)
Donor
1
2
3
4
5
6

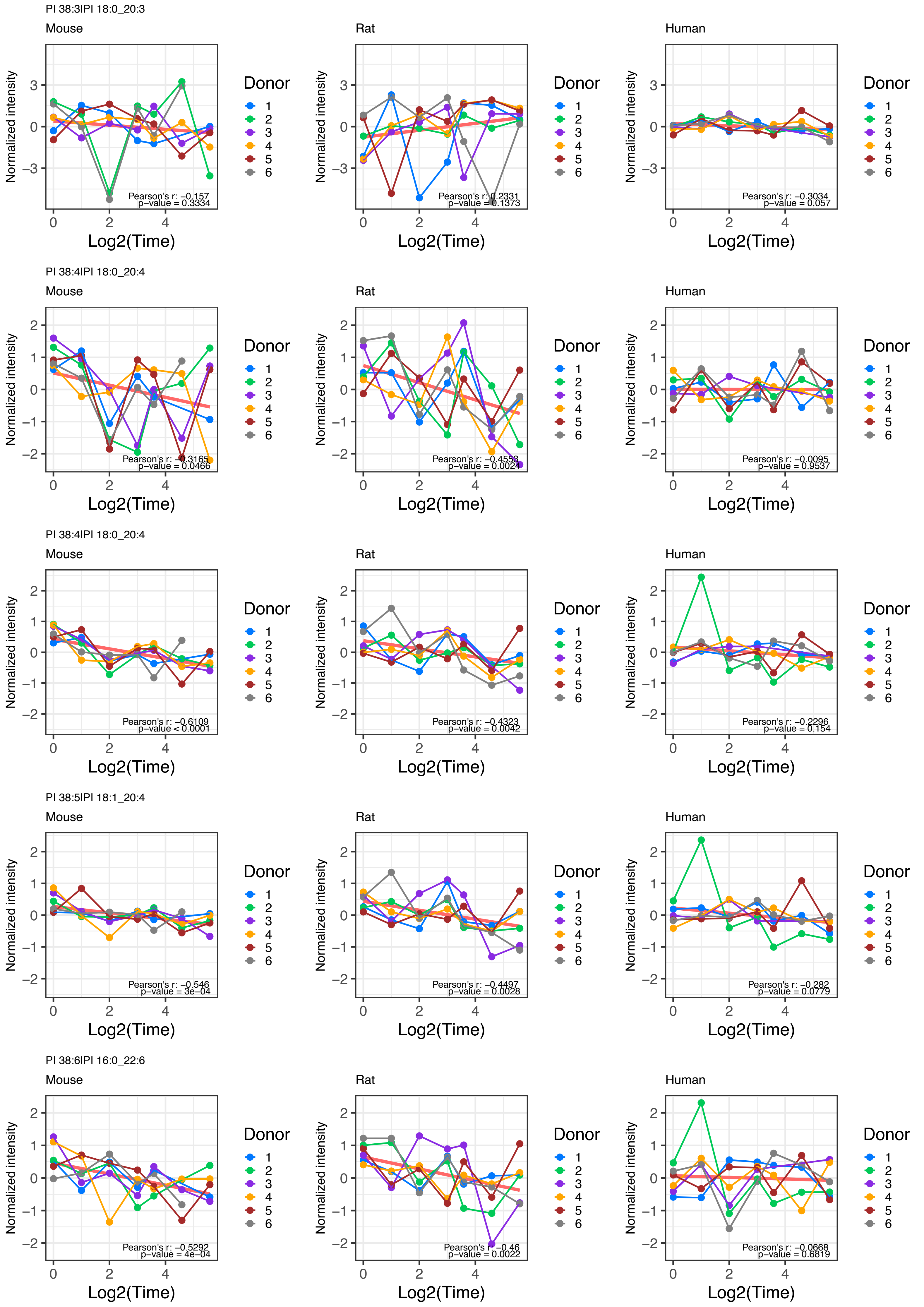
PI 38:3|PI 18:0_20:3
Mouse
Pearson's r: −0.157
p−value = 0.3334
Rat
Pearson's r: 0.2331
p−value = 0.1373
Human
Pearson's r: −0.3034
p−value = 0.057
PI 38:4|PI 18:0_20:4
Mouse
Pearson's r: −0.3165
p−value = 0.0466
Rat
Pearson's r: −0.4553
p−value = 0.0024
Human
Pearson's r: −0.0095
p−value = 0.9537
PI 38:4|PI 18:0_20:4
Mouse
Pearson's r: −0.6109
p−value < 0.0001
Rat
Pearson's r: −0.4323
p−value = 0.0042
Human
Pearson's r: −0.2296
p−value = 0.154
PI 38:5|PI 18:1_20:4
Mouse
Pearson's r: −0.546
p−value = 3e−04
Rat
Pearson's r: −0.4497
p−value = 0.0028
Human
Pearson's r: −0.282
p−value = 0.0779
PI 38:6|PI 16:0_22:6
Mouse
Pearson's r: −0.5292
p−value = 4e−04
Rat
Pearson's r: −0.46
p−value = 0.0022
Human
Pearson's r: −0.0668
p−value = 0.6819
Normalized intensity
Log2(Time)
Donor
1
2
3
4
5
6

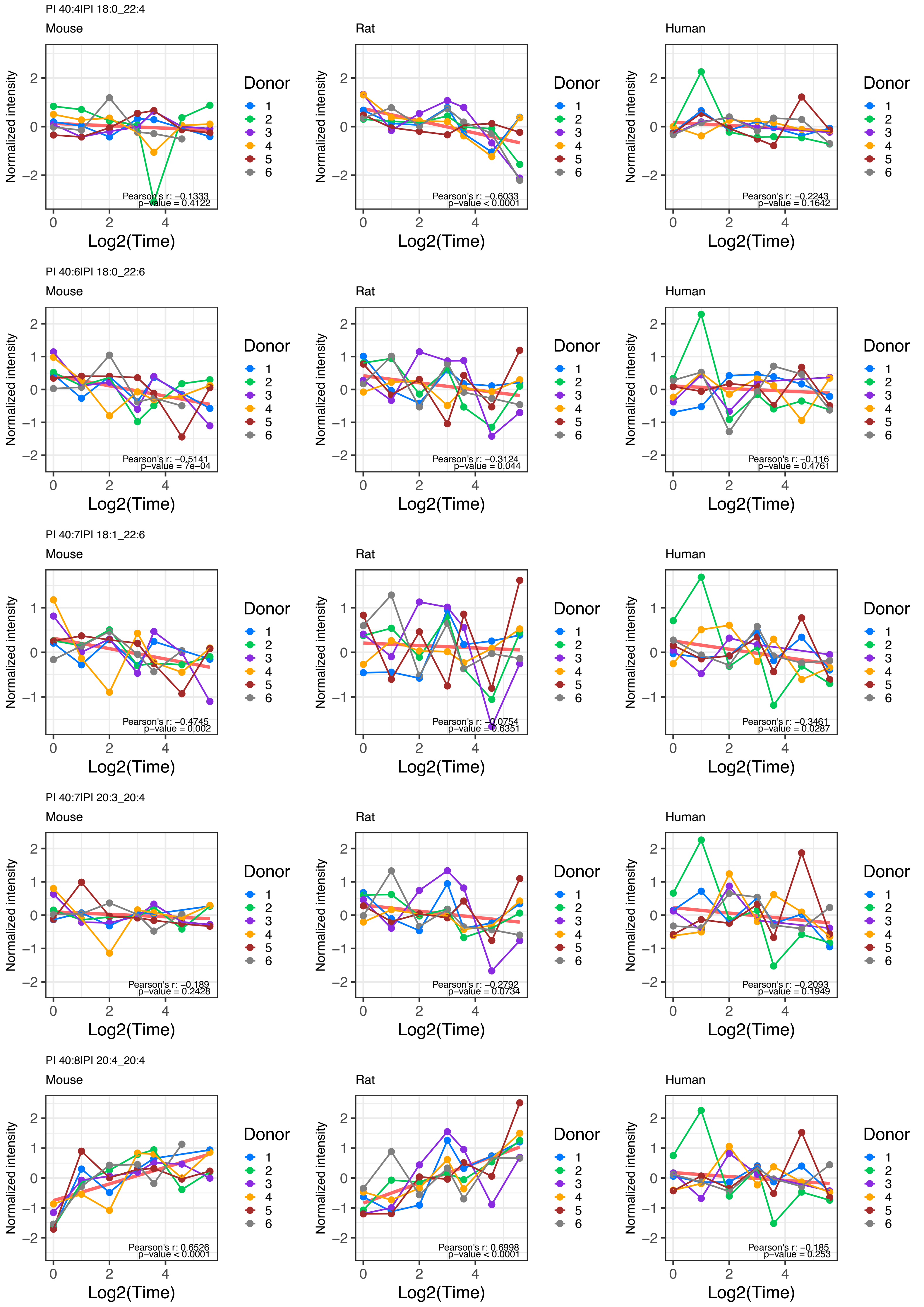
PI 40:4|PI 18:0_22:4
Mouse
Pearson's r: −0.1333
p−value = 0.4122
Rat
Pearson's r: −0.6033
p−value < 0.0001
Human
Pearson's r: −0.2243
p−value = 0.1642
PI 40:6|PI 18:0_22:6
Mouse
Pearson's r: −0.5141
p−value = 7e−04
Rat
Pearson's r: −0.3124
p−value = 0.044
Human
Pearson's r: −0.116
p−value = 0.4761
PI 40:7|PI 18:1_22:6
Mouse
Pearson's r: −0.4745
p−value = 0.002
Rat
Pearson's r: −0.0754
p−value = 0.6351
Human
Pearson's r: −0.3461
p−value = 0.0287
PI 40:7|PI 20:3_20:4
Mouse
Pearson's r: −0.189
p−value = 0.2428
Rat
Pearson's r: −0.2792
p−value = 0.0734
Human
Pearson's r: −0.2093
p−value = 0.1949
PI 40:8|PI 20:4_20:4
Mouse
Pearson's r: 0.6526
p−value < 0.0001
Rat
Pearson's r: 0.6998
p−value < 0.0001
Human
Pearson's r: −0.185
p−value = 0.253
Normalized intensity
Log2(Time)
Donor
1
2
3
4
5
6

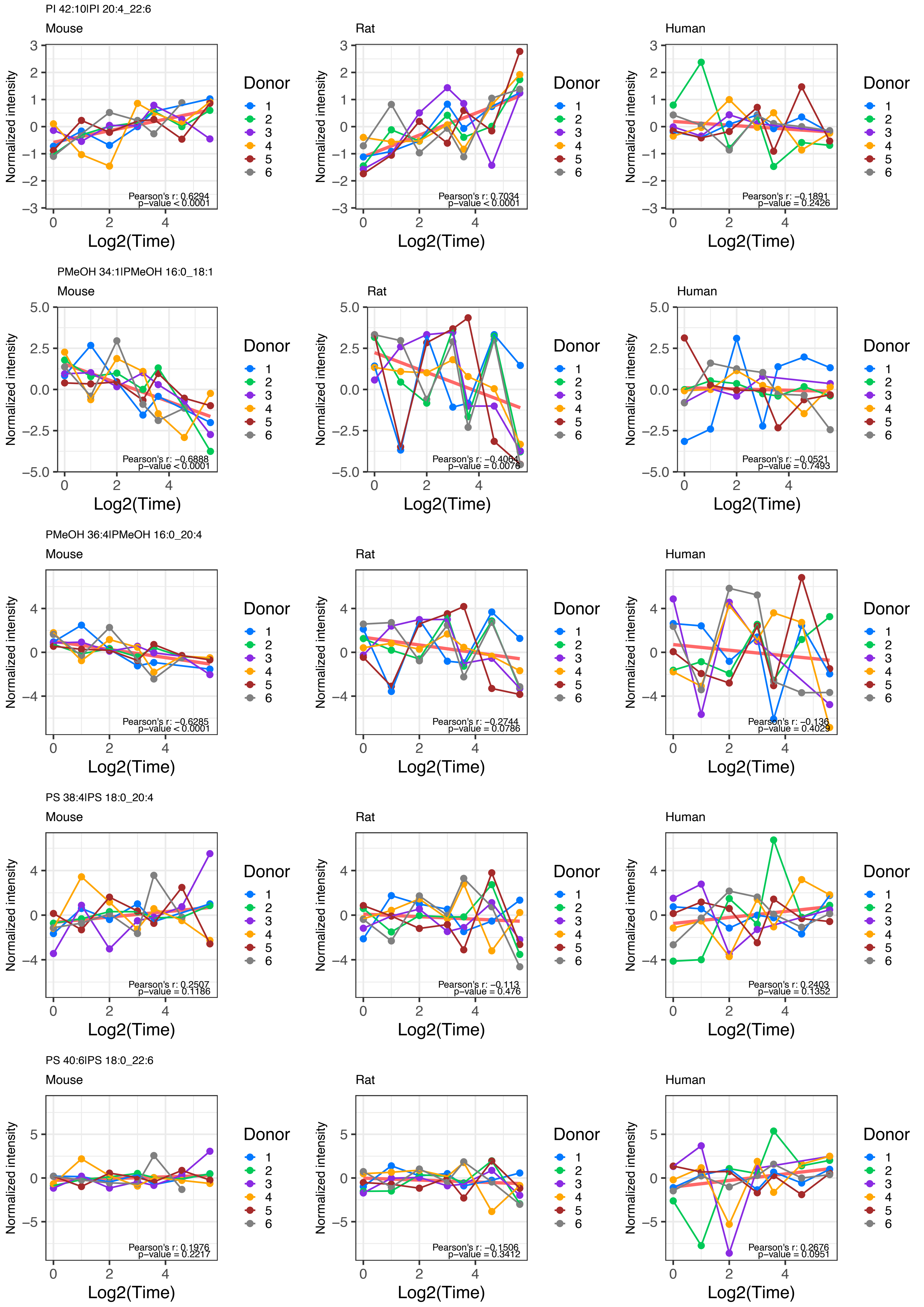
PI 42:10|PI 20:4_22:6
Mouse
Rat
Human
Normalized intensity
Log2(Time)
Donor
1
2
3
4
5
6
Pearson's r: 0.6294
p−value < 0.0001
Pearson's r: 0.7034
p−value < 0.0001
Pearson's r: −0.1891
p−value = 0.2426
PMeOH 34:1|PMeOH 16:0_18:1
Pearson's r: −0.6888
p−value < 0.0001
Pearson's r: −0.4064
p−value = 0.0076
Pearson's r: −0.0521
p−value = 0.7493
PMeOH 36:4|PMeOH 16:0_20:4
Pearson's r: −0.6285
p−value < 0.0001
Pearson's r: −0.2744
p−value = 0.0786
Pearson's r: −0.136
p−value = 0.4029
PS 38:4|PS 18:0_20:4
Pearson's r: 0.2507
p−value = 0.1186
Pearson's r: −0.113
p−value = 0.476
Pearson's r: 0.2403
p−value = 0.1352
PS 40:6|PS 18:0_22:6
Pearson's r: 0.1976
p−value = 0.2217
Pearson's r: −0.1506
p−value = 0.3412
Pearson's r: 0.2676
p−value = 0.0951

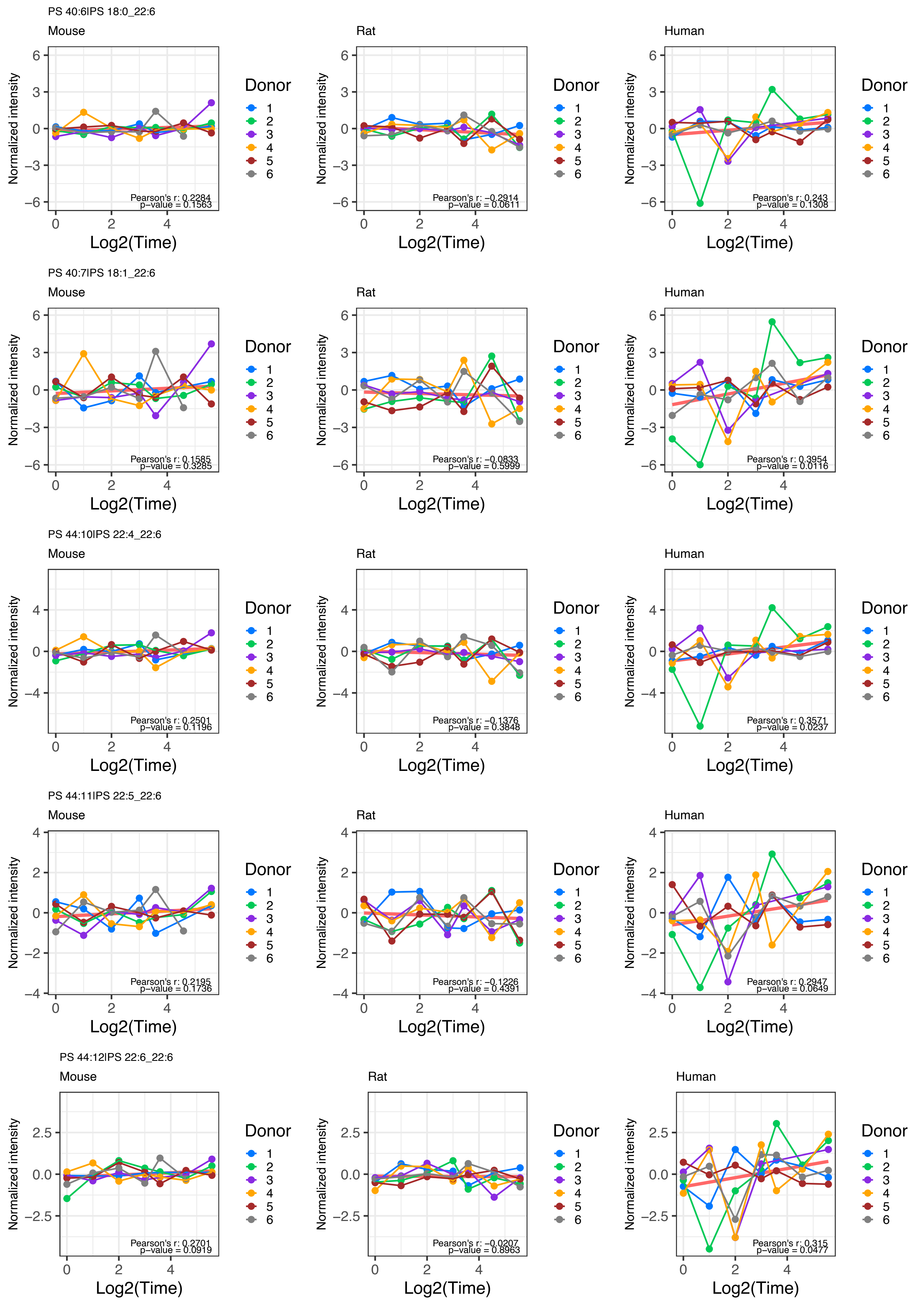

PS 40:6|PS 18:0_22:6
Mouse
Pearson's r: 0.2284
p−value = 0.1563
Rat
Pearson's r: −0.2914
p−value = 0.0611
Human
Pearson's r: 0.243
p−value = 0.1308
PS 40:7|PS 18:1_22:6
Mouse
Pearson's r: 0.1585
p−value = 0.3285
Rat
Pearson's r: −0.0833
p−value = 0.5999
Human
Pearson's r: 0.3954
p−value = 0.0116
PS 44:10|PS 22:4_22:6
Mouse
Pearson's r: 0.2501
p−value = 0.1196
Rat
Pearson's r: −0.1376
p−value = 0.3848
Human
Pearson's r: 0.3571
p−value = 0.0237
PS 44:11|PS 22:5_22:6
Mouse
Pearson's r: 0.2195
p−value = 0.1736
Rat
Pearson's r: −0.1226
p−value = 0.4391
Human
Pearson's r: 0.2947
p−value = 0.0649
PS 44:12|PS 22:6_22:6
Mouse
Pearson's r: 0.2701
p−value = 0.0919
Rat
Pearson's r: −0.0207
p−value = 0.8963
Human
Pearson's r: 0.315
p−value = 0.0477
Normalized intensity
Log2(Time)
Donor
1
2
3
4
5
6

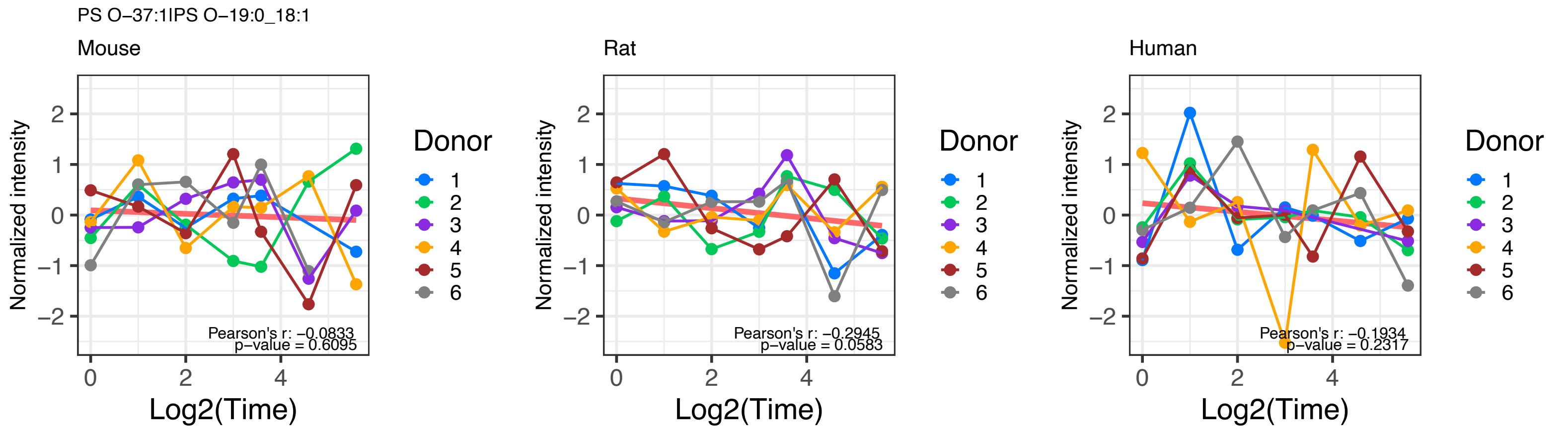
PS O−37:1|PS O−19:0_18:1
Mouse
Rat
Human
Normalized intensity
Log2(Time)
Donor
1
2
3
4
5
6
Pearson's r: −0.0833
p−value = 0.6095
Pearson's r: −0.2945
p−value = 0.0583
Pearson's r: −0.1934
p−value = 0.2317

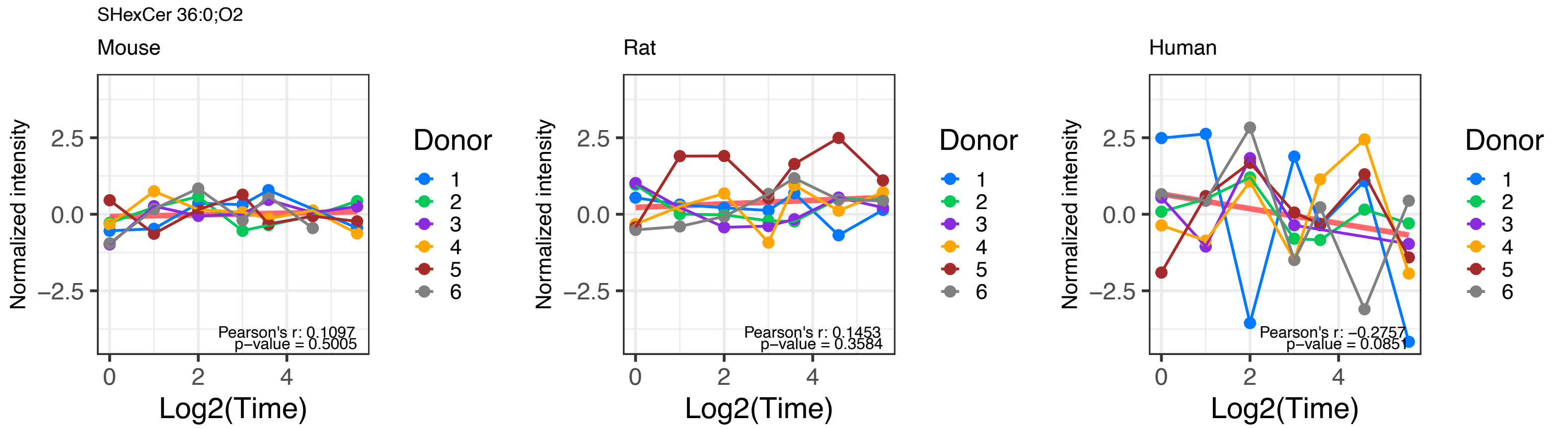
SHexCer 36:0;O2
Mouse
Rat
Human
Normalized intensity
Log2(Time)
Donor
1
2
3
4
5
6
Pearson's r: 0.1097
p−value = 0.5005
Pearson's r: 0.1453
p−value = 0.3584
Pearson's r: −0.2757
p−value = 0.0851

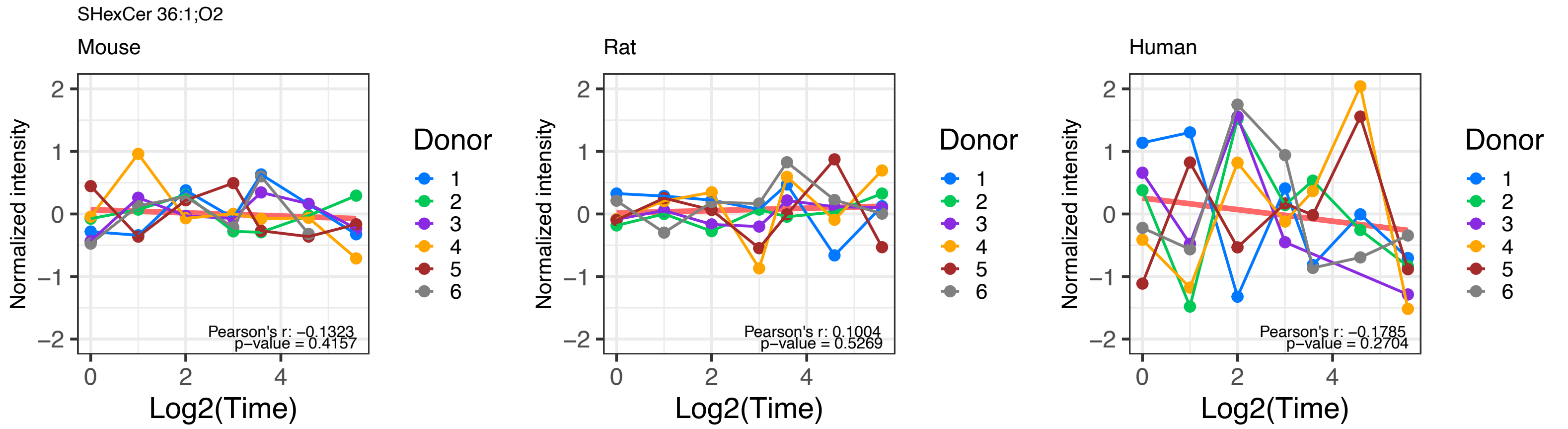
SHexCer 36:1;O2
Mouse
Rat
Human
Normalized intensity
Log2(Time)
Donor
1
2
3
4
5
6
Pearson's r: −0.1323
p−value = 0.4157
Pearson's r: 0.1004
p−value = 0.5269
Pearson's r: −0.1785
p−value = 0.2704

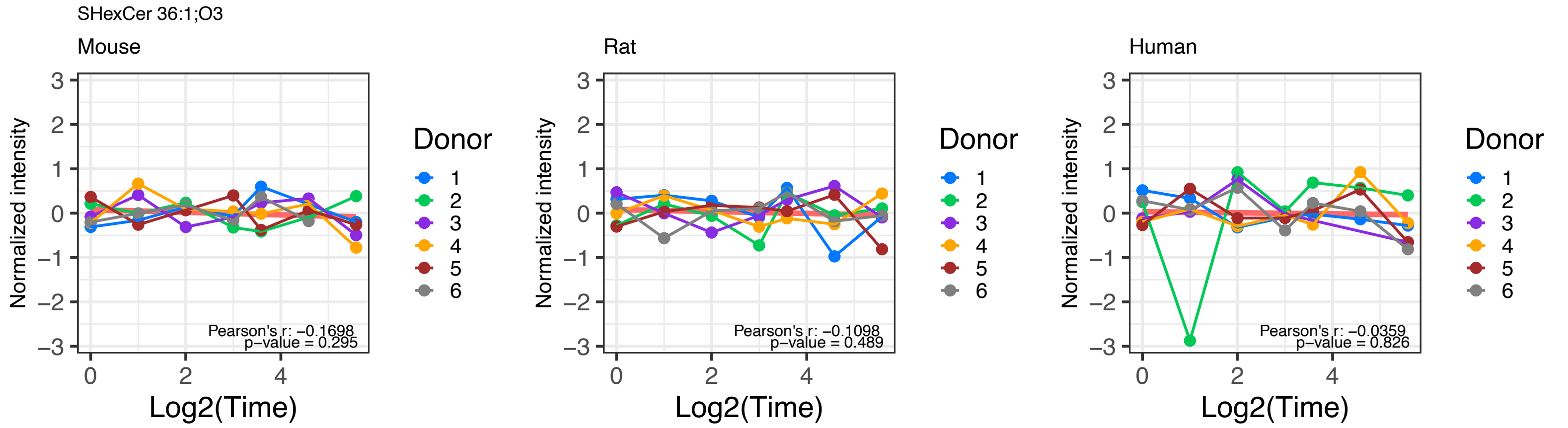
SHexCer 36:1;O3
Mouse
Rat
Human
Normalized intensity
Log2(Time)
Donor
1
2
3
4
5
6
Pearson's r: −0.1698
p−value = 0.295
Pearson's r: −0.1098
p−value = 0.489
Pearson's r: −0.0359
p−value = 0.826

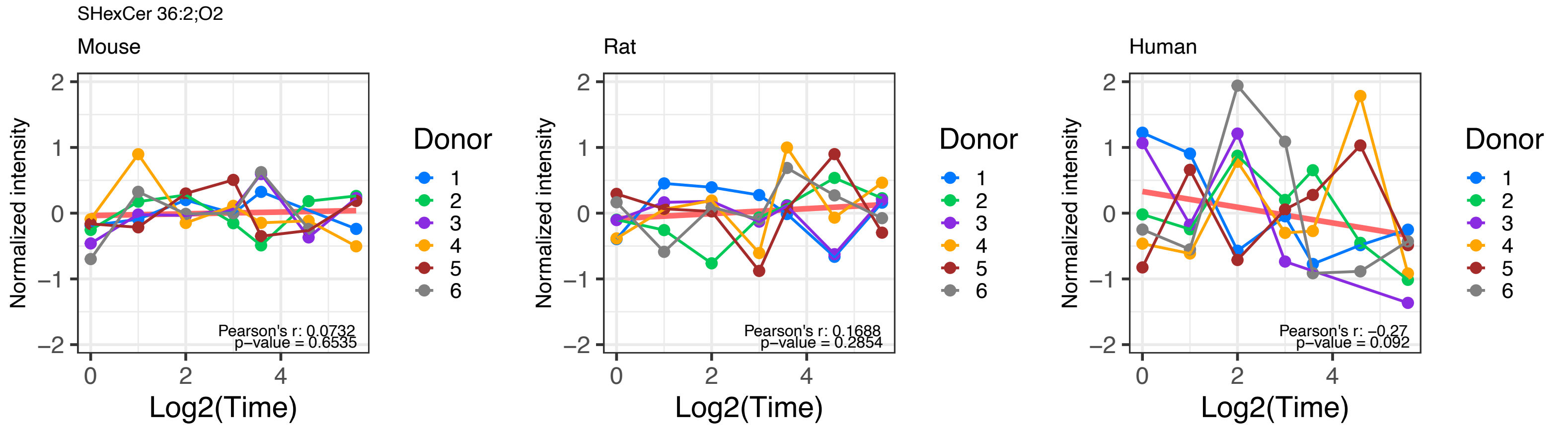
SHexCer 36:2;O2
Mouse
Rat
Human
Normalized intensity
Log2(Time)
Donor
1
2
3
4
5
6
Pearson's r: 0.0732
p−value = 0.6535
Pearson's r: 0.1688
p−value = 0.2854
Pearson's r: −0.27
p−value = 0.092

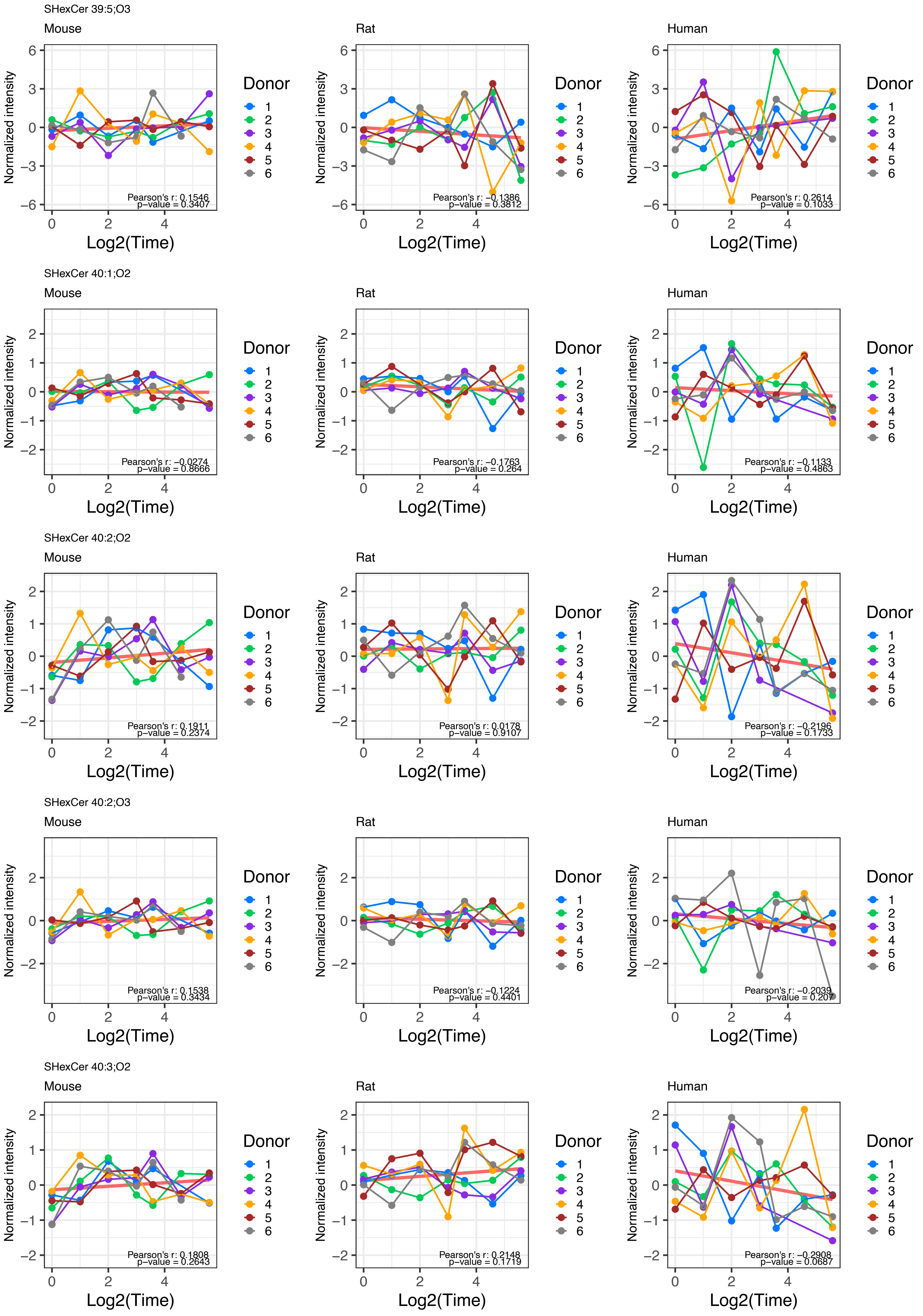
SHexCer 39:5;O3
Mouse
Pearson's r: 0.1546
p−value = 0.3407
Rat
Pearson's r: −0.1386
p−value = 0.3812
Human
Pearson's r: 0.2614
p−value = 0.1033
SHexCer 40:1;O2
Mouse
Pearson's r: −0.0274
p−value = 0.8666
Rat
Pearson's r: −0.1763
p−value = 0.264
Human
Pearson's r: −0.1133
p−value = 0.4863
SHexCer 40:2;O2
Mouse
Pearson's r: 0.1911
p−value = 0.2374
Rat
Pearson's r: 0.0178
p−value = 0.9107
Human
Pearson's r: −0.2196
p−value = 0.1733
SHexCer 40:2;O3
Mouse
Pearson's r: 0.1538
p−value = 0.3434
Rat
Pearson's r: −0.1224
p−value = 0.4401
Human
Pearson's r: −0.2039
p−value = 0.207
SHexCer 40:3;O2
Mouse
Pearson's r: 0.1808
p−value = 0.2643
Rat
Pearson's r: 0.2148
p−value = 0.1719
Human
Pearson's r: −0.2908
p−value = 0.0687
Normalized intensity
Log2(Time)
Donor
1
2
3
4
5
6

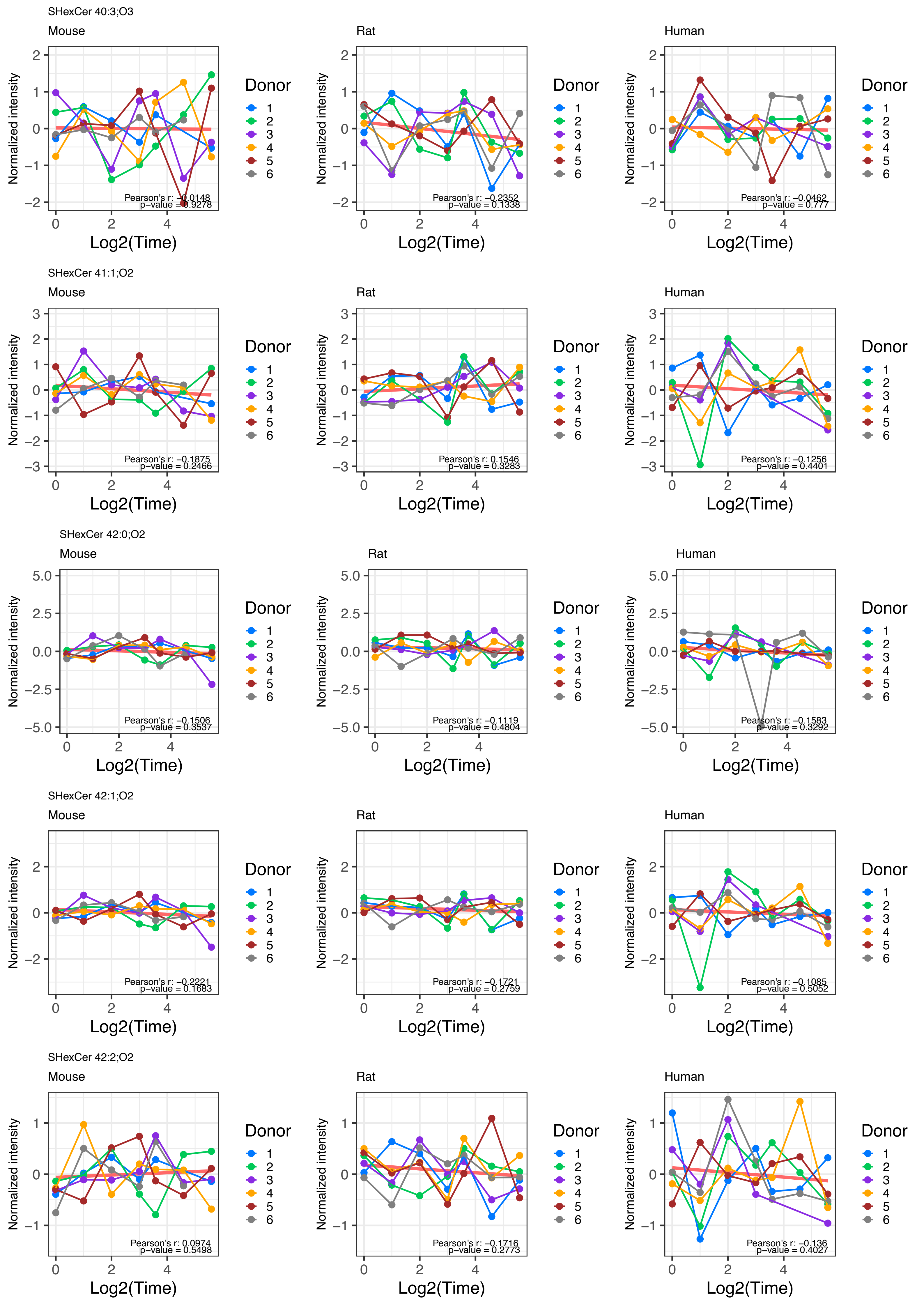

SHexCer 40:3;O3
Mouse
Pearson's r: −0.0148
p−value = 0.9278
Rat
Pearson's r: −0.2352
p−value = 0.1338
Human
Pearson's r: −0.0462
p−value = 0.777
SHexCer 41:1;O2
Mouse
Pearson's r: −0.1875
p−value = 0.2466
Rat
Pearson's r: 0.1546
p−value = 0.3283
Human
Pearson's r: −0.1256
p−value = 0.4401
SHexCer 42:0;O2
Mouse
Pearson's r: −0.1506
p−value = 0.3537
Rat
Pearson's r: −0.1119
p−value = 0.4804
Human
Pearson's r: −0.1583
p−value = 0.3292
SHexCer 42:1;O2
Mouse
Pearson's r: −0.2221
p−value = 0.1683
Rat
Pearson's r: −0.1721
p−value = 0.2759
Human
Pearson's r: −0.1085
p−value = 0.5052
SHexCer 42:2;O2
Mouse
Pearson's r: 0.0974
p−value = 0.5498
Rat
Pearson's r: −0.1716
p−value = 0.2773
Human
Pearson's r: −0.136
p−value = 0.4027
Normalized intensity
Log2(Time)
Donor
1
2
3
4
5
6

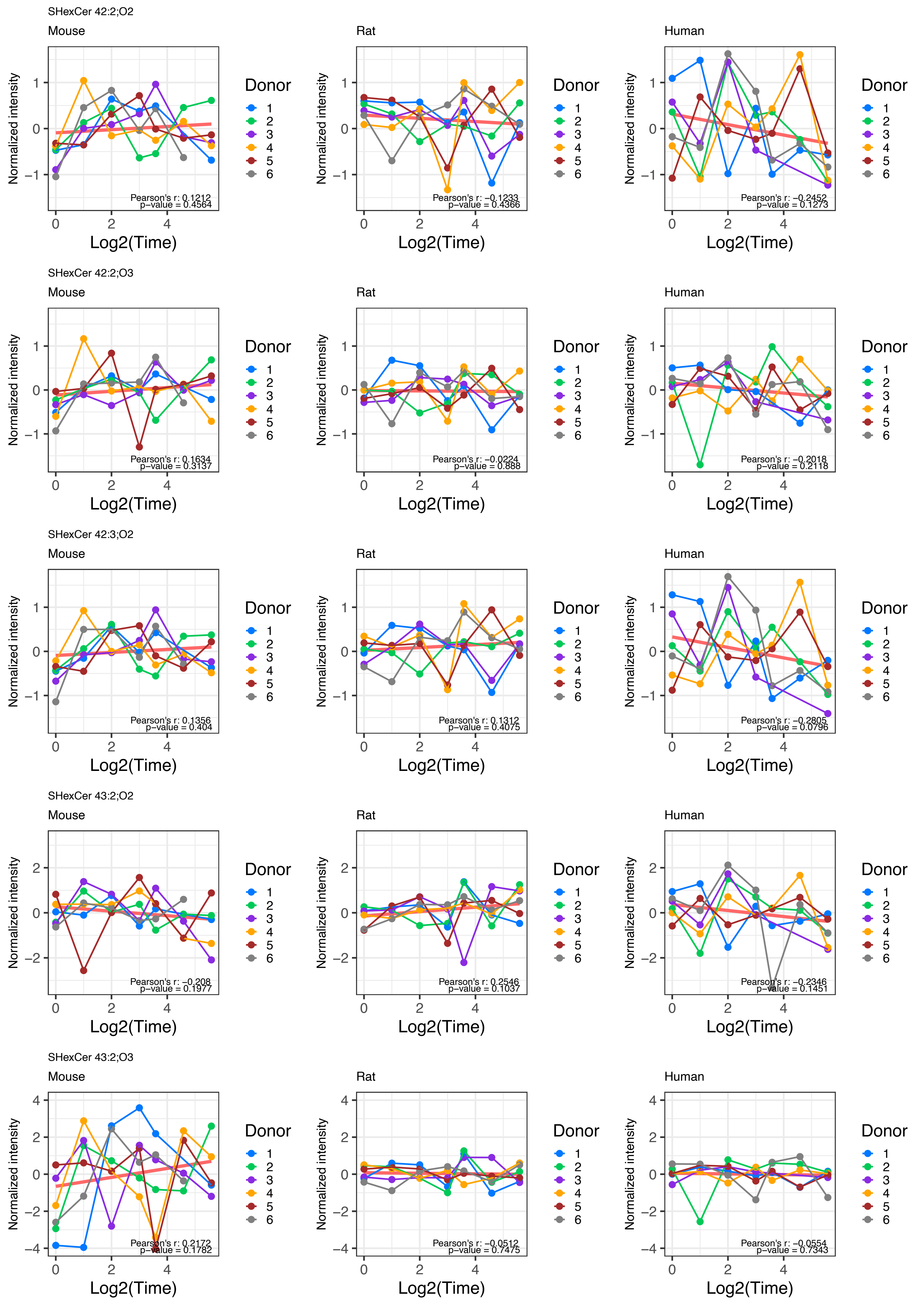
SHexCer 42:2;O2
Mouse
Pearson's r: 0.1212
p−value = 0.4564
Rat
Pearson's r: −0.1233
p−value = 0.4366
Human
Pearson's r: −0.2452
p−value = 0.1273
SHexCer 42:2;O3
Mouse
Pearson's r: 0.1634
p−value = 0.3137
Rat
Pearson's r: −0.0224
p−value = 0.888
Human
Pearson's r: −0.2018
p−value = 0.2118
SHexCer 42:3;O2
Mouse
Pearson's r: 0.1356
p−value = 0.404
Rat
Pearson's r: 0.1312
p−value = 0.4075
Human
Pearson's r: −0.2805
p−value = 0.0796
SHexCer 43:2;O2
Mouse
Pearson's r: −0.208
p−value = 0.1977
Rat
Pearson's r: 0.2546
p−value = 0.1037
Human
Pearson's r: −0.2346
p−value = 0.1451
SHexCer 43:2;O3
Mouse
Pearson's r: 0.2172
p−value = 0.1782
Rat
Pearson's r: −0.0512
p−value = 0.7475
Human
Pearson's r: −0.0554
p−value = 0.7343
Normalized intensity
Log2(Time)
Donor
1
2
3
4
5
6

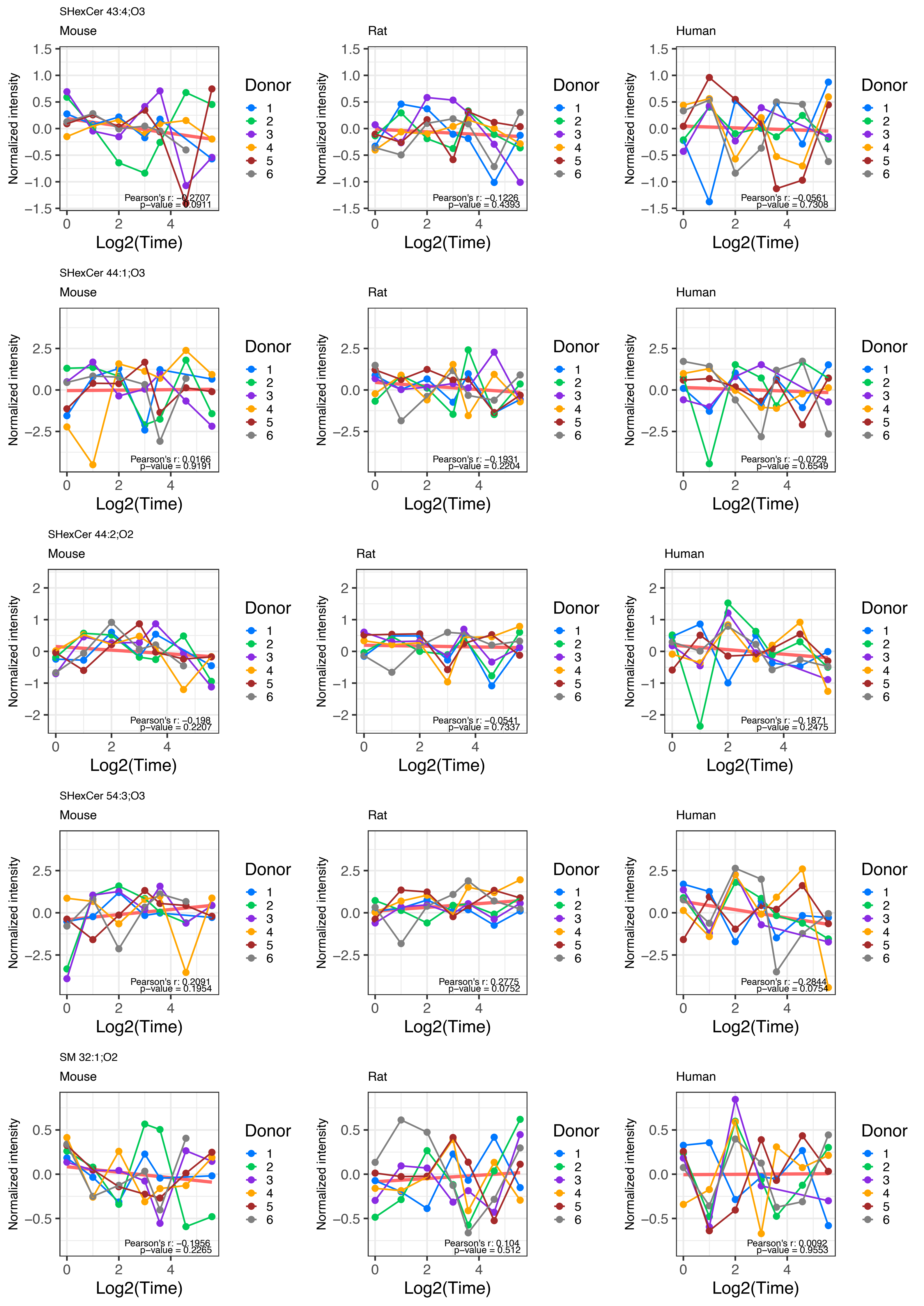
SHexCer 43:4;O3
Mouse
Pearson's r: −0.2707
p−value = 0.0911
Rat
Pearson's r: −0.1226
p−value = 0.4393
Human
Pearson's r: −0.0561
p−value = 0.7308
SHexCer 44:1;O3
Mouse
Pearson's r: 0.0166
p−value = 0.9191
Rat
Pearson's r: −0.1931
p−value = 0.2204
Human
Pearson's r: −0.0729
p−value = 0.6549
SHexCer 44:2;O2
Mouse
Pearson's r: −0.198
p−value = 0.2207
Rat
Pearson's r: −0.0541
p−value = 0.7337
Human
Pearson's r: −0.1871
p−value = 0.2475
SHexCer 54:3;O3
Mouse
Pearson's r: 0.2091
p−value = 0.1954
Rat
Pearson's r: 0.2775
p−value = 0.0752
Human
Pearson's r: −0.2844
p−value = 0.0754
SM 32:1;O2
Mouse
Pearson's r: −0.1956
p−value = 0.2265
Rat
Pearson's r: 0.104
p−value = 0.512
Human
Pearson's r: 0.0092
p−value = 0.9553
Normalized intensity
Log2(Time)
Donor
1
2
3
4
5
6

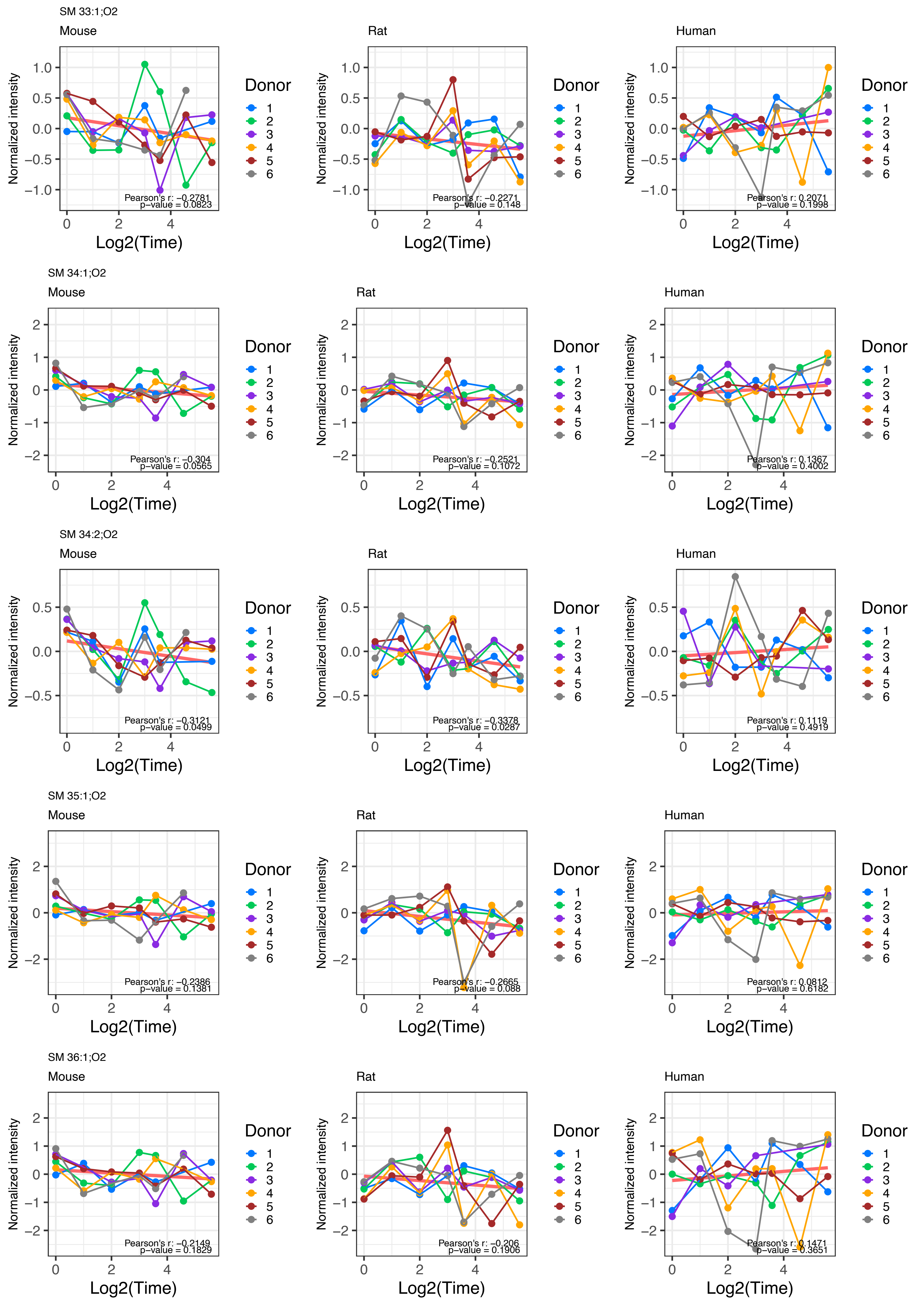
SM 33:1;O2
Mouse
Pearson's r: −0.2781
p−value = 0.0823
Rat
Pearson's r: −0.2271
p−value = 0.148
Human
Pearson's r: 0.2071
p−value = 0.1998
SM 34:1;O2
Mouse
Pearson's r: −0.304
p−value = 0.0565
Rat
Pearson's r: −0.2521
p−value = 0.1072
Human
Pearson's r: 0.1367
p−value = 0.4002
SM 34:2;O2
Mouse
Pearson's r: −0.3121
p−value = 0.0499
Rat
Pearson's r: −0.3378
p−value = 0.0287
Human
Pearson's r: 0.1119
p−value = 0.4919
SM 35:1;O2
Mouse
Pearson's r: −0.2386
p−value = 0.1381
Rat
Pearson's r: −0.2665
p−value = 0.088
Human
Pearson's r: 0.0812
p−value = 0.6182
SM 36:1;O2
Mouse
Pearson's r: −0.2149
p−value = 0.1829
Rat
Pearson's r: −0.206
p−value = 0.1906
Human
Pearson's r: 0.1471
p−value = 0.3651
Normalized intensity
Log2(Time)
Donor
1
2
3
4
5
6

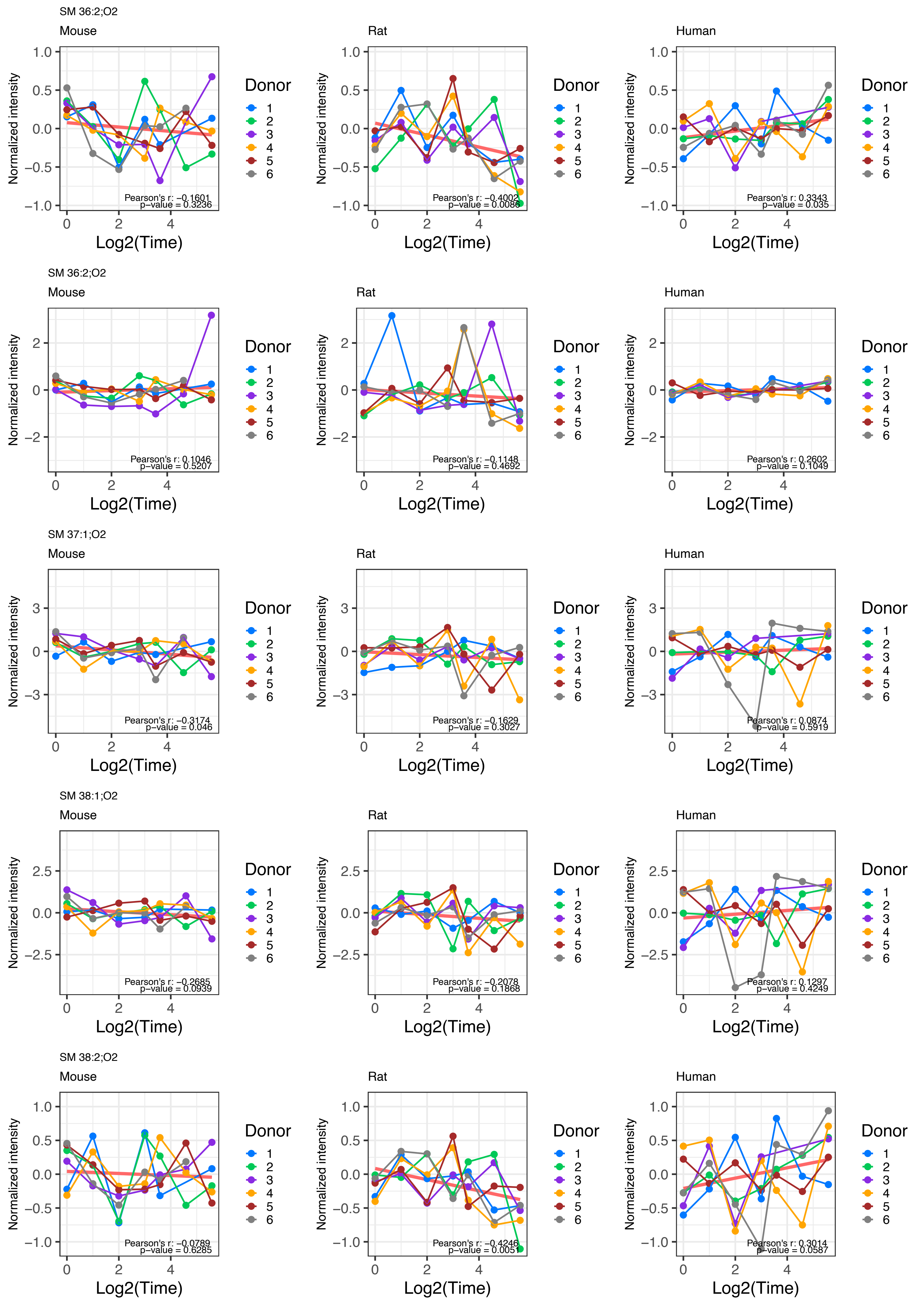

SM 36:2;O2
Mouse
Pearson's r: −0.1601
p−value = 0.3236
Rat
Pearson's r: −0.4002
p−value = 0.0086
Human
Pearson's r: 0.3343
p−value = 0.035
SM 36:2;O2
Mouse
Pearson's r: 0.1046
p−value = 0.5207
Rat
Pearson's r: −0.1148
p−value = 0.4692
Human
Pearson's r: 0.2602
p−value = 0.1049
SM 37:1;O2
Mouse
Pearson's r: −0.3174
p−value = 0.046
Rat
Pearson's r: −0.1629
p−value = 0.3027
Human
Pearson's r: 0.0874
p−value = 0.5919
SM 38:1;O2
Mouse
Pearson's r: −0.2685
p−value = 0.0939
Rat
Pearson's r: −0.2078
p−value = 0.1868
Human
Pearson's r: 0.1297
p−value = 0.4249
SM 38:2;O2
Mouse
Pearson's r: −0.0789
p−value = 0.6285
Rat
Pearson's r: −0.4246
p−value = 0.0051
Human
Pearson's r: 0.3014
p−value = 0.0587
Normalized intensity
Log2(Time)
Donor
1
2
3
4
5
6

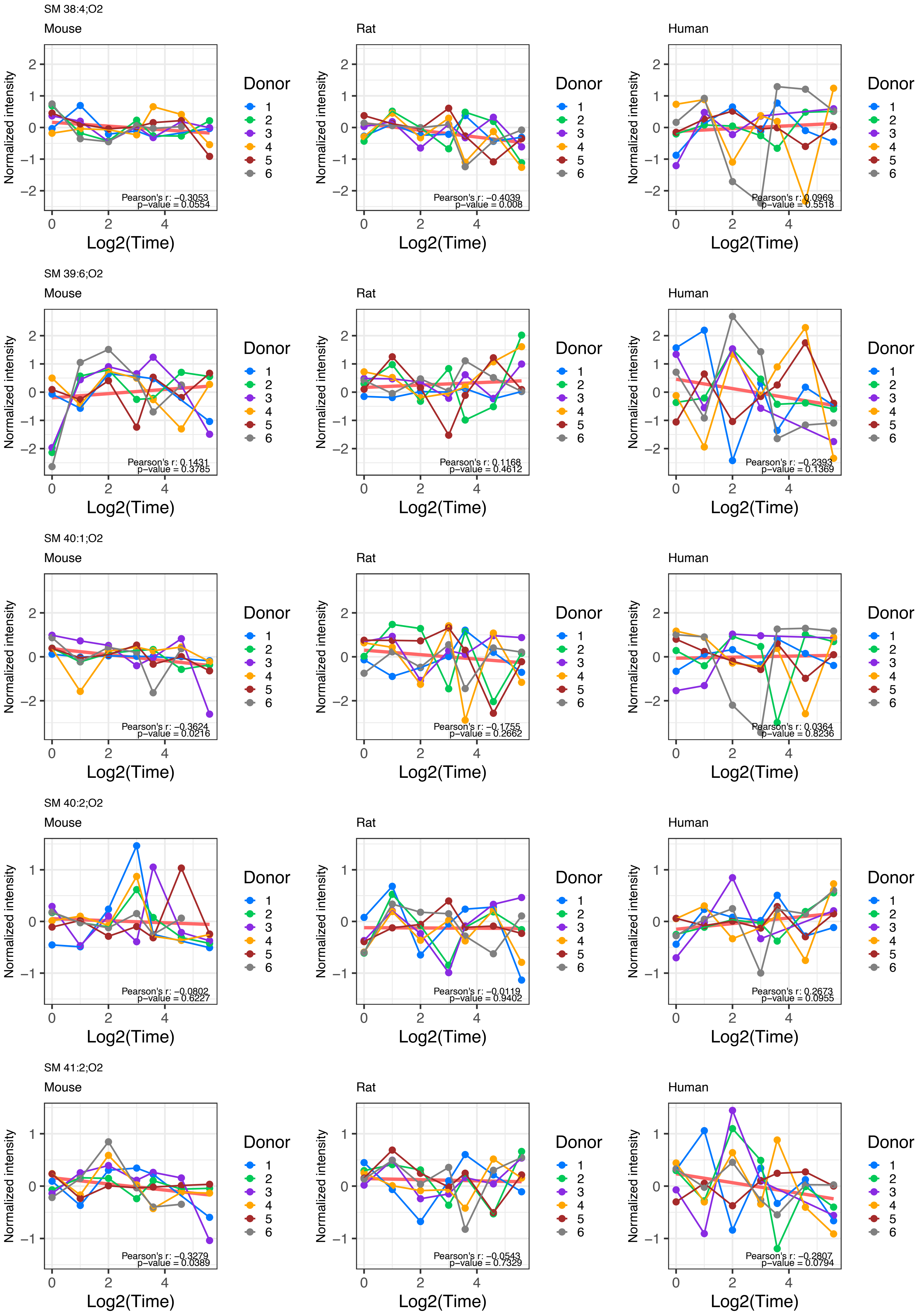

SM 38:4;O2
Mouse
Pearson's r: −0.3053
p−value = 0.0554
Rat
Pearson's r: −0.4039
p−value = 0.008
Human
Pearson's r: 0.0969
p−value = 0.5518
SM 39:6;O2
Mouse
Pearson's r: 0.1431
p−value = 0.3785
Rat
Pearson's r: 0.1168
p−value = 0.4612
Human
Pearson's r: −0.2393
p−value = 0.1369
SM 40:1;O2
Mouse
Pearson's r: −0.3624
p−value = 0.0216
Rat
Pearson's r: −0.1755
p−value = 0.2662
Human
Pearson's r: 0.0364
p−value = 0.8236
SM 40:2;O2
Mouse
Pearson's r: −0.0802
p−value = 0.6227
Rat
Pearson's r: −0.0119
p−value = 0.9402
Human
Pearson's r: 0.2673
p−value = 0.0955
SM 41:2;O2
Mouse
Pearson's r: −0.3279
p−value = 0.0389
Rat
Pearson's r: −0.0543
p−value = 0.7329
Human
Pearson's r: −0.2807
p−value = 0.0794
Normalized intensity
Log2(Time)
Donor
1
2
3
4
5
6

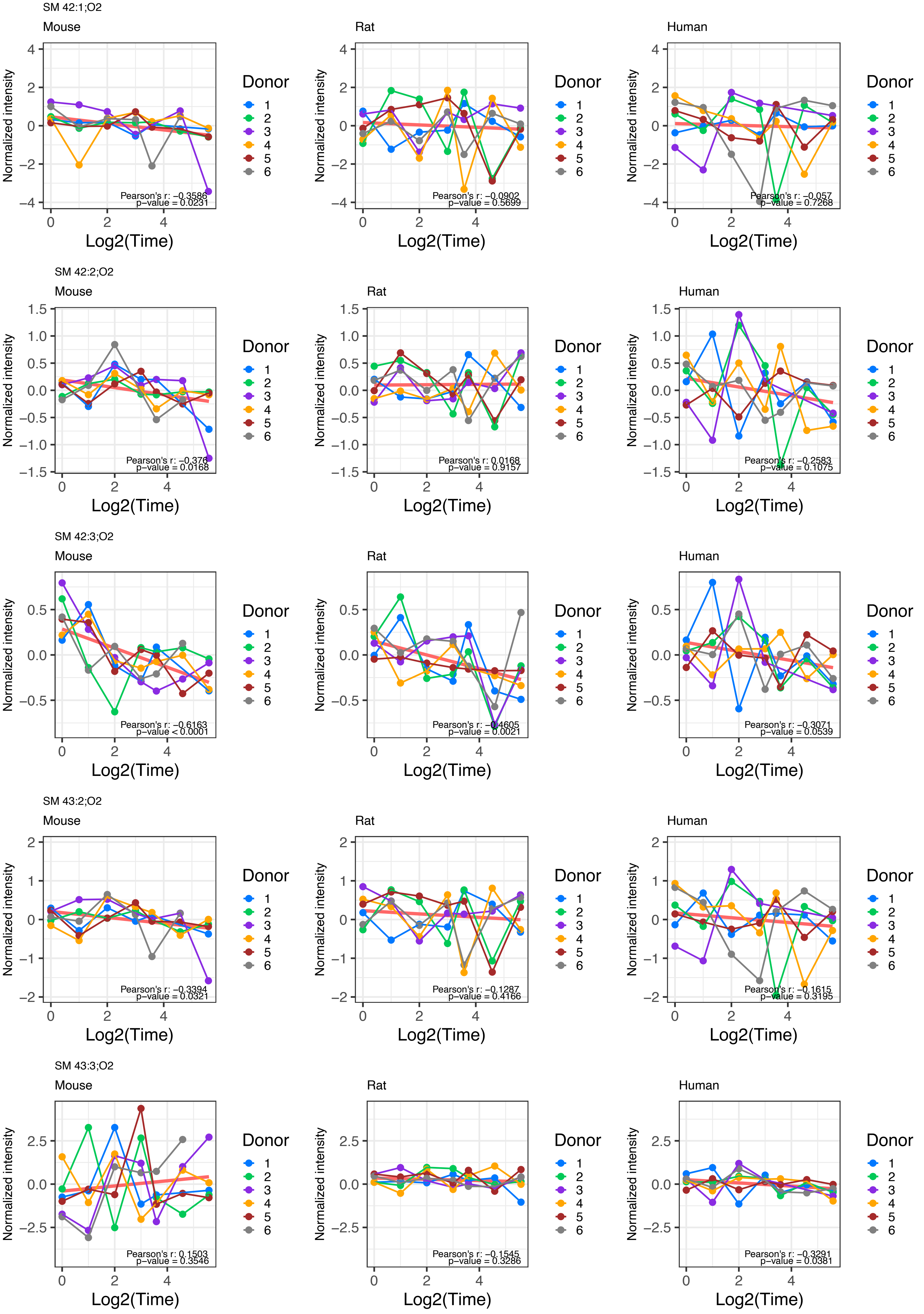
SM 42:1;O2
Mouse
Pearson's r: −0.3586
p−value = 0.0231
Rat
Pearson's r: −0.0902
p−value = 0.5699
Human
Pearson's r: −0.057
p−value = 0.7268
SM 42:2;O2
Mouse
Pearson's r: −0.376
p−value = 0.0168
Rat
Pearson's r: 0.0168
p−value = 0.9157
Human
Pearson's r: −0.2583
p−value = 0.1075
SM 42:3;O2
Mouse
Pearson's r: −0.6163
p−value < 0.0001
Rat
Pearson's r: −0.4605
p−value = 0.0021
Human
Pearson's r: −0.3071
p−value = 0.0539
SM 43:2;O2
Mouse
Pearson's r: −0.3394
p−value = 0.0321
Rat
Pearson's r: −0.1287
p−value = 0.4166
Human
Pearson's r: −0.1615
p−value = 0.3195
SM 43:3;O2
Mouse
Pearson's r: 0.1503
p−value = 0.3546
Rat
Pearson's r: −0.1545
p−value = 0.3286
Human
Pearson's r: −0.3291
p−value = 0.0381
Normalized intensity
Log2(Time)
Donor
1
2
3
4
5
6

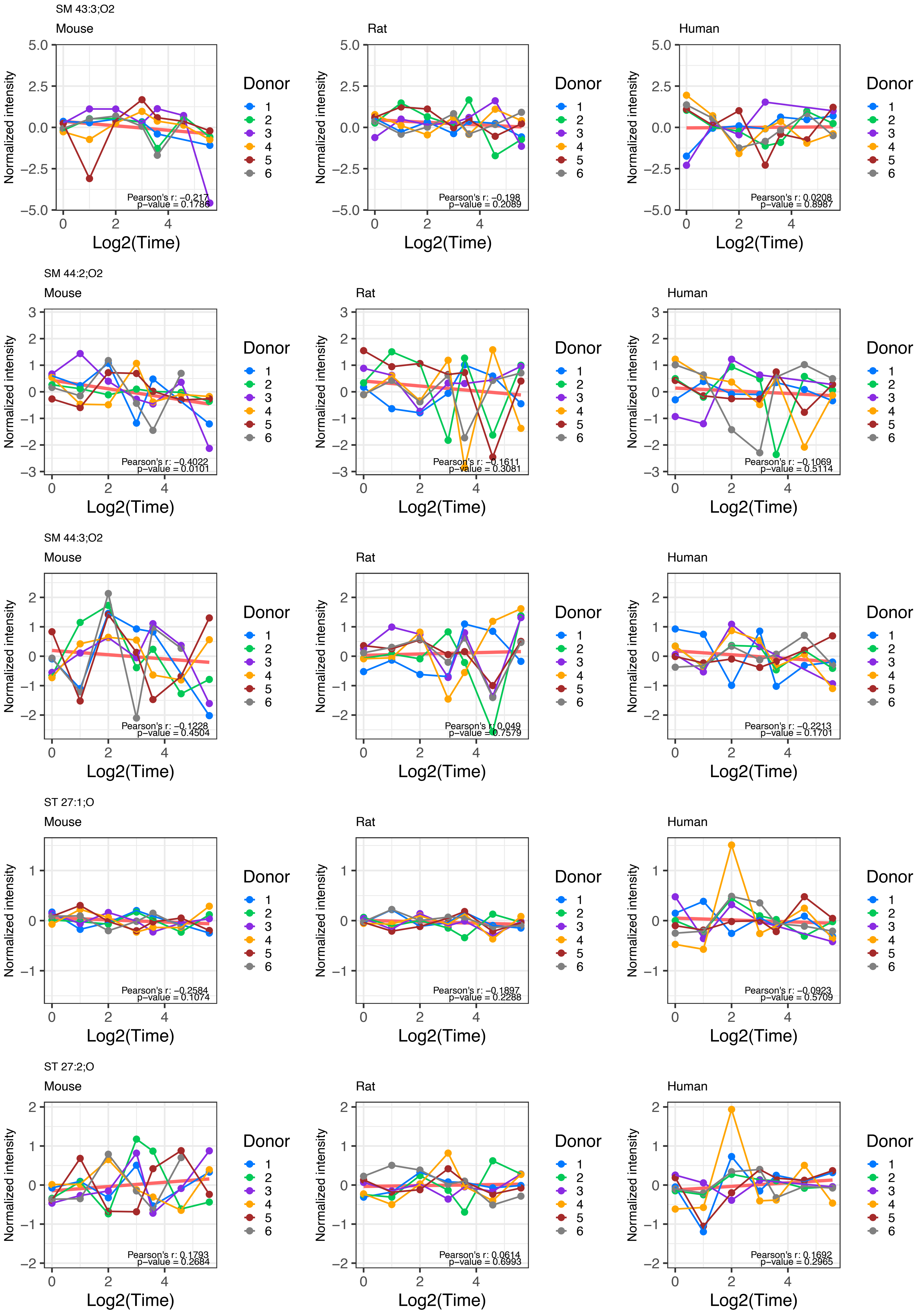
SM 43:3;O2
Mouse
Pearson's r: −0.217
p−value = 0.1786
Rat
Pearson's r: −0.198
p−value = 0.2089
Human
Pearson's r: 0.0208
p−value = 0.8987
SM 44:2;O2
Mouse
Pearson's r: −0.4022
p−value = 0.0101
Rat
Pearson's r: −0.1611
p−value = 0.3081
Human
Pearson's r: −0.1069
p−value = 0.5114
SM 44:3;O2
Mouse
Pearson's r: −0.1228
p−value = 0.4504
Rat
Pearson's r: 0.049
p−value = 0.7579
Human
Pearson's r: −0.2213
p−value = 0.1701
ST 27:1;O
Mouse
Pearson's r: −0.2584
p−value = 0.1074
Rat
Pearson's r: −0.1897
p−value = 0.2288
Human
Pearson's r: −0.0923
p−value = 0.5709
ST 27:2;O
Mouse
Pearson's r: 0.1793
p−value = 0.2684
Rat
Pearson's r: 0.0614
p−value = 0.6993
Human
Pearson's r: 0.1692
p−value = 0.2965
Normalized intensity
Log2(Time)
Donor
1
2
3
4
5
6

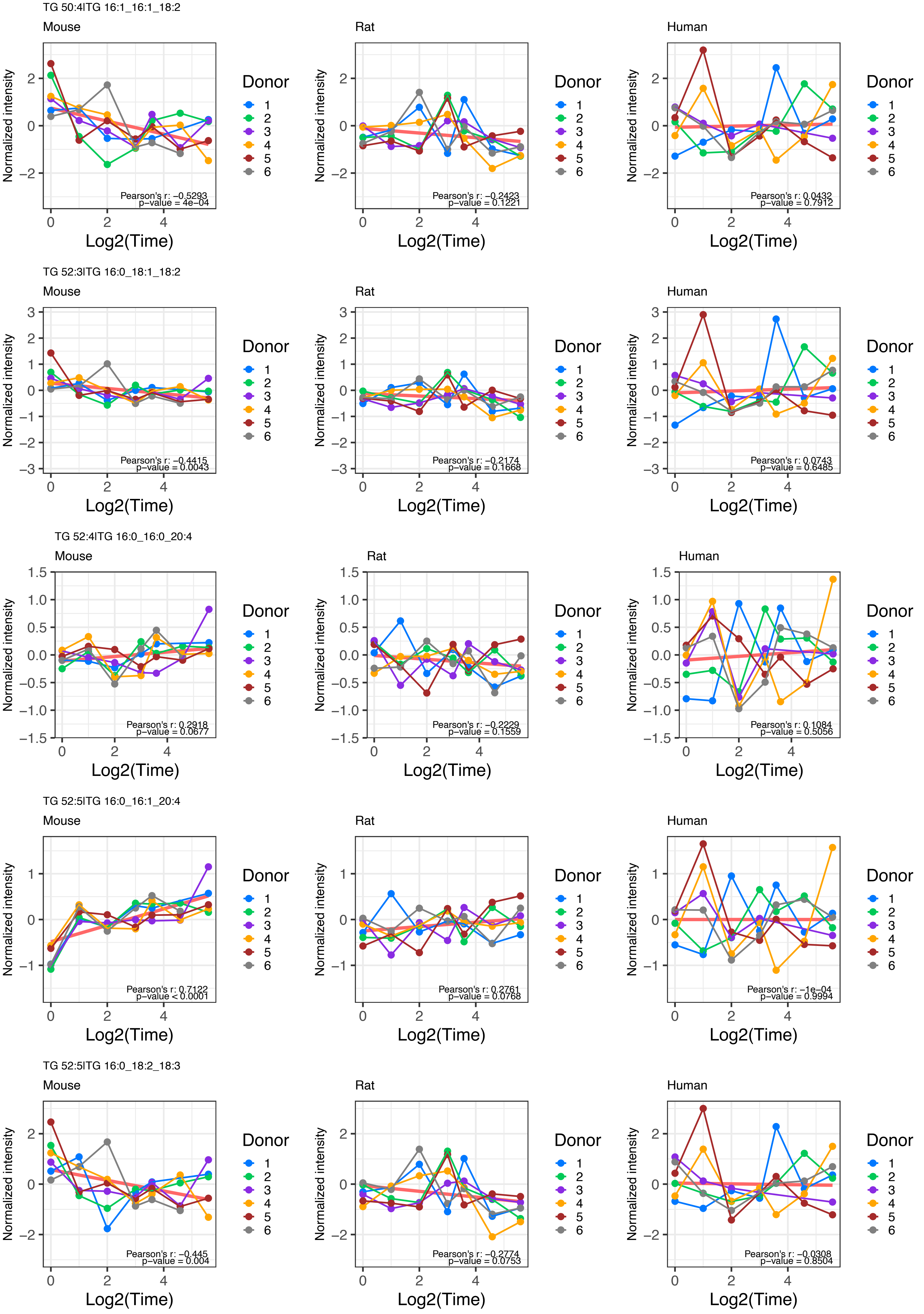

TG 50:4|TG 16:1_16:1_18:2
Mouse
Pearson's r: −0.5293
p−value = 4e−04
Rat
Pearson's r: −0.2423
p−value = 0.1221
Human
Pearson's r: 0.0432
p−value = 0.7912
TG 52:3|TG 16:0_18:1_18:2
Mouse
Pearson's r: −0.4415
p−value = 0.0043
Rat
Pearson's r: −0.2174
p−value = 0.1668
Human
Pearson's r: 0.0743
p−value = 0.6485
TG 52:4|TG 16:0_16:0_20:4
Mouse
Pearson's r: 0.2918
p−value = 0.0677
Rat
Pearson's r: −0.2229
p−value = 0.1559
Human
Pearson's r: 0.1084
p−value = 0.5056
TG 52:5|TG 16:0_16:1_20:4
Mouse
Pearson's r: 0.7122
p−value < 0.0001
Rat
Pearson's r: 0.2761
p−value = 0.0768
Human
Pearson's r: −1e−04
p−value = 0.9994
TG 52:5|TG 16:0_18:2_18:3
Mouse
Pearson's r: −0.445
p−value = 0.004
Rat
Pearson's r: −0.2774
p−value = 0.0753
Human
Pearson's r: −0.0308
p−value = 0.8504
Normalized intensity
Log2(Time)
Donor
1
2
3
4
5
6

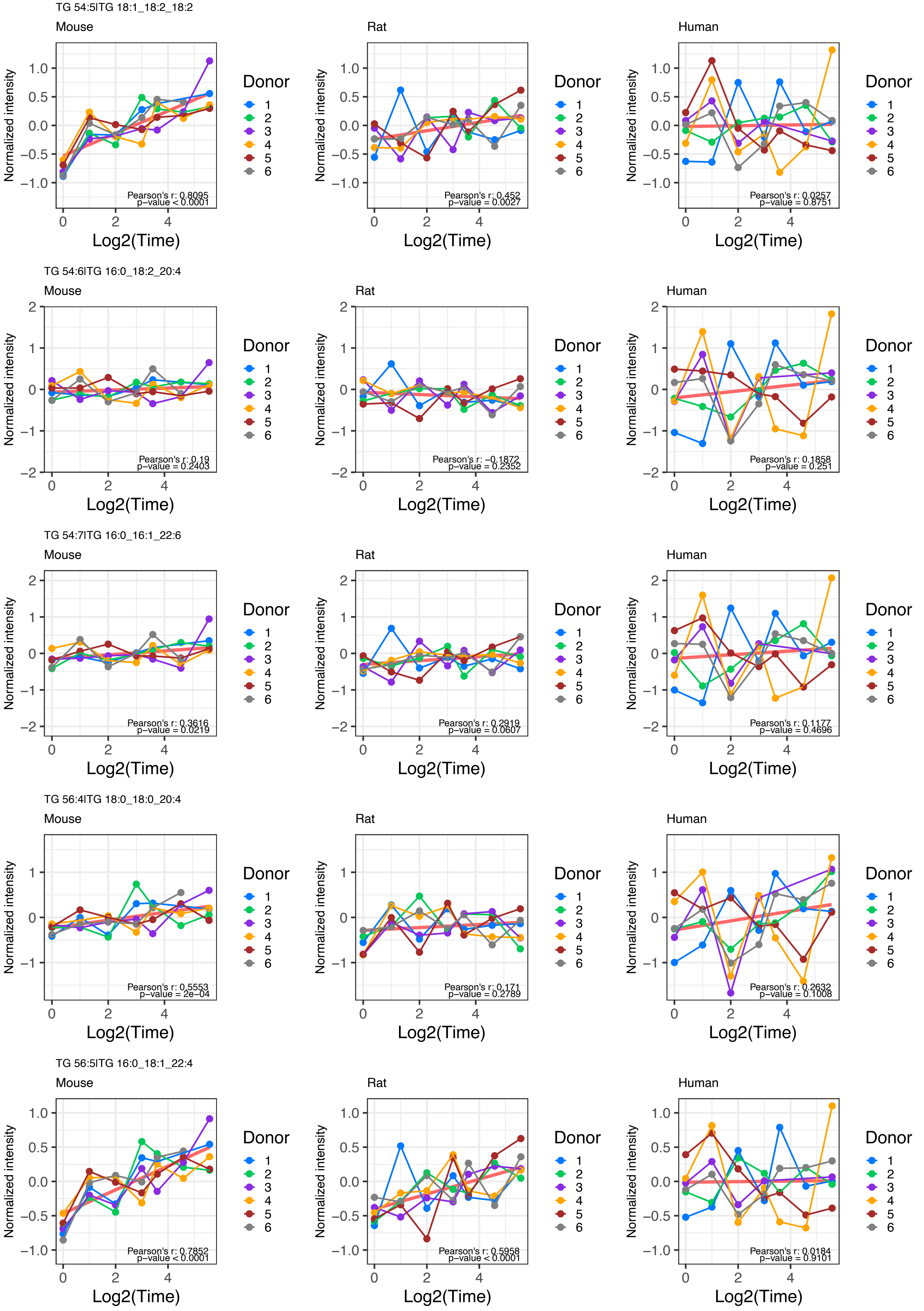
TG 54:5|TG 18:1_18:2_18:2
Mouse
Pearson's r: 0.8095
p–value < 0.0001
Rat
Pearson's r: 0.452
p–value = 0.0027
Human
Pearson's r: 0.0257
p–value = 0.8751
TG 54:6|TG 16:0_18:2_20:4
Mouse
Pearson's r: 0.19
p–value = 0.2403
Rat
Pearson's r: −0.1872
p–value = 0.2352
Human
Pearson's r: 0.1858
p–value = 0.251
TG 54:7|TG 16:0_16:1_22:6
Mouse
Pearson's r: 0.3616
p–value = 0.0219
Rat
Pearson's r: 0.2919
p–value = 0.0607
Human
Pearson's r: 0.1177
p–value = 0.4696
TG 56:4|TG 18:0_18:0_20:4
Mouse
Pearson's r: 0.5553
p–value = 2e−04
Rat
Pearson's r: 0.171
p–value = 0.2789
Human
Pearson's r: 0.2632
p–value = 0.1008
TG 56:5|TG 16:0_18:1_22:4
Mouse
Pearson's r: 0.7852
p–value < 0.0001
Rat
Pearson's r: 0.5958
p–value < 0.0001
Human
Pearson's r: 0.0184
p–value = 0.9101
Normalized intensity
Log2(Time)
Donor
1
2
3
4
5
6

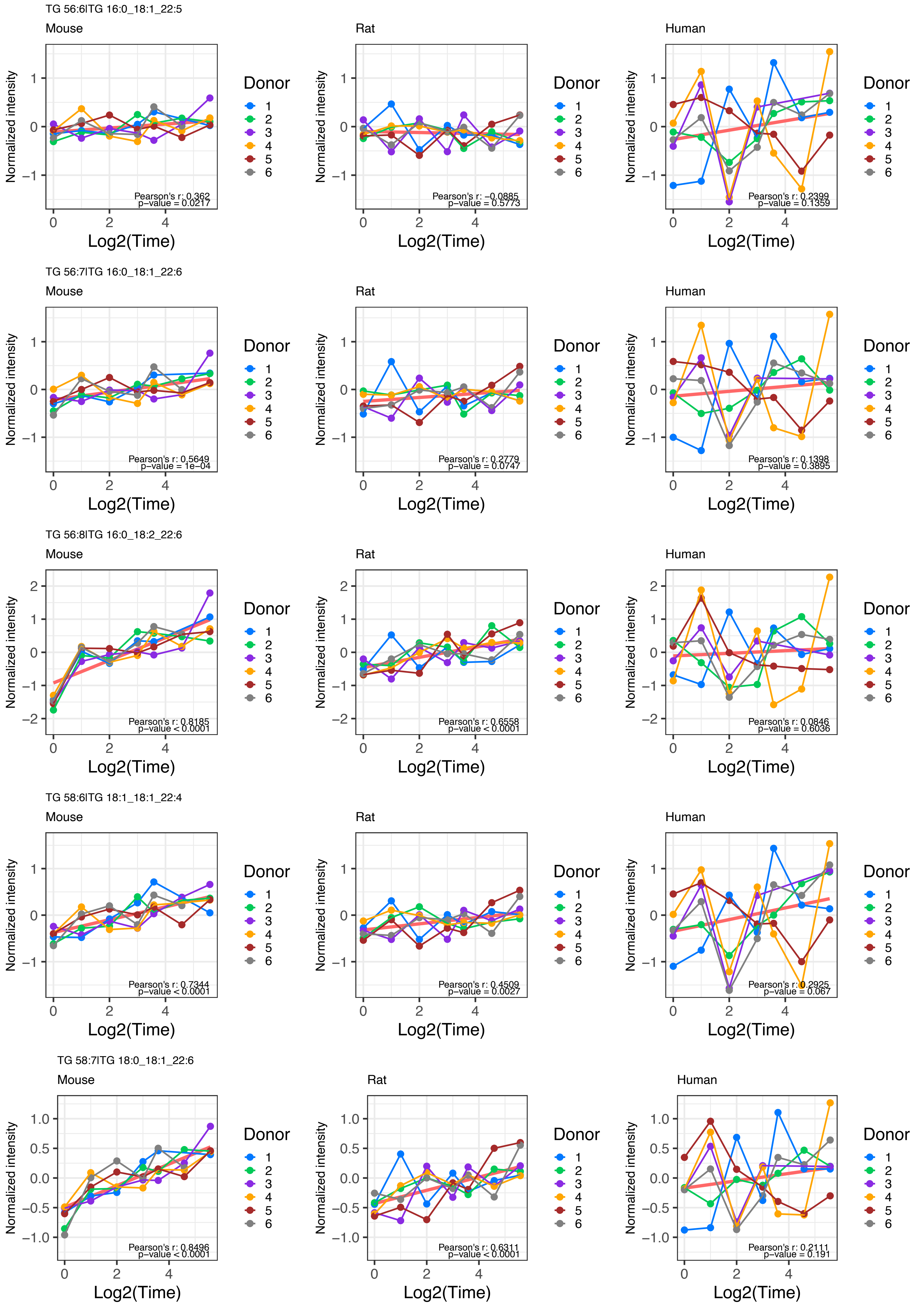

TG 56:6|TG 16:0_18:1_22:5
Mouse
Pearson's r: 0.362
p−value = 0.0217
Rat
Pearson's r: −0.0885
p−value = 0.5773
Human
Pearson's r: 0.2399
p−value = 0.1359
TG 56:7|TG 16:0_18:1_22:6
Mouse
Pearson's r: 0.5649
p−value = 1e−04
Rat
Pearson's r: 0.2779
p−value = 0.0747
Human
Pearson's r: 0.1398
p−value = 0.3895
TG 56:8|TG 16:0_18:2_22:6
Mouse
Pearson's r: 0.8185
p−value < 0.0001
Rat
Pearson's r: 0.6558
p−value < 0.0001
Human
Pearson's r: 0.0846
p−value = 0.6036
TG 58:6|TG 18:1_18:1_22:4
Mouse
Pearson's r: 0.7344
p−value < 0.0001
Rat
Pearson's r: 0.4509
p−value = 0.0027
Human
Pearson's r: 0.2925
p−value = 0.067
TG 58:7|TG 18:0_18:1_22:6
Mouse
Pearson's r: 0.8496
p−value < 0.0001
Rat
Pearson's r: 0.6311
p−value < 0.0001
Human
Pearson's r: 0.2111
p−value = 0.191
Normalized intensity
Log2(Time)
Donor
1
2
3
4
5
6

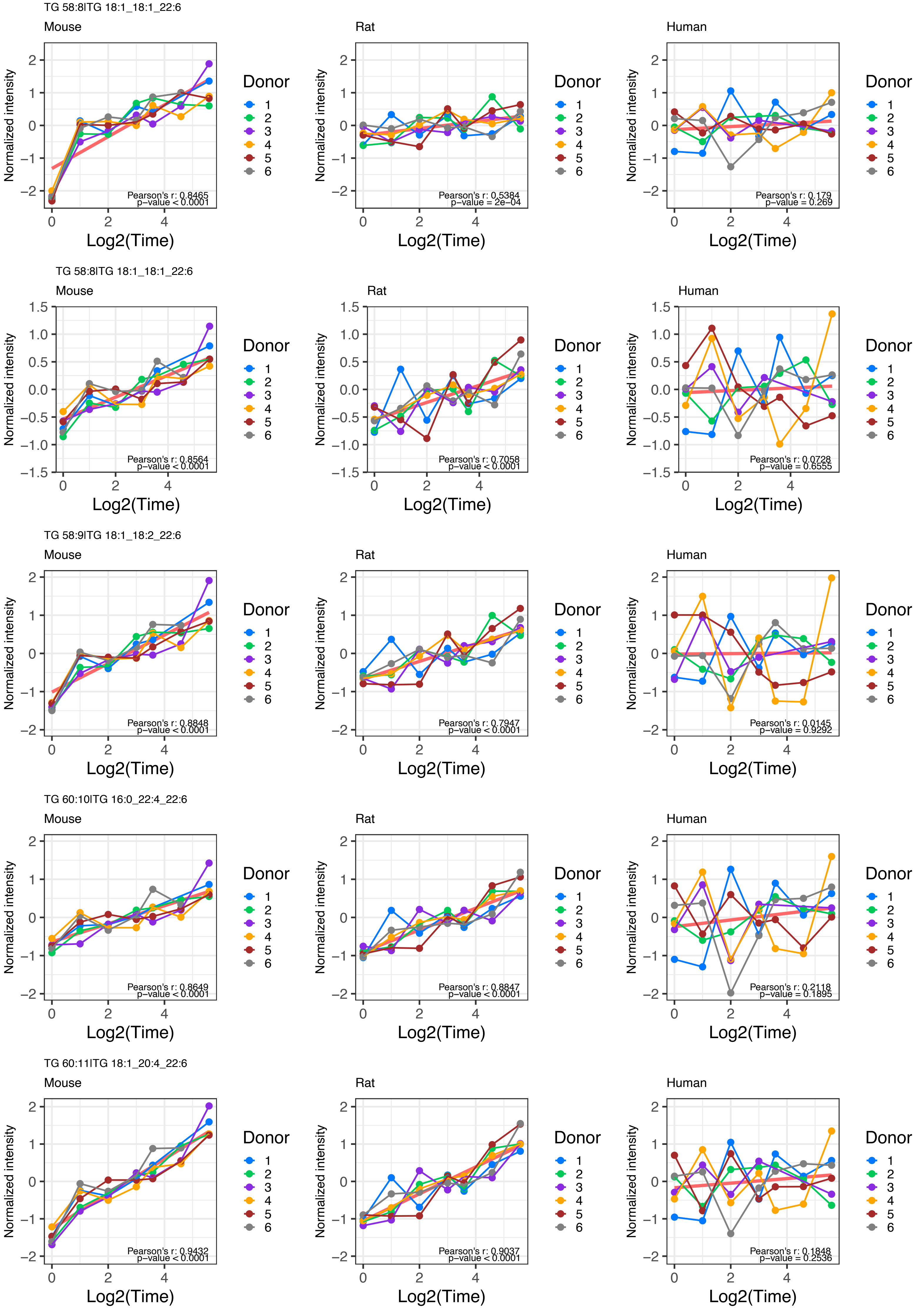
TG 58:8|TG 18:1_18:1_22:6
Mouse
Pearson's r: 0.8465
p−value < 0.0001
Rat
Pearson's r: 0.5384
p−value = 2e−04
Human
Pearson's r: 0.179
p−value = 0.269
TG 58:8|TG 18:1_18:1_22:6
Mouse
Pearson's r: 0.8564
p−value < 0.0001
Rat
Pearson's r: 0.7058
p−value < 0.0001
Human
Pearson's r: 0.0728
p−value = 0.6555
TG 58:9|TG 18:1_18:2_22:6
Mouse
Pearson's r: 0.8848
p−value < 0.0001
Rat
Pearson's r: 0.7947
p−value < 0.0001
Human
Pearson's r: 0.0145
p−value = 0.9292
TG 60:10|TG 16:0_22:4_22:6
Mouse
Pearson's r: 0.8649
p−value < 0.0001
Rat
Pearson's r: 0.8847
p−value < 0.0001
Human
Pearson's r: 0.2118
p−value = 0.1895
TG 60:11|TG 18:1_20:4_22:6
Mouse
Pearson's r: 0.9432
p−value < 0.0001
Rat
Pearson's r: 0.9037
p−value < 0.0001
Human
Pearson's r: 0.1848
p−value = 0.2536
Normalized intensity
Log2(Time)
Donor
1
2
3
4
5
6

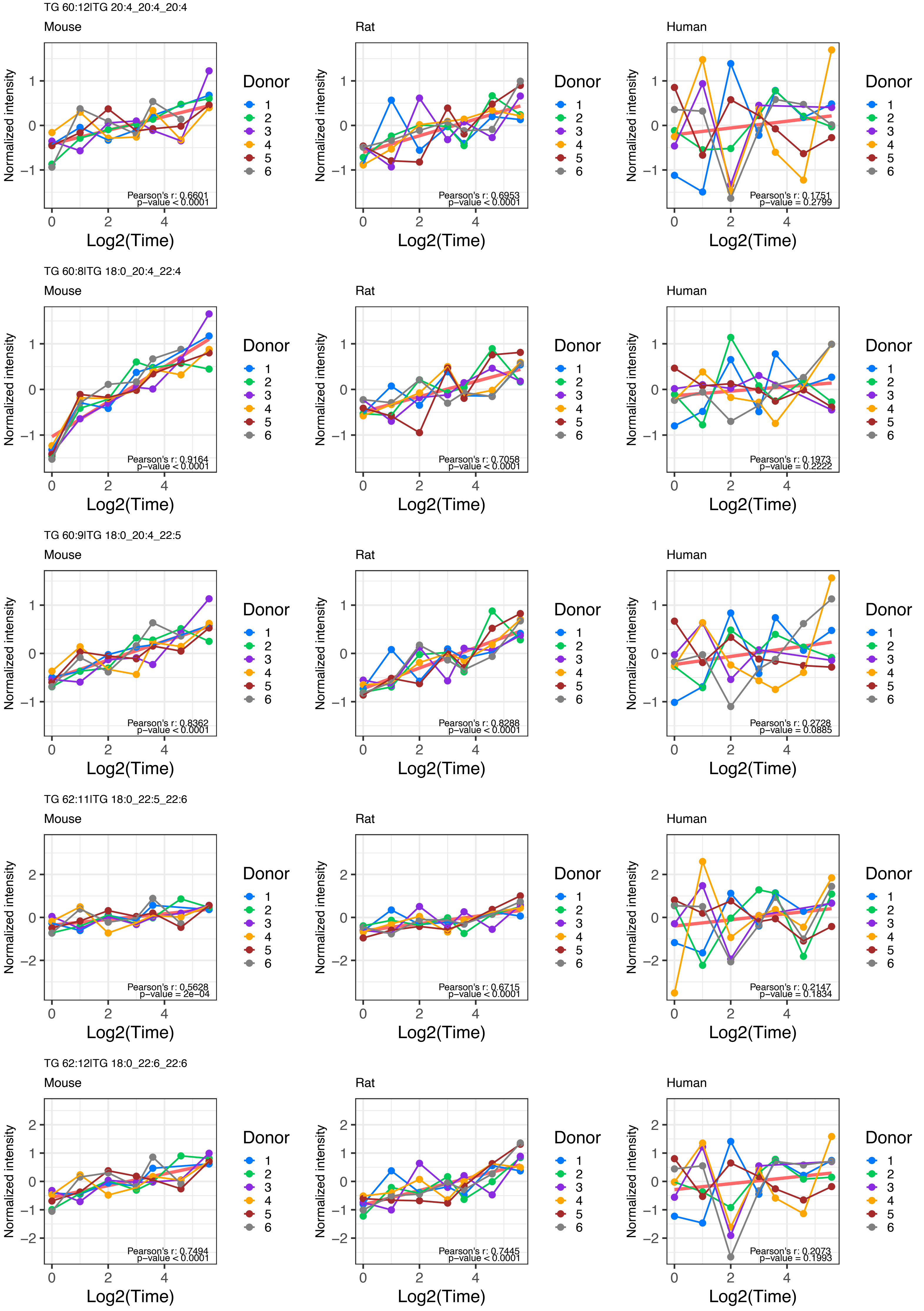
TG 60:12|TG 20:4_20:4_20:4
Mouse
Pearson's r: 0.6601
p−value < 0.0001
Rat
Pearson's r: 0.6953
p−value < 0.0001
Human
Pearson's r: 0.1751
p−value = 0.2799
TG 60:8|TG 18:0_20:4_22:4
Mouse
Pearson's r: 0.9164
p−value < 0.0001
Rat
Pearson's r: 0.7058
p−value < 0.0001
Human
Pearson's r: 0.1973
p−value = 0.2222
TG 60:9|TG 18:0_20:4_22:5
Mouse
Pearson's r: 0.8362
p−value < 0.0001
Rat
Pearson's r: 0.8288
p−value < 0.0001
Human
Pearson's r: 0.2728
p−value = 0.0885
TG 62:11|TG 18:0_22:5_22:6
Mouse
Pearson's r: 0.5628
p−value = 2e−04
Rat
Pearson's r: 0.6715
p−value < 0.0001
Human
Pearson's r: 0.2147
p−value = 0.1834
TG 62:12|TG 18:0_22:6_22:6
Mouse
Pearson's r: 0.7494
p−value < 0.0001
Rat
Pearson's r: 0.7445
p−value < 0.0001
Human
Pearson's r: 0.2073
p−value = 0.1993
Normalized intensity
Log2(Time)
Donor
1
2
3
4
5
6

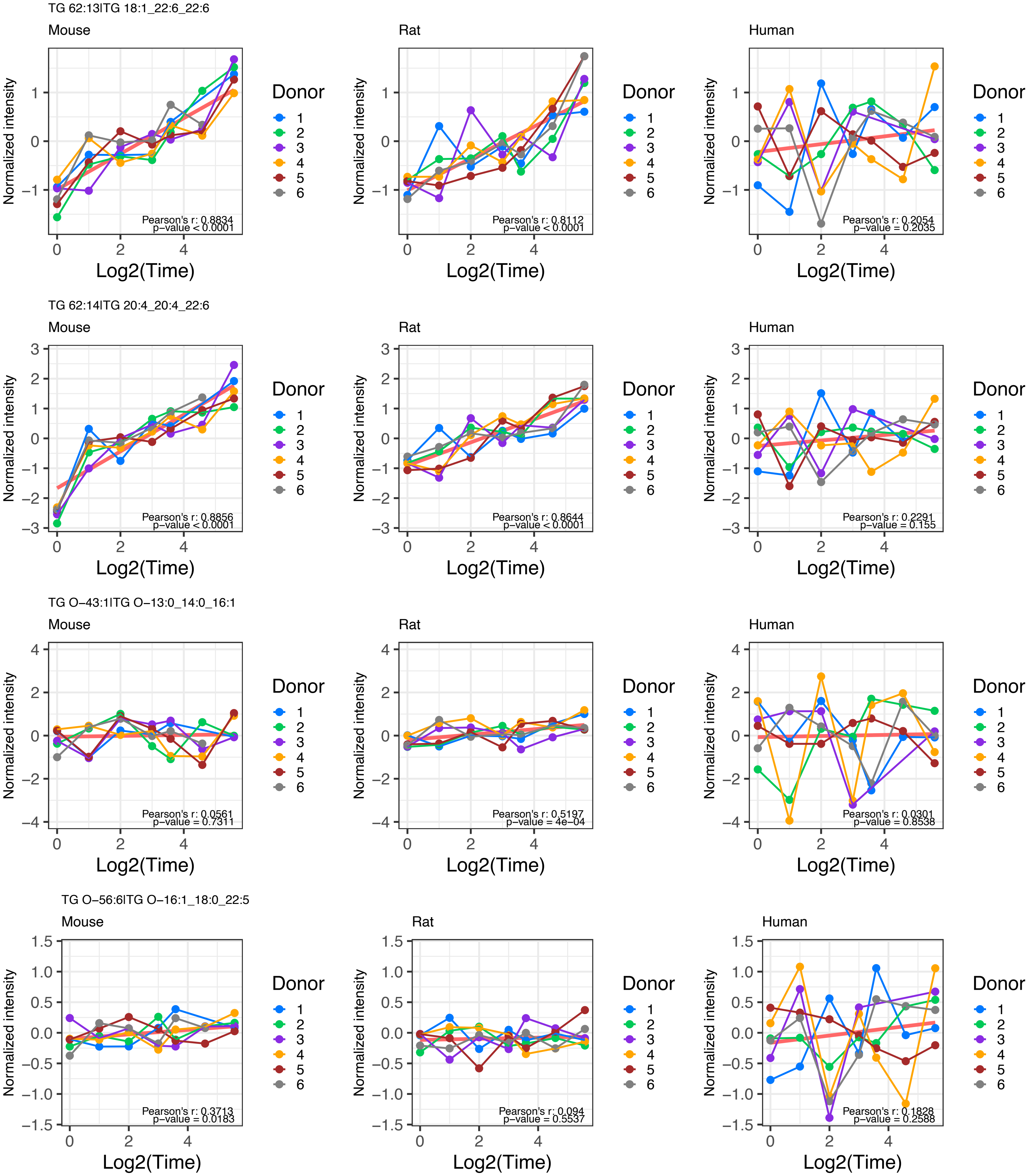
TG 62:13|TG 18:1_22:6_22:6
Mouse
Rat
Human
Normalized intensity
Log2(Time)
Donor
1
2
3
4
5
6
Pearson's r: 0.8834
p−value < 0.0001
Pearson's r: 0.8112
p−value < 0.0001
Pearson's r: 0.2054
p−value = 0.2035
TG 62:14|TG 20:4_20:4_22:6
Mouse
Rat
Human
Pearson's r: 0.8856
p−value < 0.0001
Pearson's r: 0.8644
p−value < 0.0001
Pearson's r: 0.2291
p−value = 0.155
TG O−43:1|TG O−13:0_14:0_16:1
Mouse
Rat
Human
Pearson's r: 0.0561
p−value = 0.7311
Pearson's r: 0.5197
p−value = 4e−04
Pearson's r: 0.0301
p−value = 0.8538
TG O−56:6|TG O−16:1_18:0_22:5
Mouse
Rat
Human
Pearson's r: 0.3713
p−value = 0.0183
Pearson's r: 0.094
p−value = 0.5537
Pearson's r: 0.1828
p−value = 0.2588

Figure S2. Normalized postmortem intensity changes of individual polar metabolites in mouse, rat, and human brain tissue. Compound and species names are shown on top of the plot and the corresponding panels, respectively. Each line represents an individual donor (n=6 per species). Pearson's correlation coefficient (r) and significance (p-value) of the relationship between normalized polar metabolite abundance levels and $\log_2$-transformed postmortem interval duration are indicated within each panel.

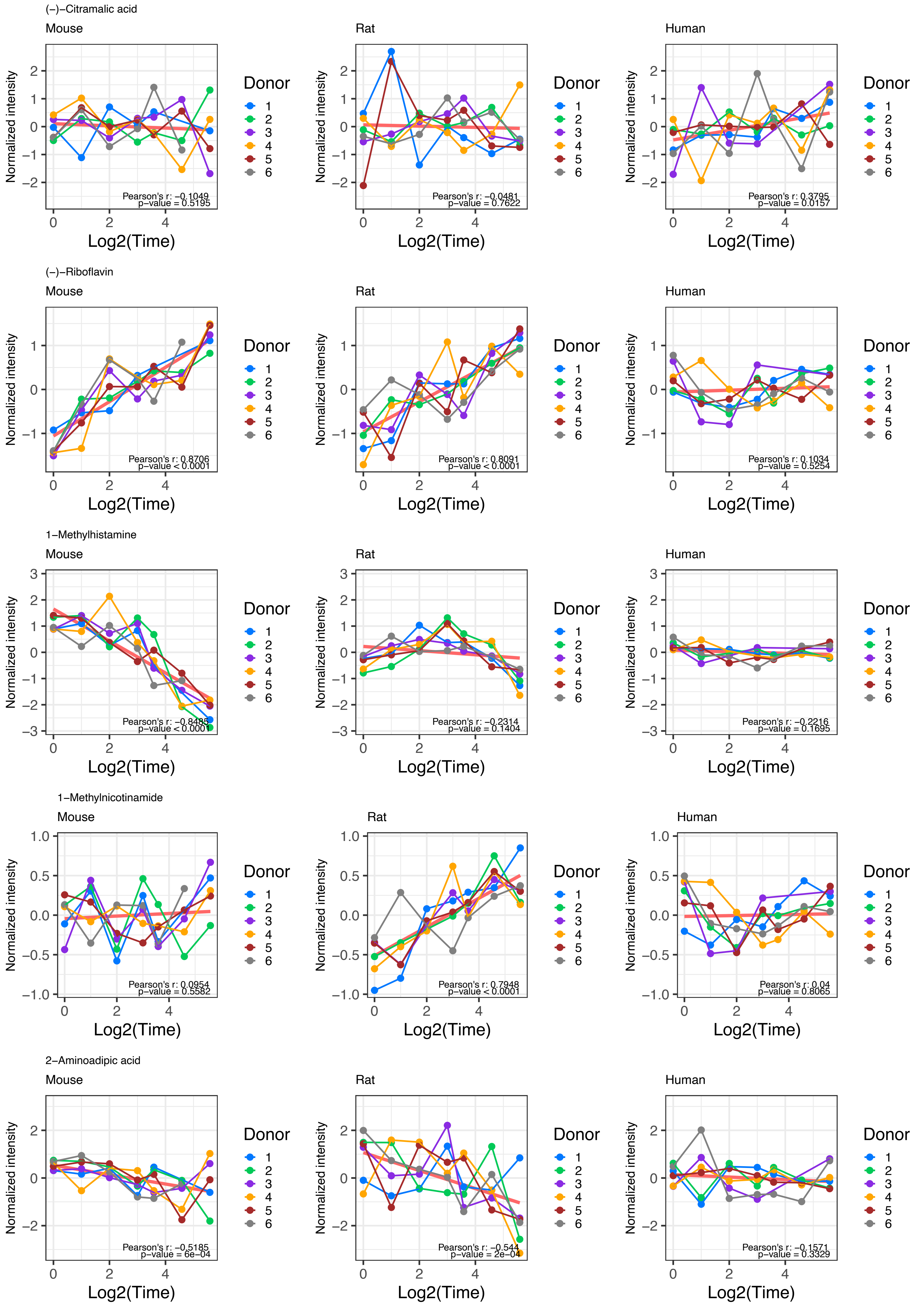

(−)−Citramalic acid
Mouse
Rat
Human
Normalized intensity
Log2(Time)
Donor
1
2
3
4
5
6
Pearson's r: −0.1049
p−value = 0.5195
Pearson's r: −0.0481
p−value = 0.7622
Pearson's r: 0.3795
p−value = 0.0157
(−)−Riboflavin
Pearson's r: 0.8706
p−value < 0.0001
Pearson's r: 0.8091
p−value < 0.0001
Pearson's r: 0.1034
p−value = 0.5254
1−Methylhistamine
Pearson's r: −0.8485
p−value < 0.0001
Pearson's r: −0.2314
p−value = 0.1404
Pearson's r: −0.2216
p−value = 0.1695
1−Methylnicotinamide
Pearson's r: 0.0954
p−value = 0.5582
Pearson's r: 0.7948
p−value < 0.0001
Pearson's r: 0.04
p−value = 0.8065
2−Aminoadipic acid
Pearson's r: −0.5185
p−value = 6e−04
Pearson's r: −0.544
p−value = 2e−04
Pearson's r: −0.1571
p−value = 0.3329

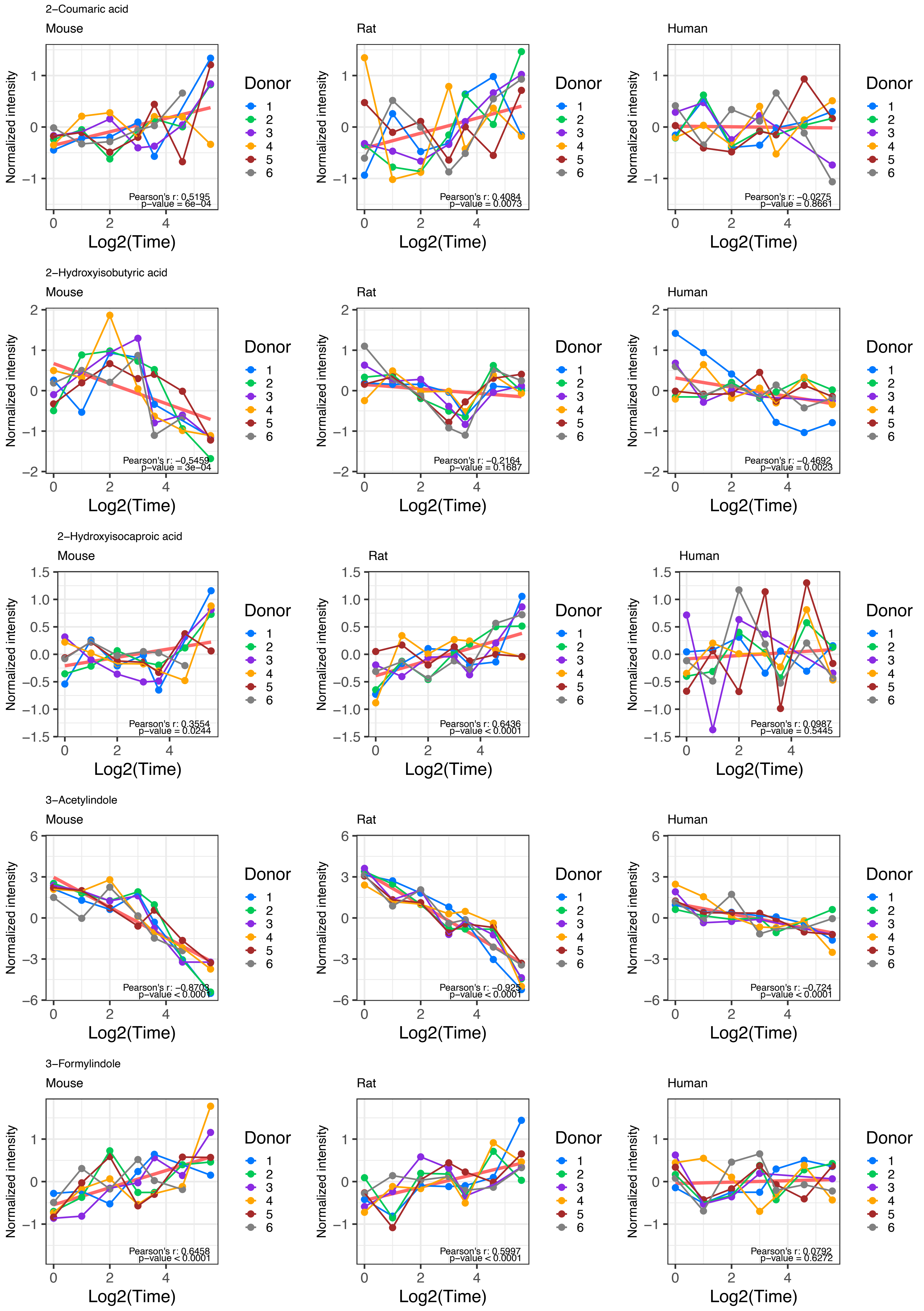

2−Coumaric acid
Mouse
Rat
Human
Normalized intensity
Log2(Time)
Donor
1
2
3
4
5
6
Pearson's r: 0.5195
p−value = 6e−04
Pearson's r: 0.4084
p−value = 0.0073
Pearson's r: −0.0275
p−value = 0.8661
2−Hydroxyisobutyric acid
Pearson's r: −0.5459
p−value = 3e−04
Pearson's r: −0.2164
p−value = 0.1687
Pearson's r: −0.4692
p−value = 0.0023
2−Hydroxyisocaproic acid
Pearson's r: 0.3554
p−value = 0.0244
Pearson's r: 0.6436
p−value < 0.0001
Pearson's r: 0.0987
p−value = 0.5445
3−Acetylindole
Pearson's r: −0.8708
p−value < 0.0001
Pearson's r: −0.925
p−value < 0.0001
Pearson's r: −0.724
p−value < 0.0001
3−Formylindole
Pearson's r: 0.6458
p−value < 0.0001
Pearson's r: 0.5997
p−value < 0.0001
Pearson's r: 0.0792
p−value = 0.6272

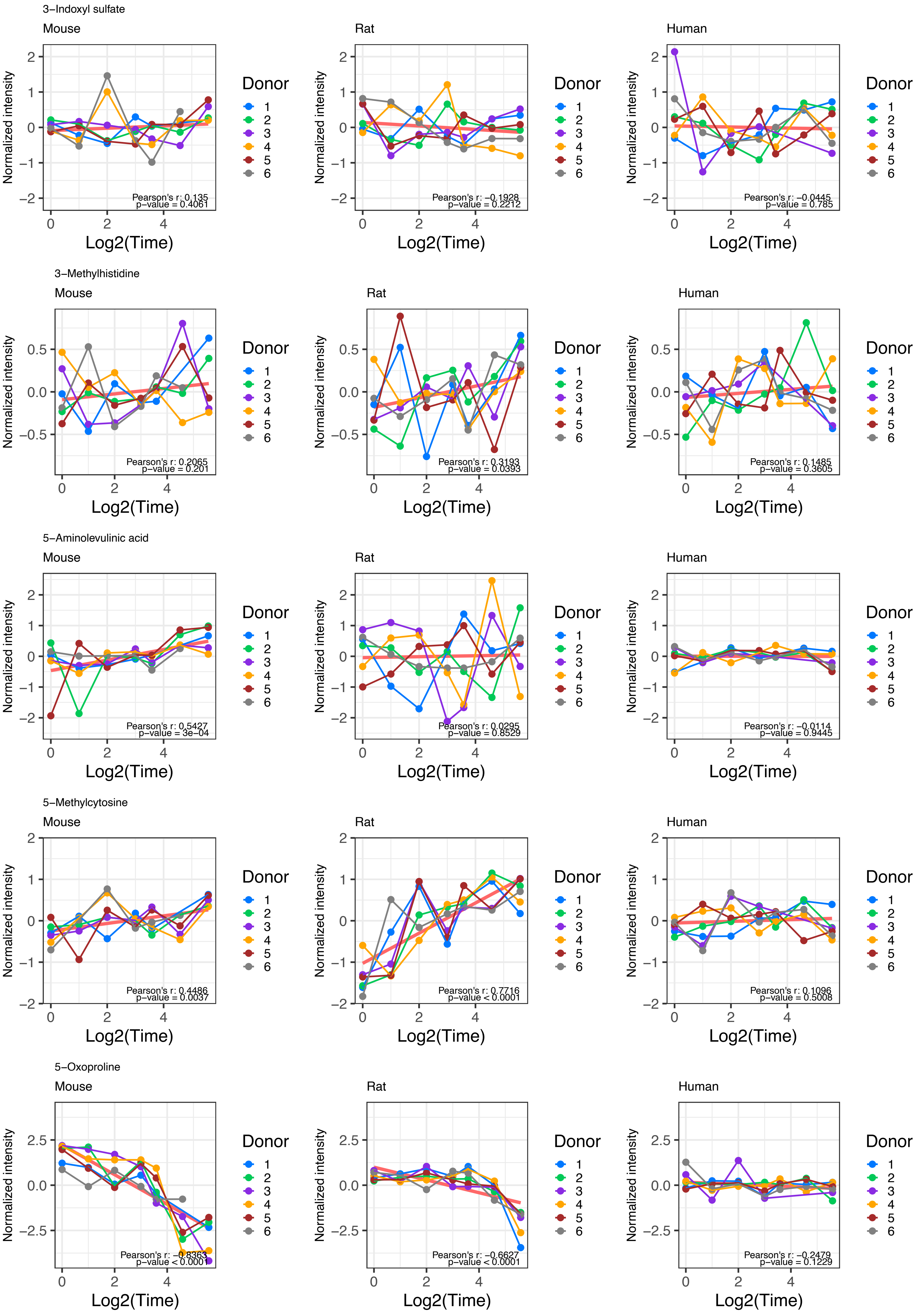
3−Indoxyl sulfate
Mouse
Pearson's r: 0.135
p−value = 0.4061
Rat
Pearson's r: −0.1928
p−value = 0.2212
Human
Pearson's r: −0.0445
p−value = 0.785
3−Methylhistidine
Mouse
Pearson's r: 0.2065
p−value = 0.201
Rat
Pearson's r: 0.3193
p−value = 0.0393
Human
Pearson's r: 0.1485
p−value = 0.3605
5−Aminolevulinic acid
Mouse
Pearson's r: 0.5427
p−value = 3e−04
Rat
Pearson's r: 0.0295
p−value = 0.8529
Human
Pearson's r: −0.0114
p−value = 0.9445
5−Methylcytosine
Mouse
Pearson's r: 0.4486
p−value = 0.0037
Rat
Pearson's r: 0.7716
p−value < 0.0001
Human
Pearson's r: 0.1096
p−value = 0.5008
5−Oxoproline
Mouse
Pearson's r: −0.8363
p−value < 0.0001
Rat
Pearson's r: −0.6627
p−value < 0.0001
Human
Pearson's r: −0.2479
p−value = 0.1229
Normalized intensity
Log2(Time)
Donor
1
2
3
4
5
6

5,6−Dihydrouracil

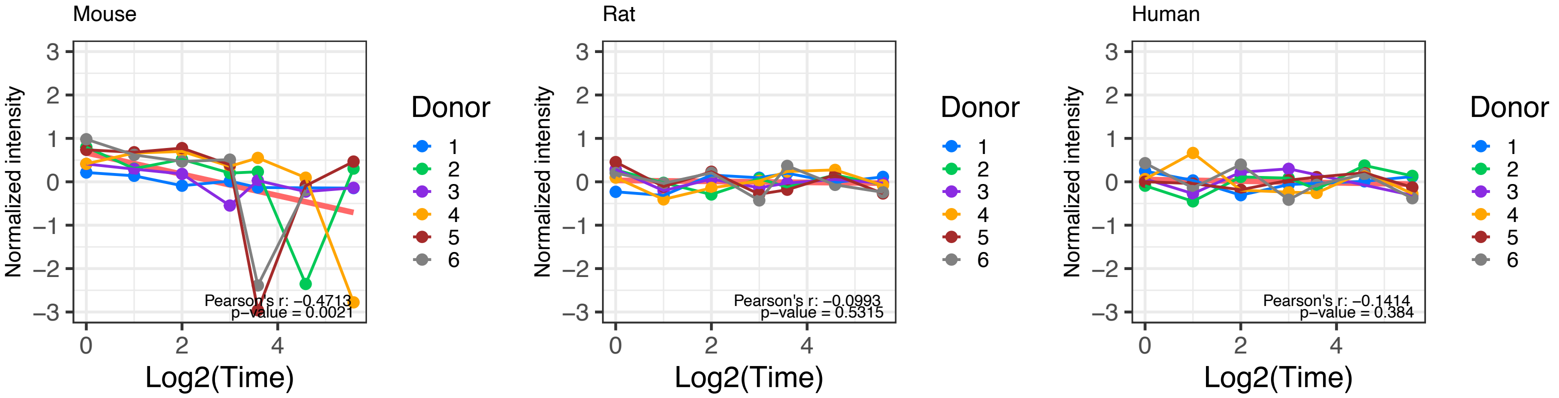

5'−S−Methylthioadenosine

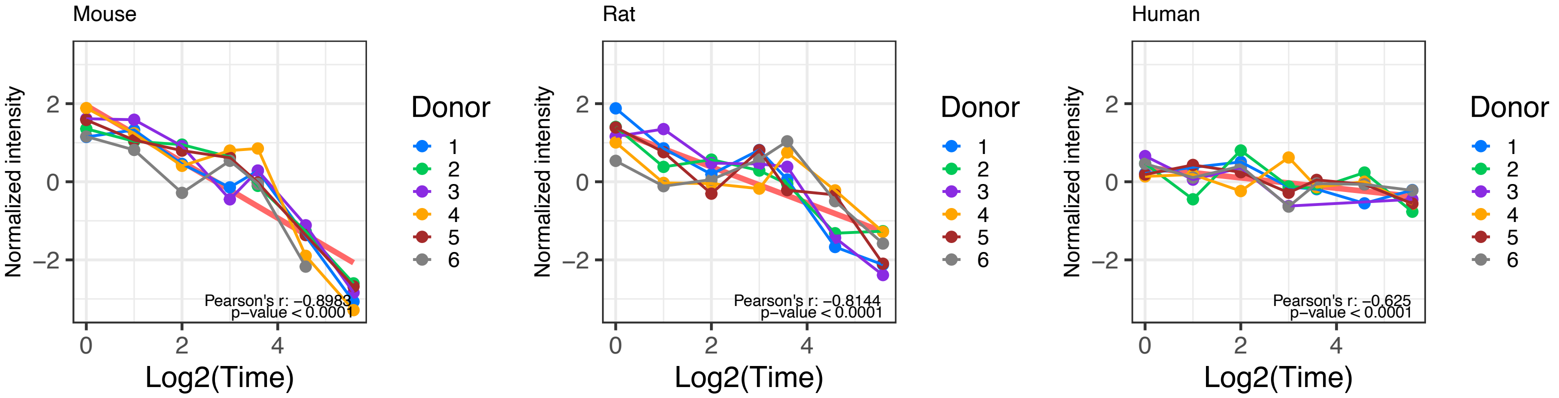

Acetylcholine

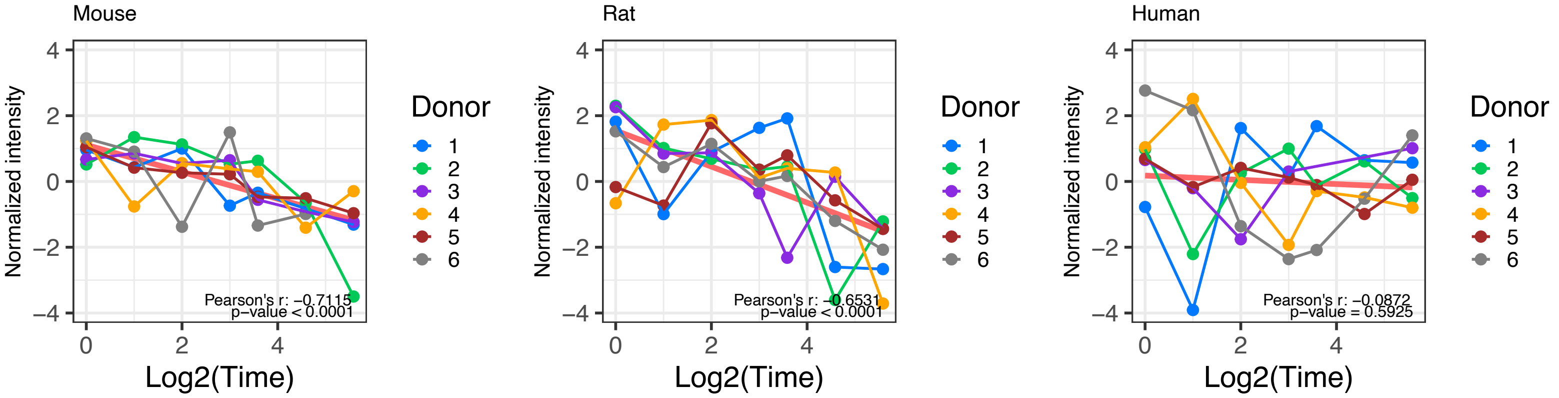

Adenine

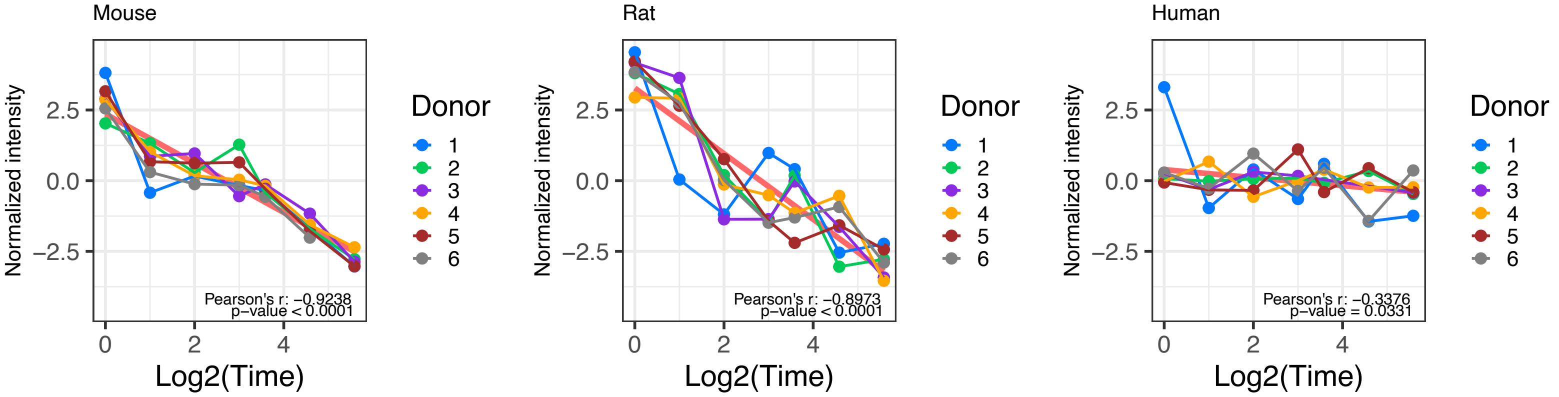

Adenosine 3'−monophosphate

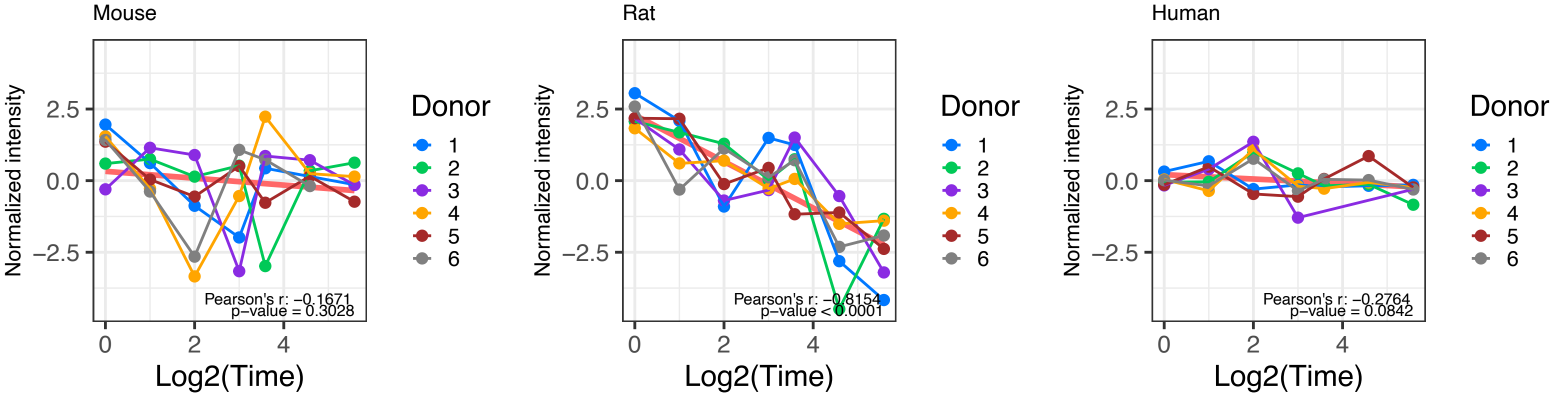

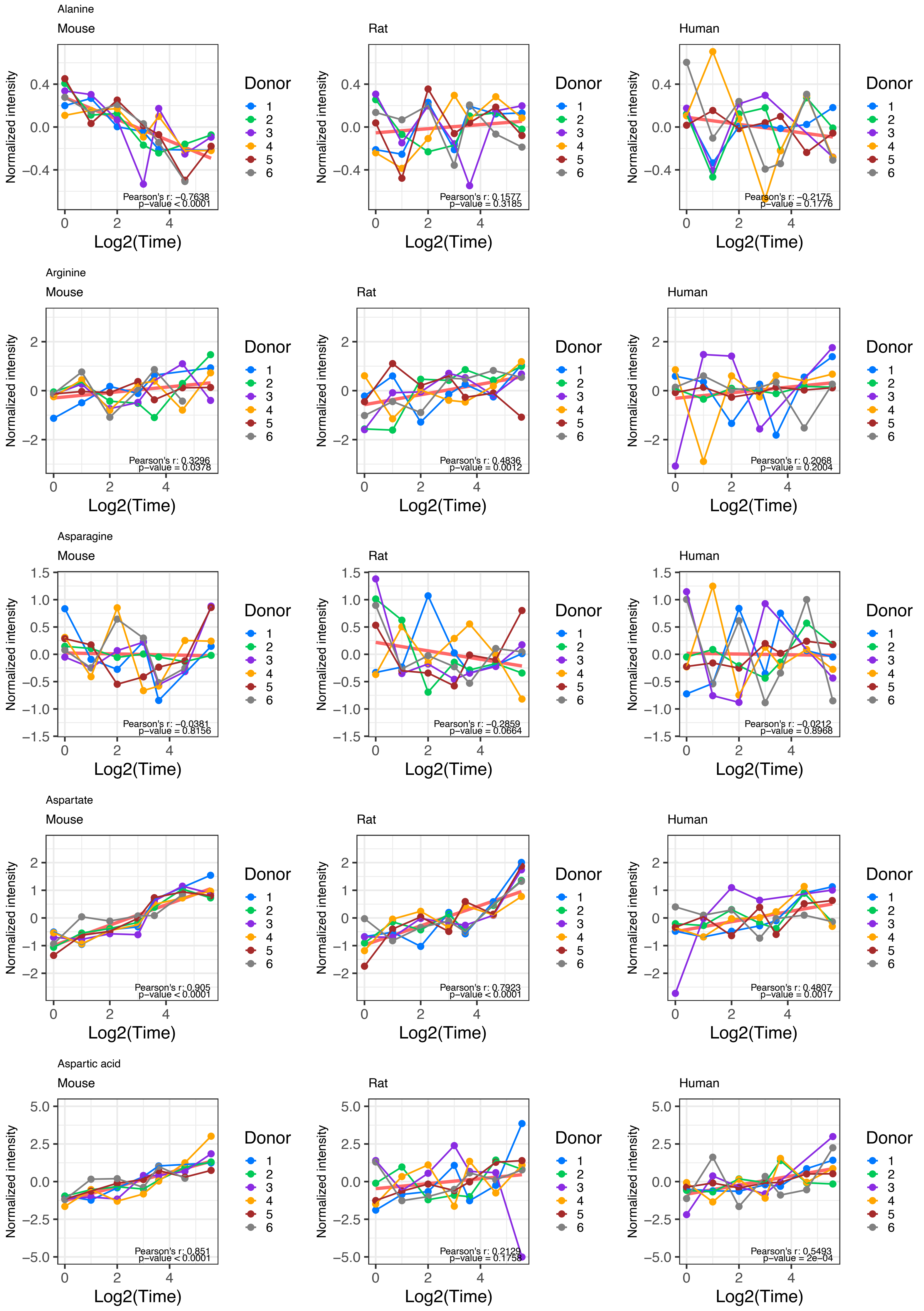
Alanine
Mouse
Pearson's r: −0.7638
p−value < 0.0001
Rat
Pearson's r: 0.1577
p−value = 0.3185
Human
Pearson's r: −0.2175
p−value = 0.1776
Arginine
Mouse
Pearson's r: 0.3296
p−value = 0.0378
Rat
Pearson's r: 0.4836
p−value = 0.0012
Human
Pearson's r: 0.2068
p−value = 0.2004
Asparagine
Mouse
Pearson's r: −0.0381
p−value = 0.8156
Rat
Pearson's r: −0.2859
p−value = 0.0664
Human
Pearson's r: −0.0212
p−value = 0.8968
Aspartate
Mouse
Pearson's r: 0.905
p−value < 0.0001
Rat
Pearson's r: 0.7923
p−value < 0.0001
Human
Pearson's r: 0.4807
p−value = 0.0017
Aspartic acid
Mouse
Pearson's r: 0.851
p−value < 0.0001
Rat
Pearson's r: 0.2129
p−value = 0.1758
Human
Pearson's r: 0.5493
p−value = 2e−04
Normalized intensity
Log2(Time)
Donor
1
2
3
4
5
6

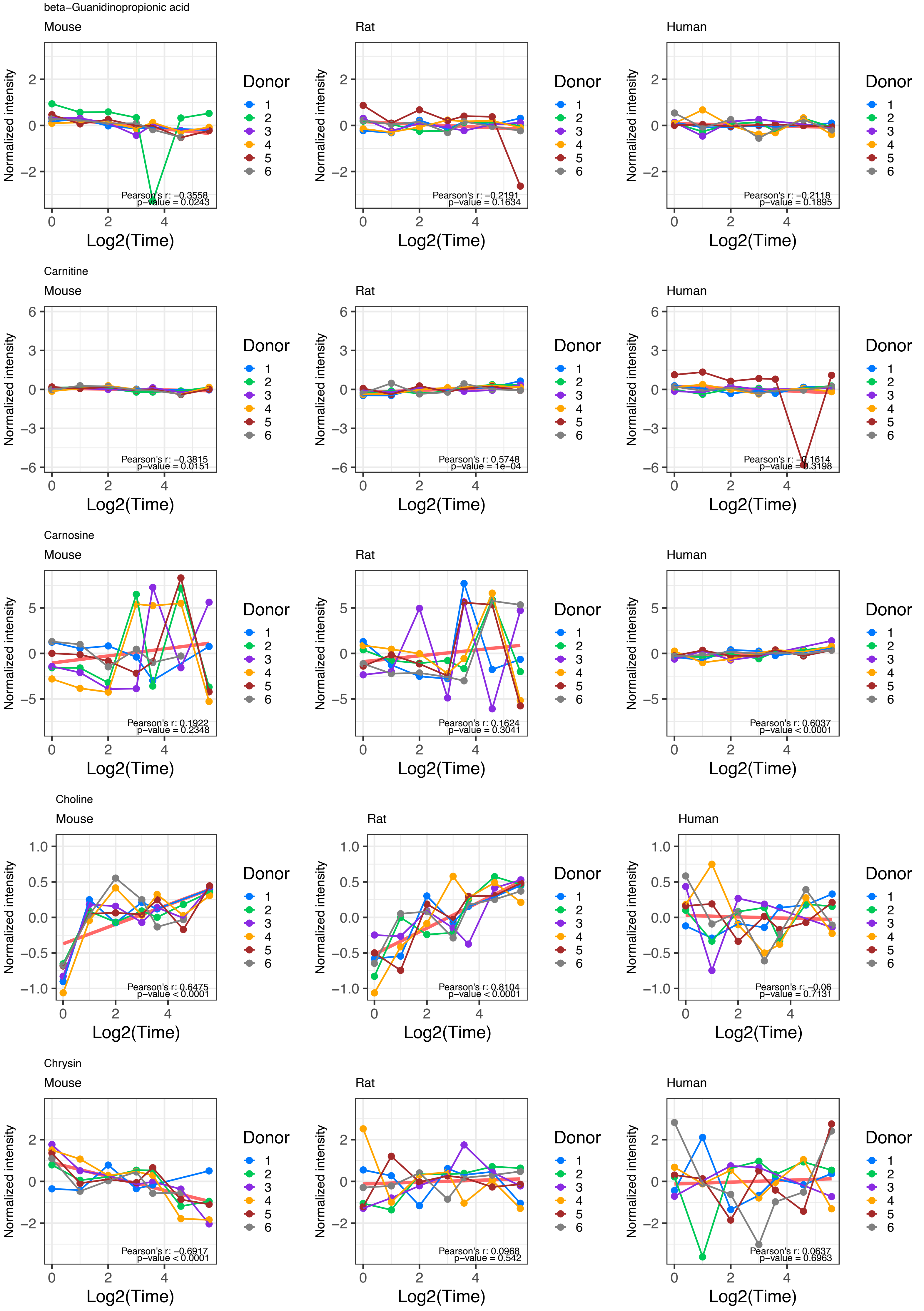

beta−Guanidinopropionic acid
Mouse
Pearson's r: −0.3558
p−value = 0.0243
Rat
Pearson's r: −0.2191
p−value = 0.1634
Human
Pearson's r: −0.2118
p−value = 0.1895
Carnitine
Mouse
Pearson's r: −0.3815
p−value = 0.0151
Rat
Pearson's r: 0.5748
p−value = 1e−04
Human
Pearson's r: −0.1614
p−value = 0.3198
Carnosine
Mouse
Pearson's r: 0.1922
p−value = 0.2348
Rat
Pearson's r: 0.1624
p−value = 0.3041
Human
Pearson's r: 0.6037
p−value < 0.0001
Choline
Mouse
Pearson's r: 0.6475
p−value < 0.0001
Rat
Pearson's r: 0.8104
p−value < 0.0001
Human
Pearson's r: −0.06
p−value = 0.7131
Chrysin
Mouse
Pearson's r: −0.6917
p−value < 0.0001
Rat
Pearson's r: 0.0968
p−value = 0.542
Human
Pearson's r: 0.0637
p−value = 0.6963
Normalized intensity
Log2(Time)
Donor
1
2
3
4
5
6

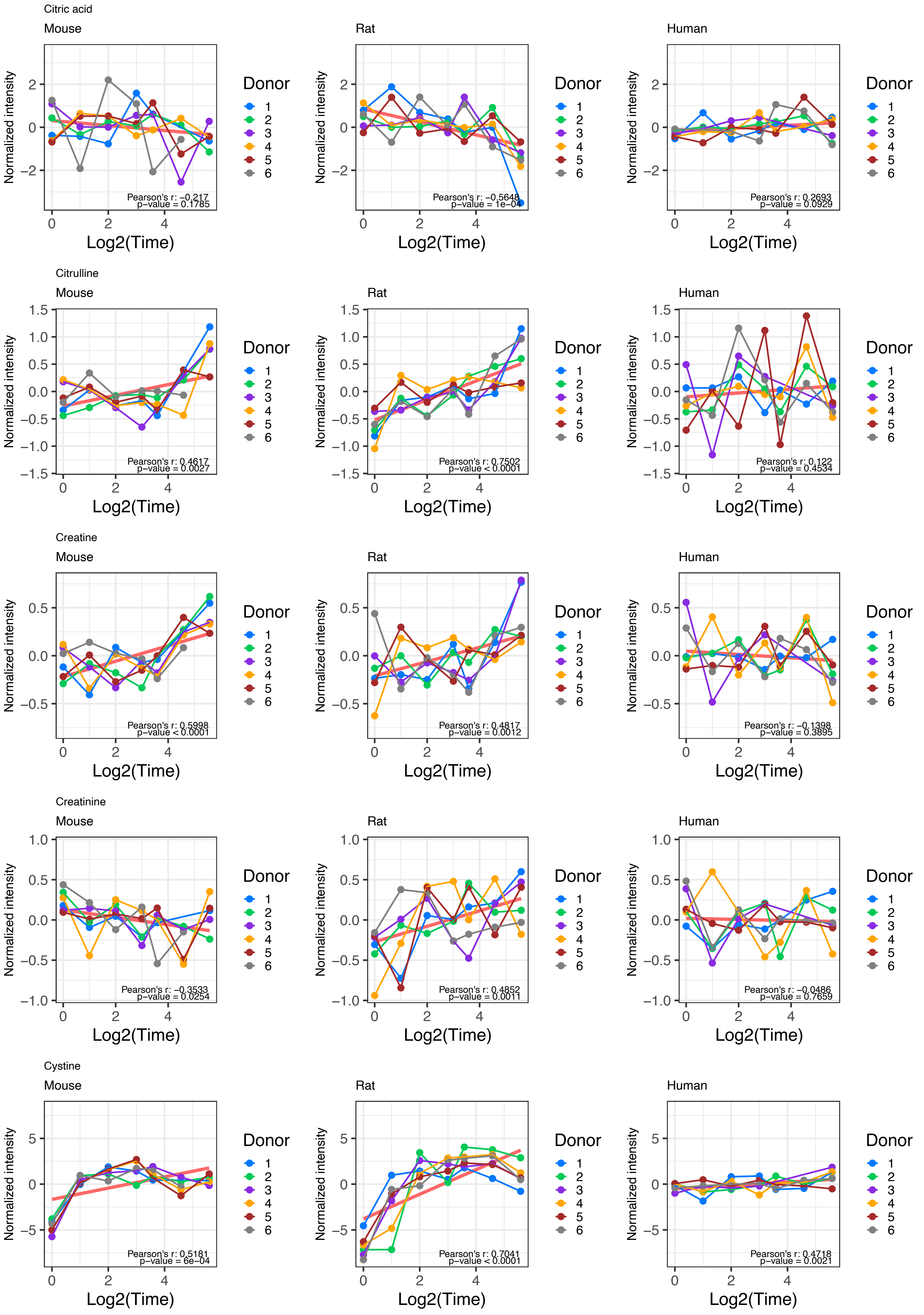
Citric acid
Mouse
Pearson's r: −0.217
p−value = 0.1785
Rat
Pearson's r: −0.5648
p−value = 1e−04
Human
Pearson's r: 0.2693
p−value = 0.0929
Citrulline
Mouse
Pearson's r: 0.4617
p−value = 0.0027
Rat
Pearson's r: 0.7502
p−value < 0.0001
Human
Pearson's r: 0.122
p−value = 0.4534
Creatine
Mouse
Pearson's r: 0.5998
p−value < 0.0001
Rat
Pearson's r: 0.4817
p−value = 0.0012
Human
Pearson's r: −0.1398
p−value = 0.3895
Creatinine
Mouse
Pearson's r: −0.3533
p−value = 0.0254
Rat
Pearson's r: 0.4852
p−value = 0.0011
Human
Pearson's r: −0.0486
p−value = 0.7659
Cystine
Mouse
Pearson's r: 0.5181
p−value = 6e−04
Rat
Pearson's r: 0.7041
p−value < 0.0001
Human
Pearson's r: 0.4718
p−value = 0.0021
Normalized intensity
Log2(Time)
Donor
1
2
3
4
5
6

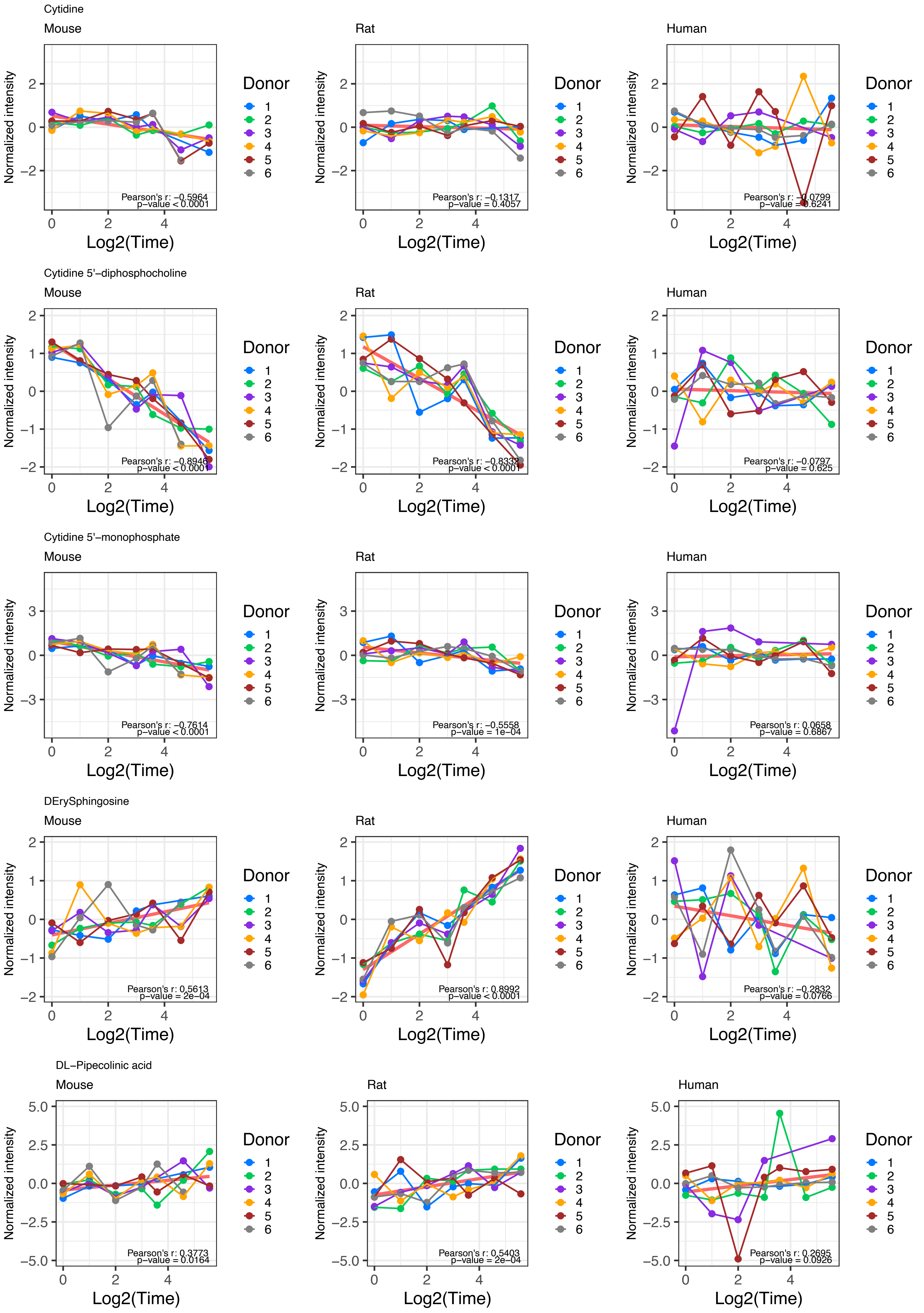
Cytidine
Mouse
Pearson's r: −0.5964
p−value < 0.0001
Rat
Pearson's r: −0.1317
p−value = 0.4057
Human
Pearson's r: −0.0799
p−value = 0.6241
Cytidine 5'−diphosphocholine
Mouse
Pearson's r: −0.8946
p−value < 0.0001
Rat
Pearson's r: −0.8332
p−value < 0.0001
Human
Pearson's r: −0.0797
p−value = 0.625
Cytidine 5'−monophosphate
Mouse
Pearson's r: −0.7614
p−value < 0.0001
Rat
Pearson's r: −0.5558
p−value = 1e−04
Human
Pearson's r: 0.0658
p−value = 0.6867
DErySphingosine
Mouse
Pearson's r: 0.5613
p−value = 2e−04
Rat
Pearson's r: 0.8992
p−value < 0.0001
Human
Pearson's r: −0.2832
p−value = 0.0766
DL−Pipecolinic acid
Mouse
Pearson's r: 0.3773
p−value = 0.0164
Rat
Pearson's r: 0.5403
p−value = 2e−04
Human
Pearson's r: 0.2695
p−value = 0.0926
Normalized intensity
Log2(Time)
Donor
1
2
3
4
5
6

GABA

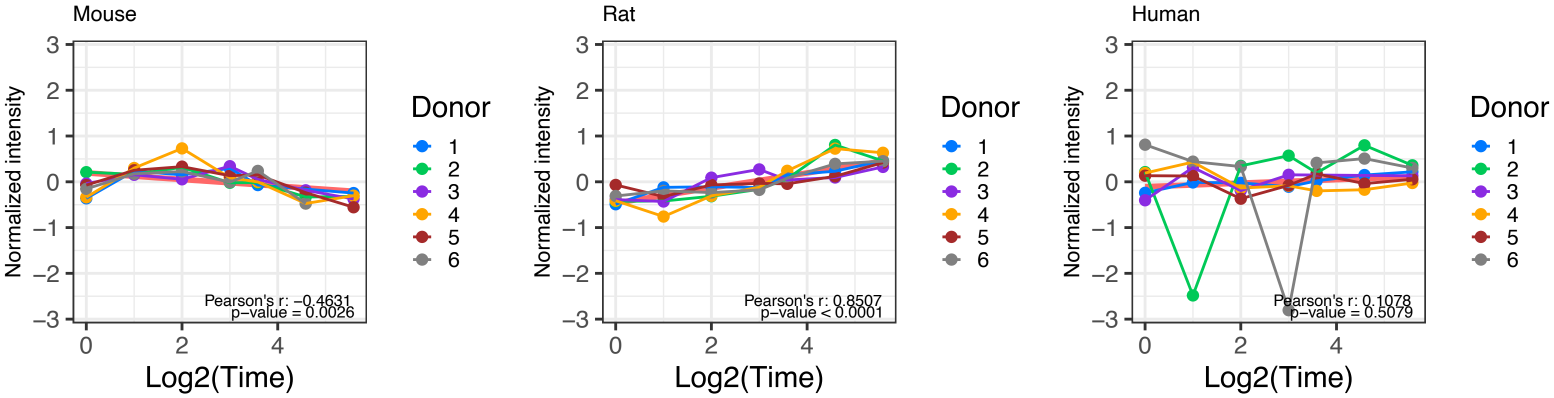

gamma−Glutamyl−L−glutamic Acid

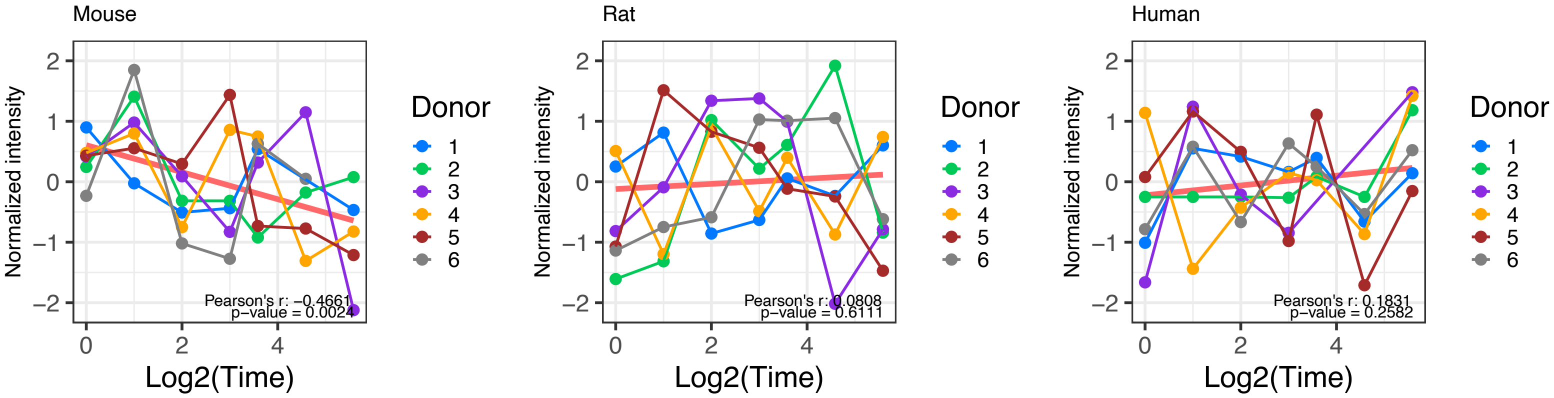

Gluconic acid

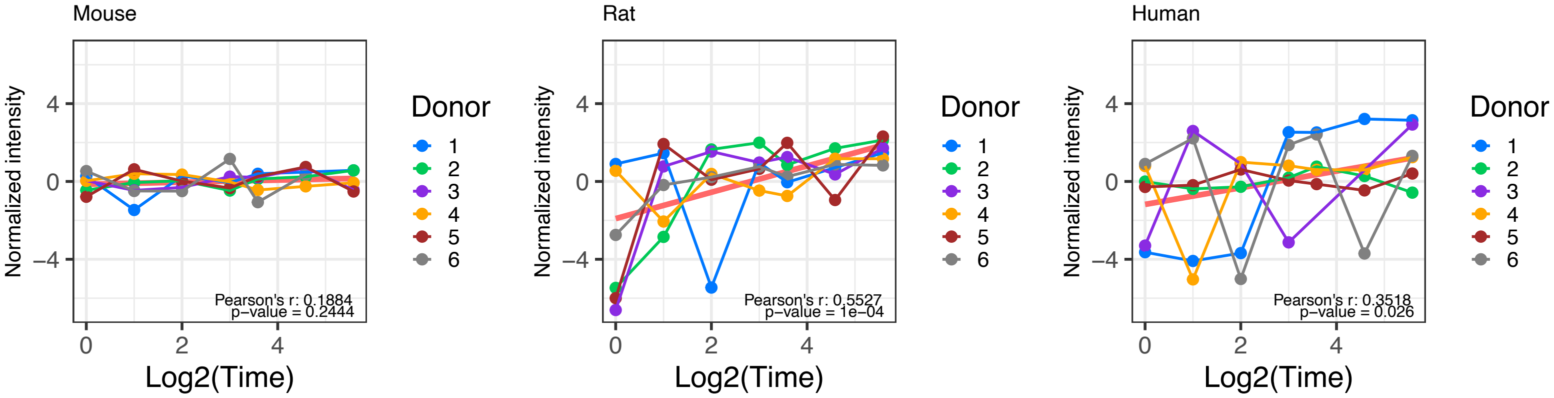

Glucosamine−6−phosphate

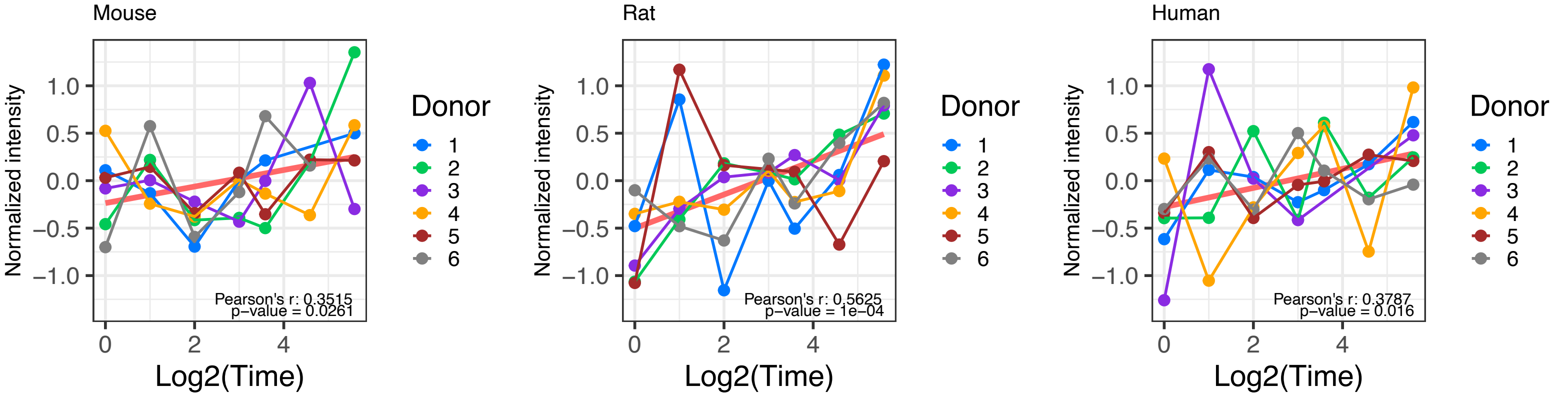

Glucose 6−phosphate

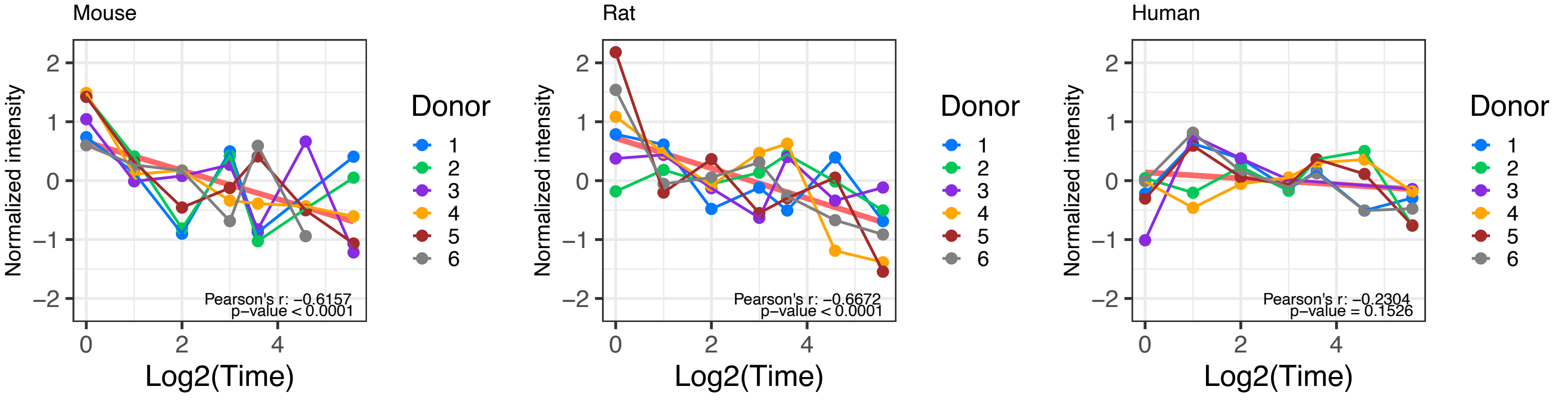

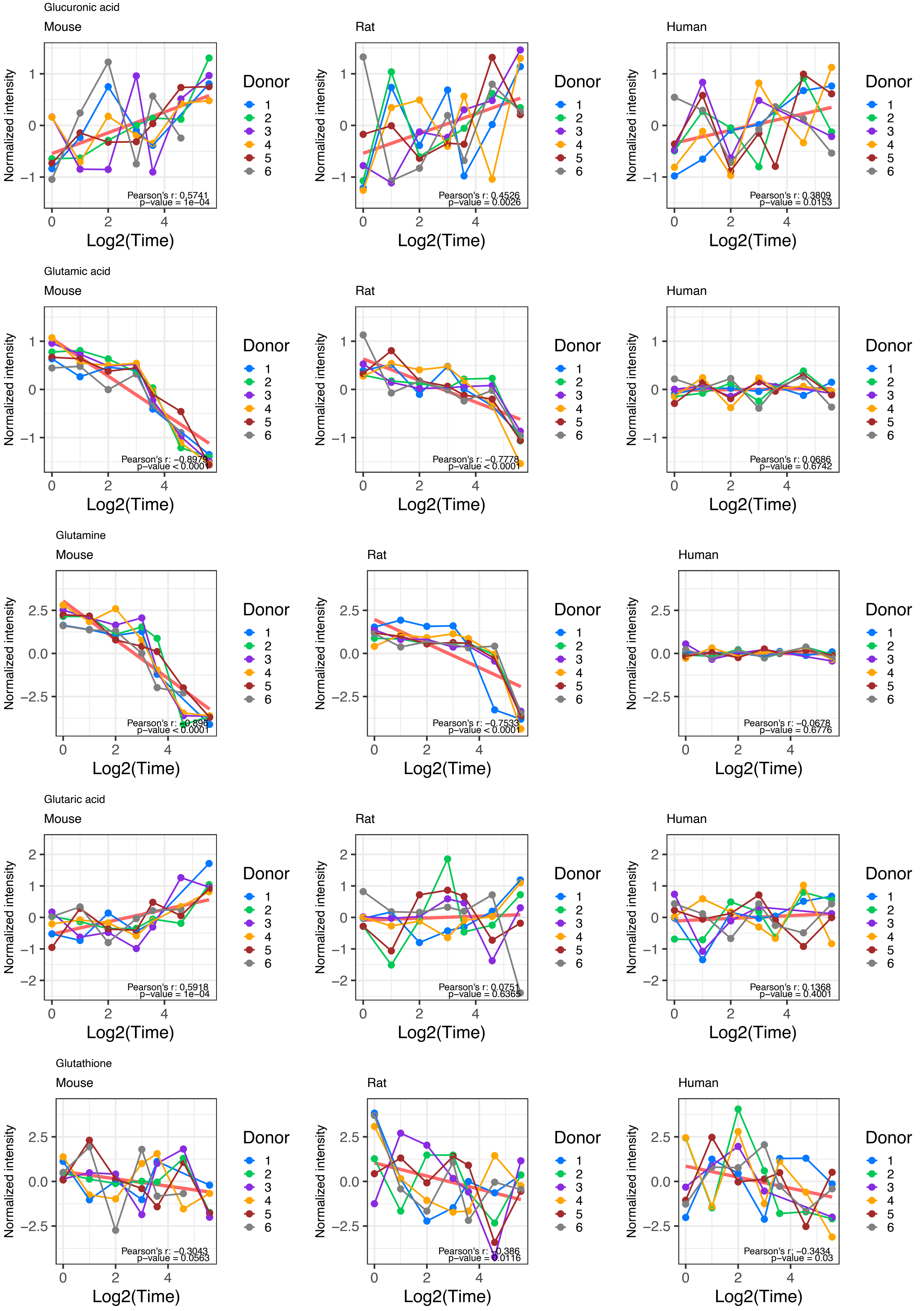
Glucuronic acid
Mouse
Pearson's r: 0.5741
p−value = 1e−04
Rat
Pearson's r: 0.4526
p−value = 0.0026
Human
Pearson's r: 0.3809
p−value = 0.0153
Glutamic acid
Mouse
Pearson's r: −0.8979
p−value < 0.0001
Rat
Pearson's r: −0.7778
p−value < 0.0001
Human
Pearson's r: 0.0686
p−value = 0.6742
Glutamine
Mouse
Pearson's r: −0.896
p−value < 0.0001
Rat
Pearson's r: −0.7533
p−value < 0.0001
Human
Pearson's r: −0.0678
p−value = 0.6776
Glutaric acid
Mouse
Pearson's r: 0.5918
p−value = 1e−04
Rat
Pearson's r: 0.0751
p−value = 0.6365
Human
Pearson's r: 0.1368
p−value = 0.4001
Glutathione
Mouse
Pearson's r: −0.3043
p−value = 0.0563
Rat
Pearson's r: −0.386
p−value = 0.0116
Human
Pearson's r: −0.3434
p−value = 0.03
Normalized intensity
Log2(Time)
Donor
1
2
3
4
5
6

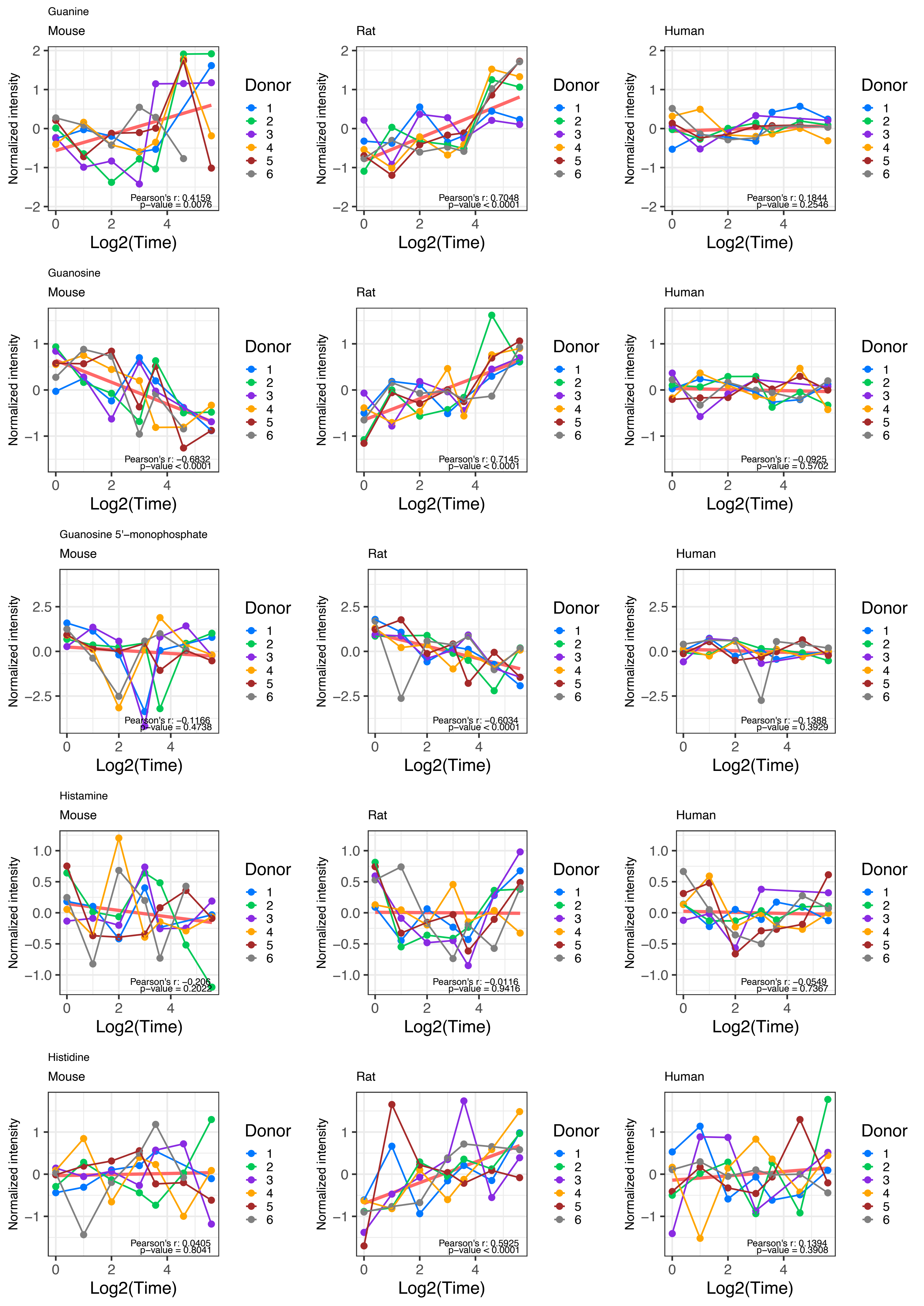

Guanine
Mouse
Rat
Human
Pearson's r: 0.4159
p−value = 0.0076
Pearson's r: 0.7048
p−value < 0.0001
Pearson's r: 0.1844
p−value = 0.2546
Guanosine
Mouse
Rat
Human
Pearson's r: −0.6832
p−value < 0.0001
Pearson's r: 0.7145
p−value < 0.0001
Pearson's r: −0.0925
p−value = 0.5702
Guanosine 5'−monophosphate
Mouse
Rat
Human
Pearson's r: −0.1166
p−value = 0.4738
Pearson's r: −0.6034
p−value < 0.0001
Pearson's r: −0.1388
p−value = 0.3929
Histamine
Mouse
Rat
Human
Pearson's r: −0.206
p−value = 0.2022
Pearson's r: −0.0116
p−value = 0.9416
Pearson's r: −0.0549
p−value = 0.7367
Histidine
Mouse
Rat
Human
Pearson's r: 0.0405
p−value = 0.8041
Pearson's r: 0.5925
p−value < 0.0001
Pearson's r: 0.1394
p−value = 0.3908
Normalized intensity
Log2(Time)
Donor
1
2
3
4
5
6

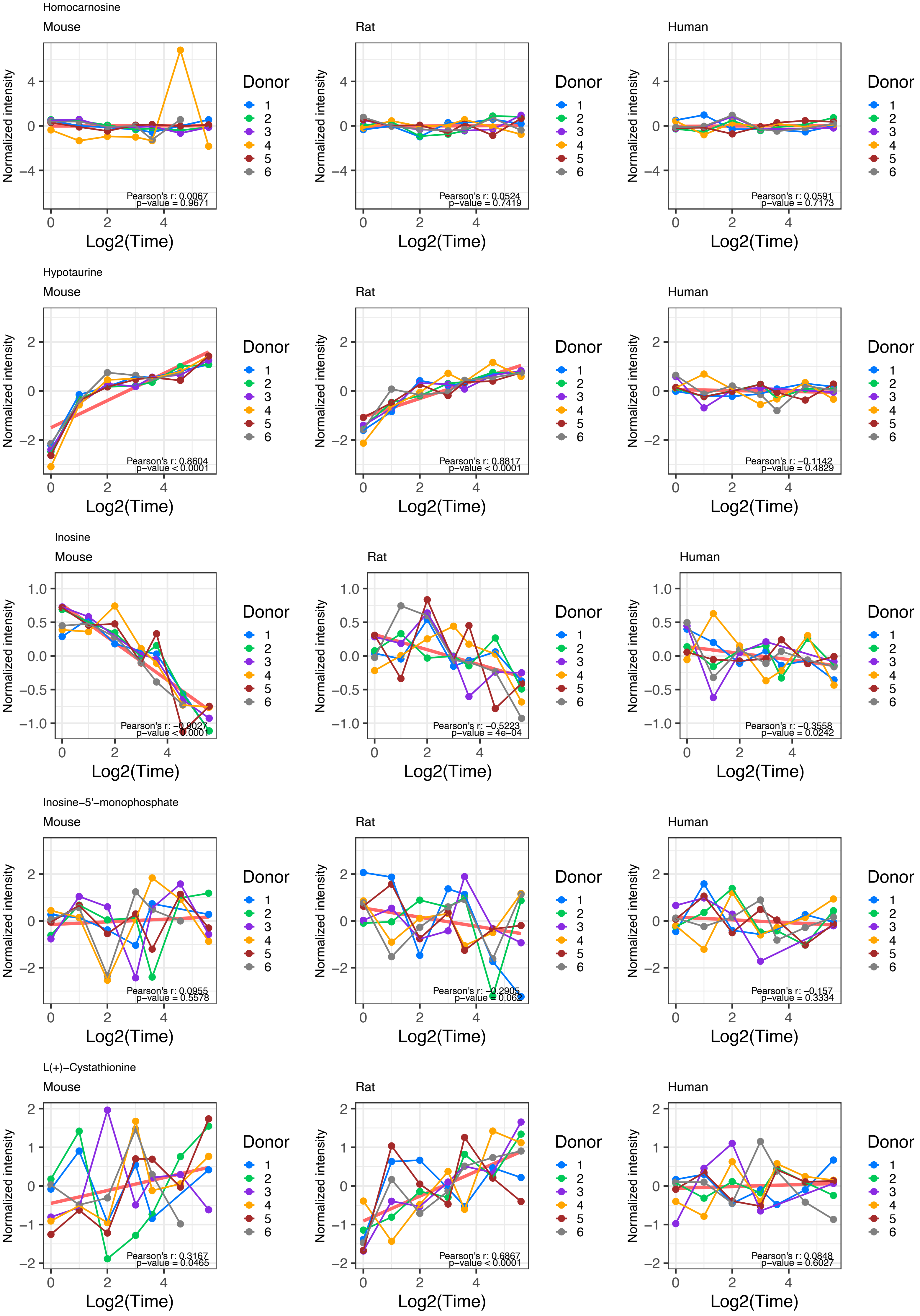

Homocarnosine
Mouse
Pearson's r: 0.0067
p−value = 0.9671
Rat
Pearson's r: 0.0524
p−value = 0.7419
Human
Pearson's r: 0.0591
p−value = 0.7173
Hypotaurine
Mouse
Pearson's r: 0.8604
p−value < 0.0001
Rat
Pearson's r: 0.8817
p−value < 0.0001
Human
Pearson's r: −0.1142
p−value = 0.4829
Inosine
Mouse
Pearson's r: −0.9027
p−value < 0.0001
Rat
Pearson's r: −0.5223
p−value = 4e−04
Human
Pearson's r: −0.3558
p−value = 0.0242
Inosine−5'−monophosphate
Mouse
Pearson's r: 0.0955
p−value = 0.5578
Rat
Pearson's r: −0.2905
p−value = 0.062
Human
Pearson's r: −0.157
p−value = 0.3334
L(+)−Cystathionine
Mouse
Pearson's r: 0.3167
p−value = 0.0465
Rat
Pearson's r: 0.6867
p−value < 0.0001
Human
Pearson's r: 0.0848
p−value = 0.6027
Normalized intensity
Log2(Time)
Donor
1
2
3
4
5
6

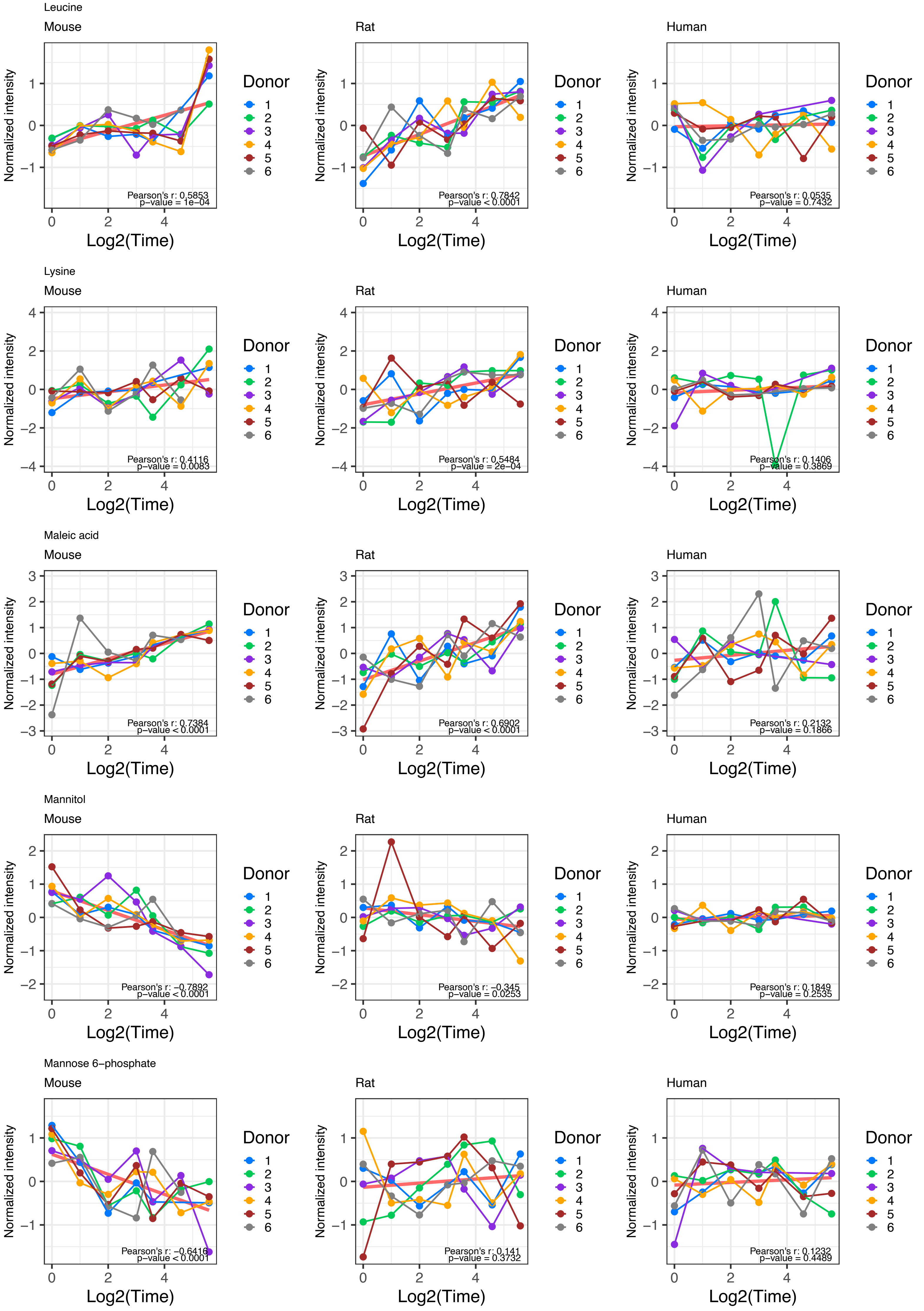
Leucine
Mouse
Pearson's r: 0.5853
p−value = 1e−04
Rat
Pearson's r: 0.7842
p−value < 0.0001
Human
Pearson's r: 0.0535
p−value = 0.7432
Lysine
Mouse
Pearson's r: 0.4116
p−value = 0.0083
Rat
Pearson's r: 0.5484
p−value = 2e−04
Human
Pearson's r: 0.1406
p−value = 0.3869
Maleic acid
Mouse
Pearson's r: 0.7384
p−value < 0.0001
Rat
Pearson's r: 0.6902
p−value < 0.0001
Human
Pearson's r: 0.2132
p−value = 0.1866
Mannitol
Mouse
Pearson's r: −0.7892
p−value < 0.0001
Rat
Pearson's r: −0.345
p−value = 0.0253
Human
Pearson's r: 0.1849
p−value = 0.2535
Mannose 6−phosphate
Mouse
Pearson's r: −0.6416
p−value < 0.0001
Rat
Pearson's r: 0.141
p−value = 0.3732
Human
Pearson's r: 0.1232
p−value = 0.4489
Normalized intensity
Log2(Time)
Donor
1
2
3
4
5
6

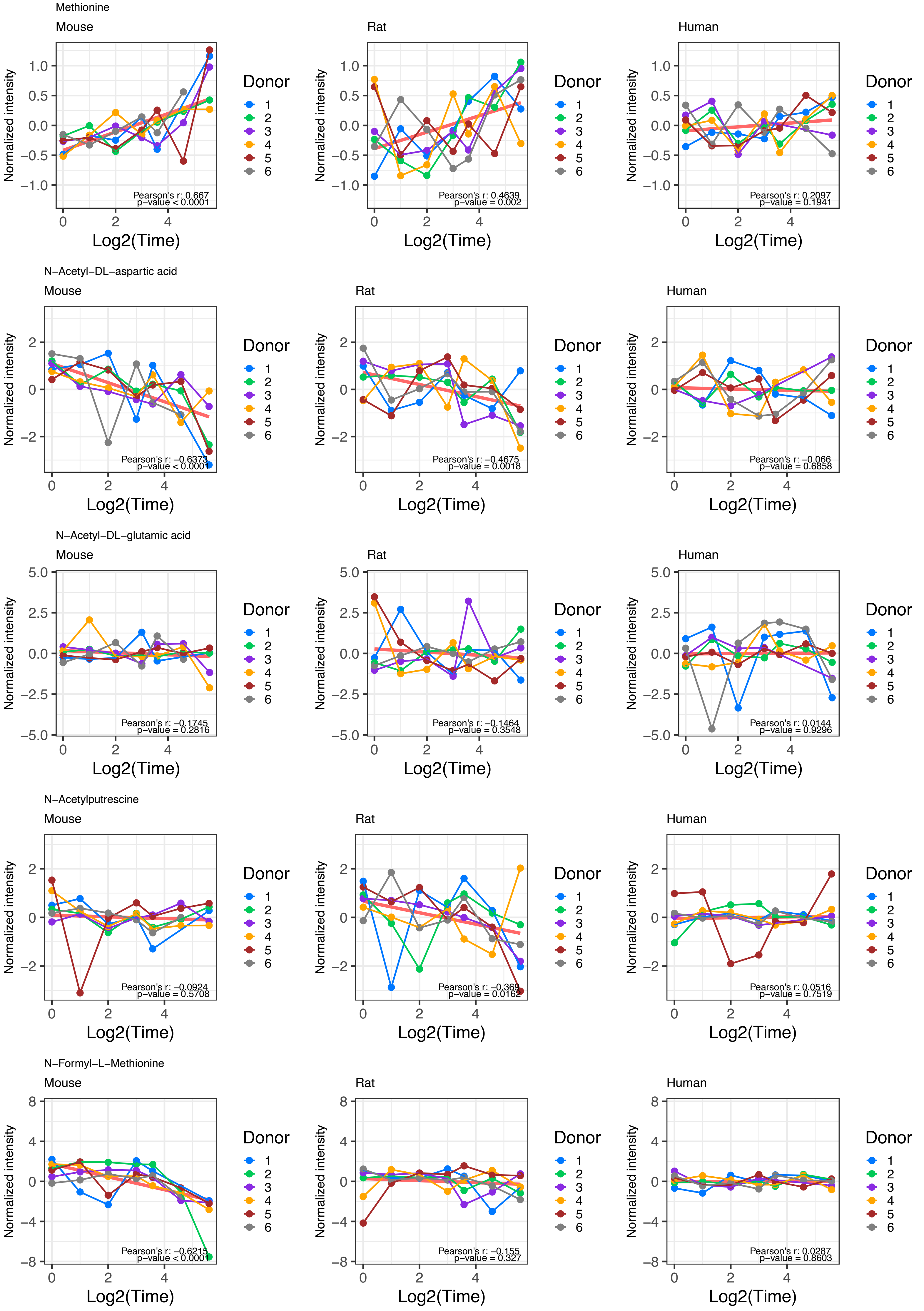

Methionine
Mouse
Rat
Human
Normalized intensity
Log2(Time)
Donor
1
2
3
4
5
6
Pearson's r: 0.667
p−value < 0.0001
Pearson's r: 0.4639
p−value = 0.002
Pearson's r: 0.2097
p−value = 0.1941
N−Acetyl−DL−aspartic acid
Pearson's r: −0.6373
p−value < 0.0001
Pearson's r: −0.4675
p−value = 0.0018
Pearson's r: −0.066
p−value = 0.6858
N−Acetyl−DL−glutamic acid
Pearson's r: −0.1745
p−value = 0.2816
Pearson's r: −0.1464
p−value = 0.3548
Pearson's r: 0.0144
p−value = 0.9296
N−Acetylputrescine
Pearson's r: −0.0924
p−value = 0.5708
Pearson's r: −0.369
p−value = 0.0162
Pearson's r: 0.0516
p−value = 0.7519
N−Formyl−L−Methionine
Pearson's r: −0.6215
p−value < 0.0001
Pearson's r: −0.155
p−value = 0.327
Pearson's r: 0.0287
p−value = 0.8603

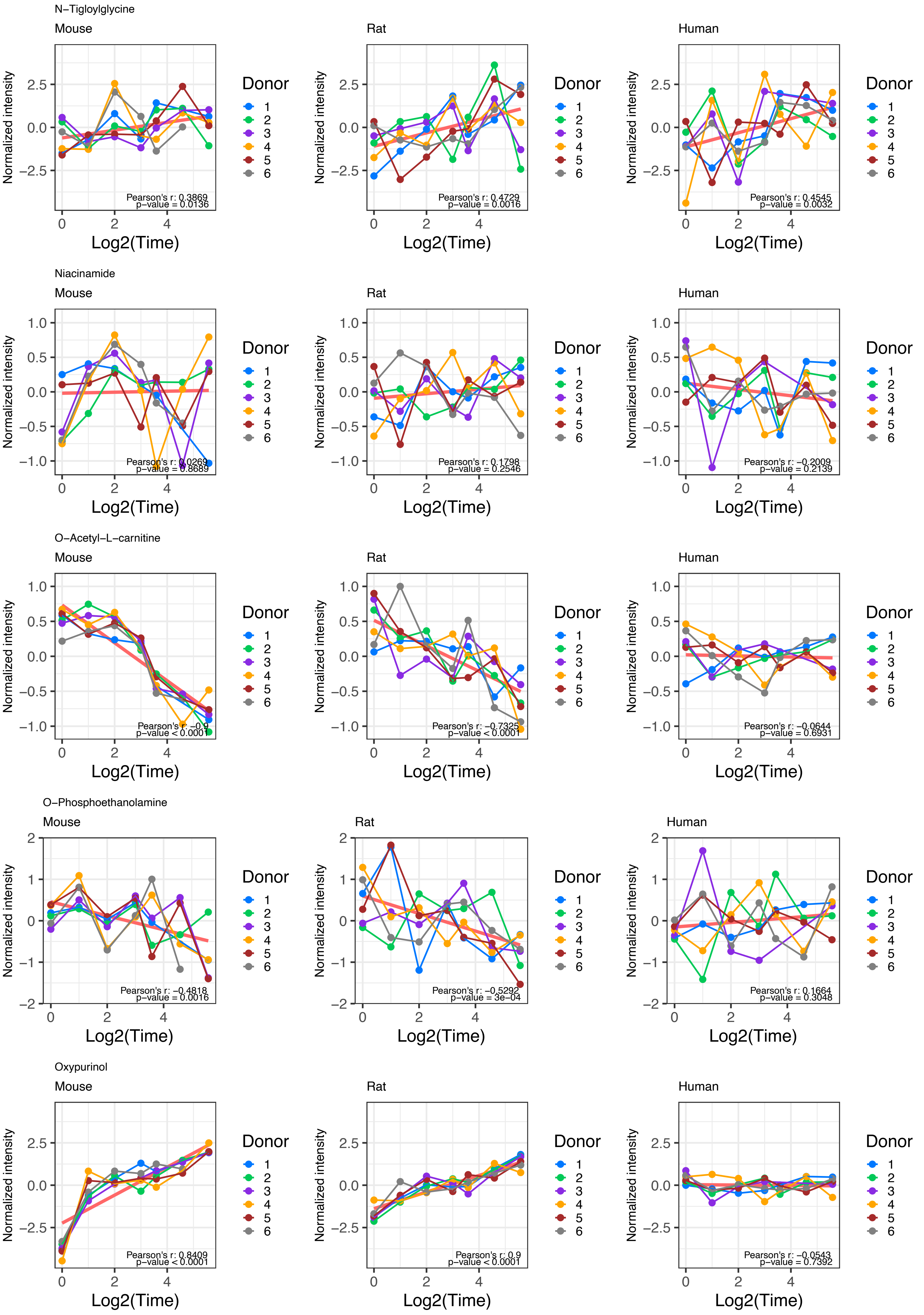
N−Tigloylglycine
Mouse
Pearson's r: 0.3869
p−value = 0.0136
Rat
Pearson's r: 0.4729
p−value = 0.0016
Human
Pearson's r: 0.4545
p−value = 0.0032
Niacinamide
Mouse
Pearson's r: 0.0269
p−value = 0.8689
Rat
Pearson's r: 0.1798
p−value = 0.2546
Human
Pearson's r: −0.2009
p−value = 0.2139
O−Acetyl−L−carnitine
Mouse
Pearson's r: −0.9
p−value < 0.0001
Rat
Pearson's r: −0.7325
p−value < 0.0001
Human
Pearson's r: −0.0644
p−value = 0.6931
O−Phosphoethanolamine
Mouse
Pearson's r: −0.4818
p−value = 0.0016
Rat
Pearson's r: −0.5292
p−value = 3e−04
Human
Pearson's r: 0.1664
p−value = 0.3048
Oxypurinol
Mouse
Pearson's r: 0.8409
p−value < 0.0001
Rat
Pearson's r: 0.9
p−value < 0.0001
Human
Pearson's r: −0.0543
p−value = 0.7392
Normalized intensity
Log2(Time)
Donor
1
2
3
4
5
6

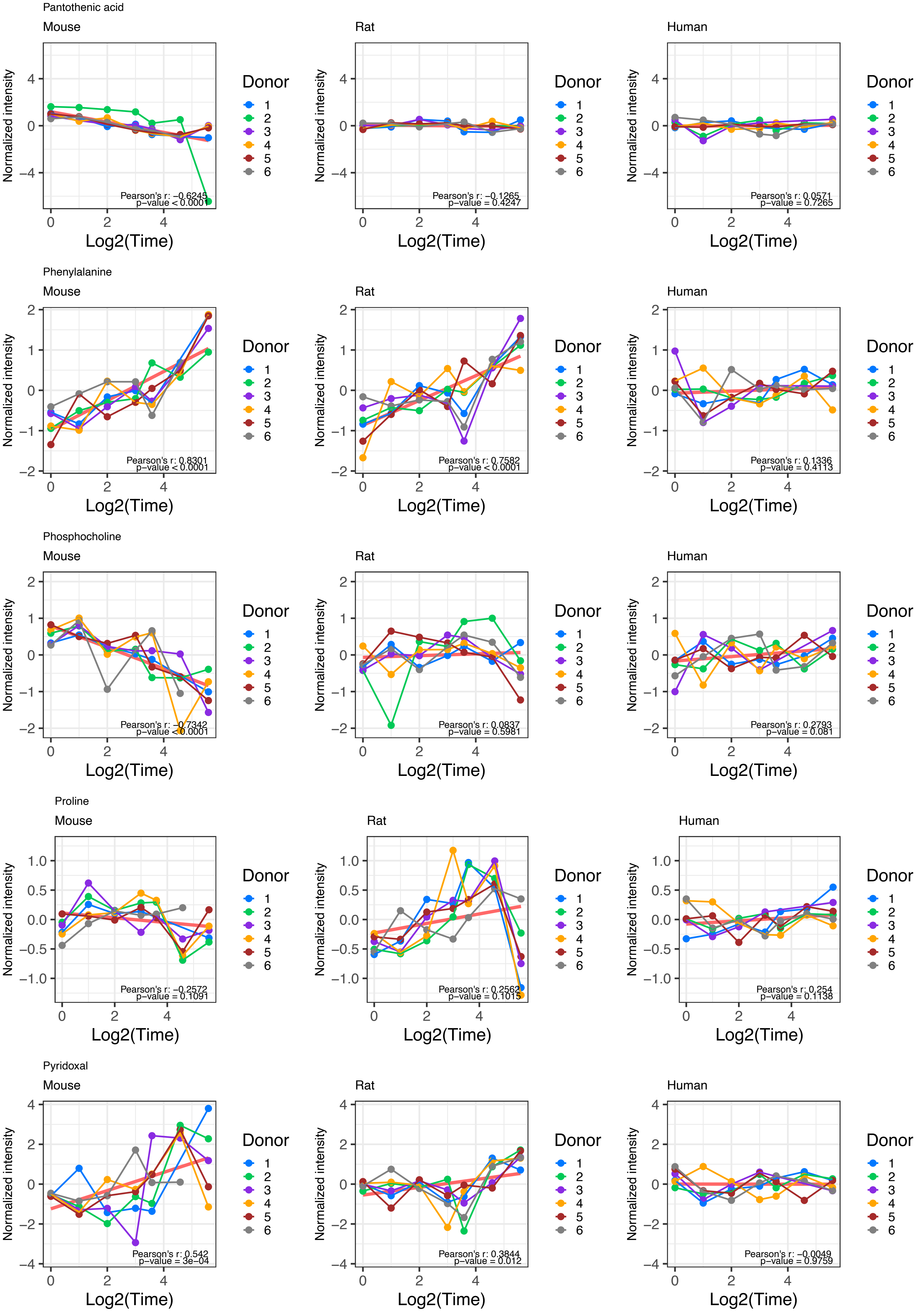
Pantothenic acid
Mouse
Rat
Human
Normalized intensity
Log2(Time)
Donor
1
2
3
4
5
6
Pearson's r: −0.6245
p−value < 0.0001
Pearson's r: −0.1265
p−value = 0.4247
Pearson's r: 0.0571
p−value = 0.7265
Phenylalanine
Mouse
Rat
Human
Pearson's r: 0.8301
p−value < 0.0001
Pearson's r: 0.7582
p−value < 0.0001
Pearson's r: 0.1336
p−value = 0.4113
Phosphocholine
Mouse
Rat
Human
Pearson's r: −0.7342
p−value < 0.0001
Pearson's r: 0.0837
p−value = 0.5981
Pearson's r: 0.2793
p−value = 0.081
Proline
Mouse
Rat
Human
Pearson's r: −0.2572
p−value = 0.1091
Pearson's r: 0.2562
p−value = 0.1015
Pearson's r: 0.254
p−value = 0.1138
Pyridoxal
Mouse
Rat
Human
Pearson's r: 0.542
p−value = 3e−04
Pearson's r: 0.3844
p−value = 0.012
Pearson's r: −0.0049
p−value = 0.9759

rac−Glycerol 3−phosphoate

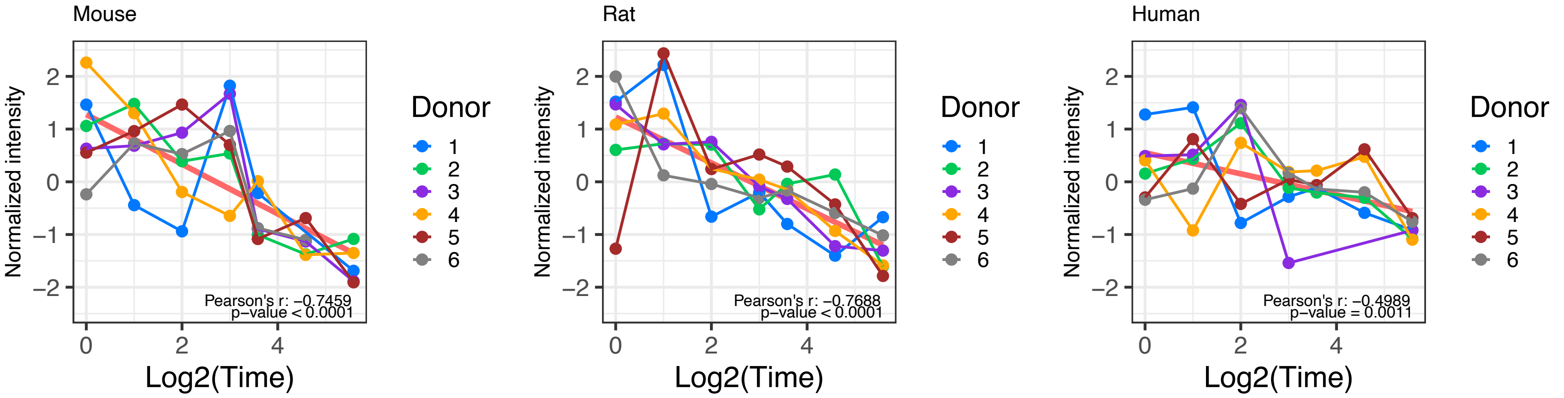

Ribose 5−phosphate

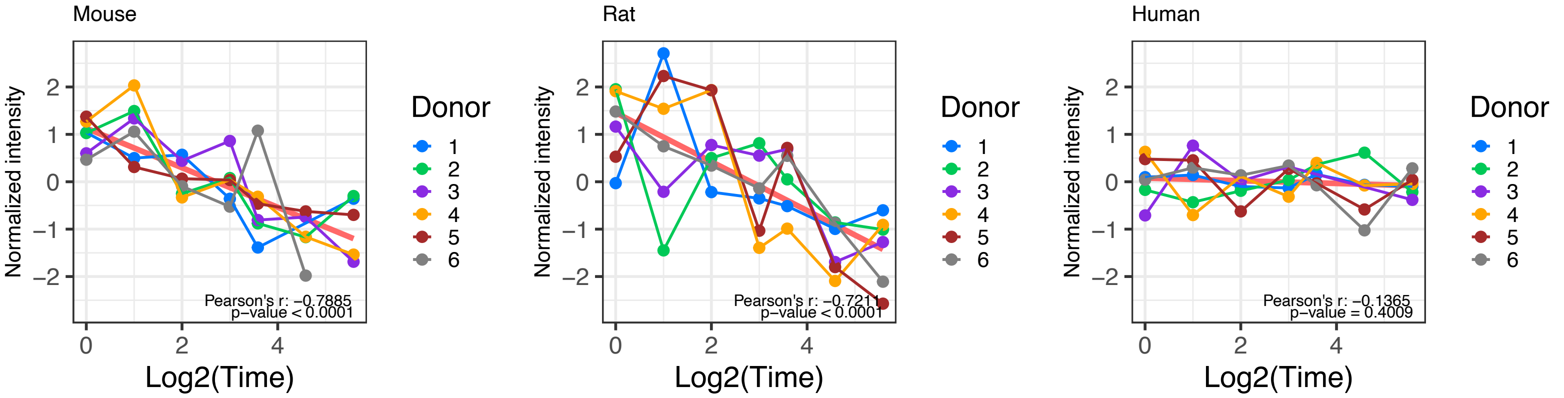

S−Adenosyl−L−homocysteine

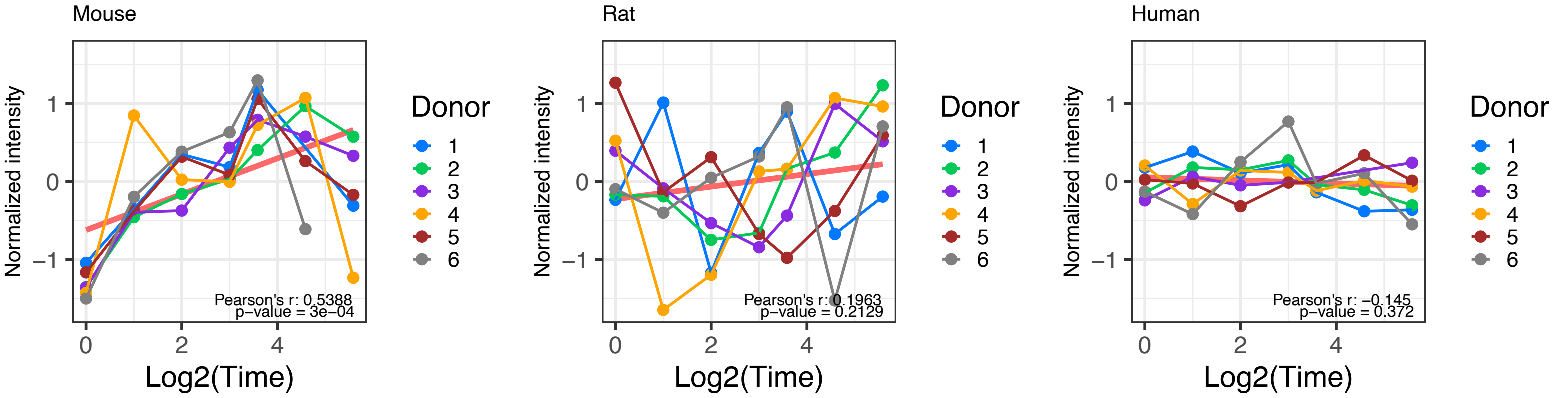

Saccharopine

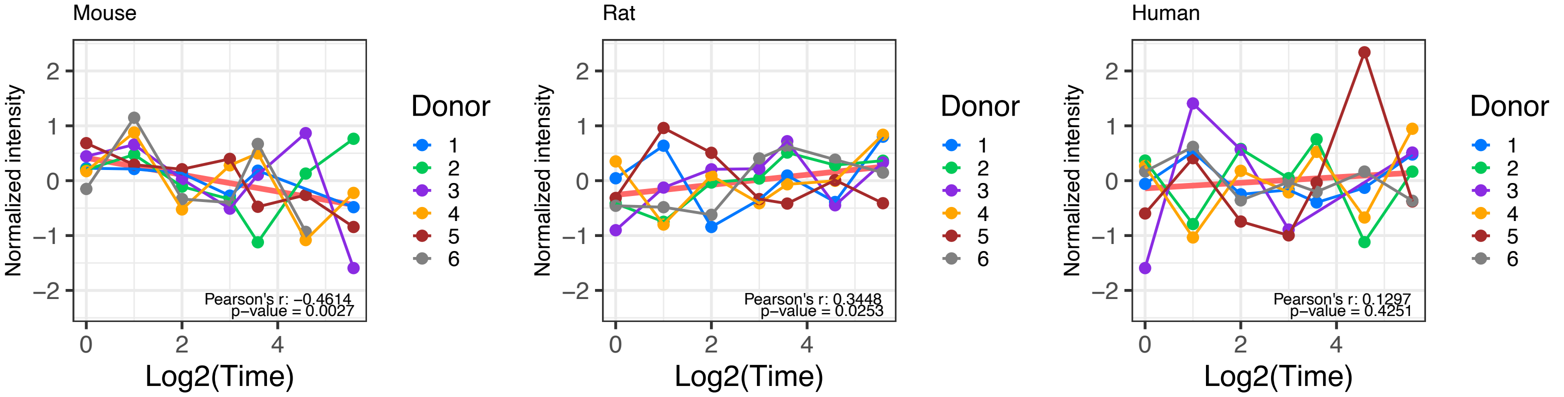

Sebacic acid

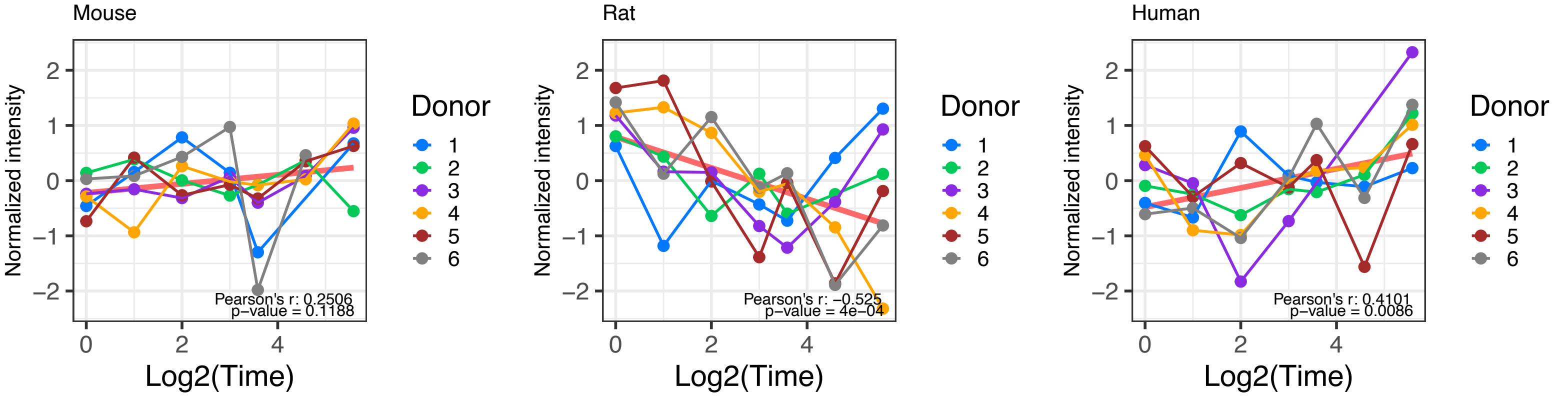

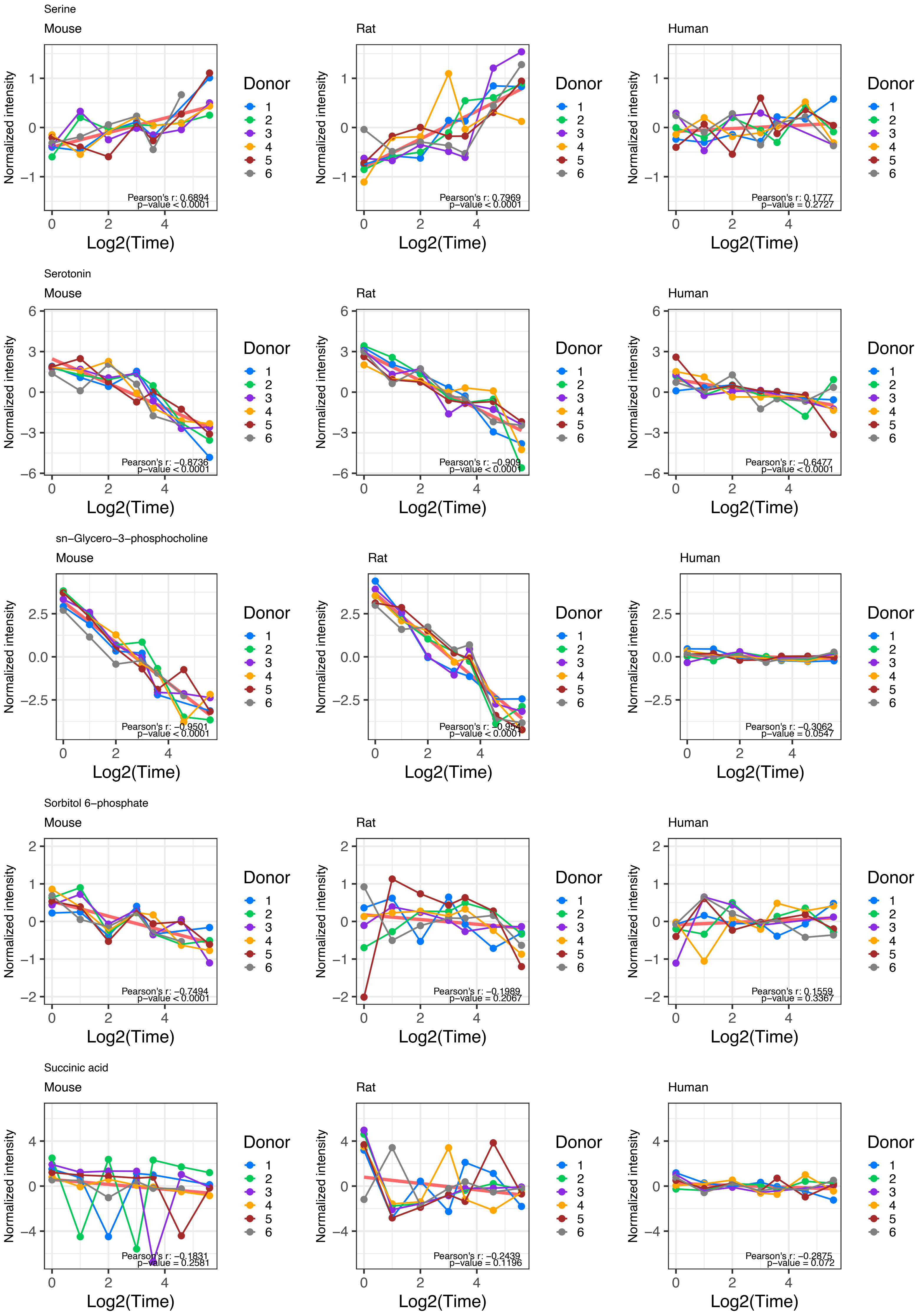
Serine
Mouse
Pearson's r: 0.6894
p−value < 0.0001
Rat
Pearson's r: 0.7969
p−value < 0.0001
Human
Pearson's r: 0.1777
p−value = 0.2727
Serotonin
Mouse
Pearson's r: −0.8736
p−value < 0.0001
Rat
Pearson's r: −0.909
p−value < 0.0001
Human
Pearson's r: −0.6477
p−value < 0.0001
sn−Glycero−3−phosphocholine
Mouse
Pearson's r: −0.9501
p−value < 0.0001
Rat
Pearson's r: −0.954
p−value < 0.0001
Human
Pearson's r: −0.3062
p−value = 0.0547
Sorbitol 6−phosphate
Mouse
Pearson's r: −0.7494
p−value < 0.0001
Rat
Pearson's r: −0.1989
p−value = 0.2067
Human
Pearson's r: 0.1559
p−value = 0.3367
Succinic acid
Mouse
Pearson's r: −0.1831
p−value = 0.2581
Rat
Pearson's r: −0.2439
p−value = 0.1196
Human
Pearson's r: −0.2875
p−value = 0.072
Normalized intensity
Log2(Time)
Donor
1
2
3
4
5
6

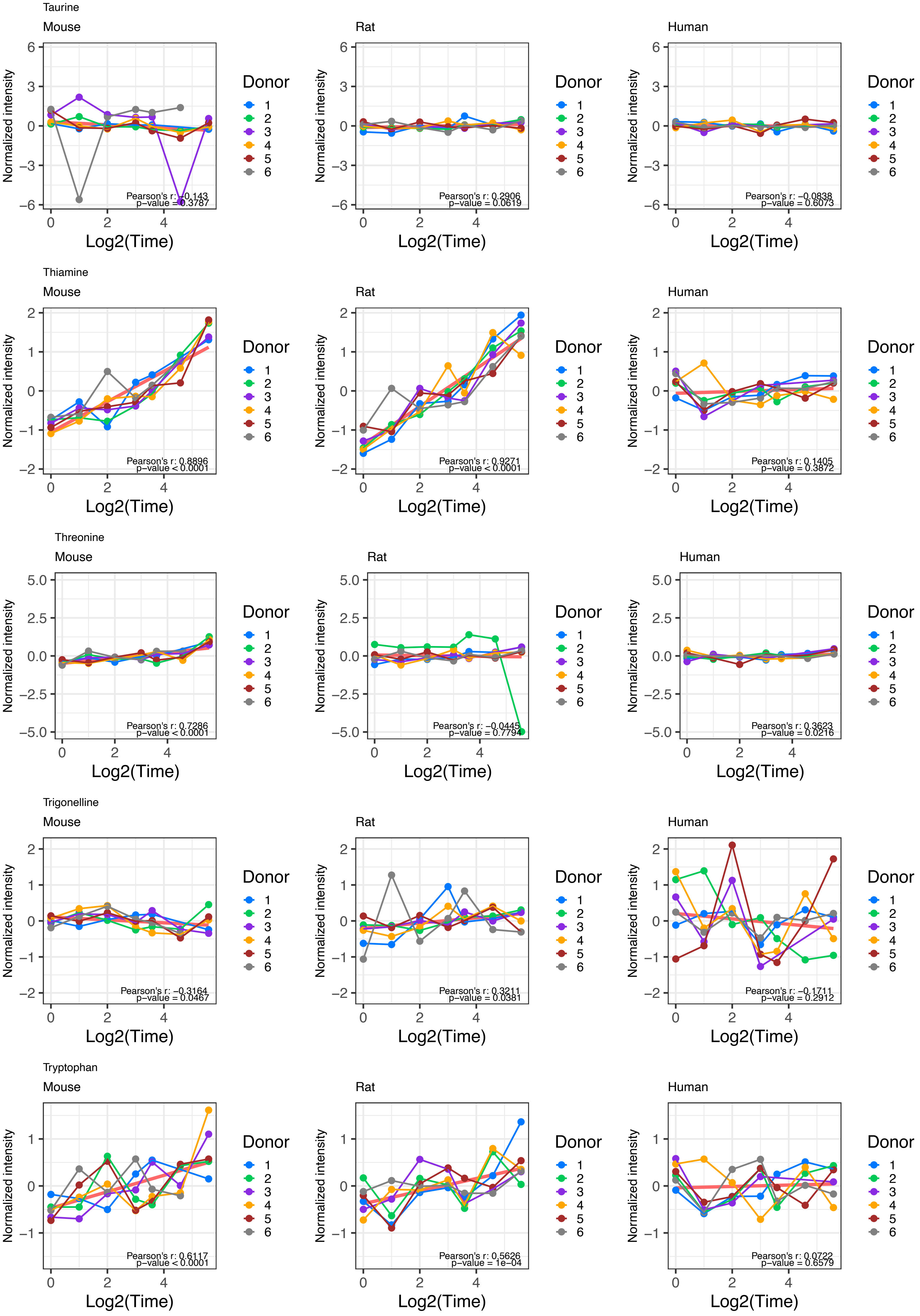
Taurine
Mouse
Rat
Human
Normalized intensity
Log2(Time)
Donor
1
2
3
4
5
6
Pearson's r: −0.143
p−value = 0.3787
Pearson's r: 0.2906
p−value = 0.0619
Pearson's r: −0.0838
p−value = 0.6073
Thiamine
Pearson's r: 0.8896
p−value < 0.0001
Pearson's r: 0.9271
p−value < 0.0001
Pearson's r: 0.1405
p−value = 0.3872
Threonine
Pearson's r: 0.7286
p−value < 0.0001
Pearson's r: −0.0445
p−value = 0.7794
Pearson's r: 0.3623
p−value = 0.0216
Trigonelline
Pearson's r: −0.3164
p−value = 0.0467
Pearson's r: 0.3211
p−value = 0.0381
Pearson's r: −0.1711
p−value = 0.2912
Tryptophan
Pearson's r: 0.6117
p−value < 0.0001
Pearson's r: 0.5626
p−value = 1e−04
Pearson's r: 0.0722
p−value = 0.6579

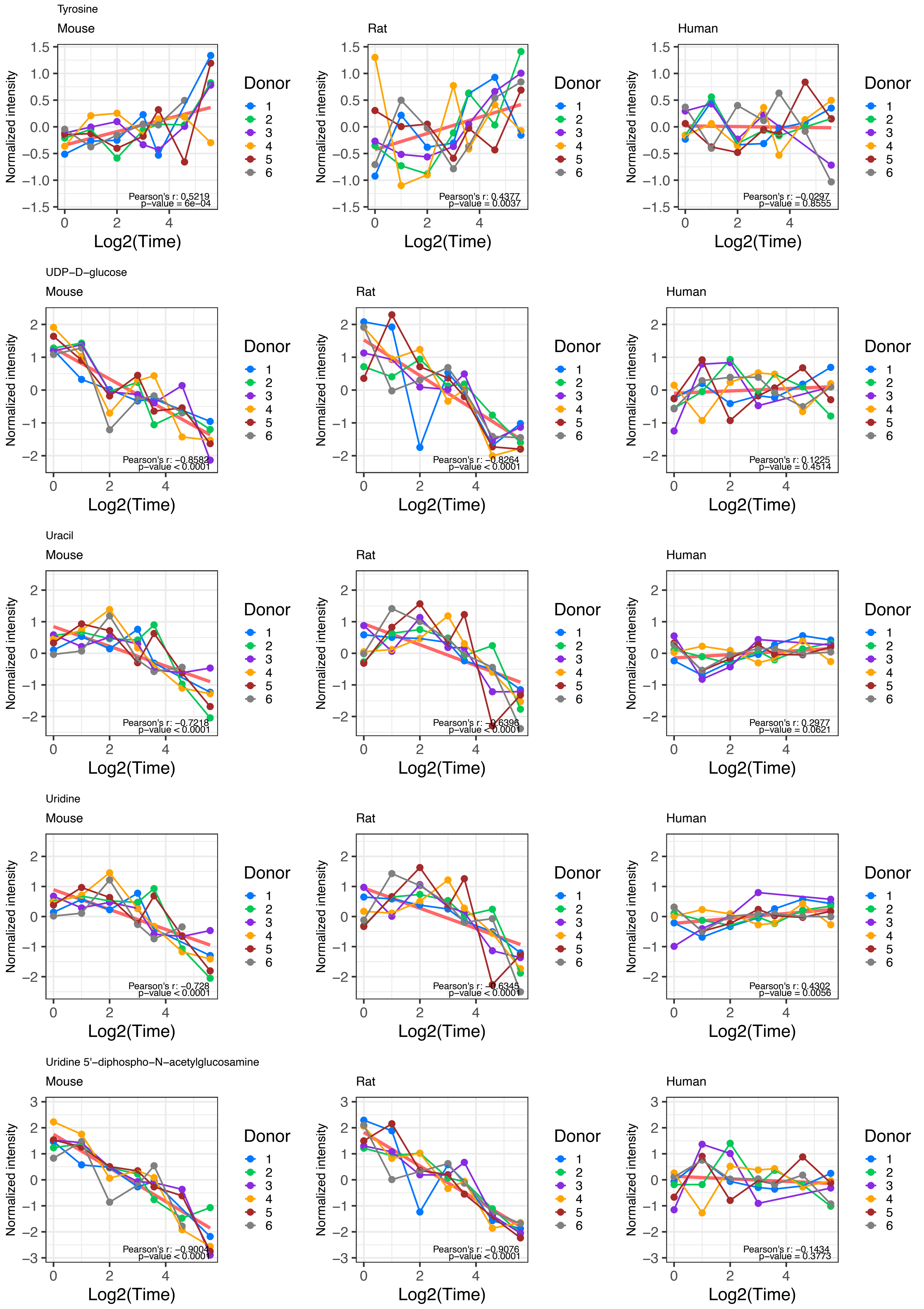
Tyrosine
Mouse
Rat
Human
Normalized intensity
Log2(Time)
Donor
1
2
3
4
5
6
Pearson's r: 0.5219
p−value = 6e−04
Pearson's r: 0.4377
p−value = 0.0037
Pearson's r: −0.0297
p−value = 0.8555
UDP−D−glucose
Pearson's r: −0.8582
p−value < 0.0001
Pearson's r: −0.8264
p−value < 0.0001
Pearson's r: 0.1225
p−value = 0.4514
Uracil
Pearson's r: −0.7218
p−value < 0.0001
Pearson's r: −0.6396
p−value < 0.0001
Pearson's r: 0.2977
p−value = 0.0621
Uridine
Pearson's r: −0.728
p−value < 0.0001
Pearson's r: −0.6345
p−value < 0.0001
Pearson's r: 0.4302
p−value = 0.0056
Uridine 5'−diphospho−N−acetylglucosamine
Pearson's r: −0.9004
p−value < 0.0001
Pearson's r: −0.9076
p−value < 0.0001
Pearson's r: −0.1434
p−value = 0.3773

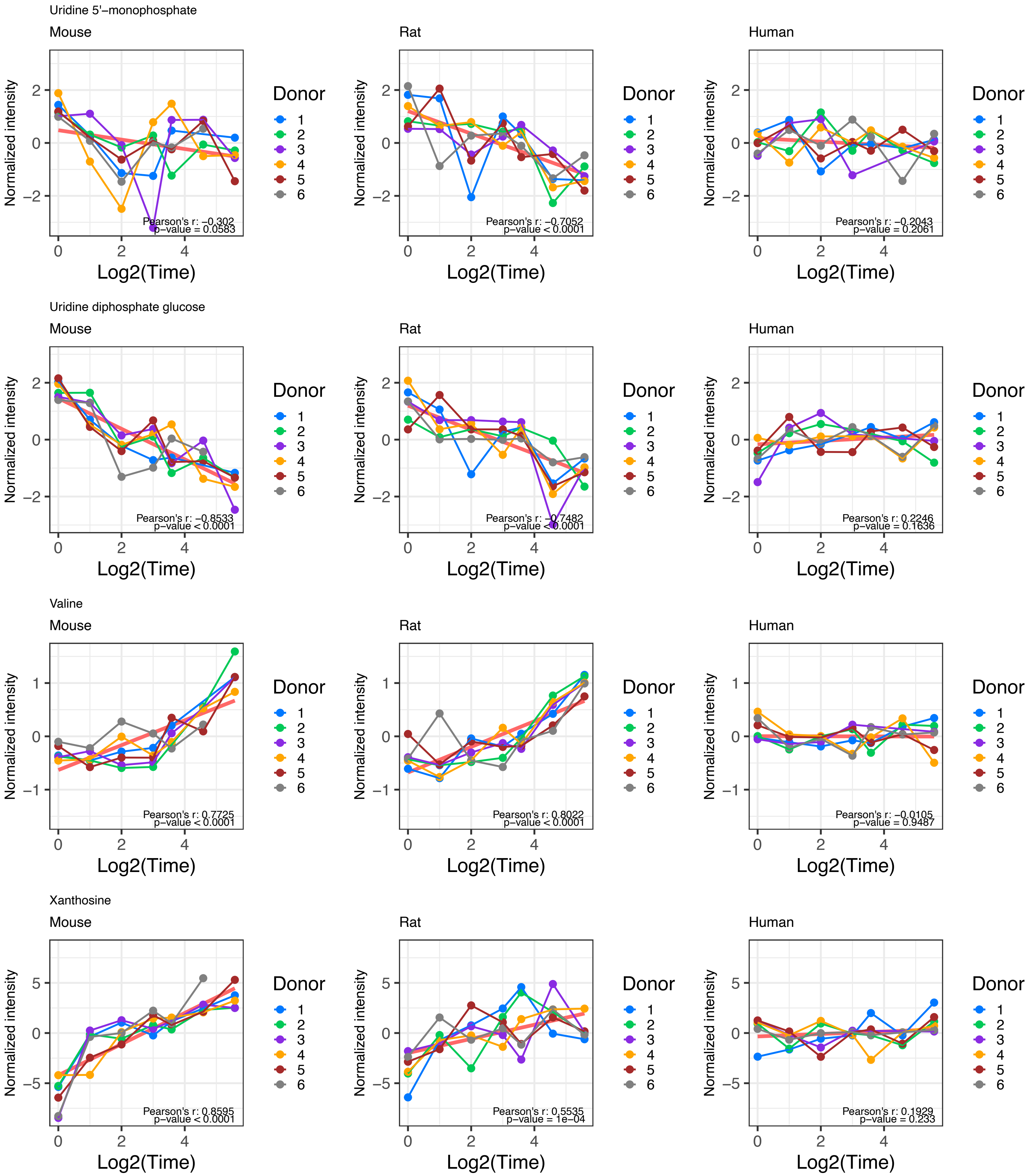
Uridine 5'−monophosphate
Mouse
Rat
Human
Normalized intensity
Log2(Time)
Donor
1
2
3
4
5
6
Pearson's r: −0.302
p−value = 0.0583
Pearson's r: −0.7052
p−value < 0.0001
Pearson's r: −0.2043
p−value = 0.2061
Uridine diphosphate glucose
Pearson's r: −0.8533
p−value < 0.0001
Pearson's r: −0.7482
p−value < 0.0001
Pearson's r: 0.2246
p−value = 0.1636
Valine
Pearson's r: 0.7725
p−value < 0.0001
Pearson's r: 0.8022
p−value < 0.0001
Pearson's r: −0.0105
p−value = 0.9487
Xanthosine
Pearson's r: 0.8595
p−value < 0.0001
Pearson's r: 0.5535
p−value = 1e−04
Pearson's r: 0.1929
p−value = 0.233

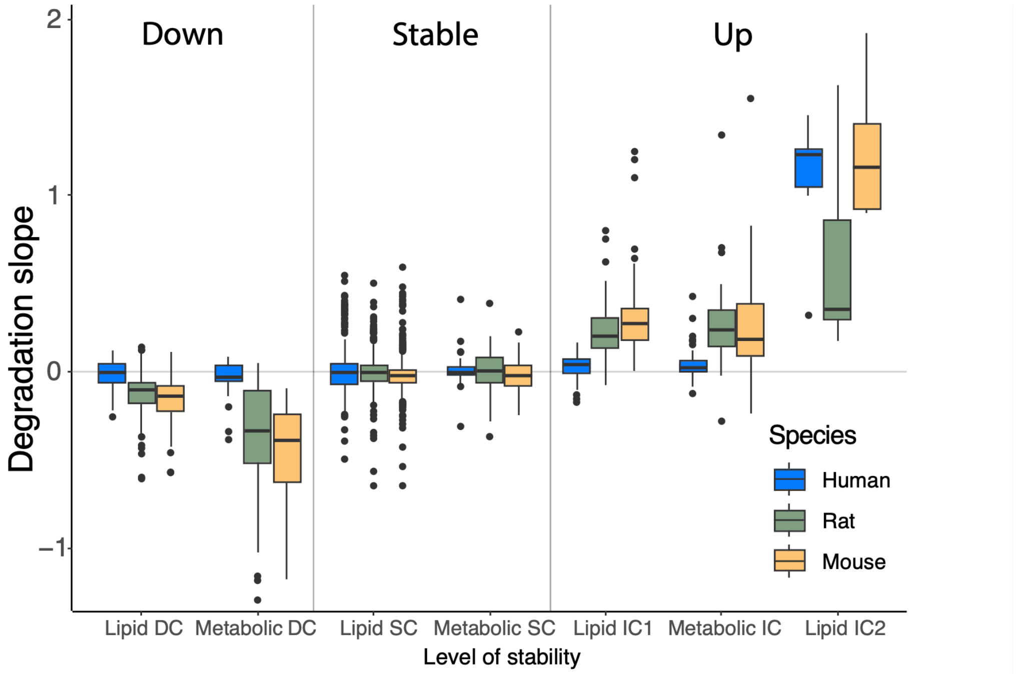

Figure S3. Distributions of lipid and polar metabolite postmortem alteration rates in each species for each of the clusters.

Current study	(Melissa Scholefield, 2020) Down	(Melissa Scholefield, 2020) Up
Up	Threonine	Aspartic acid, Tyrosine, Phenylalanine, Valine, Methionine, Proline, GABA, Leucine, Serine
Down	Glycerol-3-phosphate, Uridine	Hydroxybutyric acid, Pantothenic acid, Uracil, Ribose-5-phosphate

p-value (Fisher's test) = 0.035

Figure S4. Comparison of postmortem metabolite level changes reported both in the current study and in [31]. Shown is the contingency table displaying metabolites falling into each of the four quarters with Fisher exact test p-value based on this table marked below.

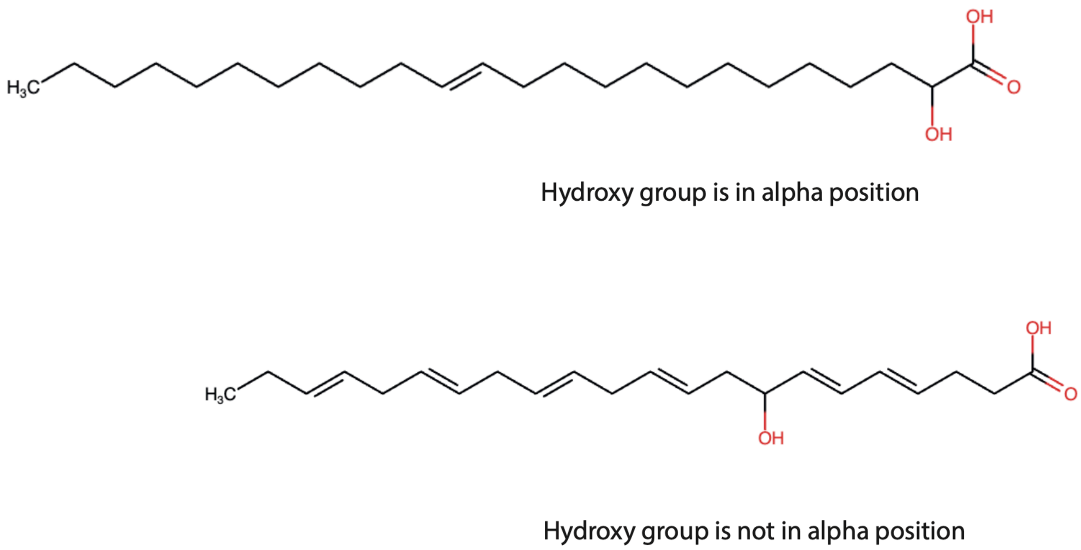

Figure S5. Structural comparison of fatty acids differing in their postmortem stability due to their hydroxy group position: the top molecule features a hydroxy group in the alpha position, whereas in the bottom molecule the hydroxy group is located elsewhere.
